# Combining Passive Sampling with Suspect and Nontarget Screening to Characterize Organic Micropollutants in Streams Draining Mixed-Use Watersheds

**DOI:** 10.1021/acs.est.2c02938

**Published:** 2022-11-04

**Authors:** Shiru Wang, Ruta Basijokaite, Bethany L. Murphy, Christa A. Kelleher, Teng Zeng

**Affiliations:** † Department of Civil and Environmental Engineering, Syracuse University, 151 Link Hall, Syracuse, New York 13244, United States; ‡ Department of Earth and Environmental Sciences, Syracuse University, 204 Heroy Geology Laboratory, Syracuse, New York 13244, United States

**Keywords:** passive sampling, high-resolution mass spectrometry, watershed attribute, field sampling rate

## Abstract

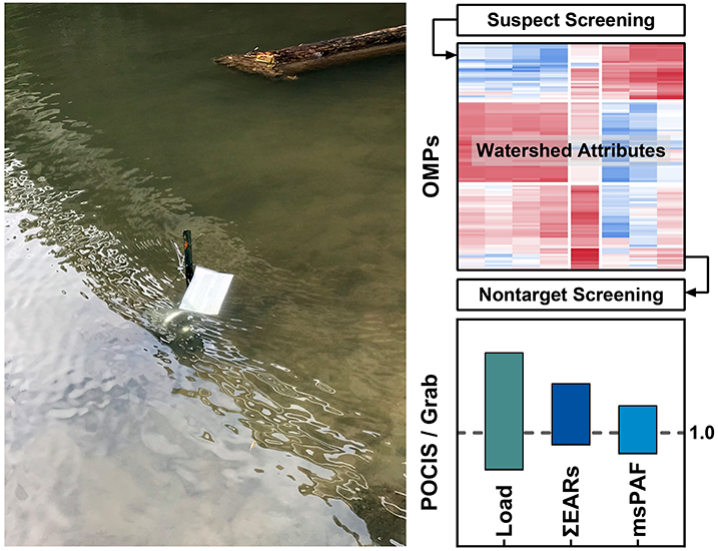

Organic micropollutants (OMPs) represent an anthropogenic stressor on stream ecosystems. In this work, we combined passive sampling with suspect and nontarget screening enabled by liquid chromatography–high-resolution mass spectrometry to characterize complex mixtures of OMPs in streams draining mixed-use watersheds. Suspect screening identified 122 unique OMPs for target quantification in polar organic chemical integrative samplers (POCIS) and grab samples collected from 20 stream sites in upstate New York over two sampling seasons. Hierarchical clustering established the co-occurrence profiles of OMPs in connection with watershed attributes indicative of anthropogenic influences. Nontarget screening leveraging the time-integrative nature of POCIS and the cross-site variability in watershed attributes prioritized and confirmed 11 additional compounds that were ubiquitously present in monitored streams. Field sampling rates for 37 OMPs that simultaneously occurred in POCIS and grab samples spanned the range of 0.02 to 0.22 L/d with a median value of 0.07 L/d. Comparative analyses of the daily average loads, cumulative exposure–activity ratios, and multi-substance potentially affected fractions supported the feasibility of complementing grab sampling with POCIS for OMP load estimation and screening-level risk assessments. Overall, this work demonstrated a multi-watershed sampling and screening approach that can be adapted to assess OMP contamination in streams across landscapes.

## Abstract 2

Organic micropollutants in streams draining mixed-use watersheds are characterized by passive sampling coupled with suspect and nontarget screening.

## Introduction

Organic micropollutants (OMPs) comprise a complex cocktail of synthetic organic chemicals (e.g., pharmaceuticals, pesticides, household and industrial chemicals) and their transformation products (TPs)^1^ that originate from diverse point and diffuse sources. Over the past two decades, the spatiotemporal heterogeneity of OMP occurrence in U.S. streams has been extensively documented by reconnaissance efforts led by the U.S. Geological Survey (USGS) and the U.S. Environmental Protection Agency.^2−7^ Watershed characteristics (e.g., soil properties), climate conditions (e.g., precipitation events), anthropogenic factors (e.g., land use patterns), and their interactions all contribute to the vulnerability of streams to OMP contamination.^8−10^ OMPs may impair stream ecosystem functioning because of their potential to cause adverse effects on the survival, growth, or reproduction of nontarget aquatic organisms.^11,12^ Combining integrated sampling and analytical efforts with evidence-based ecotoxicological studies is thus essential to improving the exposure and effect assessments of OMPs in streams.^13,14^

Considering the widespread occurrence and ecological relevance of OMPs, numerous studies have leveraged disk-based or thin-films passive sampling devices, such as the polar organic chemical integrative sampler (POCIS),^15^ as an alternative means to traditional grab or composite sampling for time-integrative monitoring of OMPs in streams and other aquatic environments.^16^ Many POCIS-based investigations have focused on laboratory calibration and field evaluation to gain insights into compound-specific (e.g., octanol–water partition coefficients^17^) or exposure-specific effects (e.g., hydrodynamic conditions, temperature, fouling^18−22^) on the uptake rate control and aquatic exposure assessment during passive sampling. Other efforts utilizing POCIS have sought to establish the concentration profiles and mass flows of OMPs (e.g., biocides,^23,24^ human-use pharmaceuticals,^25,26^ veterinary drugs^27,28^), quantify the attenuation rates of OMPs,^29^ or assess the biological potency (e.g., estrogenicity^30,31^) associated with OMPs in streams. Collectively, prior work underscores the key benefits of incorporating passive samplers into OMP research and highlights unique methodological considerations for passive sampling in environmental monitoring.

Our specific objectives of this study were (i) to perform suspect screening (enabled by liquid chromatography–high-resolution mass spectrometry (LC-HRMS)) and source-related clustering of OMPs in POCIS and grab samples collected from 20 sites on streams draining mixed-use watersheds in upstate New York; (ii) to develop a nontarget screening approach for prioritization and identification of OMPs by examining the connection between chemical features in POCIS and watershed attributes; and (iii) to evaluate the comparability of POCIS and grab sampling for OMP load estimation and screening-level risk assessments across stream sites. Only a few recent studies have explored the *in situ* enrichment feature afforded by passive samplers for suspect or nontarget screening of OMPs in mixed-use watersheds.^32−36^ Our work stands in the gap between studies that focused on the development of qualitative screening workflows^32,35,36^ and the spatiotemporal profiling of passive sampler data^33−35^ with the goal of applying a multi-watershed sampling and screening framework to comparatively assess OMP contamination in streams.

## Materials and Methods

Chemical sources and sampling supplies are detailed in the Supporting Information. OMP reference standards and isotope-labeled internal standards are listed in Table S1.

### Field Sampling

**Figure 1:**
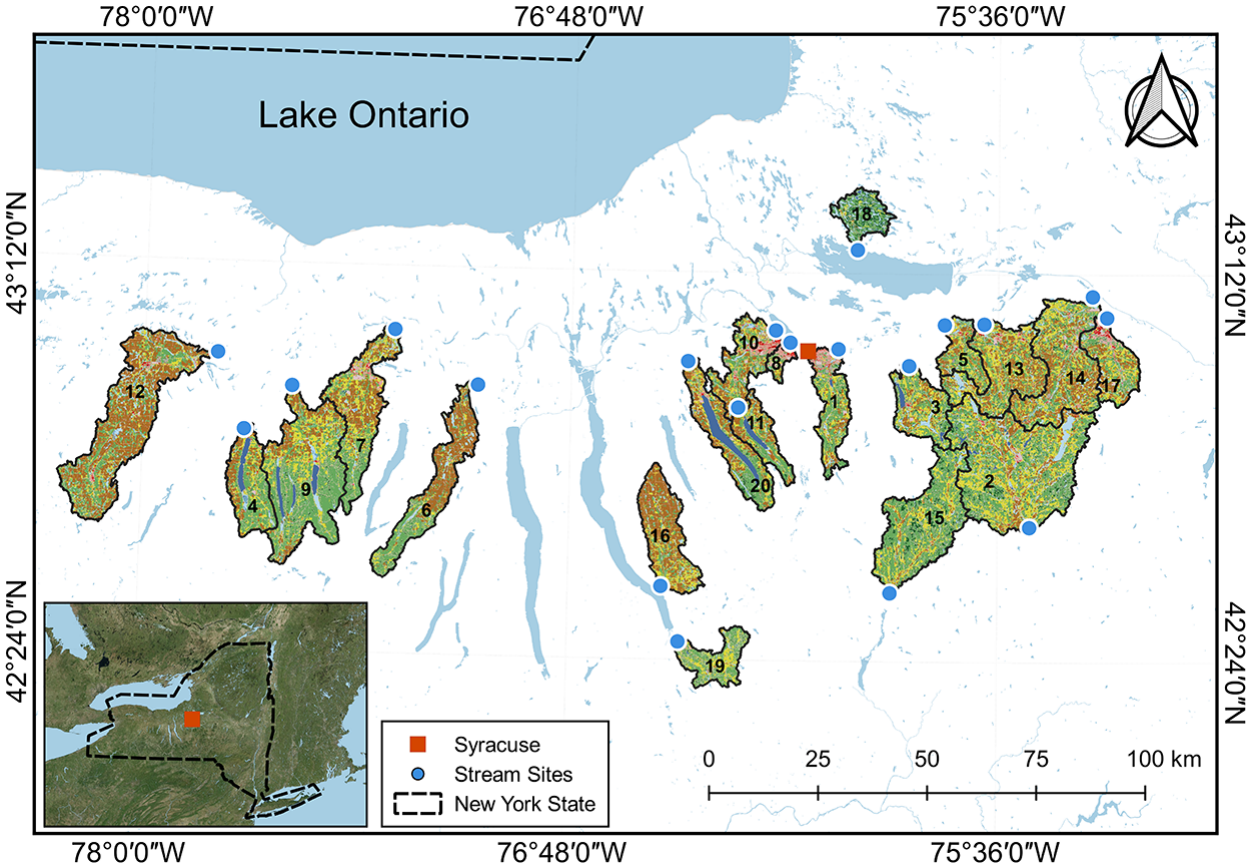
Map of stream sites with watershed ID numbers and names: 1 - Butternut Creek; 2 - Chenango River; 3 - Chittenango Creek; 4 - Conesus Creek; 5 - Cowaselon Creek; 6 - Flint Creek; 7 - Ganargua Creek; 8 - Harbor Brook; 9 - Honeoye Creek; 10 - Ninemile Creek at Lakeland; 11 - Ninemile Creek at Marietta; 12 - Oatka Creek; 13 - Oneida Creek; 14 - Oriskany Creek; 15 - Otselic River; 16 - Salmon Creek; 17 - Sauquoit Creek; 18 - Scriba Creek; 19 - Sixmile Creek; 20 - Skaneateles Creek. Colors depict land use as described in the 2016 National Land Cover Database.^44^ Generally, yellow/brown represent cultivated cropland, red/pink represent developed areas, greens represent forest, and blues represent open water and wetlands. Further details about stream sampling events and watershed characteristics are summarized in Tables S2 and S3, respectively. Satellite image source: Esri, Maxar, GeoEye, Earthstar Geographics, CNES/Airbus DS, USDA, USGS, AeroGRID, IGN, and the GIS User Community.

Twenty stream sites covering a gradient of agricultural and developed watershed land uses in upstate New York (Figure 1) were chosen for investigation using the Geospatial Attributes of Gages for Evaluating Streamflow data set.^37^ Eighteen sites are collocated with operational USGS stream gages with continuous streamflow monitoring, whereas the two remaining sites are collocated with USGS stream gages with historical streamflow data. Two rounds of field sampling were conducted during July–September 2018 and May–July 2019 to capture dry and wet years, respectively (Table S2). Polar organic chemical integrative samplers (POCIS; each containing Oasis HLB sorbent with an average exposed polyethersulfone membrane surface area to sorbent mass ratio of 220 cm^2^/g)^20^ and perforated stainless-steel canisters were purchased from Environmental Sampling Technologies (St. Joseph, MO). Preassembled passive samplers (with triplicate POCIS disks housed in one perforated stainless-steel canister) were either secured to a steel rod hammered into the streambed or anchored to the shore by a stainless-steel wire rope for an average sampling period of 23 ± 2 days at each site. Minimal biofouling and depositions of suspended solids were observed on POCIS membrane surfaces. Grab samples were collected along each stream at the time of passive sampler deployment and retrieval. Two sites (i.e., Ninemile Creek at Marietta and Skaneateles Creek) were also sampled following a stratified exposure design^23^ with multiple passive samplers deployed and grab samples collected over specified sampling periods in 2019. Field blanks (i.e., POCIS disks wrapped in aluminum foil and sampling bottles filled with ultrapure water, opened in the field, and brought back to the laboratory) were prepared for each sampling event to check for unintended contamination during sample collection and processing. Passive samplers and grab samples were transported to Syracuse University within 6 ± 2 h after collection. POCIS disks and grab samples (following the analysis of physicochemical and optical properties) were stored under −20 °C until extraction.

### Sample Analysis

Within 48 h of collection, POCIS disks (triplicate in each canister) were extracted following the published protocol.^38^ Briefly, Oasis HLB sorbent in each POCIS disk was rinsed into preweighed solid-phase extraction (SPE) cartridges using ultrapure water, dried under ultrahigh-purity N_2_, and weighed to measure the mass recovery of sorbent (i.e., 95 ± 8% for all POCIS disks). Each recovered sorbent was spiked with a mixture of isotope-labeled internal standards (Table S5) and eluted using methanol followed by a mixture of ethyl acetate/methanol (50:50 v/v). Finally, the combined solvent extract was evaporated to dryness under N_2_ and reconstituted with methanol/water (10:90 v/v) to a final volume of 1 mL. Grab samples (duplicate; 500 mL each) were spiked with the same mixture of isotope-labeled internal standards, vacuum filtered through 0.7-μm glass fiber filters, and extracted by mixed-mode SPE cartridges packed with 200 mg of Sepra ZT (Phenomenex), 100 mg of Sepra ZT-SAX (Phenomenex), 100 mg of Sepra ZT-SCX (Phenomenex), and 150 mg of ISOLUTE ENV+ (Biotage) sorbents as the top layer and 200 mg of Enviro-Clean graphitized nonporous carbon (United Chemical Technologies) as the bottom layer.^39^ POCIS and grab sample extracts were batched by sampling events and analyzed in randomized sequences with field blanks by a Dionex UltiMate 3000 high-performance liquid chromatograph interfaced with a Thermo Scientific LTQ XL hybrid ion trap–Orbitrap high-resolution mass spectrometer. Further descriptions of grab sample extraction and LC-HRMS instrument settings are provided in section S4.

Suspect screening was performed in *TraceFinder 4.1* (Thermo Scientific) with optimized parameters^40^ by comparing the exact masses and isotopic patterns of peaks (i.e., mass spectral features) in the full scan mass spectra of POCIS and grab sample extracts against those in a custom database (Table S20) containing compound-specific information for 3360 pharmaceuticals, pesticides, personal care products, household chemicals, and industrial additives, as well as their TPs compiled from public listings and the peer-reviewed literature.^40^ Only peaks with a mass accuracy tolerance of 5 ppm and an isotopic pattern fit threshold of >50% were selected for data-dependent tandem mass (dd-MS2) spectra acquisition using higher energy collision-induced dissociation across three collision energies (i.e., 30%, 45%, and 60%). Nontarget screening was performed based on the full scan spectra of POCIS extracts using a node-based workflow developed in *Compound Discoverer 3.3* (Thermo Scientific) by filtering and clustering mass spectral features with peak intensities covarying with watershed characteristics for stream sites. Only peaks fulfilling the following criteria were retained for dd-MS2 spectra acquisition: (a) peak intensity above 10^5^ with reasonable peak width and symmetry; (b) molecular formula predicted from the exact mass exhibiting a spectral similarity score of >0.90 between the theoretical and measured isotope patterns; (c) present in POCIS deployed across all stream sites but not in our suspect compound database; and (d) *z*-score standardized peak intensities in POCIS exhibiting statistically significant correlations with watershed attributes reflecting anthropogenic influences. Multi-energy dd-MS2 spectra of suspect and nontarget compounds were imported into *Compound Discoverer* for interrogation against those available through *mzCloud*(41) and *MassBank*(42) libraries. Parameter settings for *TraceFinder* and *Compound Discoverer* are provided in Tables S7 and S8. Suspect and nontarget compounds with a spectral match factor of >70 were confirmed or rejected by comparing their chromatographic retention times and dd-MS2 spectra to those of authentic reference standards. Out of the 335 compounds prioritized by suspect or nontarget screening (Table S9), 133 were confirmed at level 1 by reference standards (Table S10), whereas the other 202 were rejected and excluded from further quantitative analysis.

Target quantification of OMPs (*n* = 133) in POCIS and grab samples was performed retrospectively by comparing the ratios of their peak areas to those of assigned isotope-labeled internal standards to the corresponding ratios in the calibration standards. Calibration curves were constructed in *TraceFinder* by the non-weighted linear least-squares regression. None of the OMPs quantified in POCIS and grab samples was detectable in the field blanks. Calibration standards of OMPs (processed by SPE as mixtures) and solvent blanks (every 10 samples) were run with each analytical sequence. For each confirmed OMP, the absolute SPE recovery, ion suppression or enhancement, matrix factor, and limits of quantification were determined as described in our previous work.^39^ Complete details of the SPE-LC-HRMS method validation are summarized in Table S10.

### Data Analysis

Watersheds were delineated using sampling sites as the outlets using the USGS *StreamStats* application.^43^ Watershed characteristics (Table S3) for each stream site, such as watershed area, the percent of agricultural or developed land use, the density of septic systems, the number of concentrated animal feeding operations (CAFOs), the capacity of municipal wastewater treatment plants (WWTPs), road density, runoff propensity index (RPI), and population density were extracted from the 2016 National Land Cover Database^44^ and publicly available data sets from state and federal agencies using *QGIS* v.3.1.16^45^ or the *tidycensus*(46) package in *R* 4.0.3.

Following the screening and quantification of OMPs by SPE-LC-HRMS, the daily average mass of OMPs accumulated in POCIS (*m*_POCIS_ in ng/d) and the concentrations of OMPs measured in grab samples (*c*_grab_ in ng/L) were aggregated for statistical analysis using *GraphPad Prism 8.4*. Hierarchical cluster analysis was performed with Spearman’s rank correlation coefficients (ρ) between OMP levels and watershed attributes based on Euclidean distance with Ward’s method using the *ComplexHeatmap*(47) package in *R*. For a subset of 37 OMPs that co-occurred in POCIS and grab samples from a minimum of five stream sites, the field sampling rates (*R*_s_ in L/d) were calculated as the slopes of linear least-squares regression of *m*_POCIS_ against *c*_grab_.^24,26,48^ Field *R*_s_ determined by this approach only represented empirical values without accounting for the cumulative effects associated with fluctuations in site- or event-specific environmental variables. Compound-specific field *R*_s_ of these 37 OMPs were further used to derive time-weighted average concentrations (*c*_TWA_) assuming first-order accumulation kinetics in POCIS.^49^ For each gauged site, the daily average loads of OMPs (*L*_TWA_ and *L*_grab_ in g/d) during each sampling event were estimated using the discharge data (*Q* in m^3^/s) and *c*_TWA_ or *c*_grab_ by the time-weighted concentration algorithm of the *RiverLoad*(50) package in *R*. Lastly, two screening-level risk assessment methods were applied to evaluate the potential for biological effects associated with OMPs. For each site, the cumulative exposure–activity ratios (∑EARs) were calculated for the mixture of OMPs (assuming concentration addition^51,52^) with reliable exposure–response metrics in the ToxCast high-throughput screening database^53^ using the *toxEval* package^54^ in *R*. The multi-substance potentially affected fractions (msPAFs) were calculated by the species sensitivity distributions method (assuming response addition^55^) using the log-transformed acute median toxicity values for multiple species and OMP combinations.^56^ Uncertainties for *L*_TWA_, *L*_grab_, ∑EARs, and msPAFs were assessed by accounting for the variance in field *R*_s_ and propagated errors associated with *c*_TWA_ and *c*_grab_.

## Results and Discussion

### Suspect Screening and Source-Related Clustering of OMPs

Suspect screening prioritized and confirmed 100 OMPs in POCIS (Table S11) and 86 OMPs in grab samples (Table S12), respectively, including 122 unique compounds detected at least once in POCIS and/or grab samples. Of these 122 OMPs, 70 can be broadly classified as pharmaceuticals (e.g., analgesics, antiallergics, antibiotics, antidepressants, antiepileptics, antihypertensives), 23 as pesticides (e.g., fungicides, herbicides, insecticides), 17 as household chemicals (e.g., insect repellents and sunscreen agents) or industrial additives (e.g., vulcanization accelerators and corrosion inhibitors), and 12 as TPs. Most of these OMPs have been detected in U.S. streams draining mixed-use watersheds,^4−7^ but several (e.g., 2-ethyl-2-phenylmalonamide, bamethan, bifenazate, guaifenesin, molindone, prilocaine, rimantadine) have rarely been targeted by previous studies. Sixty-four OMPs were simultaneously detected in POCIS and grab samples with the median *m*_POCIS_ ranging from 0.3 to 91.5 ng/d and median *c*_grab_ ranging from 5 to 750 ng/L, respectively. Thirty-two pharmaceuticals, three insecticides, and *N*-ethyl-*p*-toluenesulfonamide were only detected in POCIS, whereas 13 pharmaceuticals and pharmaceutical TPs, four herbicides, benzophenone, oxybenzone, triclosan, caprolactam, and 1,3-diphenylguanidine were detected in grab samples but not in POCIS. The fact that POCIS and grab samples captured an overlapping but non-identical pool of OMPs confirmed that these two sampling methods complemented each other, with POCIS providing time-integrative enrichment of low-concentration OMPs (assuming no apparent loss of OMPs during POCIS deployment) and grab samples enabling mixed-mode extraction of OMPs beyond those retained by the Oasis HLB sorbent in POCIS. Furthermore, the cumulative daily average mass of OMPs in POCIS (∑*m*_POCIS_; ranging from 23 to 1680 ng/d with a median of 240 ng/d) showed a strong positive correlation (Spearman’s ρ = 0.928; *p* < 0.0001; Figure S3) with the cumulative concentration of OMPs in grab samples (∑*c*_grab_; ranging from 180 to 13 700 ng/L with a median of 2310 ng/L), confirming the relevance of both metrics for inferring the extent of OMP pollution in streams. Fourteen OMPs (i.e., 2,4-D, atrazine, atrazine-2-hydroxy, atrazine-desethyl, benzothiazole, DEET, galaxolidone, lamotrigine, lidocaine, methyl-1H-benzotriazole, metolachlor, metolachlor ethanesulfonic acid, metolachlor oxanilic acid, and sucralose) constituted the most frequently detected compound mixture in POCIS and grab samples and collectively served as a set of indicator compounds for estimating ∑*m*_POCIS_ and ∑*c*_grab_ across stream sites (Figure S4).

**Figure 2:**
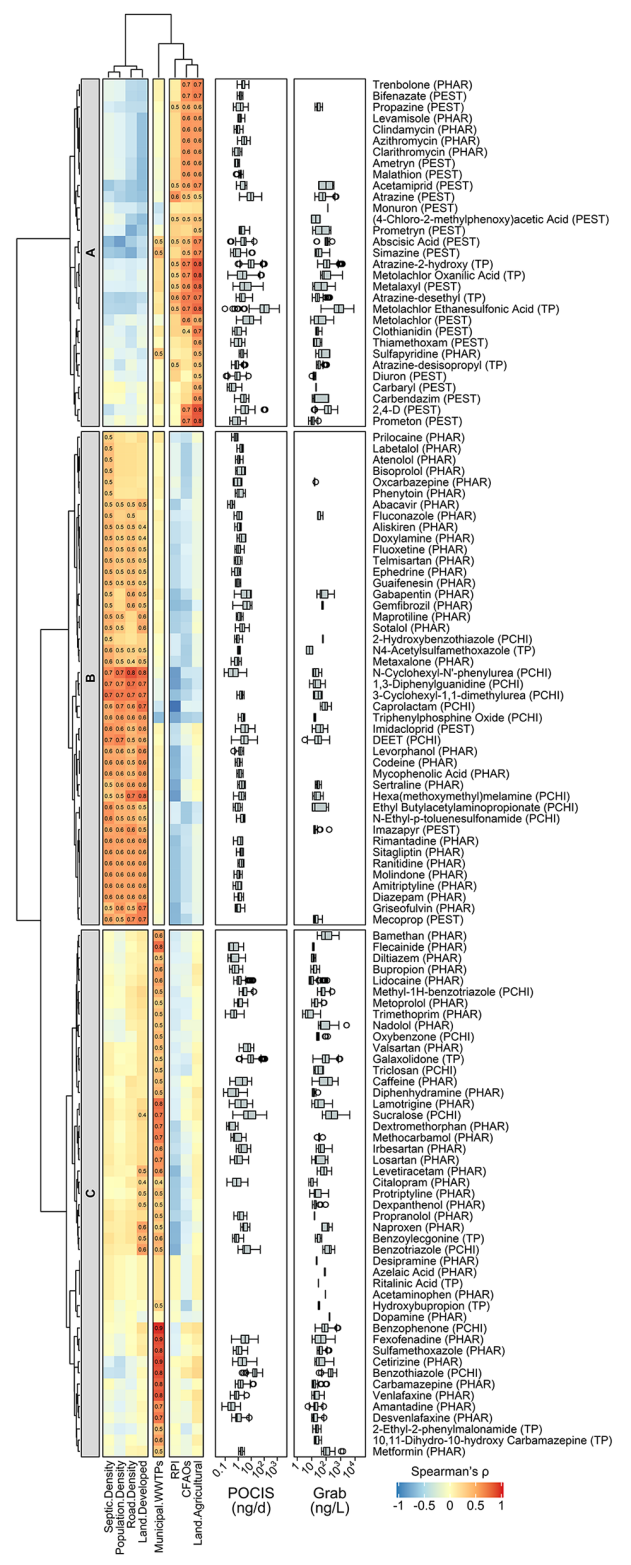
Hierarchical clustering of the Spearman’s rank correlation coefficients (ρ) between *z*-score standardized OMP levels and watershed attributes based on Ward’s method and Euclidean distance. The color scale (red to blue) measures Spearman’s ρ values. Statistically significant Spearman’s ρ values are marked in corresponding heatmap cells. OMPs identified via suspect screening (*n* = 122) are grouped into three clusters (i.e., clusters A, B, and C). Cluster A contains 31 OMPs primarily derived from agricultural landscapes. Cluster B contains 44 OMPs primarily derived from developed landscapes. Cluster C contains 47 OMPs primarily of wastewater origin. The row annotations correspond to the quantifiable daily average mass of OMPs accumulated in POCIS (*m*_POCIS_ in ng/d) and the quantifiable concentrations of OMPs measured in grab samples (*c*_grab_ in ng/L), respectively. “PHAR” represents pharmaceuticals. “PEST” represents pesticides. “PCHI” represents personal care, household, and industrial chemicals, and “TP” represents transformation products, respectively. Each box extends from the 25th to 75th percentiles. The whiskers extend down to the 25th percentile minus 1.5 times the interquartile range and up to the 75th percentile plus 1.5 times the interquartile range. The centerline in each box marks the median. Data points plotted beyond the whiskers are outliers. The column annotations correspond to watershed attributes for stream sites. “CAFOs” represents the number of concentrated animal feeding operations within the watershed. “RPI” represents runoff propensity index. OMP levels and detection frequencies at the 20 stream sites are summarized in Tables S11 and S12, respectively.

To explore the source-related occurrence patterns of OMPs across stream sites, hierarchical cluster analysis was applied to the Spearman’s correlation matrix between the *z*-score standardized *m*_POCIS_ or *c*_grab_ of OMPs and watershed attributes, which partitioned 122 OMPs into three clusters (Figure 2). Cluster A OMPs (*n* = 31) contain 11 herbicides (i.e., 2,4-D, (4-chloro-2-methylphenoxy)acetic acid, metolachlor, two phenylureas, and six *s*-triazines), six insecticides (i.e., bifenazate, carbaryl, malathion, and three neonicotinoids), two fungicides (i.e., carbendazim and metalaxyl), one plant hormone (i.e., abscisic acid), five TPs of atrazine and metolachlor, and six veterinary drugs (i.e., azithromycin, clarithromycin, clindamycin, levamisole, sulfapyridine, and trenbolone). On average, the site-specific detection frequency of cluster A OMPs was 68 ± 33%. Atrazine, metolachlor, and five of their TPs were detected in all streams, among which metolachlor ethanesulfonic acid occurred at the highest median *m*_POCIS_ and *c*_grab_ of 91.5 ng/d and 750 ng/L, respectively. With the exception of monuron (only detected in grab samples from Salmon Creek), the *m*_POCIS_ or *c*_grab_ of cluster A OMPs exhibited positive correlations with the percent agricultural land use (ρ = 0.460–0.824; *p* < 0.0001–0.0412; Table S13) and/or the number of CAFOs within watersheds (ρ = 0.448–0.707; *p* = 0.0005–0.0474), suggesting agricultural activities and/or CFAOs as likely sources of cluster A OMPs (Figure 2). Furthermore, the *m*_POCIS_ or *c*_grab_ of abscisic acid, acetamiprid, atrazine, (4-chloro-2-methylphenoxy)acetic acid, metalaxyl, propazine, and five TPs of atrazine and metolachlor showed positive correlations with RPI (ρ = 0.454–0.641; *p* = 0.0023–0.0442), which corroborates prior work reporting that streams draining watersheds with runoff-prone soils are more vulnerable to contamination by pesticides.^8^ Lastly, the site-specific ∑*m*_POCIS_ and ∑*c*_grab_ of cluster A OMPs showed stronger correlations with the percent agricultural land use and the number of CAFOs (ρ = 0.701–0.797; *p* < 0.0001–0.0006; Figure S5) than with RPI (ρ = 0.496–0.537; *p* = 0.0147–0.0261), further supporting runoffs from agricultural fields and/or CFAOs as the key factor driving the export of cluster A OMPs to streams from their watersheds. Cluster B OMPs (*n* = 44) include 30 pharmaceuticals in diverse therapeutic classes (e.g., fluconazole, gabapentin, gemfibrozil, oxcarbazepine, sertraline), N4-acetylsulfamethoxazole, imidacloprid, two herbicides (i.e., imazapyr and mecoprop), two insect repellents (i.e., DEET and ethyl butylacetylaminopropionate), and eight tire rubber-derived compounds (e.g., hexa(methoxymethyl)melamine). On average, the site-specific detection frequency of cluster B OMPs was 40 ± 27%, which was lower than those of clusters A and C OMPs (Tukey’s multiple comparisons test *p* = 0.0002–0.0010). DEET represented the only cluster B OMP detected in all streams and occurred at a median *m*_POCIS_ and *c*_grab_ of 2.5 ng/d and 27 ng/L, respectively. Contrary to the correlation patterns observed with cluster A OMPs, the *m*_POCIS_ or *c*_grab_ of cluster B OMPs showed positive correlations with septic system density (ρ = 0.465–0.744; *p* = 0.0002–0.0383) but no statistically significant correlations with agriculture-related watershed attributes. With the exception of five pharmaceuticals exclusively captured by POCIS, oxcarbazepine, and 2-hydroxybenzothiazole, the *m*_POCIS_ or *c*_grab_ of cluster B OMPs also exhibited positive correlations with the percent watershed developed land use (ρ = 0.447–0.759; *p* = 0.0001–0.0479), road density (ρ = 0.447–0.840; *p* < 0.0001–0.0483), and/or population density (ρ = 0.468–0.750; *p* = 0.0001–0.0375). Together, the site-specific ∑*m*_POCIS_ and ∑*c*_grab_ of cluster B OMPs showed comparable positive correlations with these four watershed attributes (ρ = 0.638–0.812; *p* < 0.0001–0.0025; Figure S5), indicating the joint contribution of septic effluents and runoffs from developed landscapes (e.g., roads and urban areas) to the loading of cluster B OMPs into streams. Cluster C OMPs (*n* = 47) consist of 34 pharmaceuticals (e.g., citalopram, diphenhydramine, flecainide, methocarbamol, naproxen), five pharmaceutical TPs (e.g., benzoylecgonine), two UV filters (i.e., benzophenone and oxybenzone), sucralose, triclosan, galaxolidone, and three industrial additives (i.e., benzotriazole, methyl-1H-benzotriazole, and benzothiazole). On average, the site-specific detection frequency of cluster C OMPs was 55 ± 29%. Benzothiazole and sucralose were detected in all steams and occurred at the highest median *m*_POCIS_ and *c*_grab_ of 18.6 ng/d and 230 ng/L, respectively, among cluster C OMPs. With the exception of four pharmaceuticals and ritalinic acid (only detected in grab samples from Ganargua Creek), the *m*_POCIS_ or *c*_grab_ of cluster C OMPs exhibited positive correlations with the summed capacity of municipal WWTPs within watersheds (ρ = 0.448–0.935; *p* < 0.0001–0.0477). Likewise, the site-specific ∑*m*_POCIS_ and ∑*c*_grab_ of cluster C OMPs both showed positive correlations with the watershed-specific capacity of municipal WWTPs (ρ = 0.725–0.776; *p* < 0.0001–0.0003; Figure S5), highlighting effluents discharged from municipal WWTPs as a plausible source of cluster C OMPs to streams. Taken together, suspect screening and hierarchical clustering identified and classified 122 OMPs in relation to land cover characteristics and source heterogeneity within mixed-use watersheds, which not only underscored the complex linkage between the occurrence profiles of OMPs in streams and watershed attributes but also served to inform our subsequent nontarget screening efforts.

### Nontarget Screening of OMPs Based on Watershed Attributes

**Figure 3:**
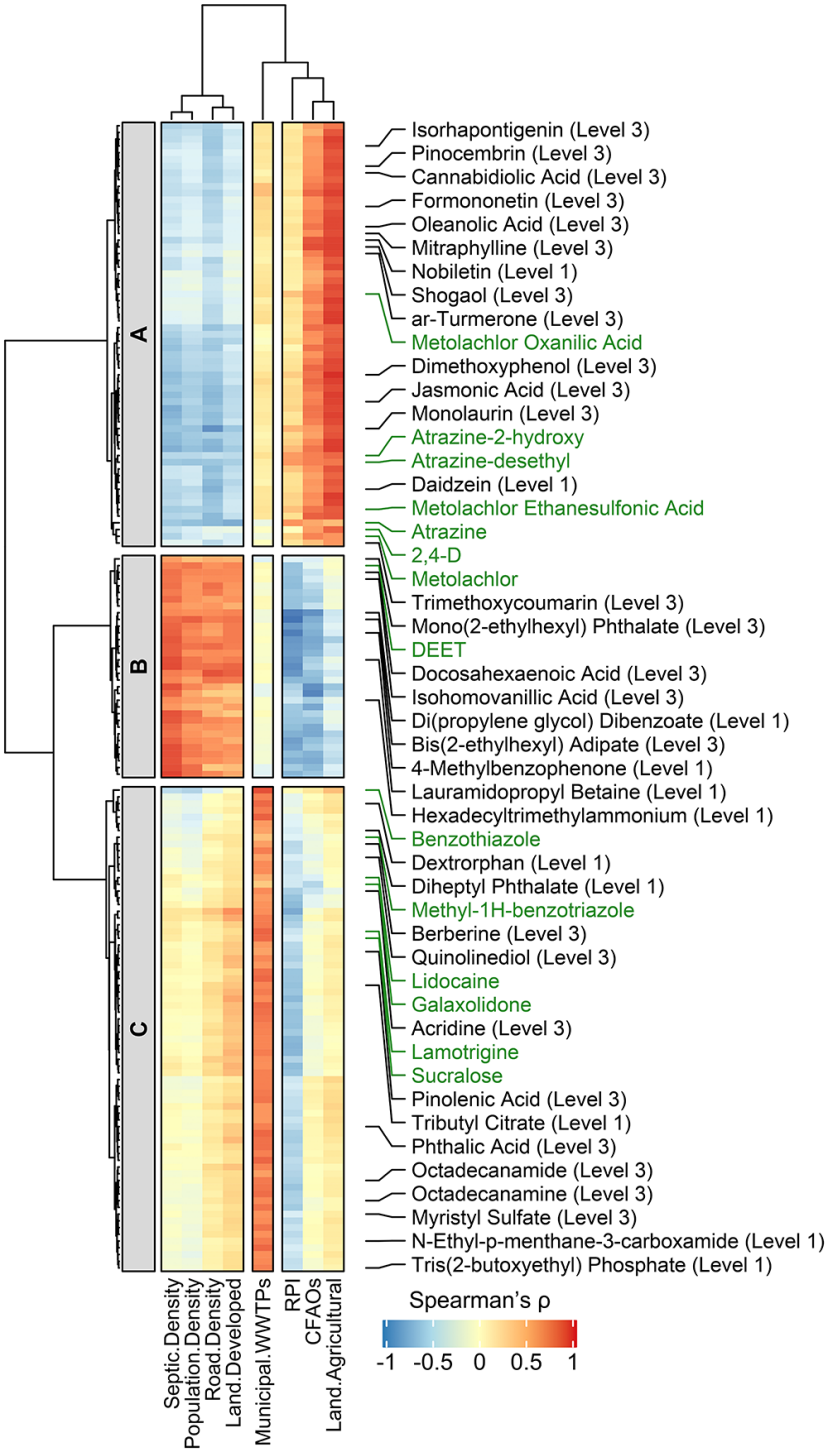
Hierarchical clustering of the Spearman’s rank correlation coefficients (ρ) between *z*-score standardized peak intensities of nontarget features and watershed attributes based on Ward’s method and Euclidean distance. The color scale (red to blue) measures Spearman’s ρ values. Nontarget features (*n* = 154) are grouped into three clusters (i.e., clusters A, B, and C) with 14 most frequently detected OMPs identified via suspect screening (i.e., 2,4-D, atrazine, atrazine-2-hydroxy, atrazine-desethyl, benzothiazole, DEET, galaxolidone, lamotrigine, lidocaine, methyl-1H-benzotriazole, metolachlor, metolachlor ethanesulfonic acid, metolachlor oxanilic acid, and sucralose; compound names highlighted in green). Cluster A contains 56 nontarget features primarily derived from agricultural landscapes. Cluster B contains 32 nontarget features primarily derived from developed landscapes. Cluster C contains 66 nontarget features primarily of wastewater origin. The row annotations correspond to the names of 11 confirmed (at level 1) and 24 tentatively identified (at level 3; top-ranked structures in *mzCloud*) nontarget compounds. The column annotations correspond to watershed attributes for stream sites. “CAFOs” represents the number of concentrated animal feeding operations within the watershed. “RPI” represents runoff propensity index.

Given the time-integrative nature of passive sampling, nontarget screening was performed using POCIS extracts to identify additional compounds not on the suspect list and/or inadequately captured by grab sampling. Hypothetically, the source-related clustering of 122 OMPs can be leveraged to filter and prioritize nontarget features assuming that compounds with similarities in their watershed source dynamics and transport mechanisms would have a high probability to co-occur in the receiving streams. For example, POCIS deployed in streams featuring higher levels of cluster C OMPs might accumulate more nontarget compounds of wastewater origin. In contrast, enrichment of nontarget compounds derived from agricultural and developed landscapes likely occurred to a greater extent in POCIS retrieved from sites featuring elevated levels of clusters A and B OMPs, respectively. To test this hypothesis, mass spectral features with *z*-score standardized peak intensities exhibiting statistically significant positive correlations with one or more watershed attributes were extracted from the POCIS data set, resulting in a total of 154 non-redundant mass spectral features (Table S15) following retention time alignment, peak componentization, and background subtraction. Hierarchical cluster analysis was then applied to the Spearman’s correlation matrix, which grouped 154 nontarget features into three clusters with the 14 most prevalent OMPs identified via suspect screening (Figure 3).

Cluster A nontarget features (*n* = 56) co-occurred with 2,4-D, atrazine, metolachlor, and four TPs of atrazine and metolachlor, pointing to their potential associations with diffuse runoff inputs from agricultural landscapes. Indeed, the peak intensities of cluster A nontarget features showed positive correlations with the percent agricultural land use (ρ = 0.617–0.893; *p* < 0.0001–0.0038; Table S14) and the number of CAFOs (ρ = 0.491–0.811; *p* < 0.0001–0.0278) within watersheds. Two cluster A nontarget features were confirmed at level 1 as daidzein and nobiletin (Figure S6) by reference standards, whereas 12 other nontarget features were also tentatively identified at level 3^57^ as phytochemicals based on the top-ranked structures in *mzCloud* (Table S15). Compared to atrazine and metolachlor, daidzein and nobiletin occurred at a relatively low median *m*_POCIS_ of 0.3 ng/d; however, they have garnered increasing attention as phytotoxins^58^ because of their known estrogenic activity and off-field transport potentials in agricultural-impacted streams.^59,60^ Cluster B nontarget features (*n* = 32) were likely associated with septic system discharge and runoff contribution from adjoining developed areas because their peak intensities showed positive correlations with septic system density (ρ = 0.598–0.841; *p* < 0.0001–0.0053), the percent developed land use (ρ = 0.448–0.761; *p* < 0.0001–0.0475), road density (ρ = 0.451–0.797; *p* < 0.0001–0.0459), and population density (ρ = 0.492–0.764; *p* < 0.0001–0.0277) within watersheds. Four cluster B nontarget features were confirmed at level 1, including hexadecyltrimethylammonium, lauramidopropyl betaine, di(propylene glycol) dibenzoate, and 4-methylbenzophenone (Figure S6), which occurred at a median *m*_POCIS_ of 0.2 to 0.4 ng/d. Hexadecyltrimethylammonium and lauramidopropyl betaine are quaternary ammonium compounds commonly used in cosmetics and personal hygiene products or as industrial additives. Hexadecyltrimethylammonium has been detected in multiple environmental media, ranging from river waters^61^ and wastewater effluents^62^ to sewage sludge^63^ and urban estuarine sediments.^64^ Likewise, lauramidopropyl betaine has been detected in various environmental samples such as aqueous film-forming foams,^65^ hydraulic fracturing flowback and produced waters,^66^ and wastewater-impacted river waters.^67^ Di(propylene glycol) dibenzoate is a high production volume chemical previously identified in household dust^68^ and tire rubber leachates^69^ and may undergo biotransformation to form more toxic monobenzoate isomers.^70,71^ 4-Methylbenzophenone is one of the most frequently studied photoinitiators due to concerns over its tendency to leach from food packaging,^72^ but its environmental occurrence data remain scarce. Cluster C nontarget features (*n* = 66) co-occurred with sucralose as well as five other frequently detected wastewater-derived OMPs, and their peak intensities showed positive correlations with the summed capacity of municipal WWTPs within watersheds (ρ = 0.575–0.761; *p* < 0.0001–0.0079). Five cluster C nontarget features were confirmed at level 1, including dextrorphan, *N*-ethyl-*p*-menthane-3-carboxamide, diheptyl phthalate, tributyl citrate, and tris(2-butoxyethyl) phosphate (TBEP). TBEP occurred at a slightly higher median *m*_POCIS_ of 8.8 ng/d than that of sucralose, whereas the other four compounds occurred at a low median *m*_POCIS_ of 0.2 to 0.8 ng/d. Dextrorphan is a TP of dextromethorphan (an antitussive with a site-specific detection frequency of 50%) and has been detected in wastewater effluents and effluent-receiving river waters.^73,74^*N*-Ethyl-*p*-menthane-3-carboxamide is a cooling agent added in consumer and industrial products, but its environmental occurrence has not been documented despite its known antiandrogenic properties.^75^ Diheptyl phthalate and tributyl citrate are two high production volume plastic additives that were first identified via suspect and nontarget screening of fine particulate matter^76^ and stormwater runoffs,^77^ respectively. TBEP is a plasticizer and flame retardant that may possess the potential for endocrine disrupting effects^78^ and has been found to accumulate in POCIS deployed in the Great Lakes tributaries.^3^ With the exception of TBEP, none of the level 1 nontarget compounds was captured by grab samples despite their widespread detection in streams. Overall, nontarget screening identified additional compounds of potential relevance for exposure and effect assessments and further supported the notion that OMPs enter streams from mixed sources linked to agricultural production, urban development, and point and nonpoint wastewater discharge within watersheds.

### OMP Load Estimation and Screening-Level Risk Assessments

**Figure 4:**
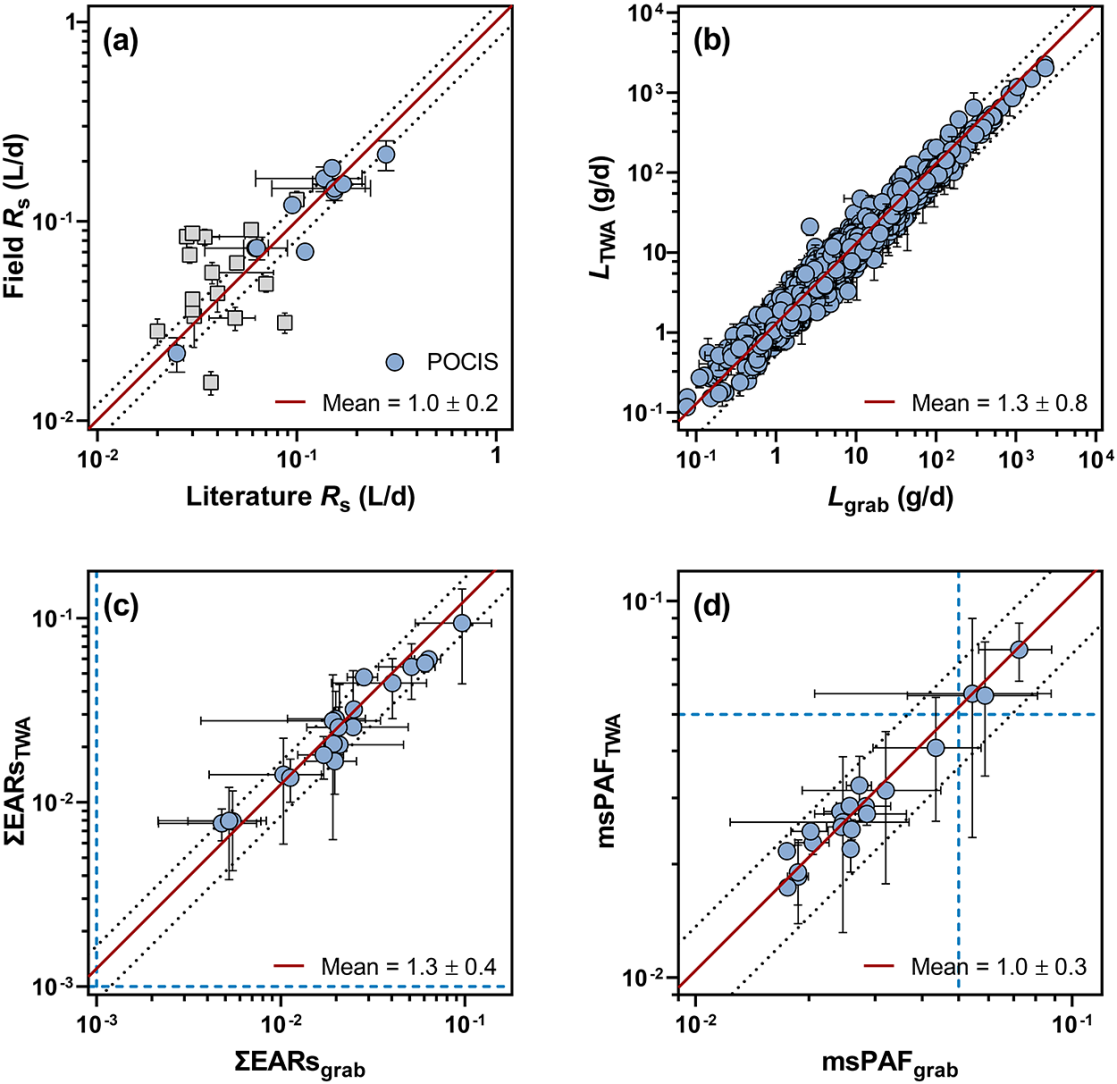
Comparison of POCIS and grab samples for OMP load estimation and screening-level risk assessments: (a) Cross plot of field sampling rates (*R*_s_ in L/d) for OMPs determined herein and those reported in previous field-based passive sampling studies. Field *R*_s_ for 37 OMPs from this work are summarized in Table S16. Literature *R*_s_ for OMPs are listed in Table S17. Compound-wise comparisons of field *R*_s_ were only feasible for 28 of the 37 OMPs, among which blue circles (*n* = 11) and gray squares (*n* = 17) represent POCIS- and non-POCIS-based comparisons, respectively. Further details regarding the comparative analysis are provided in section S7. Error bars represent the standard deviations of field *R*_s_ determined in this work and *R*_s_ reported in the literature. The red solid line represents the mean POCIS-based field *R*_s_/literature *R*_s_ ratio (*n* = 11). The black dotted lines bracket the standard deviation of POCIS-based field *R*_s_/literature *R*_s_ ratios. (b) Cross plot of OMP loads (*L* in g/d) estimated by POCIS (*L*_TWA_) and grab samples (*L*_grab_). Error bars represent the standard deviations of *L*_TWA_ and *L*_grab_. The red solid line represents the mean *L*_TWA_/*L*_grab_ ratio (*n* = 796). The black dotted lines bracket the standard deviation of *L*_TWA_/*L*_grab_ ratios. (c) Cross plot of the site-specific cumulative exposure–activity ratios (∑EARs) calculated based on *c*_TWA_ (∑EARs_TWA_) and *c*_grab_ (∑EARs_grab_). Error bars represent the standard deviations of ∑EARs_TWA_ and ∑EARs_grab_. The red solid line represents the mean ∑EARs_TWA_/∑EARs_grab_ ratio (*n* = 20). The two black dotted lines bracket the standard deviation of ∑EARs_TWA_/∑EARs_grab_ ratios. The two blue dashed lines mark the conservative effects-screening threshold of 0.001. (d) Cross plot of the site-specific multi-substance potentially affected fractions (msPAFs) calculated based on *c*_TWA_ (msPAF_TWA_) and *c*_grab_ (msPAF_grab_). Error bars represent the standard deviations of msPAF_TWA_ and msPAF_grab_. The red solid line represents the mean msPAF_TWA_/msPAF_grab_ ratio (*n* = 20). The two black dotted lines bracket the standard deviation of msPAF_TWA_/msPAF_grab_ ratios. The two blue dashed lines mark the generally accepted effect threshold of 5%.

Concurrent passive and grab sampling at 20 stream sites over two multi-month sampling seasons generated a unique data set for deriving field *R*_s_ for OMPs present at quantifiable levels in both POCIS and grab samples. TBEP and 36 OMPs identified via suspect screening exhibited a coefficient of determination of >0.90 for the linear least-squares regression of *m*_POCIS_ against *c*_grab_ across stream sites (Figure S7). Compound-specific field *R*_s_ for these 37 OMPs ranged from 0.016 ± 0.002 L/d for caffeine to 0.216 ± 0.037 L/d for imidacloprid with a median of 0.070 ± 0.006 L/d for metoprolol (Table S16), which agreed with values reported by previous field-based studies (Figure 4a).

To compare OMP load estimation by POCIS and grab sampling, the *L*_TWA_ of 37 OMPs with field *R*_s_ were evaluated against *L*_grab_ on a compound-, site-, or season-specific basis. Over the POCIS deployment periods, the mean compound-specific *L*_TWA_/*L*_grab_ ratios (Figure S10) varied from 0.9 ± 0.1 for atrazine-desethyl to 2.2 ± 1.1 for bupropion with a median of 1.3 ± 0.7 for lidocaine. Furthermore, the mean *L*_TWA_/*L*_grab_ ratios across stream sites (Figure S11) ranged from 1.2 ± 0.5 for Ganargua Creek to 1.5 ± 1.0 for Skaneateles Creek with a median of 1.3 ± 0.7 for Flint Creek. Lastly, the mean *L*_TWA_/*L*_grab_ ratios over the 2018 and 2019 sampling seasons (Figure S12) was 1.3 ± 0.8 to 1.2 ± 0.7, respectively. Consolidating the *L*_TWA_/*L*_grab_ ratios measured for 37 OMPs across stream sites over two sampling seasons yielded a mean *L*_TWA_/*L*_grab_ ratio of 1.3 ± 0.8 (Figure 4b), which supports the comparability of POCIS and grab samples for OMP load estimation with acknowledged uncertainties in field *R*_s_ estimation. To further examine the impacts of event-scale sampling on *L*_TWA_, the relative changes in OMP loads calculated for stratified and non-stratified sampling were investigated at two sites located in adjacent watersheds (i.e., Ninemile Creek at Marietta and Skaneateles Creek). Each site was sampled multiple times by grab sampling during the same precipitation event with concurrent deployment and retrieval of POCIS covering corresponding time segments (Figure S13). On average, the *L*_TWA_non-stratified_/*L*_grab_stratified_ ratios of OMPs at these two sites were 1.2 ± 0.7 and 1.3 ± 0.7 (Figure S13), respectively, demonstrating the comparable performance of non-stratified passive sampling and stratified grab sampling at both sites during our sampling periods. Similarly, the *L*_TWA_stratified_/*L*_grab_stratified_ ratios of OMPs at these two sites were 1.8 ± 1.0 and 1.6 ± 0.8, respectively (Figure S13), suggesting that stratified passive sampling did not introduce statistically significant deviations in load estimation from stratified grab sampling either. Ideally, the rising and falling limbs of a hydrograph should be further stratified for sampling due to fluctuations in POCIS sampling rates and OMP fluxes during different hydrological regimes.^23,79^ However, optimizing a sampling program is challenging given the dynamic nature of watershed hydrology and stream responses. Moreover, the nonsystematic covariance between concentration (i.e., chemographs) and discharge (i.e., hydrographs) complicated load calculations,^23^ so long-term monitoring of OMPs at representative stream sites is required for improved characterization of the covariance to correct for potential bias in load estimation.

To screen for possible ecologically relevant effects of OMPs on aquatic life, the EAR and msPAF metrics were applied to evaluate the potential for *in vitro* vertebrate-centric sublethal effects^52^ and *in vivo* lethal effects toward aquatic species assemblages^56^ associated with OMPs, respectively. Of the 133 OMPs quantified via suspect and nontarget screening, 118 were represented in the ToxCast database (including their free and salt forms; Table S18),^53^ while 111 had species sensitivity distribution data (Table S19).^56^ On average, the site-specific ∑EARs_TWA_ and ∑EARs_grab_ varied from 0.006 ± 0.004 to 0.095 ± 0.066 with a mean ∑EARs_TWA_/∑EARs_grab_ ratio of 1.3 ± 0.4 (Figure 4c). Together, clusters A and C OMPs contributed to 98 ± 4% of ∑EARs_TWA_ and ∑EARs_grab_ (Figure S14), but only 2,4-D, atrazine, metolachlor, benzothiazole, and TBEP exhibited a median EAR at or above the precautionary effects-screening threshold of 0.001 under mean exposure conditions.^78^ Cluster A OMPs typically dominated ∑EARs_TWA_ and ∑EARs_grab_ over cluster C OMPs, as reflected by the positive correlations between ∑EARs_TWA_ or ∑EARs_grab_ and the percent agricultural land use (ρ = 0.707–0.747; *p* = 0.0002–0.0005; Figure S15) or the number of CAFOs within watersheds (ρ = 0.552–0.604; *p* = 0.0048–0.0117). Most stream sites exhibited elevated EARs in the nuclear receptor, DNA binding, and/or oxidoreductase assay groupings, which was consistent with results from the bioactivity profiling of surface water samples from watersheds in the Great Lakes Basin.^52^ However, linking EAR-prioritized assay responses to the potential for adverse outcomes warrants further investigation. On the other hand, the site-specific msPAF_TWA_ and msPAF_grab_ varied from 1.7 ± 0.1% to 7.3 ± 2.1% with a mean msPAF_TWA_/msPAF_grab_ ratio of 1.0 ± 0.3 (Figure 4d). Only msPAF_TWA_ for three stream sites exceeded the generally accepted effect threshold of 5%,^80^ suggesting that >5% of the sensitive aquatic species could potentially be affected by the mixture of OMPs under environmentally relevant exposure scenarios. Clusters A and B OMPs jointly explained 99 ± 1% of msPAF_TWA_ and msPAF_grab_ (Figure S16), with atrazine-desethyl and imidacloprid serving as two top contributing compounds. msPAF_TWA_ and msPAF_grab_ only showed positive correlations with the percent agricultural land use (ρ = 0.582–0.768; *p* < 0.0001–0.0071; Figure S17) and the number of CAFOs within watersheds (ρ = 0.592–0.650; *p* = 0.0019–0.0049), further underlining cluster A OMPs as drivers for the potential for biological effects. Such screening-level calculations likely underestimated the mixture effects of OMPs as they only accounted for OMPs amenable to our sampling and analytical techniques and those with available bioactivity data. Still, the EAR and msPAF frameworks provided initial insights into the potential for biological effects associated with the mixture of OMPs, which may guide further ecotoxicological studies at sites of concern. Overall, these results illustrate the feasibility of coupling POCIS and grab sampling for OMP load estimation and screening-level assessments of biological effects associated with OMPs.

### Environmental Implications

This work demonstrates the prospect of combining passive sampling with high-resolution accurate mass screening for the multi-watershed characterization of OMP contamination status in streams. Suspect and nontarget screening based on LC-HRMS confirmed 133 unique OMPs in POCIS and grab samples, although future work may consider incorporating mixed-mode passive sampling and HRMS configurations with complementary chromatography and ionization techniques to expand analytical coverage. Our hierarchical cluster analysis highlighted commonalities and differences in OMP occurrence profiles in relation to watershed attributes indicative of anthropogenic influences. Furthermore, our work demonstrated a nontarget screening approach through the cross-site comparison of POCIS-amenable mass spectral features to prioritize and identify OMPs beyond those captured by grab sampling and suspect screening. This approach may be extended to improve OMP characterization in streams targeted by multi-region assessment studies^5,6^ or adapted for the retrospective analysis of nontarget OMPs in POCIS extracts from sites of concern assuming the long-term storage stability of OMPs archived on POCIS.^81^ Our results also supported the versatility of POCIS for estimating loads of OMPs and assessing their potential for biological effects, although high-frequency field measurements^82^ represent the best means to resolve the fine-scale variability in OMP profiles if operational feasibility is not a constraint for the spatiotemporal extent of studies. Overall, the sampling and screening framework developed in this work is anticipated to be transferable to future OMP studies with a similar or broader scope. However, more focused efforts on the fate and transport modeling of OMPs under dynamic hydrological and biogeochemical conditions are still required to reconcile OMP signatures and hydrological responses in streams, generalize mechanistic drivers underlying OMP occurrence dynamics, and inform the development of OMP mitigation measures across landscapes.

## Supporting Information Available

Stream site coordinates, sampling dates, and watershed attributes; summary of physicochemical and optical properties of streamwater samples; LC-HRMS instrument parameters and screening workflow settings; SPE-LC-HRMS method performance for OMP analysis; summary statistics of OMP occurrence in POCIS and grab samples; list of compounds identified via nontarget screening; field sampling rates for 37 OMPs; comparison of load estimation for 37 OMPs by POCIS and grab samples; exposure–activity ratio analysis; multi-substance potentially affected fraction analysis; compound database for suspect screening (PDF)
