# Supplementary material for: Combining Passive Sampling with Suspect and Nontarget Screening to Characterize Organic Micropollutants in Streams Draining Mixed-Use Watersheds
Source: Environ Sci Technol. 2022 Nov 4;56(23):16726–36. doi: 10.1021/acs.est.2c02938 (PMC9730844; doi:10.1021/acs.est.2c02938)
Supplement: Supplementary file 1 — es2c02938_si_001.pdf [file es2c02938_si_001.pdf]

Supporting Information for

Combining Passive Sampling with Suspect and Nontarget Screening to Characterize Organic Micropollutants in Mixed-Use Watersheds

Shiru Wang[1], Ruta Basijokaite[2], Bethany L. Murphy[1], Christa A. Kelleher[2], Teng Zeng[1]

[1]Department of Civil and Environmental Engineering, Syracuse University, 151 Link Hall, Syracuse, New York 13244, United States

[2]Department of Earth and Environmental Sciences, Syracuse University, 204 Heroy Geology Laboratory, Syracuse, New York 13244, United States

(Total 149 pages, 5 texts, 20 tables, 17 figures)

Table of Contents

S1. Chemical sources and sampling supplies S3

S2. Stream sampling events and watershed attributes S13

S3. Analysis of physicochemical and optical properties of stream water samples S15

S4. Screening and quantification of OMPs in POCIS and grab samples by SPE-LC-HRMS S18

S5. Watershed attributes as predictors for OMP levels in streams S44

S6. Watershed attributes as factors for prioritization of nontarget compounds in POCIS S49

S7. Field sampling rates for OMPs S60

S8. Comparison of load estimation for OMPs by POCIS and grab samples S75

S9. Exposure-activity ratio (EAR) analysis S83

S10. Multi-substance potentially affected fraction (msPAF) analysis S89

S11. Compound database for SPE-LC-HRMS suspect screening S95

References S146

S1. Chemical sources and sampling supplies

Chemicals and reagents were used as received without further purification unless otherwise noted. Methanol (MeOH; HPLC and LC-MS grade), acetonitrile (HPLC and LC-MS grade); water (H_2O; HPLC and LC-MS grade), ethyl acetate (LC-MS grade), formic acid solution (FA; ≥99.0%; LC-MS grade), ammonium hydroxide solution (≥25%; LC-MS grade), and ammonium acetate (LC-MS grade) were supplied by Fisher Scientific. Five solid-phase extraction (SPE) sorbents, including Sepra ZT (30 µm polymer, 85 Å), Sepra ZT-SAX (30 µm polymer, 85 Å), Sepra ZT-SCX (30 µm polymer, 85 Å), ISOLUTE ENV+ (90 µm hydroxylated polystyrene-divinyl benzene copolymer, 800 Å), and Enviro-Clean graphitized carbon (non-porous 120/400 mesh), were purchased from Phenomenex, Biotage, and United Chemical Technologies. Other SPE supplies were purchased from United Chemical Technologies. POCIS and perforated stainless-steel canisters were purchased from Environmental Sampling Technologies (St. Joseph, MO). POCIS consist of washers (5.4 cm inner diameter and 10 cm outer diameter), polyethersulfone membranes (140 µm thickness and 0.1 mm pore diameter), and Oasis HLB sorbent (220 mg).[1] Field sampling supplies (e.g., wheeled coolers, sampling bottles, steel rods, and wire rope kits) were purchased from Fisher Scientific or local hardware stores. Unlabeled reference standards and isotope-labeled internal standards were purchased from Sigma-Aldrich, Thermo Scientific Chemicals, Toronto Research Chemicals, AccuStandard, Cayman Chemical Company, Alfa Aesar, Santa Cruz Biotechnology, TCI America, C/D/N Isotopes, and Cambridge Isotope Laboratories as high-purity substances or concentrated solutions (**Table S1**). Stock solutions were prepared by dissolving or diluting a gravimetrically weighted amount of solid or liquid reference standards into LC-MS grade methanol, acetonitrile, or pH-adjusted water. Spike solutions and calibration standards were prepared by diluting predetermined volumes of stock solutions into LC-MS grade water. Mobile phases for LC-HRMS analysis were prepared using LC-MS grade water, methanol, and formic acid. Sampling bottles, POCIS, and stainless-steel canisters were rinsed with HPLC grade methanol followed by ultrapure water and wrapped in aluminum foil prior to field sampling. Non-volumetric glassware was rinsed with ultrapure water and combusted at 450 °C in a Thermo Scientific Lindberg/Blue M Moldatherm box furnace for a minimum of 5 h.

Table S1. List of unlabeled reference standards and isotope-labeled internal standards

Compound Name	CAS	Supplier	Catalog Number	Category
17β-Estradiol	50-28-2	Toronto Research Chemicals	E888000	PHAR
3,4-Methylenedioxymethamphetamine (MDMA)	42542-10-9	Sigma-Aldrich	M-013-1ML	PHAR
3,4-Methylenedioxy-N-ethylamphetamine (MDEA)	82801-81-8	Sigma-Aldrich	M-065-1ML	PHAR
Abacavir	136470-78-5	Toronto Research Chemicals	A104990	PHAR
Acamprosate	77337-76-9	Toronto Research Chemicals	A120000	PHAR
Acebutolol	37517-30-9	Sigma-Aldrich	A3669-1G	PHAR
Acetaminophen	103-90-2	Sigma-Aldrich	A3035-1VL	PHAR
Albendazole	54965-21-8	AccuStandard	P-498S	PHAR
Albuterol	18559-94-9	Toronto Research Chemicals	A514501	PHAR
Aliskiren	173334-57-1	Sigma-Aldrich	SML2077-50MG	PHAR
Allopurinol	315-30-0	Sigma-Aldrich	A8003-5G	PHAR
Amantadine	768-94-5	Sigma-Aldrich	A1260-5G	PHAR
Amitriptyline	50-48-6	Sigma-Aldrich	A-923-1ML	PHAR
Amobarbital	57-43-2	Sigma-Aldrich	A-020-1ML	PHAR
Amphetamine	300-62-9	Sigma-Aldrich	A-007-1ML	PHAR
Androstanolone (5α-Dihydrotestosterone; DHT)	521-18-6	Sigma-Aldrich	D-073-1ML	PHAR
Androstenedione	63-05-8	Sigma-Aldrich	A-075-1ML	PHAR
Atenolol	29122-68-7	Toronto Research Chemicals	A790075	PHAR
Atomoxetine	83015-26-3	Sigma-Aldrich	PHR1679-500MG	PHAR
Atropine	51-55-8	Toronto Research Chemicals	A794630	PHAR
Azelaic Acid	123-99-9	Sigma-Aldrich	95054-100MG	PHAR
Azithromycin	83905-01-5	Sigma-Aldrich	PHR1088-1G	PHAR
Bamethan	3703-79-5	Sigma-Aldrich	B0382-5G	PHAR
Betamethasone	378-44-9	Sigma-Aldrich	PHR1398-1G	PHAR
Betaxolol	63659-18-7	Sigma-Aldrich	B5683-10MG	PHAR
Bisoprolol	66722-44-9	Toronto Research Chemicals	B510500	PHAR
Bupivacaine	38396-39-3	Sigma-Aldrich	PHR1128-1G	PHAR
Buprenorphine	52485-79-7	Sigma-Aldrich	B-044-1ML	PHAR
Bupropion	34911-55-2	Toronto Research Chemicals	B689625	PHAR
Butalbital	77-26-9	Sigma-Aldrich	B-006-1ML	PHAR
Butylone	802575-11-7	Sigma-Aldrich	B-045-1ML	PHAR
Caffeine	58-08-2	Sigma-Aldrich	C0750-5G	PHAR
Cannabinol	521-35-7	AccuStandard	CP-CBN-01S	PHAR
Capsaicin	404-86-4	Toronto Research Chemicals	C175685	PHAR
Carbamazepine	298-46-4	Sigma-Aldrich	C4024-1G	PHAR
Celiprolol	56980-93-9	Sigma-Aldrich	SML2617-5MG	PHAR
Cetirizine	83881-51-0	Toronto Research Chemicals	C281100	PHAR
Cimetidine	51481-61-9	Sigma-Aldrich	C4522-5G	PHAR
Citalopram	59729-33-8	Toronto Research Chemicals	C505000	PHAR
Clarithromycin	81103-11-9	Sigma-Aldrich	PHR1038-500MG	PHAR
Clenbuterol	37148-27-9	Sigma-Aldrich	C5423-10MG	PHAR
Clindamycin	18323-44-9	Toronto Research Chemicals	C580000	PHAR
Codeine	76-57-3	Sigma-Aldrich	C-006-1ML	PHAR
Crotamiton	124236-29-9	Toronto Research Chemicals	C818000	PHAR
Cortisone	53-06-5	Sigma-Aldrich	C-130-1ML	PHAR
Cycloheximide	66-81-9	AccuStandard	P-411S	PHAR
Cyclopentolate	512-15-2	Toronto Research Chemicals	C988430	PHAR
Desipramine	50-47-5	Sigma-Aldrich	PHR1723-400MG	PHAR
Desomorphine	427-00-9	Sigma-Aldrich	D-083-1ML	PHAR

**Table S1.** List of unlabeled reference standards and isotope-labeled internal standards (continued)				
Compound Name	CAS	Supplier	Catalog Number	Category
Desvenlafaxine (O-Desmethyl Venlafaxine)	93413-62-8	Sigma-Aldrich	V-007-1ML	PHAR
Detomidine	76631-46-4	Toronto Research Chemicals	D297975	PHAR
Dexpanthenol	81-13-0	Sigma-Aldrich	PHR1228-500MG	PHAR
Dextromethorphan	125-71-3	Sigma-Aldrich	D-013-1ML	PHAR
Diazepam	439-14-5	Sigma-Aldrich	D-907-1ML	PHAR
Diclofenac	15307-86-5	Sigma-Aldrich	93484-100MG	PHAR
Dienogest	65928-58-7	Sigma-Aldrich	SML1468-10MG	PHAR
Dihydrocodeine	125-28-0	Sigma-Aldrich	D-019-1ML	PHAR
Dihydromorphine	509-60-4	Sigma-Aldrich	D-033-1ML	PHAR
Diltiazem	42399-41-7	Sigma-Aldrich	D-035-1ML	PHAR
Dinoprostone (Prostaglandin E2)	363-24-6	Toronto Research Chemicals	P838610	PHAR
Diphenhydramine	58-73-1	Sigma-Aldrich	D-015-1ML	PHAR
Dobutamine	34368-04-2	Sigma-Aldrich	D0676-10MG	PHAR
Dopamine	51-61-6	Sigma-Aldrich	H8502-5G	PHAR
Doxylamine	469-21-6	Sigma-Aldrich	D3775-5G	PHAR
Enalapril	75847-73-3	Sigma-Aldrich	PHR1289-1G	PHAR
Enalaprilat	76420-72-9	Toronto Research Chemicals	E555375	PHAR
Ephedrine	299-42-3	Sigma-Aldrich	E-011-1ML	PHAR
Epinephrine	51-43-4	Sigma-Aldrich	E4250-1G	PHAR
Estriol	50-27-1	Sigma-Aldrich	E1253-100MG	PHAR
Estrone	53-16-7	Toronto Research Chemicals	E889050	PHAR
Ethosuximide	77-67-8	Sigma-Aldrich	PHR1413-1G	PHAR
Fexofenadine	83799-24-0	Toronto Research Chemicals	F322470	PHAR
Fingolimod	162359-55-9	Sigma-Aldrich	SML0700-5MG	PHAR
Flecainide	54143-55-4	Sigma-Aldrich	F-017-1ML	PHAR
Fluconazole	86386-73-4	Sigma-Aldrich	PHR1160-1G	PHAR
Fluoxetine	54910-89-3	Sigma-Aldrich	F-918-1ML	PHAR
Flurandrenolide	1524-88-5	Sigma-Aldrich	1284000-100MG	PHAR
Furosemide	54-31-9	Sigma-Aldrich	F4381-1G	PHAR
Gabapentin	60142-96-3	Sigma-Aldrich	PHR1049-1G	PHAR
Gemfibrozil	25812-30-0	Sigma-Aldrich	PHR1286-1G	PHAR
Glutethimide	77-21-4	Sigma-Aldrich	G-005-1ML	PHAR
Griseofulvin	126-07-8	Sigma-Aldrich	G4753-5G	PHAR
Guaifenesin	93-14-1	Toronto Research Chemicals	G810500	PHAR
Hydrocodone	125-29-1	Sigma-Aldrich	H-003-1ML	PHAR
Hydrocortisone	50-23-7	Sigma-Aldrich	H0888-1G	PHAR
Hydromorphone	466-99-9	Sigma-Aldrich	H-004-1ML	PHAR
Ibuprofen	15687-27-1	Sigma-Aldrich	I4883-1G	PHAR
Imidocarb	27885-92-3	Toronto Research Chemicals	I387628	PHAR
Irbesartan	138402-11-6	Sigma-Aldrich	PHR1443-1G	PHAR
Ketamine	6740-88-1	Sigma-Aldrich	K-002-1ML	PHAR
Labetalol	36894-69-6	Sigma-Aldrich	PHR1335-1G	PHAR
Lamotrigine	84057-84-1	Toronto Research Chemicals	L173250	PHAR
Levamisole	14769-73-4	Toronto Research Chemicals	L331100	PHAR
Levetiracetam	102767-28-2	Toronto Research Chemicals	L331500	PHAR
Levorphanol	77-07-6	Sigma-Aldrich	L-044-1ML	PHAR
Lidocaine	137-58-6	Toronto Research Chemicals	L397800	PHAR
Linezolid	165800-03-3	Toronto Research Chemicals	L466500	PHAR
Losartan	114798-26-4	Sigma-Aldrich	PHR1602-1G	PHAR

Table S1. List of unlabeled reference standards and isotope-labeled internal standards (continued)

Compound Name	CAS	Supplier	Catalog Number	Category
Lovastatin	75330-75-5	Toronto Research Chemicals	L472225	PHAR
Maprotiline	10262-69-8	Sigma-Aldrich	M9651-1G	PHAR
Medroxyprogesterone	520-85-4	Sigma-Aldrich	M6013-250MG	PHAR
Mefenamic Acid	61-68-7	Sigma-Aldrich	92574-250MG	PHAR
Melatonin	73-31-4	Sigma-Aldrich	M5250-250MG	PHAR
Memantine	19982-08-2	Sigma-Aldrich	M9292-25MG	PHAR
Meperidine	57-42-1	Sigma-Aldrich	M-035-1ML	PHAR
Metaraminol	54-49-9	Toronto Research Chemicals	M225565	PHAR
Metaxalone	1665-48-1	Sigma-Aldrich	M-074-1ML	PHAR
Metformin	657-24-9	Sigma-Aldrich	PHR1084-500MG	PHAR
Methamphetamine	537-46-2	Sigma-Aldrich	M-009-1ML	PHAR
Methcathinone	5650-44-2	Sigma-Aldrich	M-061-1ML	PHAR
Methocarbamol	532-03-6	Sigma-Aldrich	PHR1395-1G	PHAR
Methylphenidate	113-45-1	Sigma-Aldrich	M-083-1ML	PHAR
Methyltestosterone	58-18-4	Sigma-Aldrich	M-906-1ML	PHAR
Metoprolol	51384-51-1	Sigma-Aldrich	PHR1076-1G	PHAR
Molindone	7416-34-4	Toronto Research Chemicals	M487500	PHAR
Monuron	150-68-5	AccuStandard	M-632-14	PHAR
Morphine	57-27-2	Sigma-Aldrich	M-005-1ML	PHAR
Mycophenolic Acid	24280-93-1	Sigma-Aldrich	M3536-50MG	PHAR
Nadolol	42200-33-9	Sigma-Aldrich	N1892-1G	PHAR
Nalidixic Acid	389-08-2	Sigma-Aldrich	97023-100MG	PHAR
Nalorphine	62-67-9	Sigma-Aldrich	N-924-1ML	PHAR
Naloxone	465-65-6	Sigma-Aldrich	N-004-1ML	PHAR
Naproxen	22204-53-1	Toronto Research Chemicals	N377520	PHAR
N-Ethylamphetamine	33817-11-7	Sigma-Aldrich	E-018-1ML	PHAR
Nevirapine	129618-40-2	Toronto Research Chemicals	N391275	PHAR
Nicotine	54-11-5	Sigma-Aldrich	N-008-1ML	PHAR
Norgestrel	6533-00-2	Toronto Research Chemicals	N689500	PHAR
Nortriptyline	72-69-5	Sigma-Aldrich	N7261-10G	PHAR
Ondansetron	99614-02-5	Toronto Research Chemicals	O655005	PHAR
Oxcarbazepine	28721-07-5	Sigma-Aldrich	O-025-1ML	PHAR
Oxprenolol	6452-71-7	Toronto Research Chemicals	O870500	PHAR
Oxycodone	76-42-6	Sigma-Aldrich	O-002-1ML	PHAR
Oxymorphone	76-41-5	Sigma-Aldrich	O-004-1ML	PHAR
Paliperidone	144598-75-4	Sigma-Aldrich	P0099-10MG	PHAR
Pentazocine	359-83-1	Sigma-Aldrich	P-073-1ML	PHAR
Pentedrone	879722-57-3	Sigma-Aldrich	P-087-1ML	PHAR
Phencyclidine	77-10-1	Sigma-Aldrich	P-007-1ML	PHAR
Phendimetrazine	634-03-7	Sigma-Aldrich	P-127-1ML	PHAR
Phenmetrazine	134-49-6	Sigma-Aldrich	P-128-1ML	PHAR
Phentermine	122-09-8	Sigma-Aldrich	P-023-1ML	PHAR
Phenylephrine	59-42-7	Sigma-Aldrich	PHR1017-500MG	PHAR
Phenytoin	57-41-0	Sigma-Aldrich	P-063-1ML	PHAR
Pilocarpine	92-13-7	Sigma-Aldrich	PHR1493-500MG	PHAR
Pirlimycin	79548-73-5	Toronto Research Chemicals	P509305	PHAR
Prasterone (Dehydroepiandrosterone; DHEA)	53-43-0	Sigma-Aldrich	D-063-1ML	PHAR
Praziquantel	55268-74-1	Toronto Research Chemicals	P702095	PHAR
Pregabalin	148553-50-8	Sigma-Aldrich	P-066-1ML	PHAR

Table S1. List of unlabeled reference standards and isotope-labeled internal standards (continued)

Compound Name	CAS	Supplier	Catalog Number	Category
Prilocaine	721-50-6	Sigma-Aldrich	P9547-1G	PHAR
Primidone	125-33-7	Sigma-Aldrich	P-075-1ML	PHAR
Propafenone	54063-53-5	Sigma-Aldrich	P4670-5G	PHAR
Propoxyphene	469-62-5	Sigma-Aldrich	P-011-1ML	PHAR
Propranolol	525-66-6	Sigma-Aldrich	P0884-1G	PHAR
Protriptyline	438-60-8	Sigma-Aldrich	P8813-100MG	PHAR
Pseudoephedrine	90-82-4	Sigma-Aldrich	P-035-1ML	PHAR
Pyrovalerone	3563-49-3	Sigma-Aldrich	P-081-1ML	PHAR
Quinine	130-95-0	Toronto Research Chemicals	Q694000	PHAR
Ramipril	87333-19-5	Sigma-Aldrich	PHR1446-1G	PHAR
Ranitidine	66357-35-5	Sigma-Aldrich	R101-1G	PHAR
Rimantadine	13392-28-4	Toronto Research Chemicals	R517000	PHAR
Ropivacaine	84057-95-4	Sigma-Aldrich	R0283-10MG	PHAR
Secobarbital	76-73-3	Sigma-Aldrich	S-002-1ML	PHAR
Sertraline	79617-96-2	Sigma-Aldrich	S-021-1ML	PHAR
Sitagliptin	486460-32-6	Sigma-Aldrich	PHR1857-1G	PHAR
Sotalol	3930-20-9	Toronto Research Chemicals	S677300	PHAR
Stavudine	3056-17-5	Toronto Research Chemicals	S685250	PHAR
Sulfadoxine	2447-57-6	Toronto Research Chemicals	S699070	PHAR
Sulfamethazine	57-68-1	Sigma-Aldrich	S6256-25G	PHAR
Sulfamethoxazole	723-46-6	Sigma-Aldrich	S7507-10G	PHAR
Sulfapyridine	144-83-2	Sigma-Aldrich	31738-250MG	PHAR
Sulfisomidine	515-64-0	Sigma-Aldrich	46908-250MG-R	PHAR
Tacrolimus	104987-11-3	Sigma-Aldrich	T-049-1ML	PHAR
Tapentadol	175591-23-8	Sigma-Aldrich	T-058-1ML	PHAR
Telmisartan	144701-48-4	Sigma-Aldrich	PHR1855-500MG	PHAR
Thebaine	115-37-7	Sigma-Aldrich	T-116-1ML	PHAR
Tolperisone	728-88-1	Sigma-Aldrich	T3577-50MG	PHAR
Tramadol	27203-92-5	Sigma-Aldrich	T-027-1ML	PHAR
Trenbolone	10161-33-8	Sigma-Aldrich	T-043-1ML	PHAR
Triamterene	396-01-0	Sigma-Aldrich	T4143-10G	PHAR
Trihexyphenidyl	144-11-6	Sigma-Aldrich	T1516-5G	PHAR
Trimethoprim	738-70-5	Sigma-Aldrich	PHR1056-1G	PHAR
Valsartan	137862-53-4	Toronto Research Chemicals	V095750	PHAR
Varenicline	249296-44-4	Sigma-Aldrich	PZ0004-5MG	PHAR
Venlafaxine	93413-69-5	Sigma-Aldrich	PHR1736-1G	PHAR
Zidovudine	30516-87-1	Toronto Research Chemicals	A825000	PHAR
10,11-Dihydro-10-hydroxy Carbamazepine	29331-92-8	Sigma-Aldrich	D-091-1ML	PHAR TP
1-Methyluric Acid	708-79-2	Toronto Research Chemicals	M338180	PHAR TP
2-Ethyl-2-phenylmalonamide (PEMA)	7206-76-0	Sigma-Aldrich	95923-25MG	PHAR TP
2-Ethylidene-1,5-dimethyl-3,3-diphenylpyrrolidine (EDDP)	30223-73-5	Sigma-Aldrich	E-022-1ML	PHAR TP
Albendazole-2-aminosulfone	80983-34-2	Toronto Research Chemicals	A580950	PHAR TP
Albendazole Sulfoxide	54029-12-8	Toronto Research Chemicals	A511620	PHAR TP
3-Hydroxy Cotinine	34834-67-8	Sigma-Aldrich	H-101-1ML	PHAR TP
Androsterone	53-41-8	Sigma-Aldrich	31579-250MG	PHAR TP
Benzoylecgonine	519-09-5	Sigma-Aldrich	B-004-1ML	PHAR TP
Carbamazepine-10,11-epoxide	36507-30-9	Sigma-Aldrich	C-121-1ML	PHAR TP
Cotinine	486-56-6	Toronto Research Chemicals	C725000	PHAR TP
Cotinine N-Oxide	36508-80-2	Toronto Research Chemicals	C725200	PHAR TP

Table S1. List of unlabeled reference standards and isotope-labeled internal standards (continued)

Compound Name	CAS	Supplier	Catalog Number	Category
Ecgonine Methyl Ester	7143-09-1	Sigma-Aldrich	E-001-1ML	PHAR TP
Hydroxybupropion	92264-81-8	Sigma-Aldrich	H-066-1ML	PHAR TP
Ibuprofen Carboxylic Acid (Carboxyibuprofen)	15935-54-3	Toronto Research Chemicals	I140015	PHAR TP
Lidocaine N-Oxide	2903-45-9	Toronto Research Chemicals	L397815	PHAR TP
Metoprolol Acid (Atenolol Acid)	56392-14-4	Toronto Research Chemicals	M338785	PHAR TP
N4-Acetylsulfamethoxazole	21312-10-7	Toronto Research Chemicals	A187885	PHAR TP
N-Desmethyl Citalopram	62498-67-3	Sigma-Aldrich	D-047-1ML	PHAR TP
N-Desmethyl Tramadol	73806-55-0	Sigma-Aldrich	D-023-1ML	PHAR TP
N-Desmethyl Venlafaxine	149289-30-5	Toronto Research Chemicals	M266250	PHAR TP
Norcodeine	467-15-2	Sigma-Aldrich	N-005-1ML	PHAR TP
Norfentanyl	1609-66-1	Sigma-Aldrich	N-031-1ML	PHAR TP
Norlidocaine (Monoethylglycinexylidide)	7728-40-7	Toronto Research Chemicals	N713500	PHAR TP
Noroxycodone	57664-96-7	Sigma-Aldrich	N-011-1ML	PHAR TP
O-Desmethyl Tramadol	73986-53-5	Sigma-Aldrich	T-035-1ML	PHAR TP
Ritalinic Acid	19395-41-6	Sigma-Aldrich	R-011-1ML	PHAR TP
Tramadol N-Oxide	147441-56-3	Toronto Research Chemicals	T712530	PHAR TP
Venlafaxine N-Oxide	1094598-37-4	Toronto Research Chemicals	V120020	PHAR TP
(4-Chloro-2-methylphenoxy)acetic Acid (MCPA)	94-74-6	AccuStandard	P-153S	PEST
2,4-Dichlorophenoxyacetic Acid (2,4-D)	94-75-7	Sigma-Aldrich	31518-250MG	PEST
2-Naphthoxyacetic Acid (NOA)	120-23-0	Sigma-Aldrich	N3019-25G	PEST
8-Hydroxyquinoline	148-24-3	Sigma-Aldrich	H6878-25G	PEST
Abscisic Acid	21293-29-8	Sigma-Aldrich	90769-25MG	PEST
Acetamiprid	135410-20-7	AccuStandard	P-820S-CN	PEST
Aldicarb	116-06-3	AccuStandard	M-8318-01	PEST
Ametryn	834-12-8	AccuStandard	M-619-01	PEST
Atrazine	1912-24-9	Sigma-Aldrich	45330-250MG-R	PEST
Bifenazate	149877-41-8	AccuStandard	P-772S	PEST
Butralin	33629-47-9	Sigma-Aldrich	36528-250MG	PEST
Camphor	76-22-2	AccuStandard	CP-TER-016S	PEST
Carbaryl	63-25-2	AccuStandard	M-8318-03	PEST
Carbendazim	10605-21-7	AccuStandard	P-278S	PEST
Carbofuran	1563-66-2	AccuStandard	M-8318-04	PEST
Clothianidin	210880-92-5	AccuStandard	P-947S	PEST
Dimethachlor	50563-36-5	AccuStandard	P-642S	PEST
Diuron	330-54-1	AccuStandard	Z-031-07	PEST
Ethoxyquin	91-53-2	AccuStandard	P-388S-CN	PEST
Ethyl Butylacetylaminopropionate	52304-36-6	Toronto Research Chemicals	E900625	PEST
Fenamidone	161326-34-7	AccuStandard	P-850S-CN	PEST
Fluridone	59756-60-4	AccuStandard	P-193S	PEST
Imazapyr	81334-34-1	AccuStandard	P-589S	PEST
Imidacloprid	138261-41-3	AccuStandard	P-596S	PEST
Indole-3-butyric Acid	133-32-4	Sigma-Aldrich	I5386-1G	PEST
Isoproturon	34123-59-6	AccuStandard	P-302S	PEST
Kresoxim-methyl	143390-89-0	Toronto Research Chemicals	K659000	PEST
Malathion	121-75-5	AccuStandard	P-060S	PEST
Mecoprop (MCPP)	93-65-2	AccuStandard	P-1053S-A	PEST
Metalaxyl	57837-19-1	AccuStandard	P-120S	PEST
Metolachlor	51218-45-2	Sigma-Aldrich	36163-100MG	PEST
Napropamide	15299-99-7	AccuStandard	P-179S	PEST

Table S1. List of unlabeled reference standards and isotope-labeled internal standards (continued)

Compound Name	CAS	Supplier	Catalog Number	Category
Oxamyl	23135-22-0	AccuStandard	P-161S	PEST
Piperonyl Butoxide	51-03-6	AccuStandard	P-348S	PEST
Pirimicarb	23103-98-2	AccuStandard	P-304S	PEST
Prometon	1610-18-0	Sigma-Aldrich	45635-50MG	PEST
Prometryn	7287-19-6	Sigma-Aldrich	45636-50MG	PEST
Propazine	139-40-2	Sigma-Aldrich	45640-250MG	PEST
Pyracarbolid	24691-76-7	AccuStandard	P-792S-CN	PEST
Pyrimethanil	53112-28-0	Sigma-Aldrich	31577-250MG	PEST
Siduron	1982-49-6	AccuStandard	P-063S	PEST
Simazine	122-34-9	Sigma-Aldrich	32059-250MG	PEST
Terbumeton	33693-04-8	Sigma-Aldrich	31527-250MG	PEST
Terbuthylazine	5915-41-3	AccuStandard	M-619-10	PEST
Thiabendazole	148-79-8	AccuStandard	P-068S	PEST
Terbutryn	886-50-0	AccuStandard	M-619-11	PEST
Thiamethoxam	153719-23-4	AccuStandard	P-866S-CN	PEST
Tridemorph	24602-86-6	AccuStandard	P-307S	PEST
Trinexapac-ethyl	95266-40-3	AccuStandard	P-1034S	PEST
2-Aminobenzimidazole	934-32-7	Sigma-Aldrich	171778-5G	PEST TP
Atrazine-2-hydroxy	2163-68-0	AccuStandard	P-326S	PEST TP
Atrazine-desethyl	6190-65-4	AccuStandard	P-343S	PEST TP
Atrazine-desisopropyl	1007-28-9	AccuStandard	P-345S	PEST TP
Carbofuran-3-hydroxy	16655-82-6	AccuStandard	M-8318-06	PEST TP
Metolachlor Ethanesulfonic Acid (Metolachlor ESA)	171118-09-5	Sigma-Aldrich	34149-10MG	PEST TP
Metolachlor Oxanilic Acid (Metolachlor OA)	152019-73-3	Sigma-Aldrich	34148-10MG	PEST TP
N-(2,4-Dimethylphenyl)formamide	60397-77-5	Sigma-Aldrich	592587-1G	PEST TP
Propachlor Oxanilic Acid (Propachlor OA)	70628-36-3	AccuStandard	P-921S	PEST TP
2-Hydroxybenzothiazole	934-34-9	Sigma-Aldrich	407607-5G	PCHI
4-Methyl-1H-benzotriazole	29878-31-7	Sigma-Aldrich	14593-50MG	PCHI
5-Methyl-1H-benzotriazole	136-85-6	Sigma-Aldrich	196304-10G	PCHI
Benzophenone	119-61-9	Sigma-Aldrich	B9300-25G-A	PCHI
Benzophenone-3 (Oxybenzone)	131-57-7	Sigma-Aldrich	H36206-5G	PCHI
Benzothiazole	95-16-9	Sigma-Aldrich	101338-5G	PCHI
Benzotriazole	95-14-7	Sigma-Aldrich	B11400-100G	PCHI
Benzyl Butyl Phthalate	85-68-7	Sigma-Aldrich	308501-5ML	PCHI
Butylparaben	94-26-8	AccuStandard	ALR-085S	PCHI
Dibutyl Phthalate	84-74-2	Sigma-Aldrich	524980-25ML	PCHI
Diethyl Phthalate	84-66-2	Sigma-Aldrich	524972-5ML	PCHI
Diisobutyl Phthalate	84-69-5	Sigma-Aldrich	152641-100ML	PCHI
Dimethyl Phthalate	131-11-3	Sigma-Aldrich	41320-1ML-F	PCHI
Ethylparaben	120-47-8	AccuStandard	ALR-113S	PCHI
Icaridin	119515-38-7	AccuStandard	BIOC-228S-CN	PCHI
Isopropylparaben	4191-73-5	AccuStandard	ALR-122S	PCHI
Melamine	108-78-1	Sigma-Aldrich	M2659-5G	PCHI
N,N-Diethyl-3-methylbenzamide (DEET)	134-62-3	Sigma-Aldrich	36542-250MG	PCHI
Propylparaben	94-13-3	AccuStandard	ALR-153S	PCHI
Sucralose	56038-13-2	Toronto Research Chemicals	S692500	PCHI
Triclosan	3380-34-5	Sigma-Aldrich	72779-5G-F	PCHI
3-Cyclohexyl-1,1-dimethylurea (C-DMU)	31468-12-9	Sigma-Aldrich	S604542-250MG	PCHI
1,1-Dicyclohexyl-3-phenylurea	5765-54-8	Sigma-Aldrich	S611131-250MG	PCHI

Table S1. List of unlabeled reference standards and isotope-labeled internal standards (continued)

Compound Name	CAS	Supplier	Catalog Number	Category
1,3-Di-o-tolylguanidine (DTG)	97-39-2	TCI America	D095325G	PCHI
1,3-Diphenylguanidine (N,N'-Diphenylguanidine; DPG)	102-06-7	TCI America	D089225G	PCHI
4-Methylbenzenesulfonamide	70-55-3	TCI America	T028125G	PCHI
2-Phenyl-2-imidazoline	936-49-2	Alfa Aesar	AAH5909509	PCHI
2-Phenylbenzimidazole-5-sulfonic Acid (Ensulizole)	27503-81-7	Thermo Scientific Chemicals	AC259700050	PCHI
Caprolactam	105-60-2	TCI America	C020325G	PCHI
N-(1,3-Dimethylbutyl)-N'-phenyl-1,4-phenylenediamine (6PPD)	793-24-8	TCI America	D333125G	PCHI
N-Cyclohexyl-N-methylcyclohexanamine (N,N-Dicyclohexylmethylamine; DCA)	7560-83-0	TCI America	D082025ML	PCHI
N-Cyclohexyl-N'-phenylurea (1-Cyclohexyl-3-phenylurea; CPU)	886-59-9	Sigma-Aldrich	S591327-250MG	PCHI
N-Ethylphthalimide	5022-29-7	Alfa Aesar	AAA1601406	PCHI
N-Ethyl-p-toluenesulfonamide	80-39-7	TCI America	E067425G	PCHI
N-Isopropyl-N'-phenyl-p-phenylenediamine (IPPD)	101-72-4	TCI America	P032725G	PCHI
N-Phenylbenzenesulfonamide	1678-25-7	TCI America	B002925G	PCHI
N-Phenyl-p-phenylenediamine (4-Aminodiphenylamine)	101-54-2	TCI America	P020325G	PCHI
N,N'-Diphenyl-p-phenylenediamine (DPPD)	74-31-7	TCI America	D060925G	PCHI
Hexa(methoxymethyl)melamine (HMMM)	3089-11-0	TCI America	T20595G	PCHI
Triphenylphosphine Oxide	791-28-6	Thermo Scientific Chemicals	AC140430250	PCHI
2,4-Quinolinediol (2,4-Dihydroxyquinoline)	86-95-3	TCI America	D175325G	PCHI
2,6-Dimethoxyphenol	91-10-1	TCI America	D063925G	PCHI
4-Methylbenzophenone	134-84-9	TCI America	M124825G	PCHI
Octadecylamine	124-30-1	Alfa Aesar	AAL1545814	PCHI
Acridine	260-94-6	Alfa Aesar	AAL0165706	PCHI
Berberine	633-65-8	Alfa Aesar	AAJ6231106	PCHI
Bis(2-ethylhexyl) Adipate	103-23-1	AccuStandard	P-233S	PCHI
Daidzein	486-66-8	Thermo Scientific Chemicals	AC328230250	PCHI
Dextrorphan	125-73-5	Cayman Chemical Company	15886	PCHI
Diheptyl Phthalate	3648-21-3	AccuStandard	PHTH-020S	PCHI
Di(propylene glycol) Dibenzoate	27138-31-4	AccuStandard	PLAS-PL-101S	PCHI
Docosahexaenoic Acid	6217-54-5	Toronto Research Chemicals	D494500	PCHI
Hexadecyltrimethylammonium (Cetrimonium)	112-02-7	TCI America	H008225G	PCHI
Lauramidopropyl Betaine	86438-79-1	Santa Cruz Biotechnology	sc-357318	PCHI
Mono(2-ethylhexyl) Phthalate (Monoethylhexyl Phthalate)	4376-20-9	AccuStandard	ALR-138N	PCHI
Monolaurin	42-18-7	TCI America	G00815G	PCHI
Myristyl Sulfate (Tetradecyl Sulfate)	1191-50-0	Alfa Aesar	AAB2194103	PCHI
N-Ethyl-p-menthane-3-carboxamide	39711-79-0	TCI America	E07965G	PCHI
Nobiletin	478-01-3	Cayman Chemical Company	15421	PCHI
Phthalic Acid	88-99-3	TCI America	P028725G	PCHI
Octadecanamide (Stearamide)	124-26-5	TCI America	S007525G	PCHI
Tributyl Citrate	77-94-1	Alfa Aesar	AAA1026618	PCHI
Tris(2-butoxyethyl) Phosphate (TBEP)	78-51-3	AccuStandard	PLAS-PL-104S	PCHI
ar-Turmerone	532-65-0	Toronto Research Chemicals	T897275	PCHI
1-Methyl-1H-benzotriazole	13351-73-0	Toronto Research Chemicals	M289810	PCHI TP
Carbanilide	102-07-8	Sigma-Aldrich	142158-25G	PCHI TP
Galaxolidone	507442-49-1	Toronto Research Chemicals	G189005	PCHI TP
3,4-Methylenedioxymethamphetamine-d_5	136765-43-0	Sigma-Aldrich	M-011-1ML	ILIS
Abacavir-d_4	1217731-56-0	Toronto Research Chemicals	A105002	ILIS
Acetaminophen-d_3 (N-(4-Hydroxyphenyl)acetamide-2,2,2-d_3)	60902-28-5	C/D/N Isotopes	D-6324	ILIS
Amantadine-d_{15}	33830-10-3	C/D/N Isotopes	D-7965	ILIS
Amphetamine-d_{10}	169565-17-7	Cambridge Isotope Laboratories	A-038-1ML	ILIS

Table S1. List of unlabeled reference standards and isotope-labeled internal standards (continued)

Compound Name	CAS	Supplier	Catalog Number	Category
Atenolol-d_7	1202864-50-3	C/D/N Isotopes	D-6202	ILIS
Buprenorphine-d_4	136781-89-0	Sigma-Aldrich	B-901-1ML	ILIS
Bupropion-d_9	1189725-26-5	Sigma-Aldrich	B-052-1ML	ILIS
Caffeine-d_9	72238-85-8	C/D/N Isotopes	D-5972	ILIS
Carbamazepine-d_{10}	132183-78-9	C/D/N Isotopes	D-3542	ILIS
Cetirizine-d_8	2070015-04-0	Toronto Research Chemicals	C281102	ILIS
Cimetidine-d_3	1185237-29-9	C/D/N Isotopes	D-6876	ILIS
Clarithromycin-d_3	NA	Toronto Research Chemicals	C559752	ILIS
Codeine-d_3	70420-71-2	Sigma-Aldrich	C-005-1ML	ILIS
Cotinine-d_3	110952-70-0	C/D/N Isotopes	D-3518	ILIS
Diclofenac-d_4	153466-65-0	C/D/N Isotopes	D-6183	ILIS
Diphenhydramine-d_3	170082-18-5	Sigma-Aldrich	D-017-1ML	ILIS
Dopamine-d_4	203633-19-6	Cambridge Isotope Laboratories	D-072-1ML	ILIS
Ephedrine-d_3	285979-73-9	Sigma-Aldrich	E-025-1ML	ILIS
Fentanyl-d_5	118357-29-2	Cambridge Isotope Laboratories	F-001-1ML	ILIS
Fexofenadine-d_6	548783-71-7	Toronto Research Chemicals	F322500	ILIS
Fluconazole-$^{13}C_3$	NA	Sigma-Aldrich	F-035-1ML	ILIS
Fluoxetine-d_5	1173020-43-3	C/D/N Isotopes	D-5749	ILIS
Gabapentin-d_{10}	1126623-20-8	Cambridge Isotope Laboratories	G-901-1ML	ILIS
Gemfibrozil-d_6	1184986-45-5	C/D/N Isotopes	D-6144	ILIS
Hydrocodone-d_3	136765-36-1	Sigma-Aldrich	H-005-1ML	ILIS
Hydromorphone-d_3	136765-37-2	Sigma-Aldrich	H-006-1ML	ILIS
Lamotrigine-$^{13}C,^{15}N_4$	NA	Cambridge Isotope Laboratories	L-022-1ML	ILIS
Levetiracetam-d_6	1435933-72-4	Cambridge Isotope Laboratories	L-023-1ML	ILIS
Lidocaine-d_{10}	851528-09-1	C/D/N Isotopes	D-6745	ILIS
Losartan-d_4	1030937-27-9	Toronto Research Chemicals	L470503	ILIS
Meperidine-d_4	53484-73-4	Sigma-Aldrich	M-036-1ML	ILIS
Metaxalone-d_3	1192812-66-0	C/D/N Isotopes	D-6414	ILIS
Metformin-d_6	1185166-01-1	Sigma-Aldrich	53183-5MG	ILIS
Methadone-d_3	60263-63-0	Cambridge Isotope Laboratories	M-008-1ML	ILIS
Methamphetamine-d_8	136765-40-7	Cambridge Isotope Laboratories	M-016-1ML	ILIS
Methocarbamol-d_3	1346600-86-9	Sigma-Aldrich	M-202-1ML	ILIS
Metoprolol-d_7	1219798-61-4	C/D/N Isotopes	D-6682	ILIS
Morphine-d_3	67293-88-3	Cambridge Isotope Laboratories	M-003-1ML	ILIS
Naproxen-d_3	958293-77-1	C/D/N Isotopes	D-6523	ILIS
Nicotine-d_4	350818-69-8	Cambridge Isotope Laboratories	N-048-1ML	ILIS
Oxycodone-d_3	160227-46-3	Cambridge Isotope Laboratories	O-005-1ML	ILIS
Oxymorphone-d_3	145225-03-2	Sigma-Aldrich	O-003-1ML	ILIS
Phendimetrazine-d_5	NA	Sigma-Aldrich	P-132-1ML	ILIS
Phenytoin-d_{10}	65854-97-9	C/D/N Isotopes	D-2158	ILIS
Phentermine-d_5	1330236-21-9	Sigma-Aldrich	P-034-1ML	ILIS
Pregabalin-d_6	NA	Cambridge Isotope Laboratories	P-072-1ML	ILIS
Protriptyline-d_3	1435934-21-6	Cambridge Isotope Laboratories	P-088-1ML	ILIS
Sulfamethoxazole-d_4	1020719-86-1	C/D/N Isotopes	D-7398	ILIS
Theophylline-d_6	117490-39-8	Toronto Research Chemicals	T343853	ILIS
Tramadol-$^{13}C,d_3$	NA	Cambridge Isotope Laboratories	T-029-1ML	ILIS
Trimethoprim-d_9	1189460-62-5	Sigma-Aldrich	32414-10MG	ILIS
Valsartan-d_3	1331908-02-1	Toronto Research Chemicals	V095752	ILIS
Venlafaxine-d_6	1062606-12-5	C/D/N Isotopes	D-6826	ILIS

Table S1. List of unlabeled reference standards and isotope-labeled internal standards (continued)

Compound Name	CAS	Supplier	Catalog Number	Category
2-Ethylidene-1,5-dimethyl-3,3-diphenylpyrrolidine-d_3 (EDDP-d_3)	136765-23-6	Cambridge Isotope Laboratories	E-021-1ML	ILIS
3-Hydroxy Cotinine-d_3	159956-78-2	Cambridge Isotope Laboratories	H-108-1ML	ILIS
Benzoylecgonine-d_3	115732-68-8	Cambridge Isotope Laboratories	B-001-1ML	ILIS
Ecgonine Methyl Ester-d_3	136765-34-9	Sigma-Aldrich	E-002-1ML	ILIS
Hydroxybupropion-d_6	1184984-06-2	Cambridge Isotope Laboratories	H-062-1ML	ILIS
Norcodeine-d_3	NA	Sigma-Aldrich	N-082-1ML	ILIS
Norfentanyl-d_5	1211527-23-9	Sigma-Aldrich	N-030-1ML	ILIS
Norhydrocodone-d_3	NA	Sigma-Aldrich	N-054-1ML	ILIS
Noroxycodone-d_3	1426174-79-9	Cambridge Isotope Laboratories	N-032-1ML	ILIS
Ritalinic Acid-d_{10}	NA	Cambridge Isotope Laboratories	R-014-1ML	ILIS
(4-Chloro-2-methylphenoxy-d_3)acetic Acid (MCPA-d_3)	352431-14-2	C/D/N Isotopes	D-5320	ILIS
(2,4-Dichlorophenoxy-d_3)acetic Acid (2,4-D-d_3)	202480-67-9	C/D/N Isotopes	D-5750	ILIS
Acetamiprid-d_3	1353869-35-8	C/D/N Isotopes	D-7632	ILIS
Atrazine-d_5	163165-75-1	C/D/N Isotopes	D-4389	ILIS
Carbaryl-d_7	362049-56-7	C/D/N Isotopes	D-5468	ILIS
Carbendazim-d_4	291765-95-2	C/D/N Isotopes	D-6302	ILIS
Clofibric Acid-d_4	1184991-14-7	C/D/N Isotopes	D-6005	ILIS
Clothianidin-d3	1262776-24-8	Toronto Research Chemicals	C588501	ILIS
Diuron-d_6	1007536-67-5	Sigma-Aldrich	34018-10MG-R	ILIS
Imidacloprid-d_4	1015855-75-0	C/D/N Isotopes	D-7456	ILIS
Mecoprop-d_3	352431-15-3	C/D/N Isotopes	D-5321	ILIS
Metalaxyl-d_3	NA	Sigma-Aldrich	08963-5MG	ILIS
Metolachlor-d_6	1219803-97-0	C/D/N Isotopes	D-5647	ILIS
Prometon-d_3	1219803-43-6	C/D/N Isotopes	D-6802	ILIS
Simazine-d_{10}	220621-39-6	C/D/N Isotopes	D-5654	ILIS
Thiamethoxam-d_3	1294048-82-0	C/D/N Isotopes	D-7457	ILIS
Benzophenone-d_{10}	22583-75-1	C/D/N Isotopes	D-0263	ILIS
Benzothiazole-d_4	194423-51-3	Toronto Research Chemicals	B206642	ILIS
Benzotriazole-d_4	1185072-03-0	C/D/N Isotopes	D-7358	ILIS
Benzyl Butyl Phthalate-d_4	93951-88-3	C/D/N Isotopes	D-2940	ILIS
Caprolactam-d_{10}	169297-53-4	C/D/N Isotopes	D-5253	ILIS
N,N-Diethyl-3-methyl-d_3-benzamide-2,4,5,6-d_4 (DEET-d_7)	1219799-37-7	C/D/N Isotopes	D-6756	ILIS
Diethyl Phthalate-d_{14}	99873-99-1	C/D/N Isotopes	D-5533	ILIS
Dimethyl Phthalate-d_6	85448-30-2	C/D/N Isotopes	D-2413	ILIS
Melamine-$^{13}C_3$,$^{15}N_3$	1246816-14-7	Toronto Research Chemicals	M208704	ILIS
Oxybenzone-d_5	1219798-54-5	Sigma-Aldrich	73875-10MG	ILIS
Propylparaben-d_4	1219802-67-1	C/D/N Isotopes	D-7114	ILIS
Sucralose-d_6	1459161-55-7	Toronto Research Chemicals	S692502	ILIS
Triclosan-d_3	1020719-98-5	C/D/N Isotopes	D-6983	ILIS
Tris(2-chloroethyl) Phosphate-d_{12}	1276500-47-0	C/D/N Isotopes	D-8091	ILIS

"PHAR" = pharmaceutical; "PEST" = pesticide; "PCHI" = personal care, household and industrial chemical; "TP" = transformation product; "ILIS" = isotope-labeled internal standard.

S2. Stream sampling events and watershed attributes

Table S2. Summary of stream site coordinates and sampling dates

Site Name [Watershed ID]	Site Coordinates	POCIS and Grab Sampling Dates	USGS Gauge Station
Butternut Creek [1]	43.043900, -76.049858	POCIS: 7/26/2018 – 8/20/2018; 6/11/2019 – 7/3/2019<br>Grab: 7/26/2018; 8/20/2018; 6/11/2019; 7/3/2019	04245200 (discontinued)
Chenango River [2]	42.678442, -75.510378	POCIS: 9/1/2018 – 9/21/2018; 6/14/2019 – 7/8/2019<br>Grab: 9/1/2018; 9/21/2018; 6/14/2019; 7/8/2019	01505000
Chittenango Creek [3]	43.010683, -75.850548	POCIS: 7/26/2018 – 8/20/2018; 5/6/2019 – 5/27/2019<br>Grab: 7/26/2018; 8/20/2018; 5/6/2019; 5/27/2019	04244000
Conesus Creek [4]	42.853971, -77.715343	POCIS: 8/1/2018 – 8/22/2018; 5/8/2019 – 5/29/2019<br>Grab: 8/1/2018; 8/22/2018; 5/8/2019; 5/29/2019	04227995
Cowaselon Creek [5]	43.095539, -75.751190	POCIS: 9/1/2018 – 9/21/2018; 6/11/2019 – 7/3/2019<br>Grab: 9/1/2018; 9/21/2018; 6/11/2019; 7/3/2019	04243783
Flint Creek [6]	42.957429, -77.061685	POCIS: 8/1/2018 – 8/22/2018; 5/8/2019 – 5/29/2019<br>Grab: 8/1/2018; 8/22/2018; 5/8/2019; 5/29/2019	04235250
Ganargua Creek [7]	43.067652, -77.298232	POCIS: 8/1/2018 – 8/22/2018; 5/8/2019 – 5/29/2019<br>Grab: 8/1/2018; 8/22/2018; 5/8/2019; 5/29/2019	04234254
Harbor Brook [8]	43.056336, -76.185324	POCIS: 7/26/2018 – 8/20/2018; 6/11/2019 – 7/3/2019<br>Grab: 7/26/2018; 8/20/2018; 6/11/2019; 7/3/2019	04240100
Honeoye Creek [9]	42.946213, -77.583595	POCIS: 8/1/2018 – 8/22/2018; 5/8/2019 – 5/29/2019<br>Grab: 8/1/2018; 8/22/2018; 5/8/2019; 5/29/2019	04229500
Ninemile Creek at Lakeland [10]	43.081017, -76.226575	POCIS: 7/26/2018 – 8/20/2018; 6/11/2019 – 7/3/2019<br>Grab: 7/26/2018; 8/20/2018; 6/11/2019; 7/3/2019	04240300
Ninemile Creek at Marietta [11]	42.920901, -76.329807	POCIS: 9/1/2018 – 9/21/2018; 6/11/2019 – 6/14/2019; 6/14/2019 – 6/18/2019; 6/18/2019 – 7/3/2019; 6/11/2019 – 7/3/2019<br>Grab: 9/1/2018; 9/21/2018; 6/11/2019; 6/14/2019; 6/18/2019; 6/21/2019; 7/3/2019	04240180 (discontinued)
Oatka Creek [12]	43.010555, -77.794794	POCIS: 8/1/2018 – 8/22/2018; 5/8/2019 – 5/29/2019<br>Grab: 8/1/2018; 8/22/2018; 5/8/2019; 5/29/2019	04230500
Oneida Creek [13]	43.097652, -75.639657	POCIS: 9/1/2018 – 9/21/2018; 5/6/2019 – 5/27/2019<br>Grab: 9/1/2018; 9/21/2018; 5/6/2019; 5/27/2019	04243500
Oriskany Creek [14]	43.155338, -75.334414	POCIS: 9/1/2018 – 9/21/2018; 5/6/2019 – 5/27/2019<br>Grab: 9/1/2018; 9/21/2018; 5/6/2019; 5/27/2019	01338000
Otselic River [15]	42.541647, -75.899253	POCIS: 9/1/2018 – 9/21/2018; 6/14/2019 – 7/8/2019<br>Grab: 9/1/2018; 9/21/2018; 6/14/2019; 7/8/2019	01510000
Salmon Creek [16]	42.550328, -76.539050	POCIS: 9/1/2018 – 9/21/2018; 5/6/2019 – 5/27/2019<br>Grab: 9/1/2018; 9/21/2018; 5/6/2019; 5/27/2019	0423401815
Sauquoit Creek [17]	43.111839, -75.293933	POCIS: 9/1/2018 – 9/21/2018; 5/6/2019 – 5/27/2019<br>Grab: 9/1/2018; 9/21/2018; 5/6/2019; 5/27/2019	01339060
Scriba Creek [18]	43.248707, -75.998785	POCIS: 7/26/2018 – 8/20/2018; 6/11/2019 – 7/3/2019<br>Grab: 7/26/2018; 8/20/2018; 6/11/2019; 7/3/2019	04245840
Sixmile Creek [19]	42.436451, -76.488932	POCIS: 9/1/2018 – 9/21/2018; 5/6/2019 – 5/27/2019; 6/14/2019 – 7/8/2019<br>Grab: 9/1/2018; 9/21/2018; 5/6/2019; 5/27/2019; 6/14/2019; 7/8/2019	04233300
Skaneateles Creek [20]	43.015014, -76.472343	POCIS: 9/1/2018 – 9/21/2018; 5/6/2019 – 5/27/2019; 6/11/2019 – 6/14/2019; 6/14/2019 – 6/18/2019; 6/18/2019 – 6/21/2019; 6/21/2019 – 7/3/2019; 6/11/2019 – 7/3/2019<br>Grab: 9/1/2018; 9/21/2018; 5/6/2019; 5/27/2019; 6/11/2019; 6/14/2019; 6/18/2019; 6/21/2019; 7/3/2019	04236800

Table S3. Summary of watershed attributes for the 20 stream sites

Site Name [Watershed ID]	Watershed Area (km^2)	Agricultural Land Usage (%)[a]	Developed Land Usage (%)[b]	Septic Density (km^{-2})[c]	CSOs[d]	CAFOs[e]	Municipal WWTPs (MGD)[f]	Industrial WWTPs (MGD)[g]	Road Density (km/km^2)[h]	RPI[i]	Population Density (km^{-2})[j]
Butternut Creek [1]	162.3	11.6	16.9	44	0	1	0	2	3.53	1.14	331±10
Chenango River [2]	670.0	11.0	6.0	7	0	5	1.48	0	1.97	1.23	31±1
Chittenango Creek [3]	168.9	13.7	6.7	15	0	0	0.95	0	2.29	1.03	60±3
Conesus Creek [4]	174.0	20.9	6.7	22	0	1	0.98	0	2.44	1.23	83±4
Cowaselon Creek [5]	106.9	21.4	8.1	15	0	2	0	0	2.62	0.86	90±8
Flint Creek [6]	261.9	43.3	5.0	19	0	7	0.06	0	1.73	1.74	84±3
Ganargua Creek [7]	296.2	27.9	10.2	33	0	1	4.82	0.46	2.40	1.30	162±5
Harbor Brook [8]	31.1	6.9	48.5	69	19	0	0	0	6.57	0.70	1208±46
Honeoye Creek [9]	483.8	15.0	5.3	9	0	1	0.04	1.95	1.62	2.09	42±2
Ninemile Creek at Lakeland [10]	288.0	24.1	14.2	22	0	6	0.38	1.75	3.11	0.85	169±5
Ninemile Creek at Marietta [11]	107.9	27.9	5.8	7	0	3	0	0	2.22	1.50	32±2
Oatka Creek [12]	529.6	48.4	6.5	16	0	18	2.28	0.61	2.39	1.20	72±2
Oneida Creek [13]	294.0	27.0	8.0	14	0	5	1.76	0	1.80	1.08	71±3
Oriskany Creek [14]	372.7	28.3	8.5	16	0	4	3.39	4.17	1.96	0.94	82±3
Otselic River [15]	379.5	4.5	4.6	6	0	0	0	0	1.33	1.39	25±1
Salmon Creek [16]	229.4	54.0	5.1	16	0	13	0	0	1.84	1.62	65±4
Sauquoit Creek [17]	154.9	20.9	18.4	31	1	1	0	0.53	3.11	0.86	265±9
Scriba Creek [18]	104.9	0.6	3.6	20	0	0	0	0	1.10	1.14	78±5
Sixmile Creek [19]	125.4	1.3	7.2	41	0	0	0	0.45	2.50	1.17	202±11
Skaneateles Creek [20]	186.3	24.8	7.3	18	0	2	0.85	0.88	2.05	1.17	74±3

[a] Calculated by dividing the area of "Cultivated Crops" (as defined by the 2016 National Land Cover Database[2]) by the watershed area; [b] Calculated by dividing the summed area of "Developed, Open Space", "Developed, Low Intensity", "Developed, Medium Intensity", and "Developed, High Intensity" (as defined by the 2016 National Land Cover Database[2]) by the watershed area; [c] Calculated by dividing the total number of septic systems (derived from 2011 county parcel data by the New York State Water Resources Institute [3]) by the watershed area; [d] Defined as the total number of combined sewer overflow outfalls (CSOs) within the watershed (updated on November 10, 2020 by the New York State Department of Environmental Conservation[4]); [e] Defined as the total number of concentrated animal feeding operations (CAFOs) within the watershed (updated on February 7, 2016 by the New York State Department of Environmental Conservation[5]); [f] Calculated by summing the average design hydraulic flow (million gallons per day) of municipal wastewater treatment plants (WWTPs) within the watershed (updated on October 9, 2019 by the New York State Department of Environmental Conservation[6]); [g] Calculated by summing the average design hydraulic flow (million gallons per day) of industrial wastewater treatment plants (WWTPs) within the watershed (updated on October 9, 2019 by the New York State Department of Environmental Conservation[7]); [h] Calculated by dividing the summed road length within the watershed (calculated based on the 2019 TIGER/Line Shapefiles[8]) by the watershed area; [i] Runoff propensity index (RPI) of a watershed defined as $\log_{10}(Q_{90\text{th percentile}}/Q_{10\text{th percentile}})$ by Blanchard and Lerch.[9] [j] Calculated by dividing the population within the watershed (extracted from the U.S. Census Bureau's 2014-2018 American Community Survey[10] as estimates ± margins of error) by the watershed area.

S3. Analysis of physicochemical and optical properties of stream water samples

Upon return to the laboratory, grab samples were analyzed for a suite of physicochemical and optical properties. pH and specific conductance were measured by a Mettler Toledo SevenExcellence multi-channel meter with an InLab Science Pro ISM pH/ATC electrode and an InLab 731 conductivity probe, respectively. Chloride and nitrate were measured by a Thermo Scientific Integrion high-pressure ion chromatograph. Fluorescence excitation-emission matrices (EEMs) and UV-visible absorbance spectra were simultaneously measured in a Starna Cells 3-Q-10 quartz cuvette (1-cm pathlength) using a Horiba Scientific Aqualog spectrofluorometer as described in our previous work.[11] Optical indices[12-15] were extracted from the absorbance and EEM fluorescence data using an in-house *MATLAB* script, including Napierian absorption coefficients (a)[16] at 254 nm,[17] E_2:E_3 (the ratio of absorption coefficients at 250 and 365 nm),[18] fluorescence index (FI),[19, 20] humification index (HIX),[21-23] and freshness index (β:α).[24-26] Parallel factor analysis (PARAFAC) was conducted to deconvolute EEMs using the *drEEM* toolbox (v. 0.6.3)[27] in *MATLAB R2019a* as described in our previous work.[11] A 3-component model was identified via $S_4C_6T_3$ split-half validations[27] as the most robust model that explained 99.8% of the measured spectral variation across reverse-normalized EEMs (**Figure S1**). Following the model validation, the true scores were converted to the maximum fluorescence intensity (F_{max}) to generate intensities in water Raman unit (R.U.) for each component,[27] and the excitation and emission wavelengths of individual components (**Figure S2**) were queried through the *OpenFluor* database[28] for comparisons with published spectra[29, 30] using a Tucker's congruence coefficient criterion of 0.95.[31-34] Three PARAFAC components were operationally defined as component 1 (C1), component 2 (C2), and component 3 (C3), respectively. C2 had the highest number of matches in the *OpenFluor* database at the time of access (September 2022), followed by C1 and C3, respectively. C1 is a terrestrial humic-like component (fulvic acid-type; high aromaticity, high molecular weight),[14, 35] C2 is another terrestrial humic-like component (humic acid-type; high aromaticity, high molecular weight),[14, 35] and C3 is a microbial humic-like component (aliphatic, low molecular weight).[14, 35] C1, C2, and C3 all matched with corresponding components in PARAFAC models

previously developed for New York surface waters.[11, 36] Measured physicochemical and optical properties of stream water samples (mean±standard deviation for each site) are summarized in **Table S4**.

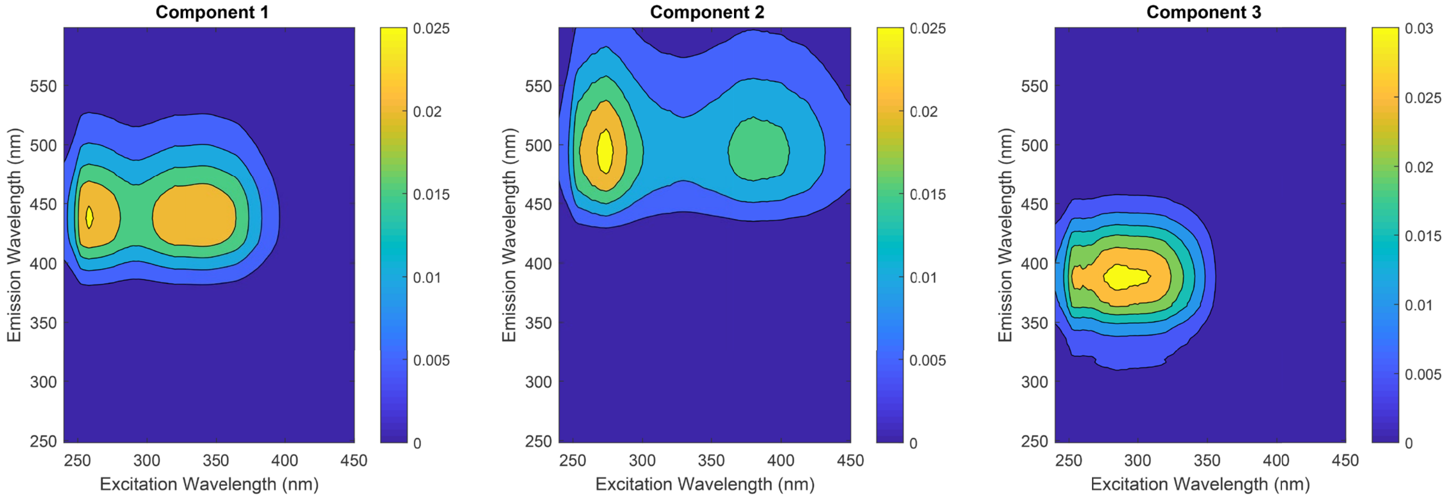

Figure S1. Contour plots of 3 fluorescent components validated for the 3-component PARAFAC model.

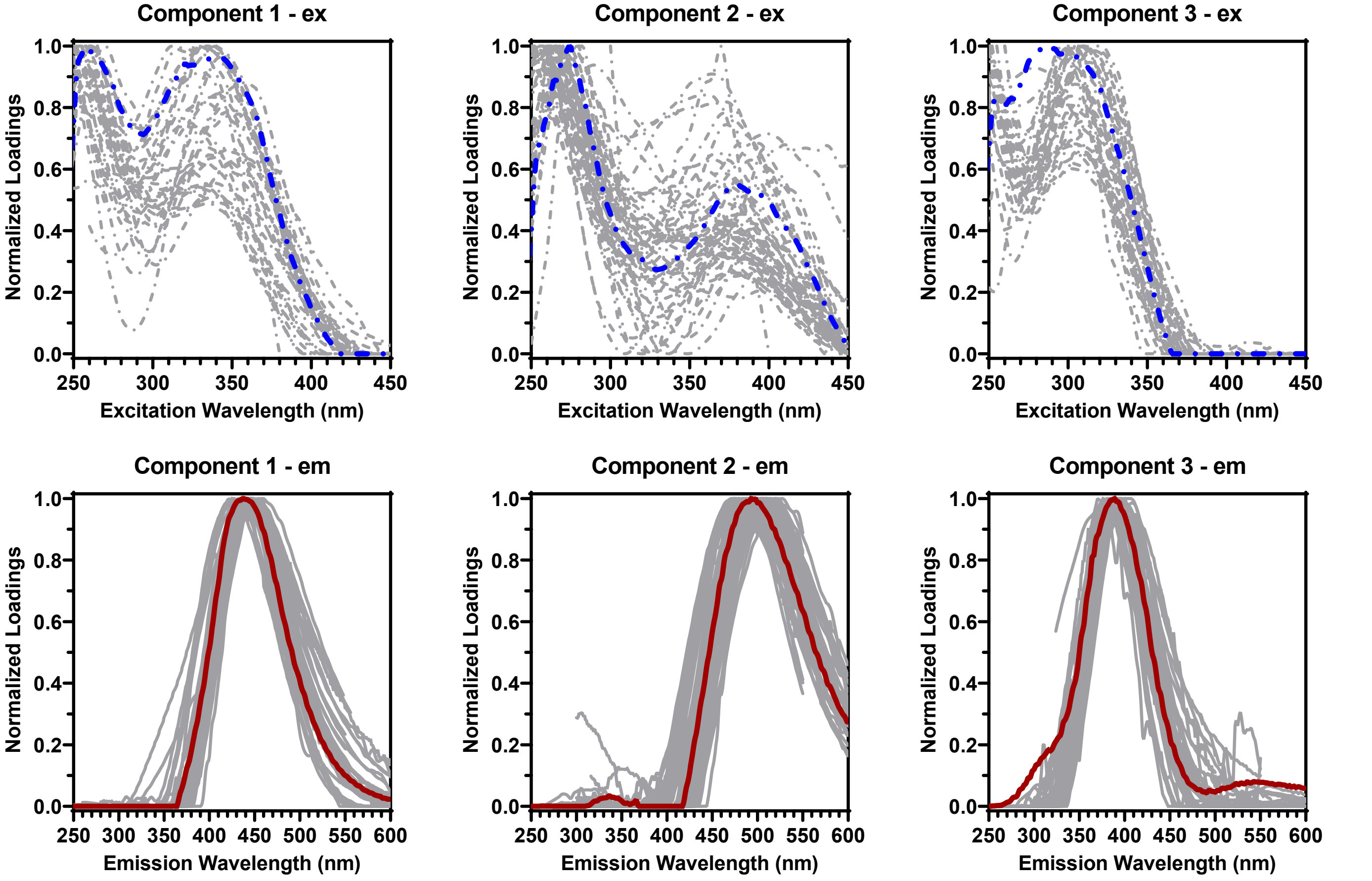

Figure S2. Spectral comparisons of the 3-component PARAFAC model with those published in the *OpenFluor* database.

Table S4. Physicochemical and optical properties of stream water samples

Site Name	pH	Specific Conductance (μS/cm)	Cl^- (mg/L)	NO_3^- (mg/L)	a_{254} (m^{-1})	*E2*:*E3*	FI	HIX	$\beta:\alpha$	C1 (R.U.)	C2 (R.U.)	C3 (R.U.)
Butternut Creek	7.87±0.37	794±326	107±43	2.1±0.4	15.6±9.2	6.91±0.78	1.70±0.06	0.90±0.05	0.58±0.02	1.49±0.87	1.15±0.79	0.87±0.43
Chenango River	7.67±0.34	591±255	28±2	4.2±0.5	19.0±6.2	6.42±0.50	1.70±0.03	0.93±0.03	0.57±0.01	1.82±0.58	1.30±0.44	0.99±0.21
Chittenango Creek	7.68±0.36	462±132	30±3	3.2±0.8	16.7±2.0	6.67±0.56	1.66±0.03	0.94±0.01	0.57±0.01	1.61±0.20	1.26±0.12	0.89±0.11
Conesus Creek	7.72±0.33	351±96	81±7	4.9±1.0	17.4±1.5	8.52±1.77	1.74±0.09	0.89±0.01	0.66±0.02	1.32±0.25	0.91±0.16	0.92±0.12
Cowaselon Creek	7.72±0.34	304±95	35±11	2.9±1.6	12.7±7.9	7.08±1.14	1.79±0.07	0.95±0.05	0.59±0.03	1.42±0.83	1.00±0.66	0.74±0.37
Flint Creek	7.82±0.32	284±106	46±5	5.1±3.5	42.2±1.9	6.42±0.16	1.65±0.04	0.96±0.01	0.54±0.02	4.43±0.33	3.59±0.44	2.16±0.08
Ganargua Creek	7.72±0.16	359±236	150±32	12.4±6.2	31.9±11.3	6.12±0.64	1.70±0.08	0.94±0.02	0.58±0.06	3.51±1.20	2.74±1.15	1.88±0.45
Harbor Brook	7.84±0.15	455±202	246±138	4.6±2.6	7.2±5.0	8.16±0.60	1.80±0.07	0.90±0.02	0.66±0.02	0.93±0.62	0.67±0.52	0.72±0.48
Honeoye Creek	7.84±0.15	539±80	64±12	1.2±0.7	35.5±15.1	6.67±0.49	1.66±0.06	0.95±0.01	0.57±0.02	3.66±1.72	2.69±1.12	1.97±0.87
Ninemile Creek at Lakeland	7.83±0.12	517±79	350±123	4.7±1.7	20.2±14.6	7.14±1.05	1.72±0.05	0.91±0.04	0.60±0.03	2.06±1.39	1.60±1.34	1.23±0.71
Ninemile Creek at Marietta	7.83±0.13	489±28	54±8	4.3±3.0	11.8±1.2	8.11±0.68	1.73±0.05	0.87±0.07	0.65±0.01	0.85±0.14	0.61±0.10	0.61±0.07
Oatka Creek	7.80±0.11	506±49	73±2	11.9±1.4	18.4±5.4	7.02±0.47	1.70±0.04	0.95±0.01	0.60±0.02	2.05±0.62	1.48±0.42	1.15±0.28
Oneida Creek	7.89±0.11	506±49	41±4	5.9±0.9	17.6±3.2	6.37±0.26	1.73±0.04	0.95±0.02	0.61±0.02	1.90±0.42	1.34±0.23	1.07±0.23
Oriskany Creek	7.92±0.09	698±349	48±4	7.8±1.5	14.4±1.2	6.20±0.26	1.73±0.06	0.96±0.03	0.58±0.01	1.55±0.19	1.11±0.20	0.83±0.14
Otselic River	7.85±0.15	1284±1104	15±2	3.5±0.6	14.0±2.4	6.47±0.47	1.70±0.02	0.94±0.05	0.55±0.01	1.29±0.24	0.90±0.18	0.65±0.10
Salmon Creek	7.89±0.16	1322±1074	42±4	14.8±6.4	14.3±2.6	7.23±0.85	1.78±0.09	0.96±0.03	0.63±0.03	1.66±0.21	1.11±0.24	0.91±0.14
Sauquoit Creek	7.91±0.18	1418±987	86±6	6.6±1.0	11.0±2.7	6.20±1.22	1.71±0.04	0.93±0.01	0.62±0.02	1.24±0.23	0.88±0.21	0.81±0.15
Scriba Creek	7.96±0.21	1254±1082	10±2	1.2±0.7	79.0±4.2	5.24±0.19	1.58±0.03	0.96±0.01	0.44±0.02	5.80±0.31	4.91±0.43	2.20±0.24
Sixmile Creek	8.07±0.06	660±169	45±8	1.5±0.6	18.7±10.5	6.25±1.26	1.70±0.06	0.93±0.03	0.58±0.03	1.56±0.49	1.16±0.40	0.89±0.20
Skaneateles Creek	8.07±0.06	626±167	38±11	2.9±0.8	10.2±4.7	7.44±2.30	1.71±0.06	0.87±0.09	0.60±0.03	0.93±0.55	0.69±0.41	0.57±0.28

S4. Screening and quantification of OMPs in POCIS and grab samples by SPE-LC-HRMS

Grab samples were extracted by mixed-mode solid-phase extraction (SPE) and analyzed by liquid chromatography-HRMS (LC-HRMS) as described in our previous work.[11] Within 24 h of collection, duplicate samples (500 mL each) were adjusted to pH 6.8±0.1 with ammonium acetate and formic acid, spiked with a mixture of isotope-labeled internal standards (200 ng/L each; **Table S5**), and filtered through precombusted 0.7-µm glass fiber filters. Samples were then passed through preconditioned dual SPE cartridges containing 200 mg of Sepra ZT (Phenomenex), 100 mg of Sepra ZT-SAX (Phenomenex), 100 mg of Sepra ZT-SCX (Phenomenex), and 150 mg of ISOLUTE ENV+ (Biotage) sorbents as the top layer and 200 mg of Enviro-Clean graphitized nonporous carbon (United Chemical Technologies) as the bottom layer. SPE cartridges were dried under ultrahigh-purity N_2 following extraction, reconnected inversely (with graphitized nonporous carbon as the top layer), and eluted sequentially with 6 mL of methanol/ethyl acetate (50:50 v/v; amended with 2% ammonia), 3 mL of methanol/ethyl acetate (50:50 v/v; amended with 1.7% formic acid), and 2 mL of methanol.[11] Sample extracts were concentrated to 0.1 mL under ultrahigh-purity N_2, reconstituted with methanol:water (10:90 v/v) to a final volume of 1 mL, and transferred to amber autosampler vials for analysis by a Dionex UltiMate 3000 high-performance liquid chromatograph interfaced with a Thermo Scientific LTQ XL hybrid ion trap-Orbitrap high-resolution mass spectrometer (**Table S6**). For chromatographic separation, 20 µL of SPE extracts were injected onto a Hypersil GOLD C18 analytical column (100 × 2.1 mm, 1.9 µm; preceded with a 10 × 2.1 mm guard cartridge) running water and methanol (both acidified with 0.1% v/v formic acid) as the mobile phases at a flow rate of 200 µL/min and a column temperature of 35 °C.[11] For mass spectrometric analysis, full scan mass spectra were acquired from 100 to 1000 Da with a mass resolution of 60,000 at *m/z* 400 using both positive and negative electrospray ionization in separate runs. Full scan triggered data-dependent tandem mass (dd-MS2) spectra were also acquired (upon reinjection of sample extracts) with a mass resolution of 7,500 at *m/z* 400 using higher energy collision-induced dissociation across stepped collision energies (i.e., 30%, 45%, and 60%) while maintaining a full scan mass resolution of 30,000 at *m/z* 400.[11] Mass accuracy and chromatographic reproducibility were monitored with reference to isotope-labeled internal standards.

**Table S5.** LC-HRMS method for isotope-labeled internal standards						
Compound Name	CAS	Molecular Formula	Adduct	Exact Mass (*m/z*)	RT (min)	%RSD
3,4-Methylenedioxymethamphetamine-d_5	136765-43-0	$C_{11}H_{10}D_5NO_2$	[M+H]+	199.1489	7.97	2.5
Abacavir-d_4	1217731-56-0	$C_{14}H_{14}D_4N_6O$	[M+H]+	291.1866	8.66	4.3
Acetaminophen-d_3	60902-28-5	$C_8H_6D_3NO_2$	[M+H]+	155.0894	3.56	3.2
Amantadine-d_{15}	33830-10-3	$C_{10}H_2D_{15}N$	[M+H]+	167.2375	9.61	7.4
Amphetamine-d_{10}	169565-17-7	$C_9H_3D_{10}N$	[M+H]+	146.1748	7.09	7.4
Atenolol-d_7	1202864-50-3	$C_{14}H_{15}D_7N_2O_3$	[M+H]+	274.2143	3.12	8.0
Buprenorphine-d_4	136781-89-0	$C_{29}H_{37}D_4NO_4$	[M+H]+	472.3359	13.15	3.8
Bupropion-d_9	1189725-26-5	$C_{13}H_9D_9ClNO$	[M+H]+	249.1715	11.78	2.5
Caffeine-d_9	72238-85-8	$C_8HD_9N_4O_2$	[M+H]+	204.1441	8.37	2.4
Carbamazepine-d_{10}	132183-78-9	$C_{15}H_2D_{10}N_2O$	[M+H]+	247.1650	14.54	3.4
Cetirizine-d_8	2070015-04-0	$C_{21}H_{17}D_8ClN_2O_3$	[M+H]+	397.2129	16.40	2.2
Cimetidine-d_3	1185237-29-9	$C_{10}H_{13}D_3N_6S$	[M+H]+	256.1418	3.28	3.2
Clarithromycin-d_3	NA	$C_{38}H_{66}D_3NO_{13}$	[M+H]+	751.5030	16.82	4.2
Codeine-d_3	70420-71-2	$C_{18}H_{18}D_3NO_3$	[M+H]+	303.1783	4.67	2.7
Cotinine-d_3	110952-70-0	$C_{10}H_9D_3N_2O$	[M+H]+	180.1211	1.84	7.4
Diclofenac-d_4	153466-65-0	$C_{14}H_7D_4Cl_2NO_2$	[M-H]-	298.0345	19.89	8.7
Diphenhydramine-d_3	170082-18-5	$C_{17}H_{18}D_3NO$	[M+H]+	259.1884	13.21	14.6
Dopamine-d_4	203633-19-6	$C_8H_7D_4NO_2$	[M+H]+	158.1114	1.69	2.4
Ephedrine-d_3	285979-73-9	$C_{10}H_{12}D_3NO$	[M+H]+	169.1414	5.02	7.4
Fentanyl-d_5	118357-29-2	$C_{22}H_{23}D_5N_2O$	[M+H]+	342.2588	12.36	2.2
Fexofenadine-d_6	548783-71-7	$C_{32}H_{33}D_6NO_4$	[M+H]+	508.3329	15.26	2.6
Fluconazole-$^{13}C_3$	NA	$C_{10}{}^{13}C_3H_{12}F_2N_6O$	[M+H]+	310.1214	11.00	2.3
Fluoxetine-d_5	1173020-43-3	$C_{17}H_{13}D_5F_3NO$	[M+H]+	315.1727	16.31	2.1
Gabapentin-d_{10}	1126623-20-8	$C_9H_7D_{10}NO_2$	[M+H]+	182.1960	7.14	11.7
Gemfibrozil-d_6	1184986-45-5	$C_{15}H_{16}D_6O_3$	[M+H]+	257.2018	21.86	4.6
Hydrocodone-d_3	136765-36-1	$C_{18}H_{18}D_3NO_3$	[M+H]+	303.1783	6.96	6.1
Hydromorphone-d_3	136765-37-2	$C_{17}H_{16}D_3NO_3$	[M+H]+	289.1626	2.42	13.6
Lamotrigine-^{13}C,$^{15}N_4$	NA	$^{13}C_1C_8H_7Cl_2N_1{}^{15}N_4$	[M+H]+	261.0066	10.87	2.2
Levetiracetam-d_6	1435933-72-4	$C_8H_8D_6N_2O_2$	[M+H]+	177.1505	5.94	11.6
Lidocaine-d_{10}	851528-09-1	$C_{14}H_{12}D_{10}N_2O$	[M+H]+	245.2433	8.93	5.9
Losartan-d_4	1030937-27-9	$C_{22}H_{19}D_4ClN_6O$	[M+H]+	427.1946	16.20	1.1
Meperidine-d_4	53484-73-4	$C_{15}H_{17}D_4NO_2$	[M+H]+	252.1896	11.21	2.4
Metaxalone-d_3	1192812-66-0	$C_{12}H_{12}D_3NO_3$	[M+H]+	225.1313	15.03	15.3
Metformin-d_6	1185166-01-1	$C_4H_5D_6N_5$	[M+H]+	136.1464	1.60	4.9
Methadone-d_3	60263-63-0	$C_{21}H_{24}D_3NO$	[M+H]+	313.2354	15.12	3.5
Methamphetamine-d_8	136765-40-7	$C_{10}H_7D_8N$	[M+H]+	158.1779	7.77	5.8
Methocarbamol-d_3	1346600-86-9	$C_{11}H_{12}D_3NO_5$	[M+H]+	245.1211	11.19	1.6
Metoprolol-d_7	1219798-61-4	$C_{15}H_8D_7NO_3$	[M+H]+	275.2347	10.62	2.4
Morphine-d_3	67293-88-3	$C_{17}H_{16}D_3NO_3$	[M+H]+	289.1626	1.99	3.2
Naproxen-d_3	958293-77-1	$C_{14}H_{11}D_3O_3$	[M+H]+	234.1204	17.23	1.5
Nicotine-d_4	350818-69-8	$C_{10}H_{10}D_4N_2$	[M+H]+	167.1481	1.66	17.1
Oxycodone-d_3	160227-46-3	$C_{18}H_{18}D_3NO_4$	[M+H]+	319.1732	6.12	6.8

**Table S5.** LC-HRMS method for isotope-labeled internal standards (continued)						
Compound Name	CAS	Molecular Formula	Adduct	Exact Mass (*m/z*)	RT (min)	%RSD
Oxymorphone-d_3	145225-03-2	$C_{17}H_{16}D_3NO_4$	[M+H]+	305.1575	2.12	4.7
Phendimetrazine-d_5	NA	$C_{12}H_{12}D_5NO$	[M+H]+	197.1697	6.90	11.7
Phenytoin-d_{10}	65854-97-9	$C_{15}H_2D_{10}N_2O_2$	[M+H]+	263.1599	14.17	4.9
Phentermine-d_5	1330236-21-9	$C_{10}H_{10}D_5N$	[M+H]+	155.1591	9.24	5.4
Pregabalin-d_6	NA	$C_8H_{11}D_6NO_2$	[M+H]+	166.1709	7.01	5.3
Protriptyline-d_3	1435934-21-6	$C_{19}H_{18}D_3N$	[M+H]+	267.1935	15.11	3.2
Sulfamethoxazole-d_4	1020719-86-1	$C_{10}H_7D_4N_3O_3S$	[M+H]+	258.0845	9.68	4.1
Theophylline-d_6	117490-39-8	$C_7H_2D_6N_4O_2$	[M+H]+	187.1097	6.53	6.5
Tramadol-$^{13}C,d_3$	NA	$^{13}C_1C_{15}H_{22}D_3NO_2$	[M+H]+	268.2180	10.31	3.5
Trimethoprim-d_9	1189460-62-5	$C_{14}H_9D_9N_4O_3$	[M+H]+	300.2017	8.36	3.6
Valsartan-d_3	1331908-02-1	$C_{24}H_{26}D_3N_5O_3$	[M+H]+	439.2532	18.03	5.6
Venlafaxine-d_6	1062606-12-5	$C_{17}H_{21}D_6NO_2$	[M+H]+	284.2491	12.51	2.9
2-Ethylidene-1,5-dimethyl-3,3-diphenylpyrrolidine-d_3	136765-23-6	$C_{20}H_{21}D_3N$	[M]+	281.2092	13.08	2.2
3-Hydroxy Cotinine-d_3	159956-78-2	$C_{10}H_9D_3N_2O_2$	[M+H]+	196.1160	1.63	4.3
Benzoylecgonine-d_3	115732-68-8	$C_{16}H_{16}D_3NO_4$	[M+H]+	293.1575	9.78	5.9
Ecgonine Methyl Ester-d_3	136765-34-9	$C_{10}H_{14}D_3NO_3$	[M+H]+	203.1470	1.63	11.7
Hydroxybupropion-d_6	1184984-06-2	$C_{13}H_{12}D_6ClNO_2$	[M+H]+	262.1475	11.22	1.2
Norcodeine-d_3	NA	$C_{17}H_{16}D_3NO_3$	[M+H]+	289.1626	4.98	5.1
Norfentanyl-d_5	1211527-23-9	$C_{14}H_{15}D_5N_2O$	[M+H]+	238.1962	10.05	3.6
Norhydrocodone-d_3	NA	$C_{17}H_{16}D_3NO_3$	[M+H]+	289.1626	7.24	5.2
Noroxycodone-d_3	1426174-79-9	$C_{17}H_{16}D_3NO_4$	[M+H]+	305.1575	15.73	2.3
Ritalinic Acid-d_{10}	NA	$C_{13}H_7D_{10}NO_2$	[M+H]+	230.1960	9.76	2.9
(4-Chloro-2-methylphenoxy-d_3)acetic Acid (MCPA-d_3)	352431-14-2	$C_9H_6D_3ClO_3$	[M-H]-	202.0356	16.41	11.8
(2,4-Dichlorophenoxy-d_3)acetic Acid (2,4-D-d_3)	202480-67-9	$C_8H_3D_3Cl_2O_3$	[M-H]-	221.9810	16.06	11.0
Acetamiprid-d_3	1353869-35-8	$C_{10}H_8D_3ClN_4$	[M+H]+	226.0933	10.94	3.4
Atrazine-d_5	163165-75-1	$C_8H_9D_5ClN_5$	[M+H]+	221.1324	15.40	5.4
Carbaryl-d_7	362049-56-7	$C_{12}H_4D_7NO_2$	[M+H]+	209.1302	14.35	5.4
Carbendazim-d_4	291765-95-2	$C_9H_5D_4N_3O_2$	[M+H]+	196.1019	6.55	5.2
Clofibric Acid-d_4	1184991-14-7	$C_{10}H_7D_4ClO_3$	[M-H]-	217.0575	17.04	11.5
Clothianidin-d_3	1262776-24-8	$C_6H_5D_3ClN_5O_2S$	[M+H]+	253.0348	10.04	4.3
Diuron-d_6	1007536-67-5	$C_9H_4D_6Cl_2N_2O$	[M+H]+	239.0620	15.88	3.3
Imidacloprid-d_4	1015855-75-0	$C_9H_6D_4ClN_5O_2$	[M-H]-	258.0701	9.93	9.4
Mecoprop-d_3	352431-15-3	$C_{10}H_8D_3ClO_3$	[M-H]-	216.0512	17.96	9.3
Metalaxyl-d_3	NA	$C_{15}H_{18}D_3NO_4$	[M+H]+	283.1732	15.73	4.7
Metolachlor-d_6	1219803-97-0	$C_{15}H_{16}D_6ClNO_2$	[M+H]+	290.1788	19.07	2.0
Prometon-d_3	1219803-43-6	$C_{10}H_{16}D_3N_5O$	[M+H]+	229.1851	13.38	2.7
Simazine-d_{10}	220621-39-6	$C_7H_2D_{10}ClN_5$	[M+H]+	212.1482	13.47	3.9
Thiamethoxam-d_3	1294048-82-0	$C_8H_7D_3ClN_5O_3S$	[M+H]+	295.0454	8.46	8.1
Benzophenone-d_{10}	22583-75-1	$C_{13}D_{10}O$	[M+H]+	193.1432	17.24	4.2
Benzothiazole-d_4	194423-51-3	C_7HD_4NS	[M+H]+	140.0467	12.87	6.1
Benzotriazole-d_4	1185072-03-0	$C_6HD_4N_3$	[M+H]+	124.0807	9.04	1.8
Benzyl Butyl Phthalate-d_4	93951-88-3	$C_{19}H_{16}D_4O_4$	[M+H]+	317.1685	21.24	7.0
Caprolactam-d_{10}	169297-53-4	$C_6HD_{10}NO$	[M+H]+	124.1541	6.55	4.9
N,N-Diethyl-3-methyl-d_3-benzamide-2,4,5,6-d_4 (DEET-d_7)	1219799-37-7	$C_{12}H_{10}D_7NO$	[M+H]+	199.1822	15.72	3.4

**Table S5.** LC-HRMS method for isotope-labeled internal standards (continued)						
Compound Name	CAS	Molecular Formula	Adduct	Exact Mass (*m/z*)	RT (min)	%RSD
Diethyl Phthalate-d_{14}	99873-99-1	$C_{12}D_{14}O_4$	[M+H]+	237.1844	15.87	4.5
Dimethyl Phthalate-d_6	85448-30-2	$C_{10}H_4D_6O_4$	[M+H]+	201.1029	13.10	7.7
Melamine-$^{13}C_3$,$^{15}N_3$	1246816-14-7	$^{13}C_3H_6N_3{}^{15}N_3$	[M+H]+	133.0738	1.39	5.2
Oxybenzone-d_5	1219798-54-5	$C_{14}H_7D_5O_3$	[M+H]+	234.1173	19.00	5.3
Propylparaben-d_4	1219802-67-1	$C_{10}H_8D4O_3$	[M+H]+	185.1110	15.76	5.8
Sucralose-d_6	1459161-55-7	$C_{12}H_{13}D_6Cl_3O_8$	[M+FA-H]-	447.0504	9.17	13.5
Triclosan-d_3	1020719-98-5	$C_{12}H_4D_3Cl_3O_2$	[M-H]-	289.9627	21.87	14.1
Tris(2-chloroethyl) Phosphate-d_{12}	1276500-47-0	$C_6D_{12}Cl_3O_4P$	[M+H]+	297.0365	13.93	5.1

"RT" = retention time; "NA" = not available; "%RSD" = the percent relative standard deviation of the isotope-labeled internal standard peak areas in calibration standards analyzed over the project period.

**Table S6.** LC-HRMS instrument settings		
Dionex UltiMate 3000 High-Performance Liquid Chromatograph		
Time (min)	Mobile Phase A (H_2O + 0.1% FA)	Mobile Phase B (MeOH + 0.1% FA)
0	90%	10%
1	90%	10%
8	50%	50%
21	5%	95%
29	5%	95%
29.5	90%	10%
36	90%	10%
Thermo Scientific LTQ XL Hybrid Ion Trap-Orbitrap High-Resolution Mass Spectrometer		
	Positive ESI	Negative ESI
	Ion Source	
Source Voltage (V)	3,900 (+)	3,500 (-)
Capillary Temperature (°C)	270.00	310.00
Capillary Voltage (V)	38.00 (+)	35.00 (-)
Tube Lense (V)	95.00 (+)	85.00 (-)
Sheath Gas Flow (arb)	25.00	25.00
Auxiliary Gas Flow (arb)	5.00	3.00
Sweep Gas Flow (arb)	0.00	0.00
	ITMS (Linear Ion Trap MS)	
Full MS Scan Range (*m/z*)	100.0-1000.0	100.0-1000.0
Full MS AGC Target	1,000,000	1,000,000
Full MS Maximum Injection Time (ms)	100	100
dd-MS2 Isolation Width	2.0	2.0
HCD dd-MS2 AGC Target	100,000	100,000
HCD dd-MS2 Maximum Injection Time (ms)	250.00	250.00
HCD dd-MS2 Normalized Collision Energy (%)	30, 45, 60	30, 45, 60
dd-MS2 Dynamic Exclusion (s)	4.0	4.0
	FTMS (Orbitrap MS)	
Full MS Scan Range (*m/z*)	100.0-1000.0	100.0-1000.0
Full MS Scan Resolution (at *m/z* 400)	60,000	60,000
dd-MS2 Scan Resolution (at *m/z* 400)	7,500	7,500

Table S7. *TraceFinder 4.1* settings

Peak Detection	
Threshold Override	5 E5
S/N Ratio Threshold	100
Mass Tolerance (ppm)	5
Detection Algorithm	ICIS
Detection Method	Highest peak
Smoothing	1
Area Noise Factor	100
Peak Noise Factor	50
Baseline Window	5
Min Peak Height (S/N)	5
Noise Method	Incos
Min Peak Width	5
Multiplet Resolution	10
Area Tail Extension	5
Area Scan Window	0
Isotopic Pattern	
Fit Threshold (%)	50
Allowed Mass Deviation (ppm)	10
Allowed Intensity Deviation (%)	10

Table S8. *Compound Discoverer 3.3* settings for suspect/nontarget screening

Select Spectra	Align Retention Times	Search mzVault
1. General Settings:<br>-Precursor Selection: Use MS(n-1) Precursor;<br>2. Spectrum Properties Filters:<br>-Lower RT Limit: 0.5;<br>-Upper RT Limit: 0;<br>-First Scan: 0;<br>-Last Scan: 0;<br>-Lowest Charge State: 0;<br>-Highest Charge State: 0;<br>-Min. Precursor Mass: 100 Da;<br>-Max. Precursor Mass: 1000 Da;<br>-Total Intensity Threshold: 0;<br>-Minimum Peak Count: 1;<br>3. Scan Event Filters:<br>-Mass Analyzer: (Not specified);<br>-MS Order: Any;<br>-Activation Type: Not specified;<br>-Min. Collision Energy: 0,<br>-Max. Collision Energy: 1000;<br>-Scan Type: Any;<br>-Polarity Mode: (Not specified);<br>4. Peak Filters:<br>-S/N Threshold (FT-only): 1.5;<br>5. Replacements for Unrecognized Properties:<br>-Unrecognized Charge Replacements: 1;<br>-Unrecognized Mass Analyzer Replacements: ITMS;<br>-Unrecognized MS Order Replacements: MS2;<br>-Unrecognized Activation Type Replacements: CID;<br>-Unrecognized Polarity Replacements: +;<br>-Unrecognized MS Resolution @ 200 Replacements: 60000;<br>-Unrecognized MSn Resolution@ 200 Replacements: 7500.	1. General Settings:<br>-Alignment Model: Adaptive Curve;<br>-Mass Tolerance: 5.0 ppm;<br>-Maximum Shift (min): 2;<br>**Detect Compounds**<br>1. General Settings:<br>-Mass Tolerance [ppm]: 5.0 ppm;<br>-Intensity Tolerance (%): 30; -S/N Threshold: 3;<br>-Min. Peak Intensity: 1000;<br>-Ions: [M+H]+1; [M-H]-1; [M+Na]+1;<br>-Min Element Counts: C4 H4 O;<br>-Max Element Counts: C50 H100 Cl N3 O40 P S;<br>**Group Compounds**<br>1. Compound Consolidation: -Mass Tolerance [ppm]: 5.0 ppm;<br>-RT Tolerance [min]: 0.5;<br>-Preferred Ions: [M+H]+1; [M-H]-1; [M+Na]+1; [M+FA-H]-1; [M+Cl]-1 [M]+1; [M]-1;<br>**Merge Features**<br>1. Peak Consolidation:<br>-Mass Tolerance [ppm]: 5.0 ppm;<br>-RT Tolerance [min]: 0.5;<br>**Apply mzLogic**<br>1. Search Settings:<br>-Max. #Compounds: 10;<br>-Max. #mzCloud Similarity Results to consider per compound: 10;<br>-Match Factor Threshold: 10;	1. Search Settings:<br>-mzVault Library: \ mzVault September 2020.db; \MassBankNA.db; \Eawag.db; \Eawag_Additional_Specs.db;<br>-Compound Classes: All;<br>-Match Ion Activation Type: True;<br>-Match Ion Activation Energy: Match with Tolerance;<br>-Ion Activation Energy Tolerance: 20;<br>-Match Ionization Method: True;<br>-Apply Intensity Threshold: True;<br>-Precursor Mass Tolerance: 5.0 ppm;<br>-Match Analyzer Type; True;<br>-Search Algorithm: HighChem HighRes;<br>-Match Factor Threshold: 70;<br>-RT Tolerance [min]: 2;<br>-Use Retention Time: False;<br>**Search Mass Lists**<br>1. Search Settings:<br>-Mass Lists: Shiru_SuspectDatabase or NORMANSusDat;<br>-Use Retention Time: True;<br>-RT Tolerance [min]: 0.5;<br>-Mass Tolerance: 5.0 ppm;<br>**Search mzCloud**<br>1. Search Settings:<br>-Compound Classes: All;<br>-Match Ion Activation Type: True;<br>-Match Ion Activation Energy: Match with Tolerance;<br>-Ion Activation Energy Tolerance: 20;<br>-Match Ionization Method: True;<br>-Apply Intensity Threshold: True;<br>-Identity Search: Cosine;<br>-Similarity Search: Similarity Forward;<br>-Match Factor Threshold: 70;<br>**Search ChemSpider**<br>1. Search Settings:<br>-Database(s): EPA DSSTox; EPA Toxcast;<br>-Search Mode: By Formula or Mass;<br>-Mass Tolerance: 5.0 ppm;<br>-Max. # of results per compound: 10;<br>-Max. # of Predicted Compositions to be searched: 3;

<table>
<tr><th colspan="3">Table S8. Compound Discoverer 3.3 settings for suspect/nontarget screening (continued)</th></tr>
<tr><th>Predict Compositions</th><th>Fill Gaps</th><th>Assign Compound Annotations</th></tr>
<tr><td rowspan="7">1. General Settings:
-Mass Tolerance: 5.0 ppm;
-Min. Element Counts: C4 H4 O;
-Max Element Counts: C50 H100 Cl N3 O40 P S;
-Min. RDBE: 0;
-Max. RDBE: 40;
-Min. H/C: 0.3;
-Max. H/C: 2.3;
-Max. # Candidates: 10;
2. Pattern Matching
-Intensity Tolerance [%]: 30;
-Intensity Threshold: 0.1;
-S/N Threshold: 3;
-Use Dynamic Recalibration: True;
3. Fragments Matching
-Use Fragments Matching: True;
-Mass Tolerance; 10 ppm;
-S/N Threshold: 3;</td><td>1. General Settings:
-Mass Tolerance: 5.0 ppm;
-S/N Threshold: 3;</td><td rowspan="3">1. General Settings:
-Mass Tolerance [ppm]: 5.0 ppm;
2. Data Sources:
-Data Source #1: mzCloud Search;
-Data Source #2: mzVault Search;
-Data Source #3: Predicted Composition;</td></tr>
<tr><th>Normalize Areas</th></tr>
<tr><td>1. QC-based Area Correction:
-Min. QC Coverage [%]: 50;
-Max. QC Area RSD [%]: 30;
2. Area Normalization:
-Normalization Type: Constant Sum;
-Exclude Blanks: True</td></tr>
<tr><th>Mark Background Compounds</th><th>Scripting Node</th></tr>
<tr><td>1. General Settings:
-Max. Sample/Blank: 5;
-Max. Blank/Sample: 0;
-Hide Background: True;</td><td rowspan="3">1. Executable and Parameters:
-Path to Executable: C:\Program Files\R\R-4.1.0\bin\Rscript.exe;
-Command Line Arguments: C:\Rscripts\OCRatio\OCRatio.R %NODEARGS%;
-Requested Tables and Columns: Compounds: Formula;
-Use R-Friendly Columns: True;
-Archive Datafiles: False;</td></tr>
<tr><th>Differential Analysis</th></tr>
<tr><td>1. General Settings:
-Log Transformation Values: True;</td></tr>
</table>

Overall, suspect and nontarget screening prioritized 335 compounds (**Table S9**), among which 133 were confirmed by verifying their chromatographic retention times and dd-MS2 spectra against those of the respective reference standards. For each confirmed suspect or nontarget compound, the absolute SPE recovery, ion suppression or enhancement, matrix factor, and limits of quantification (LOQs) were determined as detailed in our previous work[11] and summarized in **Table S10**. Target analysis was performed retrospectively to quantify confirmed OMPs in POCIS and grab samples. Briefly, twelve calibration standards (triplicate; each 500 mL) prepared in HPLC grade water containing target OMPs (typically 0-3000 ng/L) and ILIS (200 ng/L) were extracted and analyzed following the SPE-LC-HRMS method described in the *Main Text*. Calibration curves were constructed in *TraceFinder* by the non-weighted linear least squares regression algorithm (average R^2 = 0.9944±0.0065 for 133 OMPs with absolute SPE recoveries). Quantification was performed using the peak area ratios of target OMPs and structurally identical ILIS or ILIS with the closest chromatographic retention times. On average, the absolute spike recovery of ILIS was 83±20%. Over the project period, method reproducibility was monitored based on the relative standard deviations (ranging from 1.1% to 17.1% with a mean of 5.6±3.6%) of the ILIS peak areas in calibration standards (**Table S5**). None of the target OMPs was detected in the field blanks. Nondetects were assigned a concentration of 0 ng/L, while concentrations below the LOQs were used as is if peaks contained at least five consecutive full scan data points and one diagnostic MS2 fragment.

**Table S9.** LC-HRMS method for 335 suspect and nontarget OMPs						
Compound Name	CAS	Molecular Formula	Adduct	Exact Mass (*m/z*)	Diagnostic Fragment (*m/z*)	RT (min)
17β-Estradiol	50-28-2	$C_{18}H_{24}O_2$	[M+H]+	273.1849	255.1745	17.46
3,4-Methylenedioxymethamphetamine	42542-10-9	$C_{11}H_{15}NO_2$	[M+H]+	194.1176	163.0760	8.00
3,4-Methylenedioxy-N-ethylamphetamine	82801-81-8	$C_{12}H_{17}NO_2$	[M+H]+	208.1332	163.0746	8.93
Abacavir	136470-78-5	$C_{14}H_{18}N_6O$	[M+H]+	287.1615	191.1030	8.65
Acamprosate	77337-76-9	$C_5H_{11}NO_4S$	[M+H]+	182.0482	140.0371	1.62
Acebutolol	37517-30-9	$C_{18}H_{28}N_2O_4$	[M+H]+	337.2122	116.1064	10.45
Acetaminophen	103-90-2	$C_8H_9NO_2$	[M+H]+	152.0706	126.0100	3.65
Albendazole	54965-21-8	$C_{12}H_{15}N_3O_2S$	[M+H]+	266.0958	234.0693	15.01
Albuterol	18559-94-9	$C_{13}H_{21}NO_3$	[M+H]+	240.1594	148.0749	3.18
Aliskiren	173334-57-1	$C_{30}H_{53}N_3O_6$	[M+H]+	552.4007	436.3054	16.44
Amantadine	768-94-5	$C_{10}H_{17}N$	[M+H]+	152.1434	135.1162	9.78
Amitriptyline	50-48-6	$C_{20}H_{23}N$	[M+H]+	278.1903	233.1315	15.40
Amphetamine	300-62-9	$C_9H_{13}N$	[M+H]+	136.1121	119.0859	7.32
Androstanolone (Stanolone/5α-Dihydrotestosterone)	521-18-6	$C_{19}H_{30}O_2$	[M+H]+	291.2319	273.2212	19.71
Androstenedione	63-05-8	$C_{19}H_{26}O_2$	[M+H]+	287.2006	269.1892	17.38
Atenolol	29122-68-7	$C_{14}H_{22}N_2O_3$	[M+H]+	267.1703	190.0851	3.20
Atomoxetine	83015-26-3	$C_{17}H_{21}NO$	[M+H]+	256.1696	147.0796	14.65
Atropine	51-55-8	$C_{17}H_{23}NO_3$	[M+H]+	290.1751	124.1120	9.38
Azelaic Acid	123-99-9	$C_9H_{16}O_4$	[M+H]+	189.1121	102.0546	13.17
Azithromycin	83905-01-5	$C_{38}H_{72}N_2O_{12}$	[M+H]+	749.5158	158.1171	12.20
Bamethan	3703-79-5	$C_{12}H_{19}NO_2$	[M+H]+	210.1489	192.1375	5.28
Betamethasone	378-44-9	$C_{22}H_{29}FO_5$	[M+H]+	393.2072	355.1892	16.00
Betaxolol	63659-18-7	$C_{18}H_{29}NO_3$	[M+H]+	308.2220	116.1066	13.32
Bisoprolol	66722-44-9	$C_{18}H_{31}NO_4$	[M+H]+	326.2326	116.1066	12.33
Bupivacaine	38396-39-3	$C_{18}H_{28}N_2O$	[M+H]+	289.2274	140.1430	12.42
Buprenorphine	52485-79-7	$C_{29}H_{41}NO_4$	[M+H]+	468.3108	414.2650	13.15
Bupropion	34911-55-2	$C_{13}H_{18}ClNO$	[M+H]+	240.1150	184.0517	11.83
Butalbital	77-26-9	$C_{11}H_{16}N_2O_3$	[M+H]+	225.1234	182.1168	9.05
Butylone	802575-11-7	$C_{12}H_{15}NO_3$	[M+H]+	222.1125	204.1011	8.85
Caffeine	58-08-2	$C_8H_{10}N_4O_2$	[M+H]+	195.0877	138.0656	8.46
Cannabinol	521-35-7	$C_{21}H_{26}O_2$	[M+H]+	311.2006	223.1129	23.99
Capsaicin	404-86-4	$C_{18}H_{27}NO_3$	[M+H]+	306.2064	137.0597	18.69
Carbamazepine	298-46-4	$C_{15}H_{12}N_2O$	[M+H]+	237.1022	194.0955	14.68
Celiprolol	56980-93-9	$C_{20}H_{33}N_3O_4$	[M+H]+	380.2544	324.1909	11.42
Cetirizine	83881-51-0	$C_{21}H_{25}ClN_2O_3$	[M+H]+	389.1627	201.0458	16.24
Cimetidine	51481-61-9	$C_{10}H_{16}N_6S$	[M+H]+	253.1230	159.0690	3.44
Citalopram	59729-33-8	$C_{20}H_{21}FN_2O$	[M+H]+	325.1711	262.1021	13.11
Clarithromycin	81103-11-9	$C_{38}H_{69}NO_{13}$	[M+H]+	748.4842	158.1170	16.50
Clenbuterol	37148-27-9	$C_{12}H_{18}Cl_2N_2O$	[M+H]+	277.0869	203.0132	10.12
Clindamycin	18323-44-9	$C_{18}H_{33}ClN_2O_5S$	[M+H]+	425.1872	126.1272	13.30
Codeine	76-57-3	$C_{18}H_{21}NO_3$	[M+H]+	300.1594	215.1057	4.67
Crotamiton	124236-29-9	$C_{13}H_{17}NO$	[M+H]+	204.1383	136.1112	17.32
Cortisone	53-06-5	$C_{21}H_{28}O_5$	[M+H]+	361.2010	163.1112	14.33
Cycloheximide	66-81-9	$C_{15}H_{23}NO_4$	[M+H]+	282.1700	247.1324	12.83
Cyclopentolate	512-15-2	$C_{17}H_{25}NO_3$	[M+H]+	292.1907	274.1794	12.50
Desipramine	50-47-5	$C_{18}H_{22}N_2$	[M+H]+	267.1856	238.9279	15.18
Desomorphine	427-00-9	$C_{17}H_{21}NO_2$	[M+H]+	272.1645	215.1058	7.44
Desvenlafaxine (O-Desmethyl Venlafaxine)	93413-62-8	$C_{16}H_{25}NO_2$	[M+H]+	264.1958	246.1841	10.40
Detomidine	76631-46-4	$C_{12}H_{14}N_2$	[M+H]+	187.1230	81.0443	11.64

**Table S9.** LC-HRMS method for 335 suspect and nontarget OMPs (continued)						
Compound Name	CAS	Molecular Formula	Adduct	Exact Mass (*m/z*)	Diagnostic Fragment (*m/z*)	RT (min)
Dexpanthenol	81-13-0	$C_9H_{19}NO_4$	[M+H]+	206.1387	189.1111	3.81
Dextromethorphan	125-71-3	$C_{18}H_{25}NO$	[M+H]+	272.2009	215.1421	13.00
Diazepam	439-14-5	$C_{16}H_{13}ClN_2O$	[M+H]+	285.0789	154.0410	17.45
Diclofenac	15307-86-5	$C_{14}H_{11}Cl_2NO_2$	[M+H]+	296.0240	215.0491	19.88
Dienogest	65928-58-7	$C_{20}H_{25}NO_2$	[M+H]+	312.1958	161.0960	15.35
Dihydrocodeine	125-28-0	$C_{18}H_{23}NO_3$	[M+H]+	302.1751	284.1279	4.66
Dihydromorphine	509-60-4	$C_{17}H_{21}NO_3$	[M+H]+	288.1594	231.1027	1.95
Diltiazem	42399-41-7	$C_{22}H_{26}N_2O_4S$	[M+H]+	415.1686	397.1172	13.88
Dinoprostone	363-24-6	$C_{20}H_{32}O_5$	[M-H]-	351.2177	333.2072	17.83
Diphenhydramine	58-73-1	$C_{17}H_{21}NO$	[M+H]+	256.1696	224.0828	13.22
Dobutamine	34368-04-2	$C_{18}H_{23}NO_3$	[M+H]+	302.1751	137.0593	9.09
Dopamine	51-61-6	$C_8H_{11}NO_2$	[M+H]+	154.0863	137.0590	1.48
Doxylamine	469-21-6	$C_{17}H_{22}N_2O$	[M+H]+	271.1805	182.0959	8.14
Enalapril	75847-73-3	$C_{20}H_{28}N_2O_5$	[M+H]+	377.2071	234.1479	13.15
Enalaprilat	76420-72-9	$C_{18}H_{24}N_2O_5$	[M+H]+	349.1758	206.1173	10.48
Ephedrine	299-42-3	$C_{10}H_{15}NO$	[M+H]+	166.1226	148.1156	5.19
Epinephrine	51-43-4	$C_9H_{13}NO_3$	[M+H]+	184.0968	166.0855	1.60
Estriol	50-27-1	$C_{18}H_{24}O_3$	[M+H]+	289.1798	253.1577	13.48
Estrone	53-16-7	$C_{18}H_{22}O_2$	[M+H]+	271.1693	197.0951	17.54
Ethosuximide	77-67-8	$C_7H_{11}NO_2$	[M-H]-	140.0717	NA	13.81
Fexofenadine	83799-24-0	$C_{32}H_{39}NO_4$	[M+H]+	502.2952	466.2721	14.98
Fingolimod	162359-55-9	$C_{19}H_{33}NO_2$	[M+H]+	308.2584	255.2100	20.53
Flecainide	54143-55-4	$C_{17}H_{20}F_6N_2O_3$	[M+H]+	415.1451	398.1173	13.11
Fluconazole	86386-73-4	$C_{13}H_{12}F_2N_6O$	[M+H]+	307.1113	238.0774	11.06
Fluoxetine	54910-89-3	$C_{17}H_{18}F_3NO$	[M+H]+	310.1413	202.4488	16.11
Flurandrenolide	1524-88-5	$C_{24}H_{33}FO_6$	[M+H]+	437.2334	285.1645	16.94
Furosemide	54-31-9	$C_{12}H_{11}ClN_2O_5S$	[M+H]+	331.0150	312.9846	13.10
Gabapentin	60142-96-3	$C_9H_{17}NO_2$	[M+H]+	172.1332	154.1221	7.34
Gemfibrozil	25812-30-0	$C_{15}H_{22}O_3$	[M+H]+	251.1642	202.5128	21.84
Glutethimide	77-21-4	$C_{13}H_{15}NO_2$	[M+H]+	218.1176	175.0986	14.38
Griseofulvin	126-07-8	$C_{17}H_{17}ClO_6$	[M+H]+	353.0786	165.0543	15.62
Guaifenesin	93-14-1	$C_{10}H_{14}O_4$	[M+H]+	199.0965	125.0591	10.84
Hydrocodone	125-29-1	$C_{18}H_{21}NO_3$	[M+H]+	300.1594	199.0760	6.96
Hydrocortisone	50-23-7	$C_{21}H_{30}O_5$	[M+H]+	363.2166	327.1955	14.90
Hydromorphone	466-99-9	$C_{17}H_{19}NO_3$	[M+H]+	286.1438	185.0608	2.41
Ibuprofen	15687-27-1	$C_{13}H_{18}O_2$	[M+H]+	207.1380	189.1629	20.37
Imidocarb	27885-92-3	$C_{19}H_{20}N_6O$	[M+H]+	349.1771	188.0844	8.17
Irbesartan	138402-11-6	$C_{25}H_{28}N_6O$	[M+H]+	429.2397	207.0907	16.48
Ketamine	6740-88-1	$C_{13}H_{16}ClNO$	[M+H]+	238.0993	179.0615	9.80
Labetalol	36894-69-6	$C_{19}H_{24}N_2O_3$	[M+H]+	329.1860	162.0545	12.07
Lamotrigine	84057-84-1	$C_9H_7Cl_2N_5$	[M+H]+	256.0151	210.9816	10.90
Levamisole	14769-73-4	$C_{11}H_{12}N_2S$	[M+H]+	205.0794	178.0677	5.83
Levetiracetam	102767-28-2	$C_8H_{14}N_2O_2$	[M+H]+	171.1128	126.0908	6.14
Levorphanol	77-07-6	$C_{17}H_{23}NO$	[M+H]+	258.1852	199.1114	10.29
Lidocaine	137-58-6	$C_{14}H_{22}N_2O$	[M+H]+	235.1805	86.0958	8.90
Linezolid	165800-03-3	$C_{16}H_{20}FN_3O_4$	[M+H]+	338.1511	296.1422	11.66
Losartan	114798-26-4	$C_{22}H_{23}ClN_6O$	[M+H]+	423.1695	207.0908	16.20
Lovastatin	75330-75-5	$C_{24}H_{36}O_5$	[M+H]+	405.2636	199.1475	22.12
Maprotiline	10262-69-8	$C_{20}H_{23}N$	[M+H]+	278.1903	250.1584	15.41

Table S9. LC-HRMS method for 335 suspect and nontarget OMPs (continued)						
Compound Name	CAS	Molecular Formula	Adduct	Exact Mass (*m/z*)	Diagnostic Fragment (*m/z*)	RT (min)
Medroxyprogesterone	520-85-4	$C_{22}H_{32}O_3$	[M+H]+	345.2424	123.0800	19.54
Melatonin	73-31-4	$C_{13}H_{16}N_2O_2$	[M+H]+	233.1285	174.0907	11.39
Memantine	19982-08-2	$C_{12}H_{21}N$	[M+H]+	180.1747	163.1476	13.39
Meperidine	57-42-1	$C_{15}H_{21}NO_2$	[M+H]+	248.1645	220.1328	11.27
Metaraminol	54-49-9	$C_9H_{13}NO_2$	[M+H]+	168.1019	150.0907	1.92
Metaxalone	1665-48-1	$C_{12}H_{15}NO_3$	[M+H]+	222.1125	161.0956	15.11
Metformin	657-24-9	$C_4H_{11}N_5$	[M+H]+	130.1087	113.0817	1.64
Methamphetamine	537-46-2	$C_{10}H_{15}N$	[M+H]+	150.1277	91.0538	7.77
Methcathinone	5650-44-2	$C_{10}H_{13}NO$	[M+H]+	164.1070	146.0961	4.99
Methocarbamol	532-03-6	$C_{11}H_{15}NO_5$	[M+H]+	242.1023	163.0748	11.28
Methylphenidate	113-45-1	$C_{14}H_{19}NO_2$	[M+H]+	234.1489	117.0694	10.78
Methyltestosterone	58-18-4	$C_{20}H_{30}O_2$	[M+H]+	303.2319	202.4471	19.13
Metoprolol	51384-51-1	$C_{15}H_{25}NO_3$	[M+H]+	268.1907	191.1058	10.60
Molindone	7416-34-4	$C_{16}H_{24}N_2O_2$	[M+H]+	277.1911	100.0753	10.26
Morphine	57-27-2	$C_{17}H_{19}NO_3$	[M+H]+	286.1438	201.0904	1.92
Mycophenolic Acid	24280-93-1	$C_{17}H_{20}O_6$	[M+H]+	321.1333	207.0648	16.60
Nadolol	42200-33-9	$C_{17}H_{27}NO_4$	[M+H]+	310.2013	254.1376	9.06
Nalidixic Acid	389-08-2	$C_{12}H_{12}N_2O_3$	[M+H]+	233.0921	215.0808	13.56
Nalorphine	62-67-9	$C_{19}H_{21}NO_3$	[M+H]+	312.1594	251.0690	10.00
Naloxone	465-65-6	$C_{19}H_{21}NO_4$	[M+H]+	328.1543	310.1455	4.68
Naproxen	22204-53-1	$C_{14}H_{14}O_3$	[M+H]+	231.1016	188.0688	17.24
N-Ethylamphetamine	33817-11-7	$C_{11}H_{17}N$	[M+H]+	164.1434	119.0850	8.84
Nevirapine	129618-40-2	$C_{15}H_{14}N_4O$	[M+H]+	267.1240	226.0842	12.07
Nicotine	54-11-5	$C_{10}H_{14}N_2$	[M+H]+	163.1230	132.0802	1.45
Norgestrel	6533-00-2	$C_{21}H_{28}O_2$	[M+H]+	313.2162	202.9126	18.95
Nortriptyline	72-69-5	$C_{19}H_{21}N$	[M+H]+	264.1747	233.1318	15.15
Ondansetron	99614-02-5	$C_{18}H_{19}N_3O$	[M+H]+	294.1601	212.1063	11.07
Oxcarbazepine	28721-07-5	$C_{15}H_{12}N_2O_2$	[M+H]+	253.0972	210.0908	13.22
Oxprenolol	6452-71-7	$C_{15}H_{23}NO_3$	[M+H]+	266.1751	116.1065	11.83
Oxycodone	76-42-6	$C_{18}H_{21}NO_4$	[M+H]+	316.1543	298.1457	6.06
Oxymorphone	76-41-5	$C_{17}H_{19}NO_4$	[M+H]+	302.1387	284.1294	2.16
Paliperidone	144598-75-4	$C_{23}H_{27}FN_4O_3$	[M+H]+	427.2140	207.1126	11.25
Pentazocine	359-83-1	$C_{19}H_{27}NO$	[M+H]+	286.2165	218.1537	11.83
Pentedrone	879722-57-3	$C_{12}H_{17}NO$	[M+H]+	192.1383	174.1271	10.32
Phencyclidine	77-10-1	$C_{17}H_{25}N$	[M+H]+	244.2060	159.1160	12.10
Phendimetrazine	634-03-7	$C_{12}H_{17}NO$	[M+H]+	192.1383	174.1271	15.75
Phenmetrazine	134-49-6	$C_{11}H_{15}NO$	[M+H]+	178.1226	134.0956	7.54
Phentermine	122-09-8	$C_{10}H_{15}N$	[M+H]+	150.1277	133.1006	9.43
Phenylephrine	59-42-7	$C_9H_{13}NO_2$	[M+H]+	168.1019	150.0906	9.76
Phenytoin	57-41-0	$C_{15}H_{12}N_2O_2$	[M+H]+	253.0972	182.0958	14.25
Pilocarpine	92-13-7	$C_{11}H_{16}N_2O_2$	[M+H]+	209.1285	163.1226	2.10
Pirlimycin	79548-73-5	$C_{17}H_{31}ClN_2O_5S$	[M+H]+	411.1715	363.1671	13.02
Prasterone	53-43-0	$C_{19}H_{28}O_2$	[M+H]+	289.2162	271.2043	18.77
Praziquantel	55268-74-1	$C_{19}H_{24}N_2O_2$	[M+H]+	313.1911	203.1193	17.28
Pregabalin	148553-50-8	$C_8H_{17}NO_2$	[M+H]+	160.1332	142.1221	7.12
Prilocaine	721-50-6	$C_{13}H_{20}N_2O$	[M+H]+	221.1648	136.0756	9.63
Primidone	125-33-7	$C_{12}H_{14}N_2O_2$	[M+H]+	219.1128	162.0905	10.90
Propafenone	54063-53-5	$C_{21}H_{27}NO_3$	[M+H]+	342.2064	116.1064	14.93
Propoxyphene	469-62-5	$C_{22}H_{29}NO_2$	[M+H]+	340.2271	266.1893	14.70

Table S9. LC-HRMS method for 335 suspect and nontarget OMPs (continued)						
Compound Name	CAS	Molecular Formula	Adduct	Exact Mass (*m/z*)	Diagnostic Fragment (*m/z*)	RT (min)
Propranolol	525-66-6	$C_{16}H_{21}NO_2$	[M+H]+	260.1645	183.0797	13.05
Protriptyline	438-60-8	$C_{19}H_{21}N$	[M+H]+	264.1747	233.1318	15.68
Pseudoephedrine	90-82-4	$C_{10}H_{15}NO$	[M+H]+	166.1226	148.1156	5.61
Pyrovalerone	3563-49-3	$C_{16}H_{23}NO$	[M+H]+	246.1852	175.1111	12.36
Quinine	130-95-0	$C_{20}H_{24}N_2O_2$	[M+H]+	325.1911	307.1793	9.88
Ramipril	87333-19-5	$C_{23}H_{32}N_2O_5$	[M+H]+	417.2384	234.1480	15.41
Ranitidine	66357-35-5	$C_{13}H_{22}N_4O_3S$	[M+H]+	315.1485	176.0480	3.12
Rimantadine	13392-28-4	$C_{12}H_{21}N$	[M+H]+	180.1747	163.1476	13.46
Ropivacaine	84057-95-4	$C_{17}H_{26}N_2O$	[M+H]+	275.2118	126.1272	11.19
Sertraline	79617-96-2	$C_{17}H_{17}Cl_2N$	[M+H]+	306.0811	275.0381	16.60
Secobarbital	76-73-3	$C_{12}H_{18}N_2O_3$	[M+H]+	239.1390	182.1282	10.60
Sotalol	3930-20-9	$C_{12}H_{20}N_2O_3S$	[M+H]+	273.1267	213.0684	2.76
Sitagliptin	486460-32-6	$C_{16}H_{15}F_6N_5O$	[M+H]+	408.1254	235.0792	10.89
Stavudine	3056-17-5	$C_{10}H_{12}N_2O_4$	[M+H]+	225.0870	182.1168	4.24
Sulfadoxine	2447-57-6	$C_{12}H_{14}N_4O_4S$	[M+H]+	311.0809	156.0109	10.11
Sulfamethazine	57-68-1	$C_{12}H_{14}N_4O_2S$	[M+H]+	279.0910	204.0431	8.43
Sulfamethoxazole	723-46-6	$C_{10}H_{11}N_3O_3S$	[M+H]+	254.0594	156.0107	9.72
Sulfapyridine	144-83-2	$C_{11}H_{11}N_3O_2S$	[M+H]+	250.0645	156.0108	5.69
Sulfisomidine	515-64-0	$C_{12}H_{14}N_4O_2S$	[M+H]+	279.0910	186.0326	3.89
Tacrolimus	104987-11-3	$C_{44}H_{69}NO_{12}$	[M+H]+	804.4893	202.4592	22.27
Tapentadol	175591-23-8	$C_{14}H_{23}NO$	[M+H]+	222.1852	121.0643	11.03
Telmisartan	144701-48-4	$C_{33}H_{30}N_4O_2$	[M+H]+	515.2442	497.2315	16.23
Thebaine	115-37-7	$C_{19}H_{21}NO_3$	[M+H]+	312.1594	201.0883	4.09
Tolperisone	728-88-1	$C_{16}H_{23}NO$	[M+H]+	246.1852	98.0959	11.45
Tramadol	27203-92-5	$C_{16}H_{25}NO_2$	[M+H]+	264.1958	246.1840	10.22
Trenbolone	10161-33-8	$C_{18}H_{22}O_2$	[M+H]+	271.1693	253.1580	17.00
Triamterene	396-01-0	$C_{12}H_{11}N_7$	[M+H]+	254.1149	237.0874	9.63
Trihexyphenidyl	144-11-6	$C_{20}H_{31}NO$	[M+H]+	302.2478	98.0959	14.95
Trimethoprim	738-70-5	$C_{14}H_{18}N_4O_3$	[M+H]+	291.1452	230.1152	8.45
Valsartan	137862-53-4	$C_{24}H_{29}N_5O_3$	[M+H]+	436.2343	207.0911	18.09
Varenicline	249296-44-4	$C_{13}H_{13}N_3$	[M+H]+	212.1182	169.0757	6.64
Venlafaxine	93413-69-5	$C_{17}H_{27}NO_2$	[M+H]+	278.2115	260.2000	12.49
Zidovudine	30516-87-1	$C_{10}H_{13}N_5O_4$	[M+H]+	268.1040	139.0306	8.88
10,11-Dihydro-10-hydroxy Carbamazepine	29331-92-8	$C_{15}H_{14}N_2O_2$	[M+H]+	255.1128	237.1012	12.49
1-Methyluric Acid	708-79-2	$C_6H_6N_4O_3$	[M+H]+	183.0513	155.0562	NA
2-Ethyl-2-phenylmalonamide (PEMA)	7206-76-0	$C_{11}H_{14}N_2O_2$	[M+H]+	207.1128	130.9783	8.16
2-Ethylidene-1,5-dimethyl-3,3-diphenylpyrrolidine (EDDP)	30223-73-5	$C_{20}H_{23}N$	[M+H]+	278.1903	249.1502	13.07
3-Hydroxy Cotinine	34834-67-8	$C_{10}H_{12}N_2O_2$	[M+H]+	193.0972	174.1496	1.63
Albendazole-2-aminosulfone	80983-34-2	$C_{10}H_{13}N_3O_2S$	[M+H]+	240.0801	165.0532	7.74
Albendazole Sulfoxide	54029-12-8	$C_{12}H_{15}N_3O_3S$	[M+H]+	282.0907	208.0172	11.18
Androsterone	53-41-8	$C_{19}H_{30}O_2$	[M+H]+	291.2319	255.2090	20.42
Benzoylecgonine	519-09-5	$C_{16}H_{19}NO_4$	[M+H]+	290.1387	168.1012	9.77
Carbamazepine-10,11-epoxide	36507-30-9	$C_{15}H_{12}N_2O_2$	[M+H]+	253.0972	210.0910	12.65
Cotinine	486-56-6	$C_{10}H_{12}N_2O$	[M+H]+	177.1022	149.0224	1.76
Cotinine N-Oxide	36508-80-2	$C_{10}H_{12}N_2O_2$	[M+H]+	193.0972	162.0547	2.15
Ecgonine Methyl Ester	7143-09-1	$C_{10}H_{17}NO_3$	[M+H]+	200.1281	182.1168	1.48
Hydroxybupropion	92264-81-8	$C_{13}H_{18}ClNO_2$	[M+H]+	256.1099	238.0985	11.25
Ibuprofen Carboxylic Acid	15935-54-3	$C_{13}H_{16}O_4$	[M-H]-	235.0976	194.0954	14.75
Lidocaine N-Oxide	2903-45-9	$C_{14}H_{22}N_2O_2$	[M+H]+	251.1754	164.1057	10.32

**Table S9.** LC-HRMS method for 335 suspect and nontarget OMPs (continued)						
Compound Name	CAS	Molecular Formula	Adduct	Exact Mass (*m/z*)	Diagnostic Fragment (*m/z*)	RT (min)
Metoprolol Acid (Atenolol Acid)	56392-14-4	$C_{14}H_{21}NO_4$	[M+H]+	268.1543	191.0695	8.23
N4-Acetylsulfamethoxazole	21312-10-7	$C_{12}H_{13}N_3O_4S$	[M+H]+	296.0700	198.0212	11.31
N-Desmethyl Citalopram	62498-67-3	$C_{19}H_{19}FN_2O$	[M+H]+	311.1554	149.0225	8.15
N-Desmethyl Tramadol	73806-55-0	$C_{15}H_{23}NO_2$	[M+H]+	250.1802	189.1264	10.86
N-Desmethyl Venlafaxine	149289-30-5	$C_{16}H_{25}NO_2$	[M+H]+	264.1958	215.1425	12.56
Norcocaine	18717-72-1	$C_{16}H_{19}NO_4$	[M+H]+	290.1387	168.1012	11.15
Norfentanyl	1609-66-1	$C_{14}H_{20}N_2O$	[M+H]+	233.1648	150.0919	10.12
Norlidocaine (Monoethylglycinexylidide)	7728-40-7	$C_{12}H_{18}N_2O$	[M+H]+	207.1492	122.0956	7.82
Noroxycodone	57664-96-7	$C_{17}H_{19}NO_4$	[M+H]+	302.1387	NA	15.75
O-Desmethyl Tramadol	73986-53-5	$C_{15}H_{23}NO_2$	[M+H]+	250.1802	189.1264	10.86
Ritalinic Acid	19395-41-6	$C_{13}H_{17}NO_2$	[M+H]+	220.1332	84.0804	9.87
Tramadol N-Oxide	147441-56-3	$C_{16}H_{25}NO_3$	[M+H]+	280.1907	262.1790	10.95
Venlafaxine N-Oxide	1094598-37-4	$C_{17}H_{27}NO_3$	[M+H]+	294.2064	121.0643	13.36
(4-Chloro-2-methylphenoxy)acetic Acid (MCPA)	94-74-6	$C_9H_9ClO_3$	[M-H]-	199.0168	141.0114	16.54
2,4-Dichlorophenoxyacetic Acid (2,4-D)	94-75-7	$C_8H_6Cl_2O_3$	[M-H]-	218.9621	160.9570	16.15
2-Naphthoxyacetic Acid	120-23-0	$C_{12}H_{10}O_3$	[M-H]-	201.0557	143.0505	15.34
8-Hydroxyquinoline	148-24-3	C_9H_7NO	[M+H]+	146.0600	118.0648	3.08
Abscisic Acid	21293-29-8	$C_{15}H_{20}O_4$	[M+H]+	265.1434	222.0544	13.66
Acetamiprid	135410-20-7	$C_{10}H_{11}ClN_4$	[M+H]+	223.0745	126.0100	10.93
Aldicarb	116-06-3	$C_7H_{14}N_2O_2S$	[M+Na]+	213.0668	170.1028	12.40
Ametryn	834-12-8	$C_9H_{17}N_5S$	[M+H]+	228.1277	186.0801	14.06
Atrazine	1912-24-9	$C_8H_{14}ClN_5$	[M+H]+	216.1011	174.0532	15.46
Bifenazate	149877-41-8	$C_{17}H_{20}N_2O_3$	[M+H]+	301.1547	198.0908	18.47
Butralin	33629-47-9	$C_{14}H_{21}N_3O_4$	[M+H]+	296.1605	240.0972	23.40
Camphor	76-22-2	$C_{10}H_{16}O$	[M+H]+	153.1274	135.1162	16.35
Carbaryl	63-25-2	$C_{12}H_{11}NO_2$	[M+H]+	202.0863	145.0642	14.47
Carbendazim	10605-21-7	$C_9H_9N_3O_2$	[M+H]+	192.0768	148.1115	6.76
Carbofuran	1563-66-2	$C_{12}H_{15}NO_3$	[M+H]+	222.1125	165.0903	13.90
Clothianidin	210880-92-5	$C_6H_8ClN_5O_2S$	[M+H]+	250.0160	169.0537	9.99
Dimethachlor	50563-36-5	$C_{13}H_{18}ClNO_2$	[M+H]+	256.1099	224.0828	16.28
Diuron	330-54-1	$C_9H_{10}Cl_2N_2O$	[M+H]+	233.0243	159.9709	16.03
Ethoxyquin	91-53-2	$C_{14}H_{19}NO$	[M+H]+	218.1539	175.1114	13.92
Fenamidone	161326-34-7	$C_{17}H_{17}N_3OS$	[M+H]+	312.1165	236.1179	17.42
Fluridone	59756-60-4	$C_{19}H_{14}F_3NO$	[M+H]+	330.1100	310.1030	16.63
Imazapyr	81334-34-1	$C_{13}H_{15}N_3O_3$	[M+H]+	262.1186	149.0344	10.01
Imidacloprid	138261-41-3	$C_9H_{10}ClN_5O_2$	[M+H]+	256.0596	175.0970	9.97
Indole-3-butyric Acid	133-32-4	$C_{12}H_{13}NO_2$	[M+H]+	204.1019	186.0907	14.53
Isoproturon	34123-59-6	$C_{12}H_{18}N_2O$	[M+H]+	207.1492	165.1016	15.82
Kresoxim-methyl	143390-89-0	$C_{18}H_{19}NO_4$	[M+H]+	314.1387	222.0908	19.65
Malathion	121-75-5	$C_{10}H_{19}O_6PS_2$	[M+H]+	331.0433	109.0644	17.89
Metalaxyl	57837-19-1	$C_{15}H_{21}NO_4$	[M+H]+	280.1543	220.1324	15.89
Mecoprop	93-65-2	$C_{10}H_{11}ClO_3$	[M-H]-	213.0324	141.0114	17.94
Metolachlor	51218-45-2	$C_{15}H_{22}ClNO_2$	[M+H]+	284.1412	252.1139	19.10
Monuron	150-68-5	$C_9H_{11}ClN_2O$	[M+H]+	199.0633	126.0099	13.22
Napropamide	15299-99-7	$C_{17}H_{21}NO_2$	[M+H]+	272.1645	171.0800	18.87
Oxamyl	23135-22-0	$C_7H_{13}N_3O_3S$	[M+H]+	220.0750	116.9735	7.07
Piperonyl Butoxide	51-03-6	$C_{19}H_{30}O_5$	[M+H]+	339.2166	202.6737	22.37
Pirimicarb	23103-98-2	$C_{11}H_{18}N_4O_2$	[M+H]+	239.1503	182.1282	10.54
Prometon	1610-18-0	$C_{10}H_{19}N_5O$	[M+H]+	226.1662	184.1186	13.38

Table S9. LC-HRMS method for 335 suspect and nontarget OMPs (continued)

Compound Name	CAS	Molecular Formula	Adduct	Exact Mass (*m/z*)	Diagnostic Fragment (*m/z*)	RT (min)
Prometryn	7287-19-6	$C_{10}H_{19}N_5S$	[M+H]+	242.1434	200.0961	15.62
Propazine	139-40-2	$C_9H_{16}ClN_5$	[M+H]+	230.1167	188.0691	17.07
Pyracarbolid	24691-76-7	$C_{13}H_{15}NO_2$	[M+H]+	218.1176	175.0985	13.84
Pyrimethanil	53112-28-0	$C_{12}H_{13}N_3$	[M+H]+	200.1182	183.0911	14.41
Siduron	1982-49-6	$C_{14}H_{20}N_2O$	[M+H]+	233.1648	137.0704	17.23
Simazine	122-34-9	$C_7H_{12}ClN_5$	[M+H]+	202.0854	132.0318	13.62
Terbumeton	33693-04-8	$C_{10}H_{19}N_5O$	[M+H]+	226.1662	170.1653	13.40
Terbuthylazine	5915-41-3	$C_9H_{16}ClN_5$	[M+H]+	230.1167	174.0535	17.40
Thiabendazole	148-79-8	$C_{10}H_7N_3S$	[M+H]+	202.0433	175.0321	8.33
Terbutryn	886-50-0	$C_{10}H_{19}N_5S$	[M+H]+	242.1434	186.0815	15.88
Thiamethoxam	153719-23-4	$C_8H_{10}ClN_5O_3S$	[M+H]+	292.0266	211.0641	8.42
Tridemorph	24602-86-6	$C_{19}H_{39}NO$	[M+H]+	298.3104	130.1221	19.30
Trinexapac-ethyl	95266-40-3	$C_{13}H_{16}O_5$	[M+H]+	253.1071	207.0644	15.88
2-Aminobenzimidazole	934-32-7	$C_7H_7N_3$	[M+H]+	134.0713	107.0598	4.40
Atrazine-2-hydroxy	2163-68-0	$C_8H_{15}N_5O$	[M+H]+	198.1349	156.0876	9.37
Atrazine-desethyl	6190-65-4	$C_6H_{10}ClN_5$	[M+H]+	188.0698	146.0224	11.44
Atrazine-desisopropyl	1007-28-9	$C_5H_8ClN_5$	[M+H]+	174.0541	132.0318	8.99
Carbofuran-3-hydroxy	16655-82-6	$C_{12}H_{15}NO_4$	[M+H]+	238.1074	163.0746	10.85
Metolachlor Ethanesulfonic Acid (Metolachlor ESA)	171118-09-5	$C_{15}H_{23}NO_5S$	[M+H]+	330.1370	298.1100	14.77
Metolachlor Oxanilic Acid (Metolachlor OA)	152019-73-3	$C_{15}H_{21}NO_4$	[M+H]+	280.1543	248.1274	16.02
N-(2,4-Dimethylphenyl)formamide	60397-77-5	$C_9H_{11}NO$	[M+H]+	150.0913	132.0800	15.72
Propachlor Oxanilic Acid (Propachlor OA)	70628-36-3	$C_{11}H_{13}NO_3$	[M+H]+	208.0968	120.0438	11.35
1H-Benzotriazole	95-14-7	$C_6H_5N_3$	[M+H]+	120.0556	120.0556	9.12
2-Hydroxybenzothiazole	934-34-9	C_7H_5NOS	[M+H]+	152.0165	124.0211	12.73
4-Methyl-1H-benzotriazole	29878-31-7	$C_7H_7N_3$	[M+H]+	134.0713	106.0647	11.80
5-Methyl-1H-benzotriazole	136-85-6	$C_7H_7N_3$	[M+H]+	134.0713	106.0647	11.82
Benzophenone	119-61-9	$C_{13}H_{10}O$	[M+H]+	183.0804	105.0330	17.49
Benzophenone-3 (Oxybenzone)	131-57-7	$C_{14}H_{12}O_3$	[M+H]+	229.0859	151.0384	19.23
Benzothiazole	95-16-9	C_7H_5NS	[M+H]+	136.0216	122.3269	12.97
Benzyl Butyl Phthalate	85-68-7	$C_{19}H_{20}O_4$	[M+H]+	313.1434	149.0230	21.37
Butylparaben	94-26-8	$C_{11}H_{14}O_3$	[M-H]-	193.0870	137.0246	17.60
Dibutyl Phthalate	84-74-2	$C_{16}H_{22}O_4$	[M+H]+	279.1591	149.0227	21.34
Diethyl Phthalate	84-66-2	$C_{12}H_{14}O_4$	[M+H]+	223.0965	149.0228	16.07
Diisobutyl Phthalate	84-69-5	$C_{16}H_{22}O_4$	[M+H]+	279.1591	149.0228	21.55
Dimethyl Phthalate	131-11-3	$C_{10}H_{10}O_4$	[M+H]+	195.0652	163.0383	13.18
Ethyl Butylacetylaminopropionate	52304-36-6	$C_{11}H_{21}NO_3$	[M+H]+	216.1594	170.1170	14.67
Ethylparaben	120-47-8	$C_9H_{10}O_3$	[M+H]+	167.0703	105.0328	13.85
Icaridin	119515-38-7	$C_{12}H_{23}NO_3$	[M+H]+	230.1751	130.1221	16.73
Isopropylparaben	4191-73-5	$C_{10}H_{12}O_3$	[M+H]+	181.0859	139.0385	15.45
Melamine	108-78-1	$C_3H_6N_6$	[M+H]+	127.0727	113.0184	1.64
N,N-Diethyl-3-methylbenzamide (DEET)	134-62-3	$C_{12}H_{17}NO$	[M+H]+	192.1383	119.0484	15.75
Propylparaben	94-13-3	$C_{10}H_{12}O_3$	[M-H]-	179.0714	137.0248	15.80
Sucralose	56038-13-2	$C_{12}H_{19}Cl_3O_8$	[M+FA-H]-	441.0128	395.0071	9.24
Triclosan	3380-34-5	$C_{12}H_7Cl_3O_2$	[M-H]-	286.9439	130.3132	21.94
1-Methyl-1H-benzotriazole	13351-73-0	$C_7H_7N_3$	[M+H]+	134.0713	106.0646	10.06
Carbanilide	102-07-8	$C_{13}H_{12}N_2O$	[M+H]+	213.1022	94.0646	15.01
Galaxolidone	507442-49-1	$C_{18}H_{24}O_2$	[M+H]+	273.1849	240.1500	21.86
3-Cyclohexyl-1,1-dimethylurea (C-DMU)	31468-12-9	$C_9H_{18}N_2O$	[M+H]+	171.1492	89.0705	12.63
1,1-Dicyclohexyl-3-phenylurea	5765-54-8	$C_{19}H_{28}N_2O$	[M+H]+	301.2274	219.1481	22.39

Table S9. LC-HRMS method for 335 suspect and nontarget OMPs (continued)						
Compound Name	CAS	Molecular Formula	Adduct	Exact Mass (*m/z*)	Diagnostic Fragment (*m/z*)	RT (min)
1,3-Di-o-tolylguanidine (DTG)	97-39-2	$C_{15}H_{17}N_3$	[M+H]+	240.1495	133.0754	11.11
1,3-Diphenylguanidine (DPG)	102-06-7	$C_{13}H_{13}N_3$	[M+H]+	212.1182	195.0911	9.69
4-Methylbenzenesulfonamide	70-55-3	$C_7H_9NO_2S$	[M+H]+	172.0427	158.0158	9.60
2-Phenyl-2-imidazoline	936-49-2	$C_9H_{10}N_2$	[M+H]+	147.0917	118.9419	3.01
2-Phenylbenzimidazole-5-sulfonic Acid (Ensulizole)	27503-81-7	$C_{13}H_{10}N_2O_3S$	[M+H]+	275.0485	226.0725	7.59
Caprolactam	105-60-2	$C_6H_{11}NO$	[M+H]+	114.0913	96.0802	6.77
N-(1,3-Dimethylbutyl)-N'-phenyl-1,4-phenylenediamine (6PPD)	793-24-8	$C_{18}H_{24}N_2$	[M+H]+	269.2012	184.0988	16.02
N-Cyclohexyl-N-methylcyclohexanamine	7560-83-0	$C_{13}H_{25}N$	[M+H]+	196.2060	114.1272	10.69
N-Cyclohexyl-N'-phenylurea (CPU)	886-59-9	$C_{13}H_{18}N_2O$	[M+H]+	219.1492	137.0703	16.08
N-Ethylphthalimide	5022-29-7	$C_{10}H_9NO_2$	[M+H]+	176.0706	148.0388	7.32
N-Ethyl-p-toluenesulfonamide	80-39-7	$C_9H_{13}NO_2S$	[M+H]+	200.0740	155.0156	13.37
N-Isopropyl-N'-phenyl-p-phenylenediamine (IPPD)	101-72-4	$C_{15}H_{18}N_2$	[M+H]+	227.1543	184.0989	12.79
N-Phenylbenzenesulfonamide	1678-25-7	$C_{12}H_{11}NO_2S$	[M+H]+	234.0583	93.0569	14.63
N-Phenyl-p-phenylenediamine (4-Aminodiphenylamine)	101-54-2	$C_{12}H_{12}N_2$	[M+H]+	185.1073	168.0801	10.37
N,N'-Diphenyl-p-phenylenediamine (DPPD)	74-31-7	$C_{18}H_{16}N_2$	[M+H]+	261.1386	184.0992	20.20
Hexa(methoxymethyl)melamine (HMMM)	3089-11-0	$C_{15}H_{30}N_6O_6$	[M+H]+	391.2300	149.0227	14.66
Triphenylphosphine Oxide	791-28-6	$C_{18}H_{15}OP$	[M+H]+	279.0933	219.0563	17.04
2,4-Quinolinediol *	86-95-3	$C_9H_7NO_2$	[M+H]+	162.0550	144.0439	11.73
2,6-Dimethoxyphenol *	91-10-1	$C_8H_{10}O_3$	[M+H]+	155.0703	95.0490	11.15
4-Methylbenzophenone *	134-84-9	$C_{14}H_{12}O$	[M+H]+	197.0961	105.0329	18.83
Octadecylamine *	124-30-1	$C_{18}H_{39}N$	[M+H]+	270.3155	203.5501	24.60
Acridine *	260-94-6	$C_{13}H_9N$	[M+H]+	180.0808	130.7704	9.53
Berberine *	633-65-8	$C_{20}H_{18}NO_4^+$	[M]+	336.1230	292.0965	12.04
Bis(2-ethylhexyl) Adipate *	103-23-1	$C_{22}H_{42}O_4$	[M+H]+	371.3156	111.0432	26.52
Daidzein *	486-66-8	$C_{15}H_{10}O_4$	[M+H]+	255.0652	199.0745	13.50
Dextrorphan *	125-73-5	$C_{17}H_{23}NO$	[M+H]+	258.1852	199.1116	10.46
Diheptyl Phthalate *	3648-21-3	$C_{22}H_{34}O_4$	[M+H]+	363.2530	149.0225	25.89
Di(propylene glycol) Dibenzoate *	27138-31-4	$C_{20}H_{22}O_5$	[M+H]+	343.1540	163.0745	20.85
Docosahexaenoic Acid *	6217-54-5	$C_{22}H_{32}O_2$	[M+H]+	329.2475	98.9838	25.63
Hexadecyltrimethylammonium (Cetrimonium) *	112-02-7	$C_{19}H_{42}N^+$	[M]+	284.3312	203.0763	22.96
Lauramidopropyl Betaine *	86438-79-1	$C_{19}H_{38}N_2O_3$	[M+H]+	343.2954	109.1011	21.08
Monoethylhexyl Phthalate *	4376-20-9	$C_{16}H_{22}O_4$	[M+H]+	279.1591	149.0228	21.54
Monolaurin *	42-18-7	$C_{15}H_{30}O_4$	[M+H]+	275.2217	203.5570	22.78
Myristyl Sulfate *	1191-50-0	$C_{14}H_{30}O_4S$	[M-H]-	293.1781	96.9601	26.39
N-Ethyl-p-menthane-3-carboxamide *	39711-79-0	$C_{13}H_{25}NO$	[M+H]+	212.2009	170.1537	18.64
Nobiletin *	478-01-3	$C_{21}H_{22}O_8$	[M+H]+	403.1387	327.0855	17.75
Phthalic Acid *	88-99-3	$C_8H_6O_4$	[M+H]+	167.0339	130.9809	8.22
Stearamide *	124-26-5	$C_{18}H_{37}NO$	[M+H]+	284.2948	102.0908	26.49
Tributyl Citrate *	77-94-1	$C_{18}H_{32}O_7$	[M+H]+	361.2221	129.0177	21.86
Tris(2-butoxyethyl) Phosphate (TBEP) *	78-51-3	$C_{18}H_{39}O_7P$	[M+H]+	399.2506	143.0098	22.04
ar-Turmerone *	532-65-0	$C_{15}H_{20}O$	[M+H]+	217.1587	119.0847	21.03

"RT" = retention time; "NA" = not available. OMPs with an asterisk ("*") denote those prioritized via nontarget screening and confirmed or rejected by reference standards.

**Table S10.** SPE-LC-HRMS method performance for 133 OMPs confirmed in POCIS and grab samples											
Compound Name	CAS	Molecular Formula	Adduct	Exact Mass (*m/z*)	Diagnostic Fragment (*m/z*)	RT (min)	SPE Recovery (%)	Ion Suppression / Enhancement (%)	Matrix Factor (%)	LOQs (ng/L | ng/d)	R^2
Abacavir	136470-78-5	$C_{14}H_{18}N_6O$	[M+H]+	287.1615	191.1030	8.65	88.5±9.0	104.3±3.7	98.5±6.7	1.0 | 0.02	0.9980
Acetaminophen	103-90-2	$C_8H_9NO_2$	[M+H]+	152.0706	126.0100	3.65	88.3±11.5	121.7±1.6	111±14.4	23 | 0.49	0.9995
Aliskiren	173334-57-1	$C_{30}H_{53}N_3O_6$	[M+H]+	552.4007	436.3054	16.44	65.3±4.7	101.2±1.7	90.4±14.5	1.1 | 0.02	0.9928
Amantadine	768-94-5	$C_{10}H_{17}N$	[M+H]+	152.1434	135.1162	9.78	88.5±6.3	103.7±7.1	107.3±3.5	4.7 | 0.10	0.9952
Amitriptyline	50-48-6	$C_{20}H_{23}N$	[M+H]+	278.1903	233.1315	15.40	70.3±6.0	100.3±0.8	123.9±18.8	8.1 | 0.18	0.9963
Atenolol	29122-68-7	$C_{14}H_{22}N_2O_3$	[M+H]+	267.1703	190.0851	3.20	90.9±10.4	128.3±1.8	118.6±10.7	8.4 | 0.18	0.9985
Azelaic Acid	123-99-9	$C_9H_{16}O_4$	[M+H]+	189.1121	102.0546	13.17	112.8±6.3	36.5±13.2	30.9±15.1	32 | 0.70	0.9978
Azithromycin	83905-01-5	$C_{38}H_{72}N_2O_{12}$	[M+H]+	749.5158	158.1171	12.20	89.9±1.8	102.0±1.6	105.0±2.2	95 | 2.07	0.9869
Bamethan	3703-79-5	$C_{12}H_{19}NO_2$	[M+H]+	210.1489	192.1375	5.28	89.2±9.6	94.9±3.5	99.7±8.4	10 | 0.22	0.9973
Bisoprolol	66722-44-9	$C_{18}H_{31}NO_4$	[M+H]+	326.2326	116.1066	12.33	92.1±9.2	99.3±5.9	91.8±8.3	5.4 | 0.12	0.9968
Bupropion	34911-55-2	$C_{13}H_{18}ClNO$	[M+H]+	240.1150	184.0517	11.83	78.3±6.5	87.9±5.3	88.4±12.0	5.7 | 0.12	0.9984
Caffeine	58-08-2	$C_8H_{10}N_4O_2$	[M+H]+	195.0877	138.0656	8.46	94.1±9.2	86.4±4.2	87.9±6.4	1.1 | 0.02	0.9922
Carbamazepine	298-46-4	$C_{15}H_{12}N_2O$	[M+H]+	237.1022	194.0955	14.68	95.8±7.9	95.7±1.5	96.3±11.7	10 | 0.23	0.9970
Cetirizine	83881-51-0	$C_{21}H_{25}ClN_2O_3$	[M+H]+	389.1627	201.0458	16.24	182.8±18.3	12.8±3.1	109.8±12.1	9.1 | 0.20	0.9989
Citalopram	59729-33-8	$C_{20}H_{21}FN_2O$	[M+H]+	325.1711	262.1021	13.11	83.1±6.6	90.6±8.0	100.2±17.7	5.0 | 0.11	0.9958
Clarithromycin	81103-11-9	$C_{38}H_{69}NO_{13}$	[M+H]+	748.4842	158.1170	16.50	76.3±7.8	99.6±2.5	108.8±12.8	9.2 | 0.20	0.9953
Clindamycin	18323-44-9	$C_{18}H_{33}ClN_2O_5S$	[M+H]+	425.1872	126.1272	13.30	83.1±8.5	103.6±6.3	107.8±13.1	23 | 0.50	0.9927
Codeine	76-57-3	$C_{18}H_{21}NO_3$	[M+H]+	300.1594	215.1057	4.67	82.9±9.9	132.5±1.9	130.6±19.7	7.7 | 0.17	0.9948
Desipramine	50-47-5	$C_{18}H_{22}N_2$	[M+H]+	267.1856	238.9279	15.18	62.4±7.1	102.3±1.7	148.6±14.2	3.4 | 0.07	0.9963
Desvenlafaxine (O-Desmethyl Venlafaxine)	93413-62-8	$C_{16}H_{25}NO_2$	[M+H]+	264.1958	246.1841	10.40	89.7±11.0	47.6±8.5	45.3±4.7	22 | 0.48	0.9958
Dexpanthenol	81-13-0	$C_9H_{19}NO_4$	[M+H]+	206.1387	189.1111	3.81	75.4±8.4	109.5±7.5	119.2±14.0	8.4 | 0.18	0.9985
Dextromethorphan	125-71-3	$C_{18}H_{25}NO$	[M+H]+	272.2009	215.1421	13.00	73.3±6.3	90.7±8.4	117.0±20.5	0.9 | 0.02	0.9993
Diazepam	439-14-5	$C_{16}H_{13}ClN_2O$	[M+H]+	285.0789	154.0410	17.45	94.5±8.6	112.8±3.9	113.5±12.6	0.9 | 0.02	0.9968
Diltiazem	42399-41-7	$C_{22}H_{26}N_2O_4S$	[M+H]+	415.1686	397.1172	13.88	80.3±9.1	102.9±2.6	107.6±21.2	9.3 | 0.20	0.9960
Diphenhydramine	58-73-1	$C_{17}H_{21}NO$	[M+H]+	256.1696	224.0828	13.22	79.3±8.0	106.5±5.6	116.1±23.0	8.6 | 0.19	0.9988
Dopamine	51-61-6	$C_8H_{11}NO_2$	[M+H]+	154.0863	137.0590	1.48	93.5±9.4	120.6±1.3	113.1±7.4	22 | 0.48	0.9986
Doxylamine	469-21-6	$C_{17}H_{22}N_2O$	[M+H]+	271.1805	182.0959	8.14	85.6±6.6	108.9±2.9	149.0±27.4	6.7 | 0.15	0.9964
Ephedrine	299-42-3	$C_{10}H_{15}NO$	[M+H]+	166.1226	148.1156	5.19	87.7±5.3	114.8±6.1	118.8±8.7	4.2 | 0.09	0.9975
Fexofenadine	83799-24-0	$C_{32}H_{39}NO_4$	[M+H]+	502.2952	466.2721	14.98	86.0±9.9	98.0±3.1	104.8±11.7	9.5 | 0.21	0.9935
Flecainide	54143-55-4	$C_{17}H_{20}F_6N_2O_3$	[M+H]+	415.1451	398.1173	13.11	90.0±6.3	92.0±7.5	92.7±11.7	5.4 | 0.12	0.9947
Fluconazole	86386-73-4	$C_{13}H_{12}F_2N_6O$	[M+H]+	307.1113	238.0774	11.06	97.8±7.4	88.1±4.0	87.9±4.8	1.1 | 0.02	0.9914
Fluoxetine	54910-89-3	$C_{17}H_{18}F_3NO$	[M+H]+	310.1413	202.4488	16.11	79.0±10.1	99.5±9.2	98.4±5.4	5.1 | 0.11	0.9918
Gabapentin	60142-96-3	$C_9H_{17}NO_2$	[M+H]+	172.1332	154.1221	7.34	51.9±6.9	144.7±1.0	80.2±15.2	0.2 | 0.01	0.9975
Gemfibrozil	25812-30-0	$C_{15}H_{22}O_3$	[M+H]+	251.1642	202.5128	21.84	86.5±6.7	114.7±2.2	98.7±11.7	10 | 0.22	0.9950
Griseofulvin	126-07-8	$C_{17}H_{17}ClO_6$	[M+H]+	353.0786	165.0543	15.62	92.3±6.7	91.9±1.7	95.7±7.1	10 | 0.23	0.9954
Guaifenesin	93-14-1	$C_{10}H_{14}O_4$	[M+H]+	199.0965	125.0591	10.84	93.2±4.4	93.1±6.4	92.3±5.8	11 | 0.24	0.9922
Irbesartan	138402-11-6	$C_{25}H_{28}N_6O$	[M+H]+	429.2397	207.0907	16.48	84.0±9.7	117.0±5.5	112.1±19.6	8.9 | 0.19	0.9841
Labetalol	36894-69-6	$C_{19}H_{24}N_2O_3$	[M+H]+	329.1860	162.0545	12.07	85.7±5.9	89.5±4.5	129.8±2.0	7.7 | 0.17	0.9971
Lamotrigine	84057-84-1	$C_9H_7Cl_2N_5$	[M+H]+	256.0151	210.9816	10.90	92.4±9.1	89.8±1.9	89.4±6.8	11 | 0.24	0.9962
Levamisole	14769-73-4	$C_{11}H_{12}N_2S$	[M+H]+	205.0794	178.0677	5.83	87.0±11.7	121.9±2.3	113.2±11.3	8.8 | 0.19	0.9968
Levetiracetam	102767-28-2	$C_8H_{14}N_2O_2$	[M+H]+	171.1128	126.0908	6.14	94.0±9.7	116.7±1.6	113.0±8.6	8.8 | 0.19	0.9986
Levorphanol	77-07-6	$C_{17}H_{23}NO$	[M+H]+	258.1852	199.1114	10.29	78.0±10.9	119.5±3.3	127.3±28.9	1.6 | 0.03	0.9985
Lidocaine	137-58-6	$C_{14}H_{22}N_2O$	[M+H]+	235.1805	86.0958	8.90	88.4±6.6	101.4±4.8	102.8±9.7	1.9 | 0.04	0.9991
Losartan	114798-26-4	$C_{22}H_{23}ClN_6O$	[M+H]+	423.1695	207.0908	16.20	72.6±6.9	102.2±3.5	100.3±13.6	10 | 0.22	0.9947
Maprotiline	10262-69-8	$C_{20}H_{23}N$	[M+H]+	278.1903	250.1584	15.41	70.3±6.0	100.3±0.8	123.9±18.8	4.0 | 0.09	0.9969
Metaxalone	1665-48-1	$C_{12}H_{15}NO_3$	[M+H]+	222.1125	161.0956	15.11	95.9±10.6	88.3±0.7	88.3±9.8	11 | 0.25	0.9970

**Table S10.** SPE-LC-HRMS method performance for 133 OMPs confirmed in POCIS and grab samples (continued)											
Compound Name	CAS	Molecular Formula	Adduct	Exact Mass (*m/z*)	Diagnostic Fragment (*m/z*)	RT (min)	SPE Recovery (%)	Ion Suppression / Enhancement (%)	Matrix Factor (%)	LOQs (ng/L | ng/d)	R^2
Metformin	657-24-9	$C_4H_{11}N_5$	[M+H]+	130.1087	113.0817	1.64	52.7±10.8	184.4±13.7	124.9±38.2	8.0 | 0.17	0.9670
Methocarbamol	532-03-6	$C_{11}H_{15}NO_5$	[M+H]+	242.1023	163.0748	11.28	96.6±6.7	92.8±7.5	89.5±5.9	11 | 0.24	0.9944
Metoprolol	51384-51-1	$C_{15}H_{25}NO_3$	[M+H]+	268.1907	191.1058	10.60	91.7±7.4	97.7±3.9	98.6±9.4	10 | 0.22	0.9970
Molindone	7416-34-4	$C_{16}H_{24}N_2O_2$	[M+H]+	277.1911	100.0753	10.26	19.4±2.5	102.4±7.3	95.3±21.7	10 | 0.23	0.9874
Mycophenolic Acid	24280-93-1	$C_{17}H_{20}O_6$	[M+H]+	321.1333	207.0648	16.60	88.8±7.7	94.5±3.3	97.1±6.0	10 | 0.22	0.9985
Nadolol	42200-33-9	$C_{17}H_{27}NO_4$	[M+H]+	310.2013	254.1376	9.06	92.9±5.5	100.6±3.2	94.3±6.6	11 | 0.23	0.9991
Naproxen	22204-53-1	$C_{14}H_{14}O_3$	[M+H]+	231.1016	188.0688	17.24	94.7±8.0	98.6±2.4	98.9±10.1	10 | 0.22	0.9874
Oxcarbazepine	28721-07-5	$C_{15}H_{12}N_2O_2$	[M+H]+	253.0972	210.0908	13.22	97.2±7.3	86.8±4.3	88.3±9.2	5.7 | 0.12	0.9922
Phenytoin	57-41-0	$C_{15}H_{12}N_2O_2$	[M+H]+	253.0972	182.0958	14.25	93.4±8.0	84.8±3.1	83.2±10.5	12 | 0.26	0.9973
Prilocaine	721-50-6	$C_{13}H_{20}N_2O$	[M+H]+	221.1648	136.0756	9.63	93.4±8.3	107.5±6.3	108.1±3.2	0.9 | 0.02	0.9961
Propranolol	525-66-6	$C_{16}H_{21}NO_2$	[M+H]+	260.1645	183.0797	13.05	87.6±6.3	93.7±4.9	94.1±9.9	5.3 | 0.12	0.9949
Protriptyline	438-60-8	$C_{19}H_{21}N$	[M+H]+	264.1747	233.1318	15.68	59.6±8.8	103.6±0.5	201.3±16.6	2.5 | 0.05	0.9967
Ranitidine	66357-35-5	$C_{13}H_{22}N_4O_3S$	[M+H]+	315.1485	176.0480	3.12	71.2±1.6	109.9±7.4	95.4±2.3	10 | 0.23	0.9765
Rimantadine	13392-28-4	$C_{12}H_{21}N$	[M+H]+	180.1747	163.1476	13.46	64.6±6.8	103.3±2.4	163.1±68.2	6.1 | 0.13	0.9956
Sertraline	79617-96-2	$C_{17}H_{17}Cl_2N$	[M+H]+	306.0811	275.0381	16.60	73.6±10.9	123.5±7.9	120.3±7.7	4.2 | 0.09	0.9455
Sitagliptin	486460-32-6	$C_{16}H_{15}F_6N_5O$	[M+H]+	408.1254	235.0792	10.89	74.0±7.5	54.3±1.6	50.9±4.9	2.0 | 0.04	0.9956
Sotalol	3930-20-9	$C_{12}H_{20}N_2O_3S$	[M+H]+	273.1267	213.0684	2.76	90.6±12.3	133.7±0.8	124.8±15.4	20 | 0.44	0.9967
Sulfamethoxazole	723-46-6	$C_{10}H_{11}N_3O_3S$	[M+H]+	254.0594	156.0107	9.72	90.0±8.4	84.6±5.0	89.5±2.2	1.1 | 0.02	0.9991
Sulfapyridine	144-83-2	$C_{11}H_{11}N_3O_2S$	[M+H]+	250.0645	156.0108	5.69	92.2±3.3	78.8±6.1	95.7±5.4	26 | 0.57	0.9899
Telmisartan	144701-48-4	$C_{33}H_{30}N_4O_2$	[M+H]+	515.2442	497.2315	16.23	76.4±7.7	101.8±5.8	165.0±25.3	3.0 | 0.07	0.9982
Trenbolone	10161-33-8	$C_{18}H_{22}O_2$	[M+H]+	271.1693	253.1580	17.00	94.6±14.1	120.6±5.6	126.8±20.0	7.9 | 0.17	0.9980
Trimethoprim	738-70-5	$C_{14}H_{18}N_4O_3$	[M+H]+	291.1452	230.1152	8.45	90.4±7.7	95.1±3.3	95.1±4.4	1.1 | 0.02	0.9988
Valsartan	137862-53-4	$C_{24}H_{29}N_5O_3$	[M+H]+	436.2343	207.0911	18.09	11.5±0.8	91.3±2.2	110.0±1.6	91 | 1.98	0.9971
Venlafaxine	93413-69-5	$C_{17}H_{27}NO_2$	[M+H]+	278.2115	260.2000	12.49	91.5±7.3	95.9±7.2	101.6±15.0	9.8 | 0.21	0.9981
10,11-Dihydro-10-hydroxy Carbamazepine	29331-92-8	$C_{15}H_{14}N_2O_2$	[M+H]+	255.1128	237.1012	12.49	95.0±10.4	88.3±1.7	84.4±6.5	12 | 0.26	0.9934
2-Ethyl-2-phenylmalonamide (PEMA)	7206-76-0	$C_{11}H_{14}N_2O_2$	[M+H]+	207.1128	130.9783	8.16	98.0±13.1	105.4±2.9	105.8±10.9	9.5 | 0.21	0.9966
Benzoylecgonine	519-09-5	$C_{16}H_{19}NO_4$	[M+H]+	290.1387	168.1012	9.77	98.1±8.2	98.3±6.7	99.7±7.3	1.0 | 0.02	0.9939
Hydroxybupropion	92264-81-8	$C_{13}H_{18}ClNO_2$	[M+H]+	256.1099	238.0985	11.25	89.5±3.2	94.6±10.5	90.8±8.9	28 | 0.60	0.9953
N4-Acetylsulfamethoxazole	21312-10-7	$C_{12}H_{13}N_3O_4S$	[M+H]+	296.0700	198.0212	11.31	92.6±3.0	66.7±7.6	45.8±20.8	2.2 | 0.05	0.9953
Ritalinic Acid	19395-41-6	$C_{13}H_{17}NO_2$	[M+H]+	220.1332	84.0804	9.87	57.6±5.0	103.5±4.4	69.5±2.6	7.2 | 0.16	0.9980
(4-Chloro-2-methylphenoxy)acetic Acid (MCPA)	94-74-6	$C_9H_9ClO_3$	[M-H]-	199.0168	141.0114	16.54	94.6±12.1	96.6±3.4	100.1±10.4	5.0 | 0.11	0.9967
2,4-Dichlorophenoxyacetic Acid (2,4-D)	94-75-7	$C_8H_6Cl_2O_3$	[M-H]-	218.9621	160.9570	16.15	93.7±18.2	96.5±11.5	102.7±15.3	4.9 | 0.11	0.9969
Abscisic Acid	21293-29-8	$C_{15}H_{20}O_4$	[M+H]+	265.1434	222.0544	13.66	94.0±14.0	107.7±3.5	106.6±3.4	23 | 0.51	0.9915
Acetamiprid	135410-20-7	$C_{10}H_{11}ClN_4$	[M+H]+	223.0745	126.0100	10.93	97.7±8.4	86.2±3.1	85.6±7.4	5.8 | 0.13	0.9936
Ametryn	834-12-8	$C_9H_{17}N_5S$	[M+H]+	228.1277	186.0801	14.06	86.5±8.5	93.0±0.8	100.5±13.7	1.0 | 0.02	0.9906
Atrazine	1912-24-9	$C_8H_{14}ClN_5$	[M+H]+	216.1011	174.0532	15.46	89.8±10.9	87.2±5.8	84.3±13.0	5.9 | 0.13	0.9963
Bifenazate	149877-41-8	$C_{17}H_{20}N_2O_3$	[M+H]+	301.1547	198.0908	18.47	135.4±26.2	100.5±0.8	97.8±2.0	26 | 0.56	0.9843
Carbaryl	63-25-2	$C_{12}H_{11}NO_2$	[M+H]+	202.0863	145.0642	14.47	87.8±5.8	100.3±1.8	87.0±9.5	11 | 0.25	0.9955
Carbendazim	10605-21-7	$C_9H_9N_3O_2$	[M+H]+	192.0768	148.1115	6.76	88.7±20.1	157.2±2.2	142.1±18.9	0.7 | 0.02	0.9893
Clothianidin	210880-92-5	$C_6H_8ClN_5O_2S$	[M+H]+	250.0160	169.0537	9.99	94.5±9.8	93.2±0.9	88.2±10.5	11 | 0.25	0.9963
Diuron	330-54-1	$C_9H_{10}Cl_2N_2O$	[M+H]+	233.0243	159.9709	16.03	78.8±17.3	109.3±27.6	96.6±11.4	10 | 0.23	0.9957
Imazapyr	81334-34-1	$C_{13}H_{15}N_3O_3$	[M+H]+	262.1186	149.0344	10.01	87.5±8.9	112.2±4.1	103.6±18.1	4.8 | 0.10	0.9965
Imidacloprid	138261-41-3	$C_9H_{10}ClN_5O_2$	[M+H]+	256.0596	175.0970	9.97	87.4±19.6	102.8±28.7	90.7±6.5	1.1 | 0.02	0.9958
Mecoprop	93-65-2	$C_{10}H_{11}ClO_3$	[M-H]-	213.0324	141.0114	17.94	86.3±16.1	117.4±7.0	115.5±10.9	4.3 | 0.09	0.9978
Malathion	121-75-5	$C_{10}H_{19}O_6PS_2$	[M+H]+	331.0433	109.0644	17.89	93.4±10.5	101.5±5.1	99.5±11.9	5.0 | 0.11	0.9963

**Table S10.** SPE-LC-HRMS method performance for 133 OMPs confirmed in POCIS and grab samples (continued)											
Compound Name	CAS	Molecular Formula	Adduct	Exact Mass (*m/z*)	Diagnostic Fragment (*m/z*)	RT (min)	SPE Recovery (%)	Ion Suppression / Enhancement (%)	Matrix Factor (%)	LOQs (ng/L | ng/d)	R^2
Metalaxyl	57837-19-1	$C_{15}H_{21}NO_4$	[M+H]+	280.1543	220.1324	15.89	78.6±12.3	108.8±20.6	99.9±11.5	10 | 0.22	0.9970
Metolachlor	51218-45-2	$C_{15}H_{22}ClNO_2$	[M+H]+	284.1412	252.1139	19.10	86.8±10.1	96.2±5.8	90.0±11.5	5.6 | 0.12	0.9975
Monuron	150-68-5	$C_9H_{11}ClN_2O$	[M+H]+	199.0633	126.0099	13.22	94.3±11.5	100.3±3.3	93.3±12.3	5.4 | 0.12	0.9997
Prometon	1610-18-0	$C_{10}H_{19}N_5O$	[M+H]+	226.1662	184.1186	13.38	92.0±8.6	103.9±5.5	97.8±9.2	1.0 | 0.02	0.9988
Prometryn	7287-19-6	$C_{10}H_{19}N_5S$	[M+H]+	242.1434	200.0961	15.62	78.4±9.0	109.1±11.2	112.2±14.5	0.9 | 0.02	0.9960
Propazine	139-40-2	$C_9H_{16}ClN_5$	[M+H]+	230.1167	188.0691	17.07	85.4±20.9	136.5±32.2	121.1±17.2	4.1 | 0.09	0.9892
Simazine	122-34-9	$C_7H_{12}ClN_5$	[M+H]+	202.0854	132.0318	13.62	81.1±21.0	110.6±29.5	97.6±15.8	5.1 | 0.11	0.9988
Thiamethoxam	153719-23-4	$C_8H_{10}ClN_5O_3S$	[M+H]+	292.0266	211.0641	8.42	88.5±16.8	133.9±22.4	114.1±11.3	8.8 | 0.19	0.9981
Atrazine-2-hydroxy	2163-68-0	$C_8H_{15}N_5O$	[M+H]+	198.1349	156.0876	9.37	88.0±10.0	101.3±7.6	100.5±2.8	10 | 0.22	0.9954
Atrazine-desethyl	6190-65-4	$C_6H_{10}ClN_5$	[M+H]+	188.0698	146.0224	11.44	90.0±5.9	88.7±7.0	84.5±8.5	1.2 | 0.03	0.9914
Atrazine-desisopropyl	1007-28-9	$C_5H_8ClN_5$	[M+H]+	174.0541	132.0318	8.99	92.3±11.3	93.2±5.3	88.8±4.9	11 | 0.24	0.9954
Metolachlor Ethanesulfonic Acid	171118-09-5	$C_{15}H_{23}NO_5S$	[M+H]+	330.1370	298.1100	14.77	90.1±19.6	54.7±5.3	136.4±4.0	7.3 | 0.16	0.9805
Metolachlor Oxanilic Acid	152019-73-3	$C_{15}H_{21}NO_4$	[M+H]+	280.1543	248.1274	16.02	78.6±12.7	100.5±19.1	91.8±10.5	27 | 0.59	0.9983
1H-Benzotriazole	95-14-7	$C_6H_5N_3$	[M+H]+	120.0556	120.0556	9.12	73.8±9.2	66.5±5.4	66.4±5.4	15 | 0.33	0.9992
2-Hydroxybenzothiazole	934-34-9	C_7H_5NOS	[M+H]+	152.0165	124.0211	12.73	97.2±7.8	82.7±2.1	86.2±10.0	12 | 0.25	0.9984
5-Methyl-1H-benzotriazole	136-85-6	$C_7H_7N_3$	[M+H]+	134.0713	106.0647	11.82	86.8±8.1	82.7±5.3	84.2±8.4	12 | 0.26	0.9994
Benzophenone	119-61-9	$C_{13}H_{10}O$	[M+H]+	183.0804	105.0330	17.49	77.0±13.2	54.9±12.5	51.8±22.0	19 | 0.42	0.9868
Benzophenone-3 (Oxybenzone)	131-57-7	$C_{14}H_{12}O_3$	[M+H]+	229.0859	151.0384	19.23	76.5±5.1	105.9±4.3	97.6±10.5	5.1 | 0.11	0.9971
Benzothiazole	95-16-9	C_7H_5NS	[M+H]+	136.0216	122.3269	12.97	77.9±13.4	82.3±7.1	101.3±26.3	9.9 | 0.21	0.9853
N,N-Diethyl-3-methylbenzamide (DEET)	134-62-3	$C_{12}H_{17}NO$	[M+H]+	192.1383	119.0484	15.75	90.6±9.2	109.1±2.3	106.3±8.7	0.2 | 0.004	0.9988
Ethyl Butylacetylaminopropionate	52304-36-6	$C_{11}H_{21}NO_3$	[M+H]+	216.1594	170.1170	14.67	94.6±8.4	98.3±1.9	98.6±11.8	10 | 0.22	0.9974
Sucralose	56038-13-2	$C_{12}H_{19}Cl_3O_8$	[M+FA-H]-	441.0128	395.0071	9.24	69.7±14.7	112.8±2.2	97.0±19.6	10 | 0.22	0.9991
Triclosan	3380-34-5	$C_{12}H_7Cl_3O_2$	[M-H]-	286.9439	130.3132	21.94	82.7±16.8	118.2±7.3	78.1±15.5	13 | 0.28	0.9962
Galaxolidone	507442-49-1	$C_{18}H_{24}O_2$	[M+H]+	273.1849	240.1500	21.86	70.6±7.1	138.4±17.1	127.1±21.5	7.9 | 0.17	0.9963
Caprolactam	105-60-2	$C_6H_{11}NO$	[M+H]+	114.0913	96.0802	6.77	142.2±8.7	95.1±10.1	142.3±13.8	18 | 0.38	0.9858
1,3-Diphenylguanidine (DPG)	102-06-7	$C_{13}H_{13}N_3$	[M+H]+	212.1182	195.0911	9.69	39.5±3.1	234.5±9.6	114.9±5.9	0.9 | 0.02	0.9900
3-Cyclohexyl-1,1-dimethylurea (C-DMU)	31468-12-9	$C_9H_{18}N_2O$	[M+H]+	171.1492	89.0705	12.63	48.8±5.2	200.0±4.9	86.5±8.0	1.2 | 0.03	0.9998
Hexa(methoxymethyl)melamine (HMMM)	3089-11-0	$C_{15}H_{30}N_6O_6$	[M+H]+	391.2300	149.0227	14.66	39.7±6.0	324.6±55.6	145.4±18.9	0.7 | 0.01	0.9954
N-Cyclohexyl-N'-phenylurea (CPU)	886-59-9	$C_{13}H_{18}N_2O$	[M+H]+	219.1492	137.0703	16.08	42.2±2.0	227.4±8.7	100.6±16.3	1.0 | 0.02	0.9987
N-Ethyl-p-toluenesulfonamide	80-39-7	$C_9H_{13}NO_2S$	[M+H]+	200.0740	155.0156	13.37	39.1±3.3	181.9±4.0	76.2±6.0	1.3 | 0.03	0.9937
Triphenylphosphine Oxide	791-28-6	$C_{18}H_{15}OP$	[M+H]+	279.0933	219.0563	17.04	46.5±4.0	193.7±8.2	100.5±18.0	1.0 | 0.02	0.9944
Dextrorphan *	125-73-5	$C_{17}H_{23}NO$	[M+H]+	258.1852	199.1116	10.46	82.2±6.7	125.6±6.0	123.5±15.4	0.8 | 0.02	0.9959
4-Methylbenzophenone *	134-84-9	$C_{14}H_{12}O$	[M+H]+	197.0961	105.0329	18.83	38.5±1.5	238.7±15.5	93.9±13.0	0.1 | 0.002	0.9988
Diheptyl Phthalate *	3648-21-3	$C_{22}H_{34}O_4$	[M+H]+	363.2530	149.0225	25.89	81.6±0.9	299.4±10.7	123.2±10.5	41 | 0.88	0.9860
Di(propylene glycol) Dibenzoate *	27138-31-4	$C_{20}H_{22}O_5$	[M+H]+	343.1540	163.0745	20.85	38.4±2.2	309.2±28.5	109.7±0.9	0.9 | 0.02	0.9970
Hexadecyltrimethylammonium (Cetrimonium) *	112-02-7	$C_{19}H_{42}N^+$	[M]+	284.3312	203.0763	22.96	25.7±2.9	119.7±10.8	167.9±10.3	0.6 | 0.01	0.9885
Lauramidopropyl Betaine *	86438-79-1	$C_{19}H_{38}N_2O_3$	[M+H]+	343.2954	109.1011	21.08	111.5±8.9	78.3±1.4	85.0±4.1	0.1 | 0.003	0.9950
N-Ethyl-p-menthane-3-carboxamide *	39711-79-0	$C_{13}H_{25}NO$	[M+H]+	212.2009	170.1537	18.64	94.6±19.1	131.6±9.1	128.8±20.5	0.8 | 0.02	0.9955
Tributyl Citrate *	77-94-1	$C_{18}H_{32}O_7$	[M+H]+	361.2221	129.0177	21.86	84.7±8.5	86.1±5.9	118.2±7.3	0.1 | 0.002	0.9816
Tris(2-butoxyethyl) Phosphate (TBEP) *	78-51-3	$C_{18}H_{39}O_7P$	[M+H]+	399.2506	143.0098	22.04	64.8±11.4	163.3±19.0	94.3±10.7	0.1 | 0.002	0.9925
Daidzein *	486-66-8	$C_{15}H_{10}O_4$	[M+H]+	255.0652	199.0745	13.50	44.6±3.1	191.6±9.4	88.1±12.2	1.1 | 0.02	0.9935
Nobiletin *	478-01-3	$C_{21}H_{22}O_8$	[M+H]+	403.1387	327.0855	17.75	87.8±14.1	116.0±4.8	153.7±13.2	0.7 | 0.01	0.9873

"RT" = retention time; "SPE Recovery" = absolute SPE recovery; "LOQs" = limits of quantification determined based on the SPE of 500 mL stream water samples (ng/L) or the extraction of singlet POCIS disks assuming an average deployment period of 23 days (ng/d); "R^2" = coefficient of determination; "NA" = not available. OMPs with an asterisk ("*") denote those prioritized via nontarget screening and confirmed by reference standards.

**Table S11.** Summary statistics of quantifiable OMPs in POCIS								
Compound Name	Category[a]	Minimum (ng/d)	Mean (ng/d)	Median (ng/d)	Maximum (ng/d)	Sample-Specific Detection Frequency[b]	Site-Specific Detection Frequency[c]	Cluster[d]
Abacavir	PHAR	0.17	0.32	0.35	0.45	14.3%	15.0%	B
Aliskiren	PHAR	0.64	1.22	1.26	1.67	19.0%	20.0%	B
Amantadine	PHAR	0.08	0.45	0.33	1.27	31.0%	40.0%	C
Amitriptyline	PHAR	0.51	1.12	0.96	1.75	23.8%	25.0%	B
Atenolol	PHAR	0.69	1.26	1.18	2.21	14.3%	15.0%	B
Azithromycin	PHAR	0.91	3.42	3.27	5.98	14.3%	15.0%	A
Bisoprolol	PHAR	0.76	1.92	1.83	3.48	14.3%	15.0%	B
Bupropion	PHAR	0.28	0.53	0.49	1.20	26.2%	30.0%	C
Caffeine	PHAR	0.30	2.59	0.99	9.80	42.9%	70.0%	C
Carbamazepine	PHAR	0.79	2.30	1.50	14.16	61.9%	75.0%	C
Cetirizine	PHAR	0.44	4.44	1.83	24.35	59.5%	60.0%	C
Citalopram	PHAR	0.15	1.23	0.65	3.94	45.2%	45.0%	C
Clarithromycin	PHAR	0.39	1.06	1.11	1.78	14.3%	15.0%	A
Clindamycin	PHAR	0.49	1.11	1.02	1.72	14.3%	15.0%	A
Codeine	PHAR	0.71	1.23	1.23	1.99	28.6%	30.0%	B
Desvenlafaxine	PHAR	0.48	1.52	0.89	5.82	38.1%	55.0%	C
Dextromethorphan	PHAR	0.15	0.32	0.21	0.74	50.0%	50.0%	C
Diazepam	PHAR	0.66	1.35	1.13	2.49	23.8%	25.0%	B
Diltiazem	PHAR	0.19	0.90	0.30	3.49	38.1%	40.0%	C
Diphenhydramine	PHAR	0.08	1.12	0.44	4.97	50.0%	50.0%	C
Doxylamine	PHAR	1.03	2.25	1.76	3.76	19.0%	20.0%	B
Ephedrine	PHAR	0.37	0.91	0.93	1.42	19.0%	20.0%	B
Fexofenadine	PHAR	0.36	6.96	3.07	32.36	47.6%	55.0%	C
Flecainide	PHAR	0.18	0.73	0.29	2.56	50.0%	50.0%	C
Fluconazole	PHAR	0.60	1.23	1.12	1.89	19.0%	20.0%	B
Fluoxetine	PHAR	0.58	1.24	0.98	2.75	19.0%	20.0%	B
Gabapentin	PHAR	0.56	4.74	4.51	9.90	23.8%	25.0%	B
Gemfibrozil	PHAR	0.53	4.92	4.74	10.51	23.8%	25.0%	B
Griseofulvin	PHAR	0.65	1.23	0.82	3.05	66.7%	70.0%	B
Guaifenesin	PHAR	0.70	1.02	0.96	1.51	19.0%	20.0%	B
Irbesartan	PHAR	0.80	2.39	1.42	7.43	28.6%	50.0%	C
Labetalol	PHAR	1.09	1.80	1.91	2.39	14.3%	15.0%	B
Lamotrigine	PHAR	0.23	2.66	1.22	11.47	83.3%	85.0%	C
Levamisole	PHAR	1.03	1.62	1.50	2.36	14.3%	15.0%	A
Levorphanol	PHAR	0.57	1.51	1.57	2.53	28.6%	30.0%	B
Lidocaine	PHAR	0.63	1.68	1.01	12.34	100.0%	100.0%	C
Losartan	PHAR	0.31	1.45	0.65	5.03	33.3%	45.0%	C
Maprotiline	PHAR	0.78	1.39	1.36	2.29	23.8%	25.0%	B
Metaxalone	PHAR	0.31	0.87	0.85	1.63	26.2%	25.0%	B
Metformin	PHAR	1.18	1.87	1.77	2.96	14.3%	25.0%	C
Methocarbamol	PHAR	0.32	1.02	0.47	3.98	54.8%	55.0%	C
Metoprolol	PHAR	0.73	1.47	1.06	4.93	52.4%	60.0%	C
Molindone	PHAR	0.67	1.13	1.14	1.85	23.8%	25.0%	C

**Table S11.** Summary statistics of quantifiable OMPs in POCIS (continued)								
Compound Name	Category[a]	Minimum (ng/d)	Mean (ng/d)	Median (ng/d)	Maximum (ng/d)	Sample-Specific Detection Frequency[b]	Site-Specific Detection Frequency[c]	Cluster[d]
Mycophenolic Acid	PHAR	0.93	1.43	1.29	2.25	28.6%	30.0%	B
Naproxen	PHAR	1.67	3.27	2.56	7.69	21.4%	20.0%	C
Oxcarbazepine	PHAR	0.48	1.07	1.04	1.76	14.3%	15.0%	B
Phenytoin	PHAR	0.63	1.23	1.02	2.35	21.4%	15.0%	B
Prilocaine	PHAR	0.39	0.66	0.66	0.90	19.0%	20.0%	B
Propranolol	PHAR	0.51	1.90	1.30	4.49	23.8%	25.0%	C
Ranitidine	PHAR	1.08	1.80	1.78	2.59	23.8%	25.0%	B
Rimantadine	PHAR	0.58	1.51	1.59	2.18	23.8%	25.0%	B
Sertraline	PHAR	1.07	2.12	1.87	3.46	19.0%	20.0%	B
Sitagliptin	PHAR	1.00	1.71	1.68	2.37	23.8%	20.0%	B
Sotalol	PHAR	0.77	1.85	1.60	3.45	31.0%	30.0%	B
Sulfamethoxazole	PHAR	0.53	1.32	0.93	4.48	52.4%	60.0%	C
Sulfapyridine	PHAR	1.34	2.22	1.97	3.59	19.0%	20.0%	A
Telmisartan	PHAR	0.63	1.13	0.98	1.91	19.0%	20.0%	B
Trenbolone	PHAR	1.03	2.57	2.02	4.76	19.0%	20.0%	A
Trimethoprim	PHAR	0.13	0.74	0.46	2.51	33.3%	40.0%	C
Valsartan	PHAR	1.30	5.18	4.40	13.15	42.9%	45.0%	C
Venlafaxine	PHAR	0.25	0.92	0.73	3.96	59.5%	60.0%	C
Benzoylecgonine	PHAR TP	0.44	0.74	0.63	1.51	35.7%	45.0%	C
N4-Acetylsulfamethoxazole	PHAR TP	0.91	1.15	1.15	1.40	19.0%	20.0%	B
2,4-D	PEST	0.85	3.93	2.80	13.97	83.3%	90.0%	A
Abscisic Acid	PEST	0.68	3.33	2.96	9.17	100.0%	100.0%	A
Acetamiprid	PEST	0.57	0.84	0.84	1.08	14.3%	15.0%	A
Ametryn	PEST	0.80	2.68	3.07	4.36	19.0%	25.0%	A
Atrazine	PEST	2.02	11.48	8.69	58.95	100.0%	100.0%	A
Bifenazate	PEST	1.07	1.50	1.45	2.09	19.0%	20.0%	A
Carbaryl	PEST	0.16	0.46	0.28	1.89	47.6%	50.0%	A
Carbendazim	PEST	0.65	2.94	2.72	5.87	38.1%	40.0%	A
Clothianidin	PEST	0.41	1.03	0.64	3.42	57.1%	60.0%	A
Diuron	PEST	0.15	1.43	0.75	6.13	47.6%	50.0%	A
Imidacloprid	PEST	0.79	4.64	3.27	18.35	92.9%	95.0%	B
Malathion	PEST	0.97	1.51	1.64	1.90	14.3%	15.0%	A
Metalaxyl	PEST	0.45	9.51	2.73	71.82	69.0%	80.0%	A
Metolachlor	PEST	0.91	10.63	4.81	44.83	100.0%	100.0%	A
Prometon	PEST	0.30	0.99	0.45	3.58	83.3%	90.0%	A
Prometryn	PEST	1.26	2.59	1.74	7.38	19.0%	20.0%	A
Propazine	PEST	0.43	1.69	1.08	5.23	64.3%	65.0%	A
Simazine	PEST	0.33	1.51	0.62	11.70	57.1%	65.0%	A
Thiamethoxam	PEST	0.37	1.29	0.89	3.17	38.1%	45.0%	A
Atrazine-2-hydroxy	PEST TP	1.27	16.24	8.86	94.89	100.0%	100.0%	A
Atrazine-desethyl	PEST TP	0.86	2.52	1.54	11.95	100.0%	100.0%	A
Atrazine-desisopropyl	PEST TP	0.42	0.76	0.63	2.17	81.0%	100.0%	A
Metolachlor Ethanesulfonic Acid (Metolachlor ESA)	PEST TP	0.49	216.45	91.47	1368.27	100.0%	100.0%	A

**Table S11.** Summary statistics of quantifiable OMPs in POCIS (continued)								
Compound Name	Category[a]	Minimum (ng/d)	Mean (ng/d)	Median (ng/d)	Maximum (ng/d)	Sample-Specific Detection Frequency[b]	Site-Specific Detection Frequency[c]	Cluster[d]
Metolachlor Oxanilic Acid (Metolachlor OA)	PEST TP	0.21	5.87	1.98	55.30	100.0%	100.0%	A
2-Hydroxybenzothiazole	PCHI	0.61	0.98	0.87	1.56	9.5%	10.0%	B
Benzothiazole	PCHI	2.83	18.74	18.61	49.65	97.6%	100.0%	C
Benzotriazole	PCHI	1.56	4.44	2.64	14.89	45.2%	50.0%	C
Methyl-1H-benzotriazole	PCHI	1.50	3.46	2.39	15.28	95.2%	95.0%	C
Ethyl Butylacetylaminopropionate	PEST	0.54	1.06	0.96	1.99	19.0%	20.0%	B
DEET	PCHI	0.66	4.46	2.50	22.39	95.2%	100.0%	B
Sucralose	PCHI	0.49	19.61	5.03	138.98	97.6%	100.0%	C
Galaxolidone	PCHI TP	1.44	17.67	10.39	97.00	97.6%	100.0%	C
3-Cyclohexyl-1,1-dimethylurea	PCHI	0.96	1.79	1.72	2.76	19.0%	20.0%	B
Hexa(methoxymethyl)melamine (HMMM)	PCHI	0.59	1.96	2.06	3.36	21.4%	25.0%	B
N-Cyclohexyl-N'-phenylurea	PCHI	0.09	0.91	0.37	4.32	64.3%	65.0%	B
N-Ethyl-p-toluenesulfonamide	PCHI	1.05	2.20	2.49	2.97	19.0%	20.0%	B
Triphenylphosphine Oxide	PCHI	1.08	2.24	2.30	2.99	14.3%	15.0%	B
Dextrorphan *	PHAR	0.10	0.80	0.23	4.89	100.0%	100.0%	C
4-Methylbenzophenone *	PCHI	0.11	1.15	0.18	9.88	100.0%	100.0%	B
Diheptyl Phthalate *	PCHI	0.12	1.33	0.79	5.68	100.0%	100.0%	C
Di(propylene glycol) Dibenzoate *	PCHI	0.10	0.96	0.37	7.06	100.0%	100.0%	B
Hexadecyltrimethylammonium (Cetrimonium) *	PCHI	0.10	0.45	0.19	2.70	100.0%	100.0%	B
Lauramidopropyl Betaine *	PCHI	0.11	0.39	0.20	2.30	100.0%	100.0%	B
N-Ethyl-p-menthane-3-carboxamide *	PCHI	0.10	1.44	0.26	10.99	100.0%	100.0%	C
Tributyl Citrate *	PCHI	0.18	0.71	0.54	2.82	100.0%	100.0%	C
Tris(2-butoxyethyl) Phosphate (TBEP) *	PCHI	2.67	17.77	8.78	129.96	100.0%	100.0%	C
Daidzein *	Phytochemical	0.13	1.58	0.35	11.82	100.0%	100.0%	A
Nobiletin *	Phytochemical	0.11	0.64	0.33	3.80	100.0%	100.0%	A

[a] "PHAR" = pharmaceutical, "PEST" = pesticide, "PCHI" = personal care, household and industrial chemical, "TP" = transformation product; [b] Defined as the detection frequency in POCIS; [c] Defined as the detection frequency at the 20 stream sites; [d] Assigned based on Figures 2 and 3 in the *Main Text*. OMPs with an asterisk ("*") denote those prioritized via nontarget screening and confirmed by reference standards.

Table S12. Summary statistics of quantifiable OMPs in grab samples

Compound Name	Category[a]	Minimum (ng/L)	Mean (ng/L)	Median (ng/L)	Maximum (ng/L)	Sample-Specific Detection Frequency[b]	Site-Specific Detection Frequency[c]	Cluster[d]
Acetaminophen	PHAR	87	88	88	89	1.1%	5.0%	C
Amantadine	PHAR	5	23	17	69	26.7%	45.0%	C
Azelaic Acid	PHAR	75	82	82	88	1.1%	5.0%	C
Bamethan	PHAR	24	196	92	780	8.9%	40.0%	C
Bupropion	PHAR	9	17	14	35	27.8%	60.0%	C
Caffeine	PHAR	11	180	128	676	38.9%	70.0%	C
Carbamazepine	PHAR	11	24	14	110	27.8%	55.0%	C
Cetirizine	PHAR	16	73	32	338	36.7%	45.0%	C
Citalopram	PHAR	6	11	9	23	11.1%	40.0%	C
Desipramine	PHAR	20	22	22	23	1.1%	5.0%	C
Desvenlafaxine	PHAR	10	25	14	72	16.7%	40.0%	C
Dexpanthenol	PHAR	11	27	19	93	10.0%	35.0%	C
Diltiazem	PHAR	10	13	13	19	6.7%	25.0%	C
Diphenhydramine	PHAR	11	16	14	27	7.8%	30.0%	C
Dopamine	PHAR	161	170	170	180	1.1%	5.0%	C
Fexofenadine	PHAR	11	87	43	419	37.8%	50.0%	C
Flecainide	PHAR	11	13	12	15	4.4%	15.0%	C
Fluconazole	PHAR	25	35	27	60	4.4%	10.0%	B
Gabapentin	PHAR	23	97	48	367	21.1%	35.0%	B
Gemfibrozil	PHAR	49	57	58	63	3.3%	5.0%	B
Irbesartan	PHAR	20	76	40	259	40.0%	55.0%	C
Lamotrigine	PHAR	10	57	27	271	73.3%	90.0%	C
Levetiracetam	PHAR	24	88	71	238	8.9%	40.0%	C
Lidocaine	PHAR	7	17	9	117	67.8%	100.0%	C
Losartan	PHAR	10	48	20	131	20.0%	30.0%	C
Metformin	PHAR	48	160	95	1439	48.9%	80.0%	C
Methocarbamol	PHAR	23	36	32	58	5.6%	10.0%	C
Metoprolol	PHAR	10	22	16	73	55.6%	90.0%	C
Nadolol	PHAR	29	243	43	2636	22.2%	50.0%	C
Naproxen	PHAR	50	117	122	262	13.3%	55.0%	C
Oxcarbazepine	PHAR	15	16	16	19	5.6%	10.0%	B
Propranolol	PHAR	15	16	16	16	2.2%	10.0%	C
Protriptyline	PHAR	7	34	18	145	21.1%	55.0%	C
Sertraline	PHAR	18	28	25	47	5.6%	25.0%	B
Sulfamethoxazole	PHAR	24	51	34	161	32.2%	35.0%	C
Sulfapyridine	PHAR	19	80	49	181	7.8%	10.0%	A
Trimethoprim	PHAR	2	9	5	41	31.1%	75.0%	C
Venlafaxine	PHAR	10	24	16	74	32.2%	55.0%	C
10,11-Dihydro-10-hydroxy Carbamazepine	PHAR TP	15	24	19	47	14.4%	20.0%	C
2-Ethyl-2-phenylmalonamide (PEMA)	PHAR TP	12	25	21	44	10.0%	20.0%	C
Benzoylecgonine	PHAR TP	18	30	25	49	17.8%	35.0%	C
Hydroxbupropion	PHAR TP	28	30	29	35	4.4%	10.0%	C
N4-Acetylsulfamethoxazole	PHAR TP	4	8	8	12	2.2%	10.0%	B

Table S12. Summary statistics of quantifiable OMPs in grab samples (continued)								
Compound Name	Category[a]	Minimum (ng/L)	Mean (ng/L)	Median (ng/L)	Maximum (ng/L)	Sample-Specific Detection Frequency[b]	Site-Specific Detection Frequency[c]	Cluster[d]
Ritalinic Acid	PHAR TP	28	29	29	29	1.1%	5.0%	C
(4-Chloro-2-methylphenoxy)acetic Acid (MCPA)	PEST	9	23	22	38	2.2%	10.0%	A
2,4-D	PEST	14	177	136	631	47.8%	90.0%	A
Abscisic Acid	PEST	21	123	117	264	13.3%	30.0%	A
Ametryn	PEST	30	160	140	341	4.4%	20.0%	A
Atrazine	PEST	16	81	58	496	100.0%	100.0%	A
Carbaryl	PEST	19	20	20	20	1.1%	5.0%	A
Carbendazim	PEST	11	58	16	150	3.3%	10.0%	A
Clothianidin	PEST	19	27	24	51	7.8%	25.0%	A
Diuron	PEST	10	15	16	19	7.8%	15.0%	A
Imazapyr	PEST	14	23	15	163	26.7%	50.0%	B
Imidacloprid	PEST	11	45	40	128	21.1%	50.0%	B
Mecoprop	PEST	13	22	18	55	21.1%	50.0%	B
Metalaxyl	PEST	12	97	45	429	34.4%	55.0%	A
Metolachlor	PEST	9	65	29	330	100.0%	100.0%	A
Monuron	PEST	129	130	130	131	1.1%	5.0%	A
Prometon	PEST	6	12	11	29	33.3%	45.0%	A
Prometryn	PEST	12	89	65	208	4.4%	5.0%	A
Propazine	PEST	17	30	25	56	8.9%	35.0%	A
Simazine	PEST	17	51	28	176	12.2%	25.0%	A
Thiamethoxam	PEST	13	27	21	49	5.6%	25.0%	A
Atrazine-2-hydroxy	PEST TP	26	183	111	1342	100.0%	100.0%	A
Atrazine-desethyl	PEST TP	11	37	24	178	100.0%	100.0%	A
Atrazine-desisopropyl	PEST TP	21	37	28	109	34.4%	60.0%	A
Metolachlor Ethanesulfonic Acid (Metolachlor ESA)	PEST TP	44	1406	750	8265	73.3%	95.0%	A
Metolachlor Oxanilic Acid (Metolachlor OA)	PEST TP	41	218	71	1340	60.0%	70.0%	A
2-Hydroxybenzothiazole	PCHI	56	60	60	63	1.1%	5.0%	B
Benzothiazole	PCHI	31	220	212	540	85.6%	100.0%	C
Benzotriazole	PCHI	58	169	169	393	22.2%	30.0%	C
Methyl-1H-benzotriazole	PCHI	25	71	50	263	63.3%	90.0%	C
Benzophenone	PCHI	17	123	79	663	81.1%	100.0%	C
Benzophenone-3 (Oxybenzone)	PCHI	22	33	25	127	18.9%	70.0%	C
Ethyl Butylacetylaminopropionate	PEST	11	50	16	144	3.3%	15.0%	B
DEET	PCHI	3	43	27	174	52.2%	95.0%	B
Sucralose	PCHI	54	660	230	4325	74.4%	80.0%	C
Triclosan	PCHI	14	36	28	65	5.6%	25.0%	C
Galaxolidone	PCHI TP	12	148	88	907	100.0%	100.0%	C
Caprolactam	PCHI	39	103	85	228	5.6%	25.0%	B
1,3-Diphenylguanidine	PCHI	7	29	17	86	6.7%	30.0%	B
3-Cyclohexyl-1,1-dimethylurea	PCHI	13	30	28	53	5.6%	25.0%	B
Hexa(methoxymethyl)melamine (HMMM)	PCHI	12	27	20	63	23.3%	65.0%	B
N-Cyclohexyl-N'-phenylurea	PCHI	12	24	18	49	4.4%	20.0%	B
Triphenylphosphine Oxide	PCHI	14	16	16	19	1.1%	5.0%	B

Table S12. Summary statistics of quantifiable OMPs in grab samples (continued)								
Compound Name	Category[a]	Minimum (ng/L)	Mean (ng/L)	Median (ng/L)	Maximum (ng/L)	Sample-Specific Detection Frequency[b]	Site-Specific Detection Frequency[c]	Cluster[d]
Tris(2-butoxyethyl) Phosphate (TBEP) *	PCHI	31	207	100	1527	81.1%	100.0%	C

[a] "PHAR" = pharmaceutical, "PEST" = pesticide, "PCHI" = personal care, household and industrial chemical, "TP" = transformation product; [b] Defined as the detection frequency in POCIS; [c] Defined as the detection frequency at the 20 stream sites; [d] Assigned based on Figures 2 and 3 in the *Main Text*. OMPs with an asterisk ("*") denote those prioritized via nontarget screening and confirmed by reference standards.

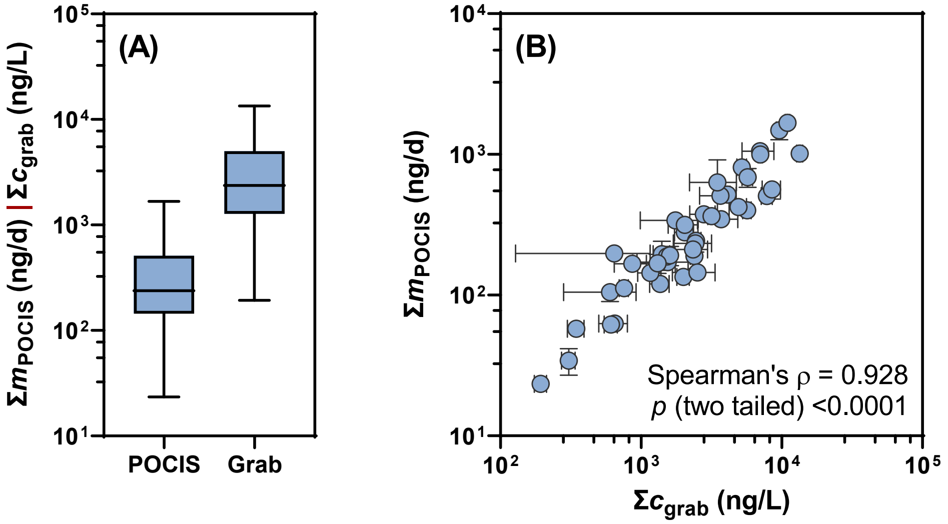

Figure S3. Comparison of OMP levels in POCIS and grab samples: **(A)** Boxplots of the cumulative daily average mass of OMPs in POCIS (Σm_{POCIS} in ng/d) and the cumulative concentration of OMPs in grab samples (Σc_{grab} in ng/L) measured at the 20 stream sites . The whiskers and centerline mark the range (minimum to maximum) and median of Σm_{POCIS} or Σc_{grab}, respectively. **(B)** Spearman's correlation between the cumulative daily average mass of OMPs (Σm_{POCIS}) in POCIS and the cumulative concentration of OMPs (Σc_{grab}) in grab samples across the stream sites over two sampling seasons.

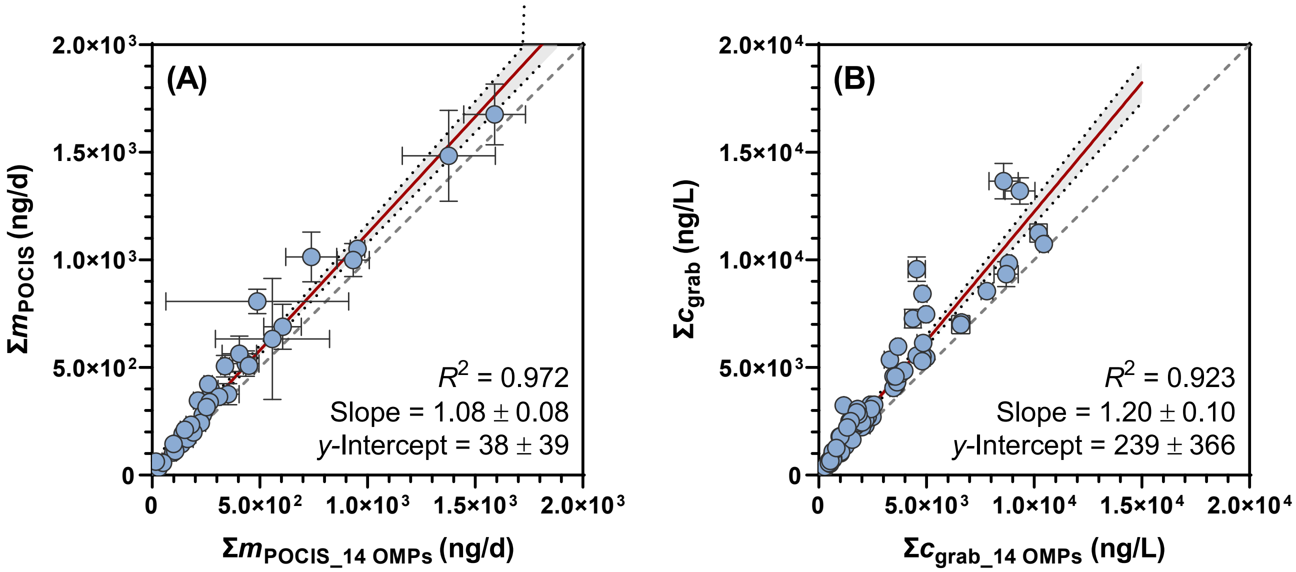

Figure S4. Fourteen OMPs (i.e., 2,4-D, atrazine, atrazine-2-hydroxy, atrazine-desethyl, benzothiazole, DEET, galaxolidone, lamotrigine, lidocaine, methyl-1H-benzotriazole, metolachlor, metolachlor ethanesulfonic acid, metolachlor oxanilic acid, sucralose) as quantitative indicators for OMP levels at the 20 stream sites: **(A)** Linear correlation between the cumulative daily average mass of 14 OMPs (Σ$m_{POCIS_14\ OMPs}$) and the cumulative daily average mass of all OMPs (Σm_{POCIS}) detected in POCIS. Error bars on individual data points represent the standard deviations from triplicate analyses of POCIS. The red solid line represents the slope derived from the linear least squares regression analysis of Σm_{POCIS} versus Σ$m_{POCIS_14\ OMPs}$. The black dotted lines bracket the 95% confidence interval for the slope. The grey dashed line represents the line of identity. **(B)** Linear correlation between the cumulative concentration of 14 OMPs (Σ$c_{grab_14\ OMPs}$) and the cumulative concentration of all OMPs detected in grab samples (Σc_{grab}). Error bars on individual data points represent the standard deviations from duplicate analyses of grab samples. The red solid line represents the slope derived from the linear least squares regression analysis of Σc_{grab} versus Σ$c_{grab_14\ OMPs}$. The black dotted lines bracket the 95% confidence interval for the slope. The grey dashed line represents the line of identity.

S5. Watershed attributes as predictors for OMP levels in streams

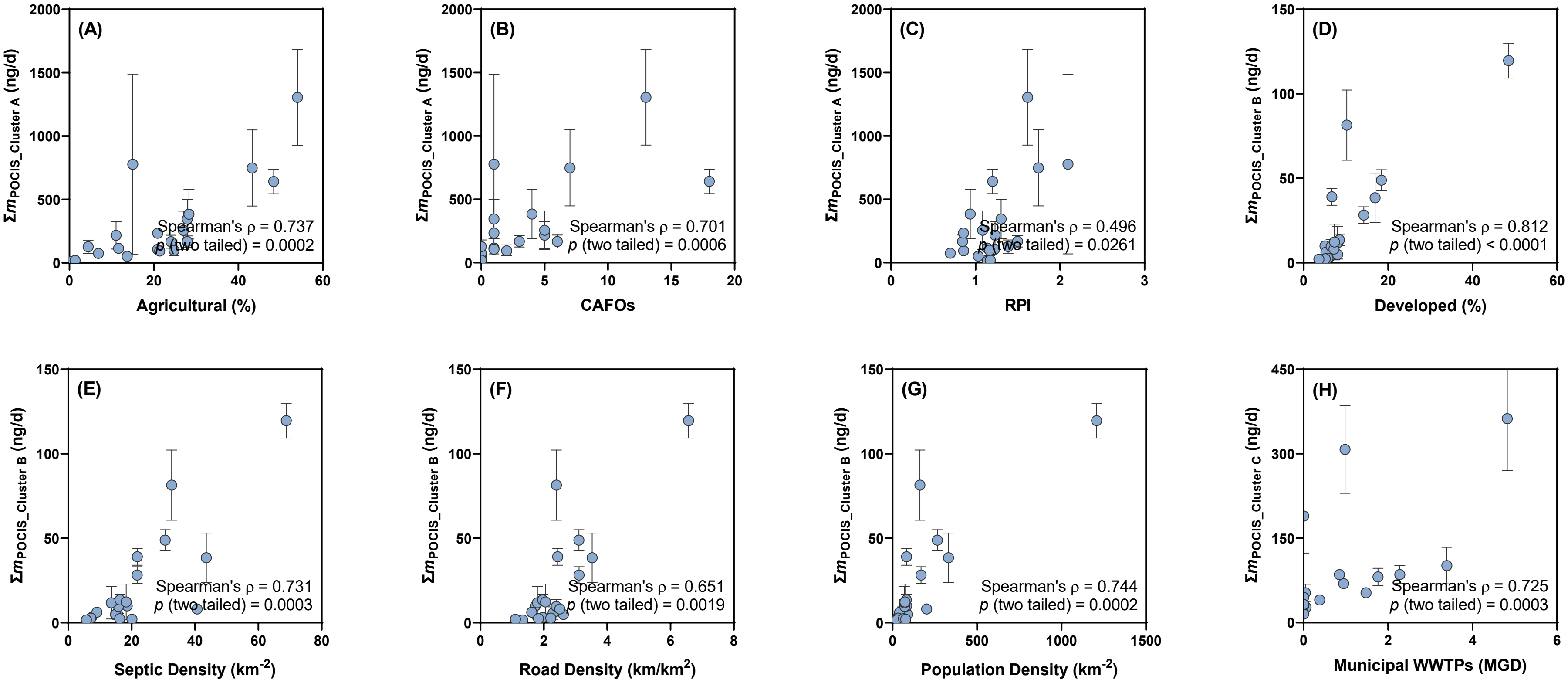

Figure S5a. Watershed attributes as predictors for the levels of OMP clusters measured at the 20 stream sites: **(A)** Spearman's correlation between the site-specific cumulative daily average mass of cluster A OMPs in POCIS ($\Sigma m_{\text{POCIS\_Cluster A}}$ (ng/d)) and the percent watershed agricultural land usage (Agricultural). **(B)** Spearman's correlation between $\Sigma m_{\text{POCIS\_Cluster A}}$ (ng/d) and the number of concentrated animal feeding operations (CAFOs) within the watershed. **(C)** Spearman's correlation between $\Sigma m_{\text{POCIS\_Cluster A}}$ (ng/d) and the runoff propensity index (RPI) of the watershed. **(D)** Spearman's correlation between the site-specific cumulative daily average mass of cluster B OMPs in POCIS ($\Sigma m_{\text{POCIS\_Cluster B}}$ (ng/d)) and the percent watershed developed land usage (Developed). **(E)** Spearman's correlation between $\Sigma m_{\text{POCIS\_Cluster B}}$ (ng/d) and the number of septic systems per km^2 of the watershed (Septic Density). **(F)** Spearman's correlation between $\Sigma m_{\text{POCIS\_Cluster B}}$ (ng/d) and the road length per km^2 in the watershed (Road Density). **(G)** Spearman's correlation between $\Sigma m_{\text{POCIS\_Cluster B}}$ (ng/d) and the population per km^2 of a watershed (Population Density). **(H)** Spearman's correlation between the site-specific cumulative daily average mass of cluster C OMPs in POCIS ($\Sigma m_{\text{POCIS\_Cluster C}}$ (ng/d)) and the summed capacity (in MGD) of municipal wastewater treatment plants within the watershed (Municipal WWTPs). Error bars represent the standard deviations of $\Sigma m_{\text{POCIS\_Cluster A}}$, $\Sigma m_{\text{POCIS\_Cluster B}}$, and $\Sigma m_{\text{POCIS\_Cluster C}}$. Note that the number of combined sewer overflow outfalls (CSOs) within the watershed and the summed capacity (in MGD) of industrial wastewater treatment plants within the watershed were excluded from correlation analysis.

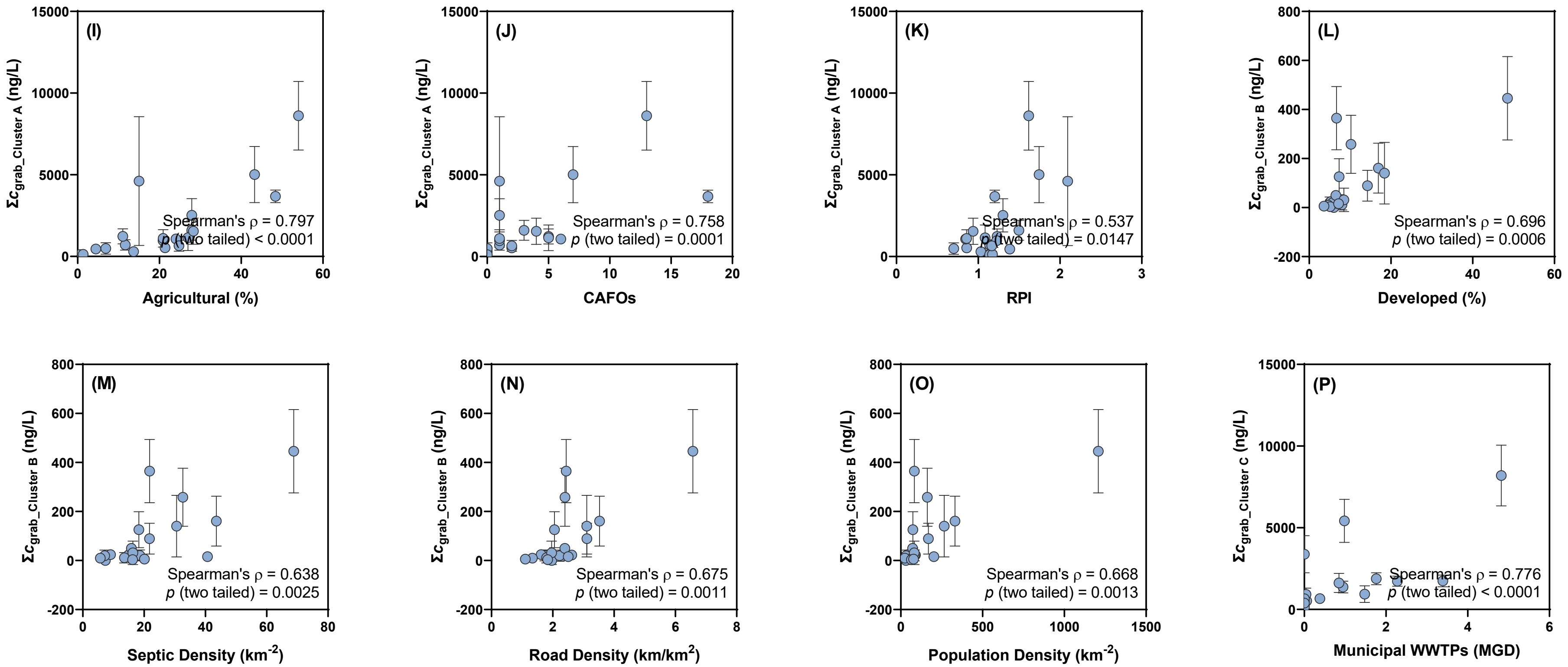

Figure S5b. Watershed attributes as predictors for the levels of OMP clusters measured at the 20 stream sites: **(I)** Spearman's correlation between the site-specific cumulative concentration of cluster A OMPs in grab samples ($\Sigma c_{\text{grab\_Cluster A}}$ (ng/L)) and the percent watershed agricultural land usage (Agricultural). **(J)** Spearman's correlation between $\Sigma c_{\text{grab\_Cluster A}}$ (ng/L) and the number of concentrated animal feeding operations (CAFOs) within the watershed. **(K)** Spearman's correlation between $\Sigma c_{\text{grab\_Cluster A}}$ (ng/L) and the runoff propensity index (RPI) of the watershed. **(L)** Spearman's correlation between the site-specific cumulative concentration of cluster B OMPs in grab samples ($\Sigma c_{\text{grab\_Cluster B}}$ (ng/L)) and the percent watershed developed land usage (Developed). **(M)** Spearman's correlation between $\Sigma c_{\text{grab\_Cluster B}}$ (ng/L) and the number of septic systems per km^2 of the watershed (Septic Density). **(N)** Spearman's correlation between $\Sigma c_{\text{grab\_Cluster B}}$ (ng/L) and the road length per km^2 in the watershed (Road Density). **(O)** Spearman's correlation between $\Sigma c_{\text{grab\_Cluster B}}$ (ng/L) and the population per km^2 of a watershed (Population Density). **(P)** Spearman's correlation between the site-specific cumulative concentration of cluster C OMPs in grab samples ($\Sigma c_{\text{grab\_Cluster C}}$ (ng/L)) and the summed capacity (in MGD) of municipal wastewater treatment plants within the watershed (Municipal WWTPs). Error bars represent the standard deviations of $\Sigma c_{\text{grab\_Cluster A}}$, $\Sigma c_{\text{grab\_Cluster B}}$, and $\Sigma c_{\text{grab\_Cluster C}}$. Note that the number of combined sewer overflow outfalls (CSOs) within the watershed and the summed capacity (in MGD) of industrial wastewater treatment plants within the watershed were excluded from correlation analysis.

Table S13. Spearman's correlation matrix between the z-score standardized m_{POCIS} or c_{grab} of 122 OMPs and watershed attributes								
Compound Name	Agricultural	CFAOs	RPI	Developed	SepticDensity	RoadDensity	PopulationDensity	MunicipalWWTPs
Acetaminophen	0.22	-0.10	0.18	0.22	0.26	0.10	0.18	0.40
Abacavir	-0.01	-0.12	-0.33	0.53	0.49	0.48	0.48	0.12
Aliskiren	-0.04	-0.18	-0.24	0.45	0.54	0.50	0.47	0.21
Amantadine	0.32	-0.02	0.05	0.19	-0.03	-0.01	-0.13	0.67
Amitriptyline	-0.05	-0.23	-0.37	0.59	0.61	0.59	0.58	0.07
Atenolol	-0.07	-0.32	-0.09	0.35	0.51	0.39	0.39	0.19
Azithromycin	0.62	0.63	0.41	-0.39	-0.07	-0.21	-0.12	0.08
Azelaic Acid	0.22	-0.10	0.18	0.22	0.26	0.10	0.18	0.40
Bamethan	0.10	-0.05	-0.18	0.31	0.11	0.21	-0.01	0.61
Bisoprolol	-0.07	-0.32	-0.09	0.35	0.51	0.39	0.39	0.19
Bupropion	0.29	-0.07	-0.08	0.27	0.11	0.19	0.04	0.60
Caffeine	0.17	0.13	-0.10	0.29	0.15	0.35	0.09	0.51
Carbamazepine	0.15	-0.06	0.05	0.10	0.15	-0.11	-0.02	0.79
Cetirizine	0.41	0.19	0.15	0.05	-0.12	-0.07	-0.25	0.86
Citalopram	0.22	-0.16	-0.35	0.45	0.14	0.30	0.15	0.45
Clarithromycin	0.62	0.63	0.41	-0.39	-0.07	-0.21	-0.12	0.08
Clindamycin	0.62	0.63	0.41	-0.39	-0.07	-0.21	-0.12	0.08
Codeine	0.04	-0.17	-0.44	0.64	0.59	0.53	0.58	0.20
Desipramine	0.22	-0.10	0.18	0.22	0.26	0.10	0.18	0.40
Desvenlafaxine	0.31	-0.01	0.03	0.16	0.08	0.05	-0.04	0.72
Dexpanthenol	0.09	-0.15	-0.35	0.47	0.28	0.28	0.20	0.46
Dextromethorphan	0.15	-0.01	-0.29	0.39	0.24	0.25	0.11	0.70
Diazepam	-0.05	-0.23	-0.37	0.59	0.61	0.59	0.58	0.07
Diltiazem	0.17	-0.11	-0.14	0.23	0.06	0.20	-0.02	0.51
Diphenhydramine	0.31	0.03	-0.13	0.28	0.22	0.30	0.11	0.53
Dopamine	-0.06	-0.10	0.10	-0.06	0.18	0.14	0.06	0.19
Doxylamine	-0.04	-0.18	-0.24	0.45	0.54	0.50	0.47	0.21
Ephedrine	-0.03	-0.16	-0.26	0.46	0.54	0.51	0.48	0.20
Fexofenadine	0.28	0.15	-0.13	0.27	0.08	0.12	-0.04	0.85
Flecainide	0.13	-0.04	-0.16	0.28	0.10	0.15	-0.05	0.75
Fluconazole	0.00	-0.16	-0.18	0.42	0.52	0.47	0.44	0.26
Fluoxetine	-0.04	-0.14	-0.29	0.47	0.53	0.52	0.48	0.18
Gabapentin	0.00	-0.24	-0.39	0.51	0.50	0.56	0.39	0.28
Gemfibrozil	-0.14	-0.37	-0.41	0.50	0.50	0.56	0.42	0.14
Griseofulvin	-0.06	-0.18	-0.44	0.71	0.48	0.52	0.62	0.10
Guaifenesin	-0.03	-0.16	-0.26	0.46	0.54	0.51	0.48	0.20
Irbesartan	0.16	-0.13	-0.23	0.39	0.26	0.27	0.18	0.62
Labetalol	-0.07	-0.32	-0.09	0.35	0.51	0.39	0.39	0.19
Lamotrigine	0.23	0.07	-0.15	0.41	0.31	0.26	0.17	0.75
Levetiracetam	0.23	-0.06	-0.42	0.54	0.25	0.19	0.20	0.64
Levamisole	0.62	0.63	0.41	-0.39	-0.07	-0.21	-0.12	0.08
Levorphanol	0.07	-0.17	-0.45	0.65	0.58	0.50	0.57	0.22
Lidocaine	0.24	-0.04	-0.11	0.34	0.18	0.24	0.06	0.59
Losartan	0.23	-0.03	-0.28	0.40	0.31	0.29	0.19	0.65

Table S13. Spearman's correlation matrix between the z-score standardized m_{POCIS} or c_{grab} of 122 OMPs and watershed attributes (continued)								
Compound Name	Agricultural	CFAOs	RPI	Developed	SepticDensity	RoadDensity	PopulationDensity	MunicipalWWTPs
Maprotiline	0.03	-0.28	-0.32	0.56	0.55	0.42	0.51	0.19
Metaxalone	-0.03	-0.32	-0.24	0.51	0.56	0.45	0.47	0.09
Metformin	0.15	-0.02	0.07	0.22	0.08	0.20	-0.07	0.49
Methocarbamol	0.09	-0.08	-0.26	0.38	0.25	0.27	0.11	0.72
Metoprolol	0.08	-0.15	-0.15	0.40	0.12	0.30	0.10	0.46
Nadolol	0.01	0.00	-0.18	0.30	-0.04	0.24	-0.06	0.49
Molindone	-0.05	-0.23	-0.37	0.59	0.61	0.59	0.58	0.07
Mycophenolic Acid	0.04	-0.17	-0.42	0.62	0.59	0.53	0.57	0.22
Naproxen	-0.02	-0.15	-0.43	0.60	0.29	0.35	0.26	0.46
Oxcarbazepine	-0.03	-0.29	-0.03	0.32	0.49	0.36	0.36	0.25
Phenytoin	-0.02	-0.28	-0.09	0.36	0.48	0.34	0.34	0.23
Prilocaine	0.05	-0.23	-0.18	0.41	0.46	0.30	0.38	0.34
Propranolol	0.08	-0.20	-0.29	0.40	0.36	0.33	0.26	0.50
Protriptyline	0.20	-0.11	-0.35	0.47	0.13	0.18	0.10	0.51
Ranitidine	-0.05	-0.23	-0.37	0.59	0.61	0.59	0.58	0.07
Rimantadine	-0.04	-0.21	-0.39	0.60	0.61	0.60	0.59	0.07
Sertraline	0.16	-0.02	-0.48	0.61	0.49	0.60	0.56	0.23
Sitagliptin	-0.05	-0.23	-0.37	0.59	0.61	0.59	0.58	0.07
Sotalol	0.07	-0.25	-0.32	0.57	0.54	0.38	0.47	0.21
Sulfamethoxazole	0.18	0.06	-0.14	0.32	0.10	0.14	-0.04	0.83
Sulfapyridine	0.54	0.30	0.23	-0.20	0.03	-0.09	-0.12	0.51
Telmisartan	-0.03	-0.16	-0.26	0.46	0.54	0.51	0.48	0.20
Trenbolone	0.70	0.65	0.28	-0.28	-0.07	-0.26	-0.10	0.24
Trimethoprim	0.10	-0.15	-0.18	0.35	0.05	0.28	0.00	0.51
Valsartan	0.17	-0.04	-0.16	0.33	0.24	0.15	0.15	0.52
Venlafaxine	0.38	0.18	0.06	0.15	0.03	0.00	-0.07	0.83
10,11-Dihydro-10-hydroxy Carbamazepine	0.26	-0.03	0.09	0.06	0.12	0.14	-0.05	0.61
2-Ethyl-2-phenylmalonamide	0.07	-0.19	0.14	0.06	-0.06	-0.11	-0.15	0.53
Benzoylecgonine	0.16	-0.15	-0.43	0.53	0.37	0.35	0.28	0.55
Hydroxybupropion	0.03	-0.31	0.07	0.10	0.19	0.15	0.02	0.46
N4-Acetylsulfamethoxazole	-0.04	-0.32	-0.16	0.45	0.56	0.45	0.48	0.13
Ritalinic Acid	0.22	-0.10	0.18	0.22	0.26	0.10	0.18	0.40
(4-Chloro-2-methylphenoxy)acetic Acid	0.49	0.47	0.46	-0.40	0.00	-0.31	-0.07	-0.16
2,4-D	0.82	0.67	0.14	0.01	-0.24	-0.09	-0.09	0.14
Abscisic Acid	0.68	0.51	0.47	-0.24	-0.42	-0.33	-0.52	0.47
Ametryn	0.62	0.62	0.44	-0.41	-0.06	-0.24	-0.11	0.03
Acetamiprid	0.65	0.59	0.49	-0.41	-0.36	-0.35	-0.28	0.00
Atrazine	0.46	0.50	0.64	-0.38	-0.22	-0.36	-0.30	0.04
Bifenazate	0.70	0.65	0.29	-0.29	-0.07	-0.27	-0.11	0.21
Carbaryl	0.56	0.36	0.14	0.06	0.16	-0.03	0.17	0.29
Carbendazim	0.64	0.41	0.20	0.09	0.11	-0.12	0.10	0.33
Clothianidin	0.70	0.45	0.43	-0.06	-0.07	-0.22	-0.13	0.33
Diuron	0.48	0.17	0.43	-0.15	0.07	-0.08	-0.11	0.15
Imazapyr	-0.02	-0.22	-0.29	0.55	0.58	0.64	0.60	0.05

**Table S13.** Spearman's correlation matrix between the *z*-score standardized m_{POCIS} or c_{grab} of 122 OMPs and watershed attributes (continued)								
Compound Name	Agricultural	CFAOs	RPI	Developed	SepticDensity	RoadDensity	PopulationDensity	MunicipalWWTPs
Imidacloprid	0.22	0.01	-0.24	0.62	0.64	0.52	0.61	0.20
Malathion	0.61	0.62	0.44	-0.41	-0.05	-0.25	-0.10	0.05
Mecoprop	-0.09	-0.28	-0.43	0.70	0.59	0.67	0.54	0.06
Metalaxyl	0.76	0.64	0.48	-0.10	-0.15	-0.16	-0.15	0.43
Metolachlor	0.56	0.64	0.43	-0.10	-0.20	-0.04	-0.09	0.03
Monuron	0.38	0.34	0.30	-0.26	-0.06	-0.18	-0.18	-0.23
Prometon	0.81	0.70	0.15	0.10	-0.05	-0.05	0.08	0.22
Prometryn	0.49	0.41	0.44	-0.34	-0.13	-0.47	-0.15	0.06
Propazine	0.56	0.57	0.46	-0.25	-0.16	-0.25	-0.20	0.13
Simazine	0.67	0.53	0.41	-0.26	-0.34	-0.46	-0.38	0.55
Thiamethoxam	0.60	0.39	0.30	-0.03	-0.08	-0.21	-0.08	0.15
Atrazine-2-hydroxy	0.82	0.70	0.51	-0.14	-0.16	-0.17	-0.15	0.42
Atrazine-desethyl	0.72	0.71	0.58	-0.25	-0.29	-0.30	-0.32	0.32
Atrazine-desisopropyl	0.49	0.35	0.47	-0.14	-0.09	-0.01	-0.23	0.17
Metolachlor Ethanesulfonic Acid	0.76	0.69	0.51	-0.17	-0.28	-0.29	-0.28	0.36
Metolachlor Oxanilic Acid	0.81	0.65	0.45	-0.07	-0.09	-0.17	-0.11	0.41
Benzotriazole	0.15	-0.07	-0.45	0.61	0.31	0.26	0.25	0.53
2-Hydroxybenzothiazole	-0.05	-0.30	-0.16	0.44	0.47	0.36	0.41	0.10
Methyl-1H-benzotriazole	0.11	-0.01	-0.17	0.42	0.15	0.32	0.09	0.55
Benzophenone	0.37	0.30	-0.16	0.39	0.12	0.15	0.07	0.93
Oxybenzone	0.06	-0.08	-0.10	0.32	0.00	0.05	-0.06	0.53
Benzothiazole	0.43	0.32	0.08	0.17	-0.22	-0.07	-0.23	0.84
DEET	0.17	-0.04	-0.23	0.57	0.65	0.55	0.69	0.15
Ethyl Butylacetylaminopropionate	-0.19	-0.34	-0.16	0.47	0.64	0.54	0.54	0.01
Sucralose	0.27	0.11	-0.15	0.45	0.31	0.29	0.20	0.73
Triclosan	0.17	-0.21	-0.16	0.32	0.26	0.16	0.17	0.50
Galaxolidone	0.23	-0.17	-0.12	0.34	0.25	0.18	0.12	0.47
Caprolactam	-0.12	-0.11	-0.68	0.75	0.57	0.61	0.66	-0.21
1,3-Diphenylguanidine	-0.20	-0.33	-0.52	0.72	0.68	0.75	0.66	-0.07
3-Cyclohexyl-1,1-dimethylurea	-0.15	-0.27	-0.40	0.70	0.74	0.72	0.72	-0.07
N-Ethyl-p-toluenesulfonamide	-0.18	-0.34	-0.15	0.49	0.64	0.54	0.55	0.02
Hexa(methoxymethyl)melamine	0.10	-0.06	-0.53	0.75	0.47	0.73	0.53	0.07
N-Cyclohexyl-N'-phenylurea	-0.20	-0.20	-0.53	0.76	0.67	0.84	0.75	0.03
Triphenylphosphine Oxide	-0.28	-0.32	-0.49	0.62	0.57	0.60	0.62	-0.42
Cells highlighted in green and red indicate statistically significant ($p < 0.05$) positive and negative correlations, respectively.								

S6. Watershed attributes as factors for prioritization of nontarget compounds in POCIS

Table S14. Spearman's correlation matrix between the *z*-score standardized peak intensities of 154 nontarget features and watershed attributes

ID	Agricultural	CFAOs	RPI	Developed	SepticDensity	RoadDensity	PopulationDensity	MunicipalWWTPs
NP197a	-0.13	-0.40	-0.56	0.69	0.76	0.58	0.65	0.11
NP212	0.30	0.16	-0.18	0.29	0.07	0.21	0.00	0.69
NP255a	0.76	0.60	0.40	-0.19	-0.23	-0.39	-0.22	0.35
NP258a	0.36	0.22	-0.05	0.22	0.04	0.15	-0.04	0.74
NP284d	-0.04	-0.28	-0.15	0.50	0.64	0.58	0.59	0.11
NP343a	-0.16	-0.50	-0.57	0.67	0.65	0.64	0.63	0.04
NP343b	-0.02	-0.28	-0.48	0.68	0.81	0.65	0.71	0.09
NP361	0.27	0.13	-0.33	0.47	0.19	0.41	0.14	0.66
NP363	0.23	0.05	-0.13	0.26	0.00	0.21	-0.06	0.58
NP399	0.23	0.08	-0.19	0.39	0.07	0.23	0.04	0.68
NP403	0.84	0.79	0.29	-0.09	-0.23	-0.22	-0.15	0.28
NP155	0.89	0.71	0.45	-0.19	-0.35	-0.32	-0.28	0.37
NP162	0.20	-0.06	-0.12	0.32	0.10	0.22	0.00	0.58
NP167a	0.38	0.21	-0.21	0.45	0.15	0.33	0.09	0.68
NP180	-0.04	-0.17	-0.27	0.40	0.14	0.26	0.02	0.65
NP183	0.02	-0.13	-0.35	0.64	0.71	0.64	0.63	0.18
NP211	0.84	0.68	0.41	-0.21	-0.30	-0.36	-0.24	0.31
NP217	0.84	0.68	0.22	0.02	-0.12	-0.13	-0.02	0.26
NP237a	0.62	0.49	0.35	-0.12	-0.07	-0.22	-0.07	0.15
NP257	0.82	0.62	0.37	-0.14	-0.16	-0.26	-0.09	0.32
NP259a	0.82	0.60	0.36	-0.14	-0.17	-0.31	-0.12	0.37
NP269a	0.77	0.63	0.29	-0.08	-0.17	-0.23	-0.11	0.43
NP270	0.20	0.05	-0.26	0.42	0.05	0.32	0.05	0.65
NP275	0.78	0.60	0.52	-0.31	-0.31	-0.50	-0.32	0.26
NP277	0.85	0.81	0.34	-0.07	-0.16	-0.14	-0.07	0.29
NP279b	0.10	-0.11	-0.10	0.52	0.60	0.54	0.49	-0.11
NP279d	0.28	0.12	-0.24	0.46	0.22	0.36	0.12	0.58
NP284c	0.29	0.15	-0.27	0.41	0.10	0.31	0.11	0.69
NP329b	0.09	-0.18	-0.32	0.65	0.72	0.66	0.66	0.15
NP335b	0.17	0.00	-0.22	0.35	-0.01	0.21	-0.03	0.59
NP359	0.80	0.63	0.32	-0.17	-0.20	-0.28	-0.12	0.22
NP369	0.82	0.64	0.36	-0.09	-0.12	-0.19	-0.09	0.37
NP371b	-0.14	-0.40	-0.60	0.71	0.73	0.69	0.76	0.03
NP457	0.83	0.71	0.37	-0.10	-0.15	-0.17	-0.09	0.41
NN293	0.29	0.13	-0.12	0.41	0.14	0.30	0.05	0.63
NP150	0.83	0.60	0.33	-0.06	-0.04	-0.23	-0.04	0.34
NP153	0.82	0.60	0.29	-0.05	-0.20	-0.20	-0.12	0.32
NP157	0.84	0.70	0.34	-0.22	-0.38	-0.35	-0.28	0.33
NP167b	0.79	0.63	0.47	-0.21	-0.17	-0.35	-0.14	0.26
NP170	0.86	0.71	0.50	-0.29	-0.40	-0.39	-0.34	0.29
NP185	0.78	0.60	0.35	-0.09	-0.11	-0.29	-0.09	0.33

**Table S14.** Spearman's correlation matrix between the *z*-score standardized peak intensities of 154 nontarget features and watershed attributes (continued)								
ID	Agricultural	CFAOs	RPI	Developed	SepticDensity	RoadDensity	PopulationDensity	MunicipalWWTPs
NP185	0.78	0.60	0.35	-0.09	-0.11	-0.29	-0.09	0.33
NP187a	0.86	0.64	0.43	-0.17	-0.25	-0.34	-0.20	0.34
NP187b	0.30	0.18	-0.17	0.41	0.11	0.31	0.02	0.71
NP196	0.77	0.55	0.27	-0.07	-0.07	-0.16	-0.03	0.35
NP197b	0.23	0.04	-0.33	0.49	0.14	0.30	0.12	0.70
NP197c	0.79	0.62	0.40	-0.24	-0.31	-0.29	-0.20	0.12
NP205	0.80	0.58	0.41	-0.25	-0.35	-0.27	-0.29	0.21
NP216	0.78	0.62	0.30	-0.02	-0.20	-0.26	-0.11	0.23
NP220	-0.02	-0.22	-0.35	0.62	0.72	0.72	0.61	0.06
NP221	0.75	0.63	0.32	-0.19	-0.27	-0.21	-0.19	0.16
NP225	0.75	0.58	0.38	-0.18	-0.16	-0.31	-0.15	0.26
NP227	0.32	0.11	-0.23	0.46	0.18	0.38	0.12	0.61
NP231a	0.40	0.26	-0.17	0.38	0.08	0.28	0.10	0.69
NP231b	0.18	0.05	-0.35	0.52	0.32	0.39	0.21	0.64
NP231c	-0.26	-0.42	-0.40	0.59	0.72	0.57	0.62	0.09
NP233	-0.08	-0.41	-0.35	0.60	0.74	0.63	0.60	0.08
NP237b	-0.14	-0.31	-0.38	0.57	0.84	0.66	0.72	-0.09
NP241a	0.85	0.63	0.32	-0.14	-0.21	-0.30	-0.15	0.33
NP241b	0.40	0.15	-0.22	0.38	0.17	0.25	0.10	0.61
NP247	0.86	0.67	0.37	-0.05	-0.09	-0.22	-0.06	0.39
NP250	0.14	0.01	-0.32	0.50	0.27	0.41	0.18	0.67
NP255b	0.28	0.08	-0.20	0.38	0.06	0.25	0.02	0.66
NP258b	0.35	0.15	-0.22	0.42	0.08	0.26	0.05	0.64
NP259b	0.28	0.20	-0.24	0.41	0.23	0.30	0.14	0.72
NP263	-0.11	-0.37	-0.29	0.50	0.81	0.46	0.72	-0.07
NP265a	-0.16	-0.33	-0.49	0.76	0.68	0.80	0.65	0.14
NP265b	0.39	0.26	0.00	0.30	0.02	0.19	-0.05	0.69
NP269b	0.43	0.22	-0.19	0.48	0.20	0.37	0.17	0.62
NP271	-0.11	-0.43	-0.30	0.59	0.61	0.51	0.53	0.11
NP272	0.07	-0.07	-0.33	0.41	0.15	0.29	0.07	0.60
NP274	0.14	0.00	-0.38	0.49	0.17	0.35	0.14	0.66
NP278	0.82	0.74	0.37	-0.15	-0.31	-0.25	-0.23	0.29
NP279a	0.77	0.72	0.20	-0.11	-0.34	-0.31	-0.24	0.19
NP279c	0.75	0.73	0.30	-0.23	-0.23	-0.33	-0.16	0.18
NP284a	0.82	0.71	0.43	-0.20	-0.41	-0.32	-0.28	0.29
NP284b	0.80	0.63	0.26	-0.05	-0.14	-0.25	-0.12	0.42
NP288a	0.42	0.26	-0.15	0.30	0.04	0.22	0.01	0.67
NP288b	-0.32	-0.51	-0.26	0.49	0.80	0.57	0.66	-0.06
NP288c	0.30	0.16	-0.32	0.42	0.09	0.30	0.05	0.71
NP291a	0.83	0.66	0.29	-0.09	-0.18	-0.23	-0.10	0.39
NP291b	0.70	0.58	0.29	-0.11	-0.25	-0.27	-0.22	0.37
NP292a	0.81	0.69	0.30	-0.09	-0.20	-0.21	-0.16	0.49
NP292b	0.20	0.13	-0.23	0.48	0.14	0.41	0.12	0.67
NP295	0.13	0.01	-0.37	0.46	0.11	0.32	0.08	0.66

**Table S14.** Spearman's correlation matrix between the *z*-score standardized peak intensities of 154 nontarget features and watershed attributes (continued)								
ID	Agricultural	CFAOs	RPI	Developed	SepticDensity	RoadDensity	PopulationDensity	MunicipalWWTPs
NP296a	0.85	0.65	0.45	-0.24	-0.36	-0.37	-0.31	0.25
NP296b	0.20	0.07	-0.29	0.39	0.10	0.24	0.07	0.71
NP298	0.79	0.61	0.37	-0.23	-0.23	-0.37	-0.14	0.22
NP301	0.82	0.61	0.25	-0.02	-0.06	-0.16	0.01	0.38
NP302	-0.15	-0.41	-0.47	0.68	0.77	0.73	0.71	0.11
NP303	0.30	0.08	-0.41	0.63	0.34	0.47	0.33	0.64
NP306	-0.02	-0.32	-0.32	0.63	0.79	0.66	0.69	0.09
NP307	0.18	0.05	-0.34	0.47	0.16	0.37	0.12	0.68
NP309	0.83	0.68	0.35	-0.16	-0.29	-0.26	-0.23	0.26
NP311	0.17	0.05	-0.38	0.52	0.19	0.35	0.14	0.69
NP312	0.85	0.67	0.38	-0.09	-0.06	-0.19	-0.05	0.26
NP314	0.04	-0.07	-0.36	0.42	0.23	0.32	0.15	0.63
NP320	0.86	0.66	0.34	-0.05	-0.10	-0.21	-0.06	0.40
NP323a	0.26	0.12	-0.33	0.51	0.16	0.32	0.17	0.72
NP323b	0.05	-0.29	-0.33	0.62	0.73	0.58	0.62	0.19
NP326	0.85	0.66	0.42	-0.23	-0.35	-0.37	-0.27	0.34
NP329a	-0.15	-0.38	-0.46	0.64	0.77	0.65	0.63	0.10
NP330	0.17	0.05	-0.29	0.48	0.16	0.41	0.12	0.67
NP332	0.20	0.01	-0.21	0.29	0.12	0.16	0.00	0.68
NP335a	0.25	0.14	-0.25	0.40	0.06	0.36	0.08	0.59
NP335c	0.29	0.09	-0.10	0.28	-0.05	0.17	-0.10	0.61
NP345	0.77	0.66	0.30	-0.15	-0.19	-0.27	-0.10	0.32
NP347a	-0.21	-0.34	-0.45	0.53	0.76	0.62	0.65	0.08
NP347b	-0.28	-0.43	-0.48	0.69	0.79	0.66	0.67	0.15
NP349	0.41	0.24	-0.17	0.36	0.16	0.27	0.09	0.61
NP350	0.83	0.62	0.42	-0.15	-0.22	-0.32	-0.17	0.34
NP354	0.33	0.15	-0.05	0.27	-0.02	0.15	-0.11	0.67
NP358a	0.23	0.10	-0.31	0.40	0.19	0.28	0.18	0.72
NP358b	0.36	0.21	-0.13	0.40	0.05	0.28	0.03	0.68
NP364	0.27	0.07	-0.13	0.32	0.09	0.22	0.03	0.75
NP365	0.41	0.24	-0.20	0.42	0.09	0.30	0.07	0.71
NP366	0.79	0.53	0.34	-0.16	-0.21	-0.29	-0.19	0.35
NP367	-0.16	-0.29	-0.43	0.64	0.81	0.61	0.70	0.04
NP370	0.38	0.21	-0.27	0.45	0.14	0.29	0.14	0.75
NP371a	0.82	0.67	0.37	-0.16	-0.35	-0.35	-0.25	0.32
NP375	-0.22	-0.41	-0.40	0.60	0.80	0.64	0.65	0.06
NP379	0.29	0.13	-0.27	0.43	0.07	0.25	0.04	0.73
NP381	0.29	0.10	-0.21	0.50	0.25	0.29	0.16	0.61
NP383	-0.19	-0.39	-0.35	0.62	0.80	0.66	0.70	0.06
NP386	0.30	0.23	-0.21	0.37	0.12	0.23	0.12	0.75
NP391	0.22	0.06	-0.25	0.48	0.26	0.44	0.17	0.64
NP397	-0.07	-0.24	-0.51	0.75	0.82	0.72	0.71	0.18
NP398	0.21	0.10	-0.09	0.19	-0.02	0.04	-0.12	0.76
NP400	0.23	0.10	-0.29	0.50	0.22	0.37	0.18	0.73

Table S14. Spearman's correlation matrix between the *z*-score standardized peak intensities of 154 nontarget features and watershed attributes (continued)

ID	Agricultural	CFAOs	RPI	Developed	SepticDensity	RoadDensity	PopulationDensity	MunicipalWWTPs
NP423	0.78	0.76	0.38	-0.19	-0.24	-0.36	-0.22	0.34
NP428	0.24	0.07	-0.37	0.56	0.18	0.38	0.17	0.65
NP432	0.82	0.67	0.34	-0.11	-0.15	-0.24	-0.09	0.40
NP435	0.06	-0.26	-0.31	0.60	0.69	0.69	0.62	0.08
NP441a	0.79	0.62	0.32	-0.16	-0.29	-0.24	-0.22	0.28
NP441b	0.82	0.71	0.41	-0.23	-0.29	-0.35	-0.28	0.36
NP449	0.31	0.18	-0.31	0.52	0.22	0.41	0.19	0.69
NP469	0.85	0.74	0.38	-0.16	-0.34	-0.32	-0.26	0.41
NP472	0.34	0.14	-0.16	0.39	0.04	0.24	-0.04	0.64
NP475	0.80	0.62	0.31	-0.12	-0.14	-0.25	-0.10	0.34
NP481a	0.72	0.53	0.28	0.02	-0.01	-0.21	-0.03	0.34
NP481b	-0.09	-0.37	-0.48	0.69	0.75	0.60	0.68	0.13
NP488	0.32	0.18	-0.26	0.35	0.14	0.27	0.15	0.68
NP511a	0.33	0.19	-0.30	0.56	0.35	0.41	0.29	0.69
NP511b	-0.01	-0.25	-0.31	0.46	0.80	0.53	0.71	0.17
NP516a	0.33	0.20	-0.28	0.51	0.11	0.34	0.09	0.72
NP516b	0.07	-0.19	-0.33	0.54	0.64	0.62	0.59	0.18
NP532	0.27	0.11	-0.32	0.45	0.16	0.25	0.15	0.74
NP546	0.27	0.07	-0.27	0.51	0.22	0.35	0.15	0.66
NP578	0.85	0.74	0.39	-0.14	-0.20	-0.28	-0.18	0.50
NP590	-0.24	-0.54	-0.22	0.45	0.74	0.45	0.56	-0.06
NP647	-0.06	-0.36	-0.37	0.70	0.66	0.71	0.57	0.15
NP671	0.20	0.05	-0.26	0.45	0.29	0.29	0.17	0.69
NP692	0.16	-0.01	-0.17	0.36	0.14	0.27	0.06	0.65
NP748	0.21	0.03	-0.18	0.34	0.07	0.30	-0.01	0.65
NP803	0.30	0.18	-0.22	0.40	0.11	0.35	0.05	0.63

Cells highlighted in green and red indicate statistically significant ($p < 0.05$) positive and negative correlations, respectively.

**Table S15.** Summary of 154 compounds prioritized by nontarget screening												
ID	Molecular Formula	RDBE	H/C	O/C	SFit [%]	Adduct	Exact Mass (*m/z*)	MS/MS Fragment (*m/z*)	RT (min)	*mzCloud* Match Factor	Level	Compound Name
NP197a	$C_{14}H_{12}O$	9	0.9	0.4	99.9	[M+H]+	197.0967	105.0337, 119.0494,	18.86	72.3	1	4-Methylbenzophenone
NP212	$C_{13}H_{25}NO$	2	1.9	0.1	98.9	[M+H]+	212.2009	123.1169, 149.1320, 170.1539	18.60	85.9	1	N-Ethyl-p-menthane-3-carboxamide
NP255a	$C_{15}H_{10}O_4$	11	0.7	0.3	97.4	[M+H]+	255.0660	137.0236, 199.0761, 227.0707	13.56	85.9	1	Daidzein
NP258a	$C_{17}H_{23}NO$	7	1.4	0.1	99.0	[M+H]+	258.1858	133.0642, 157.0637, 199.1106	10.49	93.0	1	Dextrorphan
NP284d	$C_{19}H_{42}N^{+}$	0	2.2	-	99.9	[M]+	284.3310	203.0763	22.92	87.8	1	Hexadecyltrimethylammonium
NP343a	$C_{20}H_{22}O_5$	10	1.1	0.3	100	[M+H]+	343.1540	105.0336, 163.0756	20.86	90.6	1	Di(propylene glycol) Dibenzoate
NP343b	$C_{19}H_{38}N_2O_3$	2	2.0	0.2	99.8	[M+H]+	343.2955	109.1010, 183.1739, 240.2320	21.03	90.3	1	Lauramidopropyl Betaine
NP361	$C_{18}H_{32}O_7$	3	1.8	0.4	97.7	[M+H]+	361.2221	111.0073, 129.0177, 139.0025	21.77	86.7	1	Tributyl Citrate
NP363	$C_{22}H_{34}O_4$	6	1.5	0.2	99.7	[M+H]+	363.2530	121.0283, 149.0234	25.60	88.3	1	Diheptyl Phthalate
NP399	$C_{18}H_{39}O_7P$	0	2.2	0.4	100	[M+H]+	399.2514	143.0104, 199.0761, 299.1623	22.05	90.3	1	Tris(2-butoxyethyl) Phosphate
NP403	$C_{21}H_{22}O_8$	7	1.5	0.5	97.7	[M+H]+	403.1390	327.0855, 355.0802, 373.0912	17.81	90.3	1	Nobiletin
NP155	$C_8H_{10}O_3$	4	1.3	0.4	90.7	[M+H]+	155.0703	95.0490, 123.0437	11.58	87.7	3	Dimethoxyphenol
NP162	$C_9H_7NO_2$	7	0.8	0.2	90.7	[M+H]+	162.0550	144.0444	10.21	86.2	3	Quinolinediol
NP167a	$C_8H_6O_4$	6	0.8	0.5	98.4	[M+H]+	167.0339	130.9809	21.21	85.9	3	Phthalic Acid
NP180	$C_{13}H_9N$	10	0.7	-	99.0	[M+H]+	180.0808	112.4798	9.72	79.9	3	Acridine
NP183	$C_9H_{10}O_4$	5	1.1	0.4	90.6	[M+H]+	183.0652	137.05963	10.47	77.6	3	Isohomovanillic Acid
NP211	$C_{12}H_{18}O_3$	7	1.5	0.3	90.7	[M+H]+	211.1329	193.12224	10.75	85.3	3	Jasmonic Acid
NP217	$C_{15}H_{20}O$	6	1.3	0.1	98.9	[M+H]+	217.1587	119.0847	19.30	81.6	3	ar-Turmerone
NP237a	$C_{12}H_{12}O_5$	7	1.0	0.4	100	[M+H]+	237.0758	207.0290	13.92	81.2	3	Trimethoxycoumarin
NP257	$C_{15}H_{12}O_4$	10	0.8	0.3	92.9	[M+H]+	257.0808	153.01823	16.26	83.6	3	Pinocembrin
NP259a	$C_{15}H_{14}O_4$	9	0.9	0.3	97.9	[M+H]+	259.0965	241.0862	15.62	83.6	3	Isorhapontigenin
NP269a	$C_{16}H_{12}O_4$	11	0.8	0.3	98.0	[M+H]+	269.0808	254.0581	16.22	86.8	3	Formononetin
NP270	$C_{18}H_{39}N$	0	2.2	-	99.7	[M+H]+	270.3155	203.5501	19.64	89.0	3	Octadecylamine
NP275	$C_{15}H_{30}O_4$	1	2.0	0.3	98.1	[M+H]+	275.2217	203.5570	22.94	85.9	3	Monolaurin
NP277	$C_{17}H_{24}O_3$	6	1.4	0.2	98.0	[M+H]+	277.1798	259.1332	18.05	80.6	3	Shogaol
NP279b	$C_{16}H_{22}O_4$	6	1.4	0.3	99.9	[M+H]+	279.1591	149.0228	22.00	84.5	3	Mono(2-ethylhexyl) Phthalate
NP279d	$C_{18}H_{30}O_2$	4	1.7	0.1	99.0	[M+H]+	279.2319	149.0232	19.39	82.7	3	Pinolenic Acid
NP284c	$C_{18}H_{37}NO$	1	2.1	0.1	100	[M+H]+	284.2948	203.1833	15.03	84.7	3	Octadecanamide
NP329b	$C_{22}H_{32}O_2$	7	1.5	0.1	99.9	[M+H]+	329.2475	98.9838	26.38	80.1	3	Docosahexaenoic Acid
NP335b	$C_{20}H_{18}NO_4^{+}$	13	0.9	0.2	90.3	[M]+	335.1158	292.0965, 306.0761, 321.0996	11.70	82.7	3	Berberine
NP359	$C_{22}H_{30}O_4$	8	1.4	0.2	100	[M+H]+	359.2217	341.2111	26.26	85.9	3	Cannabidiolic Acid
NP369	$C_{21}H_{24}N_2O_4$	11	1.1	0.2	94.3	[M+H]+	369.1809	337.1555	11.05	88.2	3	Mitraphylline
NP371b	$C_{22}H_{42}O_4$	2	1.9	0.2	100	[M+H]+	371.3156	111.0432	27.29	91.3	3	Bis(2-ethylhexyl) Adipate
NP457	$C_{30}H_{48}O_3$	7	1.6	0.1	98.0	[M+H]+	457.3676	201.1637	23.11	84.5	3	Oleanolic Acid
NN293	$C_{14}H_{30}O_4S$	0	2.1	0.3	92.2	[M-H]-	293.1792	96.9601	25.38	83.3	3	Myristyl Sulfate
NP150	$C_5H_{11}NO_4$	1	2.2	0.8	100	[M+H]+	150.0761	-	1.63	-	4	-
NP153	$C_9H_{12}O_2$	4	1.3	0.2	96.2	[M+H]+	153.0910	-	12.40	-	4	-
NP157	$C_7H_{12}N_2O_2$	3	1.7	0.3	96.4	[M+H]+	157.0972	-	2.83	-	4	-
NP167b	$C_{10}H_{14}O_2$	4	1.4	0.2	98.2	[M+H]+	167.1067	-	14.41	-	4	-
NP170	$C_9H_{15}NO_2$	3	1.7	0.2	94.9	[M+H]+	170.1176	-	9.18	-	4	-
NP185	$C_{10}H_{16}O_3$	3	1.6	0.3	91.1	[M+H]+	185.1172	-	14.70	-	4	-
NP187a	$C_6H_6N_2O_5$	5	1.0	0.8	100	[M+H]+	187.0350	-	1.38	-	4	-
NP187b	$C_{11}H_6O_3$	9	0.6	0.3	100	[M+H]+	187.0390	-	1.38	-	4	-
NP196	$C_6H_{13}NO_4S$	1	2.2	0.7	100	[M+H]+	196.0638	-	4.62	-	4	-
NP197b	$C_{10}H_{12}O_4$	5	1.2	0.4	91.8	[M+H]+	197.0808	-	23.87	-	4	-
NP197c	$C_{11}H_{16}O_3$	4	1.5	0.3	90.3	[M+H]+	197.1172	-	10.69	-	4	-
NP205	$C_{10}H_{20}O_4$	1	2.0	0.4	96.8	[M+H]+	205.1434	-	1.99	-	4	-

Table S15. Summary of 154 compounds prioritized by nontarget screening (continued)												
ID	Molecular Formula	RDBE	H/C	O/C	SFit [%]	Adduct	Exact Mass (*m/z*)	MS/MS Fragment (*m/z*)	RT (min)	*mzCloud* Match Factor	Level	Compound Name
NP205	$C_{10}H_{20}O_4$	1	2.0	0.4	96.8	[M+H]+	205.1434	-	1.99	-	4	-
NP216	$C_8H_{13}N_3O_4$	4	1.6	0.5	100	[M+H]+	216.0979	-	15.44	-	4	-
NP220	$C_8H_{13}NO_6$	3	1.6	0.8	96.0	[M+H]+	220.0816	-	15.23	-	4	-
NP221	$C_{13}H_{16}O_3$	6	1.2	0.2	100	[M+H]+	221.1172	-	11.81	-	4	-
NP225	$C_{12}H_{16}O_4$	5	1.3	0.3	100	[M+H]+	225.1121	-	10.34	-	4	-
NP227	$C_{14}H_{26}O_2$	2	1.9	0.1	91.9	[M+H]+	227.2006	-	21.76	-	4	-
NP231a	$C_9H_{14}N_2O_5$	4	1.6	0.6	96.5	[M+H]+	231.0976	-	17.40	-	4	-
NP231b	$C_{13}H_{14}N_2O_2$	8	1.1	0.2	96.4	[M+H]+	231.1128	-	12.68	-	4	-
NP231c	$C_{11}H_{19}O_3P$	3	0.8	0.0	100	[M+H]+	231.1145	-	15.13	-	4	-
NP233	$C_{15}H_{20}O_2$	6	1.3	0.1	93.0	[M+H]+	233.1536	-	21.82	-	4	-
NP237b	$C_{15}H_{24}O_2$	4	1.6	0.1	92.8	[M+H]+	237.1849	-	17.82	-	4	-
NP241a	$C_{14}H_{24}O_3$	3	1.7	0.2	96.2	[M+H]+	241.1798	-	12.43	-	4	-
NP241b	$C_{15}H_{28}O_2$	2	1.9	0.1	92.0	[M+H]+	241.2162	-	20.32	-	4	-
NP247	$C_{12}H_{22}O_5$	2	1.8	0.1	91.0	[M+H]+	247.1540	-	16.10	-	4	-
NP250	$C_{14}H_{19}NO_3$	6	1.4	0.2	94.3	[M+H]+	250.1438	-	11.06	-	4	-
NP255b	$C_{16}H_{30}O_2$	2	1.9	0.1	92.9	[M+H]+	255.2319	-	20.33	-	4	-
NP258b	$C_{15}H_{31}NO_2$	1	2.1	0.1	92.7	[M+H]+	258.2428	-	13.26	-	4	-
NP259b	$C_{17}H_{22}O_2$	7	1.3	0.1	96.5	[M+H]+	259.1693	-	21.03	-	4	-
NP263	$C_{15}H_{18}O_4$	7	1.2	0.3	94.2	[M+H]+	263.1278	-	20.00	-	4	-
NP265a	$C_{12}H_{24}O_6$	1	2.0	0.5	100	[M+H]+	265.1646	-	13.66	-	4	-
NP265b	$C_{16}H_{24}O_3$	5	1.5	0.2	95.7	[M+H]+	265.1798	-	19.91	-	4	-
NP269b	$C_{14}H_{20}O_3S$	5	1.4	0.2	90.4	[M+H]+	269.1206	-	20.07	-	4	-
NP271	$C_{15}H_{26}O_4$	3	1.7	0.3	95.4	[M+H]+	271.1904	-	22.51	-	4	-
NP272	$C_{14}H_{13}N_3O_3$	10	0.9	0.2	100	[M+H]+	272.1030	-	10.51	-	4	-
NP274	$C_{12}H_{19}NO_6$	4	1.6	0.5	100	[M+H]+	274.1285	-	11.66	-	4	-
NP278	$C_{15}H_{19}NO_4$	7	1.3	0.3	93.2	[M+H]+	278.1387	-	13.55	-	4	-
NP279a	$C_{15}H_{18}O_5$	7	1.2	0.3	97.7	[M+H]+	279.1227	-	16.03	-	4	-
NP279c	$C_{17}H_{26}O_3$	5	1.5	0.2	93.4	[M+H]+	279.1955	-	17.32	-	4	-
NP284a	$C_{14}H_{21}NO_3S$	5	1.5	0.2	96.4	[M+H]+	284.1315	-	19.08	-	4	-
NP284b	$C_{16}H_{17}N_3O_2$	10	1.1	0.1	100	[M+H]+	284.1394	-	7.42	-	4	-
NP288a	$C_{15}H_{17}N_3O_3$	9	1.1	0.2	100	[M+H]+	288.1343	-	11.22	-	4	-
NP288b	$C_{16}H_{33}NO_3$	1	2.1	0.2	93.1	[M+H]+	288.2533	-	21.75	-	4	-
NP288c	$C_{15}H_{33}N_3O_2$	1	2.2	0.1	100	[M+H]+	288.2646	-	21.76	-	4	-
NP291a	$C_{10}H_{15}N_2O_4PS$	5	1.5	0.4	93.5	[M+H]+	291.0563	-	10.87	-	4	-
NP291b	$C_{16}H_{18}O_5$	8	1.1	0.3	90.2	[M+H]+	291.1227	-	18.35	-	4	-
NP292a	$C_9H_{14}N_3O_4PS$	5	1.6	0.4	98.6	[M+H]+	292.0515	-	15.88	-	4	-
NP292b	$C_{18}H_{29}NO_2$	5	1.6	0.1	95.6	[M+H]+	292.2271	-	20.78	-	4	-
NP295	$C_{20}H_{22}O_2$	10	1.1	0.1	100	[M+H]+	295.1693	-	21.85	-	4	-
NP296a	$C_{15}H_{21}NO_3S$	6	1.4	0.2	90.9	[M+H]+	296.1315	-	13.50	-	4	-
NP296b	$C_{17}H_{17}N_3O_2$	11	1.0	0.1	100	[M+H]+	296.1394	-	8.11	-	4	-
NP298	$C_{14}H_{19}NO_4S$	6	1.4	0.3	96.6	[M+H]+	298.1108	-	14.76	-	4	-
NP301	$C_{17}H_{16}O_5$	10	0.9	0.3	93.0	[M+H]+	301.1071	-	16.40	-	4	-
NP302	$C_{18}H_{39}NO_2$	0	2.2	0.1	91.0	[M+H]+	302.3054	-	12.83	-	4	-
NP303	$C_{16}H_{14}O_6$	10	0.9	0.4	91.2	[M+H]+	303.0863	-	12.98	-	4	-
NP306	$C_{19}H_{31}NO_2$	5	1.6	0.1	96.2	[M+H]+	306.2428	-	13.01	-	4	-
NP307	$C_{15}H_{30}O_6$	1	2.0	0.4	95.3	[M+H]+	307.2115	-	13.27	-	4	-
NP309	$C_{18}H_{16}N_2O_3$	12	0.9	0.2	100	[M+H]+	309.1234	-	11.11	-	4	-

Table S15. Summary of 154 compounds prioritized by nontarget screening (continued)												
ID	Molecular Formula	RDBE	H/C	O/C	SFit [%]	Adduct	Exact Mass (*m/z*)	MS/MS Fragment (*m/z*)	RT (min)	*mzCloud* Match Factor	Level	Compound Name
NP311	$C_{14}H_{30}O_7$	0	2.1	0.5	100	[M+H]+	311.2064	-	10.95	-	4	-
NP312	$C_{15}H_{21}NO_4S$	6	1.4	0.3	90.9	[M+H]+	312.1264	-	14.03	-	4	-
NP314	$C_{17}H_{19}N_3O_3$	10	1.1	0.2	100	[M+H]+	314.1499	-	11.33	-	4	-
NP320	$C_{12}H_{17}NO_9$	5	1.4	0.8	90.8	[M+H]+	320.0976	-	14.77	-	4	-
NP323a	$C_{16}H_{22}N_2O_5$	7	1.4	0.3	97.5	[M+H]+	323.1602	-	23.63	-	4	-
NP323b	$C_{21}H_{38}O_2$	3	1.8	0.1	95.1	[M+H]+	323.2945	-	25.58	-	4	-
NP326	$C_{16}H_{23}NO_4S$	6	1.4	0.3	95.1	[M+H]+	326.1421	-	12.43	-	4	-
NP329a	$C_{17}H_{28}O_4S$	4	1.6	0.2	92.0	[M+H]+	329.1781	-	19.18	-	4	-
NP330	$C_{20}H_{43}NO_2$	0	2.2	0.1	92.5	[M+H]+	330.3367	-	14.60	-	4	-
NP332	$C_{18}H_{21}NO_5$	9	1.2	0.3	99.1	[M+H]+	332.1493	-	16.18	-	4	-
NP335a	$C_{12}H_{19}N_2O_7P$	5	1.6	0.6	92.8	[M+H]+	335.1003	-	13.81	-	4	-
NP335c	$C_{17}H_{34}O_6$	1	2.0	0.4	100	[M+H]+	335.2428	-	18.30	-	4	-
NP345	$C_{11}H_{21}O_{10}P$	2	1.9	0.9	94.3	[M+H]+	345.0945	-	17.28	-	4	-
NP347a	$C_{20}H_{26}O_5$	8	1.3	0.3	94.3	[M+H]+	347.1853	-	23.42	-	4	-
NP347b	$C_{18}H_{34}O_6$	2	1.9	0.3	100	[M+H]+	347.2428	-	17.52	-	4	-
NP349	$C_{18}H_{36}O_6$	1	2.0	0.3	100	[M+H]+	349.2585	-	20.41	-	4	-
NP350	$C_{15}H_{27}NO_6S$	3	1.8	0.4	96.4	[M+H]+	350.1632	-	20.44	-	4	-
NP354	$C_{19}H_{19}N_3O_2S$	12	1.0	0.1	90.5	[M+H]+	354.1271	-	13.97	-	4	-
NP358a	$C_{20}H_{39}NO_4$	2	2.0	0.2	91.7	[M+H]+	358.2952	-	20.09	-	4	-
NP358b	$C_{22}H_{47}NO_2$	0	2.1	0.1	95.7	[M+H]+	358.3680	-	15.05	-	4	-
NP364	$C_{14}H_{25}N_3O_6S$	4	1.8	0.4	93.8	[M+H]+	364.1537	-	19.01	-	4	-
NP365	$C_{18}H_{37}O_5P$	1	2.1	0.3	94.6	[M+H]+	365.2451	-	11.79	-	4	-
NP366	$C_{21}H_{35}NO_4$	5	1.7	0.2	97.7	[M+H]+	366.2639	-	19.26	-	4	-
NP367	$C_{22}H_{42}N_2O_2$	3	1.9	0.1	94.2	[M+H]+	367.3319	-	19.17	-	4	-
NP370	$C_{19}H_{31}NO_6$	5	1.6	0.3	100	[M+H]+	370.2224	-	19.09	-	4	-
NP371a	$C_{22}H_{26}O_5$	10	1.2	0.2	92.4	[M+H]+	371.1853	-	22.64	-	4	-
NP375	$C_{16}H_{26}N_2O_8$	5	1.6	0.5	91.7	[M+H]+	375.1762	-	19.10	-	4	-
NP379	$C_{20}H_{42}O_6$	0	2.1	0.3	90.6	[M+H]+	379.3054	-	22.75	-	4	-
NP381	$C_{18}H_{36}O_8$	1	2.0	0.4	92.6	[M+H]+	381.2483	-	15.59	-	4	-
NP383	$C_{22}H_{38}O_3S$	4	1.7	0.1	100	[M+H]+	383.2615	-	13.52	-	4	-
NP386	$C_{23}H_{31}NO_4$	9	1.4	0.2	91.5	[M+H]+	386.2326	-	14.70	-	4	-
NP391	$C_{22}H_{46}O_5$	0	2.1	0.2	94.2	[M+H]+	391.3418	-	25.26	-	4	-
NP397	$C_{17}H_{33}O_8P$	2	1.9	0.5	91.6	[M+H]+	397.1986	-	27.06	-	4	-
NP398	$C_{21}H_{23}N_3O_3S$	12	1.1	0.1	96.7	[M+H]+	398.1533	-	13.79	-	4	-
NP400	$C_{22}H_{41}NO_5$	3	1.9	0.2	93.8	[M+H]+	400.3058	-	18.74	-	4	-
NP423	$C_{20}H_{22}O_{10}$	10	1.1	0.5	93.5	[M+H]+	423.1286	-	9.54	-	4	-
NP428	$C_{19}H_{41}NO_9$	0	2.2	0.5	95.0	[M+H]+	428.2854	-	14.57	-	4	-
NP432	$C_{26}H_{41}NO_4$	7	1.6	0.2	94.0	[M+H]+	432.3108	-	20.53	-	4	-
NP435	$C_{24}H_{34}O_7$	8	1.4	0.3	94.9	[M+H]+	435.2377	-	24.00	-	4	-
NP441a	$C_{19}H_{40}N_2O_7S$	1	2.1	0.4	96.8	[M+H]+	441.2629	-	19.50	-	4	-
NP441b	$C_{28}H_{40}O_4$	9	1.4	0.1	96.5	[M+H]+	441.2999	-	21.83	-	4	-
NP449	$C_{25}H_{52}O_6$	0	2.1	0.2	91.1	[M+H]+	449.3837	-	17.33	-	4	-
NP469	$C_{31}H_{32}O_4$	16	1.0	0.1	98.2	[M+H]+	469.2373	-	20.13	-	4	-
NP472	$C_{21}H_{45}NO_{10}$	0	2.1	0.5	94.9	[M+H]+	472.3116	-	13.85	-	4	-
NP475	$C_{30}H_{50}O_4$	6	1.7	0.1	99.7	[M+H]+	475.3782	-	26.97	-	4	-
NP481a	$C_{17}H_{37}O_{13}P$	0	2.2	0.8	97.1	[M+H]+	481.2045	-	19.37	-	4	-
NP481b	$C_{25}H_{52}O_8$	0	2.1	0.3	93.8	[M+H]+	481.3735	-	22.18	-	4	-

**Table S15.** Summary of 154 compounds prioritized by nontarget screening (continued)												
ID	Molecular Formula	RDBE	H/C	O/C	SFit [%]	Adduct	Exact Mass (*m/z*)	MS/MS Fragment (*m/z*)	RT (min)	*mzCloud* Match Factor	Level	Compound Name
NP488	$C_{31}H_{37}NO_4$	14	1.2	0.1	98.3	[M+H]+	488.2795	-	14.62	-	4	-
NP511a	$C_{27}H_{42}O_7S$	7	1.6	0.3	90.4	[M+H]+	511.2724	-	10.92	-	4	-
NP511b	$C_{26}H_{54}O_9$	0	2.1	0.4	95.9	[M+H]+	511.3841	-	22.83	-	4	-
NP516a	$C_{26}H_{45}NO_7S$	5	1.7	0.3	98.7	[M+H]+	516.2990	-	9.86	-	4	-
NP516b	$C_{23}H_{49}NO_{11}$	0	2.1	0.5	91.3	[M+H]+	516.3378	-	15.24	-	4	-
NP532	$C_{35}H_{37}N_3O_2$	19	1.1	0.1	98.2	[M+H]+	532.2959	-	11.28	-	4	-
NP546	$C_{24}H_{51}NO_{12}$	0	2.1	0.5	92.6	[M+H]+	546.3484	-	13.25	-	4	-
NP578	$C_{28}H_{51}NO_{11}$	4	1.8	0.4	93.6	[M+H]+	578.3535	-	22.00	-	4	-
NP590	$C_{26}H_{55}NO_{13}$	0	2.1	0.5	90.6	[M+H]+	590.3746	-	13.65	-	4	-
NP647	$C_{30}H_{63}O_{12}P$	0	2.1	0.4	90.2	[M+H]+	647.4130	-	25.17	-	4	-
NP671	$C_{34}H_{71}NO_{11}$	0	2.1	0.3	94.4	[M+H]+	670.5100	-	23.79	-	4	-
NP692	$C_{31}H_{65}NO_{15}$	0	2.1	0.5	92.7	[M+H]+	692.4427	-	14.80	-	4	-
NP748	$C_{42}H_{69}NO_{10}$	9	1.6	0.2	96.6	[M+H]+	748.4994	-	19.72	-	4	-
NP803	$C_{40}H_{83}NO_{14}$	0	2.1	0.4	98.0	[M+H]+	802.5886	-	24.58	-	4	-

"RDBE" = the rings and double bonds equivalent value for the predicted formula; "H/C" = the ratio of hydrogen to carbon atoms in the predicted formula; "O/C" = the ratio of oxygen to carbon atoms in the predicted formula; "SFit(%)" = the spectral similarity score between the theoretical and the measured isotope patterns as a percentage. Identification confidence levels adapted from Schymanski *et al.*:[37] Level 1 – the structure was confirmed via matching MS, MS/MS, and retention time with those of the authentic reference standard. Level 3 – the molecular formula was proposed with >90% SFit(%) and the structure was tentatively proposed via matching MS and MS/MS fragments with those of the top-ranked structure in *mzCloud*. Level 4 – the molecular formula was proposed with >90% SFit(%) but no MS/MS fragmentation information for structural elucidation. "NP" denotes nontarget compounds detected in positive electrospray ionization mode. "NN" denotes nontarget compounds detected in negative electrospray ionization mode. No Level 2 identification was assigned because authentic reference standards were purchased for confirmation or rejection if a compound was tentatively identified via matching MS and MS/MS fragments with those of the top-ranked structure in *mzCloud*.

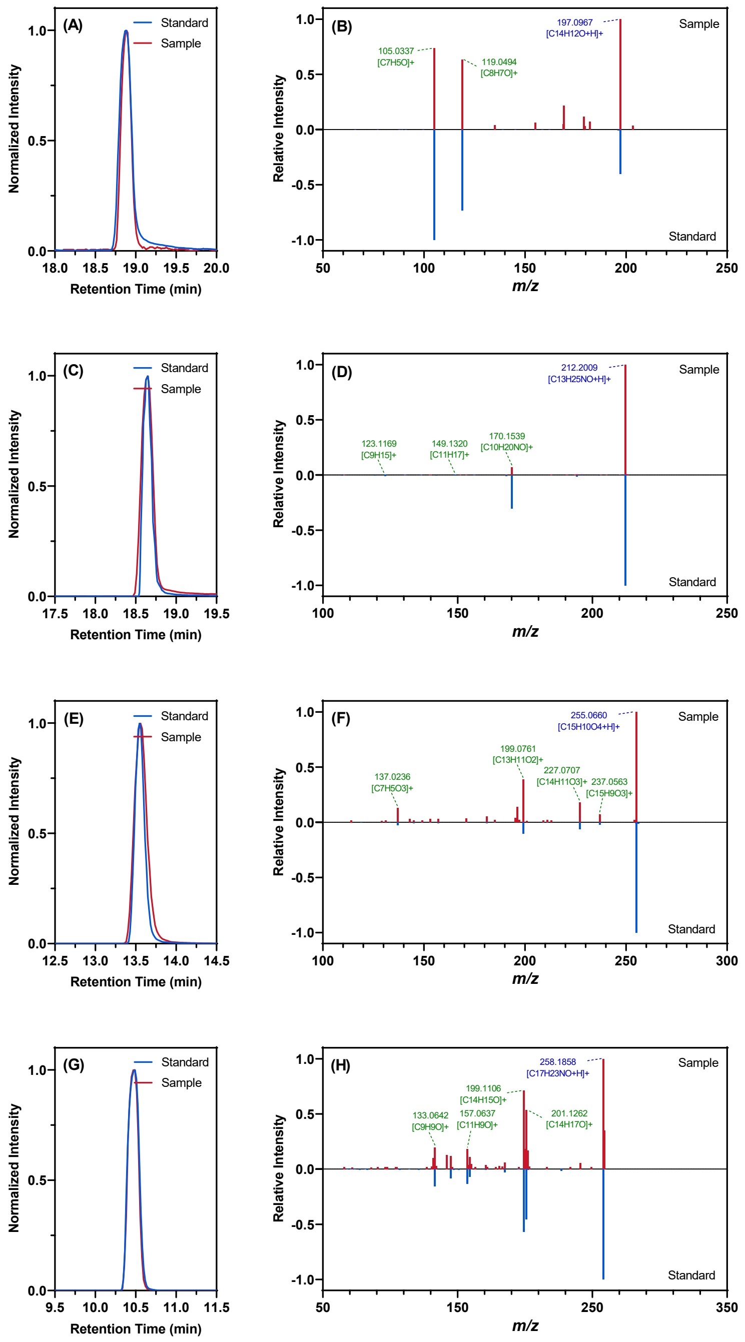

Figure S6a. Level 1 confirmation of 11 nontarget compounds: **(A)** Normalized extracted ion chromatograms of NP197a in POCIS extracts (retention time = 18.86 min) and 4-methylbenzophenone reference standard (retention time = 18.88 min). **(B)** Head-to-tail plot of dd-MS2 spectra of NP197a in POCIS extracts (HCD 60%) and 4-methylbenzophenone reference standard (HCD 60%). **(C)** Normalized extracted ion chromatograms of NP212 in POCIS extracts (retention time = 18.60 min) and N-ethyl-p-menthane-3-carboxamide reference standard (retention time = 18.65 min). **(D)** Head-to-tail plot of dd-MS2 spectra of NP212 in POCIS extracts (HCD 45%) and N-ethyl-p-menthane-3-carboxamide reference standard (HCD 45%). **(E)** Normalized extracted ion chromatograms of NP255a in POCIS extracts (retention time = 13.56 min) and daidzein reference standard (retention time = 13.55 min). **(F)** Head-to-tail plot of dd-MS2 spectra of NP255a in POCIS extracts (HCD 45%) and daidzein reference standard (HCD 45%). **(G)** Normalized extracted ion chromatograms of NP258a in POCIS extracts (retention time = 10.49 min) and dextrorphan reference standard (retention time = 10.49 min). **(H)** Head-to-tail plot of dd-MS2 spectra of NP258a in POCIS extracts (HCD 45%) and dextrorphan reference standard (HCD 45%). Further compound information is summarized in Table S15.

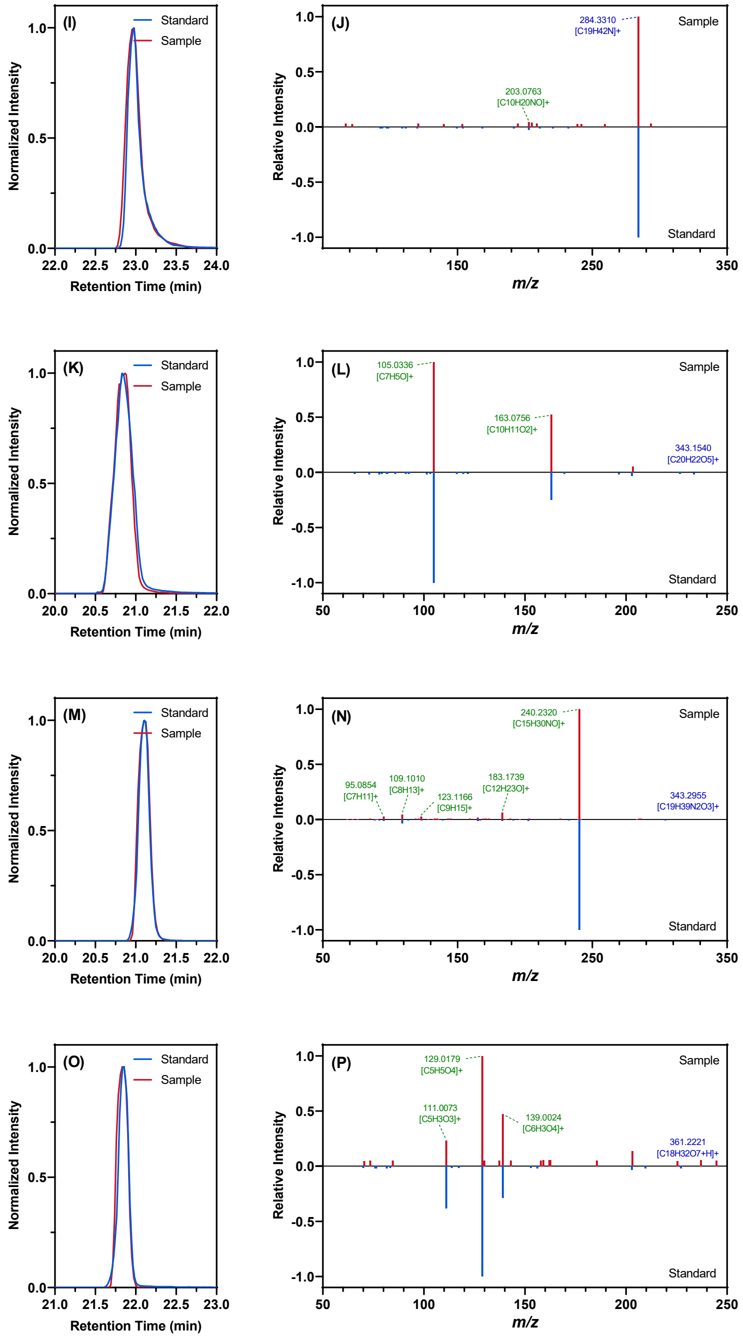

Figure S6b. Level 1 confirmation of 11 nontarget compounds: **(I)** Normalized extracted ion chromatograms of NP284d in POCIS extracts (retention time = 22.92 min) and hexadecyltrimethylammonium reference standard (retention time = 22.98 min). **(J)** Head-to-tail plot of dd-MS2 spectra of NP284d in POCIS extracts (HCD 60%) and hexadecyltrimethylammonium reference standard (HCD 60%). **(K)** Normalized extracted ion chromatograms of NP343a in POCIS extracts (retention time = 20.86 min) and di(propylene glycol) dibenzoate reference standard (retention time = 20.83 min). **(L)** Head-to-tail plot of dd-MS2 spectra of NP343a in POCIS extracts (HCD 60%) and di(propylene glycol) dibenzoate reference standard (HCD 60%). **(M)** Normalized extracted ion chromatograms of NP343b in POCIS extracts (retention time = 21.03 min) and lauramidopropyl betaine reference standard (retention time = 21.10 min). **(N)** Head-to-tail plot of dd-MS2 spectra of NP343b in POCIS extracts (HCD 60%) and lauramidopropyl betaine reference standard (HCD 60%). **(O)** Normalized extracted ion chromatograms of NP361 in POCIS extracts (retention time = 21.77 min) and tributyl citrate reference standard (retention time = 21.86 min). **(P)** Head-to-tail plot of dd-MS2 spectra of NP361 in POCIS extracts (HCD 60%) and tributyl citrate reference standard (HCD 60%). Further compound information is summarized in Table S15.

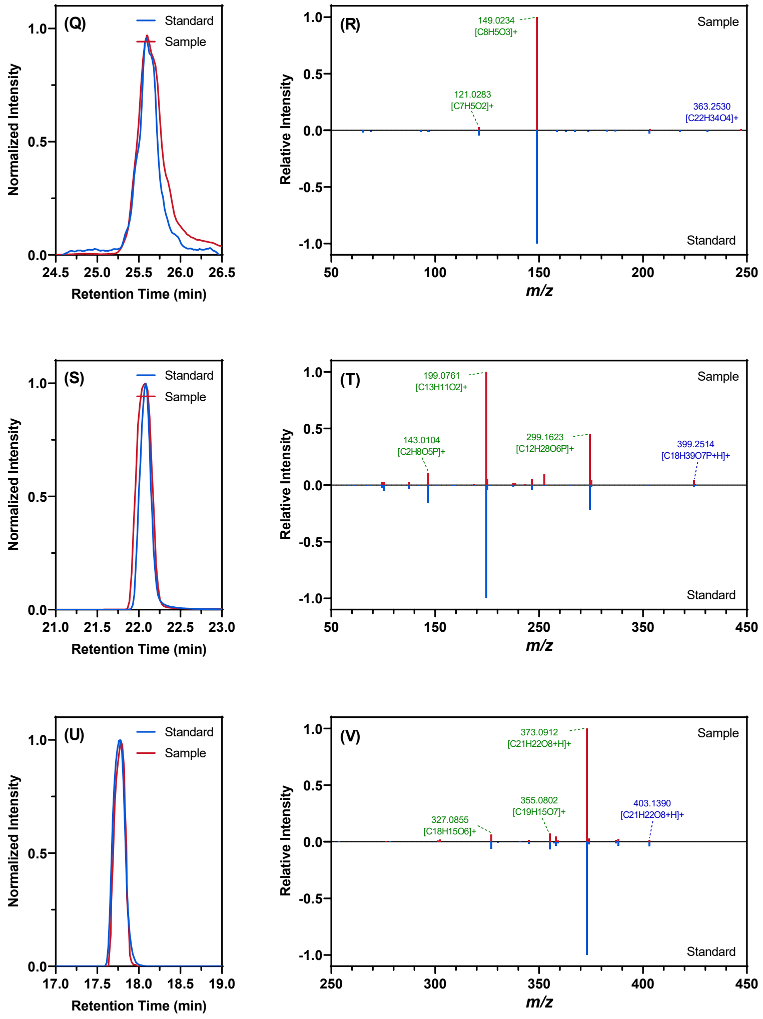

Figure S6c. Level 1 confirmation of 11 nontarget compounds: **(Q)** Normalized extracted ion chromatograms of NP363 in POCIS extracts (retention time = 25.60 min) and diheptyl phthalate reference standard (retention time = 25.59 min). **(R)** Head-to-tail plot of dd-MS2 spectra of NP363 in POCIS extracts (HCD 60%) and diheptyl phthalate reference standard (HCD 60%). **(S)** Normalized extracted ion chromatograms of NP399 in POCIS extracts (retention time = 22.05 min) and tris(2-butoxyethyl) phosphate reference standard (retention time = 22.08 min). **(T)** Head-to-tail plot of dd-MS2 spectra of NP399 in POCIS extracts (HCD 60%) and tris(2-butoxyethyl) phosphate reference standard (HCD 60%). **(U)** Normalized extracted ion chromatograms of NP403 in POCIS extracts (retention time = 17.81 min) and nobiletin reference standard (retention time = 17.78 min). **(V)** Head-to-tail plot of dd-MS2 spectra of NP403 in POCIS extracts (HCD 60%) and nobiletin reference standard (HCD 60%). Further compound information is summarized in Table S15.

S7. Field sampling rates for OMPs

Table S16. Summary of field sampling rates for 37 OMPs

Compound Name	Category[a]	R_s (L/d)	R^2	Log*D* (at pH 7.84)[b]	Cluster[c]
Caffeine	PHAR	0.016±0.002	0.974	-0.55	C
Atrazine-desisopropyl	PEST TP	0.022±0.004	0.927	1.12	A
Amantadine	PHAR	0.022±0.005	0.953	-1.18	C
2,4-D	PEST	0.028±0.004	0.937	-1.01	A
Irbesartan	PHAR	0.031±0.004	0.988	4.07	C
Sulfamethoxazole	PHAR	0.033±0.004	0.976	-0.09	C
Bupropion	PHAR	0.033±0.012	0.924	2.74	C
Benzoylecgonine	PHAR TP	0.033±0.010	0.935	-0.60	C
Abscisic Acid	PEST	0.034±0.009	0.968	-1.03	A
Sucralose	PCHI	0.036±0.003	0.981	-0.47	C
Metolachlor Oxanilic Acid (Metolachlor OA)	PEST TP	0.041±0.003	0.982	-0.62	A
Losartan	PHAR	0.041±0.007	0.978	3.66	C
Benzotriazole	PCHI	0.043±0.009	0.972	1.24	C
Lamotrigine	PHAR	0.049±0.004	0.974	1.92	C
Venlafaxine	PHAR	0.055±0.007	0.976	1.64	C
Hexa(methoxymethyl)melamine (HMMM)	PCHI	0.057±0.030	0.906	2.54	B
Methyl-1H-benzotriazole	PCHI	0.062±0.004	0.984	1.75	C
Trimethoprim	PHAR	0.068±0.006	0.990	1.21	C
Metoprolol	PHAR	0.070±0.006	0.986	-0.06	C
Atrazine-desethyl	PEST TP	0.073±0.004	0.987	1.54	A
Simazine	PEST	0.073±0.009	0.994	1.78	A
Cetirizine	PHAR	0.083±0.008	0.987	0.47	C
Desvenlafaxine	PHAR	0.084±0.010	0.992	1.48	C
Atrazine-2-hydroxy	PEST TP	0.087±0.007	0.969	-0.01	A
Benzothiazole	PCHI	0.090±0.013	0.915	2.11	C
Fexofenadine	PHAR	0.091±0.006	0.993	2.92	C
Tris(2-butoxyethyl) Phosphate (TBEP)	PCHI	0.094±0.006	0.981	3.94	C
Galaxolidone	PCHI TP	0.118±0.006	0.989	4.65	C
Lidocaine	PHAR	0.120±0.010	0.974	2.58	C
DEET	PCHI	0.128±0.013	0.970	2.50	B
Prometon	PEST	0.136±0.031	0.918	2.22	A
Carbamazepine	PHAR	0.141±0.014	0.985	2.77	C
Atrazine	PEST	0.146±0.009	0.981	2.20	A
Metolachlor	PEST	0.154±0.015	0.958	3.45	A
Metalaxyl	PEST	0.164±0.023	0.968	2.12	A
Metolachlor Ethanesulfonic Acid (Metolachlor ESA)	PEST TP	0.185±0.014	0.977	-0.26	A
Imidacloprid	PEST	0.216±0.037	0.968	0.84	B

[a] "PHAR" = pharmaceutical, "PEST" = pesticide, "PCHI" = personal care, household and industrial chemical, "TP" = transformation product; [b] Calculated by *JChem for Excel (Version 20.2.0.589)*[38]; [c] Assigned based on Figures 2 and 3 in the *Main Text*.

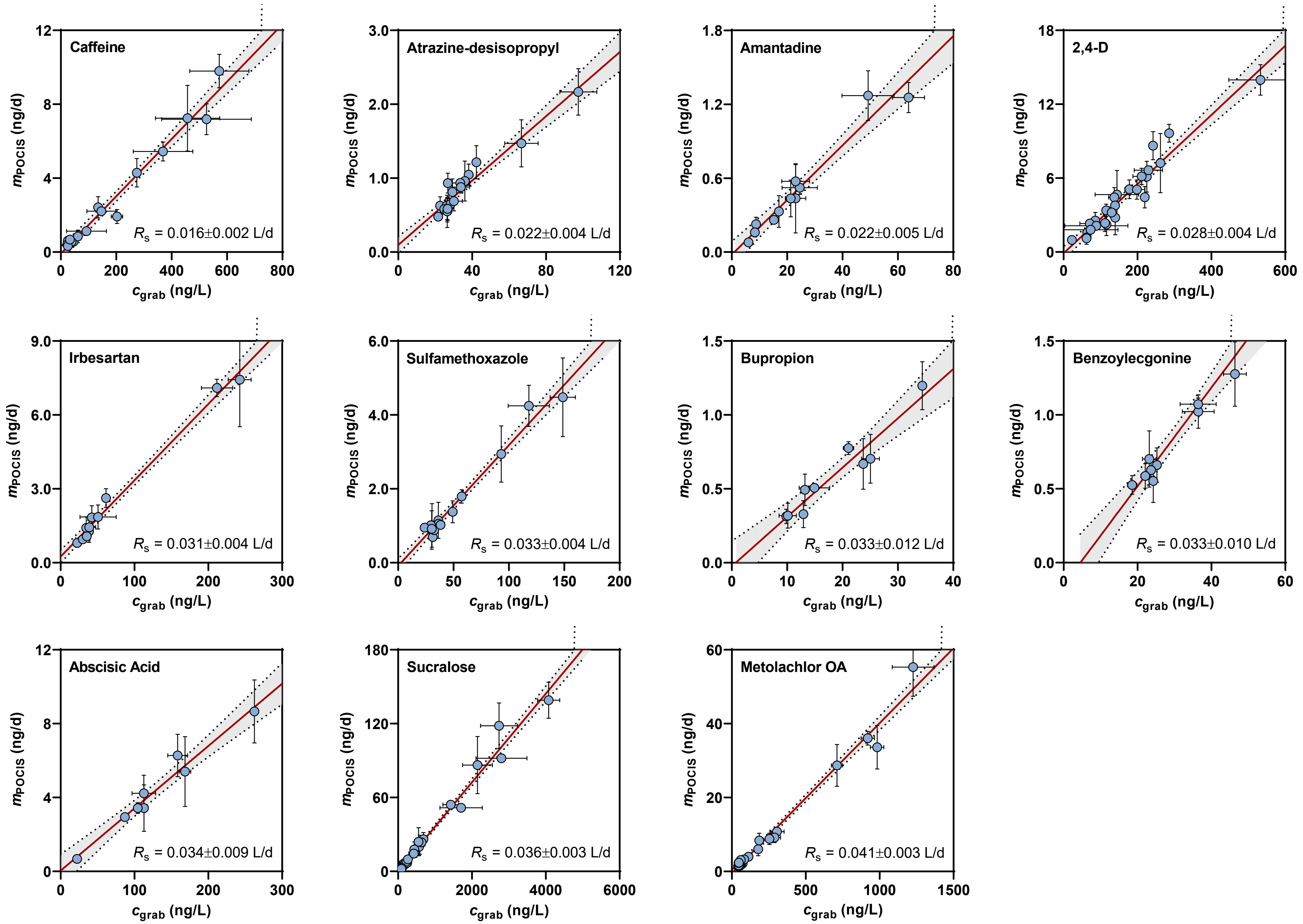

Figure S7a. Field sampling rates (R_s) for 37 OMPs. Compound-specific field R_s are summarized in Table S16.

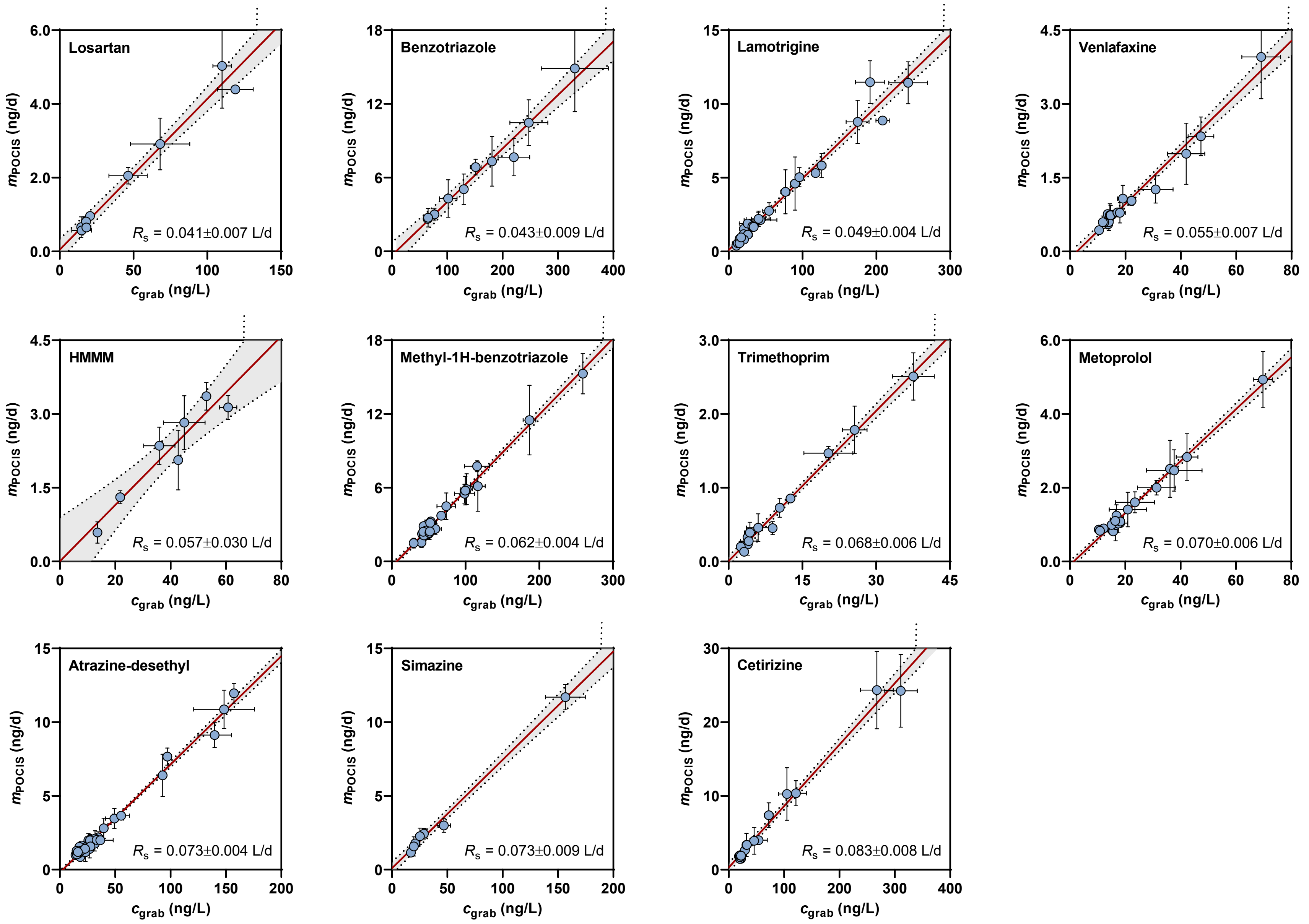

Figure S7b. Field sampling rates (R_s) for 37 OMPs. Compound-specific field R_s are summarized in Table S16.

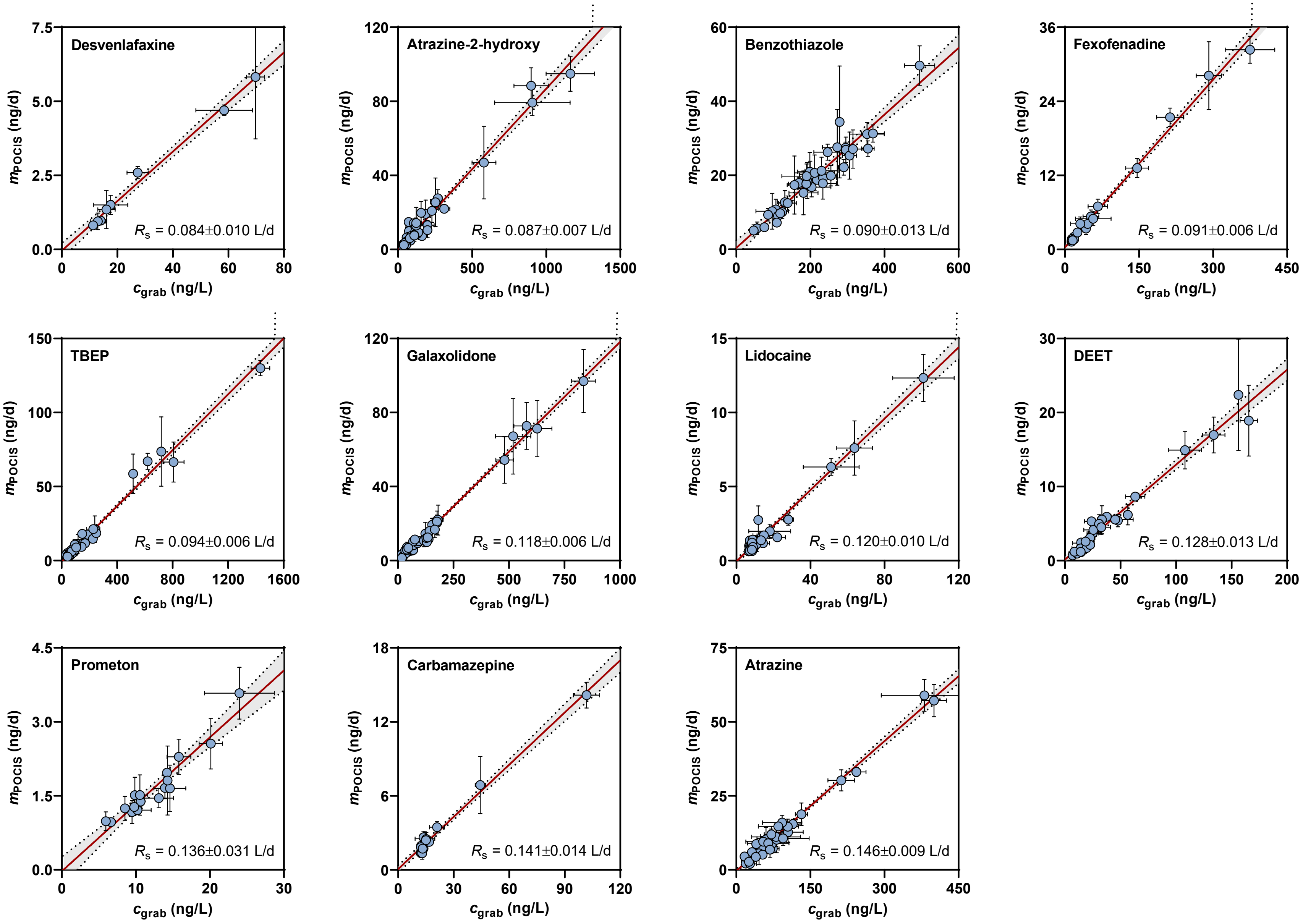

Figure S7c. Field sampling rates (R_s) for 37 OMPs. Compound-specific field R_s are summarized in Table S16.

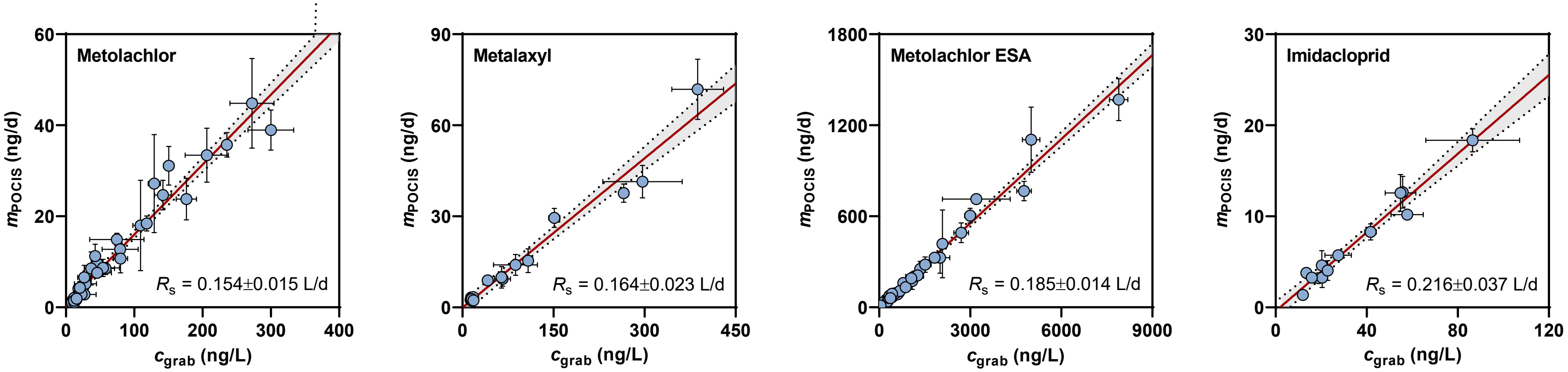

Figure S7d. Field sampling rates (R_s) for 37 OMPs: Caffeine; Atrazine-desisopropyl; Amantadine; 2,4-D; Irbesartan; Sulfamethoxazole; Bupropion; Benzoylecgonine; Abscisic Acid; Sucralose; Metolachlor Oxanilic Acid (Metolachlor OA); Losartan; Benzotriazole; Lamotrigine; Venlafaxine; Hexa(methoxymethyl)melamine (HMMM); Methyl-1H-benzotriazole; Trimethoprim; Metoprolol; Atrazine-desethyl; Simazine; Cetirizine; Desvenlafaxine; Atrazine-2-hydroxy; Benzothiazole; Fexofenadine; Tris(2-butoxyethyl) Phosphate (TBEP); Galaxolidone; Lidocaine; DEET; Prometon; Carbamazepine; Atrazine; Metolachlor; Metalaxyl; Metolachlor Ethanesulfonic Acid (Metolachlor ESA); Imidacloprid. On each plot, the red solid line represents the slope derived from the linear least squares regression analysis of m_{POCIS} versus c_{grab}. The black dotted lines bracket the 95% confidence interval for the slope. Error bars on individual data points represent the standard deviations of replicate measurements. Compound-specific field R_s are summarized in Table S16.

Comparing field R_s from this work (**Table S16**) with literature values **(Table S17**) was inherently challenging given the diverse exposure conditions and varying sampler designs among studies. Nevertheless, field R_s calculated herein generally fell within the same ranges of R_s determined by other large-scale *in situ* calibration studies such as those employing Chemcatchers[39] to monitor OMPs in agricultural and urban-impacted rivers in Switzerland (i.e., 0.01-0.4 L/d with a median of 0.07 L/d)[40] and wastewater effluents in England (i.e., 0.011-0.326 L/d with a median of 0.052 L/d),[41] respectively. Compound-wise comparisons of POCIS-based field R_s, although only feasible for a subset of 11 OMPs, further confirmed that values determined in this work were broadly comparable to those reported by previous studies. For example, the field R_s for carbamazepine (i.e., 0.141±0.014 L/d), lidocaine (i.e., 0.120±0.010 L/d), and metoprolol (i.e., 0.070±0.006 L/d) resembled the values (i.e., 0.154 L/d, 0.095 L/d, and 0.11 L/d, respectively) measured by POCIS at 14 stream sites in Luxembourg.[42] The field R_s for atrazine (i.e., 0.146±0.009 L/d) overlapped with the mean values (i.e., 0.152±0.075 L/d; n = 149) measured at headwater streams in nine U.S. agricultural watersheds[1] as well as the mean values (i.e., 0.229±0.119 L/d; n = 8) measured at 24 tributary sites in the South Nation River watershed in Canada.[43] The field R_s for atrazine-desethyl (i.e., 0.073±0.004 L/d), metalaxyl (i.e., 0.164±0.023 L/d), and metolachlor (i.e., 0.154±0.015 L/d) also compared well with the values (i.e., 0.09±0.02 L/d, 0.19±0.05 L/d, and 0.17±0.05 L/d, respectively) reported for two monitoring stations on the Marque River in France.[44] Likewise, the field R_s for metolachlor ESA (i.e., 0.185±0.014 L/d) was slightly higher than the value (i.e., 0.15 L/d) determined at five stream sites within the USDA Long-Term Agroecosystem Research network,[45] whereas the field R_s for imidacloprid (i.e., 0.216±0.037 L/d) was slightly lower than the value (i.e., 0.28 L/d) determined at two monitoring stations on agriculture-impacted streams in southern Sweden.[46] Lastly, the field R_s for atrazine-desisopropyl (i.e., 0.022±0.004 L/d) and simazine (i.e., 0.073±0.009 L/d) matched the values (i.e., 0.025±0.002 L/d and 0.063±0.009 L/d, respectively) measured during *in situ* calibration of POCIS in River Le Ruiné in France.[47]

Similar to prior studies,[40, 41] field R_s for these 37 OMPs only exhibited a weak, albeit statistically significant, correlation with their Log$D$ values[38] at the mean stream water pH of 7.84 (**Table S3**). Nevertheless, the slope

(i.e., 0.011±0.005) and *y*-intercept (i.e., 0.065±0.011) of the linear regression line between field R_s and Log*D* (**Figure S8**) were similar to those of the linear regression curve (i.e., 0.016±0.003 and 0.049±0.005, respectively) derived for OMPs via Chemcatcher in Swiss rivers,[40] which suggested some level of convergence in these empirical relationships despite differences in sampler designs and stream conditions. Fully understanding the uptake dynamics of OMPs by POCIS in streams would require field-based investigations if logistically feasible,[1] but the R_s-Log*D* relationship established herein should enable an initial estimate of field R_s for polar and semi-polar OMPs when such data are not available. Considering the spatiotemporal extent of our sampling regime, field R_s for the 37 OMPs may find applicability in future studies employing POCIS for OMP monitoring at streams sites with similar characteristics, but these values should also be evaluated with reference to the large database of sampling rates derived using laboratory calibration systems under field-relevant exposure conditions.

Comparing the limits of quantification (LOQs) of 37 OMPs in POCIS and grab samples also revealed that the median LOQs in POCIS (i.e., 2.3 ng/L) calculated based on field R_s and an average deployment period of 23 days was lower than that (i.e., 7.9 ng/L) determined based on the SPE of 500 mL stream water samples (Wilcoxon matched-pair signed rank test $p < 0.0001$; **Figure S9**). However, these LOQs were specific to the sampling and analytical conditions applied in this work and did not necessarily capture the performance disparity between POCIS and grab sampling.

Table S17. Literature-reported field sampling rates for OMPs				
Compound Name	R_s (L/d)	Sampler	Site / Location	Reference
2,4-D	0.02	Chemcatcher	Agricultural and urban impacted rivers (Furtbach, Limpach, Mentue, Salmsacher Aach, Surb), Switzerland	40
2,6-Dichlorbenzamide	0.06			
4-Acetamidoantipyrin	0.06			
4-Formylaminoantipyrin	0.09			
Acesulfame	0.0002			
Amisulpride	0.01			
Atenolol	0.01			
Atenolol Acid	0.003			
Atrazine-2-hydroxy	0.03			
Atrazine-desethyl	0.1			
Azoxystrobin Acid	0.07			
Benzotriazole	0.04			
Benzoylecgonine	0.03			
Bezafibrat	0.05			
Bicalutamide	0.1			
Candesartan	0.05			
Carbamazepine	0.1			
Carbamazepine-10,11-dihydro-10,11-dihydroxy	0.08			
Carbamazepine-10,11-epoxide	0.1			
Carbendazim	0.09			
Cetirizine	0.03			
Chloridazon-methyl-desphenyl	0.02			
Chlortoluron	0.09			
Clarithromycin	0.05			
Clindamycin	0.06			
Clopidogrel Carboxylic Acid	0.03			
Diazinon	0.1			
Diclofenac	0.06			
DEET (diethyltoluamide)	0.1			
Dimethachlor	0.1			
Dimethenamid	0.1			
Dimethoat	0.1			
Dimethomorph	0.08			
Diuron	0.1			
EDDP (2-ethylidene-1,5-dimethyl-3,3-diphenylpyrrolidin)	0.03			
Epoxiconazole	0.08			
Ethofumesate	0.08			
Fenamidone	0.2			
Fenhexamid	0.4			
Fenofibric Acid	0.08			
Fipronil	0.1			
Fluconazole	0.09			
Flufenacet	0.1			
Flufenacet-ESA	0.02			
Gabapentin	0.005			
Hydrochlorothiazide	0.05			
Indomethacine	0.07			
Isoproturon	0.08			
Lamotrigine	0.07			
Levamisol	0.1			
Levetiracetam	0.02			
Lidocaine	0.09			
Mecoprop	0.03			
Mefenamic Acid	0.06			
Metamitron	0.06			
Metamitron-desamino	0.05			
Metazachlor	0.2			
Metazachlor-ESA	0.04			

Table S17. Literature-reported field sampling rates for OMPs (continued)				
Compound Name	R_s (L/d)	Sampler	Site / Location	Reference
Metformin	0.004	Chemcatcher	Agricultural and urban impacted rivers (Furtbach, Limpach, Mentue, Salmsacher Aach, Surb), Switzerland	40
Methyl-benzotriazole	0.05			
Metolachlor-ESA	0.04			
Metolachlor-morpholinon	0.1			
Metolachlor-OXA	0.03			
Metoprolol	0.004			
N4-Acetyl-Sulfamethoxazole	0.03			
Napropamide	0.1			
Naproxen	0.07			
O-Desvenlafaxine + Tramadol	0.009			
Oxazepam	0.1			
Pethoxamid	0.1			
Phenazone (Antipyrene)	0.08			
Pirimicarb	0.1			
Prometryn + Terbutryn	0.1			
Propachlor	0.2			
Propazin-2-hydroxy + Terbutylazin-2-hydroxy	0.04			
Propiconazole	0.1			
Sitagliptin	0.09			
S-Metolachlor	0.1			
Sotalol	0.01			
Sucralose	0.03			
Sulfamethazine	0.1			
Sulfamethoxazole	0.04			
Sulfapyridine	0.1			
Tebuconazole	0.09			
Terbutylazin-desethyl	0.08			
Thiamethoxam	0.1			
Trimethoprim	0.03			
Venlafaxine	0.01			
Benzophenone-3	0.011	Chemcatcher	Wastewater treatment work, Southwest England	41
Benzophenone-4	0.049			
Methylparaben	0.055			
Propylparaben	0.068			
Bisphenol-A	0.031			
E1	0.071			
Sulfasalazine	0.154			
Clarithromycin	0.024			
Azithromycin	0.024			
Trimethoprim	0.028			
Sulfamethoxazole	0.058			
Valsartan	0.06			
Irbesartan	0.087			
Lisinopril	0.059			
Ketoprofen	0.037			
Ibuprofen	0.048			
Naproxen	0.048			
Diclofenac	0.044			
Bezafibrate	0.042			
Atorvastatin	0.013			
Fexofenadine	0.059			
Cetirizine	0.039			
Gliclazide	0.045			
Atenolol	0.034			
Metoprolol	0.05			
Propranolol	0.114			
Ranitidine	0.043			
Cimetidine	0.085			

Table S17. Literature-reported field sampling rates for OMPs (continued)				
Compound Name	R_s (L/d)	Sampler	Site / Location	Reference
Ephedrine/Pseudoephedrine	0.044	Chemcatcher	Wastewater treatment work, Southwest England	41
Venlafaxine	0.065			
Fluoxetine	0.032			
Sertraline	0.116			
Mirtazapine	0.074			
Citalopram	0.069			
Desmethylcitalopram	0.149			
Carbamazepine	0.045			
10,11-Dihydro-10-hydroxycarbamazepine	0.077			
Diltiazem	0.185			
Temazepam	0.326			
Quetiapine	0.107			
Nicotine	0.234			
Caffeine	0.037			
Cotinine	0.041			
1,7-Dimethylxantine	0.052			
Morphine	0.031			
Normorphine	0.056			
Methadone	0.226			
EDDP	0.056			
Codeine	0.056			
Norcodeine	0.052			
Dihydrocodeine	0.047			
Tramadol	0.047			
N-Desmethyltramadol	0.087			
O-Desmethyltramadol	0.023			
Amphetamine	0.028			
Methamphetamine	0.025			
MDMA	0.074			
Cocaine	0.061			
Benzoylecgonine	0.031			
Carbamazepine	0.154	POCIS	Wastewater impacted rivers (Chiers, Alzette, Eisch, Mamer, Attert, Sure, Clerve, Wiltz, Ernz blanche, Syr), Luxembourg	42
Diclofenac	0.06			
Lidocaine	0.095			
Erythromycin	0.077			
Metoprolol	0.11			
Diuron	0.05			
Carbendazim	0.128			
Terbutryn	0.119			
Atrazine	0.124	POCIS	South Nation River watershed tributaries with a gradient of agricultural intensity, Canada	43
Atrazine	0.094			
Atrazine	0.143			
Atrazine	0.131			
Atrazine	0.349			
Atrazine	0.264			
Atrazine	0.334			
Atrazine	0.391			
Metolachlor ESA	0.15	POCIS	Long-Term Agroecosystem Research (LTAR) network rivers and creeks, U.S.	45
Atrazine	0.22	POCIS	Marque River (wastewater and stormwater impacted section), France	44
Atrazine-desethyl	0.09			
Atrazine-desisopropyl	0.09			
Metalaxyl	0.19			
Metolachlor	0.17			
Cyprodinil	0.22			
Diclofenac	0.08			
Dimethenamid	0.2			
Isoproturon	0.16			

Table S17. Literature-reported field sampling rates for OMPs (continued)				
Compound Name	R_s (L/d)	Sampler	Site / Location	Reference
Atrazine	0.059	POCIS	River Le Ruiné, France	47
Atrazine-desethyl	0.061			
Atrazine-desisopropyl	0.025			
Simazine	0.063			
Terbuthylazine-desethyl	0.075			
Atrazine	0.013	POCIS	Agricultural impacted streams, southern Sweden	46
Desethylatrazine	0.035			
Azoxystrobin	0.047			
2,6-Dichlorobenzamide (BAM)	0.046			
Bentazone	0.008			
Chloridazon	0.017			
Cyazofamid	0.066			
Cyprodinil	0.013			
Ethofumesate	0.0061			
Imidacloprid	0.28			
Isoproturon	0.35			
Mandipropamid	0.003			
MCPA	0.017			
Mecoprop	0.047			
Metalaxyl	0.084			
Metamitron	0.0013			
Metazachlor	0.088			
Metribuzin	0.005			
Picoxystrobin	0.023			
Propamocarb	0.85			
Propiconazole	0.04			
Terbuthylazine	0.069			
Desethyl-terbuthylazine	0.01			
Atrazine	0.0094	Chemcatcher	Agricultural impacted streams, southern Sweden	46
Atrazine-desethyl	0.0015			
Atrazine-desisopropyl	0.0091			
Chloridazon	0.00016			
Cyazofamid	0.03			
Cyprodinil	0.006			
Ethofumesate	0.0092			
Imidacloprid	0.0018			
Isoproturona	0.06			
Metalaxyl	0.03			
Metazachlor	0.04			
Metribuzin	0.0063			
Picoxystrobin	0.0082			
Propamocarb	0.0013			
Atrazine	0.24	POCIS	Headwater streams in Iowa, Louisiana, Missouri, Nebraska, and Texas, U.S.	1
Atrazine	0.132			
Atrazine	0.175			
Atrazine	0.201			
Atrazine	0.147			
Atrazine	0.189			
Atrazine	0.171			
Atrazine	0.178			
Atrazine	0.088			
Atrazine	0.015			
Atrazine	0.429			
Atrazine	0.158			
Atrazine	0.219			
Atrazine	0.091			
Atrazine	0.167			
Atrazine	0.141			

Table S17. Literature-reported field sampling rates for OMPs (continued)				
Compound Name	R_s (L/d)	Sampler	Site / Location	Reference
Atrazine	0.131	POCIS	Headwater streams in Iowa, Louisiana, Missouri, Nebraska, and Texas, U.S.	1
Atrazine	0.163			
Atrazine	0.041			
Atrazine	0.17			
Atrazine	0.129			
Atrazine	0.214			
Atrazine	0.174			
Atrazine	0.06			
Atrazine	0.267			
Atrazine	0.139			
Atrazine	0.183			
Atrazine	0.181			
Atrazine	0.055			
Atrazine	0.19			
Atrazine	0.251			
Atrazine	0.221			
Atrazine	0.241			
Atrazine	0.099			
Atrazine	0.161			
Atrazine	0.358			
Atrazine	0.057			
Atrazine	0.118			
Atrazine	0.075			
Atrazine	0.075			
Atrazine	0.03			
Atrazine	0.11			
Atrazine	0.136			
Atrazine	0.144			
Atrazine	0.112			
Atrazine	0.128			
Atrazine	0.115			
Atrazine	0.134			
Atrazine	0.112			
Atrazine	0.082			
Atrazine	0.107			
Atrazine	0.095			
Atrazine	0.062			
Atrazine	0.03			
Atrazine	0.148			
Atrazine	0.187			
Atrazine	0.258			
Atrazine	0.152			
Atrazine	0.074			
Atrazine	0.044			
Atrazine	0.171			
Atrazine	0.087			
Atrazine	0.118			
Atrazine	0.08			
Atrazine	0.261			
Atrazine	0.169			
Atrazine	0.2			
Atrazine	0.097			
Atrazine	0.141			
Atrazine	0.086			
Atrazine	0.16			
Atrazine	0.16			
Atrazine	0.124			
Atrazine	0.121			

Table S17. Literature-reported field sampling rates for OMPs (continued)				
Compound Name	R_s (L/d)	Sampler	Site / Location	Reference
Atrazine	0.113	POCIS	Headwater streams in Iowa, Louisiana, Missouri, Nebraska, and Texas, U.S.	1
Atrazine	0.106			
Atrazine	0.198			
Atrazine	0.147			
Atrazine	0.145			
Atrazine	0.172			
Atrazine	0.121			
Atrazine	0.197			
Atrazine	0.151			
Atrazine	0.142			
Atrazine	0.099			
Atrazine	0.116			
Atrazine	0.151			
Atrazine	0.089			
Atrazine	0.143			
Atrazine	0.221			
Atrazine	0.173			
Atrazine	0.076			
Atrazine	0.149			
Atrazine	0.136			
Atrazine	0.127			
Atrazine	0.089			
Atrazine	0.126			
Atrazine	0.088			
Atrazine	0.112			
Atrazine	0.208			
Atrazine	0.193			
Atrazine	0.238			
Atrazine	0.124			
Atrazine	0.147			
Atrazine	0.133			
Atrazine	0.11			
Atrazine	0.063			
Atrazine	0.071			
Atrazine	0.14			
Atrazine	0.112			
Atrazine	0.418			
Atrazine	0.499			
Atrazine	0.417			
Atrazine	0.296			
Atrazine	0.138			
Atrazine	0.19			
Atrazine	0.189			
Atrazine	0.16			
Atrazine	0.157			
Atrazine	0.101			
Atrazine	0.157			
Atrazine	0.116			
Atrazine	0.255			
Atrazine	0.137			
Atrazine	0.252			
Atrazine	0.244			
Atrazine	0.138			
Atrazine	0.127			
Atrazine	0.149			
Atrazine	0.163			
Atrazine	0.175			
Atrazine	0.112			

**Table S17.** Literature-reported field sampling rates for OMPs (continued)				
Compound Name	R_s (L/d)	Sampler	Site / Location	Reference
Atrazine	0.075	POCIS	Headwater streams in Iowa, Louisiana, Missouri, Nebraska, and Texas, U.S.	1
Atrazine	0.165			
Atrazine	0.164			
Atrazine	0.153			
Atrazine	0.124			
Atrazine	0.065			
Atrazine	0.052			
Atrazine	0.089			
Atrazine	0.115			
Atrazine	0.251			
Atrazine	0.101			
Atrazine	0.177			
Atrazine	0.112			
Atrazine	0.15			
Atrazine	0.147			
Atrazine	0.219			
Atrazine	0.175			

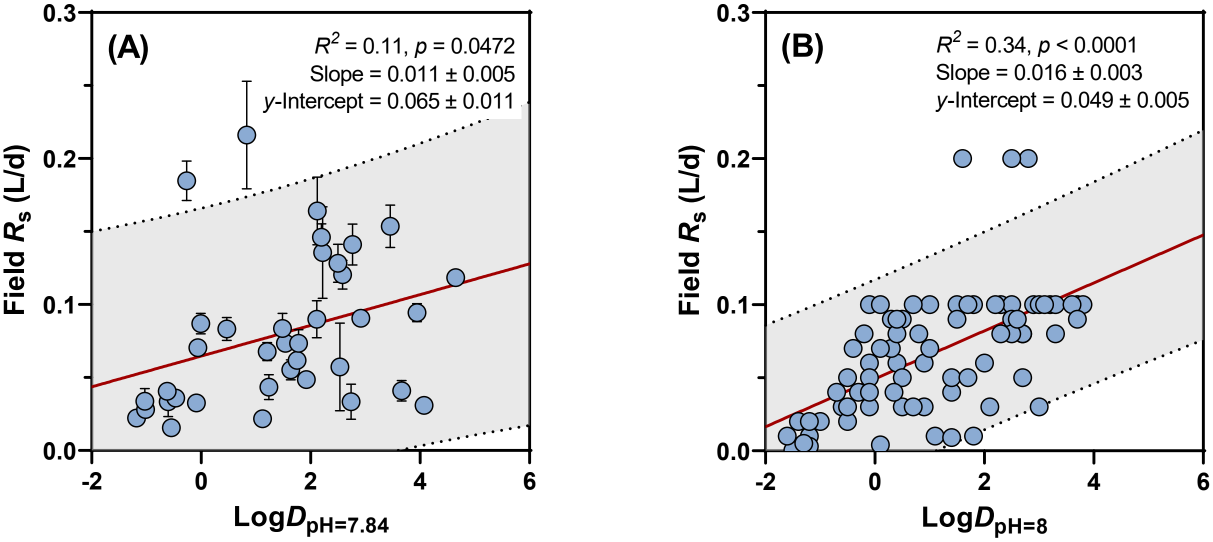

Figure S8. Correlation between field R_s and Log$D$: **(A)** Correlation between field R_s and Log$D_{pH=7.84}$. Error bars represent the 95% confidence intervals of field R_s. The red solid line represents the slope derived from the linear least squares regression analysis of field R_s versus Log$D_{pH=7.84}$ (n = 37). The black dotted lines represent the 95% prediction bands for the slope. Compound-specific field R_s (i.e., calculated as the slopes derived from the linear least squares regression analysis of m_{POCIS} versus c_{grab}; Figure S7) are summarized in Table S16. **(B)** Correlation between field R_s and Log$D_{pH=8}$ reproduced from data reported in Moschet *et al.*[40] The red solid line represents the slope derived from the linear least squares regression analysis of field R_s versus Log$D_{pH=8}$ (n = 86; excluding data for two outliers fenhexamid and metformin). The black dotted lines represent the 95% prediction bands for the slope.

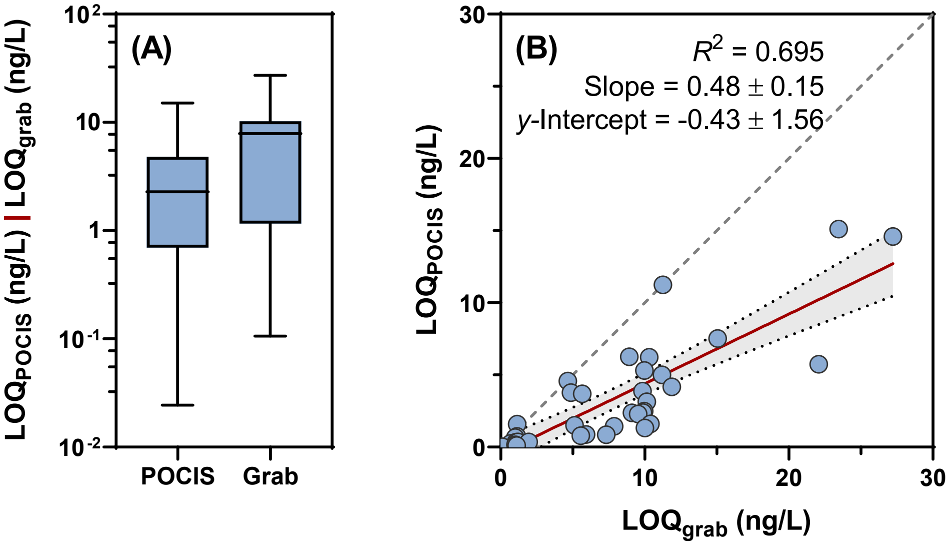

Figure S9. Comparison of the limits of quantification (LOQs) of 37 OMPs in POCIS and grab samples: **(A)** Boxplots of the LOQs of 37 OMPs in POCIS (LOQ$_{POCIS}$ in ng/L; calculated based on field R_s in Table S16 and an average deployment period of 23 days) and the LOQs of 37 OMPs in grab samples (LOQ$_{grab}$ in ng/L; from Table S10). The whiskers and centerline mark the range (minimum to maximum) and median of LOQ$_{POCIS}$ or LOQ$_{grab}$, respectively. **(B)** Linear correlation between the LOQ$_{POCIS}$ and LOQ$_{grab}$ of 37 OMPs. The red solid line represents the slope derived from the linear least squares regression analysis of LOQ$_{POCIS}$ versus LOQ$_{grab}$. The black dotted lines bracket the 95% confidence interval for the slope. The grey dashed line represents the line of identity.

S8. Comparison of load estimation for OMPs by POCIS and grab samples

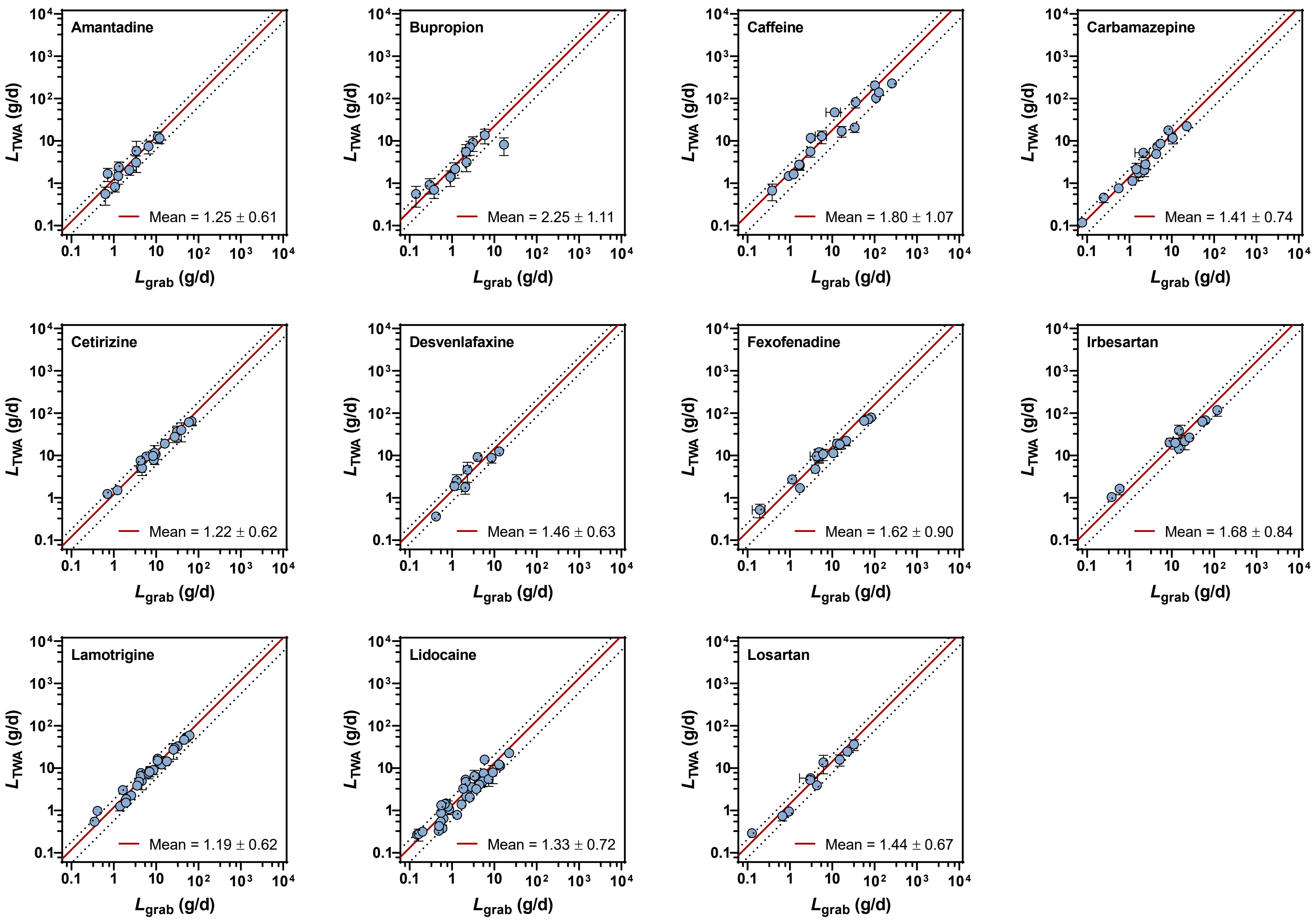

Figure S10a. Comparison of POCIS (L_{TWA}) versus grab samples (L_{grab}) for OMP load estimation on a compound basis (n = 37).

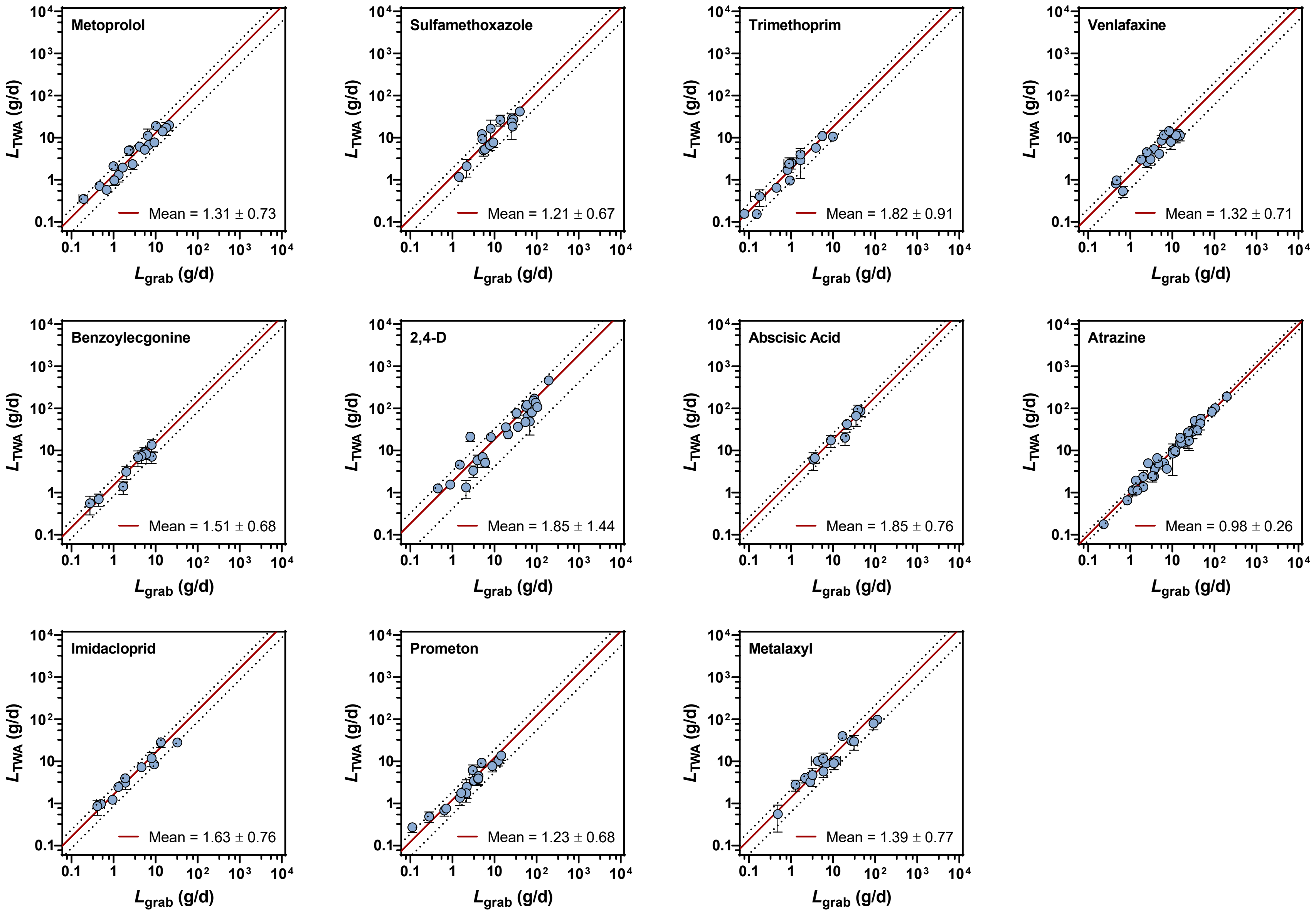

Figure S10b. Comparison of POCIS (L_{TWA}) versus grab samples (L_{grab}) for OMP load estimation on a compound basis (n = 37).

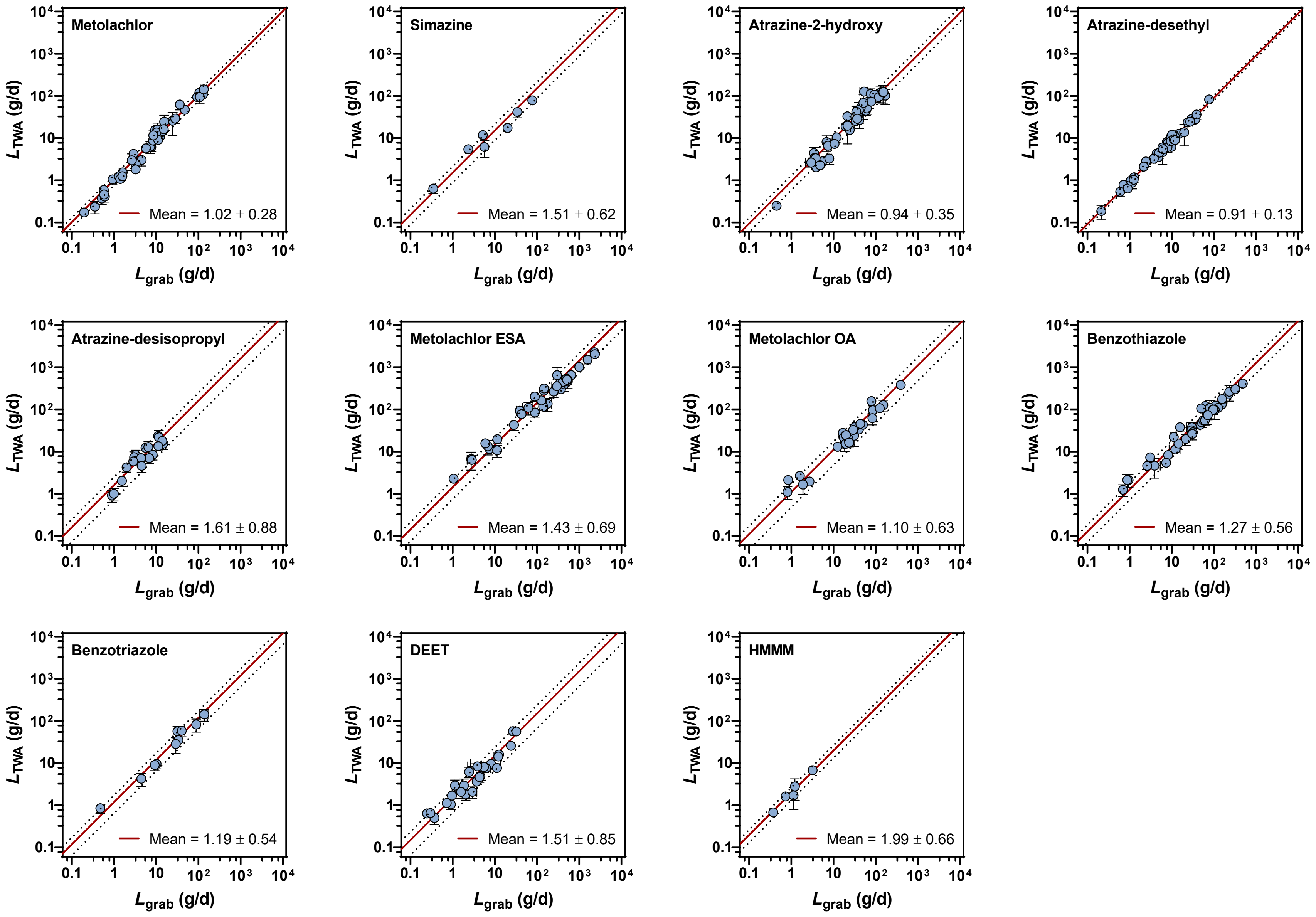

Figure S10c. Comparison of POCIS (L_{TWA}) versus grab samples (L_{grab}) for OMP load estimation on a compound basis (n = 37).

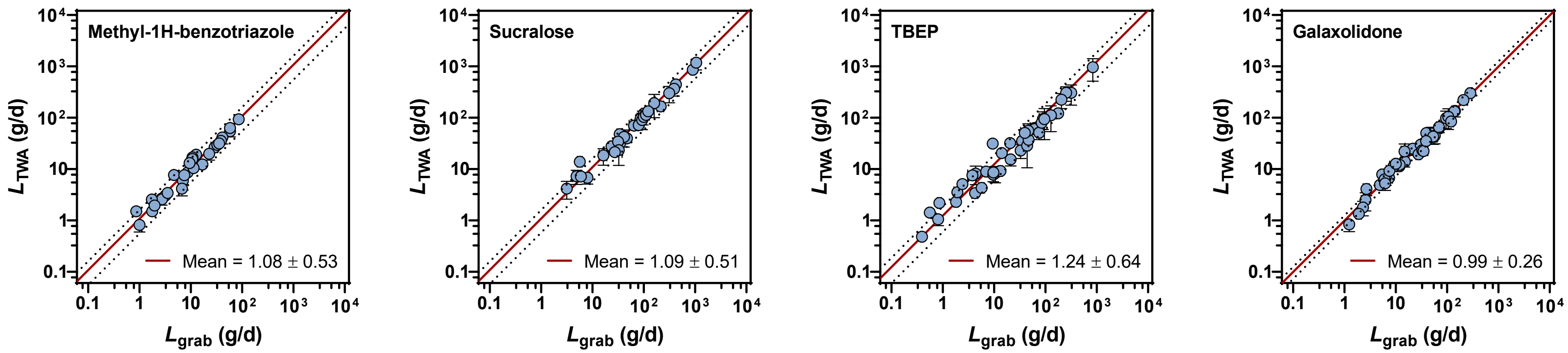

Figure S10d. Comparison of POCIS (L_{TWA}) versus grab samples (L_{grab}) for OMP load estimation on a compound basis (n = 37): Amantadine; Bupropion; Caffeine; Carbamazepine; Cetirizine; Desvenlafaxine; Fexofenadine; Irbesartan; Lamotrigine; Lidocaine; Losartan; Metoprolol; Sulfamethoxazole; Trimethoprim; Venlafaxine; Benzoylecgonine; 2,4-D; Abscisic Acid; Atrazine; Imidacloprid; Prometon; Metalaxyl; Metolachlor; Simazine; Atrazine-2-hydroxy; Atrazine-desethyl; Atrazine-desisopropyl; Metolachlor Ethanesulfonic Acid (Metolachlor ESA); Metolachlor Oxanilic Acid (Metolachlor OA); Benzothiazole; Benzotriazole; DEET; Hexa(methoxymethyl)melamine (HMMM); Methyl-1H-benzotriazole; Sucralose; Tris(2-butoxyethyl) Phosphate (TBEP); Galaxolidone. On each cross plot, the red solid line represents the mean L_{TWA}/L_{grab} ratio measured for an OMP. The black dotted lines bracket the standard deviation of L_{TWA}/L_{grab} ratios. Error bars on individual data points represent the standard deviations of replicate measurements.

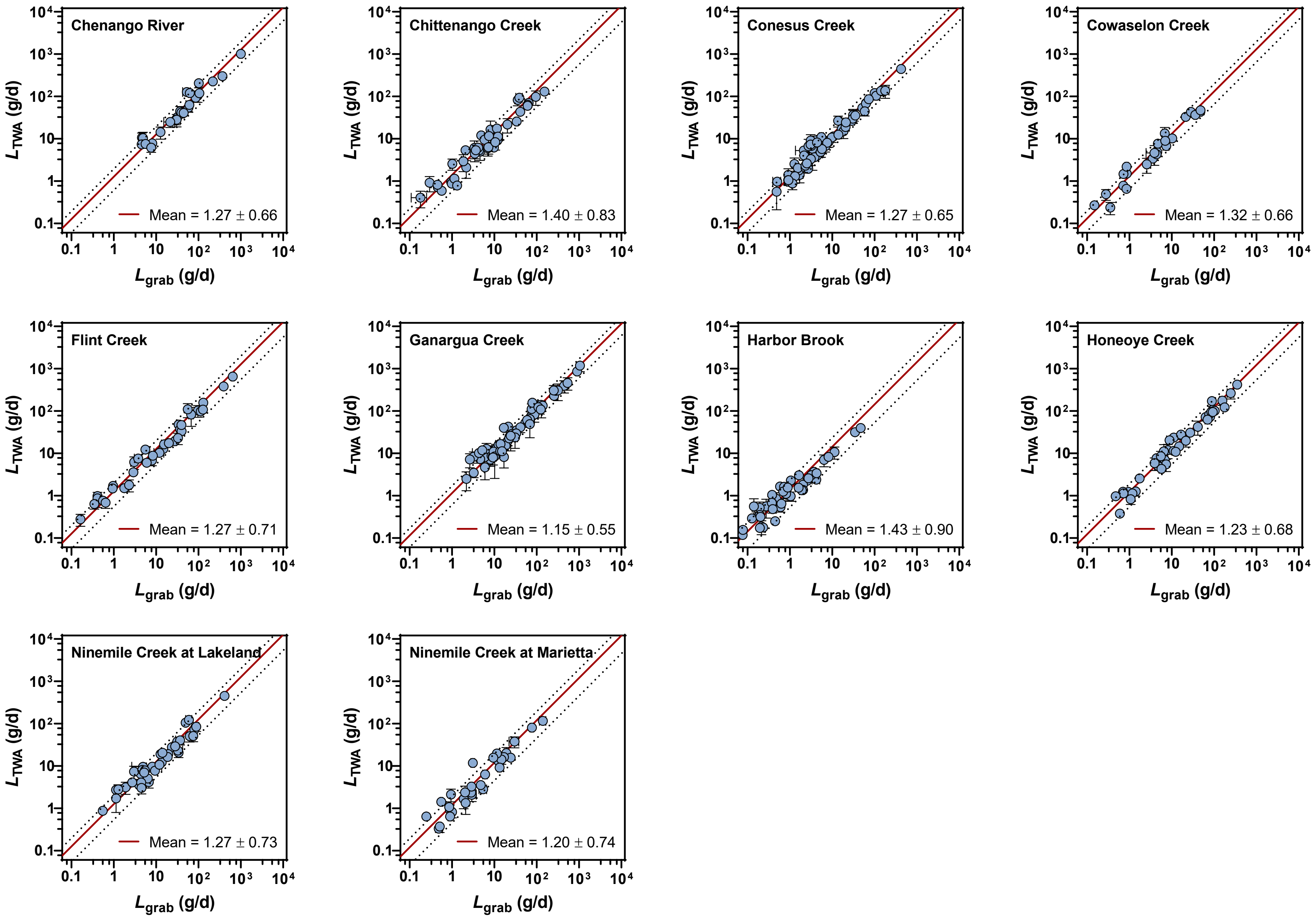

Figure S11a. Comparison of POCIS (L_{TWA}) versus grab samples (L_{grab}) for OMP load estimation on a stream site basis (n = 19).

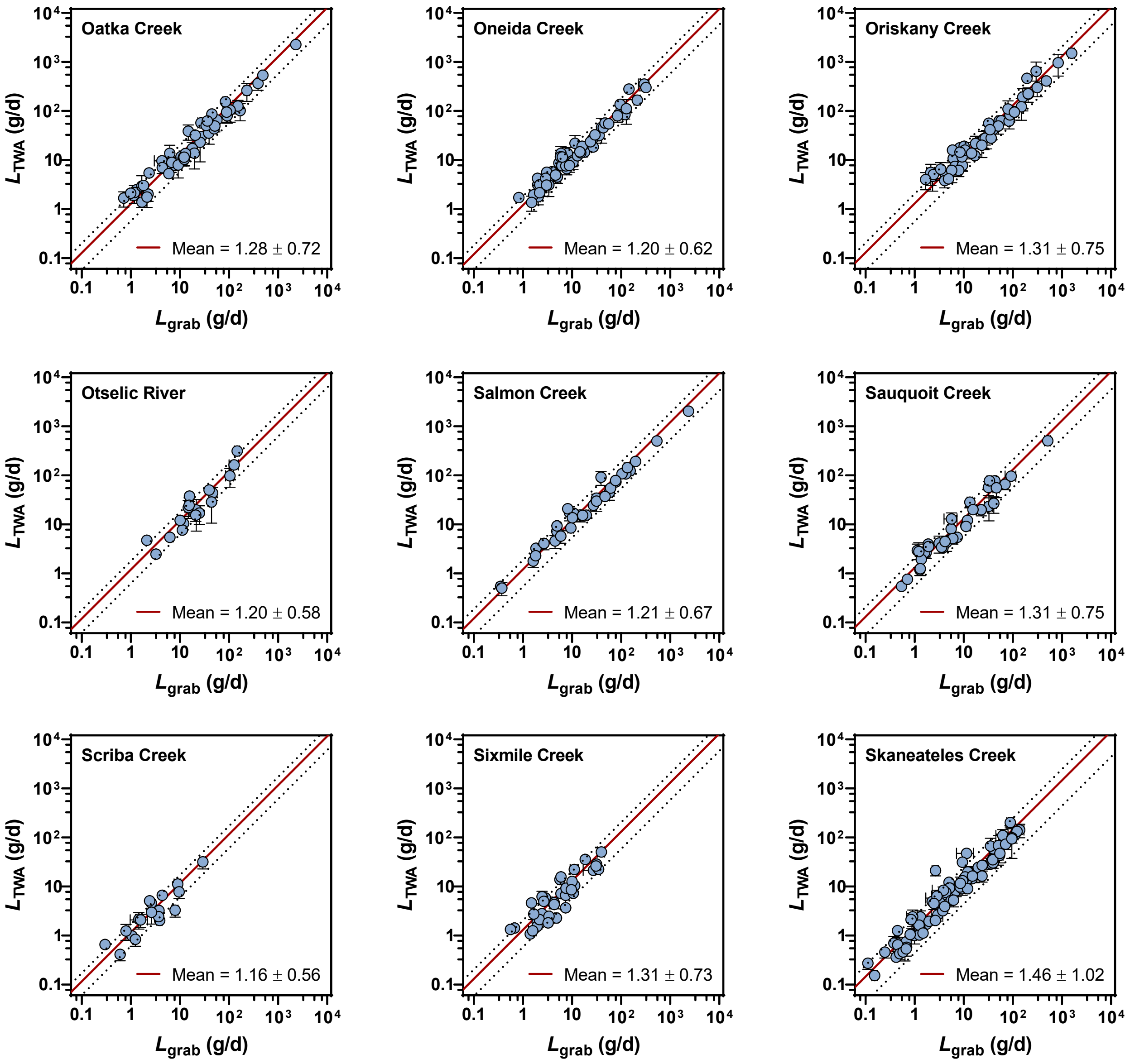

Figure S11b. Comparison of POCIS (L_{TWA}) versus grab samples (L_{grab}) for OMP load estimation on a stream site basis (*n* = 19): Chenango River; Chittenango Creek; Conesus Creek; Cowaselon Creek; Flint Creek; Ganargua Creek; Harbor Brook; Honeoye Creek; Ninemile Creek at Lakeland; Ninemile Creek at Marietta; Oatka Creek; Oneida Creek; Oriskany Creek; Otselic River; Salmon Creek; Sauquoit Creek. Scriba Creek; Sixmile Creek; Skaneateles Creek. Note that OMP loads at Butternut Creek were not calculated as the creek was not gauged during our study. On each cross plot, the red solid line represents the mean $L_{\mathrm{TWA}}/L_{\mathrm{grab}}$ ratio measured at a stream site. The black dotted lines bracket the standard deviation of $L_{\mathrm{TWA}}/L_{\mathrm{grab}}$ ratios. Error bars on individual data points represent the standard deviations of replicate measurements.

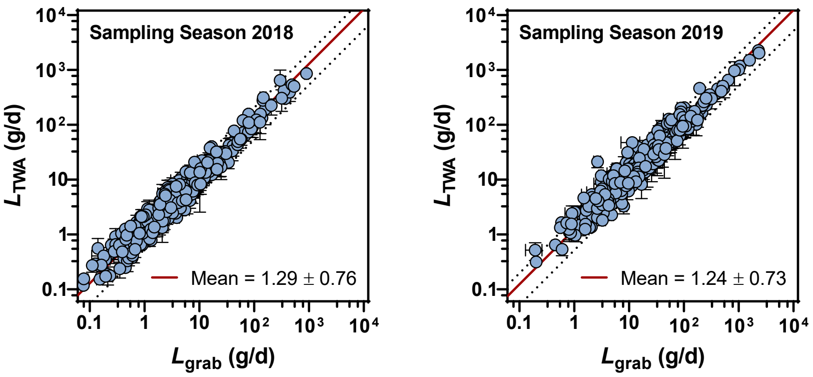

Figure S12. Comparison of POCIS (L_{TWA}) versus grab samples (L_{grab}) for OMP load estimation on a sampling season basis (i.e., 2018 and 2019). On each cross plot, the red solid line represents the mean L_{TWA}/L_{grab} ratio measured during a sampling season. The black dotted lines bracket the standard deviation of L_{TWA}/L_{grab} ratios. Error bars on individual data points represent the standard deviations of replicate measurements.

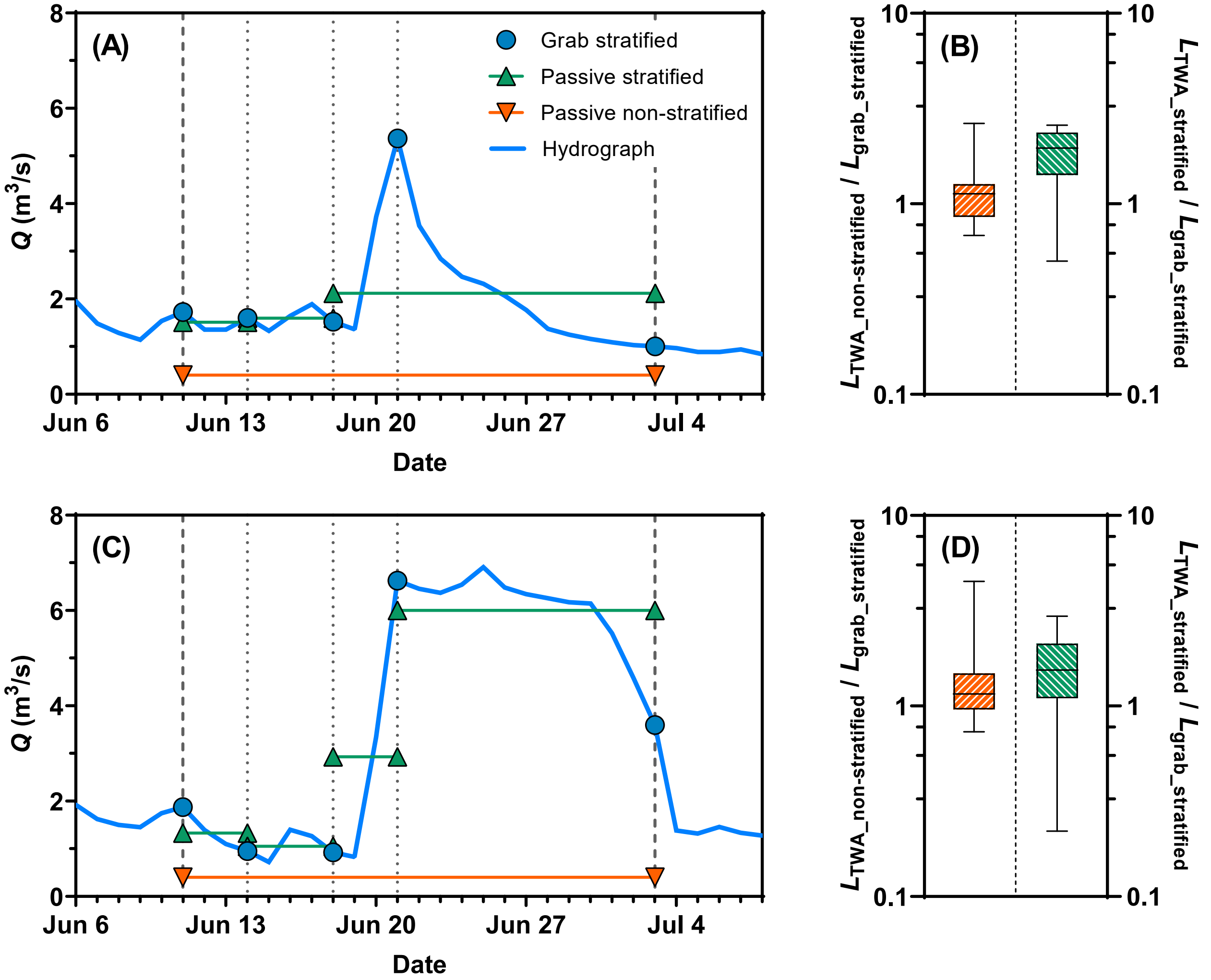

Figure S13. Comparison of stratified versus nonstratified passive sampling for OMP load estimation: **(A)** Hydrograph at Ninemile Creek at Marietta during the sampling event of June 11 to July 3, 2019. **(B)** Boxplots of the ratios of loads estimated by stratified ($L_{TWA\_stratified}$) or non-stratified ($L_{TWA\_non\text{-}stratified}$) passive sampling and stratified grab sampling ($L_{grab\_stratified}$) at Ninemile Creek at Marietta. Each box extends from the 25th to 75th percentiles. The whiskers extend down to the minimum and up to the maximum. The centerline in each box marks the median. **(C)** Hydrograph at Skaneateles Creek during the sampling event of June 11 to July 3, 2019. **(D)** Boxplots of the ratios of loads estimated by stratified ($L_{TWA\_stratified}$) or non-stratified ($L_{TWA\_non\text{-}stratified}$) passive sampling and stratified grab sampling ($L_{grab\_stratified}$) at Skaneateles Creek. Each box extends from the 25th to 75th percentiles. The whiskers extend down to the minimum and up to the maximum. The centerline in each box marks the median.

S9. Exposure-activity ratio (EAR) analysis

Table S18. ToxCast 3.2 entries for OMPs quantified in POCIS and grab samples

Compound Name	CAS	ToxCast Preferred Name	CAS
Abacavir	136470-78-5	Abacavir	136470-78-5
Acetaminophen	103-90-2	Acetaminophen	103-90-2
Aliskiren	173334-57-1	Aliskiren Hydrochloride	173399-03-6
Amantadine	768-94-5	Amantadine	768-94-5
Amitriptyline	50-48-6	Amitriptyline Hydrochloride	549-18-8
Atenolol	29122-68-7	R-(+)-Atenolol	56715-13-0
Azelaic Acid	123-99-9	Nonanedioic acid	123-99-9
Azithromycin	83905-01-5	Azithromycin	83905-01-5
Bamethan	3703-79-5	Bamethan	3703-79-5
Bisoprolol	66722-44-9	Bisoprolol Fumarate	104344-23-2
Bupropion	34911-55-2	Bupropion Hydrochloride	31677-93-7
Caffeine	58-08-2	Caffeine	58-08-2
Carbamazepine	298-46-4	Carbamazepine	298-46-4
Cetirizine	83881-51-0	Cetirizine Dihydrochloride	83881-52-1
Citalopram	59729-33-8	Citalopram Hydrobromide	59729-32-7
Clarithromycin	81103-11-9	Clarithromycin	81103-11-9
Clindamycin	18323-44-9	Clindamycin Hydrochloride	21462-39-5
Codeine	76-57-3	Codeine Hydrochloride	1422-07-7
Desipramine	50-47-5	Desipramine Hydrochloride	58-28-6
Desvenlafaxine (O-Desmethyl Venlafaxine)	93413-62-8	NA	NA
Dexpanthenol	81-13-0	Dexpanthenol	81-13-0
Dextromethorphan	125-71-3	Dextromethorphan Hydrobromide	125-69-9
Diazepam	439-14-5	Diazepam	439-14-5
Diltiazem	42399-41-7	Diltiazem Hydrochloride	33286-22-5
Diphenhydramine	58-73-1	Diphenhydramine Hydrochloride	147-24-0
Dopamine	51-61-6	Dopamine	51-61-6
Doxylamine	469-21-6	Doxylamine Succinate	562-10-7
Ephedrine	299-42-3	Ephedrine Hydrochloride	50-98-6
Fexofenadine	83799-24-0	Fexofenadine Hydrochloride	153439-40-8
Flecainide	54143-55-4	Flecainide Acetate	54143-56-5
Fluconazole	86386-73-4	Fluconazole	86386-73-4
Fluoxetine	54910-89-3	Fluoxetine Hydrochloride	56296-78-7
Gabapentin	60142-96-3	Gabapentin	60142-96-3
Gemfibrozil	25812-30-0	Gemfibrozil	25812-30-0
Griseofulvin	126-07-8	Griseofulvin	126-07-8
Guaifenesin	93-14-1	Guaifenesin	93-14-1
Irbesartan	138402-11-6	Irbesartan	138402-11-6
Labetalol	36894-69-6	Labetalol Hydrochloride	32780-64-6
Lamotrigine	84057-84-1	Lamotrigine	84057-84-1

**Table S18.** ToxCast 3.2 entries for OMPs quantified in POCIS and grab samples (continued)			
Compound Name	CAS	ToxCast Preferred Name	CAS
Levamisole	14769-73-4	Levamisole Hydrochloride	16595-80-5
Levetiracetam	102767-28-2	NA	NA
Levorphanol	77-07-6	NA	NA
Lidocaine	137-58-6	Lidocaine	137-58-6
Losartan	114798-26-4	Losartan	114798-26-4
Maprotiline	10262-69-8	Maprotiline	10262-69-8
Metaxalone	1665-48-1	Metaxalone	1665-48-1
Metformin	657-24-9	Metformin	657-24-9
Methocarbamol	532-03-6	Methocarbamol	532-03-6
Metoprolol	51384-51-1	Metoprolol	51384-51-1
Molindone	7416-34-4	Molindone Hydrochloride	15622-65-8
Mycophenolic Acid	24280-93-1	Mycophenolic Acid	24280-93-1
Nadolol	42200-33-9	Nadolol	42200-33-9
Naproxen	22204-53-1	Naproxen	22204-53-1
Oxcarbazepine	28721-07-5	Oxcarbazepine	28721-07-5
Phenytoin	57-41-0	5,5-Diphenylhydantoin	57-41-0
Prilocaine	721-50-6	Prilocaine Hydrochloride	1786-81-8
Propranolol	525-66-6	Propranolol Hydrochloride	318-98-9
Protriptyline	438-60-8	Protriptyline Hydrochloride	1225-55-4
Ranitidine	66357-35-5	Ranitidine	66357-35-5
Rimantadine	13392-28-4	Rimantadine Hydrochloride	1501-84-4
Sertraline	79617-96-2	Sertraline Hydrochloride	79559-97-0
Sitagliptin	486460-32-6	NA	NA
Sotalol	3930-20-9	Sotalol	3930-20-9
Sulfamethoxazole	723-46-6	Sulfamethoxazole	723-46-6
Sulfapyridine	144-83-2	Sulfapyridine	144-83-2
Telmisartan	144701-48-4	Telmisartan	144701-48-4
Trenbolone	10161-33-8	17beta-Trenbolone	10161-33-8
Trimethoprim	738-70-5	Trimethoprim	738-70-5
Valsartan	137862-53-4	Valsartan	137862-53-4
Venlafaxine	93413-69-5	Venlafaxine	93413-69-5
10,11-Dihydro-10-hydroxy Carbamazepine	29331-92-8	NA	NA
2-Ethyl-2-phenylmalonamide	7206-76-0	NA	NA
Benzoylecgonine	519-09-5	Benzoylecgonine	519-09-5
Hydroxybupropion	92264-81-8	NA	NA
N4-Acetylsulfamethoxazole	21312-10-7	N-Acetyl Sulfamethoxazole	21312-10-7
Ritalinic Acid	19395-41-6	NA	NA
(4-Chloro-2-methylphenoxy)acetic Acid (MCPA)	94-74-6	MCPA	94-74-6
2,4-Dichlorophenoxyacetic Acid (2,4-D)	94-75-7	2,4-Dichlorophenoxyacetic Acid	94-75-7
Abscisic Acid	21293-29-8	Abscisic Acid	14375-45-2
Acetamiprid	135410-20-7	Acetamiprid	135410-20-7
Ametryn	834-12-8	Ametryn	834-12-8

**Table S18.** ToxCast 3.2 entries for OMPs quantified in POCIS and grab samples (continued)			
Compound Name	CAS	ToxCast Preferred Name	CAS
Atrazine	1912-24-9	Atrazine	1912-24-9
Bifenazate	149877-41-8	Bifenazate	149877-41-8
Carbaryl	63-25-2	Carbaryl	63-25-2
Carbendazim	10605-21-7	Carbendazim	10605-21-7
Clothianidin	210880-92-5	Clothianidin	210880-92-5
Diuron	330-54-1	Diuron	330-54-1
Imazapyr	81334-34-1	Imazapyr	81334-34-1
Imidacloprid	138261-41-3	Imidacloprid	138261-41-3
Mecoprop	93-65-2	Mecoprop	93-65-2
Malathion	121-75-5	Malathion	121-75-5
Metalaxyl	57837-19-1	Metalaxyl	57837-19-1
Metolachlor	51218-45-2	Metolachlor	51218-45-2
Monuron	150-68-5	Monuron	150-68-5
Prometon	1610-18-0	Prometon	1610-18-0
Prometryn	7287-19-6	Prometryn	7287-19-6
Propazine	139-40-2	Propazine	139-40-2
Simazine	122-34-9	Simazine	122-34-9
Thiamethoxam	153719-23-4	Thiamethoxam	153719-23-4
Atrazine-2-hydroxy	2163-68-0	NA	NA
Atrazine-desethyl	6190-65-4	Deethylatrazine	6190-65-4
Atrazine-desisopropyl	1007-28-9	Deisopropylatrazine	1007-28-9
Metolachlor Ethanesulfonic Acid (Metolachlor ESA)	171118-09-5	Metolachlor ESA	171118-09-5
Metolachlor Oxanilic Acid (Metolachlor OA)	152019-73-3	Metolachlor OA	152019-73-3
Benzotriazole	95-14-7	1,2,3-Benzotriazole	95-14-7
2-Hydroxybenzothiazole	934-34-9	Benzothiazolone	934-34-9
Methyl-1H-benzotriazole	136-85-6	5-Methyl-1H-benzotriazole	136-85-6
Benzophenone	119-61-9	Benzophenone	119-61-9
Benzophenone-3 (Oxybenzone)	131-57-7	2-Hydroxy-4-methoxybenzophenone	131-57-7
Benzothiazole	95-16-9	Benzothiazole	95-16-9
N,N-Diethyl-3-methylbenzamide (DEET)	134-62-3	DEET	134-62-3
Ethyl Butylacetylaminopropionate	52304-36-6	Ethyl 3-(N-butylacetamido)propionate	52304-36-6
Sucralose	56038-13-2	Sucralose	56038-13-2
Triclosan	3380-34-5	Triclosan	3380-34-5
Galaxolidone	507442-49-1	NA	NA
Caprolactam	105-60-2	Caprolactam	105-60-2
1,3-Diphenylguanidine	102-06-7	1,3-Diphenylguanidine	102-06-7
3-Cyclohexyl-1,1-dimethylurea	31468-12-9	NA	NA
Hexa(methoxymethyl)melamine (HMMM)	3089-11-0	Hexa(methoxymethyl)melamine	3089-11-0
N-Cyclohexyl-N'-phenylurea	886-59-9	NA	NA
N-Ethyl-p-toluenesulfonamide	80-39-7	N-Ethyl-4-methylbenzenesulfonamide	80-39-7
Triphenylphosphine Oxide	791-28-6	Triphenylphosphine Oxide	791-28-6
Dextrorphan	125-73-5	NA	NA

Table S18. ToxCast 3.2 entries for OMPs quantified in POCIS and grab samples (continued)

Compound Name	CAS	ToxCast Preferred Name	CAS
4-Methylbenzophenone	134-84-9	4-Methylbenzophenone	134-84-9
Diheptyl Phthalate	3648-21-3	Diheptyl Phthalate	3648-21-3
Di(propylene glycol) Dibenzoate	27138-31-4	Di(propylene glycol) Dibenzoate	27138-31-4
Hexadecyltrimethylammonium (Cetrimonium)	112-02-7	Hexadecyl Trimethyl Ammonium Chloride	112-02-7
Lauramidopropyl Betaine	86438-79-1	NA	NA
N-Ethyl-p-menthane-3-carboxamide	39711-79-0	N-Ethyl-4-menthane-3-carboxamide	39711-79-0
Tributyl Citrate	77-94-1	Tributyl Citrate	77-94-1
Tris(2-butoxyethyl) Phosphate (TBEP)	78-51-3	Tris(2-butoxyethyl) Phosphate	78-51-3
Daidzein	486-66-8	Daidzein	486-66-8
Nobiletin	478-01-3	NA	NA

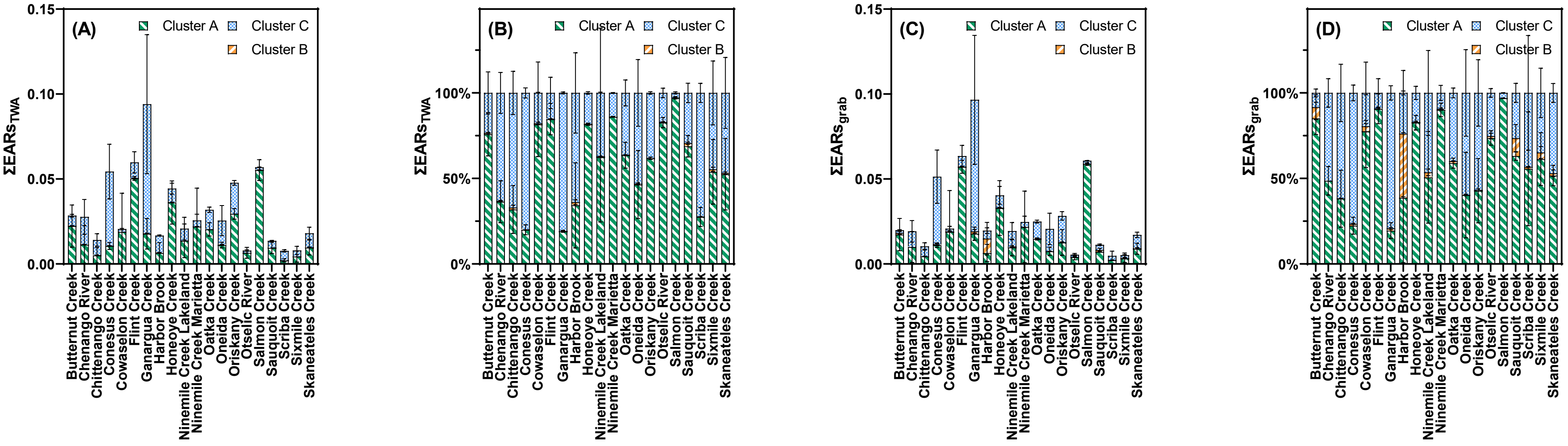

Figure S14. Comparison of the site-specific cumulative exposure-activity ratios (ΣEARs) for OMPs in POCIS and grab samples under mean exposure conditions: **(A)** ΣEARs for the 20 stream sites calculated based on c_{TWA} (ΣEARs$_{\mathrm{TWA}}$). **(B)** Percent contributions of clusters A, B, and C OMPs to ΣEARs$_{\mathrm{TWA}}$. **(C)** ΣEARs for the 20 stream sites calculated based on c_{grab} (ΣEARs$_{\mathrm{grab}}$). **(D)** Percent contributions of clusters A, B, and C OMPs to ΣEARs$_{\mathrm{grab}}$. Error bars represent the standard deviations of ΣEARs$_{\mathrm{TWA}}$ and ΣEARs$_{\mathrm{grab}}$. For each OMP, the EAR was calculated based on c_{TWA} and c_{grab}, respectively, using exposure-effects relation data in the *ToxCast* database.[48] Two sets of assays, *Apredica* and *Bioseek*, as well as *Attagene* assays reporting signal loss and *Novascreen* assays reporting signal gain were excluded from the calculation.[49] Chemical-assay combinations with the following six data quality flags were removed: "borderline active", "only highest concentration above baseline, active", "only one concentration above baseline, active", "noisy data", "gain AC50 < lowest concentration & loss AC50 < mean concentration", and "biochemical assay with < 50% efficacy".[49-51] Endpoints categorized as "background measurement", "undefined", "cell cycle", and "cell morphology" were also excluded from the calculation.[49]

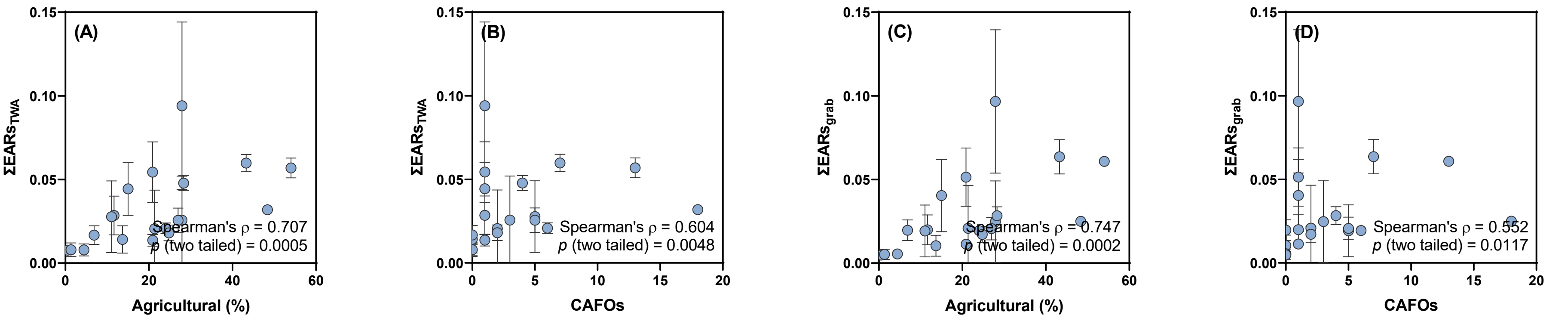

Figure S15. Watershed attributes as predictors for the site-specific cumulative exposure-activity ratios (ΣEARs) for OMPs under mean exposure conditions at the 20 stream sites: **(A)** Spearman's correlation between $\Sigma EARs_{TWA}$ and the percent watershed agricultural land usage (Agricultural). **(B)** Spearman's correlation between $\Sigma EARs_{TWA}$ and the number of concentrated animal feeding operations (CAFOs) within the watershed. **(C)** Spearman's correlation between $\Sigma EARs_{grab}$ and the percent watershed agricultural land usage (Agricultural). **(D)** Spearman's correlation between $\Sigma EARs_{grab}$ and the number of concentrated animal feeding operations (CAFOs) within the watershed. Error bars represent the standard deviations of $\Sigma EARs_{TWA}$ and $\Sigma EARs_{grab}$.

S10. Multi-substance potentially affected fraction (msPAF) analysis

Table S19. Species sensitivity distribution data for OMPs quantified in POCIS and grab samples

Compound Name	CAS	TMoA	# Species	μ	σ	Quality Score
Abacavir	136470-78-5	Antiviral, Reverse transcriptase inhibitor	3	3.69	1.49	1336
Acetaminophen	103-90-2	Analgesic, Antipyretic	9	5.22	0.81	1211
Aliskiren	173334-57-1	Antihypertensive, Renin inhibitor	3	3.59	0.38	1336
Amantadine	768-94-5	Antiviral, M2 protein inhibitor, Antiparkinsonian, Dopamine secretagogue	4	4.49	0.20	1311
Amitriptyline	50-48-6	Antidepressant	3	2.56	0.38	1336
Atenolol	29122-68-7	Antiarrhythmic, Antihypertensive, Vasodilator, beta1-Adrenergic receptor antagonist	24	5.20	1.04	1111
Azelaic Acid	123-99-9	Anti-acne, Thioredoxin reductase inhibitor	3	4.05	0.68	1325
Azithromycin	83905-01-5	Antibacterial, Protein biosynthesis inhibitor	15	2.40	1.29	1111
Bamethan	3703-79-5	Vasodilator (peripheral)	NA	NA	NA	NA
Bisoprolol	66722-44-9	Antihypertensive, beta1-Adrenergic receptor antagonist	3	3.82	0.67	1336
Bupropion	34911-55-2	Antidepressant, Noradrenalin and dopamine reuptake inhibitor	3	3.07	0.33	1336
Caffeine	58-08-2	Stimulant (central), Adenosine receptor antagonist, Phosphodiesterase inhibitor	15	4.59	0.85	1111
Carbamazepine	298-46-4	Analgesic, Antiepileptic	19	4.59	0.42	1111
Cetirizine	83881-51-0	Antiallergic, H1 receptor antagonist	3	3.44	0.58	1336
Citalopram	59729-33-8	Antidepressant, Selective serotonin reuptake inhibitor (SSRI)	3	2.91	0.36	1336
Clarithromycin	81103-11-9	Antibacterial, Protein biosynthesis inhibitor	24	3.45	1.29	1111
Clindamycin	18323-44-9	Antibacterial, Protein biosynthesis inhibitor	3	4.59	0.77	1336
Codeine	76-57-3	Analgesic (narcotic), Antitussive, Opioid receptor agonist	3	3.56	0.54	1336
Desipramine	50-47-5	Antidepressant, Serotonin-noradrenaline reuptake inhibitor (SNRI)	3	2.94	0.51	1336
Desvenlafaxine (O-Desmethyl Venlafaxine)	93413-62-8	Antidepressant, Serotonin-noradrenaline reuptake inhibitor (SNRI)	3	4.13	0.34	1311
Dexpanthenol	81-13-0	Supplement (pantothenic acid), Muscarinic acetylcholine receptor agonist	3	6.17	1.15	1322
Dextromethorphan	125-71-3	Antitussive	3	3.10	0.54	1336
Diazepam	439-14-5	Antianxiety, Minor tranquilizer, Sedative-hypnotic	3	3.74	0.32	1311
Diltiazem	42399-41-7	Antiarrhythmic, Antihypertensive, Vasodilator (coronary), Calcium channel blocker	3	3.54	0.37	1336
Diphenhydramine	58-73-1	Antiallergic, H1 receptor antagonist	3	3.16	0.50	1336
Dopamine	51-61-6	Cardiotonic, Dopamine receptor agonist	3	3.80	0.59	1336
Doxylamine	469-21-6	Antiallergic, Sedative-hypnotic, H1 receptor antagonist	3	3.45	0.61	1336
Ephedrine	299-42-3	Bronchodilator, Adrenergic receptor agonist	3	4.06	0.79	1336
Fexofenadine	83799-24-0	Antiallergic, H1 receptor antagonist	4	5.69	0.28	1311
Flecainide	54143-55-4	Antiarrhythmic, Sodium channel blocker	3	3.20	0.40	1336
Fluconazole	86386-73-4	Antifungal, Ergosterol biosynthesis inhibitor	3	4.79	0.37	1311
Fluoxetine	54910-89-3	Antidepressant, Selective serotonin reuptake inhibitor (SSRI)	11	3.78	1.39	1111
Gabapentin	60142-96-3	Anticonvulsant, Antiepileptic	3	4.57	1.11	1336
Gemfibrozil	25812-30-0	Antihyperlipidemic, Triglyceride synthesis inhibitor, Peroxisome proliferator-activated receptor (PPAR) alpha agonist	14	4.54	0.67	1111
Griseofulvin	126-07-8	Antifungal	3	4.81	0.70	1311
Guaifenesin	93-14-1	Antitussive, Expectorant	3	5.65	0.76	1336
Irbesartan	138402-11-6	Antihypertensive, Angiotensin II receptor antagonist	3	5.34	0.33	1311
Labetalol	36894-69-6	Antihypertensive, alpha1/beta-Adrenergic receptor antagonist	3	3.66	0.40	1336

**Table S19.** Species sensitivity distribution data for OMPs quantified in POCIS and grab samples (continued)						
Compound Name	CAS	TMoA	# Species	μ	σ	Quality Score
Lamotrigine	84057-84-1	Anti-bipolar disorder, Anticonvulsant, Antiepileptic	3	4.76	0.70	1311
Levamisole	14769-73-4	Anthelmintic	3	4.06	0.61	1336
Levetiracetam	102767-28-2	Antiepileptic	3	5.07	1.60	1336
Levorphanol	77-07-6	Analgesic (narcotic)	NA	NA	NA	NA
Lidocaine	137-58-6	Anesthetic (topical), Antiarrhythmic, Sodium channel blocker	3	3.55	0.50	1336
Losartan	114798-26-4	Antihypertensive, Angiotensin II receptor antagonist	3	6.20	0.45	1325
Maprotiline	10262-69-8	Antidepressant, Selective noradrenaline reuptake inhibitor (NRI)	3	2.67	0.45	1336
Metaxalone	1665-48-1	Skeletal muscle relaxant	3	4.77	0.30	1311
Metformin	657-24-9	Antidiabetic, Hypoglycemic, AMP kinase activator	4	5.37	0.58	1311
Methocarbamol	532-03-6	Skeletal muscle relaxant	3	5.00	1.09	1336
Metoprolol	51384-51-1	Antihypertensive, Vasodilator, beta1-Adrenergic receptor antagonist	6	4.70	0.61	1211
Molindone	7416-34-4	Antipsychotic, Dopamine D2 receptor antagonist	NA	NA	NA	NA
Mycophenolic Acid	24280-93-1	Immunosuppressant, Inosine monophosphate dehydrogenase inhibitor	3	3.63	0.38	1336
Nadolol	42200-33-9	Antiarrhythmic, Antihypertensive, Vasodilator, beta-Adrenergic receptor antagonist	15	5.06	1.04	1111
Naproxen	22204-53-1	Analgesic, Anti-inflammatory, Antipyretic, COX inhibitor	10	4.33	1.12	1211
Oxcarbazepine	28721-07-5	Anticonvulsant, Antiepileptic	3	3.84	0.87	1336
Phenytoin	57-41-0	Antiepileptic	3	3.71	0.90	1336
Prilocaine	721-50-6	Anesthetic (local)	3	3.84	0.61	1336
Propranolol	525-66-6	Antiarrhythmic, Antihypertensive, beta-Adrenergic receptor antagonist	30	3.64	1.04	1111
Protriptyline	438-60-8	Antidepressant	NA	NA	NA	NA
Ranitidine	66357-35-5	Anti-ulcerative, H2 receptor antagonist	4	5.00	0.88	1311
Rimantadine	13392-28-4	Antiviral, M2 protein inhibitor	NA	NA	NA	NA
Sertraline	79617-96-2	Antidepressant, Selective serotonin reuptake inhibitor (SSRI)	11	2.67	0.87	1122
Sitagliptin	486460-32-6	Antidiabetic, Dipeptidyl peptidase-4 (DPP-4) inhibitor	3	3.82	1.81	1336
Sotalol	3930-20-9	Antiarrhythmic, beta-Adrenergic receptor antagonist, Potassium channel blocker	12	5.52	1.04	1111
Sulfamethoxazole	723-46-6	Antibacterial, Folic acid biosynthesis inhibitor	15	3.98	1.36	1111
Sulfapyridine	144-83-2	Antibacterial, Folic acid biosynthesis inhibitor	3	4.32	1.03	1325
Telmisartan	144701-48-4	Antihypertensive, Angiotensin II receptor antagonist	3	2.59	1.76	1336
Trenbolone	10161-33-8	Anabolic	6	-0.06	1.61	1224
Trimethoprim	738-70-5	Antibacterial, Folic acid biosynthesis inhibitor	11	4.70	0.62	1111
Valsartan	137862-53-4	Antihypertensive, Angiotensin II receptor antagonist	3	5.15	0.55	1311
Venlafaxine	93413-69-5	Antidepressant, Serotonin-noradrenaline reuptake inhibitor (SNRI)	3	3.32	0.79	1325
10,11-Dihydro-10-hydroxy Carbamazepine	29331-92-8	Analgesic, Antiepileptic	3	4.06	1.05	1336
2-Ethyl-2-phenylmalonamide	7206-76-0	Unclassified	3	4.66	1.37	1336
Benzoylecgonine	519-09-5	Anesthetic (topical), Narcotic	3	4.43	0.86	1336
Hydroxybupropion	92264-81-8	Antidepressant, Noradrenalin and dopamine reuptake inhibitor	3	3.59	0.54	1336
N4-Acetylsulfamethoxazole	21312-10-7	Antibacterial, Folic acid biosynthesis inhibitor	3	4.26	1.09	1336
Ritalinic Acid	19395-41-6	Psychostimulant, Stimulant (central)	3	3.64	1.03	1336
(4-Chloro-2-methylphenoxy)acetic Acid (MCPA)	94-74-6	Phenoxyacetic acid, Auxin mimic	22	4.46	1.11	1111
2,4-Dichlorophenoxyacetic Acid (2,4-D)	94-75-7	Phenoxyacetic acid, Auxin mimic	51	4.71	0.93	1111
Abscisic Acid	14375-45-2	Plant growth regulator	NA	NA	NA	NA

Table S19. Species sensitivity distribution data for OMPs quantified in POCIS and grab samples (continued)						
Compound Name	CAS	TMoA	# Species	μ	σ	Quality Score
Acetamiprid	135410-20-7	Pyridylmethylamine neonicotinoid, Nicotinic acetylcholine receptor (nAChR) competitive modulator	10	3.27	1.79	1211
Ametryn	834-12-8	Methylthiotriazine, Photosynthesis at PSll inhibitor	35	3.09	1.19	1111
Atrazine	1912-24-9	Chlorotriazine, Photosynthesis at PSll inhibitor	134	3.28	1.13	1111
Bifenazate	149877-41-8	Carbazate, Mitochondrial complex III electron transport inhibitor	13	2.88	0.57	1111
Carbaryl	63-25-2	Carbamate, Acetylcholinesterase (ACHE) inhibitor	161	2.86	1.21	1111
Carbendazim	10605-21-7	Benzimidazole, β-tubulin assembly in mitosis inhibitor	29	3.53	1.22	1111
Clothianidin	210880-92-5	Nitroguanidine neonicotinoid, Nicotinic acetylcholine receptor (nAChR) competitive modulator	11	3.69	1.81	1111
Diuron	330-54-1	Phenylurea, Photosynthesis at PSll inhibitor	86	2.82	1.26	1111
Imazapyr	81334-34-1	Imidazolinone, Acetolactate synthase inhibitor	9	4.76	0.55	1211
Imidacloprid	138261-41-3	Pyridylmethylamine neonicotinoid, Nicotinic acetylcholine receptor (nAChR) competitive modulator	37	2.95	1.78	1111
Mecoprop	93-65-2	Phenoxy-carboxylate, Auxin Mimic	3	4.67	0.58	1311
Malathion	121-75-5	Organothiophosphate, Acetylcholinesterase (ACHE) inhibitor	208	2.45	1.32	1111
Metalaxyl	57837-19-1	Acylalanine, RNA polymerase I inhibitor	21	4.93	0.80	1111
Metolachlor	51218-45-2	Chloroacetanilide, Very long-chain fatty acid synthesis inhibitor	33	3.83	0.82	1111
Monuron	150-68-5	Phenylurea, Photosynthesis at PSll inhibitor	13	4.53	0.52	1111
Prometon	1610-18-0	Methoxytriazine, Photosynthesis at PSll inhibitor	13	4.46	0.23	1111
Prometryn	7287-19-6	Methylthiotriazine, Photosynthesis at PSll inhibitor	23	3.03	1.08	1111
Propazine	139-40-2	Chlorotriazine, Photosynthesis at PSll inhibitor	10	4.00	1.05	1211
Simazine	122-34-9	Chlorotriazine, Photosynthesis at PSll inhibitor	56	3.88	1.16	1111
Thiamethoxam	153719-23-4	Nitroguanidine neonicotinoid, Nicotinic acetylcholine receptor (nAChR) competitive modulator	15	3.61	1.51	1111
Atrazine-2-hydroxy	2163-68-0	Chlorotriazine, Photosynthesis at PSll inhibitor	1	5.00	0.70	2411
Atrazine-desethyl	6190-65-4	Chlorotriazine, Photosynthesis at PSll inhibitor	4	1.24	1.44	1325
Atrazine-desisopropyl	1007-28-9	Chlorotriazine, Photosynthesis at PSll inhibitor	1	3.95	0.70	2411
Metolachlor Ethanesulfonic Acid (Metolachlor ESA)	171118-09-5	Chloroacetanilide, Very long-chain fatty acid synthesis inhibitor	NA	NA	NA	NA
Metolachlor Oxanilic Acid (Metolachlor OA)	152019-73-3	Chloroacetanilide, Very long-chain fatty acid synthesis inhibitor	NA	NA	NA	NA
Benzotriazole	95-14-7	Benzotriazole	19	4.74	0.50	1111
2-Hydroxybenzothiazole	934-34-9	Benzothiazole	NA	NA	NA	NA
Methyl-1H-benzotriazole	136-85-6	Benzotriazole	3	4.68	0.27	1325
Benzophenone	119-61-9	Nonpolar narcosis	24	3.55	0.70	1111
Benzophenone-3 (Oxybenzone)	131-57-7	Nonpolar narcosis	3	3.42	0.53	1311
Benzothiazole	95-16-9	Benzothiazole	10	4.58	0.58	1211
N,N-Diethyl-3-methylbenzamide (DEET)	134-62-3	Insect repellent	5	5.03	0.70	1311
Ethyl Butylacetylaminopropionate	52304-36-6	Insect repellent	NA	NA	NA	NA
Sucralose	56038-13-2	Artificial sweetener	NA	NA	NA	NA
Triclosan	3380-34-5	Polar narcosis	19	2.05	1.03	1111
Galaxolidone	507442-49-1	Fragrance	NA	NA	NA	NA
Caprolactam	105-60-2	Unclassified	3	6.29	0.51	1311
1,3-Diphenylguanidine	102-06-7	Unclassified	3	3.98	0.49	1311

Table S19. Species sensitivity distribution data for OMPs quantified in POCIS and grab samples (continued)						
Compound Name	CAS	TMoA	# Species	μ	σ	Quality Score
3-Cyclohexyl-1,1-dimethylurea	31468-12-9	Unclassified	NA	NA	NA	NA
Hexa(methoxymethyl)melamine (HMMM)	3089-11-0	Unclassified	NA	NA	NA	NA
N-Cyclohexyl-N'-phenylurea	886-59-9	Unclassified	NA	NA	NA	NA
N-Ethyl-p-toluenesulfonamide	80-39-7	Unclassified	NA	NA	NA	NA
Triphenylphosphine Oxide	791-28-6	Flame retardant	4	4.63	0.23	1311
Dextrorphan	125-73-5	Antitussive, NMDA receptor antagonist	3	3.21	0.27	1336
4-Methylbenzophenone	134-84-9	Nonpolar narcosis	NA	NA	NA	NA
Diheptyl Phthalate	3648-21-3	Diester	NA	NA	NA	NA
Di(propylene glycol) Dibenzoate	27138-31-4	Unclassified	3	3.65	0.68	1311
Hexadecyltrimethylammonium (Cetrimonium)	112-02-7	Unclassified	11	2.52	0.83	1111
Lauramidopropyl Betaine	86438-79-1	Unclassified	NA	NA	NA	NA
N-Ethyl-p-menthane-3-carboxamide	39711-79-0	Unclassified	NA	NA	NA	NA
Tributyl Citrate	77-94-1	Unclassified	3	4.47	0.50	1311
Tris(2-butoxyethyl) Phosphate (TBEP)	78-51-3	Flame retardant	7	4.50	0.33	1211
Daidzein	486-66-8	Unclassified	NA	NA	NA	NA
Nobiletin	478-01-3	Unclassified	NA	NA	NA	NA

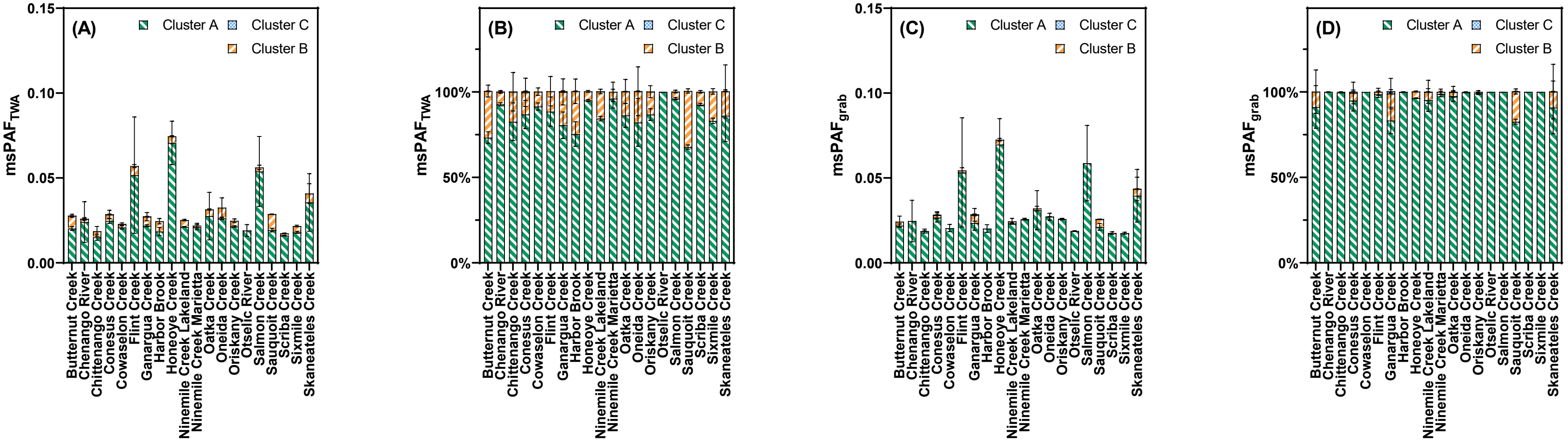

Figure S16. Comparison of the site-specific multi-substance potentially affected fraction (msPAF) for OMPs in POCIS and grab samples under mean exposure conditions: **(A)** msPAF for the 20 stream sites calculated based on c_{TWA} ($\mathrm{msPAF_{TWA}}$). **(B)** Percent contributions of clusters A, B, and C OMPs to $\mathrm{msPAF_{TWA}}$. **(C)** msPAF for the 20 stream sites calculated based on c_{grab} ($\mathrm{msPAF_{grab}}$). **(D)** Percent contributions of clusters A, B, and C OMPs to $\mathrm{msPAF_{grab}}$. Error bars represent the standard deviations of $\mathrm{msPAF_{TWA}}$ and $\mathrm{msPAF_{grab}}$. For each OMP, the PAF was calculated based on c_{TWA} and c_{grab}, respectively, as described in previous work[52] using the *Microsoft Excel*© function PAF = NORM.DIST($[\mathrm{OMP}]_i$, μ_i, σ_i, 1) where μ and σ are the SSD median and slope parameters summarized in Table S19. msPAFs were first calculated for OMPs with the same toxic mode of action (TMoA) with the concentration addition approach using $\mathrm{msPAF_{TMoA}}$ = NORM.DIST($\log10(\Sigma[\mathrm{OMP}]_i/10^{\mu_i})_{\mathrm{TMoA}}$, 0, σ_{TMoA}, 1) where σ_{TMoA} is the arithmetic mean σ for all OMPs within the same TMoA group. msPAFs for the mixture of OMPs with different TMoAs were then calculated with the response addition approach using $\mathrm{msPAF} = 1 - \prod_{j=1}^{n}(1 - \mathrm{msPAF}_{\mathrm{TMoA},j})$ as described in previous work.[53]

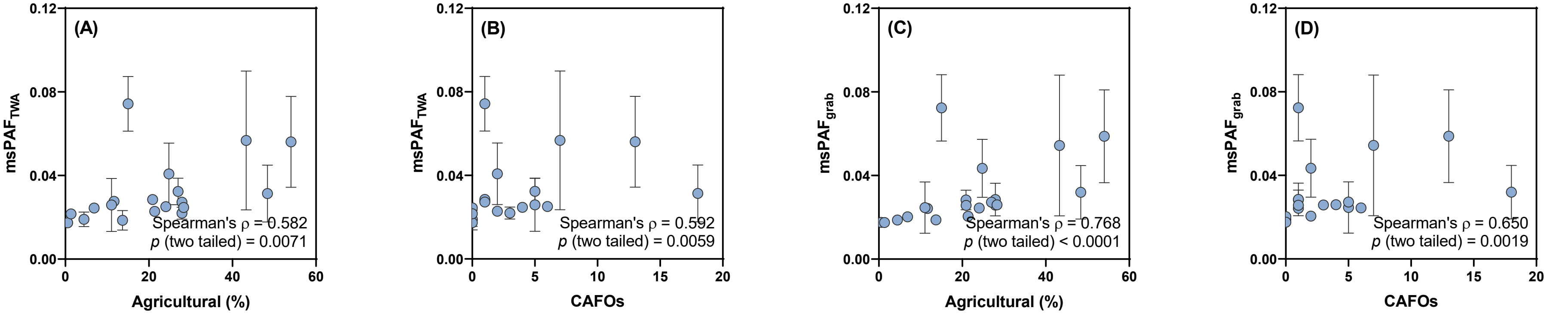

Figure S17. Watershed attributes as predictors for the multi-substance potentially affected fraction (msPAF) for OMPs under mean exposure conditions at the 20 stream sites: **(A)** Spearman's correlation between $msPAF_{TWA}$ and the percent watershed agricultural land usage (Agricultural). **(B)** Spearman's correlation between $msPAF_{TWA}$ and the number of concentrated animal feeding operations (CAFOs) within the watershed. **(C)** Spearman's correlation between $msPAF_{grab}$ and the percent watershed agricultural land usage (Agricultural). **(D)** Spearman's correlation between $msPAF_{grab}$ and the number of concentrated animal feeding operations (CAFOs) within the watershed. Error bars represent the standard deviations of $msPAF_{TWA}$ and $msPAF_{grab}$.

S11. Compound database for SPE-LC-HRMS suspect screening

Table S20. Compound database for suspect screening

Compound Name	CAS	Molecular Formula	Category
17alpha-Estradiol (Alfatradiol)	57-91-0	C18H24O2	Pharmaceutical (5alpha-Reductase inhibitor)
Mifepristone	84371-65-3	C29H35NO2	Pharmaceutical (Abortifacient, Contraceptive, Progesterone receptor antagonist)
Prasterone	53-43-0	C19H28O2	Pharmaceutical (Accelerator (cervical ripening), Androgen receptor agonist)
Pegaptanib	222716-86-1	C22H44N3O10P	Pharmaceutical (Aging-related macular degeneration therapeutic agent, Angiogenesis inhibitor, Anti-VEGF aptamer)
Clioquinol	130-26-7	C9H5ClINO	Pharmaceutical (Amebicide, Antifungal)
Clostebol	1093-58-9	C19H27ClO2	Pharmaceutical (Anabolic)
Ethylestrenol	965-90-2	C20H32O	Pharmaceutical (Anabolic)
Furazabol	1239-29-8	C20H30N2O2	Pharmaceutical (Anabolic)
Mestanolone	521-11-9	C20H32O2	Pharmaceutical (Anabolic)
Norbolethone	1235-15-0	C21H32O2	Pharmaceutical (Anabolic)
Stenbolone	5197-58-0	C20H30O2	Pharmaceutical (Anabolic)
Trenbolone	10161-33-8	C18H22O2	Pharmaceutical (Anabolic)
Zeranol	26538-44-3	C18H26O5	Pharmaceutical (Anabolic)
Androstanolone (Stanolone/5α-Dihydrotestosterone)	521-18-6	C19H30O2	Pharmaceutical (Anabolic, Androgen receptor agonist)
Bolasterone	1605-89-6	C21H32O2	Pharmaceutical (Anabolic, Androgen receptor agonist)
Boldenone	846-48-0	C19H26O2	Pharmaceutical (Anabolic, Androgen receptor agonist)
Drostanolone	58-19-5	C20H32O2	Pharmaceutical (Anabolic, Androgen receptor agonist)
Mesterolone	1424-00-6	C20H32O2	Pharmaceutical (Anabolic, Androgen receptor agonist)
Metandienone	72-63-9	C20H28O2	Pharmaceutical (Anabolic, Androgen receptor agonist)
Methandriol	521-10-8	C20H32O2	Pharmaceutical (Anabolic, Androgen receptor agonist)
Methenolone	153-00-4	C20H30O2	Pharmaceutical (Anabolic, Androgen receptor agonist)
Mibolerone	3704-09-4	C20H30O2	Pharmaceutical (Anabolic, Androgen receptor agonist)
Nandrolone	434-22-0	C18H26O2	Pharmaceutical (Anabolic, Androgen receptor agonist)
Norethandrolone	52-78-8	C20H30O2	Pharmaceutical (Anabolic, Androgen receptor agonist)
Oxandrolone	53-39-4	C19H30O3	Pharmaceutical (Anabolic, Androgen receptor agonist)
Oxymetholone	434-07-1	C21H32O3	Pharmaceutical (Anabolic, Androgen receptor agonist)
Stanozolol	10418-03-8	C21H32N2O	Pharmaceutical (Anabolic, Androgen receptor agonist)
Eugenol	97-53-0	C10H12O2	Pharmaceutical (Analgesic (dental), Disinfectant)
Levorphanol	77-07-6	C17H23NO	Pharmaceutical (Analgesic (narcotic))
Normethadone	467-85-6	C20H25NO	Pharmaceutical (Analgesic (narcotic), Antitussive)
Codeine	76-57-3	C18H21NO3	Pharmaceutical (Analgesic (narcotic), Antitussive, Opioid receptor agonist)
Dihydrocodeine	125-28-0	C18H23NO3	Pharmaceutical (Analgesic (narcotic), Antitussive, Opioid receptor agonist)
Ethylmorphine	76-58-4	C19H23NO3	Pharmaceutical (Analgesic (narcotic), Antitussive, Opioid receptor agonist)
Alfentanil	71195-58-9	C21H32N6O3	Pharmaceutical (Analgesic (narcotic), Opioid receptor agonist)
Anileridine	144-14-9	C22H28N2O2	Pharmaceutical (Analgesic (narcotic), Opioid receptor agonist)
Carfentanil	59708-52-0	C24H30N2O3	Pharmaceutical (Analgesic (narcotic), Opioid receptor agonist)
Fentanyl	437-38-7	C22H28N2O	Pharmaceutical (Analgesic (narcotic), Opioid receptor agonist)
Hydromorphone	466-99-9	C17H19NO3	Pharmaceutical (Analgesic (narcotic), Opioid receptor agonist)
Levacetylmethadol	1477-40-3	C23H31NO2	Pharmaceutical (Analgesic (narcotic), Opioid receptor agonist)
Meperidine	57-42-1	C15H21NO2	Pharmaceutical (Analgesic (narcotic), Opioid receptor agonist)
Morphine	57-27-2	C17H19NO3	Pharmaceutical (Analgesic (narcotic), Opioid receptor agonist) / Pharmaceutical TP (Heroin)
Oxycodone	76-42-6	C18H21NO4	Pharmaceutical (Analgesic (narcotic), Opioid receptor agonist)
Oxymorphone	76-41-5	C17H19NO4	Pharmaceutical (Analgesic (narcotic), Opioid receptor agonist)
Remifentanil	132875-61-7	C20H28N2O5	Pharmaceutical (Analgesic (narcotic), Opioid receptor agonist)
Sufentanil	56030-54-7	C22H30N2O2S	Pharmaceutical (Analgesic (narcotic), Opioid receptor agonist)
Methadone	76-99-3	C21H27NO	Pharmaceutical (Analgesic (narcotic), Opioid receptor agonist, NMDA receptor antagonist)
Capsaicin	404-86-4	C18H27NO3	Pharmaceutical (Analgesic (topical), Transient receptor potential (TRP) channel agonist)
Phenazopyridine	94-78-0	C11H11N5	Pharmaceutical (Analgesic (urinary tract))
Xylazine	7361-61-7	C12H16N2S	Pharmaceutical (Analgesic (veterinary), alpha2-Adrenergic receptor agonist)
Robenacoxib	220991-32-2	C16H13F4NO2	Pharmaceutical (Analgesic (veterinary), Anti-inflammatory, Antipyretic)
Detomidine	76631-46-4	C12H14N2	Pharmaceutical (Analgesic (veterinary), Sedative-hypnotic (veterinary), alpha2-Adrenergic receptor agonist)
Medetomidine	86347-14-0	C13H16N2	Pharmaceutical (Analgesic (veterinary), Sedative-hypnotic (veterinary), alpha2-Adrenergic receptor agonist)
Antipyrine (Phenazone)	60-80-0	C11H12N2O	Pharmaceutical (Analgesic)
Clonixin	17737-65-4	C13H11ClN2O2	Pharmaceutical (Analgesic)
Ketobemidone	469-79-4	C15H21NO2	Pharmaceutical (Analgesic)
Piritramide	302-41-0	C27H34N4O	Pharmaceutical (Analgesic)
Propiram	15686-91-6	C16H25N3O	Pharmaceutical (Analgesic)
Benzocaine	94-09-7	C9H11NO2	Pharmaceutical (Analgesic, Anesthetic (topical), Antipruritic)
Butacaine	149-16-6	C18H30N2O2	Pharmaceutical (Analgesic, Anesthetic (topical), Antipruritic)
Etorphine	14521-96-1	C25H33NO4	Pharmaceutical (Analgesic, Anesthetic, Opioid receptor agonist)
Carbamazepine	298-46-4	C15H12N2O	Pharmaceutical (Analgesic, Antiepileptic)
Pirfenidone	53179-13-8	C12H11NO	Pharmaceutical (Analgesic, Antifibrotic, Anti-inflammatory, Antipyretic)
Flunixin	38677-85-9	C14H11F3N2O2	Pharmaceutical (Analgesic, Anti-inflammatory)

Table S20. Compound database for suspect screening (continued)

Compound Name	CAS	Molecular Formula	Category
Aspirin (Acetylsalicylic Acid)	50-78-2	C9H8O4	Pharmaceutical (Analgesic, Anti-inflammatory, Antipyretic, Antirheumatic, Antiplatelet, COX inhibitor)
Phenylbutazone	50-33-9	C19H20N2O2	Pharmaceutical (Analgesic, Anti-inflammatory, Antipyretic, Antirheumatic, COX inhibitor)
Benzydamine	642-72-8	C19H23N3O	Pharmaceutical (Analgesic, Anti-inflammatory, Antipyretic, COX inhibitor)
Diclofenac	15307-86-5	C14H11Cl2NO2	Pharmaceutical (Analgesic, Anti-inflammatory, Antipyretic, COX inhibitor)
Ibuprofen	15687-27-1	C13H18O2	Pharmaceutical (Analgesic, Anti-inflammatory, Antipyretic, COX inhibitor)
Indomethacin	53-86-1	C19H16ClNO4	Pharmaceutical (Analgesic, Anti-inflammatory, Antipyretic, COX inhibitor)
Ketoprofen	22071-15-4	C16H14O3	Pharmaceutical (Analgesic, Anti-inflammatory, Antipyretic, COX inhibitor)
Mefenamic Acid	61-68-7	C15H15NO2	Pharmaceutical (Analgesic, Anti-inflammatory, Antipyretic, COX inhibitor)
Naproxen	22204-53-1	C14H14O3	Pharmaceutical (Analgesic, Anti-inflammatory, Antipyretic, COX inhibitor)
Firocoxib	189954-96-9	C17H20O5S	Pharmaceutical (Analgesic, Anti-inflammatory, Antipyretic, COX-2 inhibitor)
Piroxicam	36322-90-4	C15H13N3O4S	Pharmaceutical (Analgesic, Anti-inflammatory, Antirheumatic, COX inhibitor)
Bromfenac	91714-94-2	C15H12BrNO3	Pharmaceutical (Analgesic, Anti-inflammatory, COX inhibitor)
Diflunisal	22494-42-4	C13H8F2O3	Pharmaceutical (Analgesic, Anti-inflammatory, COX inhibitor)
Fenoprofen	29679-58-1	C15H14O3	Pharmaceutical (Analgesic, Anti-inflammatory, COX inhibitor)
Flurbiprofen	5104-49-4	C15H13FO2	Pharmaceutical (Analgesic, Anti-inflammatory, COX inhibitor)
Ketorolac	74103-06-3	C15H13NO3	Pharmaceutical (Analgesic, Anti-inflammatory, COX inhibitor)
Nabumetone	42924-53-8	C15H16O2	Pharmaceutical (Analgesic, Anti-inflammatory, COX inhibitor)
Nepafenac	78281-72-8	C15H14N2O2	Pharmaceutical (Analgesic, Anti-inflammatory, COX inhibitor)
Oxaprozin	21256-18-8	C18H15NO3	Pharmaceutical (Analgesic, Anti-inflammatory, COX inhibitor)
Sulindac	38194-50-2	C20H17FO3S	Pharmaceutical (Analgesic, Anti-inflammatory, COX inhibitor)
Celecoxib	169590-42-5	C17H14F3N3O2S	Pharmaceutical (Analgesic, Anti-inflammatory, COX-2 inhibitor)
Deracoxib	169590-41-4	C17H14F3N3O3S	Pharmaceutical (Analgesic, Anti-inflammatory, COX-2 inhibitor)
Etodolac	41340-25-4	C17H21NO3	Pharmaceutical (Analgesic, Anti-inflammatory, COX-2 inhibitor)
Lornoxicam	70374-39-9	C13H10ClN3O4S2	Pharmaceutical (Analgesic, Anti-inflammatory, COX-2 inhibitor)
Meloxicam	71125-38-7	C14H13N3O4S2	Pharmaceutical (Analgesic, Anti-inflammatory, COX-2 inhibitor)
Acetaminophen (Paracetamol)	103-90-2	C8H9NO2	Pharmaceutical (Analgesic, Antipyretic)
Butorphanol	42408-82-2	C21H29NO2	Pharmaceutical (Analgesic, Antitussive, Opioid receptor agonist/antagonist)
Heroin (Diamorphine)	561-27-3	C21H23NO5	Pharmaceutical (Analgesic, Opioid receptor agonist)
Propoxyphene	469-62-5	C22H29NO2	Pharmaceutical (Analgesic, Opioid receptor agonist)
Tilidine	17243-69-5	C17H23NO2	Pharmaceutical (Analgesic, Opioid receptor agonist)
Tramadol	27203-92-5	C16H25NO2	Pharmaceutical (Analgesic, Opioid receptor agonist)
Trimeperidine	64-39-1	C17H25NO2	Pharmaceutical (Analgesic, Opioid receptor agonist)
Tapentadol	175591-23-8	C14H23NO	Pharmaceutical (Analgesic, Opioid receptor agonist, Noradrenaline reuptake inhibitor)
Buprenorphine	52485-79-7	C29H41NO4	Pharmaceutical (Analgesic, Opioid receptor agonist/antagonist)
Pentazocine	359-83-1	C19H27NO	Pharmaceutical (Analgesic, Opioid receptor agonist/antagonist)
Epitestosterone	481-30-1	C19H28O2	Pharmaceutical (Androgen receptor agonist)
Isoflurane	26675-46-7	C3H2ClF5O	Pharmaceutical (Anesthetic (inhalation))
Thiamylal	77-27-0	C12H18N2O2S	Pharmaceutical (Anesthetic (intravenous))
Thiopental	76-75-5	C11H18N2O2S	Pharmaceutical (Anesthetic (intravenous), Anticonvulsant)
Midazolam	59467-70-8	C18H13ClFN3	Pharmaceutical (Anesthetic (intravenous), Anticonvulsant, Sedative-hypnotic)
Droperidol	548-73-2	C22H22FN3O2	Pharmaceutical (Anesthetic (intravenous), Antipsychotic)
Propofol	2078-54-8	C12H18O	Pharmaceutical (Anesthetic (intravenous), GABA-A receptor agonist)
Ketamine	6740-88-1	C13H16ClNO	Pharmaceutical (Anesthetic (intravenous), NMDA receptor antagonist)
Flunitrazepam	1622-62-4	C16H12FN3O3	Pharmaceutical (Anesthetic (intravenous), Sedative-hypnotic)
Articaine	23964-58-1	C13H20N2O3S	Pharmaceutical (Anesthetic (local))
Bupivacaine	38396-39-3	C18H28N2O	Pharmaceutical (Anesthetic (local))
Chloroprocaine	133-16-4	C13H19ClN2O2	Pharmaceutical (Anesthetic (local))
Dibucaine	85-79-0	C20H29N3O2	Pharmaceutical (Anesthetic (local))
Prilocaine	721-50-6	C13H20N2O	Pharmaceutical (Anesthetic (local))
Proparacaine	499-67-2	C16H26N2O3	Pharmaceutical (Anesthetic (local))
Ropivacaine	84057-95-4	C17H26N2O	Pharmaceutical (Anesthetic (local))
Benoxinate	99-43-4	C17H28N2O3	Pharmaceutical (Anesthetic (topical))
Mepivacaine	96-88-8	C15H22N2O	Pharmaceutical (Anesthetic (topical))
Tetracaine	94-24-6	C15H24N2O2	Pharmaceutical (Anesthetic (topical))
Lidocaine	137-58-6	C14H22N2O	Pharmaceutical (Anesthetic (topical), Antiarrhythmic, Sodium channel blocker)
Cocaine	50-36-2	C17H21NO4	Pharmaceutical (Anesthetic (topical), Narcotic)
Embutramide	15687-14-6	C17H27NO3	Pharmaceutical (Anesthetic (veterinary))
Phencyclidine	77-10-1	C17H25N	Pharmaceutical (Anesthetic)
Methohexital	151-83-7	C14H18N2O3	Pharmaceutical (Anesthetic, Sedative-hypnotic)
Butamisole	54400-59-8	C15H19N3OS	Pharmaceutical (Anthelmintic (veterinary))
Febantel	58306-30-2	C20H22N4O6S	Pharmaceutical (Anthelmintic (veterinary))
Morantel	20574-50-9	C12H16N2S	Pharmaceutical (Anthelmintic (veterinary))
Praziquantel	55268-74-1	C19H24N2O2	Pharmaceutical (Anthelmintic (veterinary))
Albendazole	54965-21-8	C12H15N3O2S	Pharmaceutical (Anthelmintic)
Cambendazole	26097-80-3	C14H14N4O2S	Pharmaceutical (Anthelmintic)
Ciclobendazole	31431-43-3	C13H13N3O3	Pharmaceutical (Anthelmintic)
Diethylcarbamazine	90-89-1	C10H21N3O	Pharmaceutical (Anthelmintic)

Table S20. Compound database for suspect screening (continued)

Compound Name	CAS	Molecular Formula	Category
Epsiprantel	98123-83-2	C20H26N2O2	Pharmaceutical (Anthelmintic)
Fenbendazole	43210-67-9	C15H13N3O2S	Pharmaceutical (Anthelmintic)
Levamisole	14769-73-4	C11H12N2S	Pharmaceutical (Anthelmintic)
Mebendazole	31431-39-7	C16H13N3O3	Pharmaceutical (Anthelmintic)
Nitroxinil	1689-89-0	C7H3IN2O3	Pharmaceutical (Anthelmintic)
Oxibendazole	20559-55-1	C12H15N3O3	Pharmaceutical (Anthelmintic)
Oxyclozanide	2277-92-1	C13H6Cl5NO3	Pharmaceutical (Anthelmintic)
Parbendazole	14255-87-9	C13H17N3O2	Pharmaceutical (Anthelmintic)
Pyrantel	15686-83-6	C11H14N2S	Pharmaceutical (Anthelmintic)
Rafoxanide	22662-39-1	C19H11Cl2I2NO3	Pharmaceutical (Anthelmintic)
Tioxidazole	61570-90-9	C12H14N2O3S	Pharmaceutical (Anthelmintic)
Triclabendazole	68786-66-3	C14H9Cl3N2OS	Pharmaceutical (Anthelmintic)
Oxfendazole	53716-50-0	C15H13N3O3S	Pharmaceutical (Anthelmintic) / Pharmaceutical TP (Fenbendazole)
Tazarotene	118292-40-3	C21H21NO2S	Pharmaceutical (Anti-acne, Antipsoriatic, Keratolytic, Retinoic acid receptor (RAR) agonist)
Benzoyl Peroxide	94-36-0	C14H10O4	Pharmaceutical (Anti-acne, Keratolytic)
Azelaic Acid	123-99-9	C9H16O4	Pharmaceutical (Anti-acne, Thioredoxin reductase inhibitor)
Disulfiram	97-77-8	C10H20N2S4	Pharmaceutical (Antialcohol dependence, Aldehyde dehydrogenase inhibitor)
Naltrexone	16590-41-3	C20H23NO4	Pharmaceutical (Antialcohol dependence, Narcotic antagonist, Opioid receptor antagonist)
Acamprosate	77337-76-9	C5H11NO4S	Pharmaceutical (Antialcohol dependence, NMDA receptor antagonist)
Nedocromil	69049-73-6	C19H17NO7	Pharmaceutical (Antiallergic)
Lodoxamide	53882-12-5	C11H6ClN3O6	Pharmaceutical (Antiallergic, Antiasthmatic)
Cromolyn	16110-51-3	C23H16O11	Pharmaceutical (Antiallergic, Antiasthmatic, Chemical mediator release inhibitor)
Azelastine	58581-89-8	C22H24ClN3O	Pharmaceutical (Antiallergic, Antiasthmatic, H1 receptor antagonist)
Ketotifen	34580-13-7	C19H19NOS	Pharmaceutical (Antiallergic, Antiasthmatic, H1 receptor antagonist)
Methylprednisolone	83-43-2	C22H30O5	Pharmaceutical (Antiallergic, Anti-inflammatory, Glucocorticoid receptor agonist)
Alcaftadine	147084-10-4	C19H21N3O	Pharmaceutical (Antiallergic, Anti-inflammatory, H1 receptor antagonist)
Bepotastine	125602-71-3	C21H25ClN2O3	Pharmaceutical (Antiallergic, Antipruritic, H1 receptor antagonist)
Desloratadine	100643-71-8	C19H19ClN2	Pharmaceutical (Antiallergic, Antipruritic, H1 receptor antagonist)
Acrivastine	87848-99-5	C22H24N2O2	Pharmaceutical (Antiallergic, H1 receptor antagonist)
Alimemazine (Trimeprazine)	84-96-8	C18H22N2S	Pharmaceutical (Antiallergic, H1 receptor antagonist)
Brompheniramine	86-22-6	C16H19BrN2	Pharmaceutical (Antiallergic, H1 receptor antagonist)
Carbinoxamine	486-16-8	C16H19ClN2O	Pharmaceutical (Antiallergic, H1 receptor antagonist)
Cetirizine	83881-51-0	C21H25ClN2O3	Pharmaceutical (Antiallergic, H1 receptor antagonist)
Chlorphenamine	132-22-9	C16H19ClN2	Pharmaceutical (Antiallergic, H1 receptor antagonist)
Clemastine	15686-51-8	C21H26ClNO	Pharmaceutical (Antiallergic, H1 receptor antagonist)
Cyproheptadine	129-03-3	C21H21N	Pharmaceutical (Antiallergic, H1 receptor antagonist)
Dexbrompheniramine	132-21-8	C16H19BrN2	Pharmaceutical (Antiallergic, H1 receptor antagonist)
Dexchlorpheniramine	25523-97-1	C16H19ClN2	Pharmaceutical (Antiallergic, H1 receptor antagonist)
Diphenhydramine	58-73-1	C17H21NO	Pharmaceutical (Antiallergic, H1 receptor antagonist)
Emedastine	87233-61-2	C17H26N4O	Pharmaceutical (Antiallergic, H1 receptor antagonist)
Epinastine	80012-43-7	C16H15N3	Pharmaceutical (Antiallergic, H1 receptor antagonist)
Fexofenadine	83799-24-0	C32H39NO4	Pharmaceutical (Antiallergic, H1 receptor antagonist)
Levocetirizine	130018-77-8	C21H25ClN2O3	Pharmaceutical (Antiallergic, H1 receptor antagonist)
Loratadine	79794-75-5	C22H23ClN2O2	Pharmaceutical (Antiallergic, H1 receptor antagonist)
Mepyramine (Pyrilamine)	91-84-9	C17H23N3O	Pharmaceutical (Antiallergic, H1 receptor antagonist)
Olopatadine	113806-05-6	C21H23NO3	Pharmaceutical (Antiallergic, H1 receptor antagonist)
Pheniramine	86-21-5	C16H20N2	Pharmaceutical (Antiallergic, H1 receptor antagonist)
Promethazine	60-87-7	C17H20N2S	Pharmaceutical (Antiallergic, H1 receptor antagonist)
Tripelennamine	91-81-6	C16H21N3	Pharmaceutical (Antiallergic, H1 receptor antagonist)
Tacrolimus	104987-11-3	C44H69NO12	Pharmaceutical (Antiallergic, Immunosuppressant, Calcineurin inhibitor)
Doxylamine	469-21-6	C17H22N2O	Pharmaceutical (Antiallergic, Sedative-hypnotic, H1 receptor antagonist)
Finasteride	98319-26-7	C23H36N2O2	Pharmaceutical (Antiandrogen, 5alpha-Reductase inhibitor)
Folic Acid	59-30-3	C19H19N7O6	Pharmaceutical (Anti-anemic, Hematopoietic, Supplement (folic acid))
Camazepam	36104-80-0	C19H18ClN3O3	Pharmaceutical (Antianxiety)
Ethyl Loflazepate	29177-84-2	C18H14ClFN2O3	Pharmaceutical (Antianxiety)
Medazepam	2898-12-6	C16H15ClN2	Pharmaceutical (Antianxiety)
Pinazepam	52463-83-9	C18H13ClN2O	Pharmaceutical (Antianxiety)
Lorazepam	846-49-1	C15H10Cl2N2O2	Pharmaceutical (Antianxiety, Anticonvulsant, Minor tranquilizer)
Phenobarbital	50-06-6	C12H12N2O3	Pharmaceutical (Antianxiety, Anticonvulsant, Sedative-hypnotic)
Vortioxetine	508233-74-7	C18H22N2S	Pharmaceutical (Antianxiety, Antidepressant, Serotonin receptor antagonist)
Ondansetron	99614-02-5	C18H19N3O	Pharmaceutical (Antianxiety, Anti-emetic, Antipsychotic, Serotonin 5-HT3 receptor antagonist)
Mebutamate	64-55-1	C10H20N2O4	Pharmaceutical (Antianxiety, Antihypertensive)
Clorazepic Acid	23887-31-2	C16H11ClN2O3	Pharmaceutical (Antianxiety, Minor tranquilizer)
Fludiazepam	3900-31-0	C16H12ClFN2O	Pharmaceutical (Antianxiety, Minor tranquilizer)
Alprazolam	28981-97-7	C17H13ClN4	Pharmaceutical (Antianxiety, Minor tranquilizer, Sedative-hypnotic)
Bromazepam	1812-30-2	C14H10BrN3O	Pharmaceutical (Antianxiety, Minor tranquilizer, Sedative-hypnotic)
Chlordiazepoxide	58-25-3	C16H14ClN3O	Pharmaceutical (Antianxiety, Minor tranquilizer, Sedative-hypnotic)

Table S20. Compound database for suspect screening (continued)

Compound Name	CAS	Molecular Formula	Category
Cloxazolam	24166-13-0	C17H14Cl2N2O2	Pharmaceutical (Antianxiety, Minor tranquilizer, Sedative-hypnotic)
Diazepam	439-14-5	C16H13ClN2O	Pharmaceutical (Antianxiety, Minor tranquilizer, Sedative-hypnotic)
Oxazolam	24143-17-7	C18H17ClN2O2	Pharmaceutical (Antianxiety, Minor tranquilizer, Sedative-hypnotic)
Buspirone	36505-84-7	C21H31N5O2	Pharmaceutical (Antianxiety, Minor tranquilizer, Serotonin 5-HT1A receptor agonist)
Clotiazepam	33671-46-4	C16H15ClN2OS	Pharmaceutical (Antianxiety, Sedative-hypnotic)
Meprobamate	57-53-4	C9H18N2O4	Pharmaceutical (Antianxiety, Sedative-hypnotic)
Propranolol	525-66-6	C16H21NO2	Pharmaceutical (Antiarrhythmic, Antihypertensive, beta-Adrenergic receptor antagonist)
Diltiazem	42399-41-7	C22H26N2O4S	Pharmaceutical (Antiarrhythmic, Antihypertensive, Vasodilator (coronary), Calcium channel blocker)
Acebutolol	37517-30-9	C18H28N2O4	Pharmaceutical (Antiarrhythmic, Antihypertensive, Vasodilator, beta1-Adrenergic receptor antagonist)
Atenolol	29122-68-7	C14H22N2O3	Pharmaceutical (Antiarrhythmic, Antihypertensive, Vasodilator, beta1-Adrenergic receptor antagonist)
Nadolol	42200-33-9	C17H27NO4	Pharmaceutical (Antiarrhythmic, Antihypertensive, Vasodilator, beta-Adrenergic receptor antagonist)
Pindolol	13523-86-9	C14H20N2O2	Pharmaceutical (Antiarrhythmic, Antihypertensive, Vasodilator, beta-Adrenergic receptor antagonist)
Quinidine	56-54-2	C20H24N2O2	Pharmaceutical (Antiarrhythmic, Antimalarial, Sodium channel blocker)
Esmolol	81147-92-4	C16H25NO4	Pharmaceutical (Antiarrhythmic, beta1-Adrenergic receptor antagonist)
Sotalol	3930-20-9	C12H20N2O3S	Pharmaceutical (Antiarrhythmic, beta-Adrenergic receptor antagonist, Potassium channel blocker)
Dofetilide	115256-11-6	C19H27N3O5S2	Pharmaceutical (Antiarrhythmic, Potassium channel blocker)
Ibutilide	122647-31-8	C20H36N2O3S	Pharmaceutical (Antiarrhythmic, Potassium channel blocker)
Disopyramide	3737-09-5	C21H29N3O	Pharmaceutical (Antiarrhythmic, Sodium channel blocker)
Flecainide	54143-55-4	C17H20F6N2O3	Pharmaceutical (Antiarrhythmic, Sodium channel blocker)
Mexiletine	31828-71-4	C11H17NO	Pharmaceutical (Antiarrhythmic, Sodium channel blocker)
Procainamide	51-06-9	C13H21N3O	Pharmaceutical (Antiarrhythmic, Sodium channel blocker)
Propafenone	54063-53-5	C21H27NO3	Pharmaceutical (Antiarrhythmic, Sodium channel blocker)
Oxprenolol	6452-71-7	C15H23NO3	Pharmaceutical (Antiarrhythmic, Vasodilator, beta-Adrenergic receptor antagonist)
Verapamil	52-53-9	C27H38N2O4	Pharmaceutical (Antiarrhythmic, Vasodilator, Calcium channel blocker)
Triamcinolone	124-94-7	C21H27FO6	Pharmaceutical (Antiasthmatic, Anti-inflammatory, Antirheumatic, Glucocorticoid receptor agonist)
Beclomethasone	4419-39-0	C22H29ClO5	Pharmaceutical (Antiasthmatic, Anti-inflammatory, Glucocorticoid receptor agonist)
Budesonide	51333-22-3	C25H34O6	Pharmaceutical (Antiasthmatic, Anti-inflammatory, Glucocorticoid receptor agonist)
Ciclesonide	126544-47-6	C32H44O7	Pharmaceutical (Antiasthmatic, Anti-inflammatory, Glucocorticoid receptor agonist)
Zileuton	111406-87-2	C11H12N2O2S	Pharmaceutical (Antiasthmatic, Arachidonate 5-lipoxygenase inhibitor)
Levalbuterol	34391-04-3	C13H21NO3	Pharmaceutical (Antiasthmatic, beta2-Adrenergic receptor agonist)
Arformoterol	67346-49-0	C19H24N2O4	Pharmaceutical (Antiasthmatic, Bronchodilator, beta2-Adrenergic receptor agonist)
Zafirlukast	107753-78-6	C31H33N3O6S	Pharmaceutical (Antiasthmatic, Leukotriene receptor antagonist)
Roflumilast	162401-32-3	C17H14Cl2F2N2O3	Pharmaceutical (Antiasthmatic, Phosphodiesterase IV inhibitor)
Rifabutin	72559-06-9	C46H62N4O11	Pharmaceutical (Antibacterial (antimycobacterial), RNA polymerase inhibitor)
Dapsone	80-08-0	C12H12N2O2S	Pharmaceutical (Antibacterial (leprostatic))
Ethionamide	536-33-4	C8H10N2S	Pharmaceutical (Antibacterial (tuberculostatic))
Isoniazid	54-85-3	C6H7N3O	Pharmaceutical (Antibacterial (tuberculostatic))
Pyrazinamide	98-96-4	C5H5N3O	Pharmaceutical (Antibacterial (tuberculostatic))
Ethambutol	74-55-5	C10H24N2O2	Pharmaceutical (Antibacterial (tuberculostatic), Arabinosyltransferase inhibitor)
Bedaquiline	843663-66-1	C32H31BrN2O2	Pharmaceutical (Antibacterial (tuberculostatic), ATP synthase inhibitor)
Cycloserine	68-41-7	C3H6N2O2	Pharmaceutical (Antibacterial (tuberculostatic), Cell wall biosynthesis inhibitor)
Cefovecin	234096-34-5	C17H19N5O6S2	Pharmaceutical (Antibacterial (veterinary))
Marbofloxacin	115550-35-1	C17H19FN4O4	Pharmaceutical (Antibacterial (veterinary))
Sulfachlorpyrazine	1672-91-9	C10H9ClN4O2S	Pharmaceutical (Antibacterial (veterinary))
Sulfachlorpyridazine	80-32-0	C10H9ClN4O2S	Pharmaceutical (Antibacterial (veterinary))
Tildipirosin	328898-40-4	C41H71N3O8	Pharmaceutical (Antibacterial (veterinary))
Tulathromycin A	217500-96-4	C41H79N3O12	Pharmaceutical (Antibacterial (veterinary))
Cefalonium	5575-21-3	C20H18N4O5S2	Pharmaceutical (Antibacterial (veterinary), Cell wall biosynthesis inhibitor)
Ceftiofur	80370-57-6	C19H17N5O7S3	Pharmaceutical (Antibacterial (veterinary), Cell wall biosynthesis inhibitor)
Danofloxacin	112398-08-0	C19H20FN3O3	Pharmaceutical (Antibacterial (veterinary), Nucleic acid biosynthesis inhibitor)
Difloxacin	98106-17-3	C21H19F2N3O3	Pharmaceutical (Antibacterial (veterinary), Nucleic acid biosynthesis inhibitor)
Enrofloxacin	93106-60-6	C19H22FN3O3	Pharmaceutical (Antibacterial (veterinary), Nucleic acid biosynthesis inhibitor)
Flumequine	42835-25-6	C14H12FNO3	Pharmaceutical (Antibacterial (veterinary), Nucleic acid biosynthesis inhibitor)
Orbifloxacin	113617-63-3	C19H20F3N3O3	Pharmaceutical (Antibacterial (veterinary), Nucleic acid biosynthesis inhibitor)
Pradofloxacin	195532-12-8	C21H21FN4O3	Pharmaceutical (Antibacterial (veterinary), Nucleic acid biosynthesis inhibitor)
Florfenicol	73231-34-2	C12H14Cl2FNO4S	Pharmaceutical (Antibacterial (veterinary), Protein biosynthesis inhibitor)
Gamithromycin	145435-72-9	C40H76N2O12	Pharmaceutical (Antibacterial (veterinary), Protein biosynthesis inhibitor)
Tiamulin	55297-95-5	C28H47NO4S	Pharmaceutical (Antibacterial (veterinary), Protein biosynthesis inhibitor)
Tilmicosin	108050-54-0	C46H80N2O13	Pharmaceutical (Antibacterial (veterinary), Protein biosynthesis inhibitor)
Tylosin	1401-69-0	C46H77NO17	Pharmaceutical (Antibacterial (veterinary), Protein biosynthesis inhibitor)
Carbadox	6804-07-5	C11H10N4O4	Pharmaceutical (Antibacterial)
Dalfopristin	112362-50-2	C34H50N4O9S	Pharmaceutical (Antibacterial)
Mafenide	138-39-6	C7H10N2O2S	Pharmaceutical (Antibacterial)
Nifurpirinol	13411-16-0	C12H10N2O4	Pharmaceutical (Antibacterial)
Nitrofurantoin	67-20-9	C8H6N4O5	Pharmaceutical (Antibacterial)
Nitrofurazone	59-87-0	C6H6N4O4	Pharmaceutical (Antibacterial)
Novobiocin	303-81-1	C31H36N2O11	Pharmaceutical (Antibacterial)

Table S20. Compound database for suspect screening (continued)

Compound Name	CAS	Molecular Formula	Category
Ormetoprim	6981-18-6	C14H18N4O2	Pharmaceutical (Antibacterial)
Sulfabenzamide	127-71-9	C13H12N2O3S	Pharmaceutical (Antibacterial)
Sulfameter	651-06-9	C11H12N4O3S	Pharmaceutical (Antibacterial)
Secnidazole	3366-95-8	C7H11N3O3	Pharmaceutical (Antibacterial, Amebicide, Antiprotozoal, DNA synthesis inhibitor)
Tetracycline	60-54-8	C22H24N2O8	Pharmaceutical (Antibacterial, Amebicide, Antirickettsial, Protein biosynthesis inhibitor)
Paromomycin	7542-37-2	C23H45N5O14	Pharmaceutical (Antibacterial, Amebicide, Protein biosynthesis inhibitor)
Natamycin	7681-93-8	C33H47NO13	Pharmaceutical (Antibacterial, Antifungal)
Monensin	17090-79-8	C36H62O11	Pharmaceutical (Antibacterial, Antifungal, Antiprotozoal)
Rifaximin	80621-81-4	C43H51N3O11	Pharmaceutical (Antibacterial, Antihyperammonemic)
Sulfadoxine	2447-57-6	C12H14N4O4S	Pharmaceutical (Antibacterial, Antimalarial, Folic acid biosynthesis inhibitor)
Daunorubicin	20830-81-3	C27H29NO10	Pharmaceutical (Antibacterial, Antineoplastic, Topoisomerase II inhibitor)
Epirubicin	56420-45-2	C27H29NO11	Pharmaceutical (Antibacterial, Antineoplastic, Topoisomerase II inhibitor)
Idarubicin	58957-92-9	C26H27NO9	Pharmaceutical (Antibacterial, Antineoplastic, Topoisomerase II inhibitor)
Furazolidone	67-45-8	C8H7N3O5	Pharmaceutical (Antibacterial, Antiprotozoal)
Metronidazole	443-48-1	C6H9N3O3	Pharmaceutical (Antibacterial, Antiprotozoal, DNA synthesis inhibitor)
Chlortetracycline	57-62-5	C22H23ClN2O8	Pharmaceutical (Antibacterial, Antiprotozoal, Protein biosynthesis inhibitor)
Chloramphenicol	56-75-7	C11H12Cl2N2O5	Pharmaceutical (Antibacterial, Antirickettsial, Protein biosynthesis inhibitor)
Methenamine	100-97-0	C6H12N4	Pharmaceutical (Antibacterial, Antiseptic)
Amoxicillin	26787-78-0	C16H19N3O5S	Pharmaceutical (Antibacterial, Cell wall biosynthesis inhibitor)
Ampicillin	69-53-4	C16H19N3O4S	Pharmaceutical (Antibacterial, Cell wall biosynthesis inhibitor)
Aztreonam	78110-38-0	C13H17N5O8S2	Pharmaceutical (Antibacterial, Cell wall biosynthesis inhibitor)
Cefaclor	53994-73-3	C15H14ClN3O4S	Pharmaceutical (Antibacterial, Cell wall biosynthesis inhibitor)
Cefadroxil	50370-12-2	C16H17N3O5S	Pharmaceutical (Antibacterial, Cell wall biosynthesis inhibitor)
Cefazolin	25953-19-9	C14H14N8O4S3	Pharmaceutical (Antibacterial, Cell wall biosynthesis inhibitor)
Cefdinir	91832-40-5	C14H13N5O5S2	Pharmaceutical (Antibacterial, Cell wall biosynthesis inhibitor)
Cefepime	88040-23-7	C19H24N6O5S2	Pharmaceutical (Antibacterial, Cell wall biosynthesis inhibitor)
Cefixime	79350-37-1	C16H15N5O7S2	Pharmaceutical (Antibacterial, Cell wall biosynthesis inhibitor)
Cefoperazone	62893-19-0	C25H27N9O8S2	Pharmaceutical (Antibacterial, Cell wall biosynthesis inhibitor)
Cefotaxime	63527-52-6	C16H17N5O7S2	Pharmaceutical (Antibacterial, Cell wall biosynthesis inhibitor)
Cefotetan	69712-56-7	C17H17N7O8S4	Pharmaceutical (Antibacterial, Cell wall biosynthesis inhibitor)
Cefoxitin	35607-66-0	C16H17N3O7S2	Pharmaceutical (Antibacterial, Cell wall biosynthesis inhibitor)
Cefpodoxime	80210-62-4	C15H17N5O6S2	Pharmaceutical (Antibacterial, Cell wall biosynthesis inhibitor)
Cefprozil	92665-29-7	C18H19N3O5S	Pharmaceutical (Antibacterial, Cell wall biosynthesis inhibitor)
Cefquinome	84957-30-2	C23H24N6O5S2	Pharmaceutical (Antibacterial, Cell wall biosynthesis inhibitor)
Ceftazidime	72558-82-8	C22H22N6O7S2	Pharmaceutical (Antibacterial, Cell wall biosynthesis inhibitor)
Ceftolozane	689293-68-3	C23H30N12O8S2	Pharmaceutical (Antibacterial, Cell wall biosynthesis inhibitor)
Ceftriaxone	73384-59-5	C18H18N8O7S3	Pharmaceutical (Antibacterial, Cell wall biosynthesis inhibitor)
Cefuroxime	55268-75-2	C16H16N4O8S	Pharmaceutical (Antibacterial, Cell wall biosynthesis inhibitor)
Cephalexin	15686-71-2	C16H17N3O4S	Pharmaceutical (Antibacterial, Cell wall biosynthesis inhibitor)
Cephapirin	21593-23-7	C17H17N3O6S2	Pharmaceutical (Antibacterial, Cell wall biosynthesis inhibitor)
Cloxacillin	61-72-3	C19H18ClN3O5S	Pharmaceutical (Antibacterial, Cell wall biosynthesis inhibitor)
Dicloxacillin	3116-76-5	C19H17Cl2N3O5S	Pharmaceutical (Antibacterial, Cell wall biosynthesis inhibitor)
Ertapenem	153832-46-3	C22H25N3O7S	Pharmaceutical (Antibacterial, Cell wall biosynthesis inhibitor)
Hetacillin	3511-16-8	C19H23N3O4S	Pharmaceutical (Antibacterial, Cell wall biosynthesis inhibitor)
Imipenem	64221-86-9	C12H17N3O4S	Pharmaceutical (Antibacterial, Cell wall biosynthesis inhibitor)
Meropenem	96036-03-2	C17H25N3O5S	Pharmaceutical (Antibacterial, Cell wall biosynthesis inhibitor)
Nafcillin	147-52-4	C21H22N2O5S	Pharmaceutical (Antibacterial, Cell wall biosynthesis inhibitor)
Oxacillin	66-79-5	C19H19N3O5S	Pharmaceutical (Antibacterial, Cell wall biosynthesis inhibitor)
Penicillin G	61-33-6	C16H18N2O4S	Pharmaceutical (Antibacterial, Cell wall biosynthesis inhibitor)
Penicillin V	87-08-1	C16H18N2O5S	Pharmaceutical (Antibacterial, Cell wall biosynthesis inhibitor)
Piperacillin	61477-96-1	C23H27N5O7S	Pharmaceutical (Antibacterial, Cell wall biosynthesis inhibitor)
Ticarcillin	34787-01-4	C15H16N2O6S2	Pharmaceutical (Antibacterial, Cell wall biosynthesis inhibitor)
Avibactam	1192500-31-4	C7H11N3O6S	Pharmaceutical (Antibacterial, Cell wall biosynthesis inhibitor, beta-Lactamase inhibitor)
Clavulanic Acid	58001-44-8	C8H9NO5	Pharmaceutical (Antibacterial, Cell wall biosynthesis inhibitor, beta-Lactamase inhibitor)
Sulfanitran	122-16-7	C14H13N3O5S	Pharmaceutical (Antibacterial, Coccidiostat (for poultry))
Sulfacetamide	144-80-9	C8H10N2O3S	Pharmaceutical (Antibacterial, Folic acid biosynthesis inhibitor)
Sulfadiazine	68-35-9	C10H10N4O2S	Pharmaceutical (Antibacterial, Folic acid biosynthesis inhibitor)
Sulfadimethoxine	122-11-2	C12H14N4O4S	Pharmaceutical (Antibacterial, Folic acid biosynthesis inhibitor)
Sulfaethoxypyridazine	963-14-4	C12H14N4O3S	Pharmaceutical (Antibacterial, Folic acid biosynthesis inhibitor)
Sulfaguanidine	57-67-0	C7H10N4O2S	Pharmaceutical (Antibacterial, Folic acid biosynthesis inhibitor)
Sulfamerazine	127-79-7	C11H12N4O2S	Pharmaceutical (Antibacterial, Folic acid biosynthesis inhibitor)
Sulfamethazine	57-68-1	C12H14N4O2S	Pharmaceutical (Antibacterial, Folic acid biosynthesis inhibitor)
Sulfamethizole	144-82-1	C9H10N4O2S2	Pharmaceutical (Antibacterial, Folic acid biosynthesis inhibitor)
Sulfamethoxazole	723-46-6	C10H11N3O3S	Pharmaceutical (Antibacterial, Folic acid biosynthesis inhibitor)
Sulfamethoxypyridazine	80-35-3	C11H12N4O3S	Pharmaceutical (Antibacterial, Folic acid biosynthesis inhibitor)
Sulfamonomethoxine	1220-83-3	C11H12N4O3S	Pharmaceutical (Antibacterial, Folic acid biosynthesis inhibitor)
Sulfamoxole	729-99-7	C11H13N3O3S	Pharmaceutical (Antibacterial, Folic acid biosynthesis inhibitor)

Table S20. Compound database for suspect screening (continued)

Compound Name	CAS	Molecular Formula	Category
Sulfanilamide	63-74-1	C6H8N2O2S	Pharmaceutical (Antibacterial, Folic acid biosynthesis inhibitor)
Sulfapyridine	144-83-2	C11H11N3O2S	Pharmaceutical (Antibacterial, Folic acid biosynthesis inhibitor)
Sulfathiazole	72-14-0	C9H9N3O2S2	Pharmaceutical (Antibacterial, Folic acid biosynthesis inhibitor)
Sulfisomidine	515-64-0	C12H14N4O2S	Pharmaceutical (Antibacterial, Folic acid biosynthesis inhibitor)
Sulfisoxazole	127-69-5	C11H13N3O3S	Pharmaceutical (Antibacterial, Folic acid biosynthesis inhibitor)
Trimethoprim	738-70-5	C14H18N4O3	Pharmaceutical (Antibacterial, Folic acid biosynthesis inhibitor)
Virginiamycin M1	21411-53-0	C28H35N3O7	Pharmaceutical (Antibacterial, Food additive (veterinary))
Besifloxacin	141388-76-3	C19H21ClFN3O3	Pharmaceutical (Antibacterial, Nucleic acid biosynthesis inhibitor)
Ciprofloxacin	85721-33-1	C17H18FN3O3	Pharmaceutical (Antibacterial, Nucleic acid biosynthesis inhibitor)
Clinafloxacin	105956-97-6	C17H17ClFN3O3	Pharmaceutical (Antibacterial, Nucleic acid biosynthesis inhibitor)
Delafloxacin	189279-58-1	C18H12ClF3N4O4	Pharmaceutical (Antibacterial, Nucleic acid biosynthesis inhibitor)
Enoxacin	74011-58-8	C15H17FN4O3	Pharmaceutical (Antibacterial, Nucleic acid biosynthesis inhibitor)
Finafloxacin	209342-40-5	C20H19FN4O4	Pharmaceutical (Antibacterial, Nucleic acid biosynthesis inhibitor)
Fleroxacin	79660-72-3	C17H18F3N3O3	Pharmaceutical (Antibacterial, Nucleic acid biosynthesis inhibitor)
Gatifloxacin	112811-59-3	C19H22FN3O4	Pharmaceutical (Antibacterial, Nucleic acid biosynthesis inhibitor)
Gemifloxacin	175463-14-6	C18H20FN5O4	Pharmaceutical (Antibacterial, Nucleic acid biosynthesis inhibitor)
Levofloxacin	100986-85-4	C18H20FN3O4	Pharmaceutical (Antibacterial, Nucleic acid biosynthesis inhibitor)
Lomefloxacin	98079-51-7	C17H19F2N3O3	Pharmaceutical (Antibacterial, Nucleic acid biosynthesis inhibitor)
Moxifloxacin	151096-09-2	C21H24FN3O4	Pharmaceutical (Antibacterial, Nucleic acid biosynthesis inhibitor)
Nalidixic Acid	389-08-2	C12H12N2O3	Pharmaceutical (Antibacterial, Nucleic acid biosynthesis inhibitor)
Norfloxacin	70458-96-7	C16H18FN3O3	Pharmaceutical (Antibacterial, Nucleic acid biosynthesis inhibitor)
Ofloxacin	82419-36-1	C18H20FN3O4	Pharmaceutical (Antibacterial, Nucleic acid biosynthesis inhibitor)
Ozenoxacin	245765-41-7	C21H21N3O3	Pharmaceutical (Antibacterial, Nucleic acid biosynthesis inhibitor)
Sarafloxacin	98105-99-8	C20H17F2N3O3	Pharmaceutical (Antibacterial, Nucleic acid biosynthesis inhibitor)
Amikacin	37517-28-5	C22H43N5O13	Pharmaceutical (Antibacterial, Protein biosynthesis inhibitor)
Apramycin	37321-09-8	C21H41N5O11	Pharmaceutical (Antibacterial, Protein biosynthesis inhibitor)
Azithromycin	83905-01-5	C38H72N2O12	Pharmaceutical (Antibacterial, Protein biosynthesis inhibitor)
Clarithromycin	81103-11-9	C38H69NO13	Pharmaceutical (Antibacterial, Protein biosynthesis inhibitor)
Clindamycin	18323-44-9	C18H33ClN2O5S	Pharmaceutical (Antibacterial, Protein biosynthesis inhibitor)
Demeclocycline	127-33-3	C21H21ClN2O8	Pharmaceutical (Antibacterial, Protein biosynthesis inhibitor)
Doxycycline	564-25-0	C22H24N2O8	Pharmaceutical (Antibacterial, Protein biosynthesis inhibitor)
Erythromycin	114-07-8	C37H67NO13	Pharmaceutical (Antibacterial, Protein biosynthesis inhibitor)
Fusidic Acid	6990-06-3	C31H48O6	Pharmaceutical (Antibacterial, Protein biosynthesis inhibitor)
Gentamicin	1403-66-3	C21H43N5O7	Pharmaceutical (Antibacterial, Protein biosynthesis inhibitor)
Hygromycin B	31282-04-9	C20H37N3O13	Pharmaceutical (Antibacterial, Protein biosynthesis inhibitor)
Kanamycin A	59-01-8	C18H36N4O11	Pharmaceutical (Antibacterial, Protein biosynthesis inhibitor)
Leucomycin V	39405-35-1	C35H59NO13	Pharmaceutical (Antibacterial, Protein biosynthesis inhibitor)
Lincomycin	154-21-2	C18H34N2O6S	Pharmaceutical (Antibacterial, Protein biosynthesis inhibitor)
Linezolid	165800-03-3	C16H20FN3O4	Pharmaceutical (Antibacterial, Protein biosynthesis inhibitor)
Minocycline	10118-90-8	C23H27N3O7	Pharmaceutical (Antibacterial, Protein biosynthesis inhibitor)
Neomycin	119-04-0	C23H46N6O13	Pharmaceutical (Antibacterial, Protein biosynthesis inhibitor)
Oleandomycin	3922-90-5	C35H61NO12	Pharmaceutical (Antibacterial, Protein biosynthesis inhibitor)
Oxytetracycline	79-57-2	C22H24N2O9	Pharmaceutical (Antibacterial, Protein biosynthesis inhibitor)
Pirlimycin	79548-73-5	C17H31ClN2O5S	Pharmaceutical (Antibacterial, Protein biosynthesis inhibitor)
Retapamulin	224452-66-8	C30H47NO4S	Pharmaceutical (Antibacterial, Protein biosynthesis inhibitor)
Roxithromycin	80214-83-1	C41H76N2O15	Pharmaceutical (Antibacterial, Protein biosynthesis inhibitor)
Spectinomycin	1695-77-8	C14H24N2O7	Pharmaceutical (Antibacterial, Protein biosynthesis inhibitor)
Tedizolid	856866-72-3	C17H15FN6O3	Pharmaceutical (Antibacterial, Protein biosynthesis inhibitor)
Tigecycline	220620-09-7	C29H39N5O8	Pharmaceutical (Antibacterial, Protein biosynthesis inhibitor)
Tobramycin	32986-56-4	C18H37N5O9	Pharmaceutical (Antibacterial, Protein biosynthesis inhibitor)
Rifampicin (Rifampin)	13292-46-1	C43H58N4O12	Pharmaceutical (Antibacterial, RNA polymerase inhibitor)
Rifapentine	61379-65-5	C47H64N4O12	Pharmaceutical (Antibacterial, RNA polymerase inhibitor)
Mupirocin	12650-69-0	C26H44O9	Pharmaceutical (Antibacterial, tRNA Ligase inhibitor)
Lamotrigine	84057-84-1	C9H7Cl2N5	Pharmaceutical (Anti-bipolar disorder, Anticonvulsant, Antiepileptic)
Chenodiol	474-25-9	C24H40O4	Pharmaceutical (Anticholelithogenic)
Ursodiol	128-13-2	C24H40O4	Pharmaceutical (Anticholelithogenic, Choleretic)
Treprostinil	81846-19-7	C23H34O5	Pharmaceutical (Anticoagulant, Antihypertensive, Vasodilator, Platelet aggregation inhibitor, Prostaglandin I2 receptor agonist)
Apixaban	503612-47-3	C25H25N5O4	Pharmaceutical (Anticoagulant, Antithrombotic, Factor Xa inhibitor)
Clopidogrel	113665-84-2	C16H16ClNO2S	Pharmaceutical (Anticoagulant, Platelet aggregation inhibitor, Purinergic receptor P2Y12 antagonist)
Ticlopidine	55142-85-3	C14H14ClNS	Pharmaceutical (Anticoagulant, Platelet aggregation inhibitor, Purinergic receptor P2Y12 antagonist)
Argatroban	74863-84-6	C23H36N6O5S	Pharmaceutical (Anticoagulant, Thrombin inhibitor)
Dabigatran	211914-51-1	C25H25N7O3	Pharmaceutical (Anticoagulant, Thrombin inhibitor)
Acenocoumarol	152-72-7	C19H15NO6	Pharmaceutical (Anticoagulant, Vitamin K antagonist)
Warfarin	81-81-2	C19H16O4	Pharmaceutical (Anticoagulant, Vitamin K antagonist)
Methsuximide	77-41-8	C12H13NO2	Pharmaceutical (Anticonvulsant)
Valproic Acid	99-66-1	C8H16O2	Pharmaceutical (Anticonvulsant)
Tiletamine	14176-49-9	C12H17NOS	Pharmaceutical (Anticonvulsant, Anesthetic)

Table S20. Compound database for suspect screening (continued)

Compound Name	CAS	Molecular Formula	Category
Clonazepam	1622-61-3	C15H10ClN3O3	Pharmaceutical (Anticonvulsant, Antiepileptic)
Eslicarbazepine	104746-04-5	C15H14N2O2	Pharmaceutical (Anticonvulsant, Antiepileptic)
Fosphenytoin	93390-81-9	C16H15N2O6P	Pharmaceutical (Anticonvulsant, Antiepileptic)
Gabapentin	60142-96-3	C9H17NO2	Pharmaceutical (Anticonvulsant, Antiepileptic)
Oxcarbazepine	28721-07-5	C15H12N2O2	Pharmaceutical (Anticonvulsant, Antiepileptic)
Tiagabine	115103-54-3	C20H25NO2S2	Pharmaceutical (Anticonvulsant, Antiepileptic)
Topiramate	97240-79-4	C12H21NO8S	Pharmaceutical (Anticonvulsant, Antiepileptic, AMPA receptor antagonist)
Zonisamide	68291-97-4	C8H8N2O3S	Pharmaceutical (Anticonvulsant, Antiepileptic, Antiparkinsonian)
Melatonin	73-31-4	C13H16N2O2	Pharmaceutical (Anticonvulsant, Cerebroprotective, Sedative-hypnotic, Melatonin receptor agonist)
Clobazam	22316-47-8	C16H13ClN2O2	Pharmaceutical (Anticonvulsant, Minor tranquilizer)
Piracetam	7491-74-9	C6H10N2O2	Pharmaceutical (Anticonvulsant, Nootropi)
Brivaracetam	357336-20-0	C11H20N2O2	Pharmaceutical (Anticonvulsant, Pain relief)
Pregabalin	148553-50-8	C8H17NO2	Pharmaceutical (Anticonvulsant, Pain relief)
Mephobarbital	115-38-8	C13H14N2O3	Pharmaceutical (Anticonvulsant, Sedative-hypnotic)
Nitrazepam	146-22-5	C15H11N3O3	Pharmaceutical (Anticonvulsant, Sedative-hypnotic)
Amitriptyline	50-48-6	C20H23N	Pharmaceutical (Antidepressant)
Daledalin	22136-27-2	C19H24N2	Pharmaceutical (Antidepressant)
Nefazodone	83366-66-9	C25H32ClN5O2	Pharmaceutical (Antidepressant)
Protriptyline	438-60-8	C19H21N	Pharmaceutical (Antidepressant)
Atipamezole	104054-27-5	C14H16N2	Pharmaceutical (Antidepressant, alpha2-Adrenergic receptor antagonist)
Selegiline	14611-51-9	C13H17N	Pharmaceutical (Antidepressant, Antiparkinsonian, Monoamine oxidase B (MAO-B) inhibitor)
Sulpiride	15676-16-1	C15H23N3O4S	Pharmaceutical (Antidepressant, Antipsychotic, Anti-ulcerative, Dopamine receptor antagonist)
Doxepin	1668-19-5	C19H21NO	Pharmaceutical (Antidepressant, H1 receptor antagonist, Serotonin-noradrenaline reuptake inhibitor (SNRI))
Isocarboxazid	59-63-2	C12H13N3O2	Pharmaceutical (Antidepressant, Monoamine oxidase (MAO) inhibitor)
Phenelzine	51-71-8	C8H12N2	Pharmaceutical (Antidepressant, Monoamine oxidase (MAO) inhibitor)
Tranylcypromine	155-09-9	C9H11N	Pharmaceutical (Antidepressant, Monoamine oxidase (MAO) inhibitor)
Bupropion	34911-55-2	C13H18ClNO	Pharmaceutical (Antidepressant, Noradrenalin and dopamine reuptake inhibitor)
Pemoline	2152-34-3	C9H8N2O2	Pharmaceutical (Antidepressant, Sedative-hypnotic, Stimulant (central))
Maprotiline	10262-69-8	C20H23N	Pharmaceutical (Antidepressant, Selective noradrenaline reuptake inhibitor (NRI))
Citalopram	59729-33-8	C20H21FN2O	Pharmaceutical (Antidepressant, Selective serotonin reuptake inhibitor (SSRI))
Escitalopram	128196-01-0	C20H21FN2O	Pharmaceutical (Antidepressant, Selective serotonin reuptake inhibitor (SSRI))
Fluoxetine	54910-89-3	C17H18F3NO	Pharmaceutical (Antidepressant, Selective serotonin reuptake inhibitor (SSRI))
Fluvoxamine	54739-18-3	C15H21F3N2O2	Pharmaceutical (Antidepressant, Selective serotonin reuptake inhibitor (SSRI))
Paroxetine	61869-08-7	C19H20FNO3	Pharmaceutical (Antidepressant, Selective serotonin reuptake inhibitor (SSRI))
Sertraline	79617-96-2	C17H17Cl2N	Pharmaceutical (Antidepressant, Selective serotonin reuptake inhibitor (SSRI))
Flibanserin	167933-07-5	C20H21F3N4O	Pharmaceutical (Antidepressant, Serotonin 5-HT1A receptor agonist, Serotonin 5-HT2 receptor antagonist)
Vilazodone	163521-12-8	C26H27N5O2	Pharmaceutical (Antidepressant, Serotonin antagonist and reuptake inhibitor (SARI))
Mirtazapine	85650-52-8	C17H19N3	Pharmaceutical (Antidepressant, Serotonin receptor antagonist)
Amoxapine	14028-44-5	C17H16ClN3O	Pharmaceutical (Antidepressant, Serotonin-noradrenaline reuptake inhibitor (SNRI))
Clomipramine	303-49-1	C19H23ClN2	Pharmaceutical (Antidepressant, Serotonin-noradrenaline reuptake inhibitor (SNRI))
Desipramine	50-47-5	C18H22N2	Pharmaceutical (Antidepressant, Serotonin-noradrenaline reuptake inhibitor (SNRI))
Duloxetine	116539-59-4	C18H19NOS	Pharmaceutical (Antidepressant, Serotonin-noradrenaline reuptake inhibitor (SNRI))
Imipramine	50-49-7	C19H24N2	Pharmaceutical (Antidepressant, Serotonin-noradrenaline reuptake inhibitor (SNRI))
Levomilnacipran	96847-54-0	C15H22N2O	Pharmaceutical (Antidepressant, Serotonin-noradrenaline reuptake inhibitor (SNRI))
Milnacipran	92623-85-3	C15H22N2O	Pharmaceutical (Antidepressant, Serotonin-noradrenaline reuptake inhibitor (SNRI))
Trimipramine	739-71-9	C20H26N2	Pharmaceutical (Antidepressant, Serotonin-noradrenaline reuptake inhibitor (SNRI))
Venlafaxine	93413-69-5	C17H27NO2	Pharmaceutical (Antidepressant, Serotonin-noradrenaline reuptake inhibitor (SNRI))
Nortriptyline	72-69-5	C19H21N	Pharmaceutical (Antidepressant, Serotonin-noradrenaline reuptake inhibitor (SNRI)) / Pharmaceutical TP (Amitriptyline)
Desvenlafaxine (O-Desmethyl Venlafaxine)	93413-62-8	C16H25NO2	Pharmaceutical (Antidepressant, Serotonin-noradrenaline reuptake inhibitor (SNRI)) / Pharmaceutical TP (Venlafaxine)
Acarbose	56180-94-0	C25H43NO18	Pharmaceutical (Antidiabetic, alpha-Glucosidase inhibitor)
Alogliptin	850649-61-5	C18H21N5O2	Pharmaceutical (Antidiabetic, Dipeptidyl peptidase-4 (DPP-4) inhibitor)
Linagliptin	668270-12-0	C25H28N8O2	Pharmaceutical (Antidiabetic, Dipeptidyl peptidase-4 (DPP-4) inhibitor)
Saxagliptin	361442-04-8	C18H25N3O2	Pharmaceutical (Antidiabetic, Dipeptidyl peptidase-4 (DPP-4) inhibitor)
Sitagliptin	486460-32-6	C16H15F6N5O	Pharmaceutical (Antidiabetic, Dipeptidyl peptidase-4 (DPP-4) inhibitor)
Vildagliptin	274901-16-5	C17H25N3O2	Pharmaceutical (Antidiabetic, Dipeptidyl peptidase-4 (DPP-4) inhibitor)
Glyburide	10238-21-8	C23H28ClN3O5S	Pharmaceutical (Antidiabetic, Hypoglycemic)
Metformin	657-24-9	C4H11N5	Pharmaceutical (Antidiabetic, Hypoglycemic, AMP kinase activator)
Chlorpropamide	94-20-2	C10H13ClN2O3S	Pharmaceutical (Antidiabetic, Hypoglycemic, Sulfonylurea receptor agonist)
Glimepiride	93479-97-1	C24H34N4O5S	Pharmaceutical (Antidiabetic, Hypoglycemic, Sulfonylurea receptor agonist)
Glipizide	29094-61-9	C21H27N5O4S	Pharmaceutical (Antidiabetic, Hypoglycemic, Sulfonylurea receptor agonist)
Nateglinide	105816-04-4	C19H27NO3	Pharmaceutical (Antidiabetic, Hypoglycemic, Sulfonylurea receptor agonist)
Repaglinide	135062-02-1	C27H36N2O4	Pharmaceutical (Antidiabetic, Hypoglycemic, Sulfonylurea receptor agonist)
Tolazamide	1156-19-0	C14H21N3O3S	Pharmaceutical (Antidiabetic, Hypoglycemic, Sulfonylurea receptor agonist)
Tolbutamide	64-77-7	C12H18N2O3S	Pharmaceutical (Antidiabetic, Hypoglycemic, Sulfonylurea receptor agonist)
Pioglitazone	111025-46-8	C19H20N2O3S	Pharmaceutical (Antidiabetic, Peroxisome proliferator-activated receptor (PPAR) gamma agonist)
Rosiglitazone	122320-73-4	C18H19N3O3S	Pharmaceutical (Antidiabetic, Peroxisome proliferator-activated receptor (PPAR) gamma agonist)
Canagliflozin	842133-18-0	C24H25FO5S	Pharmaceutical (Antidiabetic, SGLT-2 inhibitor)

Table S20. Compound database for suspect screening (continued)

Compound Name	CAS	Molecular Formula	Category
Dapagliflozin	461432-26-8	C21H25ClO6	Pharmaceutical (Antidiabetic, SGLT-2 inhibitor)
Empagliflozin	864070-44-0	C23H27ClO7	Pharmaceutical (Antidiabetic, SGLT-2 inhibitor)
Ertugliflozin	1210344-57-2	C22H25ClO7	Pharmaceutical (Antidiabetic, SGLT-2 inhibitor)
Difenoxin	28782-42-5	C28H28N2O2	Pharmaceutical (Antidiarrheal, Antiperistaltic, Opioid receptor agonist)
Diphenoxylate	915-30-0	C30H32N2O2	Pharmaceutical (Antidiarrheal, Antiperistaltic, Opioid receptor agonist)
Loperamide	53179-11-6	C29H33ClN2O2	Pharmaceutical (Antidiarrheal, Antiperistaltic, Opioid receptor agonist)
Eluxadoline	864821-90-9	C32H35N5O5	Pharmaceutical (Antidiarrheal, Opioid receptor agonist/antagonist)
Telotristat	1033805-28-5	C25H22ClF3N6O3	Pharmaceutical (Antidiarrheal, Tryptophan hydroxylase inhibitor)
Acetylcysteine	616-91-1	C5H9NO3S	Pharmaceutical (Antidote (acetaminophen), Mucolytic)
Leucovorin	58-05-9	C20H23N7O7	Pharmaceutical (Antidote (antifolate))
Levoleucovorin	68538-85-2	C20H23N7O7	Pharmaceutical (Antidote (antifolate), Antineoplastic (enhancer))
Flumazenil	78755-81-4	C15H14FN3O3	Pharmaceutical (Antidote (benzodiazepines), Respiratory stimulant)
Deferasirox	201530-41-8	C21H15N3O4	Pharmaceutical (Antidote (iron), Chelating agent)
Deferiprone	30652-11-0	C7H9NO2	Pharmaceutical (Antidote (iron), Chelating agent)
Deferoxamine	70-51-9	C25H48N6O8	Pharmaceutical (Antidote (iron), Chelating agent)
Succimer	304-55-2	C4H6O4S2	Pharmaceutical (Antidote (lead), Diagnostic aid)
Tiopronin	1953-02-2	C5H9NO3S	Pharmaceutical (Antidote (mercury), Liver function improving agent)
Nalorphine	62-67-9	C19H21NO3	Pharmaceutical (Antidote (morphine), Opioid receptor agonist/antagonist)
Dexrazoxane	24584-09-6	C11H16N4O4	Pharmaceutical (Antidote)
Penicillamine	52-67-5	C5H11NO2S	Pharmaceutical (Antidote, Antirheumatic, Chelating agent)
Diprenorphine	14357-78-9	C26H35NO4	Pharmaceutical (Antidote, Narcotic antagonist, Opioid receptor agonist/antagonist)
Nalbuphine	20594-83-6	C21H27NO4	Pharmaceutical (Antidote, Narcotic antagonist, Opioid receptor agonist/antagonist)
Silodosin	160970-54-7	C25H32F3N3O4	Pharmaceutical (Antidysuria, alpha1-Adrenergic receptor antagonist)
Tamsulosin	106133-20-4	C20H28N2O5S	Pharmaceutical (Antidysuria, alpha1-Adrenergic receptor antagonist)
Alfuzosin	81403-80-7	C19H27N5O4	Pharmaceutical (Antidysuria, Antihypertensive, alpha1-Adrenergic receptor antagonist)
Terazosin	63590-64-7	C19H25N5O4	Pharmaceutical (Antidysuria, Antihypertensive, alpha1-Adrenergic receptor antagonist)
Trimethobenzamide	138-56-7	C21H28N2O5	Pharmaceutical (Anti-emetic)
Aprepitant	170729-80-3	C23H21F7N4O3	Pharmaceutical (Anti-emetic, Antidepressant, Antipsychotic, Neurokinin NK1 antagonist)
Meclizine	569-65-3	C25H27ClN2	Pharmaceutical (Anti-emetic, H1 receptor antagonist)
Naldemedine	916072-89-4	C32H34N4O6	Pharmaceutical (Anti-emetic, Laxative, Opioid receptor antagonist)
Fosaprepitant	172673-20-0	C23H22F7N4O6P	Pharmaceutical (Anti-emetic, Neurokinin NK1 antagonist)
Maropitant	147116-67-4	C32H40N2O	Pharmaceutical (Anti-emetic, Neurokinin NK1 antagonist)
Rolapitant	552292-08-7	C25H26F6N2O2	Pharmaceutical (Anti-emetic, Neurokinin NK1 antagonist)
Domperidone	57808-66-9	C22H24ClN5O2	Pharmaceutical (Anti-emetic, Prokinetic, Dopamine D2 receptor antagonist)
Metoclopramide	364-62-5	C14H22ClN3O2	Pharmaceutical (Anti-emetic, Prokinetic, Dopamine D2 receptor antagonist)
Alosetron	122852-42-0	C17H18N4O	Pharmaceutical (Anti-emetic, Serotonin 5-HT3 receptor antagonist)
Granisetron	109889-09-0	C18H24N4O	Pharmaceutical (Anti-emetic, Serotonin 5-HT3 receptor antagonist)
Palonosetron	135729-56-5	C19H24N2O	Pharmaceutical (Anti-emetic, Serotonin 5-HT3 receptor antagonist)
Danazol	17230-88-5	C22H27NO2	Pharmaceutical (Anti-endometriosis, Anterior pituitary suppressant)
Dienogest	65928-58-7	C20H25NO2	Pharmaceutical (Anti-endometriosis, Contraceptive, Replenisher (progesterone), Progesterone receptor agonist)
Ethosuximide	77-67-8	C7H11NO2	Pharmaceutical (Antiepileptic)
Ethotoin	86-35-1	C11H12N2O2	Pharmaceutical (Antiepileptic)
Felbamate	25451-15-4	C11H14N2O4	Pharmaceutical (Antiepileptic)
Levetiracetam	102767-28-2	C8H14N2O2	Pharmaceutical (Antiepileptic)
Phenytoin	57-41-0	C15H12N2O2	Pharmaceutical (Antiepileptic)
Primidone	125-33-7	C12H14N2O2	Pharmaceutical (Antiepileptic)
Retigabine (Ezogabine)	150812-12-7	C16H18FN3O2	Pharmaceutical (Antiepileptic)
Rufinamide	106308-44-5	C10H8F2N4O	Pharmaceutical (Antiepileptic)
Perampanel	380917-97-5	C23H15N3O	Pharmaceutical (Antiepileptic, AMPA receptor antagonist)
Lacosamide	175481-36-4	C13H18N2O3	Pharmaceutical (Antiepileptic, Pain relief (neuropathic))
Aminocaproic Acid	60-32-2	C6H13NO2	Pharmaceutical (Antifibrinolytic, Anti-inflammatory, Plasmin inhibitor)
Tranexamic Acid	1197-18-8	C8H15NO2	Pharmaceutical (Antifibrinolytic, Anti-inflammatory, Plasmin inhibitor)
Nintedanib	656247-17-5	C31H33N5O4	Pharmaceutical (Antifibrosis, Tyrosine kinase inhibitor)
Atovaquone	95233-18-4	C22H19ClO3	Pharmaceutical (Antifungal (pneumocystic))
Ciclopirox	29342-05-0	C12H17NO2	Pharmaceutical (Antifungal)
Griseofulvin	126-07-8	C17H17ClO6	Pharmaceutical (Antifungal)
Luliconazole	187164-19-8	C14H9Cl2N3S2	Pharmaceutical (Antifungal)
Nystatin	1400-61-9	C47H75NO17	Pharmaceutical (Antifungal)
Sertaconazole	99592-32-2	C20H15Cl3N2OS	Pharmaceutical (Antifungal)
Pentamidine	100-33-4	C19H24N4O2	Pharmaceutical (Antifungal, Antiprotozoal)
Amphotericin B	1397-89-3	C47H73NO17	Pharmaceutical (Antifungal, Cell membrane function inhibitor)
Butenafine	101828-21-1	C23H27N	Pharmaceutical (Antifungal, Ergosterol biosynthesis inhibitor)
Butoconazole	64872-76-0	C19H17Cl3N2S	Pharmaceutical (Antifungal, Ergosterol biosynthesis inhibitor)
Clotrimazole	23593-75-1	C22H17ClN2	Pharmaceutical (Antifungal, Ergosterol biosynthesis inhibitor)
Econazole	27220-47-9	C18H15Cl3N2O	Pharmaceutical (Antifungal, Ergosterol biosynthesis inhibitor)
Efinaconazole	164650-44-6	C18H22F2N4O	Pharmaceutical (Antifungal, Ergosterol biosynthesis inhibitor)
Fluconazole	86386-73-4	C13H12F2N6O	Pharmaceutical (Antifungal, Ergosterol biosynthesis inhibitor)

Table S20. Compound database for suspect screening (continued)

Compound Name	CAS	Molecular Formula	Category
Itraconazole	84625-61-6	C35H38Cl2N8O4	Pharmaceutical (Antifungal, Ergosterol biosynthesis inhibitor)
Ketoconazole	65277-42-1	C26H28Cl2N4O4	Pharmaceutical (Antifungal, Ergosterol biosynthesis inhibitor)
Miconazole	22916-47-8	C18H14Cl4N2O	Pharmaceutical (Antifungal, Ergosterol biosynthesis inhibitor)
Naftifine	65472-88-0	C21H21N	Pharmaceutical (Antifungal, Ergosterol biosynthesis inhibitor)
Oxiconazole	64211-45-6	C18H13Cl4N3O	Pharmaceutical (Antifungal, Ergosterol biosynthesis inhibitor)
Posaconazole	171228-49-2	C37H42F2N8O4	Pharmaceutical (Antifungal, Ergosterol biosynthesis inhibitor)
Sulconazole	61318-90-9	C18H15Cl3N2S	Pharmaceutical (Antifungal, Ergosterol biosynthesis inhibitor)
Terbinafine	91161-71-6	C21H25N	Pharmaceutical (Antifungal, Ergosterol biosynthesis inhibitor)
Terconazole	67915-31-5	C26H31Cl2N5O3	Pharmaceutical (Antifungal, Ergosterol biosynthesis inhibitor)
Tioconazole	65899-73-2	C16H13Cl3N2OS	Pharmaceutical (Antifungal, Ergosterol biosynthesis inhibitor)
Tolnaftate	2398-96-1	C19H17NOS	Pharmaceutical (Antifungal, Ergosterol biosynthesis inhibitor)
Voriconazole	137234-62-9	C16H14F3N5O	Pharmaceutical (Antifungal, Ergosterol biosynthesis inhibitor)
Flucytosine	2022-85-7	C4H4FN3O	Pharmaceutical (Antifungal, Nucleic acid biosynthesis inhibitor)
Levobunolol	47141-42-4	C17H25NO3	Pharmaceutical (Antiglaucoma, alpha1/beta-Adrenergic receptor antagonist)
Brimonidine	59803-98-4	C11H10BrN5	Pharmaceutical (Antiglaucoma, alpha2-Adrenergic receptor agonist)
Dichlorphenamide	120-97-8	C6H6Cl2N2O4S2	Pharmaceutical (Antiglaucoma, Antiepileptic, Carbonic anhydrase inhibitor)
Apraclonidine	66711-21-5	C9H10Cl2N4	Pharmaceutical (Antiglaucoma, Antihypertensive (intraocular), alpha2-Adrenergic receptor agonist)
Betaxolol	63659-18-7	C18H29NO3	Pharmaceutical (Antiglaucoma, Antihypertensive, beta1-Adrenergic receptor antagonist)
Metipranolol	22664-55-7	C17H27NO4	Pharmaceutical (Antiglaucoma, Antihypertensive, beta-Adrenergic receptor antagonist)
Timolol	26839-75-8	C13H24N4O3S	Pharmaceutical (Antiglaucoma, beta-Adrenergic rceptor antagonist)
Carteolol	51781-06-7	C16H24N2O3	Pharmaceutical (Antiglaucoma, beta-Adrenergic receptor antagonist)
Acetazolamide	59-66-5	C4H6N4O3S2	Pharmaceutical (Antiglaucoma, Carbonic anhydrase inhibitor)
Brinzolamide	138890-62-7	C12H21N3O5S3	Pharmaceutical (Antiglaucoma, Carbonic anhydrase inhibitor)
Dorzolamide	120279-96-1	C10H16N2O4S3	Pharmaceutical (Antiglaucoma, Carbonic anhydrase inhibitor)
Methazolamide	554-57-4	C5H8N4O3S2	Pharmaceutical (Antiglaucoma, Carbonic anhydrase inhibitor)
Bimatoprost	155206-00-1	C25H37NO4	Pharmaceutical (Antiglaucoma, Eyelash growth stimulant)
Pilocarpine	92-13-7	C11H16N2O2	Pharmaceutical (Antiglaucoma, Muscarinic acetylcholine receptor agonist)
Latanoprost	130209-82-4	C26H40O5	Pharmaceutical (Antiglaucoma, Prostaglandin F receptor agonist)
Latanoprostene Bunod	860005-21-6	C27H41NO8	Pharmaceutical (Antiglaucoma, Prostaglandin F receptor agonist)
Tafluprost	209860-87-7	C25H34F2O5	Pharmaceutical (Antiglaucoma, Prostaglandin F receptor agonist)
Travoprost	157283-68-6	C26H35F3O6	Pharmaceutical (Antiglaucoma, Prostaglandin F receptor agonist)
Netarsudil	1254032-66-0	C28H27N3O3	Pharmaceutical (Antiglaucoma, Rho-associated kinase inhibitor)
Cinacalcet	226256-56-0	C22H22F3N	Pharmaceutical (Antihypercalcemia, Antihyperparathyroidism, Calcium sensing receptor allosteric modulator)
Zoledronic Acid	118072-93-8	C5H10N2O7P2	Pharmaceutical (Antihypercalcemia, Antiresorptive, Farnesylpyrophosphate synthetase inhibitor)
Ezetimibe	163222-33-1	C24H21F2NO3	Pharmaceutical (Antihyperlipidemic, Cholesterol absorption inhibitor)
Atorvastatin	134523-00-5	C33H35FN2O5	Pharmaceutical (Antihyperlipidemic, HMG-CoA reductase inhibitor)
Fluvastatin	93957-54-1	C24H26FNO4	Pharmaceutical (Antihyperlipidemic, HMG-CoA reductase inhibitor)
Lovastatin	75330-75-5	C24H36O5	Pharmaceutical (Antihyperlipidemic, HMG-CoA reductase inhibitor)
Pitavastatin	147511-69-1	C25H24FNO4	Pharmaceutical (Antihyperlipidemic, HMG-CoA reductase inhibitor)
Pravastatin	81093-37-0	C23H36O7	Pharmaceutical (Antihyperlipidemic, HMG-CoA reductase inhibitor)
Rosuvastatin	287714-41-4	C22H28FN3O6S	Pharmaceutical (Antihyperlipidemic, HMG-CoA reductase inhibitor)
Simvastatin	79902-63-9	C25H38O5	Pharmaceutical (Antihyperlipidemic, HMG-CoA reductase inhibitor)
Lomitapide	182431-12-5	C39H37F6N3O2	Pharmaceutical (Antihyperlipidemic, Microsomal triglyceride transfer protein (MTTP) inhibitor)
Bezafibrate	41859-67-0	C19H20ClNO4	Pharmaceutical (Antihyperlipidemic, Triglyceride synthesis inhibitor, Peroxisome proliferator-activated receptor (PPAR) alpha agonist)
Clofibrate	637-07-0	C12H15ClO3	Pharmaceutical (Antihyperlipidemic, Triglyceride synthesis inhibitor, Peroxisome proliferator-activated receptor (PPAR) alpha agonist)
Fenofibrate	49562-28-9	C20H21ClO4	Pharmaceutical (Antihyperlipidemic, Triglyceride synthesis inhibitor, Peroxisome proliferator-activated receptor (PPAR) alpha agonist)
Gemfibrozil	25812-30-0	C15H22O3	Pharmaceutical (Antihyperlipidemic, Triglyceride synthesis inhibitor, Peroxisome proliferator-activated receptor (PPAR) alpha agonist)
Fenofibric Acid	42017-89-0	C17H15ClO4	Pharmaceutical (Antihyperlipidemic, Triglyceride synthesis inhibitor, Peroxisome proliferator-activated receptor (PPAR) alpha agonist) /
Paricalcitol	131918-61-1	C27H44O3	Pharmaceutical (Antihyperparathyroidism, Vitamin D receptor agonist)
Sapropterin	62989-33-7	C9H15N5O3	Pharmaceutical (Antihyperphenylalaninemia)
Debrisoquine	1131-64-2	C10H13N3	Pharmaceutical (Antihypertensive)
Mecamylamine	60-40-2	C11H21N	Pharmaceutical (Antihypertensive)
Methyldopate	2544-09-4	C12H17NO4	Pharmaceutical (Antihypertensive)
Eplerenone	107724-20-9	C24H30O6	Pharmaceutical (Antihypertensive, Aldosterone antagonist)
Carvedilol	72956-09-3	C24H26N2O4	Pharmaceutical (Antihypertensive, alpha1/beta-Adrenergic receptor antagonist)
Labetalol	36894-69-6	C19H24N2O3	Pharmaceutical (Antihypertensive, alpha1/beta-Adrenergic receptor antagonist)
Doxazosin	74191-85-8	C23H25N5O5	Pharmaceutical (Antihypertensive, alpha1-Adrenergic receptor antagonist)
Clonidine	4205-90-7	C9H9Cl2N3	Pharmaceutical (Antihypertensive, alpha2-Adrenergic receptor agonist)
Guanabenz	5051-62-7	C8H8Cl2N4	Pharmaceutical (Antihypertensive, alpha2-Adrenergic receptor agonist)
Guanfacine	29110-47-2	C9H9Cl2N3O	Pharmaceutical (Antihypertensive, alpha2-Adrenergic receptor agonist)
Methyldopa	555-30-6	C10H13NO4	Pharmaceutical (Antihypertensive, alpha2-Adrenergic receptor agonist)
Azilsartan	147403-03-0	C25H20N4O5	Pharmaceutical (Antihypertensive, Angiotensin II receptor antagonist)
Candesartan	139481-59-7	C24H20N6O3	Pharmaceutical (Antihypertensive, Angiotensin II receptor antagonist)
Eprosartan	133040-01-4	C23H24N2O4S	Pharmaceutical (Antihypertensive, Angiotensin II receptor antagonist)
Irbesartan	138402-11-6	C25H28N6O	Pharmaceutical (Antihypertensive, Angiotensin II receptor antagonist)
Losartan	114798-26-4	C22H23ClN6O	Pharmaceutical (Antihypertensive, Angiotensin II receptor antagonist)
Olmesartan	144689-24-7	C24H26N6O3	Pharmaceutical (Antihypertensive, Angiotensin II receptor antagonist)

Table S20. Compound database for suspect screening (continued)

Compound Name	CAS	Molecular Formula	Category
Telmisartan	144701-48-4	C33H30N4O2	Pharmaceutical (Antihypertensive, Angiotensin II receptor antagonist)
Valsartan	137862-53-4	C24H29N5O3	Pharmaceutical (Antihypertensive, Angiotensin II receptor antagonist)
Benazepril	86541-75-5	C24H28N2O5	Pharmaceutical (Antihypertensive, Angiotensin-converting enzyme inhibitor)
Captopril	62571-86-2	C9H15NO3S	Pharmaceutical (Antihypertensive, Angiotensin-converting enzyme inhibitor)
Cilazapril	88768-40-5	C22H31N3O5	Pharmaceutical (Antihypertensive, Angiotensin-converting enzyme inhibitor)
Enalapril	75847-73-3	C20H28N2O5	Pharmaceutical (Antihypertensive, Angiotensin-converting enzyme inhibitor)
Fosinopril	98048-97-6	C30H46NO7P	Pharmaceutical (Antihypertensive, Angiotensin-converting enzyme inhibitor)
Imidapril	89371-37-9	C20H27N3O6	Pharmaceutical (Antihypertensive, Angiotensin-converting enzyme inhibitor)
Lisinopril	76547-98-3	C21H31N3O5	Pharmaceutical (Antihypertensive, Angiotensin-converting enzyme inhibitor)
Moexipril	103775-10-6	C27H34N2O7	Pharmaceutical (Antihypertensive, Angiotensin-converting enzyme inhibitor)
Perindopril	82834-16-0	C19H32N2O5	Pharmaceutical (Antihypertensive, Angiotensin-converting enzyme inhibitor)
Quinapril	85441-61-8	C25H30N2O5	Pharmaceutical (Antihypertensive, Angiotensin-converting enzyme inhibitor)
Ramipril	87333-19-5	C23H32N2O5	Pharmaceutical (Antihypertensive, Angiotensin-converting enzyme inhibitor)
Trandolapril	87679-37-6	C24H34N2O5	Pharmaceutical (Antihypertensive, Angiotensin-converting enzyme inhibitor)
Reserpine	50-55-5	C33H40N2O9	Pharmaceutical (Antihypertensive, Antipsychotic, Vesicular monoamine transporter inhibitor)
Bisoprolol	66722-44-9	C18H31NO4	Pharmaceutical (Antihypertensive, beta1-Adrenergic receptor antagonist)
Clevidipine	167221-71-8	C21H23Cl2NO6	Pharmaceutical (Antihypertensive, Calcium channel blocker)
Isradipine	75695-93-1	C19H21N3O5	Pharmaceutical (Antihypertensive, Calcium channel blocker)
Spironolactone	52-01-7	C24H32O4S	Pharmaceutical (Antihypertensive, Diuretic, Aldosterone antagonist)
Fenoldopam	67227-56-9	C16H16ClNO3	Pharmaceutical (Antihypertensive, Diuretic, Dopamine D1 receptor agonist)
Bendroflumethiazide	73-48-3	C15H14F3N3O4S2	Pharmaceutical (Antihypertensive, Diuretic, Na+-Cl- symport inhibitor)
Chlorthalidone	77-36-1	C14H11ClN2O4S	Pharmaceutical (Antihypertensive, Diuretic, Na+-Cl- symport inhibitor)
Hydrochlorothiazide	58-93-5	C7H8ClN3O4S2	Pharmaceutical (Antihypertensive, Diuretic, Na+-Cl- symport inhibitor)
Indapamide	26807-65-8	C16H16ClN3O3S	Pharmaceutical (Antihypertensive, Diuretic, Na+-Cl- symport inhibitor)
Methyclothiazide	135-07-9	C9H11Cl2N3O4S2	Pharmaceutical (Antihypertensive, Diuretic, Na+-Cl- symport inhibitor)
Metolazone	17560-51-9	C16H16ClN3O3S	Pharmaceutical (Antihypertensive, Diuretic, Na+-Cl- symport inhibitor)
Trichlormethiazide	133-67-5	C8H8Cl3N3O4S2	Pharmaceutical (Antihypertensive, Diuretic, Na+-Cl- symport inhibitor)
Chlorothiazide	58-94-6	C7H6ClN3O4S2	Pharmaceutical (Antihypertensive, Diuretic, Na+-Cl- symport inhibitor) / Pharmaceutical TP (Hydrochlorothiazide)
Bosentan	147536-97-8	C27H29N5O6S	Pharmaceutical (Antihypertensive, Endothelin receptor antagonist)
Macitentan	441798-33-0	C19H20Br2N6O4S	Pharmaceutical (Antihypertensive, Endothelin receptor antagonist)
Ambrisentan	177036-94-1	C22H22N2O4	Pharmaceutical (Antihypertensive, Endothelin receptor type A antagonist)
Minoxidil	38304-91-5	C9H15N5O	Pharmaceutical (Antihypertensive, Hair growth stimulant (topical), ATP-sensitive potassium channel opener)
Tadalafil	171596-29-5	C22H19N3O4	Pharmaceutical (Antihypertensive, Impotence therapy, Phosphodiesterase V inhibitor)
Sacubitril	149709-62-6	C24H29NO5	Pharmaceutical (Antihypertensive, Neprilysin inhibitor)
Iloprost	78919-13-8	C22H32O4	Pharmaceutical (Antihypertensive, Platelet aggregation inhibitor, Prostaglandin I2 receptor agonist)
Aliskiren	173334-57-1	C30H53N3O6	Pharmaceutical (Antihypertensive, Renin inhibitor)
Metyrosine	672-87-7	C10H13NO3	Pharmaceutical (Antihypertensive, Tyrosine monooxygenase inhibitor)
Levonordefrin	829-74-3	C9H13NO3	Pharmaceutical (Antihypertensive, Vasoconstrictor, alpha2-Adrenergic receptor agonist)
Nitroglycerin	55-63-0	C3H5N3O9	Pharmaceutical (Antihypertensive, Vasodilator (coronary))
Hydralazine	86-54-4	C8H8N4	Pharmaceutical (Antihypertensive, Vasodilator (peripheric))
Prazosin	19216-56-9	C19H21N5O4	Pharmaceutical (Antihypertensive, Vasodilator (peripheric), alpha1-Adrenergic receptor antagonist)
Phenoxybenzamine	59-96-1	C18H22ClNO	Pharmaceutical (Antihypertensive, Vasodilator (peripheric), alpha-Adrenergic receptor antagonist)
Celiprolol	56980-93-9	C20H33N3O4	Pharmaceutical (Antihypertensive, Vasodilator, beta1-Adrenergic receptor antagonist)
Metoprolol	51384-51-1	C15H25NO3	Pharmaceutical (Antihypertensive, Vasodilator, beta1-Adrenergic receptor antagonist)
Nebivolol	99200-09-6	C22H25F2NO4	Pharmaceutical (Antihypertensive, Vasodilator, beta1-Adrenergic receptor antagonist)
Amlodipine	88150-42-9	C20H25ClN2O5	Pharmaceutical (Antihypertensive, Vasodilator, Calcium channel blocker)
Felodipine	72509-76-3	C18H19Cl2NO4	Pharmaceutical (Antihypertensive, Vasodilator, Calcium channel blocker)
Nicardipine	55985-32-5	C26H29N3O6	Pharmaceutical (Antihypertensive, Vasodilator, Calcium channel blocker)
Nifedipine	21829-25-4	C17H18N2O6	Pharmaceutical (Antihypertensive, Vasodilator, Calcium channel blocker)
Nimodipine	66085-59-4	C21H26N2O7	Pharmaceutical (Antihypertensive, Vasodilator, Calcium channel blocker)
Nisoldipine	63675-72-9	C20H24N2O6	Pharmaceutical (Antihypertensive, Vasodilator, Calcium channel blocker)
Selexipag	475086-01-2	C26H32N4O4S	Pharmaceutical (Antihypertensive, Vasodilator, Prostaglandin I2 receptor agonist)
Diazoxide	364-98-7	C8H7ClN2O2S	Pharmaceutical (Antihypoglycemia, Antihypertensive, ATP-sensitive potassium channel opener)
Midodrine	42794-76-3	C12H18N2O4	Pharmaceutical (Antihypotensive, alpha1-Adrenergic receptor agonist)
Metaraminol	54-49-9	C9H13NO2	Pharmaceutical (Antihypotensive, alpha-Adrenergic receptor agonist)
Mephentermine	100-92-5	C11H17N	Pharmaceutical (Antihypotensive, Vasoconstrictor)
Norepinephrine	51-41-2	C8H11NO3	Pharmaceutical (Antihypotensive, Vasoconstrictor, Adrenergic receptor agonist)
Furaltadone	139-91-3	C13H16N4O6	Pharmaceutical (Anti-infective (urinary))
Balsalazide	80573-04-2	C17H15N3O6	Pharmaceutical (Anti-inflammatory (gastrointestinal))
Olsalazine	15722-48-2	C14H10N2O6	Pharmaceutical (Anti-inflammatory (gastrointestinal))
Loteprednol	129260-79-3	C21H27ClO5	Pharmaceutical (Anti-inflammatory (ophthalmic), Glucocorticoid receptor agonist)
Halcinonide	3093-35-4	C24H32ClFO5	Pharmaceutical (Anti-inflammatory (topical), Glucocorticoid receptor agonist)
Vedaprofen	71109-09-6	C19H22O2	Pharmaceutical (Anti-inflammatory (veterinary))
Aminopyrine	58-15-1	C13H17N3O	Pharmaceutical (Anti-inflammatory)
Carprofen	53716-49-7	C15H12ClNO2	Pharmaceutical (Anti-inflammatory)
Isoflupredone	338-95-4	C21H27FO5	Pharmaceutical (Anti-inflammatory)
Mesalazine (Mesalamine)	89-57-6	C7H7NO3	Pharmaceutical (Anti-inflammatory)

Table S20. Compound database for suspect screening (continued)

Compound Name	CAS	Molecular Formula	Category
Dexamethasone	50-02-2	C22H29FO5	Pharmaceutical (Anti-inflammatory, Antipruritic, Glucocorticoid receptor agonist)
Flurandrenolide	1524-88-5	C24H33FO6	Pharmaceutical (Anti-inflammatory, Antipruritic, Glucocorticoid receptor agonist)
Sulfasalazine	599-79-1	C18H14N4O5S	Pharmaceutical (Anti-inflammatory, Antirheumatic)
Betamethasone	378-44-9	C22H29FO5	Pharmaceutical (Anti-inflammatory, Antirheumatic, Glucocorticoid receptor agonist)
Prednisolone	50-24-8	C21H28O5	Pharmaceutical (Anti-inflammatory, Antirheumatic, Glucocorticoid receptor agonist)
Grapiprant	415903-37-6	C26H29N5O3S	Pharmaceutical (Anti-inflammatory, Antirheumatic, Prostaglandin receptor antagonist)
Aceclofenac	89796-99-6	C16H13Cl2NO4	Pharmaceutical (Anti-inflammatory, COX inhibitor)
Flufenamic Acid	530-78-9	C14H10F3NO2	Pharmaceutical (Anti-inflammatory, COX inhibitor)
Meclofenamic Acid	644-62-2	C14H11Cl2NO2	Pharmaceutical (Anti-inflammatory, COX inhibitor)
Tolmetin	26171-23-3	C15H15NO3	Pharmaceutical (Anti-inflammatory, COX inhibitor)
Alclometasone	67452-97-5	C22H29ClO5	Pharmaceutical (Anti-inflammatory, Glucocorticoid receptor agonist)
Amcinonide	51022-69-6	C28H35FO7	Pharmaceutical (Anti-inflammatory, Glucocorticoid receptor agonist)
Clobetasol	25122-41-2	C22H28ClFO4	Pharmaceutical (Anti-inflammatory, Glucocorticoid receptor agonist)
Clocortolone	4828-27-7	C22H28ClFO4	Pharmaceutical (Anti-inflammatory, Glucocorticoid receptor agonist)
Cortisone	53-06-5	C21H28O5	Pharmaceutical (Anti-inflammatory, Glucocorticoid receptor agonist)
Deflazacort	14484-47-0	C25H31NO6	Pharmaceutical (Anti-inflammatory, Glucocorticoid receptor agonist)
Desonide	638-94-8	C24H32O6	Pharmaceutical (Anti-inflammatory, Glucocorticoid receptor agonist)
Desoximetasone	382-67-2	C22H29FO4	Pharmaceutical (Anti-inflammatory, Glucocorticoid receptor agonist)
Diflorasone	2557-49-5	C22H28F2O5	Pharmaceutical (Anti-inflammatory, Glucocorticoid receptor agonist)
Difluprednate	23674-86-4	C27H34F2O7	Pharmaceutical (Anti-inflammatory, Glucocorticoid receptor agonist)
Flumethasone	2135-17-3	C22H28F2O5	Pharmaceutical (Anti-inflammatory, Glucocorticoid receptor agonist)
Flunisolide	3385-03-3	C24H31FO6	Pharmaceutical (Anti-inflammatory, Glucocorticoid receptor agonist)
Fluocinolone	807-38-5	C21H26F2O6	Pharmaceutical (Anti-inflammatory, Glucocorticoid receptor agonist)
Fluocinonide	356-12-7	C26H32F2O7	Pharmaceutical (Anti-inflammatory, Glucocorticoid receptor agonist)
Fluorometholone	426-13-1	C22H29FO4	Pharmaceutical (Anti-inflammatory, Glucocorticoid receptor agonist)
Fluticasone	90566-53-3	C22H27F3O4S	Pharmaceutical (Anti-inflammatory, Glucocorticoid receptor agonist)
Hydrocortisone	50-23-7	C21H30O5	Pharmaceutical (Anti-inflammatory, Glucocorticoid receptor agonist)
Mometasone	105102-22-5	C22H28Cl2O4	Pharmaceutical (Anti-inflammatory, Glucocorticoid receptor agonist)
Prednicarbate	73771-04-7	C27H36O8	Pharmaceutical (Anti-inflammatory, Glucocorticoid receptor agonist)
Prednisone	53-03-2	C21H26O5	Pharmaceutical (Anti-inflammatory, Glucocorticoid receptor agonist)
Ulobetasol	98651-66-2	C22H27ClF2O4	Pharmaceutical (Anti-inflammatory, Glucocorticoid receptor agonist)
Teriflunomide	163451-81-8	C12H9F3N2O2	Pharmaceutical (Anti-inflammatory, Immunomodulator, Dihydroorotate dehydrogenase inhibitor) / Pharmaceutical TP (Leflunomide)
Lifitegrast	1025967-78-5	C29H24Cl2N2O7S	Pharmaceutical (Anti-inflammatory, Lymphocyte function-associated antigen-1 antagonist)
Apremilast	608141-41-9	C22H24N2O7S	Pharmaceutical (Anti-inflammatory, Phosphodiesterase IV inhibitor)
Amodiaquine	86-42-0	C20H22ClN3O	Pharmaceutical (Antimalarial)
Artemether	71963-77-4	C16H26O5	Pharmaceutical (Antimalarial)
Artemisinin	63968-64-9	C15H22O5	Pharmaceutical (Antimalarial)
Hydroxychloroquine	118-42-3	C18H26ClN3O	Pharmaceutical (Antimalarial)
Mefloquine	53230-10-7	C17H16F6N2O	Pharmaceutical (Antimalarial)
Primaquine	90-34-6	C15H21N3O	Pharmaceutical (Antimalarial)
Proguanil	500-92-5	C11H16ClN5	Pharmaceutical (Antimalarial)
Pyrimethamine	58-14-0	C12H13ClN4	Pharmaceutical (Antimalarial)
Quinine	130-95-0	C20H24N2O2	Pharmaceutical (Antimalarial)
Chloroquine	54-05-7	C18H26ClN3	Pharmaceutical (Antimalarial, Amebicide)
Almotriptan	154323-57-6	C17H25N3O2S	Pharmaceutical (Antimigraine, Serotonin receptor agonist)
Frovatriptan	158747-02-5	C14H17N3O	Pharmaceutical (Antimigraine, Serotonin receptor agonist)
Ergotamine	113-15-5	C33H35N5O5	Pharmaceutical (Antimigraine, Vasoconstrictor, alpha1-Adrenergic receptor antagonist, Serotonin receptor antagonist)
Dihydroergotamine	511-12-6	C33H37N5O5	Pharmaceutical (Antimigraine, Vasoconstrictor, Serotonin receptor agonist)
Eletriptan	143322-58-1	C22H26N2O2S	Pharmaceutical (Antimigraine, Vasoconstrictor, Serotonin receptor agonist)
Naratriptan	121679-13-8	C17H25N3O2S	Pharmaceutical (Antimigraine, Vasoconstrictor, Serotonin receptor agonist)
Rizatriptan	144034-80-0	C15H19N5	Pharmaceutical (Antimigraine, Vasoconstrictor, Serotonin receptor agonist)
Sumatriptan	103628-46-2	C14H21N3O2S	Pharmaceutical (Antimigraine, Vasoconstrictor, Serotonin receptor agonist)
Zolmitriptan	139264-17-8	C16H21N3O2	Pharmaceutical (Antimigraine, Vasoconstrictor, Serotonin receptor agonist)
Masitinib	790299-79-5	C28H30N6OS	Pharmaceutical (Antineoplastic (veterinary))
Rabacfosadine	859209-74-8	C21H35N8O6P	Pharmaceutical (Antineoplastic (veterinary))
Toceranib	356068-94-5	C22H25FN4O2	Pharmaceutical (Antineoplastic (veterinary), Angiogenesis inhibitor, Receptor tyrosine kinase inhibitor)
Bexarotene	153559-49-0	C24H28O2	Pharmaceutical (Antineoplastic)
Calusterone	17021-26-0	C21H32O2	Pharmaceutical (Antineoplastic)
Enasidenib	1446502-11-9	C19H17F6N7O	Pharmaceutical (Antineoplastic)
Ingenol	30220-46-3	C20H28O5	Pharmaceutical (Antineoplastic)
Mitomycin	50-07-7	C15H18N4O5	Pharmaceutical (Antineoplastic)
Procarbazine	671-16-9	C12H19N3O	Pharmaceutical (Antineoplastic)
Testolactone	968-93-4	C19H24O3	Pharmaceutical (Antineoplastic)
Thalidomide	50-35-1	C13H10N2O4	Pharmaceutical (Antineoplastic)
Altretamine	645-05-6	C9H18N6	Pharmaceutical (Antineoplastic, Alkylating agent)
Bendamustine	16506-27-7	C16H21Cl2N3O2	Pharmaceutical (Antineoplastic, Alkylating agent)
Carmustine	154-93-8	C5H9Cl2N3O2	Pharmaceutical (Antineoplastic, Alkylating agent)

Table S20. Compound database for suspect screening (continued)

Compound Name	CAS	Molecular Formula	Category
Chlorambucil	305-03-3	C14H19Cl2NO2	Pharmaceutical (Antineoplastic, Alkylating agent)
Dacarbazine	4342-03-4	C6H10N6O	Pharmaceutical (Antineoplastic, Alkylating agent)
Estramustine	2998-57-4	C23H31Cl2NO3	Pharmaceutical (Antineoplastic, Alkylating agent)
Ifosfamide	3778-73-2	C7H15Cl2N2O2P	Pharmaceutical (Antineoplastic, Alkylating agent)
Lomustine	13010-47-4	C9H16ClN3O2	Pharmaceutical (Antineoplastic, Alkylating agent)
Mechlorethamine	51-75-2	C5H11Cl2N	Pharmaceutical (Antineoplastic, Alkylating agent)
Melphalan	148-82-3	C13H18Cl2N2O2	Pharmaceutical (Antineoplastic, Alkylating agent)
Streptozocin	18883-66-4	C8H15N3O7	Pharmaceutical (Antineoplastic, Alkylating agent)
Temozolomide	85622-93-1	C6H6N6O2	Pharmaceutical (Antineoplastic, Alkylating agent)
Thiotepa	52-24-4	C6H12N3PS	Pharmaceutical (Antineoplastic, Alkylating agent)
Trabectedin	114899-77-3	C39H43N3O11S	Pharmaceutical (Antineoplastic, Alkylating agent)
Busulfan	55-98-1	C6H14O6S2	Pharmaceutical (Antineoplastic, Alkylating agent, Immunosuppressant)
Cyclophosphamide	50-18-0	C7H15Cl2N2O2P	Pharmaceutical (Antineoplastic, Alkylating agent, Immunosuppressant)
Alectinib	1256580-46-7	C30H34N4O2	Pharmaceutical (Antineoplastic, Anaplastic lymphoma kinase (ALK) inhibitor)
Brigatinib	1197953-54-0	C29H39ClN7O2P	Pharmaceutical (Antineoplastic, Anaplastic lymphoma kinase (ALK) inhibitor)
Bicalutamide	90357-06-5	C18H14F4N2O4S	Pharmaceutical (Antineoplastic, Androgen receptor antagonist)
Enzalutamide	915087-33-1	C21H16F4N4O2S	Pharmaceutical (Antineoplastic, Androgen receptor antagonist)
Flutamide	13311-84-7	C11H11F3N2O3	Pharmaceutical (Antineoplastic, Androgen receptor antagonist)
Nilutamide	63612-50-0	C12H10F3N3O4	Pharmaceutical (Antineoplastic, Androgen receptor antagonist)
Abiraterone	154229-19-3	C24H31NO	Pharmaceutical (Antineoplastic, Androgen synthesis inhibitor)
Sunitinib	341031-54-7	C22H27FN4O2	Pharmaceutical (Antineoplastic, Angiogenesis inhibitor, Receptor tyrosine kinase inhibitor)
Capecitabine	154361-50-9	C15H22FN3O6	Pharmaceutical (Antineoplastic, Antimetabolite)
Cladribine	4291-63-8	C10H12ClN5O3	Pharmaceutical (Antineoplastic, Antimetabolite)
Clofarabine	123318-82-1	C10H11ClFN5O3	Pharmaceutical (Antineoplastic, Antimetabolite)
Decitabine	2353-33-5	C8H12N4O4	Pharmaceutical (Antineoplastic, Antimetabolite)
Fludarabine	21679-14-1	C10H12FN5O4	Pharmaceutical (Antineoplastic, Antimetabolite)
Fluorouracil	51-21-8	C4H3FN2O2	Pharmaceutical (Antineoplastic, Antimetabolite)
Gemcitabine	95058-81-4	C9H11F2N3O4	Pharmaceutical (Antineoplastic, Antimetabolite)
Mercaptopurine	50-44-2	C5H4N4S	Pharmaceutical (Antineoplastic, Antimetabolite)
Methotrexate	59-05-2	C20H22N8O5	Pharmaceutical (Antineoplastic, Antimetabolite)
Nelarabine	121032-29-9	C11H15N5O5	Pharmaceutical (Antineoplastic, Antimetabolite)
Pemetrexed	137281-23-3	C20H21N5O6	Pharmaceutical (Antineoplastic, Antimetabolite)
Pralatrexate	146464-95-1	C23H23N7O5	Pharmaceutical (Antineoplastic, Antimetabolite)
Tioguanine	154-42-7	C5H5N5S	Pharmaceutical (Antineoplastic, Antimetabolite)
Tipiracil	183204-74-2	C9H11ClN4O2	Pharmaceutical (Antineoplastic, Antimetabolite)
Ixabepilone	219989-84-1	C27H42N2O5S	Pharmaceutical (Antineoplastic, Antimitotic)
Eflornithine	70052-12-9	C6H12F2N2O2	Pharmaceutical (Antineoplastic, Antiprotozoal, Ornithine decarboxylase inhibitor)
Floxuridine	50-91-9	C9H11FN2O5	Pharmaceutical (Antineoplastic, Antiviral, Antimetabolite)
Podofilox	518-28-5	C22H22O8	Pharmaceutical (Antineoplastic, Antiviral, Tubulin polymerization inhibitor)
Venetoclax	1257044-40-8	C45H50ClN7O7S	Pharmaceutical (Antineoplastic, Bcl-2 inhibitor)
Dabrafenib	1195765-45-7	C23H20F3N5O2S2	Pharmaceutical (Antineoplastic, BRAF kinase inhibitor)
Vemurafenib	918504-65-1	C23H18ClF2N3O3S	Pharmaceutical (Antineoplastic, BRAF kinase inhibitor)
Acalabrutinib	1420477-60-6	C26H23N7O2	Pharmaceutical (Antineoplastic, Bruton's tyrosine kinase inhibitor)
Ibrutinib	936563-96-1	C25H24N6O2	Pharmaceutical (Antineoplastic, Bruton's tyrosine kinase inhibitor)
Plerixafor	110078-46-1	C28H54N8	Pharmaceutical (Antineoplastic, Chemokine receptor type 4 (CXCR4) antagonist)
Medroxyprogesterone	520-85-4	C22H32O3	Pharmaceutical (Antineoplastic, Contraceptive, Progesterone receptor agonist)
Megestrol	3562-63-8	C22H30O3	Pharmaceutical (Antineoplastic, Contraceptive, Progesterone receptor agonist)
Abemaciclib	1231929-97-7	C27H32F2N8	Pharmaceutical (Antineoplastic, Cyclin-dependent kinase (CDK) inhibitor)
Palbociclib	571190-30-2	C24H29N7O2	Pharmaceutical (Antineoplastic, Cyclin-dependent kinase (CDK) inhibitor)
Ribociclib	1211441-98-3	C23H30N8O	Pharmaceutical (Antineoplastic, Cyclin-dependent kinase (CDK) inhibitor)
Anastrozole	120511-73-1	C17H19N5	Pharmaceutical (Antineoplastic, Estrogen biosynthesis inhibitor)
Exemestane	107868-30-4	C20H24O2	Pharmaceutical (Antineoplastic, Estrogen biosynthesis inhibitor)
Fadrozole	102676-47-1	C14H13N3	Pharmaceutical (Antineoplastic, Estrogen biosynthesis inhibitor)
Letrozole	112809-51-5	C17H11N5	Pharmaceutical (Antineoplastic, Estrogen biosynthesis inhibitor)
Tamoxifen	10540-29-1	C26H29NO	Pharmaceutical (Antineoplastic, Estrogen receptor agonist/antagonist)
Toremifene	89778-26-7	C26H28ClNO	Pharmaceutical (Antineoplastic, Estrogen receptor agonist/antagonist)
Belinostat	414864-00-9	C15H14N2O4S	Pharmaceutical (Antineoplastic, Histone deacetylase inhibitor)
Panobinostat	404950-80-7	C21H23N3O2	Pharmaceutical (Antineoplastic, Histone deacetylase inhibitor)
Romidepsin	128517-07-7	C24H36N4O6S2	Pharmaceutical (Antineoplastic, Histone deacetylase inhibitor)
Vorinostat	149647-78-9	C14H20N2O3	Pharmaceutical (Antineoplastic, Histone deacetylase inhibitor)
Pomalidomide	19171-19-8	C13H11N3O4	Pharmaceutical (Antineoplastic, Immunomodulator)
Lenalidomide	191732-72-6	C13H13N3O3	Pharmaceutical (Antineoplastic, Immunomodulator, TNF-alpha inhibitor)
Everolimus	159351-69-6	C53H83NO14	Pharmaceutical (Antineoplastic, Immunosuppressant, mTOR inhibitor)
Sirolimus	53123-88-9	C51H79NO13	Pharmaceutical (Antineoplastic, Immunosuppressant, mTOR inhibitor)
Ruxolitinib	941678-49-5	C17H18N6	Pharmaceutical (Antineoplastic, Janus kinase (JAK) inhibitor)
Tretinoin (Retinoic Acid)	302-79-4	C20H28O2	Pharmaceutical (Antineoplastic, Keratolytic, Retinoic acid receptor (RAR) agonist)
Ethinyl Estradiol	57-63-6	C20H24O2	Pharmaceutical (Antineoplastic, Menstruation disorder agent, Estrogen receptor agonist)

Table S20. Compound database for suspect screening (continued)

Compound Name	CAS	Molecular Formula	Category
Cobimetinib	934660-93-2	C21H21F3IN3O2	Pharmaceutical (Antineoplastic, Mitogen-activated extracellular signal-regulated kinase (MEK) inhibitor)
Trametinib	871700-17-3	C26H23FIN5O4	Pharmaceutical (Antineoplastic, Mitogen-activated extracellular signal-regulated kinase (MEK) inhibitor)
Niraparib	1038915-60-4	C19H20N4O	Pharmaceutical (Antineoplastic, PARP inhibitor)
Olaparib	763113-22-0	C24H23FN4O3	Pharmaceutical (Antineoplastic, PARP inhibitor)
Rucaparib	283173-50-2	C19H18FN3O	Pharmaceutical (Antineoplastic, PARP inhibitor)
Copanlisib	1032568-63-0	C23H28N8O4	Pharmaceutical (Antineoplastic, Phosphatidylinositol 3-kinase inhibitor)
Idelalisib	870281-82-6	C22H18FN7O	Pharmaceutical (Antineoplastic, Phosphatidylinositol 3-kinase inhibitor)
Verteporfin	129497-78-5	C41H42N4O8	Pharmaceutical (Antineoplastic, Photosensitizer)
Pentostatin	53910-25-1	C11H16N4O4	Pharmaceutical (Antineoplastic, Potentiator)
Carfilzomib	868540-17-4	C40H57N5O7	Pharmaceutical (Antineoplastic, Proteasome inhibitor)
Omacetaxine Mepesuccinate	26833-87-4	C29H39NO9	Pharmaceutical (Antineoplastic, Protein biosynthesis inhibitor)
Afatinib	850140-72-6	C24H25ClFN5O3	Pharmaceutical (Antineoplastic, Receptor tyrosine kinase inhibitor)
Axitinib	319460-85-0	C22H18N4OS	Pharmaceutical (Antineoplastic, Receptor tyrosine kinase inhibitor)
Cabozantinib	849217-68-1	C28H24FN3O5	Pharmaceutical (Antineoplastic, Receptor tyrosine kinase inhibitor)
Erlotinib	183321-74-6	C22H23N3O4	Pharmaceutical (Antineoplastic, Receptor tyrosine kinase inhibitor)
Gefitinib	184475-35-2	C22H24ClFN4O3	Pharmaceutical (Antineoplastic, Receptor tyrosine kinase inhibitor)
Lapatinib	231277-92-2	C29H26ClFN4O4S	Pharmaceutical (Antineoplastic, Receptor tyrosine kinase inhibitor)
Lenvatinib	417716-92-8	C21H19ClN4O4	Pharmaceutical (Antineoplastic, Receptor tyrosine kinase inhibitor)
Midostaurin	120685-11-2	C35H30N4O4	Pharmaceutical (Antineoplastic, Receptor tyrosine kinase inhibitor)
Neratinib	698387-09-6	C30H29ClN6O3	Pharmaceutical (Antineoplastic, Receptor tyrosine kinase inhibitor)
Osimertinib	1421373-65-0	C28H33N7O2	Pharmaceutical (Antineoplastic, Receptor tyrosine kinase inhibitor)
Pazopanib	444731-52-6	C21H23N7O2S	Pharmaceutical (Antineoplastic, Receptor tyrosine kinase inhibitor)
Regorafenib	755037-03-7	C21H15ClF4N4O3	Pharmaceutical (Antineoplastic, Receptor tyrosine kinase inhibitor)
Vandetanib	443913-73-3	C22H24BrFN4O2	Pharmaceutical (Antineoplastic, Receptor tyrosine kinase inhibitor)
Fluoxymesterone	76-43-7	C20H29FO3	Pharmaceutical (Antineoplastic, Replenisher (androgen), Androgen receptor agonist)
Sonidegib	956697-53-3	C26H26F3N3O3	Pharmaceutical (Antineoplastic, Smoothened receptor antagonist)
Vismodegib	879085-55-9	C19H14Cl2N2O3S	Pharmaceutical (Antineoplastic, Smoothened receptor antagonist)
Irinotecan	97682-44-5	C33H38N4O6	Pharmaceutical (Antineoplastic, Topoisomerase I inhibitor)
Topotecan	123948-87-8	C23H23N3O5	Pharmaceutical (Antineoplastic, Topoisomerase I inhibitor)
Doxorubicin	23214-92-8	C27H29NO11	Pharmaceutical (Antineoplastic, Topoisomerase II inhibitor)
Etoposide	33419-42-0	C29H32O13	Pharmaceutical (Antineoplastic, Topoisomerase II inhibitor)
Mitoxantrone	65271-80-9	C22H28N4O6	Pharmaceutical (Antineoplastic, Topoisomerase II inhibitor)
Valrubicin	56124-62-0	C34H36F3NO13	Pharmaceutical (Antineoplastic, Topoisomerase II inhibitor)
Cabazitaxel	183133-96-2	C45H57NO14	Pharmaceutical (Antineoplastic, Tubulin depolymerization inhibitor)
Docetaxel	114977-28-5	C43H53NO14	Pharmaceutical (Antineoplastic, Tubulin depolymerization inhibitor)
Paclitaxel	33069-62-4	C47H51NO14	Pharmaceutical (Antineoplastic, Tubulin depolymerization inhibitor)
Eribulin	253128-41-5	C40H59NO11	Pharmaceutical (Antineoplastic, Tubulin polymerization inhibitor)
Vinblastine	865-21-4	C46H58N4O9	Pharmaceutical (Antineoplastic, Tubulin polymerization inhibitor)
Vincristine	57-22-7	C46H56N4O10	Pharmaceutical (Antineoplastic, Tubulin polymerization inhibitor)
Vinorelbine	71486-22-1	C45H54N4O8	Pharmaceutical (Antineoplastic, Tubulin polymerization inhibitor)
Bosutinib	380843-75-4	C26H29Cl2N5O3	Pharmaceutical (Antineoplastic, Tyrosine kinase inhibitor)
Crizotinib	877399-52-5	C21H22Cl2FN5O	Pharmaceutical (Antineoplastic, Tyrosine kinase inhibitor)
Dasatinib	302962-49-8	C22H26ClN7O2S	Pharmaceutical (Antineoplastic, Tyrosine kinase inhibitor)
Imatinib	152459-95-5	C29H31N7O	Pharmaceutical (Antineoplastic, Tyrosine kinase inhibitor)
Nilotinib	641571-10-0	C28H22F3N7O	Pharmaceutical (Antineoplastic, Tyrosine kinase inhibitor)
Ponatinib	943319-70-8	C29H27F3N6O	Pharmaceutical (Antineoplastic, Tyrosine kinase inhibitor)
Sorafenib	284461-73-0	C21H16ClF3N4O3	Pharmaceutical (Antineoplastic, Tyrosine kinase inhibitor)
Dirlotapide	481658-94-0	C40H33F3N4O3	Pharmaceutical (Antiobesity (veterinary), Gut microsomal triglyceride transport protein (gMTP) inhibitor)
Orlistat	96829-58-2	C29H53NO5	Pharmaceutical (Antiobesity, Pancreatic lipase inhibitor)
Clorsulon	60200-06-8	C8H8Cl3N3O4S2	Pharmaceutical (Antiparasitic (fasciolicide))
Doramectin	117704-25-3	C50H74O14	Pharmaceutical (Antiparasitic (veterinary))
Eprinomectin B1a	133305-88-1	C50H75NO14	Pharmaceutical (Antiparasitic (veterinary))
Eprinomectin B1b	133305-89-2	C49H73NO14	Pharmaceutical (Antiparasitic (veterinary))
Moxidectin	113507-06-5	C37H53NO8	Pharmaceutical (Antiparasitic (veterinary))
Selamectin	165108-07-6	C43H63NO11	Pharmaceutical (Antiparasitic (veterinary))
Ivermectin	71827-03-7	C48H74O14	Pharmaceutical (Antiparasitic)
Lotilaner	1369852-71-0	C20H14Cl3F6N3O3S	Pharmaceutical (Antiparasitic)
Sarolaner	1398609-39-6	C23H18Cl2F4N2O5S	Pharmaceutical (Antiparasitic)
Carbidopa	28860-95-9	C10H14N2O4	Pharmaceutical (Antiparkinsonian (potentiator), Decarboxylase inhibitor)
Pramipexole	104632-26-0	C10H17N3S	Pharmaceutical (Antiparkinsonian, Antidepressant, Dopamine receptor agonist)
Entacapone	130929-57-6	C14H15N3O5	Pharmaceutical (Antiparkinsonian, Antidyskinetic, COMT inhibitor)
Cabergoline	81409-90-7	C26H37N5O2	Pharmaceutical (Antiparkinsonian, Antidyskinetic, Dopamine D2 receptor agonist, Prolactin inhibitor)
Pimavanserin	706779-91-1	C25H34FN3O2	Pharmaceutical (Antiparkinsonian, Antipsychotic)
Tolcapone	134308-13-7	C14H11NO5	Pharmaceutical (Antiparkinsonian, COMT inhibitor)
Rotigotine	99755-59-6	C19H25NOS	Pharmaceutical (Antiparkinsonian, Dopamine D2 receptor agonist)
Bromocriptine	25614-03-3	C32H40BrN5O5	Pharmaceutical (Antiparkinsonian, Dopamine D2 receptor agonist, Prolactin inhibitor)
Pergolide	66104-22-1	C19H26N2S	Pharmaceutical (Antiparkinsonian, Dopamine receptor agonist)

Table S20. Compound database for suspect screening (continued)

Compound Name	CAS	Molecular Formula	Category
Piribedil	3605-01-4	C16H18N4O2	Pharmaceutical (Antiparkinsonian, Dopamine receptor agonist)
Ropinirole	91374-21-9	C16H24N2O	Pharmaceutical (Antiparkinsonian, Dopamine receptor agonist)
Apomorphine	58-00-4	C17H17NO2	Pharmaceutical (Antiparkinsonian, Emetic, Impotence therapy, Dopamine receptor agonist)
Safinamide	133865-89-1	C17H19FN2O2	Pharmaceutical (Antiparkinsonian, Monoamine oxidase B (MAO-B) inhibitor)
Benztropine	86-13-5	C21H25NO	Pharmaceutical (Antiparkinsonian, Muscarinic acetylcholine receptor antagonist)
Caramiphen	77-22-5	C18H27NO2	Pharmaceutical (Antiparkinsonian, Muscarinic acetylcholine receptor antagonist)
Trihexyphenidyl	144-11-6	C20H31NO	Pharmaceutical (Antiparkinsonian, Muscarinic acetylcholine receptor antagonist)
Rasagiline	136236-51-6	C12H13N	Pharmaceutical (Antiparkinsonian, Neuroprotectant, Monoamine oxidase B (MAO-B) inhibitor)
Droxidopa	23651-95-8	C9H11NO5	Pharmaceutical (Antiparkinsonian, Replenisher (noradrenalin))
Oxybutynin	5633-20-5	C22H31NO3	Pharmaceutical (Antipollakisuria, Overactive bladder agent, Muscarinic acetylcholine receptor antagonist)
Propiverine	60569-19-9	C23H29NO3	Pharmaceutical (Antipollakisuria, Overactive bladder agent, Muscarinic acetylcholine receptor antagonist)
Flavoxate	15301-69-6	C24H25NO4	Pharmaceutical (Antipollakisuria, Phosphodiesterase inhibitor)
Ipronidazole	14885-29-1	C7H11N3O2	Pharmaceutical (Antiprotozoal (histomonas))
Benznidazole	22994-85-0	C12H12N4O3	Pharmaceutical (Antiprotozoal)
Carnidazole	42116-76-7	C8H12N4O3S	Pharmaceutical (Antiprotozoal)
Diaveridine	5355-16-8	C13H16N4O2	Pharmaceutical (Antiprotozoal)
Imidocarb	27885-92-3	C19H20N6O	Pharmaceutical (Antiprotozoal)
Miltefosine	58066-85-6	C21H46NO4P	Pharmaceutical (Antiprotozoal)
Nifurtimox	23256-30-6	C10H13N3O5S	Pharmaceutical (Antiprotozoal)
Nitazoxanide	55981-09-4	C12H9N3O5S	Pharmaceutical (Antiprotozoal)
Tinidazole	19387-91-8	C8H13N3O4S	Pharmaceutical (Antiprotozoal)
Dinitolmide	148-01-6	C8H7N3O5	Pharmaceutical (Antiprotozoal, Coccidiostat (veterinary))
Halofuginone	55837-20-2	C16H17BrClN3O3	Pharmaceutical (Antiprotozoal, Coccidiostat (veterinary))
Ponazuril	69004-04-2	C18H14F3N3O6S	Pharmaceutical (Antiprotozoal, Coccidiostat (veterinary)) / Pharmaceutical TP (Toltrazuril)
Robenidine	25875-51-8	C15H13Cl2N5	Pharmaceutical (Antiprotozoal, Coccidiostat)
Salinomycin	53003-10-4	C42H70O11	Pharmaceutical (Antiprotozoal, Coccidiostat)
Levomenthol	89-78-1	C10H20O	Pharmaceutical (Antipruritic (topical))
Oclacitinib	1208319-26-9	C15H23N5O2S	Pharmaceutical (Antipruritic, Immunosuppressant (veterinary), Janus kinase (JAK) inhibitor)
Crotamiton	483-63-6	C13H17NO	Pharmaceutical (Antipruritic, Scabicide)
Cycloheximide	66-81-9	C15H23NO4	Pharmaceutical (Antipsoriatic)
Tepoxalin	103475-41-8	C20H20ClN3O3	Pharmaceutical (Antipsoriatic)
Acitretin	55079-83-9	C21H26O3	Pharmaceutical (Antipsoriatic, Retinoic acid receptor agonist)
Calcipotriene	112965-21-6	C27H40O3	Pharmaceutical (Antipsoriatic, Vitamin D receptor agonist)
Asenapine	65576-45-6	C17H16ClNO	Pharmaceutical (Antipsychotic)
Azaperone	1649-18-9	C19H22FN3O	Pharmaceutical (Antipsychotic)
Brexpiprazole	913611-97-9	C25H27N3O2S	Pharmaceutical (Antipsychotic)
Clozapine	5786-21-0	C18H19ClN4	Pharmaceutical (Antipsychotic)
Iloperidone	133454-47-4	C24H27FN2O4	Pharmaceutical (Antipsychotic)
Olanzapine	132539-06-1	C17H20N4S	Pharmaceutical (Antipsychotic)
Haloperidol	52-86-8	C21H23ClFNO2	Pharmaceutical (Antipsychotic, Antidyskinetic, Neuroleptic, Dopamine D2 receptor antagonist)
Chlorpromazine	50-53-3	C17H19ClN2S	Pharmaceutical (Antipsychotic, Anti-emetic)
Prochlorperazine	58-38-8	C20H24ClN3S	Pharmaceutical (Antipsychotic, Anti-emetic, Dopamine D2 receptor antagonist)
Molindone	7416-34-4	C16H24N2O2	Pharmaceutical (Antipsychotic, Dopamine D2 receptor antagonist)
Perazine	84-97-9	C20H25N3S	Pharmaceutical (Antipsychotic, Dopamine D2 receptor antagonist)
Perphenazine	58-39-9	C21H26ClN3OS	Pharmaceutical (Antipsychotic, Dopamine D2 receptor antagonist)
Triflupromazine	146-54-3	C18H19F3N2S	Pharmaceutical (Antipsychotic, Dopamine D2 receptor antagonist)
Pimozide	2062-78-4	C28H29F2N3O	Pharmaceutical (Antipsychotic, Dopamine receptor antagonist)
Thiothixene	5591-45-7	C23H29N3O2S2	Pharmaceutical (Antipsychotic, Dopamine receptor antagonist)
Aripiprazole	129722-12-9	C23H27Cl2N3O2	Pharmaceutical (Antipsychotic, Dopamine receptor partial agonist, Serotonin receptor agonist/antagonist)
Cariprazine	839712-12-8	C21H32Cl2N4O	Pharmaceutical (Antipsychotic, Dopamine receptor partial agonist, Serotonin receptor partial agonist/antagonist)
Quetiapine	111974-69-7	C21H25N3O2S	Pharmaceutical (Antipsychotic, Neuroleptic)
Risperidone	106266-06-2	C23H27FN4O2	Pharmaceutical (Antipsychotic, Neuroleptic)
Ziprasidone	146939-27-7	C21H21ClN4OS	Pharmaceutical (Antipsychotic, Neuroleptic)
Fluphenazine	69-23-8	C22H26F3N3OS	Pharmaceutical (Antipsychotic, Neuroleptic, Dopamine D2 receptor antagonist)
Trifluoperazine	117-89-5	C21H24F3N3S	Pharmaceutical (Antipsychotic, Neuroleptic, Dopamine D2 receptor antagonist)
Thioridazine	50-52-2	C21H26N2S2	Pharmaceutical (Antipsychotic, Sedative-hypnotic, Dopamine D2 receptor antagonist)
Lurasidone	367514-87-2	C28H36N4O2S	Pharmaceutical (Antipsychotic, Serotonin receptor antagonist, Dopamine D2 receptor antagonist)
Paliperidone	144598-75-4	C23H27FN4O3	Pharmaceutical (Antipsychotic, Serotonin receptor antagonist, Dopamine D2 receptor antagonist)
Tiludronic Acid	89987-06-4	C7H9ClO6P2S	Pharmaceutical (Antiresorptive)
Pamidronic Acid	40391-99-9	C3H11NO7P2	Pharmaceutical (Antiresorptive, Farnesylpyrophosphate synthetase inhibitor)
Risedronic Acid	115436-72-1	C7H11NO7P2	Pharmaceutical (Antiresorptive, Farnesylpyrophosphate synthetase inhibitor)
Alendronic Acid	66376-36-1	C4H13NO7P2	Pharmaceutical (Antiresorptive, Osteoporosis agent, Farnesylpyrophosphate synthetase inhibitor)
Ibandronate	114084-78-5	C9H23NO7P2	Pharmaceutical (Antiresorptive, Osteoporosis agent, Farnesylpyrophosphate synthetase inhibitor)
Leflunomide	75706-12-6	C12H9F3N2O2	Pharmaceutical (Antirheumatic, Dihydroorotate dehydrogenase inhibitor)
Tofacitinib	477600-75-2	C16H20N6O	Pharmaceutical (Antirheumatic, Janus kinase (JAK) inhibitor)
Pirenzepine	28797-61-7	C19H21N5O2	Pharmaceutical (Antisecretory (gastric acid), Anti-ulcerative, Muscarinic acetylcholine receptor antagonist)
Esomeprazole	119141-88-7	C17H19N3O3S	Pharmaceutical (Antisecretory (gastric acid), Anti-ulcerative, Proton pump inhibitor)

Table S20. Compound database for suspect screening (continued)

Compound Name	CAS	Molecular Formula	Category
Omeprazole	73590-58-6	C17H19N3O3S	Pharmaceutical (Antisecretory (gastric acid), Anti-ulcerative, Proton pump inhibitor)
Rabeprazole	117976-89-3	C18H21N3O3S	Pharmaceutical (Antisecretory (gastric acid), Anti-ulcerative, Proton pump inhibitor)
Trilostane	13647-35-3	C20H27NO3	Pharmaceutical (Antisecretory (hormone), Adrenocortical hormone biosynthesis inhibitor)
Chlorhexidine	55-56-1	C22H30Cl2N10	Pharmaceutical (Antiseptic, Disinfectant, Cell membrane function inhibitor)
Aminopentamide	60-46-8	C19H24N2O	Pharmaceutical (Antispasmodic (veterinary))
Dicyclomine	77-19-0	C19H35NO2	Pharmaceutical (Antispasmodic, Muscarinic acetylcholine receptor antagonist)
Hyoscyamine	101-31-5	C17H23NO3	Pharmaceutical (Antispasmodic, Muscarinic acetylcholine receptor antagonist)
Cyclobenzaprine	303-53-7	C20H21N	Pharmaceutical (Antispasmodic, Muscle relaxant)
Baclofen	1134-47-0	C10H12ClNO2	Pharmaceutical (Antispasmodic, Muscle relaxant, GABA-B receptor agonist)
Papaverine	58-74-2	C20H21NO4	Pharmaceutical (Antispasmodic, Smooth muscle relaxant, Vasodilator, Phosphodiesterase inhibitor)
Pegacaristim	187139-68-0	C9H19NO5	Pharmaceutical (Antithrombocytopenia, Megakaryocyte stimulating factor)
Eltrombopag	496775-61-2	C25H22N4O4	Pharmaceutical (Antithrombocytopenia, Thrombopoietin receptor agonist)
Betrixaban	330942-05-7	C23H22ClN5O3	Pharmaceutical (Antithrombotic, Factor Xa inhibitor)
Edoxaban	480449-70-5	C24H30ClN7O4S	Pharmaceutical (Antithrombotic, Factor Xa inhibitor)
Methimazole	60-56-0	C4H6N2S	Pharmaceutical (Antithyroid, Thyroid hormone synthesis inhibitor)
Propylthiouracil	51-52-5	C7H10N2OS	Pharmaceutical (Antithyroid, Thyroid hormone synthesis inhibitor)
Benzonatate	104-31-4	C30H53NO11	Pharmaceutical (Antitussive)
Dextromethorphan	125-71-3	C18H25NO	Pharmaceutical (Antitussive)
Nicocodine	3688-66-2	C24H24N2O4	Pharmaceutical (Antitussive)
Noscapine	128-62-1	C22H23NO7	Pharmaceutical (Antitussive)
Pholcodine	509-67-1	C23H30N2O4	Pharmaceutical (Antitussive)
Guaifenesin	93-14-1	C10H14O4	Pharmaceutical (Antitussive, Expectorant)
Hydrocodone	125-29-1	C18H21NO3	Pharmaceutical (Antitussive, Opioid receptor agonist)
Nitisinone	104206-65-7	C14H10F3NO5	Pharmaceutical (Antityrosinemia)
Cimetidine	51481-61-9	C10H16N6S	Pharmaceutical (Anti-ulcerative, H2 receptor antagonist)
Famotidine	76824-35-6	C8H15N7O2S3	Pharmaceutical (Anti-ulcerative, H2 receptor antagonist)
Nizatidine	76963-41-2	C12H21N5O2S2	Pharmaceutical (Anti-ulcerative, H2 receptor antagonist)
Ranitidine	66357-35-5	C13H22N4O3S	Pharmaceutical (Anti-ulcerative, H2 receptor antagonist)
Misoprostol	59122-46-2	C22H38O5	Pharmaceutical (Anti-ulcerative, Prostaglandin E receptor agonist)
Dexlansoprazole	138530-94-6	C16H14F3N3O2S	Pharmaceutical (Anti-ulcerative, Proton pump inhibitor)
Lansoprazole	103577-45-3	C16H14F3N3O2S	Pharmaceutical (Anti-ulcerative, Proton pump inhibitor)
Pantoprazole	102625-70-7	C16H15F2N3O4S	Pharmaceutical (Anti-ulcerative, Proton pump inhibitor)
Polidocanol	3055-99-0	C30H62O10	Pharmaceutical (Antivaricose, Hemostatic, Sclerosing agent)
Cobicistat	1004316-88-4	C40H53N7O5S2	Pharmaceutical (Antiviral (potentiator), CYP3A inhibitor)
Letermovir	917389-32-3	C29H28F4N4O4	Pharmaceutical (Antiviral)
Ribavirin	36791-04-5	C8H12N4O5	Pharmaceutical (Antiviral)
Maraviroc	376348-65-1	C29H41F2N5O	Pharmaceutical (Antiviral, CCR5 antagonist)
Valganciclovir	175865-60-8	C14H22N6O5	Pharmaceutical (Antiviral, DNA polymerase ihibitor)
Acyclovir	59277-89-3	C8H11N5O3	Pharmaceutical (Antiviral, DNA polymerase inhibitor)
Cidofovir	113852-37-2	C8H14N3O6P	Pharmaceutical (Antiviral, DNA polymerase inhibitor)
Famciclovir	104227-87-4	C14H19N5O4	Pharmaceutical (Antiviral, DNA polymerase inhibitor)
Ganciclovir	82410-32-0	C9H13N5O4	Pharmaceutical (Antiviral, DNA polymerase inhibitor)
Penciclovir	39809-25-1	C10H15N5O3	Pharmaceutical (Antiviral, DNA polymerase inhibitor)
Valacyclovir	124832-26-4	C13H20N6O4	Pharmaceutical (Antiviral, DNA polymerase inhibitor)
Dolutegravir	1051375-16-6	C20H19F2N3O5	Pharmaceutical (Antiviral, HIV integrase inhibitor)
Raltegravir	518048-05-0	C20H21FN6O5	Pharmaceutical (Antiviral, HIV integrase inhibitor)
Atazanavir	198904-31-3	C38H52N6O7	Pharmaceutical (Antiviral, HIV protease inhibitor)
Darunavir	206361-99-1	C27H37N3O7S	Pharmaceutical (Antiviral, HIV protease inhibitor)
Elvitegravir	697761-98-1	C23H23ClFNO5	Pharmaceutical (Antiviral, HIV protease inhibitor)
Fosamprenavir	226700-79-4	C25H36N3O9PS	Pharmaceutical (Antiviral, HIV protease inhibitor)
Indinavir	150378-17-9	C36H47N5O4	Pharmaceutical (Antiviral, HIV protease inhibitor)
Lopinavir	192725-17-0	C37H48N4O5	Pharmaceutical (Antiviral, HIV protease inhibitor)
Nelfinavir	159989-64-7	C32H45N3O4S	Pharmaceutical (Antiviral, HIV protease inhibitor)
Ritonavir	155213-67-5	C37H48N6O5S2	Pharmaceutical (Antiviral, HIV protease inhibitor)
Saquinavir	127779-20-8	C38H50N6O5	Pharmaceutical (Antiviral, HIV protease inhibitor)
Tipranavir	174484-41-4	C31H33F3N2O5S	Pharmaceutical (Antiviral, HIV protease inhibitor)
Imiquimod	99011-02-6	C14H16N4	Pharmaceutical (Antiviral, Immunomodulator, Toll-like receptor agonist)
Rimantadine	13392-28-4	C12H21N	Pharmaceutical (Antiviral, M2 protein inhibitor)
Amantadine	768-94-5	C10H17N	Pharmaceutical (Antiviral, M2 protein inhibitor, Antiparkinsonian, Dopamine secretagogue)
Oseltamivir	196618-13-0	C16H28N2O4	Pharmaceutical (Antiviral, Neuraminidase inhibitor)
Peramivir	330600-85-6	C15H28N4O4	Pharmaceutical (Antiviral, Neuraminidase inhibitor)
Zanamivir	139110-80-8	C12H20N4O7	Pharmaceutical (Antiviral, Neuraminidase inhibitor)
Daclatasvir	1009119-64-5	C40H50N8O6	Pharmaceutical (Antiviral, NS5A inhibitor)
Elbasvir	1370468-36-2	C49H55N9O7	Pharmaceutical (Antiviral, NS5A inhibitor)
Ledipasvir	1256388-51-8	C49H54F2N8O6	Pharmaceutical (Antiviral, NS5A inhibitor)
Ombitasvir	1258226-87-7	C50H67N7O8	Pharmaceutical (Antiviral, NS5A inhibitor)
Dasabuvir	1132935-63-7	C26H27N3O5S	Pharmaceutical (Antiviral, NS5B polymerase inhibitor)

Table S20. Compound database for suspect screening (continued)

Compound Name	CAS	Molecular Formula	Category
Sofosbuvir	1190307-88-0	C22H29FN3O9P	Pharmaceutical (Antiviral, NS5B polymerase inhibitor)
Trifluridine	70-00-8	C10H11F3N2O5	Pharmaceutical (Antiviral, Nucleic acid biosynthesis inhibitor)
Glecaprevir	1365970-03-1	C38H46F4N6O9S	Pharmaceutical (Antiviral, Protease inhibitor)
Simeprevir	923604-59-5	C38H47N5O7S2	Pharmaceutical (Antiviral, Protease inhibitor)
Abacavir	136470-78-5	C14H18N6O	Pharmaceutical (Antiviral, Reverse transcriptase inhibitor)
Adefovir	106941-25-7	C8H12N5O4P	Pharmaceutical (Antiviral, Reverse transcriptase inhibitor)
Delavirdine	136817-59-9	C22H28N6O3S	Pharmaceutical (Antiviral, Reverse transcriptase inhibitor)
Didanosine	69655-05-6	C10H12N4O3	Pharmaceutical (Antiviral, Reverse transcriptase inhibitor)
Efavirenz	154598-52-4	C14H9ClF3NO2	Pharmaceutical (Antiviral, Reverse transcriptase inhibitor)
Emtricitabine	143491-57-0	C8H10FN3O3S	Pharmaceutical (Antiviral, Reverse transcriptase inhibitor)
Entecavir	142217-69-4	C12H15N5O3	Pharmaceutical (Antiviral, Reverse transcriptase inhibitor)
Etravirine	269055-15-4	C20H15BrN6O	Pharmaceutical (Antiviral, Reverse transcriptase inhibitor)
Lamivudine	134678-17-4	C8H11N3O3S	Pharmaceutical (Antiviral, Reverse transcriptase inhibitor)
Nevirapine	129618-40-2	C15H14N4O	Pharmaceutical (Antiviral, Reverse transcriptase inhibitor)
Rilpivirine	500287-72-9	C22H18N6	Pharmaceutical (Antiviral, Reverse transcriptase inhibitor)
Stavudine	3056-17-5	C10H12N2O4	Pharmaceutical (Antiviral, Reverse transcriptase inhibitor)
Tenofovir	147127-20-6	C9H14N5O4P	Pharmaceutical (Antiviral, Reverse transcriptase inhibitor)
Zalcitabine	7481-89-2	C9H13N3O3	Pharmaceutical (Antiviral, Reverse transcriptase inhibitor)
Zidovudine	30516-87-1	C10H13N5O4	Pharmaceutical (Antiviral, Reverse transcriptase inhibitor)
Methoxsalen	298-81-7	C12H8O4	Pharmaceutical (Antivitiligo, Pigmentation agent)
Phendimetrazine	634-03-7	C12H17NO	Pharmaceutical (Appetite suppressant (systemic), Stimulant (central))
Amfepramone (Diethylpropion)	90-84-6	C13H19NO	Pharmaceutical (Appetite suppressant)
Aminorex	2207-50-3	C9H10N2O	Pharmaceutical (Appetite suppressant)
Benzphetamine	156-08-1	C17H21N	Pharmaceutical (Appetite suppressant)
Cathine	492-39-7	C9H13NO	Pharmaceutical (Appetite suppressant)
Chlorphentermine	461-78-9	C10H14ClN	Pharmaceutical (Appetite suppressant)
Fenfluramine	458-24-2	C12H16F3N	Pharmaceutical (Appetite suppressant)
Fenproporex	16397-28-7	C12H16N2	Pharmaceutical (Appetite suppressant)
Mazindol	22232-71-9	C16H13ClN2O	Pharmaceutical (Appetite suppressant)
Mefenorex	17243-57-1	C12H18ClN	Pharmaceutical (Appetite suppressant)
Phentermine	122-09-8	C10H15N	Pharmaceutical (Appetite suppressant)
Sibutramine	106650-56-0	C17H26ClN	Pharmaceutical (Appetite suppressant, Antidepressant, Serotonin-noradrenaline reuptake inhibitor (SNRI))
Phenmetrazine	134-49-6	C11H15NO	Pharmaceutical (Appetite suppressant, Monoamine releaser)
Lorcaserin	616202-92-7	C11H14ClN	Pharmaceutical (Appetite suppressant, Serotonin 5-HT2c receptor agonist)
Dexfenfluramine	3239-44-9	C12H16F3N	Pharmaceutical (Appetite suppressant, Serotonin reuptake inhibitor)
Ephedrine	299-42-3	C10H15NO	Pharmaceutical (Bronchodilator, Adrenergic receptor agonist)
Albuterol (Salbutamol)	18559-94-9	C13H21NO3	Pharmaceutical (Bronchodilator, beta2-Adrenergic receptor agonist)
Formoterol	73573-87-2	C19H24N2O4	Pharmaceutical (Bronchodilator, beta2-Adrenergic receptor agonist)
Indacaterol	312753-06-3	C24H28N2O3	Pharmaceutical (Bronchodilator, beta2-Adrenergic receptor agonist)
Metaproterenol (Orciprenaline)	586-06-1	C11H17NO3	Pharmaceutical (Bronchodilator, beta2-Adrenergic receptor agonist)
Olodaterol	868049-49-4	C21H26N2O5	Pharmaceutical (Bronchodilator, beta2-Adrenergic receptor agonist)
Salmeterol	89365-50-4	C25H37NO4	Pharmaceutical (Bronchodilator, beta2-Adrenergic receptor agonist)
Terbutaline	23031-25-6	C12H19NO3	Pharmaceutical (Bronchodilator, beta2-Adrenergic receptor agonist)
Isoprenaline	7683-59-2	C11H17NO3	Pharmaceutical (Bronchodilator, beta-Adrenergic receptor agonist)
Clenbuterol	37148-27-9	C12H18Cl2N2O	Pharmaceutical (Bronchodilator, Overactive bladder agent, beta2-Adrenergic receptor agonist)
Theophylline	58-55-9	C7H8N4O2	Pharmaceutical (Bronchodilator, Phosphodiesterase inhibitor)
Epinephrine (Adrenaline)	51-43-4	C9H13NO3	Pharmaceutical (Bronchodilator, Vasoconstrictor, Adrenergic receptor agonist)
Digoxin	20830-75-5	C41H64O14	Pharmaceutical (Cardiotonic)
Ivabradine	155974-00-8	C27H36N2O5	Pharmaceutical (Cardiotonic)
Dobutamine	34368-04-2	C18H23NO3	Pharmaceutical (Cardiotonic, beta1-Adrenergic receptor agonist)
Dopamine	51-61-6	C8H11NO2	Pharmaceutical (Cardiotonic, Dopamine receptor agonist)
Inamrinone	60719-84-8	C10H9N3O	Pharmaceutical (Cardiotonic, Phosphodiesterase 3 inhibitor)
Milrinone	78415-72-2	C12H9N3O	Pharmaceutical (Cardiotonic, Phosphodiesterase 3 inhibitor)
Pimobendan	118428-36-7	C19H18N4O2	Pharmaceutical (Cardiotonic, Phosphodiesterase inhibitor)
Edaravone	89-25-8	C10H10N2O	Pharmaceutical (Cerebroprotective)
Obeticholic Acid	459789-99-2	C26H44O4	Pharmaceutical (Choleretic)
Deoxycholic Acid	83-44-3	C24H40O4	Pharmaceutical (Choleretic, Cytolytic)
Cholic Acid	81-25-4	C24H40O5	Pharmaceutical (Choleretic, Supplement (bile acid))
Clopidol	2971-90-6	C7H7Cl2NO	Pharmaceutical (Coccidiostat (for poultry))
Decoquinate	18507-89-6	C24H35NO5	Pharmaceutical (Coccidiostat (for poultry))
Diclazuril	101831-37-2	C17H9Cl3N4O2	Pharmaceutical (Coccidiostat (for poultry))
Lasalocid	25999-31-9	C34H54O8	Pharmaceutical (Coccidiostat (for poultry))
Nequinate	13997-19-8	C22H23NO4	Pharmaceutical (Coccidiostat (for poultry))
Nicarbazin	587-90-6	C13H10N4O5	Pharmaceutical (Coccidiostat (for poultry))
Toltrazuril	69004-03-1	C18H14F3N3O4S	Pharmaceutical (Coccidiostat (veterinary))
Sulfabromomethazine	116-45-0	C12H13BrN4O2S	Pharmaceutical (Coccidiostat (veterinary), Folic acid biosynthesis inhibitor)
Sulfaquinoxaline	59-40-5	C14H12N4O2S	Pharmaceutical (Coccidiostat (veterinary), Folic acid biosynthesis inhibitor)

Table S20. Compound database for suspect screening (continued)

Compound Name	CAS	Molecular Formula	Category
Narasin	55134-13-9	C43H72O11	Pharmaceutical (Coccidiostat (veterinary), Growth stimulant (veterinary))
Ethopabate	59-06-3	C12H15NO4	Pharmaceutical (Coccidiostat)
Maduramicin	61991-54-6	C47H80O17	Pharmaceutical (Coccidiostat)
Semduramicin	113378-31-7	C45H76O16	Pharmaceutical (Coccidiostat)
Drospirenone	67392-87-4	C24H30O3	Pharmaceutical (Contraceptive, Aldosterone antagonist)
Mestranol	72-33-3	C21H26O2	Pharmaceutical (Contraceptive, Estrogen receptor agonist)
Desogestrel	54024-22-5	C22H30O	Pharmaceutical (Contraceptive, Progesterone receptor agonist)
Ethynodiol	1231-93-2	C20H28O2	Pharmaceutical (Contraceptive, Progesterone receptor agonist)
Levonorgestrel	797-63-7	C21H28O2	Pharmaceutical (Contraceptive, Progesterone receptor agonist)
Norethindrone	68-22-4	C20H26O2	Pharmaceutical (Contraceptive, Progesterone receptor agonist)
Norgestimate	35189-28-7	C23H31NO3	Pharmaceutical (Contraceptive, Progesterone receptor agonist)
Norgestrel	6533-00-2	C21H28O2	Pharmaceutical (Contraceptive, Progesterone receptor agonist)
Etonogestrel	54048-10-1	C22H28O2	Pharmaceutical (Contraceptive, Progesterone receptor agonist) / Pharmaceutical TP (Desogestrel)
Ulipristal	159811-51-5	C28H35NO3	Pharmaceutical (Contraceptive, Progesterone receptor agonist/antagonist)
Ivacaftor	873054-44-5	C24H28N2O3	Pharmaceutical (Cystic fibrosis transmembrane conductance regulator (CFTR) potentiator)
Donepezil	120014-06-4	C24H29NO3	Pharmaceutical (Dementia therapeutic agent, Acetylcholinesterase inhibitor)
Galantamine	357-70-0	C17H21NO3	Pharmaceutical (Dementia therapeutic agent, Acetylcholinesterase inhibitor)
Rivastigmine	123441-03-2	C14H22N2O2	Pharmaceutical (Dementia therapeutic agent, Acetylcholinesterase inhibitor)
Memantine	19982-08-2	C12H21N	Pharmaceutical (Dementia therapeutic agent, NMDA receptor antagonist)
Macimorelin	381231-18-1	C26H30N6O3	Pharmaceutical (Diagnostic (adult growth hormone deficiency (AGHD)), Growth hormone secretagogue receptor (GHSR) /ghrelin receptor agonist)
Hexaminolevulinate	140898-97-1	C11H21NO3	Pharmaceutical (Diagnostic aid (contrast medium, bladder), Photosensitizer)
Fluorescein	2321-07-5	C20H12O5	Pharmaceutical (Diagnostic aid (corneal trauma indicator))
Metyrapone	54-36-4	C14H14N2O	Pharmaceutical (Diagnostic aid (pituitary function determination))
Diatrizoic Acid (Diatrizoate)	117-96-4	C11H9I3N2O4	Pharmaceutical (Diagnostic aid (radiopaque medium))
Iopromide	73334-07-3	C18H24I3N3O8	Pharmaceutical (Diagnostic aid (radiopaque medium))
Iothalamic Acid	2276-90-6	C11H9I3N2O4	Pharmaceutical (Diagnostic aid (radiopaque medium))
Isosorbide	652-67-5	C6H10O4	Pharmaceutical (Diuretic)
Triamterene	396-01-0	C12H11N7	Pharmaceutical (Diuretic, Aldosterone antagonist, Epithelial sodium channel blocker)
Amiloride	2609-46-3	C6H8ClN7O	Pharmaceutical (Diuretic, Epithelial sodium channel blocker)
Bumetanide	28395-03-1	C17H20N2O5S	Pharmaceutical (Diuretic, Na-K-Cl cotransporter inhibitor)
Ethacrynic Acid	58-54-8	C13H12Cl2O4	Pharmaceutical (Diuretic, Na-K-Cl cotransporter inhibitor)
Furosemide	54-31-9	C12H11ClN2O5S	Pharmaceutical (Diuretic, Na-K-Cl cotransporter inhibitor)
Torsemide	56211-40-6	C16H20N4O3S	Pharmaceutical (Diuretic, Na-K-Cl cotransporter inhibitor)
Conivaptan	210101-16-9	C32H26N4O2	Pharmaceutical (Diuretic, Vasopressin receptor antagonist)
Tolvaptan	150683-30-0	C26H25ClN2O3	Pharmaceutical (Diuretic, Vasopressin V2 receptor antagonist)
Fluralaner	864731-61-3	C22H17Cl2F6N3O3	Pharmaceutical (Ectoparasiticide (veterinary))
Norgestomet	25092-41-5	C23H32O4	Pharmaceutical (Estrus suppressant (veterinary))
Altrenogest	850-52-2	C21H26O2	Pharmaceutical (Estuary inducer (veterinary))
Urofollitropin	97048-13-0	C42H65N11O12S2	Pharmaceutical (Follicle stimulating hormone receptor agonist)
Eliglustat	491833-29-5	C23H36N2O4	Pharmaceutical (Glucosylceramide synthase inhibitor)
Colchicine	64-86-8	C22H25NO6	Pharmaceutical (Gout suppressant, Leukocyte (neutrophil) migration inhibitor, Tubulin polymerization inhibitor)
Allopurinol	315-30-0	C5H4N4O	Pharmaceutical (Gout suppressant, Uric acid biosynthesis inhibitor, Xanthine oxidase inhibitor)
Febuxostat	144060-53-7	C16H16N2O3S	Pharmaceutical (Gout suppressant, Uric acid biosynthesis inhibitor, Xanthine oxidase inhibitor)
Probenecid	57-66-9	C13H19NO4S	Pharmaceutical (Gout suppressant, Uricosuric)
Lesinurad	878672-00-5	C17H14BrN3O2S	Pharmaceutical (Gout suppressant, Uricosuric, Urate transporter inhibitor)
Laidlomycin	56283-74-0	C37H62O12	Pharmaceutical (Growth stimulant (veterinary))
Melengestrol	5633-18-1	C23H30O3	Pharmaceutical (Growth stimulant (veterinary))
Ractopamine	97825-25-7	C18H23NO3	Pharmaceutical (Growth stimulant (veterinary))
Zilpaterol	119520-05-7	C14H19N3O2	Pharmaceutical (Growth stimulant (veterinary))
Methylergonovine	113-42-8	C20H25N3O2	Pharmaceutical (Hemostatic, Oxytocic)
Ramelteon	196597-26-9	C16H21NO2	Pharmaceutical (Hypnotic, Melatonin receptor agonist)
Tasimelteon	609799-22-6	C15H19NO2	Pharmaceutical (Hypnotic, Melatonin receptor agonist)
Suvorexant	1030377-33-3	C23H23ClN6O2	Pharmaceutical (Hypnotic, Orexin receptor antagonist)
Pimecrolimus	137071-32-0	C43H68ClNO11	Pharmaceutical (Immunosuppressant, Calcineurin inhibitor)
Mycophenolic Acid	24280-93-1	C17H20O6	Pharmaceutical (Immunosuppressant, Inosine monophosphate dehydrogenase inhibitor)
Azathioprine	446-86-6	C9H7N7O2S	Pharmaceutical (Immunosuppressant, Nucleic acid biosynthesis inhibitor)
Fingolimod	162359-55-9	C19H33NO2	Pharmaceutical (Immunosuppressant, Sphingosine-1-phosphate receptor agonist)
Avanafil	330784-47-9	C23H26ClN7O3	Pharmaceutical (Impotence therapy, Phosphodiesterase V inhibitor)
Bisacodyl	603-50-9	C22H19NO4	Pharmaceutical (Laxative (suppository))
Alvimopan	156053-89-3	C25H32N2O4	Pharmaceutical (Laxative, Opioid receptor antagonist)
Naloxegol	854601-70-0	C34H53NO11	Pharmaceutical (Laxative, Opioid receptor antagonist)
Fluprostenol	40666-16-8	C23H29F3O6	Pharmaceutical (Luteolytic (veterinary))
Fenprostalene	69381-94-8	C23H30O6	Pharmaceutical (Luteolytic)
Miglustat	72599-27-0	C10H21NO4	Pharmaceutical (Lysosomal storage disease treatment, Glucosylceramide synthase inhibitor)
17beta-Estradiol (Estradiol)	50-28-2	C18H24O2	Pharmaceutical (Menstruation disorder agent, Estrogen receptor agonist)
Estriol	50-27-1	C18H24O3	Pharmaceutical (Menstruation disorder agent, Estrogen receptor agonist)
Progesterone	57-83-0	C21H30O2	Pharmaceutical (Menstruation disorder agent, Progesterone receptor agonist)

Table S20. Compound database for suspect screening (continued)

Compound Name	CAS	Molecular Formula	Category
Hydroxyprogesterone (17alpha-Hydroxyprogesterone)	68-96-2	C21H30O3	Pharmaceutical (Menstruation disorder agent, Progesterone receptor agonist) / Pharmaceutical TP (Progesterone)
Delorazepam	2894-67-9	C15H10Cl2N2O	Pharmaceutical (Minor tranquilizer)
Ketazolam	27223-35-4	C20H17ClN2O3	Pharmaceutical (Minor tranquilizer)
Oxazepam	604-75-1	C15H11ClN2O2	Pharmaceutical (Minor tranquilizer)
Temazepam	846-50-4	C16H13ClN2O2	Pharmaceutical (Minor tranquilizer) / Pharmaceutical TP (Diazepam)
Loxapine	1977-10-2	C18H18ClN3O	Pharmaceutical (Minor tranquilizer, Dopamine D2 receptor antagonist, Serotonin receptor antagonist)
Tetrazepam	10379-14-3	C16H17ClN2O	Pharmaceutical (Muscle relaxant)
Tolperisone	728-88-1	C16H23NO	Pharmaceutical (Muscle relaxant)
Tizanidine	51322-75-9	C9H8ClN5S	Pharmaceutical (Muscle relaxant, alpha2-Adrenergic receptor agonist)
Hydroxyamphetamine	103-86-6	C9H13NO	Pharmaceutical (Mydriatic)
Atropine	51-55-8	C17H23NO3	Pharmaceutical (Mydriatic, Muscarinic acetylcholine receptor antagonist)
Cyclopentolate	512-15-2	C17H25NO3	Pharmaceutical (Mydriatic, Muscarinic acetylcholine receptor antagonist)
Homatropine	87-00-3	C16H21NO3	Pharmaceutical (Mydriatic, Muscarinic acetylcholine receptor antagonist)
Scopolamine	51-34-3	C17H21NO4	Pharmaceutical (Mydriatic, Muscarinic acetylcholine receptor antagonist)
Tropicamide	1508-75-4	C17H20N2O2	Pharmaceutical (Mydriatic, Muscarinic acetylcholine receptor antagonist)
Naloxone	465-65-6	C19H21NO4	Pharmaceutical (Narcotic antagonist, Respiratory stimulant, Opioid receptor antagonist)
Riluzole	1744-22-5	C8H5F3N2OS	Pharmaceutical (Nerve cells protectant)
Promazine	58-40-2	C17H20N2S	Pharmaceutical (Neuroleptic, Dopamine D2 receptor antagonist)
Tiapride	51012-32-9	C15H24N2O4S	Pharmaceutical (Neuroleptic, Dopamine D2 receptor antagonist)
Tetrabenazine	58-46-8	C19H27NO3	Pharmaceutical (Neuroleptic, Vesicular monoamine transporter inhibitor)
Valbenazine	1025504-45-3	C24H38N2O4	Pharmaceutical (Neuroleptic, Vesicular monoamine transporter inhibitor)
Bazedoxifene	198481-32-2	C30H34N2O3	Pharmaceutical (Osteoporosis agent, Selective estrogen receptor modulator (SERM))
Ospemifene	128607-22-7	C24H23ClO2	Pharmaceutical (Osteoporosis agent, Selective estrogen receptor modulator (SERM))
Raloxifene	84449-90-1	C28H27NO4S	Pharmaceutical (Osteoporosis agent, Selective estrogen receptor modulator (SERM))
Calcitriol	32222-06-3	C27H44O3	Pharmaceutical (Osteoporosis agent, Vitamin D receptor agonist)
Mirabegron	223673-61-8	C21H24N4O2S	Pharmaceutical (Overactive bladder agent, beta3-Adrenergic receptor agonist)
Darifenacin	133099-04-4	C28H30N2O2	Pharmaceutical (Overactive bladder agent, Muscarinic acetylcholine receptor antagonist)
Fesoterodine	286930-02-7	C26H37NO3	Pharmaceutical (Overactive bladder agent, Muscarinic acetylcholine receptor antagonist)
Solifenacin	242478-37-1	C23H26N2O2	Pharmaceutical (Overactive bladder agent, Muscarinic acetylcholine receptor antagonist)
Tolterodine	124937-51-5	C22H31NO	Pharmaceutical (Overactive bladder agent, Muscarinic acetylcholine receptor antagonist)
Clomifene (Clomiphene)	911-45-5	C26H28ClNO	Pharmaceutical (Ovulation inducing agent, Gonadotropin stimulant, Estrogen receptor agonist/antagonist)
Cloprostenol	40665-92-7	C22H29ClO6	Pharmaceutical (Oxytocic (veterinary))
Luprostiol	67110-79-6	C21H29ClO6S	Pharmaceutical (Oxytocic)
Prostalene	54120-61-5	C22H36O5	Pharmaceutical (Oxytocic)
Dinoprostone	363-24-6	C20H32O5	Pharmaceutical (Oxytocic, Prostaglandin E2 receptor agonist)
Carboprost	35700-23-3	C21H36O5	Pharmaceutical (Oxytocic, Prostaglandin F receptor agonist)
Dinoprost	551-11-1	C20H34O5	Pharmaceutical (Oxytocic, Prostaglandin F receptor agonist)
Spinosad A	131929-60-7	C41H65NO10	Pharmaceutical (Pediculicide (veterinary), Nicotinic acetylcholine receptor allosteric site modulator)
Lubiprostone	136790-76-6	C20H32F2O5	Pharmaceutical (Peristaltic accelerator, Laxative, Clc chloride channel inhibitor)
Vorapaxar	618385-01-6	C29H33FN2O4	Pharmaceutical (Platelet aggregation inhibitor, Coagulation factor II receptor (PAR1) antagonist)
Eptifibatide	188627-80-7	C35H49N11O9S2	Pharmaceutical (Platelet aggregation inhibitor, Glycoprotein IIb/IIIa receptor antagonist)
Tirofiban	144494-65-5	C22H36N2O5S	Pharmaceutical (Platelet aggregation inhibitor, Glycoprotein IIb/IIIa receptor antagonist)
Epoprostenol	35121-78-9	C20H32O5	Pharmaceutical (Platelet aggregation inhibitor, Prostaglandin I2 receptor agonist)
Cangrelor	163706-06-7	C17H25Cl2F3N5O12P3S2	Pharmaceutical (Platelet aggregation inhibitor, Purinergic receptor P2Y12 antagonist)
Prasugrel	150322-43-3	C20H20FNO3S	Pharmaceutical (Platelet aggregation inhibitor, Purinergic receptor P2Y12 antagonist)
Ticagrelor	274693-27-5	C23H28F2N6O4S	Pharmaceutical (Platelet aggregation inhibitor, Purinergic receptor P2Y12 antagonist)
Cilastatin	82009-34-5	C16H26N2O5S	Pharmaceutical (Potentiator (antibacterial), Dehydropeptidase I inhibitor)
Androstenedione	63-05-8	C19H26O2	Pharmaceutical (Prohormone)
Prucalopride	179474-81-8	C18H26ClN3O3	Pharmaceutical (Prokinetic, Serotonin receptor agonist)
Dutasteride	164656-23-9	C27H30F6N2O2	Pharmaceutical (Prostatic hyperplasia treatment, Antiandrogen, 5alpha-Reductase inhibitor)
Methamphetamine	537-46-2	C10H15N	Pharmaceutical (Psychostimulant, Stimulant (central)) / Pharmaceutical TP (Selegiline)
Amifostine	20537-88-6	C5H15N2O3PS	Pharmaceutical (Radioprotector (topical))
Methyltestosterone	58-18-4	C20H30O2	Pharmaceutical (Replenisher (androgen), Androgen receptor agonist)
Testosterone	58-22-0	C19H28O2	Pharmaceutical (Replenisher (androgen), Androgen receptor agonist)
Levocarnitine	541-15-1	C7H15NO3	Pharmaceutical (Replenisher (carnitine))
Equilin	474-86-2	C18H20O2	Pharmaceutical (Replenisher (estrogen), Estrogen receptor agonist)
Estrone	53-16-7	C18H22O2	Pharmaceutical (Replenisher (estrogen), Estrogen receptor agonist)
Levothyroxine	51-48-9	C15H11I4NO4	Pharmaceutical (Replenisher (thyroid hormone))
Liothyronine	6893-02-3	C15H12I3NO4	Pharmaceutical (Replenisher (thyroid hormone))
Doxapram	309-29-5	C24H30N2O2	Pharmaceutical (Respiratory stimulant)
Cevimeline	107233-08-9	C10H17NOS	Pharmaceutical (Salivation accelaletor, Muscarinic acetylcholine receptor agonist)
Fludrocortisone	127-31-1	C21H29FO5	Pharmaceutical (Salts metabolic regulator)
Desoxycortone (21-Hydroxyprogesterone)	64-85-7	C21H30O3	Pharmaceutical (Salts metabolic regulator, Mineralocorticoid receptor agonist) / Pharmaceutical TP (Progesterone)
1,3-Benzodioxolyl-N-methylbutanamine (MBDB)	103818-46-8	C12H17NO2	Pharmaceutical (Schedule I Drug of Abuse)
(1-(5-Fluoropentyl)-1H-indazol-3-yl)(naphthalen-1-yl)methanone (THJ-2201)	1801552-01-1	C23H21FN2O	Pharmaceutical (Schedule I Drug of Abuse)
(1-Pentyl-1H-indol-3-yl)(2,2,3,3-tetramethylcyclopropyl)methanone (UR-144)	1199943-44-6	C21H29NO	Pharmaceutical (Schedule I Drug of Abuse)
[1-(5-Fluoropentyl)-1H-indol-3-yl]-1-naphthalenylmethanone (AM-2201)	335161-24-5	C24H22FNO	Pharmaceutical (Schedule I Drug of Abuse)

Table S20. Compound database for suspect screening (continued)

Compound Name	CAS	Molecular Formula	Category
1-(2-(4-Morpholinyl)ethyl)-3-(1-naphthoyl)indole (JWH-200)	103610-04-4	C25H24N2O2	Pharmaceutical (Schedule I Drug of Abuse)
1-(2-Cyclohexylethyl)-3-(2-methoxyphenylacetyl)indole (RCS-8)	1345970-42-4	C25H29NO2	Pharmaceutical (Schedule I Drug of Abuse)
1-[(5-Fluoropentyl)-1H-indol-3-yl]-(2-iodophenyl)methanone (AM-694)	335161-03-0	C20H19FINO	Pharmaceutical (Schedule I Drug of Abuse)
1-Butyl-3-(1-naphthoyl)indole (JWH-073)	208987-48-8	C23H21NO	Pharmaceutical (Schedule I Drug of Abuse)
1-Hexyl-3-(1-naphthoyl)indole (JWH-019)	209414-08-4	C25H25NO	Pharmaceutical (Schedule I Drug of Abuse)
1-Pentyl-3-(1-naphthoyl)indole (JWH-018)	209414-07-3	C24H23NO	Pharmaceutical (Schedule I Drug of Abuse)
1-Pentyl-3-(2-chlorophenylacetyl)indole (JWH-203)	864445-54-5	C21H22ClNO	Pharmaceutical (Schedule I Drug of Abuse)
1-Pentyl-3-(2-methoxyphenylacetyl)indole (JWH-250)	864445-43-2	C22H25NO2	Pharmaceutical (Schedule I Drug of Abuse)
1-Pentyl-3-(4-chloro-1-naphthoyl)indole (JWH-398)	1292765-18-4	C24H22ClNO	Pharmaceutical (Schedule I Drug of Abuse)
1-Pentyl-3-(4-methoxybenzoyl)indole (RCS-4)	1345966-78-0	C21H23NO2	Pharmaceutical (Schedule I Drug of Abuse)
1-Pentyl-3-(4-methoxynaphthoyl)indole (JWH-081)	210179-46-7	C25H25NO2	Pharmaceutical (Schedule I Drug of Abuse)
1-Pentyl-3-(4-methyl-1-naphthoyl)indole (JWH-122)	619294-47-2	C25H25NO	Pharmaceutical (Schedule I Drug of Abuse)
2-(2,5-Dimethoxy-4-propylphenyl)ethanamine (2C-P)	207740-22-5	C13H21NO2	Pharmaceutical (Schedule I Drug of Abuse)
2-(4-(Isopropylthio)-2,5-dimethoxyphenyl)ethanamine (2C-T-4)	207740-25-8	C13H21NO2S	Pharmaceutical (Schedule I Drug of Abuse)
2-(4-Bromo-2,5-dimethoxyphenyl)-N-(2-methoxybenzyl)ethanamine (25B-NBOMe)	1026511-90-9	C18H22BrNO3	Pharmaceutical (Schedule I Drug of Abuse)
2-(4-Chloro-2,5-dimethoxyphenyl)-N-(2-methoxybenzyl)ethanamine (25C-NBOMe)	1227608-02-7	C18H22ClNO3	Pharmaceutical (Schedule I Drug of Abuse)
2-(4-Iodo-2,5-dimethoxyphenyl)-N-((2-methoxyphenyl)methyl)ethanamine (25I-NBOMe)	919797-19-6	C18H22INO3	Pharmaceutical (Schedule I Drug of Abuse)
2,5-Dimethoxy-4-(ethylthio)phenethylamine (2C-T-2)	207740-24-7	C12H19NO2S	Pharmaceutical (Schedule I Drug of Abuse)
2,5-Dimethoxy-4-(n)-propylthiophenethylamine (2C-T-7)	207740-26-9	C13H21NO2S	Pharmaceutical (Schedule I Drug of Abuse)
2,5-Dimethoxy-4-chlorophenethylamine (2C-C)	88441-14-9	C10H14ClNO2	Pharmaceutical (Schedule I Drug of Abuse)
2,5-Dimethoxy-4-ethylamphetamine (DOET)	22004-32-6	C13H21NO2	Pharmaceutical (Schedule I Drug of Abuse)
2,5-Dimethoxy-4-ethylphenethylamine (2C-E)	71539-34-9	C12H19NO2	Pharmaceutical (Schedule I Drug of Abuse)
2,5-Dimethoxy-4-methylamphetamine (DOM)	15588-95-1	C12H19NO2	Pharmaceutical (Schedule I Drug of Abuse)
2,5-Dimethoxy-4-methylphenethylamine (2C-D)	24333-19-5	C11H17NO2	Pharmaceutical (Schedule I Drug of Abuse)
2,5-Dimethoxy-4-nitrophenethylamine (2C-N)	261789-00-8	C10H14N2O4	Pharmaceutical (Schedule I Drug of Abuse)
2,5-Dimethoxyamphetamine (2,5-DMA)	2801-68-5	C11H17NO2	Pharmaceutical (Schedule I Drug of Abuse)
2,5-Dimethoxyphenethylamine (2C-H)	3600-86-0	C10H15NO2	Pharmaceutical (Schedule I Drug of Abuse)
3,4,5-Trimethoxyamphetamine (TMA)	1082-88-8	C12H19NO3	Pharmaceutical (Schedule I Drug of Abuse)
3,4-Dichloro-N-(1-(dimethylamino)cyclohexyl)methylbenzamide (AH-7921)	55154-30-8	C16H22Cl2N2O	Pharmaceutical (Schedule I Drug of Abuse)
3,4-Dichloro-N-(2-(dimethylamino)cyclohexyl)-N-methylbenzamide (U-47700)	82657-23-6	C16H22Cl2N2O	Pharmaceutical (Schedule I Drug of Abuse)
3,4-Methylenedioxyamphetamine (3,4-MDA)	4764-17-4	C10H13NO2	Pharmaceutical (Schedule I Drug of Abuse)
3,4-Methylenedioxymethamphetamine (3,4-MDMA)	42542-10-9	C11H15NO2	Pharmaceutical (Schedule I Drug of Abuse)
3,4-Methylenedioxy-N-ethylamphetamine (3,4-MDEA)	82801-81-8	C12H17NO2	Pharmaceutical (Schedule I Drug of Abuse)
3,4-Methylenedioxy-N-methylcathinone (Methylone)	186028-79-5	C11H13NO3	Pharmaceutical (Schedule I Drug of Abuse)
3,4-Methylenedioxypyrovalerone (MDPV)	687603-66-3	C16H21NO3	Pharmaceutical (Schedule I Drug of Abuse)
3-Fluoromethcathinone (3-FMC)	1049677-77-1	C10H12FNO	Pharmaceutical (Schedule I Drug of Abuse)
3-Methylfentanyl	42045-86-3	C23H30N2O	Pharmaceutical (Schedule I Drug of Abuse)
3-Methylthiofentanyl	86052-04-2	C21H28N2OS	Pharmaceutical (Schedule I Drug of Abuse)
4-Bromo-2,5-dimethoxyamphetamine (DOB)	64638-07-9	C11H16BrNO2	Pharmaceutical (Schedule I Drug of Abuse)
4-Bromo-2,5-dimethoxyphenethylamine (2C-B)	66142-81-2	C10H14BrNO2	Pharmaceutical (Schedule I Drug of Abuse)
4-Fluoromethcathinone (4-FMC)	447-40-5	C10H12FNO	Pharmaceutical (Schedule I Drug of Abuse)
4-Iodo-2,5-dimethoxyphenethylamine (2C-I)	69587-11-7	C10H14INO2	Pharmaceutical (Schedule I Drug of Abuse)
4-Methoxyamphetamine (PMA)	64-13-1	C10H15NO	Pharmaceutical (Schedule I Drug of Abuse)
4'-Methyl-alpha-pyrrolidinopropiophenone (4-MePPP)	28117-80-8	C14H19NO	Pharmaceutical (Schedule I Drug of Abuse)
4-Methylaminorex (McN-822)	3568-94-3	C10H12N2O	Pharmaceutical (Schedule I Drug of Abuse)
4-Methylethcathinone (4-MEC)	1225617-18-4	C12H17NO	Pharmaceutical (Schedule I Drug of Abuse)
5-Methoxy-3,4-methylenedioxyamphetamine (MMDA)	13674-05-0	C11H15NO3	Pharmaceutical (Schedule I Drug of Abuse)
5-Methoxy-N,N-diisopropyltryptamine (5-MeO-DIPT)	4021-34-5	C17H26N2O	Pharmaceutical (Schedule I Drug of Abuse)
Acetylfentanyl	3258-84-2	C21H26N2O	Pharmaceutical (Schedule I Drug of Abuse)
alpha-Ethyltryptamine	2235-90-7	C12H16N2	Pharmaceutical (Schedule I Drug of Abuse)
alpha-Methylacetylfentanyl	101860-00-8	C22H28N2O	Pharmaceutical (Schedule I Drug of Abuse)
alpha-Methylfentanyl	79704-88-4	C23H30N2O	Pharmaceutical (Schedule I Drug of Abuse)
alpha-Methylthiofentanyl	103963-66-2	C21H28N2OS	Pharmaceutical (Schedule I Drug of Abuse)
alpha-Methyltryptamine	299-26-3	C11H14N2	Pharmaceutical (Schedule I Drug of Abuse)
alpha-Pyrrolidinobutiophenone (α-PBP)	13415-54-8	C14H19NO	Pharmaceutical (Schedule I Drug of Abuse)
alpha-Pyrrolidinopentiophenone	14530-33-7	C15H21NO	Pharmaceutical (Schedule I Drug of Abuse)
Benzylmorphine	14297-87-1	C24H25NO3	Pharmaceutical (Schedule I Drug of Abuse)
beta-Hydroxyfentanyl	78995-10-5	C22H28N2O2	Pharmaceutical (Schedule I Drug of Abuse)
beta-Hydroxythiofentanyl	1474-34-6	C20H26N2O2S	Pharmaceutical (Schedule I Drug of Abuse)
Bufotenine	487-93-4	C12H16N2O	Pharmaceutical (Schedule I Drug of Abuse)
Butylone	802575-11-7	C12H15NO3	Pharmaceutical (Schedule I Drug of Abuse)
Butyrfentanyl	1169-70-6	C23H30N2O	Pharmaceutical (Schedule I Drug of Abuse)
Cannabinol	521-35-7	C21H26O2	Pharmaceutical (Schedule I Drug of Abuse)
Cathinone	71031-15-7	C9H11NO	Pharmaceutical (Schedule I Drug of Abuse)
Clonitazene	3861-76-5	C20H23ClN4O2	Pharmaceutical (Schedule I Drug of Abuse)
Desomorphine	427-00-9	C17H21NO2	Pharmaceutical (Schedule I Drug of Abuse)
Diampromide	552-25-0	C21H28N2O	Pharmaceutical (Schedule I Drug of Abuse)

Table S20. Compound database for suspect screening (continued)

Compound Name	CAS	Molecular Formula	Category
Dihydromorphine	509-60-4	C17H21NO3	Pharmaceutical (Schedule I Drug of Abuse)
Etonitazene	911-65-9	C22H28N4O3	Pharmaceutical (Schedule I Drug of Abuse)
Furanylfentanyl	101345-66-8	C24H26N2O2	Pharmaceutical (Schedule I Drug of Abuse)
Hydromorphinol	2183-56-4	C17H21NO4	Pharmaceutical (Schedule I Drug of Abuse)
Levophenacylmorphan	10061-32-2	C24H27NO2	Pharmaceutical (Schedule I Drug of Abuse)
Lysergide (LSD)	50-37-3	C20H25N3O	Pharmaceutical (Schedule I Drug of Abuse)
Mephedrone	1189805-46-6	C11H15NO	Pharmaceutical (Schedule I Drug of Abuse)
Mescaline	54-04-6	C11H17NO3	Pharmaceutical (Schedule I Drug of Abuse)
Methadol (Dimepheptanol)	545-90-4	C21H29NO	Pharmaceutical (Schedule I Drug of Abuse)
Methcathinone	5650-44-2	C10H13NO	Pharmaceutical (Schedule I Drug of Abuse)
Methyldihydromorphine	509-56-8	C18H23NO3	Pharmaceutical (Schedule I Drug of Abuse)
Moramide	545-59-5	C25H32N2O2	Pharmaceutical (Schedule I Drug of Abuse)
N-(1-(Aminocarbonyl)-2-methylpropyl)-1-(cyclohexylmethyl)-1H-indazole-3-carboxamide	1185887-21-1	C20H28N4O2	Pharmaceutical (Schedule I Drug of Abuse)
N-(1-Amino-3,3-dimethyl-1-oxobutan-2-yl)-1-(cyclohexylmethyl)-1H-indazole-3-carboxamide	1863065-92-2	C21H30N4O2	Pharmaceutical (Schedule I Drug of Abuse)
N-(1-Amino-3,3-dimethyl-1-oxobutan-2-yl)-1-pentyl-1H-indazole-3-carboxamide (ADB-PINACA)	1633766-73-0	C19H28N4O2	Pharmaceutical (Schedule I Drug of Abuse)
N-(1-Amino-3-methyl-1-oxobutan-2-yl)-1-(4-fluorobenzyl)-1H-indazole-3-carboxamide	1185282-01-2	C20H21FN4O2	Pharmaceutical (Schedule I Drug of Abuse)
N-(1-Amino-3-methyl-1-oxobutan-2-yl)-1-pentyl-1H-indazole-3-carboxamide (AB-PINACA)	1445752-09-9	C18H26N4O2	Pharmaceutical (Schedule I Drug of Abuse)
N,N-Diethyltryptamine (DET)	61-51-8	C14H20N2	Pharmaceutical (Schedule I Drug of Abuse)
N,N-Dimethyl-5-methoxytryptamine (5-MeO-DMT)	1019-45-0	C13H18N2O	Pharmaceutical (Schedule I Drug of Abuse)
N,N-Dimethylamphetamine	17279-39-9	C11H17N	Pharmaceutical (Schedule I Drug of Abuse)
N,N-Dimethyltryptamine (DMT)	61-50-7	C12H16N2	Pharmaceutical (Schedule I Drug of Abuse)
Naphyrone	850352-11-3	C19H23NO	Pharmaceutical (Schedule I Drug of Abuse)
N-Benzylpiperazine (BZP)	2759-28-6	C11H16N2	Pharmaceutical (Schedule I Drug of Abuse)
N-Ethyl-1-phenylcyclohexylamine (PCE)	2201-15-2	C14H21N	Pharmaceutical (Schedule I Drug of Abuse)
N-Ethylamphetamine (NEA)	33817-11-7	C11H17N	Pharmaceutical (Schedule I Drug of Abuse)
N-Hydroxy-3,4-methylenedioxyamphetamine (N-Hydroxy MDA)	74698-47-8	C10H13NO3	Pharmaceutical (Schedule I Drug of Abuse)
Normorphine	466-97-7	C16H17NO3	Pharmaceutical (Schedule I Drug of Abuse)
Norpipanone	561-48-8	C23H29NO	Pharmaceutical (Schedule I Drug of Abuse)
Ohmefentanyl	78995-14-9	C23H30N2O2	Pharmaceutical (Schedule I Drug of Abuse)
Pentedrone	879722-57-3	C12H17NO	Pharmaceutical (Schedule I Drug of Abuse)
Pentylone	698963-77-8	C13H17NO3	Pharmaceutical (Schedule I Drug of Abuse)
p-Fluorofentanyl	90736-23-5	C22H27FN2O	Pharmaceutical (Schedule I Drug of Abuse)
Phenadoxone	467-84-5	C23H29NO2	Pharmaceutical (Schedule I Drug of Abuse)
Phenampromide	129-83-9	C17H26N2O	Pharmaceutical (Schedule I Drug of Abuse)
Phenomorphan	468-07-5	C24H29NO	Pharmaceutical (Schedule I Drug of Abuse)
Proheptazine	77-14-5	C17H25NO2	Pharmaceutical (Schedule I Drug of Abuse)
Properidine	561-76-2	C16H23NO2	Pharmaceutical (Schedule I Drug of Abuse)
Psilocybin	520-52-5	C12H17N2O4P	Pharmaceutical (Schedule I Drug of Abuse)
Quinolin-8-yl 1-(5-fluoropentyl)-1H-indole-3-carboxylate (5F-PB-22)	1400742-41-7	C23H21FN2O2	Pharmaceutical (Schedule I Drug of Abuse)
Quinolin-8-yl 1-pentyl-1H-indole-3-carboxylate (PB-22)	1400742-17-7	C23H22N2O2	Pharmaceutical (Schedule I Drug of Abuse)
Rolicyclidine	2201-39-0	C16H23N	Pharmaceutical (Schedule I Drug of Abuse)
Tenocyclidine	1867-65-8	C15H23NS	Pharmaceutical (Schedule I Drug of Abuse)
Thiofentanyl	1165-22-6	C20H26N2OS	Pharmaceutical (Schedule I Drug of Abuse)
Valerylfentanyl	122882-90-0	C24H32N2O	Pharmaceutical (Schedule I Drug of Abuse)
1-Phenylcyclohexylamine	2201-24-3	C12H17N	Pharmaceutical (Schedule II Drug of Abuse)
1-Piperidinocyclohexanecarbonitrile	3867-15-0	C12H20N2	Pharmaceutical (Schedule II Drug of Abuse)
4-Anilino-N-phenethyl-piperidine (ANPP)	21409-26-7	C19H24N2	Pharmaceutical (Schedule II Drug of Abuse)
Alphaprodine	77-20-3	C16H23NO2	Pharmaceutical (Schedule II Drug of Abuse)
Betaprodine	468-59-7	C16H23NO2	Pharmaceutical (Schedule II Drug of Abuse)
Ecgonine	481-37-8	C9H15NO3	Pharmaceutical (Schedule II Drug of Abuse)
Ethylphenidate	57413-43-1	C15H21NO2	Pharmaceutical (Schedule II Drug of Abuse)
Oripavine	467-04-9	C18H19NO3	Pharmaceutical (Schedule II Drug of Abuse)
Phenazocine	127-35-5	C22H27NO	Pharmaceutical (Schedule II Drug of Abuse)
Phenylacetone	103-79-7	C9H10O	Pharmaceutical (Schedule II Drug of Abuse)
Piminodine	13495-09-5	C23H30N2O2	Pharmaceutical (Schedule II Drug of Abuse)
Thebaine	115-37-7	C19H21NO3	Pharmaceutical (Schedule II Drug of Abuse)
Thiofentanil	60771-38-2	C22H28N2O3S	Pharmaceutical (Schedule II Drug of Abuse)
1,3-Benzodioxolylbutanamine (BDB)	107447-03-0	C11H15NO2	Pharmaceutical (Schedule III Drug of Abuse)
17-Methyl-5alpha-androstane-3alpha,17beta-diol	641-82-7	C20H34O2	Pharmaceutical (Schedule III Drug of Abuse)
17-Methyl-5alpha-androstane-3beta,17beta-diol	641-83-8	C20H34O2	Pharmaceutical (Schedule III Drug of Abuse)
18-Methyl-19-nortestosterone	793-55-5	C19H28O2	Pharmaceutical (Schedule III Drug of Abuse)
19-Nor-5-androstene-3beta,17beta-diol	25975-59-1	C18H28O2	Pharmaceutical (Schedule III Drug of Abuse)
19-Norandrost-4-ene-3,17-dione	734-32-7	C18H24O2	Pharmaceutical (Schedule III Drug of Abuse)
4-Androsten-3beta,17beta-diol (4-Androstenediol)	1156-92-9	C19H30O2	Pharmaceutical (Schedule III Drug of Abuse)
4-Chlorodehydromethyltestosterone	2446-23-3	C20H27ClO2	Pharmaceutical (Schedule III Drug of Abuse)
4-Hydroxy-17alpha-methyl-19-nortestosterone	2747-16-2	C19H28O3	Pharmaceutical (Schedule III Drug of Abuse)
4-Hydroxy-19-nortestosterone (Oxabolone)	4721-69-1	C18H26O3	Pharmaceutical (Schedule III Drug of Abuse)

Table S20. Compound database for suspect screening (continued)

Compound Name	CAS	Molecular Formula	Category
4-Hydroxytestosterone	2141-17-5	C19H28O3	Pharmaceutical (Schedule III Drug of Abuse)
5alpha-Androst-1-en-3,17-diol	5323-27-3	C19H30O2	Pharmaceutical (Schedule III Drug of Abuse)
5alpha-Androst-1-ene-3,17-dione (1-Androstenedione)	571-40-4	C19H26O2	Pharmaceutical (Schedule III Drug of Abuse)
5alpha-Androstane-3alpha,17beta-diol	1852-53-5	C19H32O2	Pharmaceutical (Schedule III Drug of Abuse)
5alpha-Androstane-3beta,17beta-diol	571-20-0	C19H32O2	Pharmaceutical (Schedule III Drug of Abuse)
5-Androsten-3,17-dione	571-36-8	C19H26O2	Pharmaceutical (Schedule III Drug of Abuse)
Androst-5-ene-3beta,17beta-diol (5-Androstenediol)	521-17-5	C19H30O2	Pharmaceutical (Schedule III Drug of Abuse)
Androsta-1,4-diene-3,17-dione (Boldione)	897-06-3	C19H24O2	Pharmaceutical (Schedule III Drug of Abuse)
Androstanedione	846-46-8	C19H28O2	Pharmaceutical (Schedule III Drug of Abuse)
Desoxymethyltestosterone (Madol)	3275-64-7	C20H32O	Pharmaceutical (Schedule III Drug of Abuse)
Formebolone	2454-11-7	C21H28O4	Pharmaceutical (Schedule III Drug of Abuse)
Lysergic Acid	82-58-6	C16H16N2O2	Pharmaceutical (Schedule III Drug of Abuse)
Methasterone	3381-88-2	C21H34O2	Pharmaceutical (Schedule III Drug of Abuse)
Methyl-1-testosterone	65-04-3	C20H30O2	Pharmaceutical (Schedule III Drug of Abuse)
Methyldienedione	5173-46-6	C18H22O2	Pharmaceutical (Schedule III Drug of Abuse)
Methyldienolone	14531-89-6	C19H26O2	Pharmaceutical (Schedule III Drug of Abuse)
Metribolone	965-93-5	C19H24O2	Pharmaceutical (Schedule III Drug of Abuse)
Norclostebol	13583-21-6	C18H25ClO2	Pharmaceutical (Schedule III Drug of Abuse)
Normethandrone	514-61-4	C19H28O2	Pharmaceutical (Schedule III Drug of Abuse)
Oxymesterone	145-12-0	C20H30O3	Pharmaceutical (Schedule III Drug of Abuse)
Prostanozol	1186001-41-1	C25H38N2O2	Pharmaceutical (Schedule III Drug of Abuse)
Tetrahydrogestrinone	618903-56-3	C21H28O2	Pharmaceutical (Schedule III Drug of Abuse)
Romifidine	65896-16-4	C9H9BrFN3	Pharmaceutical (Sedative (veterinary), alpha2-Adrenergic receptor agonist)
Acepromazine	61-00-7	C19H22N2OS	Pharmaceutical (Sedative-hypnotic (veterinary))
Amobarbital	57-43-2	C11H18N2O3	Pharmaceutical (Sedative-hypnotic)
Aprobarbital	77-02-1	C10H14N2O3	Pharmaceutical (Sedative-hypnotic)
Barbital	57-44-3	C8H12N2O3	Pharmaceutical (Sedative-hypnotic)
Butabarbital	125-40-6	C10H16N2O3	Pharmaceutical (Sedative-hypnotic)
Butalbital	77-26-9	C11H16N2O3	Pharmaceutical (Sedative-hypnotic)
Butobarbital	77-28-1	C10H16N2O3	Pharmaceutical (Sedative-hypnotic)
Estazolam	29975-16-4	C16H11ClN4	Pharmaceutical (Sedative-hypnotic)
Eszopiclone	138729-47-2	C17H17ClN6O3	Pharmaceutical (Sedative-hypnotic)
Ethchlorvynol	113-18-8	C7H9ClO	Pharmaceutical (Sedative-hypnotic)
Ethinamate	126-52-3	C9H13NO2	Pharmaceutical (Sedative-hypnotic)
Etomidate	33125-97-2	C14H16N2O2	Pharmaceutical (Sedative-hypnotic)
Flurazepam	17617-23-1	C21H23ClFN3O	Pharmaceutical (Sedative-hypnotic)
Glutethimide	77-21-4	C13H15NO2	Pharmaceutical (Sedative-hypnotic)
Halazepam	23092-17-3	C17H12ClF3N2O	Pharmaceutical (Sedative-hypnotic)
Haloxazolam	59128-97-1	C17H14BrFN2O2	Pharmaceutical (Sedative-hypnotic)
Loprazolam	61197-73-7	C23H21ClN6O3	Pharmaceutical (Sedative-hypnotic)
Lormetazepam	848-75-9	C16H12Cl2N2O2	Pharmaceutical (Sedative-hypnotic)
Mecloqualone	340-57-8	C15H11ClN2O	Pharmaceutical (Sedative-hypnotic)
Methaqualone	72-44-6	C16H14N2O	Pharmaceutical (Sedative-hypnotic)
Methyprylon	125-64-4	C10H17NO2	Pharmaceutical (Sedative-hypnotic)
Nimetazepam	2011-67-8	C16H13N3O3	Pharmaceutical (Sedative-hypnotic)
Pentobarbital	76-74-4	C11H18N2O3	Pharmaceutical (Sedative-hypnotic)
Prazepam	2955-38-6	C19H17ClN2O	Pharmaceutical (Sedative-hypnotic)
Quazepam	36735-22-5	C17H11ClF4N2S	Pharmaceutical (Sedative-hypnotic)
Secobarbital	76-73-3	C12H18N2O3	Pharmaceutical (Sedative-hypnotic)
Talbutal	115-44-6	C11H16N2O3	Pharmaceutical (Sedative-hypnotic)
Triazolam	28911-01-5	C17H12Cl2N4	Pharmaceutical (Sedative-hypnotic)
Zaleplon	151319-34-5	C17H15N5O	Pharmaceutical (Sedative-hypnotic)
Zolazepam	31352-82-6	C15H15FN4O	Pharmaceutical (Sedative-hypnotic)
Zolpidem	82626-48-0	C19H21N3O	Pharmaceutical (Sedative-hypnotic)
Zopiclone	43200-80-2	C17H17ClN6O3	Pharmaceutical (Sedative-hypnotic)
Dexmedetomidine	113775-47-6	C13H16N2	Pharmaceutical (Sedative-hypnotic, Tranquilizer, alpha2-Adrenergic receptor agonist)
Carisoprodol	78-44-4	C12H24N2O4	Pharmaceutical (Skeletal muscle relaxant)
Chlorphenesin	104-29-0	C9H11ClO3	Pharmaceutical (Skeletal muscle relaxant)
Chlorzoxazone	95-25-0	C7H4ClNO2	Pharmaceutical (Skeletal muscle relaxant)
Dantrolene	7261-97-4	C14H10N4O5	Pharmaceutical (Skeletal muscle relaxant)
Metaxalone	1665-48-1	C12H15NO3	Pharmaceutical (Skeletal muscle relaxant)
Methocarbamol	532-03-6	C11H15NO5	Pharmaceutical (Skeletal muscle relaxant)
Orphenadrine	83-98-7	C18H23NO	Pharmaceutical (Skeletal muscle relaxant, Muscarinic acetylcholine receptor antagonist, H1 receptor antagonist)
Nicotine	54-11-5	C10H14N2	Pharmaceutical (Smoking cessation adjunct)
Varenicline	249296-44-4	C13H13N3	Pharmaceutical (Smoking cessation adjunct, Selective nicotinic acetylcholine receptor modulator)
Amphetamine	300-62-9	C9H13N	Pharmaceutical (Stimulant (central))
Dexmethylphenidate	40431-64-9	C14H19NO2	Pharmaceutical (Stimulant (central))

Table S20. Compound database for suspect screening (continued)

Compound Name	CAS	Molecular Formula	Category
Dextroamphetamine	51-64-9	C9H13N	Pharmaceutical (Stimulant (central))
Fencamfamin	1209-98-9	C15H21N	Pharmaceutical (Stimulant (central))
Fenethylline	3736-08-1	C18H23N5O2	Pharmaceutical (Stimulant (central))
Lisdexamfetamine	608137-32-2	C15H25N3O	Pharmaceutical (Stimulant (central))
Methylphenidate	113-45-1	C14H19NO2	Pharmaceutical (Stimulant (central))
Modafinil	68693-11-8	C15H15NO2S	Pharmaceutical (Stimulant (central))
Pipradrol	467-60-7	C18H21NO	Pharmaceutical (Stimulant (central))
Caffeine	58-08-2	C8H10N4O2	Pharmaceutical (Stimulant (central), Adenosine receptor antagonist, Phosphodiesterase inhibitor)
Pyrovalerone	3563-49-3	C16H23NO	Pharmaceutical (Stimulant (central), Noradrenaline and dopamine reuptake inhibitor)
Atomoxetine	83015-26-3	C17H21NO	Pharmaceutical (Stimulant (central), Selective noradrenaline reuptake inhibitor (NRI))
Dexpanthenol	81-13-0	C9H19NO4	Pharmaceutical (Supplement (pantothenic acid), Muscarinic acetylcholine receptor agonist)
Pyridoxine	65-23-6	C8H11NO3	Pharmaceutical (Supplement (vitamin B6))
Anagrelide	68475-42-3	C10H7Cl2N3O	Pharmaceutical (Thrombocythemia treatment, Platelet aggregation inhibitor, Phosphodiesterase inhibitor)
Diethylstilbestrol	56-53-1	C18H20O2	Pharmaceutical (Tocolytic, Estrogen receptor agonist)
Trazodone	19794-93-5	C19H22ClN5O	Pharmaceutical (Tranquilizer, Antidepressant, Serotonin antagonist and reuptake inhibitor (SARI))
Hydroxyzine	68-88-2	C21H27ClN2O2	Pharmaceutical (Tranquilizer, H1 receptor antagonist)
Pseudoephedrine	90-82-4	C10H15NO	Pharmaceutical (Vasoconstrictor, Adrenergic receptor agonist)
Naphazoline	835-31-4	C14H14N2	Pharmaceutical (Vasoconstrictor, alpha1-Adrenergic receptor agonist)
Oxymetazoline	1491-59-4	C16H24N2O	Pharmaceutical (Vasoconstrictor, alpha1-Adrenergic receptor agonist)
Phenylephrine	59-42-7	C9H13NO2	Pharmaceutical (Vasoconstrictor, alpha1-Adrenergic receptor agonist)
Tetryzoline	84-22-0	C13H16N2	Pharmaceutical (Vasoconstrictor, alpha1-Adrenergic receptor agonist)
Regadenoson	313348-27-5	C15H18N8O5	Pharmaceutical (Vasodilator (coronary), Adenosine A2a receptor agonist)
Dipyridamole	58-32-2	C24H40N8O4	Pharmaceutical (Vasodilator (coronary), Platelet aggregation inhibitor, Phosphodiesterase inhibitor)
Bamethan	3703-79-5	C12H19NO2	Pharmaceutical (Vasodilator (peripheral))
Tolazoline	59-98-3	C10H12N2	Pharmaceutical (Vasodilator (peripheral), alpha-Adrenergic receptor antagonist)
Pentoxifylline	6493-05-6	C13H18N4O3	Pharmaceutical (Vasodilator)
Ranolazine	95635-55-5	C24H33N3O4	Pharmaceutical (Vasodilator)
Phentolamine	50-60-2	C17H19N3O	Pharmaceutical (Vasodilator, alpha-Adrenergic receptor antagonist)
Riociguat	625115-55-1	C20H19FN8O2	Pharmaceutical (Vasodilator, Guanylate cyclase activator)
Sildenafil	139755-83-2	C22H30N6O4S	Pharmaceutical (Vasodilator, Impotence therapy, Phosphodiesterase V inhibitor)
Vardenafil	224785-90-4	C23H32N6O4S	Pharmaceutical (Vasodilator, Impotence therapy, Phosphodiesterase V inhibitor)
Cilostazol	73963-72-1	C20H27N5O2	Pharmaceutical (Vasodilator, Platelet aggregation inhibitor, Phosphodiesterase inhibitor)
Alprostadil	745-65-3	C20H34O5	Pharmaceutical (Vasodilator, Prostaglandin E1 receptor agonist)
Abacavir Carboxylate	384380-52-3	C14H16N6O2	Pharmaceutical TP (Abacavir)
Descyclopropyl Abacavir	118237-88-0	C11H14N6O	Pharmaceutical TP (Abacavir)
4'-Hydroxy Aceclofenac	229308-90-1	C16H13Cl2NO5	Pharmaceutical TP (Aceclofenac)
6-Hydroxy Acenocoumarol	64180-13-8	C19H15NO7	Pharmaceutical TP (Acenocoumarol)
7-Hydroxy Acenocoumarol	64180-12-7	C19H15NO7	Pharmaceutical TP (Acenocoumarol)
3-Hydroxy Acetaminophen	37519-14-5	C8H9NO3	Pharmaceutical TP (Acetaminophen (Paracetamol))
3-Methoxy Acetaminophen	3251-55-6	C9H11NO3	Pharmaceutical TP (Acetaminophen (Paracetamol))
N-(4-Methoxyphenyl)acetamide	51-66-1	C9H11NO2	Pharmaceutical TP (Acetaminophen (Paracetamol))
N-Acetyl-4-benzoquinone Imine (NAPQI)	50700-49-7	C8H7NO2	Pharmaceutical TP (Acetaminophen (Paracetamol))
S-Methyl-3-thio Acetaminophen	37398-23-5	C9H11NO2S	Pharmaceutical TP (Acetaminophen (Paracetamol))
Acyclovir Carboxylic Acid (Carboxy-acyclovir)	80685-22-9	C8H9N5O4	Pharmaceutical TP (Acyclovir)
Albendazole Sulfone	75184-71-3	C12H15N3O4S	Pharmaceutical TP (Albendazole)
Albendazole Sulfoxide	54029-12-8	C12H15N3O3S	Pharmaceutical TP (Albendazole)
Albendazole-2-aminosulfone	80983-34-2	C10H13N3O2S	Pharmaceutical TP (Albendazole)
Alcaftadine 3-Carboxylic Acid	147083-93-0	C19H21N3O2	Pharmaceutical TP (Alcaftadine)
Alcaftadine N-Oxide	952649-75-1	C19H21N3O2	Pharmaceutical TP (Alcaftadine)
3′-Desmethoxy Aliskiren 3′-Carboxylic Acid	949925-75-1	C29H49N3O7	Pharmaceutical TP (Aliskiren)
Oxypurinol	2465-59-0	C5H4N4O2	Pharmaceutical TP (Allopurinol)
Almotriptan N-Oxide	603137-43-5	C17H25N3O3S	Pharmaceutical TP (Almotriptan)
Didesmethyl Almotriptan	181178-24-5	C15H21N3O2S	Pharmaceutical TP (Almotriptan)
gamma-Aminobutyric Acid Almotriptan	603137-41-3	C17H25N3O4S	Pharmaceutical TP (Almotriptan)
N-Desmethyl Alosetron	122852-63-5	C16H16N4O	Pharmaceutical TP (Alosetron)
1-Hydroxy Alprazolam	37115-43-8	C17H13ClN4O	Pharmaceutical TP (Alprazolam)
Alprazolam 5-Oxide	30896-65-2	C17H13ClN4O	Pharmaceutical TP (Alprazolam)
4-Acetamido Antipyrine	83-15-8	C13H15N3O2	Pharmaceutical TP (Aminopyrine)
4-Amino Antipyrine	83-07-8	C11H13N3O	Pharmaceutical TP (Aminopyrine)
4-Formylamino Antipyrine	1672-58-8	C12H13N3O2	Pharmaceutical TP (Aminopyrine)
Dioxyaminopyrine	519-65-3	C13H17N3O3	Pharmaceutical TP (Aminopyrine)
Noramidopyrine	519-98-2	C12H15N3O	Pharmaceutical TP (Aminopyrine)
Di-N-desethyl Amiodarone	94317-95-0	C21H21I2NO3	Pharmaceutical TP (Amiodarone)
10-Hydroxy Amitriptyline	64520-05-4	C20H23NO	Pharmaceutical TP (Amitriptyline)
Amitriptyline N-Oxide	4317-14-0	C20H23NO	Pharmaceutical TP (Amitriptyline)
O-Des[2-aminoethyl]-O-carboxymethyl-dehydroamlodipine	113994-45-9	C20H20ClNO7	Pharmaceutical TP (Amlodipine)
5-[(7-Chloro-4-quinolinyl)amino]-2-hydroxybenzaldehyde	172476-18-5	C16H11ClN2O2	Pharmaceutical TP (Amodiaquine)

Table S20. Compound database for suspect screening (continued)

Compound Name	CAS	Molecular Formula	Category
Desethyl Amodiaquine	79352-78-6	C18H18ClN3O	Pharmaceutical TP (Amodiaquine)
7-Hydroxy Amoxapine	37081-76-8	C17H16ClN3O2	Pharmaceutical TP (Amoxapine)
8-Hydroxy Amoxapine	61443-78-5	C17H16ClN3O2	Pharmaceutical TP (Amoxapine)
2-Amino-5,6-dichloro-3,4-dihydroquinazoline	444904-63-6	C8H7Cl2N3	Pharmaceutical TP (Anagrelide)
3-Hydroxy Anagrelide	733043-41-9	C10H7Cl2N3O2	Pharmaceutical TP (Anagrelide)
4-Hydroxy Antipyrine	1672-63-5	C11H12N2O2	Pharmaceutical TP (Antipyrine)
O-Demethyl Apixaban	503612-76-8	C24H23N5O4	Pharmaceutical TP (Apixaban)
(2R,3S)-2-((1R)-1-(3,5-Bis(trifluoromethyl)phenyl)ethoxy)-3-(4-fluorophenyl) Morpholine	171338-27-5	C20H18F7NO2	Pharmaceutical TP (Aprepitant)
Aprepitant N-Oxide	172673-23-3	C23H21F7N4O4	Pharmaceutical TP (Aprepitant)
Aripiprazole N,N-Dioxide	573691-13-1	C23H27Cl2N3O4	Pharmaceutical TP (Aripiprazole)
Aripiprazole N-Oxide	573691-09-5	C23H27Cl2N3O3	Pharmaceutical TP (Aripiprazole)
Dehydro Aripiprazole	129722-25-4	C23H25Cl2N3O2	Pharmaceutical TP (Aripiprazole)
Asenapine N-Oxide	128949-51-9	C17H16ClNO2	Pharmaceutical TP (Asenapine)
N-Desmethyl Asenapine	128915-56-0	C16H14ClNO	Pharmaceutical TP (Asenapine)
Salicylic Acid	69-72-7	C7H6O3	Pharmaceutical TP (Aspirin (Acetylsalicylic Acid))
4-(2-Pyridyl)benzaldehyde	127406-56-8	C12H9NO	Pharmaceutical TP (Atazanavir)
Des(benzylpyridyl) Atazanavir	1192224-24-0	C26H43N5O7	Pharmaceutical TP (Atazanavir)
Hydroxy Atenolol	68373-10-4	C14H22N2O4	Pharmaceutical TP (Atenolol)
N-Deisopropyl Atenolol	81346-71-6	C11H16N2O3	Pharmaceutical TP (Atenolol)
4-Hydroxy Atomoxetine	435293-66-6	C17H21NO2	Pharmaceutical TP (Atomoxetine)
Desmethyl Atomoxetine	109306-10-7	C16H19NO	Pharmaceutical TP (Atomoxetine)
2-Hydroxy Atorvastatin	214217-86-4	C33H35FN2O6	Pharmaceutical TP (Atorvastatin)
4-Hydroxy Atorvastatin	214217-88-6	C33H35FN2O6	Pharmaceutical TP (Atorvastatin)
Desfluoro Atorvastatin	433289-84-0	C33H36N2O5	Pharmaceutical TP (Atorvastatin)
Deschloro Atovaquone	92458-44-1	C22H20O3	Pharmaceutical TP (Atovaquone)
Nortropine	538-09-0	C7H13NO	Pharmaceutical TP (Atropine)
2-Thiouric Acid	15986-31-9	C5H4N4O2S	Pharmaceutical TP (Azathioprine)
5-Amino-1-methyl-4-nitroimidazole	4531-54-8	C4H6N4O2	Pharmaceutical TP (Azathioprine)
5-Hydroxy-1-methyl-4-nitroimidazole	73703-74-9	C4H5N3O3	Pharmaceutical TP (Azathioprine)
Azelastine N-Oxide	640279-88-5	C22H24ClN3O2	Pharmaceutical TP (Azelastine)
N-Desmethyl Azelastine	47491-38-3	C21H22ClN3O	Pharmaceutical TP (Azelastine)
N',N'-Di(desmethyl) Azithromycin	612069-27-9	C36H68N2O12	Pharmaceutical TP (Azithromycin)
Desulfated Aztreonam	102579-59-9	C13H17N5O5S	Pharmaceutical TP (Aztreonam)
Benazeprilat	86541-78-8	C22H24N2O5	Pharmaceutical TP (Benazepril)
N-Desmethyl Bendamustine	41515-13-3	C15H19Cl2N3O2	Pharmaceutical TP (Bendamustine)
Benzydamine N-Oxide	36504-71-9	C19H23N3O2	Pharmaceutical TP (Benzydamine)
Demethyl Benzydamine	32852-16-7	C18H21N3O	Pharmaceutical TP (Benzydamine)
7-Oxo Bexarotene	368451-15-4	C24H26O3	Pharmaceutical TP (Bexarotene)
6-Hydroxy Bexarotene	368451-07-4	C24H28O3	Pharmaceutical TP (Bexarotene)
7-Hydroxy Bexarotene	368451-10-9	C24H28O3	Pharmaceutical TP (Bexarotene)
Norchlorcyclizine	440341-75-3	C10H10ClNO3	Pharmaceutical TP (Bezafibrate)
Desacetyl Bisacodyl	603-41-8	C18H15NO2	Pharmaceutical TP (Bisacodyl)
Methyl 4-(2-hydroxy-3-(isopropylamino)propoxy)benzoate	33947-97-6	C14H21NO4	Pharmaceutical TP (Bisoprolol)
Desmethyl Bosentan	253688-61-8	C26H27N5O6S	Pharmaceutical TP (Bosentan)
Hydroxy Bosentan	253688-60-7	C27H29N5O7S	Pharmaceutical TP (Bosentan)
Hydroxy Desmethyl Bosentan	253688-62-9	C26H27N5O7S	Pharmaceutical TP (Bosentan)
5-Bromoquinoxalin-6-amine	50358-63-9	C8H6BrN3	Pharmaceutical TP (Brimonidine)
3-Hydroxy Bromazepam	13132-73-5	C14H10BrN3O2	Pharmaceutical TP (Bromazepam)
2-Amino-3-(4-bromobenzoyl)benzoic Acid	241496-82-2	C14H10BrNO3	Pharmaceutical TP (Bromfenac)
6alpha-Hydroxy Budesonide	577777-51-6	C25H34O7	Pharmaceutical TP (Budesonide)
6beta-Hydroxy Budesonide	88411-77-2	C25H34O7	Pharmaceutical TP (Budesonide)
16alpha-Hydroxy Prednisolone	13951-70-7	C21H28O6	Pharmaceutical TP (Budesonide, Prednisolone)
Desbutyl Bumetanide	28328-54-3	C13H12N2O5S	Pharmaceutical TP (Bumetanide)
4-Hydroxy-N-desbutyl Bupivacaine	51989-48-1	C14H20N2O2	Pharmaceutical TP (Bupivacaine)
Bupivacaine N-Oxide	1346597-81-6	C18H28N2O2	Pharmaceutical TP (Bupivacaine)
N-Desbutyl Bupivacaine	15883-20-2	C14H20N2O	Pharmaceutical TP (Bupivacaine)
Buprenorphine N-Oxide	112242-17-8	C29H41NO5	Pharmaceutical TP (Buprenorphine)
Norbuprenorphine	78715-23-8	C25H35NO4	Pharmaceutical TP (Buprenorphine)
Dihydro Bupropion	119802-68-5	C13H20ClNO	Pharmaceutical TP (Bupropion)
Hydroxybupropion	92264-81-8	C13H18ClNO2	Pharmaceutical TP (Bupropion)
5-Hydroxy Buspirone	105496-33-1	C21H31N5O3	Pharmaceutical TP (Buspirone)
6,10-Dihydroxy Buspirone	658701-59-8	C21H31N5O4	Pharmaceutical TP (Buspirone)
6-Hydroxy Buspirone	125481-61-0	C21H31N5O3	Pharmaceutical TP (Buspirone)
Buspirone N-Oxide	220747-81-9	C21H31N5O3	Pharmaceutical TP (Buspirone)
Desethylcarbamoyl Cabergoline	85329-86-8	C23H32N4O	Pharmaceutical TP (Cabergoline)
1,7-Dimethyluric Acid	33868-03-0	C7H8N4O3	Pharmaceutical TP (Caffeine)
Paraxanthine (1,7-Dimethylxanthine)	611-59-6	C7H8N4O2	Pharmaceutical TP (Caffeine)

Table S20. Compound database for suspect screening (continued)

Compound Name	CAS	Molecular Formula	Category
1-Methyluric Acid	708-79-2	C6H6N4O3	Pharmaceutical TP (Caffeine, Theophylline)
1-Methylxanthine	6136-37-4	C6H6N4O2	Pharmaceutical TP (Caffeine, Theophylline)
5'-Deoxy-5-fluorocytidine	66335-38-4	C9H12FN3O4	Pharmaceutical TP (Capecitabine)
5'-Deoxy-2',3'-di-O-acetyl-5-fluorocytidine	161599-46-8	C13H16FN3O6	Pharmaceutical TP (Capecitabine, Flucytosine)
Vanillylamine	1196-92-5	C8H11NO2	Pharmaceutical TP (Capsaicin)
3-Methylquinoxaline-2-carboxylic Acid	74003-63-7	C10H8N2O2	Pharmaceutical TP (Carbadox)
Desoxy Carbadox	55456-55-8	C11H10N4O2	Pharmaceutical TP (Carbadox)
10-Methoxy Carbamazepine	28721-09-7	C16H14N2O2	Pharmaceutical TP (Carbamazepine)
2-Hydroxy Carbamazepine	68011-66-5	C15H12N2O2	Pharmaceutical TP (Carbamazepine)
3-Hydroxy Carbamazepine	68011-67-6	C15H12N2O2	Pharmaceutical TP (Carbamazepine)
Carbamazepine-10,11-epoxide	36507-30-9	C15H12N2O2	Pharmaceutical TP (Carbamazepine)
Iminostilbene	256-96-2	C14H11N	Pharmaceutical TP (Carbamazepine)
Dihydroxycarbazepine	35079-97-1	C15H14N2O3	Pharmaceutical TP (Carbamazepine)
10,11-Dihydro-10-hydroxy Carbamazepine	29331-92-8	C15H14N2O2	Pharmaceutical TP (Carbamazepine, Oxcarbazepine)
Acridine-9-carboxylic Acid	5336-90-3	C14H9NO2	Pharmaceutical TP (Carbamazepine, Oxcarbazepine)
Acridone	578-95-0	C13H9NO	Pharmaceutical TP (Carbamazepine, Oxcarbazepine)
Hydroxy Carisoprodol	3424-34-8	C12H24N2O5	Pharmaceutical TP (Carisoprodol)
5-Hydroxy-3,4-dihydro-2(1H)-quinolinone	30389-33-4	C9H9NO2	Pharmaceutical TP (Carteolol)
1-Hydroxy Carvedilol	146574-41-6	C24H26N2O5	Pharmaceutical TP (Carvedilol)
3-Hydroxy Carvedilol	146574-43-8	C24H26N2O6	Pharmaceutical TP (Carvedilol)
4'-Benzyloxy Carvedilol	887352-95-6	C31H32N2O5	Pharmaceutical TP (Carvedilol)
4'-Hydroxyphenyl Carvedilol	142227-49-4	C24H26N2O5	Pharmaceutical TP (Carvedilol)
5'-Benzyloxy Carvedilol	887353-00-6	C31H32N2O5	Pharmaceutical TP (Carvedilol)
5'-Hydroxyphenyl Carvedilol	142227-51-8	C24H26N2O5	Pharmaceutical TP (Carvedilol)
8-Hydroxy Carvedilol	159426-95-6	C24H26N2O5	Pharmaceutical TP (Carvedilol)
Decarbazolyl Carvedilol	10461-27-5	C12H19NO4	Pharmaceutical TP (Carvedilol)
Decarbazolyl Desmethyl Carvedilol	114849-42-2	C11H17NO4	Pharmaceutical TP (Carvedilol)
O-Desmethyl Carvedilol	72956-44-6	C23H24N2O4	Pharmaceutical TP (Carvedilol)
Cefadroxil Sulfoxide	182290-77-3	C16H17N3O6S	Pharmaceutical TP (Cefadroxil)
3-Desacetyl Cefotaxime	66340-28-1	C14H15N5O6S2	Pharmaceutical TP (Cefotaxime)
Desfuroyl Ceftiofur	120882-22-6	C14H15N5O5S3	Pharmaceutical TP (Ceftiofur)
Descarbamoyl Cefuroxime	56271-94-4	C15H15N3O7S	Pharmaceutical TP (Cefuroxime)
2,5-Dimethyl Celecoxib	457639-26-8	C18H16F3N3O2S	Pharmaceutical TP (Celecoxib)
4-Desmethyl-2-methyl Celecoxib	170569-99-0	C17H14F3N3O2S	Pharmaceutical TP (Celecoxib)
4'-Hydroxy Celecoxib	170571-00-3	C17H14F3N3O3S	Pharmaceutical TP (Celecoxib)
Celecoxib Carboxylic Acid	170571-01-4	C17H12F3N3O4S	Pharmaceutical TP (Celecoxib)
7-Amino Desacetoxy Cephalosporanic Acid (7-ADCA)	22252-43-3	C8H10N2O3S	Pharmaceutical TP (Cephalexin)
Desacetyl Cephapirin	38115-21-8	C15H15N3O5S2	Pharmaceutical TP (Cephapirin)
Cetirizine N-Oxide	1076199-80-8	C21H25ClN2O4	Pharmaceutical TP (Cetirizine)
1-(4-Chlorobenzhydryl)piperazine	303-26-4	C17H19ClN2	Pharmaceutical TP (Cetirizine, Meclizine, Chlorcyclizine)
4-Chlorobenzophenone	134-85-0	C13H9ClO	Pharmaceutical TP (Cetirizine, Meclizine, Chlorcyclizine)
Cevimeline N-Oxide	469890-14-0	C10H17NO2S	Pharmaceutical TP (Cevimeline)
Desethyl Chloroquine	1476-52-4	C16H22ClN3	Pharmaceutical TP (Chloroquine)
N,N-Dideethyl Chloroquine	4298-14-0	C14H18ClN3	Pharmaceutical TP (Chloroquine)
Chlorpheniramine N-Oxide	142494-45-9	C16H19ClN2O	Pharmaceutical TP (Chlorpheniramine)
N,N-Didemethyl Chlorpheniramine	20619-13-0	C14H15ClN2	Pharmaceutical TP (Chlorpheniramine)
N-Desmethyl Chlorpheniramine	20619-12-9	C15H17ClN2	Pharmaceutical TP (Chlorpheniramine)
Chlorpromazine N-Oxide Sulfoxide	10404-90-7	C17H19ClN2O2S	Pharmaceutical TP (Chlorpromazine)
Norchlorpromazine	1225-64-5	C16H17ClN2S	Pharmaceutical TP (Chlorpromazine)
Anhydrochlortetracycline	4497-08-9	C22H21ClN2O7	Pharmaceutical TP (Chlortetracycline)
Isochlortetracycline	514-53-4	C22H23ClN2O8	Pharmaceutical TP (Chlortetracycline)
6-Hydroxy Chlorzoxazone	1750-45-4	C7H4ClNO3	Pharmaceutical TP (Chlorzoxazone)
Cilazaprilat	90139-06-3	C20H27N3O5	Pharmaceutical TP (Cilazapril)
3,4-Dehydrocilostazol	73963-62-9	C20H25N5O2	Pharmaceutical TP (Cilostazol)
4'-trans-Hydroxy Cilostazol	87153-04-6	C20H27N5O3	Pharmaceutical TP (Cilostazol)
6-Hydroxy-3,4-dihydro-2(1H)-quinolinone	54197-66-9	C9H9NO2	Pharmaceutical TP (Cilostazol)
6-Hydroxy-6-defluoro Ciprofloxacin	226903-07-7	C17H19N3O4	Pharmaceutical TP (Ciprofloxacin)
Ciprofloxacin N-Oxide	860033-22-3	C17H18FN3O4	Pharmaceutical TP (Ciprofloxacin)
Ciprofloxacin-7-ethylenediamine	103222-12-4	C15H16FN3O3	Pharmaceutical TP (Ciprofloxacin)
Decarboxy Ciprofloxacin	105394-83-0	C16H18FN3O	Pharmaceutical TP (Ciprofloxacin)
Fluoroquinolonic Acid	86393-33-1	C13H9ClFNO3	Pharmaceutical TP (Ciprofloxacin)
Formyl Ciprofloxacin	93594-39-9	C18H18FN3O4	Pharmaceutical TP (Ciprofloxacin)
N-(tert-Butoxycarbonyl) Desethylene Ciprofloxacin	105589-00-2	C20H24FN3O5	Pharmaceutical TP (Ciprofloxacin)
Oxociprofloxacin	103237-52-1	C17H16FN3O4	Pharmaceutical TP (Ciprofloxacin)
Citalopram N-Oxide	63284-72-0	C20H21FN2O2	Pharmaceutical TP (Citalopram)
Demethylchloro Citalopram	64372-52-7	C19H19ClN2O	Pharmaceutical TP (Citalopram)
Didemethyl Citalopram	62498-69-5	C18H17FN2O	Pharmaceutical TP (Citalopram)

Table S20. Compound database for suspect screening (continued)

Compound Name	CAS	Molecular Formula	Category
N-Desmethyl Citalopram	62498-67-3	C19H19FN2O	Pharmaceutical TP (Citalopram)
Clarithromycin N-Oxide	118074-07-0	C38H69NO14	Pharmaceutical TP (Clarithromycin)
N-Desmethyl Clarithromycin	101666-68-6	C37H67NO13	Pharmaceutical TP (Clarithromycin)
Hydroxymethyl Clenbuterol	38339-18-3	C12H18Cl2N2O2	Pharmaceutical TP (Clenbuterol)
Clindamycin Sulfoxide	22431-46-5	C18H33ClN2O6S	Pharmaceutical TP (Clindamycin)
Norclobazam	22316-55-8	C15H11ClN2O2	Pharmaceutical TP (Clobazam)
Clomiphene N-Oxide	97642-74-5	C26H28ClNO2	Pharmaceutical TP (Clomifene)
8-Hydroxy Clomipramine	61523-80-6	C19H23ClN2O	Pharmaceutical TP (Clomipramine)
Clomipramine N-Oxide	14171-67-6	C19H23ClN2O	Pharmaceutical TP (Clomipramine)
Desmethyl Clomipramine	303-48-0	C18H21ClN2	Pharmaceutical TP (Clomipramine)
3-Chloroiminodibenzyl	32943-25-2	C14H12ClN	Pharmaceutical TP (Clomipramine, Desipramine)
4-Hydroxy Clonidine	57101-48-1	C9H9Cl2N3O	Pharmaceutical TP (Clonidine)
4-Methoxy Clonidine	65936-24-5	C10H11Cl2N3O	Pharmaceutical TP (Clonidine)
2-Oxo Clopidogrel	109904-27-0	C16H16ClNO3S	Pharmaceutical TP (Clopidogrel)
Clopidogrel Carboxylic Acid	144457-28-3	C15H14ClNO2S	Pharmaceutical TP (Clopidogrel)
N-Desmethyl Clotiazepam	33671-37-3	C15H13ClN2OS	Pharmaceutical TP (Clotiazepam)
2-Chlorobenzophenone	5162-03-8	C13H9ClO	Pharmaceutical TP (Clotrimazole)
2-Bromo-acetamide-2',5-dichlorobenzophenone	5504-92-7	C15H10BrCl2NO2	Pharmaceutical TP (Cloxazolam)
Clozapine N-Oxide	34233-69-7	C18H19ClN4O	Pharmaceutical TP (Clozapine)
Norclozapine	6104-71-8	C17H17ClN4	Pharmaceutical TP (Clozapine)
Anhydroecgonine Methyl Ester	43021-26-7	C10H15NO2	Pharmaceutical TP (Cocaine, Ecgonine)
Benzoylecgonine	519-09-5	C16H19NO4	Pharmaceutical TP (Cocaine)
Cocaethylene	529-38-4	C18H23NO4	Pharmaceutical TP (Cocaine)
Ecgonine Methyl Ester	7143-09-1	C10H17NO3	Pharmaceutical TP (Cocaine)
m-Hydroxy Benzoylecgonine	129944-99-6	C16H19NO5	Pharmaceutical TP (Cocaine)
m-Hydroxy Cocaine	71387-58-1	C17H21NO5	Pharmaceutical TP (Cocaine)
Norcocaine	18717-72-1	C16H19NO4	Pharmaceutical TP (Cocaine)
Codeine N-Oxide	3688-65-1	C18H21NO4	Pharmaceutical TP (Codeine)
Codeinone	467-13-0	C18H19NO3	Pharmaceutical TP (Codeine)
Norcodeine	467-15-2	C17H19NO3	Pharmaceutical TP (Codeine)
2-Demethyl Colchicine	7336-36-9	C21H23NO6	Pharmaceutical TP (Colchicine)
3-Demethyl Thiocolchicine	87424-25-7	C21H23NO5S	Pharmaceutical TP (Colchicine)
3-Desmethyl Colchicine	7336-33-6	C21H23NO6	Pharmaceutical TP (Colchicine)
Colchiceine	477-27-0	C21H23NO6	Pharmaceutical TP (Colchicine)
N-Deacetyl Colchicine	3476-50-4	C20H23NO5	Pharmaceutical TP (Colchicine)
5-Bromo-2-hydroxy-3-nitropyridine	15862-34-7	C5H3BrN2O3	Pharmaceutical TP (Crizotinib)
3-Hydroxy Cyclobenzaprine	30235-48-4	C20H21NO	Pharmaceutical TP (Cyclobenzaprine)
Cyclobenzaprine N-Oxide	6682-26-4	C20H21NO	Pharmaceutical TP (Cyclobenzaprine)
Norcyclobenzaprine	303-50-4	C19H19N	Pharmaceutical TP (Cyclobenzaprine)
3-Dechloroethylifosfamide	36761-83-8	C5H12ClN2O2P	Pharmaceutical TP (Cyclophosphamide)
4-Hydroperoxy Cyclophosphamide	39800-16-3	C7H15Cl2N2O4P	Pharmaceutical TP (Cyclophosphamide)
4-Oxo Cyclophosphamide	27046-19-1	C7H13Cl2N2O3P	Pharmaceutical TP (Cyclophosphamide)
Carboxyphosphamide	22788-18-7	C7H15Cl2N2O4P	Pharmaceutical TP (Cyclophosphamide)
5-Hydroxy Dantrolene	52130-25-3	C14H10N4O6	Pharmaceutical TP (Dantrolene)
N-Acetyl Dapsone	565-20-8	C14H14N2O3S	Pharmaceutical TP (Dapsone)
N-Hydroxylamine Dapsone	32695-27-5	C12H12N2O3S	Pharmaceutical TP (Dapsone)
3-Hydroxy Darifenacin	1285875-62-8	C28H30N2O3	Pharmaceutical TP (Darifenacin)
3'-Hydroxy Darunavir	1159613-24-7	C27H37N3O8S	Pharmaceutical TP (Darunavir)
4-Hydroxy Darunavir	313682-97-2	C27H37N3O8S	Pharmaceutical TP (Darunavir)
Hydroxy Darunavir	1130635-75-4	C27H37N3O8S	Pharmaceutical TP (Darunavir)
4'-Hydroxy Dasatinib	910297-57-3	C22H26ClN7O3S	Pharmaceutical TP (Dasatinib)
Dasatinib Carboxylic Acid	910297-53-9	C22H24ClN7O3S	Pharmaceutical TP (Dasatinib)
Dasatinib N-Oxide	910297-52-8	C22H26ClN7O3S	Pharmaceutical TP (Dasatinib)
Des-6-[4-(2-hydroxyethyl)-1-piperazinyl]-6-chloro Dasatinib	910297-71-1	C16H13Cl2N5O2S	Pharmaceutical TP (Dasatinib)
Hydroxymethyl Dasatinib	910297-58-4	C22H26ClN7O3S	Pharmaceutical TP (Dasatinib)
N-Deshydroxyethyl Dasatinib	910297-51-7	C20H22ClN7OS	Pharmaceutical TP (Dasatinib)
4-Demethyl Daunomycinone	52744-22-6	C20H16O8	Pharmaceutical TP (Daunorubicin)
Daunomycinone	21794-55-8	C21H18O8	Pharmaceutical TP (Daunorubicin)
4-Hydroxy Debrisoquine	59333-79-8	C10H13N3O	Pharmaceutical TP (Debrisoquine)
5-Hydroxy Debrisoquine	70746-05-3	C10H13N3O	Pharmaceutical TP (Debrisoquine)
8-Hydroxy Debrisoquine	46286-45-7	C10H13N3O	Pharmaceutical TP (Debrisoquine)
21-Desacetyl Deflazacort	13649-57-5	C23H29NO5	Pharmaceutical TP (Deflazacort)
6-Hydroxy-21-desacetyl Deflazacort	87539-45-5	C23H29NO6	Pharmaceutical TP (Deflazacort)
N-Desisopropyl Delavirdine	165133-86-8	C19H22N6O3S	Pharmaceutical TP (Delavirdine)
2-Hydroxy Desipramine	1977-15-7	C18H22N2O	Pharmaceutical TP (Desipramine)
N-Benzyl-2-formyl Desipramine	134150-70-2	C26H28N2O	Pharmaceutical TP (Desipramine)
5-Hydroxy Desloratadine	117811-12-8	C19H19ClN2O	Pharmaceutical TP (Desloratadine)

Table S20. Compound database for suspect screening (continued)

Compound Name	CAS	Molecular Formula	Category
6-Hydroxy Desloratadine	119410-05-8	C19H19ClN2O	Pharmaceutical TP (Desloratadine)
15beta-Hydroxy Desogestrel	869627-85-0	C22H30O2	Pharmaceutical TP (Desogestrel)
1,2-Dihydro Desoxymetasone	432-54-2	C22H31FO4	Pharmaceutical TP (Desoximetasone)
3-Carboxy Detomidine	115664-39-6	C12H12N2O2	Pharmaceutical TP (Detomidine)
3-Hydroxy Detomidine	115664-37-4	C12H14N2O	Pharmaceutical TP (Detomidine)
17beta-Carboxy-17alpha-formyloxy Dexamethasone	473273-04-0	C22H27FO6	Pharmaceutical TP (Dexamethasone)
17-Oxo Dexamethasone	1880-61-1	C20H25FO3	Pharmaceutical TP (Dexamethasone)
21-O-Acetyl 6beta-Hydroxy Dexamethasone	72559-77-4	C24H31FO7	Pharmaceutical TP (Dexamethasone)
3-Hydroxy Dexamethasone	922713-68-6	C22H31FO5	Pharmaceutical TP (Dexamethasone)
6beta-Hydroxy Dexamethasone	55879-47-5	C22H29FO6	Pharmaceutical TP (Dexamethasone)
N-Nordextromethorphan	1531-23-3	C17H23NO	Pharmaceutical TP (Dextromethorphan)
3-(Acetylamino)-5-amino-2,4,6-triiodobenzoic Acid	1713-07-1	C9H7I3N2O3	Pharmaceutical TP (Diatrizoic Acid (Diatrizoate))
3,5-bis(Acetylamino)benzoic Acid	7743-39-7	C11H12N2O4	Pharmaceutical TP (Diatrizoic Acid (Diatrizoate))
3,5-Diamino-2,4,6-triiodobenzoic Acid	5505-16-8	C7H5I3N2O2	Pharmaceutical TP (Diatrizoic Acid (Diatrizoate))
3,5-Diaminobenzoic Acid	535-87-5	C7H8N2O2	Pharmaceutical TP (Diatrizoic Acid (Diatrizoate))
2-Amino-5-chlorobenzophenone	719-59-5	C13H10ClNO	Pharmaceutical TP (Diazepam)
4'-Hydroxy Diazepam	17311-35-2	C16H13ClN2O2	Pharmaceutical TP (Diazepam)
4'-Hydroxy Nordiazepam	17270-12-1	C15H11ClN2O2	Pharmaceutical TP (Diazepam)
Nordiazepam	1088-11-5	C15H11ClN2O	Pharmaceutical TP (Diazepam)
3'-Hydroxy Diclofenac	69002-85-3	C14H11Cl2NO3	Pharmaceutical TP (Diclofenac)
4'-Hydroxy Diclofenac	64118-84-9	C14H11Cl2NO3	Pharmaceutical TP (Diclofenac)
5-Hydroxy Diclofenac	69002-84-2	C14H11Cl2NO3	Pharmaceutical TP (Diclofenac)
Diclofenac Amide	15362-40-0	C14H9Cl2NO	Pharmaceutical TP (Diclofenac)
Diclofenac Carboxylic Acid	13625-57-5	C13H9Cl2NO2	Pharmaceutical TP (Diclofenac)
11beta-Hydroxy Dienogest	86153-39-1	C20H25NO3	Pharmaceutical TP (Dienogest)
Diethylcarbamazine N-Oxide	34812-73-2	C10H21N3O2	Pharmaceutical TP (Diethylcarbamazine)
Dienestrol	84-17-3	C18H18O2	Pharmaceutical TP (Diethylstilbestrol)
Digoxigenin Bisdigitoxoside	5297-05-2	C35H54O11	Pharmaceutical TP (Digoxin)
Digoxigenin Monodigitoxoside	5352-63-6	C29H44O8	Pharmaceutical TP (Digoxin)
8'-Hydroxy Dihydroergotamine	90650-44-5	C33H37N5O6	Pharmaceutical TP (Dihydroergotamine)
Deacetyl Diltiazem	42399-40-6	C20H24N2O3S	Pharmaceutical TP (Diltiazem)
Deacetyl Diltiazem N-Oxide	122619-90-3	C20H24N2O4S	Pharmaceutical TP (Diltiazem)
Deacetyl-O-demethyl Diltiazem	84903-82-2	C19H22N2O3S	Pharmaceutical TP (Diltiazem)
Diltiazem N-Oxide	142843-04-7	C22H26N2O5S	Pharmaceutical TP (Diltiazem)
N,N,O-Tridesmethyl Diltiazem	159734-23-3	C19H20N2O4S	Pharmaceutical TP (Diltiazem)
N,N-Didesmethyl Diltiazem	115973-28-9	C20H22N2O4S	Pharmaceutical TP (Diltiazem)
N-Desmethyl Diltiazem	85100-17-0	C21H24N2O4S	Pharmaceutical TP (Diltiazem)
O-Desacetyl-N-desmethyl Diltiazem	81353-09-5	C19H22N2O3S	Pharmaceutical TP (Diltiazem)
Diphenhydramine N-Oxide	3922-74-5	C17H21NO2	Pharmaceutical TP (Diphenhydramine)
N-Desmethyl Diphenhydramine	17471-10-2	C16H19NO	Pharmaceutical TP (Diphenhydramine)
S-Methyl-N,N-diethylthiocarbamate	37174-63-3	C6H13NOS	Pharmaceutical TP (Disulfiram)
S-Methyl-N,N-diethylthiocarbamate Sulfone	155514-79-7	C6H13NO3S	Pharmaceutical TP (Disulfiram)
S-Methyl-N,N-diethylthiocarbamate Sulfoxide	140703-15-7	C6H13NO2S	Pharmaceutical TP (Disulfiram)
Docetaxel Hydroxy-tert-butyl-carbamate	154044-57-2	C43H53NO15	Pharmaceutical TP (Docetaxel)
Dofetilide N-Oxide	144449-71-8	C19H27N3O6S2	Pharmaceutical TP (Dofetilide)
N-Desmethyl Dofetilide	176447-94-2	C18H25N3O5S2	Pharmaceutical TP (Dofetilide)
5-O-Desmethyl Donepezil	120013-57-2	C23H27NO3	Pharmaceutical TP (Donepezil)
6-O-Desmethyl Donepezil	120013-56-1	C23H27NO3	Pharmaceutical TP (Donepezil)
Dihydro Donepezil	120012-04-6	C24H31NO3	Pharmaceutical TP (Donepezil)
Donepezil N-Oxide	120013-84-5	C24H29NO4	Pharmaceutical TP (Donepezil)
4-(2-Amino-ethyl)-2-methoxy-phenol	554-52-9	C9H13NO2	Pharmaceutical TP (Dopamine)
N-Deethyl Dorzolamide	154154-90-2	C8H12N2O4S3	Pharmaceutical TP (Dorzolamide)
6-Hydroxy Doxazosin	102932-26-3	C22H23N5O5	Pharmaceutical TP (Doxazosin)
7-Hydroxy Doxazosin	102932-25-2	C22H23N5O5	Pharmaceutical TP (Doxazosin)
(E)-2-Hydroxy Doxepin	131523-90-5	C19H21NO2	Pharmaceutical TP (Doxepin)
(E)-3-Hydroxy Doxepin	131523-97-2	C19H21NO2	Pharmaceutical TP (Doxepin)
(E)-N-Desmethyl-N-formyl Doxepin	250331-52-3	C19H19NO2	Pharmaceutical TP (Doxepin)
Doxepin N-Oxide	22684-91-9	C20H35NO2	Pharmaceutical TP (Doxepin)
Nordoxepin	1225-56-5	C18H19NO	Pharmaceutical TP (Doxepin)
Doxorubicinone	24385-10-2	C21H18O9	Pharmaceutical TP (Doxorubicin)
N-Desmethyl Doxycycline	86271-83-2	C21H22N2O8	Pharmaceutical TP (Doxycycline)
Desmethyl Doxylamine	1221-70-1	C16H20N2O	Pharmaceutical TP (Doxylamine)
Doxylamine N-Oxide	97143-65-2	C17H22N2O2	Pharmaceutical TP (Doxylamine)
4-Hydroxy Duloxetine	662149-13-5	C18H19NO2S	Pharmaceutical TP (Duloxetine)
5-Hydroxy Duloxetine	741693-77-6	C18H19NO2S	Pharmaceutical TP (Duloxetine)
5-Hydroxy-6-methoxy Duloxetine	741693-79-8	C19H21NO3S	Pharmaceutical TP (Duloxetine)
Dihydro Dutasteride	164656-22-8	C27H32F6N2O2	Pharmaceutical TP (Dutasteride)

Table S20. Compound database for suspect screening (continued)

Compound Name	CAS	Molecular Formula	Category
7-Hydroxy Efavirenz	205754-50-3	C14H9ClF3NO3	Pharmaceutical TP (Efavirenz)
8,14-Dihydroxy Efavirenz	1189909-96-3	C14H9ClF3NO4	Pharmaceutical TP (Efavirenz)
8-Hydroxy Efavirenz	205754-32-1	C14H9ClF3NO3	Pharmaceutical TP (Efavirenz)
Emtricitabine Carboxylic Acid	1238210-10-0	C8H8FN3O4S	Pharmaceutical TP (Emtricitabine)
Emtricitabine S-Oxide	152128-77-3	C8H10FN3O4S	Pharmaceutical TP (Emtricitabine)
Enalaprilat	76420-72-9	C18H24N2O5	Pharmaceutical TP (Enalapril)
Enzalutamide Carboxylic Acid	1242137-15-0	C20H13F4N3O3S	Pharmaceutical TP (Enzalutamide)
N-Desmethyl Enzalutamide	1242137-16-1	C20H14F4N4O2S	Pharmaceutical TP (Enzalutamide)
Norephedrine	492-41-1	C9H13NO	Pharmaceutical TP (Ephedrine)
9,13beta-Dehydro Epinastine	141342-70-3	C16H13N3	Pharmaceutical TP (Epinastine)
9-Oxo Epinastine	141342-69-0	C16H13N3O	Pharmaceutical TP (Epinastine)
Deoxy Epinephrine	501-15-5	C9H13NO2	Pharmaceutical TP (Epinephrine)
Metanephrine	5001-33-2	C10H15NO3	Pharmaceutical TP (Epinephrine)
Normetanephrine	97-31-4	C9H13NO3	Pharmaceutical TP (Epinephrine)
6beta-Hydroxy Eplerenone	209253-80-5	C24H30O7	Pharmaceutical TP (Eplerenone)
Eplerenone Hydroxy Acid	579484-30-3	C24H32O7	Pharmaceutical TP (Eplerenone)
17alpha-Dihydroequilin	651-55-8	C18H22O2	Pharmaceutical TP (Equilin)
8,9-Dehydroestrone	474-87-3	C18H20O2	Pharmaceutical TP (Equilin, Estrone)
Dihydrolysergamide	2410-19-7	C16H19N3O	Pharmaceutical TP (Ergotamine)
Desmethyl Erlotinib	183321-86-0	C21H21N3O4	Pharmaceutical TP (Erlotinib)
Desmethyl Erlotinib Carboxylate Acid	882172-60-3	C21H19N3O5	Pharmaceutical TP (Erlotinib)
Didesmethyl Erlotinib	183321-84-8	C20H19N3O4	Pharmaceutical TP (Erlotinib)
Erythromycylamine	26116-56-3	C37H70N2O12	Pharmaceutical TP (Erythromycin)
N-Demethyl Erythromycin	992-62-1	C36H65NO13	Pharmaceutical TP (Erythromycin)
Esmolol Acid	81148-15-4	C15H23NO4	Pharmaceutical TP (Esmolol)
16-Keto 17beta-Estradiol	566-75-6	C18H22O3	Pharmaceutical TP (Estradiol)
2-Hydroxy-17beta-estradiol	362-05-0	C18H24O3	Pharmaceutical TP (Estradiol)
4-Hydroxy-17beta-estradiol	5976-61-4	C18H24O3	Pharmaceutical TP (Estradiol)
4-Methoxy-17beta-estradiol	26788-23-8	C19H26O3	Pharmaceutical TP (Estradiol)
6alpha-Hydroxy 17beta-Estradiol	1229-24-9	C18H24O3	Pharmaceutical TP (Estradiol)
6-Keto 17beta-Estradiol	571-92-6	C18H22O3	Pharmaceutical TP (Estradiol)
16alpha-Hydroxyestrone	566-76-7	C18H22O3	Pharmaceutical TP (Estrone)
2,4-Dibromo Estrone	60788-62-7	C18H20Br2O2	Pharmaceutical TP (Estrone)
2-Hydroxy Estrone	362-06-1	C18H22O3	Pharmaceutical TP (Estrone)
2-Methoxy Estrone	362-08-3	C19H24O3	Pharmaceutical TP (Estrone)
4-Hydroxy Estrone	3131-23-5	C18H22O3	Pharmaceutical TP (Estrone)
4-Methoxy Estrone	58562-33-7	C19H24O3	Pharmaceutical TP (Estrone)
6-Keto Estrone	1476-34-2	C18H20O3	Pharmaceutical TP (Estrone)
Ethionamide Sulfoxide	536-28-7	C8H10N2OS	Pharmaceutical TP (Ethionamide)
2,6-Dihydro-2,2,4-trimethyl-6-quinolone	4071-18-5	C12H13NO	Pharmaceutical TP (Ethoxyquin)
4-Oxo Etodolac	111478-86-5	C17H19NO4	Pharmaceutical TP (Etodolac)
6-Hydroxy Etodolac	101901-06-8	C17H21NO4	Pharmaceutical TP (Etodolac)
Etomidate Acid	56649-48-0	C12H12N2O2	Pharmaceutical TP (Etomidate)
17beta-Hydroxy Exemestane	122370-91-6	C20H26O2	Pharmaceutical TP (Exemestane)
6beta-Hydroxymethylandrosta-1,4-diene-3,17-dione	121021-51-0	C20H26O3	Pharmaceutical TP (Exemestane)
6-Deoxy Penciclovir	104227-86-3	C10H15N5O2	Pharmaceutical TP (Famciclovir)
Desacetyl Famciclovir	120687-07-2	C12H17N5O3	Pharmaceutical TP (Famciclovir)
Desdiacetyl-8-oxo Famciclovir	166197-79-1	C10H15N5O3	Pharmaceutical TP (Famciclovir)
Famotidine Sulfoxide	90237-03-9	C8H15N7O3S3	Pharmaceutical TP (Famotidine)
2-Hydroxy Felbamate	109482-32-8	C11H14N2O5	Pharmaceutical TP (Felbamate)
4-Hyrdroxy Felbamate	109482-28-2	C11H14N2O5	Pharmaceutical TP (Felbamate)
5-Carboxy-6-hydroxymethyl Dehydro Felodipine	96558-29-1	C17H15Cl2NO5	Pharmaceutical TP (Felodipine)
Dehydro Felodipine	96382-71-7	C18H17Cl2NO4	Pharmaceutical TP (Felodipine)
Fenbendazole-amine	53065-28-4	C13H11N3S	Pharmaceutical TP (Fenbendazole)
Fenbendazole Sulfone	54029-20-8	C15H13N3O4S	Pharmaceutical TP (Fenbendazole, Oxfendazole)
Dihydro Fenofibrate	61001-99-8	C20H23ClO4	Pharmaceutical TP (Fenofibrate)
Norfentanyl	1609-66-1	C14H20N2O	Pharmaceutical TP (Fentanyl)
6alpha-Hydroxy Finasteride	154387-62-9	C23H36N2O3	Pharmaceutical TP (Finasteride)
Finasteride 2-(2-Methylpropanol)amide	116285-36-0	C23H36N2O3	Pharmaceutical TP (Finasteride)
Finasteride Carboxylic Acid	116285-37-1	C23H34N2O4	Pharmaceutical TP (Finasteride)
Flecainide Meta-O-dealkylated	83526-33-4	C15H19F3N2O3	Pharmaceutical TP (Flecainide)
Florfenicol-amine	76639-93-5	C10H14FNO3S	Pharmaceutical TP (Florfenicol)
2-Amino Flubendazole	82050-13-3	C14H10FN3O	Pharmaceutical TP (Flubendazole)
Hydroxy Flubendazole	82050-12-2	C16H14FN3O3	Pharmaceutical TP (Flubendazole)
7-Amino Flunitrazepam	34084-50-9	C16H14FN3O	Pharmaceutical TP (Flunitrazepam)
5-Hydroxy Flunixin	75369-61-8	C14H11F3N2O3	Pharmaceutical TP (Flunixin)
5-Fluorodihydropyrimidine-2,4-dione	696-06-0	C4H5FN2O2	Pharmaceutical TP (Fluorouracil)

Table S20. Compound database for suspect screening (continued)

Compound Name	CAS	Molecular Formula	Category
Norfluoxetine	83891-03-6	C16H16F3NO	Pharmaceutical TP (Fluoxetine)
10-(3-Chloropropyl)-2-(trifluoromethyl)-10H-phenothiazine	1675-46-3	C16H13ClF3NS	Pharmaceutical TP (Fluphenazine)
7-Hydroxy Fluphenazine	33098-48-5	C22H26F3N3O2S	Pharmaceutical TP (Fluphenazine)
Didesethyl Flurazepam	17617-59-3	C17H15ClFN3O	Pharmaceutical TP (Flurazepam)
3',4'-Dihydroxy Flurbiprofen	66067-41-2	C15H13FO4	Pharmaceutical TP (Flurbiprofen)
4'-Hydroxy Flurbiprofen	52807-12-2	C15H13FO3	Pharmaceutical TP (Flurbiprofen)
2-Amino-5-nitro-4-(trifluoromethyl)phenol	56987-02-1	C7H5F3N2O3	Pharmaceutical TP (Flutamide)
4'-Nitro-3'-(trifluoromethyl)acetanilide	393-12-4	C9H7F3N2O3	Pharmaceutical TP (Flutamide)
Hydroxy Flutamide	52806-53-8	C11H11F3N2O4	Pharmaceutical TP (Flutamide)
N-Hydroxy-4-nitro-3-(trifluoromethyl)aniline	904328-95-6	C7H5F3N2O3	Pharmaceutical TP (Flutamide)
Fluticasone 17beta-Carboxylic Acid	28416-82-2	C21H26F2O5	Pharmaceutical TP (Fluticasone)
O-tert-Butyl-3-keto Fluvastatin	194934-95-7	C28H32FNO4	Pharmaceutical TP (Fluvastatin)
Fluvoxamine Acid	88699-91-6	C14H17F3N2O3	Pharmaceutical TP (Fluvoxamine)
N-Acetyl Fluvoxamine Acid	88699-87-0	C16H19F3N2O4	Pharmaceutical TP (Fluvoxamine)
Fosinoprilat	95399-71-6	C23H34NO5P	Pharmaceutical TP (Fosinopril)
3-Amino-2-oxazolidinone	80-65-9	C3H6N2O2	Pharmaceutical TP (Furazolidone)
3-Amino-5-(morpholin-4-ylmethyl)-1,3-oxazolidin-2-one	43056-63-9	C8H15N3O3	Pharmaceutical TP (Furazolidone)
4-Chloro-5-sulfamoylanthranilic Acid (Saluamine)	3086-91-7	C7H7ClN2O4S	Pharmaceutical TP (Furosemide)
11-Keto Fusidic Acid	16711-91-4	C31H46O6	Pharmaceutical TP (Fusidic Acid)
3-Keto Fusidic Acid	4680-37-9	C31H46O6	Pharmaceutical TP (Fusidic Acid)
9,11-Anhydro-12-hydroxy Fusidic Acid	74048-44-5	C31H46O6	Pharmaceutical TP (Fusidic Acid)
1-Carboxycyclohexaneacetic Acid (Gabapentin Related Compound E)	67950-95-2	C9H14O4	Pharmaceutical TP (Gabapentin)
Gabapentin Lactam	64744-50-9	C9H15NO	Pharmaceutical TP (Gabapentin)
Norgalanthamine	41303-74-6	C16H19NO3	Pharmaceutical TP (Galantamine)
O-Desmethyl Galanthamine	60755-80-8	C17H21NO4	Pharmaceutical TP (Galantamine)
Desethylene Gatifloxacin	172426-87-8	C17H20FN3O4	Pharmaceutical TP (Gatifloxacin)
Despropylene Gatifloxacin	172426-86-7	C16H18FN3O4	Pharmaceutical TP (Gatifloxacin)
4-Defluoro-4-hydroxy Gefitinib	847949-50-2	C22H25ClN4O4	Pharmaceutical TP (Gefitinib)
Gefitinib N-Oxide	847949-51-3	C22H24ClFN4O4	Pharmaceutical TP (Gefitinib)
O-Desmethyl Gefitinib	847949-49-9	C21H22ClFN4O3	Pharmaceutical TP (Gefitinib)
O-Desmorpholinopropyl Gefitinib	184475-71-6	C15H11ClFN3O2	Pharmaceutical TP (Gefitinib)
2',2'-Difluoro-2'-deoxyuridine	114248-23-6	C9H10F2N2O5	Pharmaceutical TP (Gemcitabine)
Carboxy Glimepiride	127554-90-9	C24H32N4O7S	Pharmaceutical TP (Glimepiride)
Hydroxy Glimepiride	600177-94-4	C24H34N4O6S	Pharmaceutical TP (Glimepiride)
5-Chloro-2-methoxy-N-(2-phenylethyl)benzamide	33924-49-1	C16H16ClNO2	Pharmaceutical TP (Glyburide)
cis-3-Hydroxy Glyburide	23074-02-4	C23H28ClN3O6S	Pharmaceutical TP (Glyburide)
trans-4-Hydroxy Glyburide	23155-00-2	C16H16ClNO2	Pharmaceutical TP (Glyburide)
7-Hydroxy Granisetron	133841-15-3	C18H24N4O2	Pharmaceutical TP (Granisetron)
4-O-Demethyl Griseofulvin	5128-41-6	C16H15ClO6	Pharmaceutical TP (Griseofulvin)
3-Hydroxy Guanfacine	78197-84-9	C9H9Cl2N3O2	Pharmaceutical TP (Guanfacine)
Haloperidol N-Oxide	148406-51-3	C21H23ClFNO3	Pharmaceutical TP (Haloperidol)
Hydroxy Haloperidol	34104-67-1	C21H25ClFNO2	Pharmaceutical TP (Haloperidol)
6-Acetylmorphine	2784-73-8	C19H21NO4	Pharmaceutical TP (Heroin)
4-Amino-6-chlorobenzene-1,3-disulfonamide	121-30-2	C6H8ClN3O4S2	Pharmaceutical TP (Hydrochlorothiazide)
Norhydrocodone	5083-62-5	C17H19NO3	Pharmaceutical TP (Hydrocodone)
6alpha-Hydroxycortisol	2242-98-0	C21H30O6	Pharmaceutical TP (Hydrocortisone)
Hydromorphone N-Oxide	109648-80-8	C17H19NO4	Pharmaceutical TP (Hydromorphone)
Cletoquine	4298-15-1	C16H22ClN3O	Pharmaceutical TP (Hydroxychloroquine)
Norhyoscyamine	537-29-1	C16H21NO3	Pharmaceutical TP (Hyoscyamine)
Dihydrodiol Ibrutinib	1654820-87-7	C25H26N6O4	Pharmaceutical TP (Ibrutinib)
1-Hydroxy Ibuprofen	53949-53-4	C13H18O3	Pharmaceutical TP (Ibuprofen)
2-Hydroxy Ibuprofen	51146-55-5	C13H18O3	Pharmaceutical TP (Ibuprofen)
3-Hydroxy Ibuprofen	53949-54-5	C13H18O3	Pharmaceutical TP (Ibuprofen)
Ibuprofen Carboxylic Acid (Carboxyibuprofen)	15935-54-3	C13H16O4	Pharmaceutical TP (Ibuprofen)
Iloperidone Carboxylic Acid	475110-48-6	C23H25FN2O5	Pharmaceutical TP (Iloperidone)
Imatinib (Piperidine)-N-oxide	571186-91-9	C29H31N7O2	Pharmaceutical TP (Imatinib)
Imatinib (Pyridine)-N-oxide	571186-92-0	C29H31N7O2	Pharmaceutical TP (Imatinib)
N-Desmethyl Imatinib	404844-02-6	C28H29N7O	Pharmaceutical TP (Imatinib)
N-[(S)-1-Ethoxycarbonyl-3-phenylpropyl]-L-alanine	82717-96-2	C15H21NO4	Pharmaceutical TP (Imidapril)
2-Hydroxy Imipramine	303-70-8	C19H24N2O	Pharmaceutical TP (Imipramine)
Imipramine N-Oxide	6829-98-7	C19H24N2O	Pharmaceutical TP (Imipramine)
Desamino Imiquimod	99010-24-9	C14H15N3	Pharmaceutical TP (Imiquimod)
Desamino Imiquimod N-Oxide	99010-63-6	C14H15N3O	Pharmaceutical TP (Imiquimod)
N-Deschlorobenzoyl Indomethacin	2882-15-7	C12H13NO3	Pharmaceutical TP (Indomethacin)
O-Desmethyl Indomethacin	2504-32-7	C18H14ClNO4	Pharmaceutical TP (Indomethacin)
O-Desmethyl-N-Deschlorobenzoyl Indomethacin	50995-53-4	C11H11NO3	Pharmaceutical TP (Indomethacin)
Hydroxy Ipronidazole	35175-14-5	C7H11N3O3	Pharmaceutical TP (Ipronidazole)

Table S20. Compound database for suspect screening (continued)

Compound Name	CAS	Molecular Formula	Category
7-Ethyl-10-(4-amino-1-piperidino)carbonyloxycamptothecin	185304-42-1	C28H30N4O6	Pharmaceutical TP (Irinotecan)
7-Ethyl-10-(4-N-aminopentanoic acid)-1-piperidino)carbonyloxycamptothecin	181467-56-1	C33H38N4O8	Pharmaceutical TP (Irinotecan)
N-Acetyl Isoniazid	1078-38-2	C8H9N3O2	Pharmaceutical TP (Isoniazid)
Dehydro Isradipine	116169-18-7	C19H19N3O5	Pharmaceutical TP (Isradipine)
Hydroxy Itraconazole	112559-91-8	C35H38Cl2N8O5	Pharmaceutical TP (Itraconazole)
Keto Itraconazole	112560-33-5	C35H36Cl2N8O5	Pharmaceutical TP (Itraconazole)
7-Demethyl Ivabradine	304462-60-0	C26H34N2O5	Pharmaceutical TP (Ivabradine)
8-Demethyl Ivabradine	304464-97-9	C26H34N2O5	Pharmaceutical TP (Ivabradine)
N-Demethyl Ivabradine	215935-23-2	C26H34N2O5	Pharmaceutical TP (Ivabradine)
Hydroxymethyl Ivacaftor	1246213-23-9	C24H28N2O4	Pharmaceutical TP (Ivacaftor)
Ivacaftor Carboxylic Acid	1246213-24-0	C24H26N2O5	Pharmaceutical TP (Ivacaftor)
Norketamine	35211-10-0	C12H14ClNO	Pharmaceutical TP (Ketamine)
Dihydro Ketoprofen	59960-32-6	C16H16O3	Pharmaceutical TP (Ketoprofen)
4-Hydroxy Ketorolac	111930-01-9	C15H13NO4	Pharmaceutical TP (Ketorolac)
4-(3-Aminobutyl)phenol	52846-75-0	C10H15NO	Pharmaceutical TP (Labetalol)
Desacetyl Desmethyl Lacosamide	175481-39-7	C10H14N2O2	Pharmaceutical TP (Lacosamide)
Desmethyl Lacosamide	175481-38-6	C12H16N2O3	Pharmaceutical TP (Lacosamide)
Lamivudine Carboxylic Acid	173602-25-0	C8H9N3O4S	Pharmaceutical TP (Lamivudine)
Lamivudine S-Oxide	160552-54-5	C8H11N3O4S	Pharmaceutical TP (Lamivudine)
2-Methyl Lamotrigine	1152091-68-3	C10H9Cl2N5	Pharmaceutical TP (Lamotrigine)
4-Hydroxy Lansoprazole Sulfide	131926-95-9	C16H14F3N3O2S	Pharmaceutical TP (Lansoprazole)
5-Hydroxy Lansoprazole	131926-98-2	C16H14F3N3O3S	Pharmaceutical TP (Lansoprazole)
5-Hydroxy Lansoprazole Sulfide	131926-96-0	C16H14F3N3O2S	Pharmaceutical TP (Lansoprazole)
Lansoprazole N-oxide	213476-12-1	C16H14F3N3O3S	Pharmaceutical TP (Lansoprazole)
Lansoprazole Sulfide	103577-40-8	C16H14F3N3OS	Pharmaceutical TP (Lansoprazole)
Lansoprazole Sulfone	131926-99-3	C16H14F3N3O3S	Pharmaceutical TP (Lansoprazole)
Latanoprost Acid	41639-83-2	C23H34O5	Pharmaceutical TP (Latanoprost)
4-Hydroxy Teriflunomide	1058722-45-4	C12H9F3N2O3	Pharmaceutical TP (Leflunomide)
5-Hydroxy Leflunomide	1058722-46-5	C12H9F3N2O3	Pharmaceutical TP (Leflunomide)
Hydroxy Lenalidomide	1421593-78-3	C13H13N3O4	Pharmaceutical TP (Lenalidomide)
N-Acetyl Lenalidomide	1421593-80-7	C15H15N3O4	Pharmaceutical TP (Lenalidomide)
Lenvatinib N-Oxide	1788901-86-9	C21H19ClN4O5	Pharmaceutical TP (Lenvatinib)
Bis(4-cyanophenyl)methanol	134521-16-7	C15H10N2O	Pharmaceutical TP (Letrozole)
2-Oxo-3-(2-mercaptoethyl)-5-phenylimidazolidine	32190-33-3	C11H14N2OS	Pharmaceutical TP (Levamisole)
4-Hydroxy Levamisole	69359-04-2	C11H12N2OS	Pharmaceutical TP (Levamisole)
4-Phenyl-4,5-dihydro-1,3-oxazol-2-amine	52883-35-9	C9H10N2O	Pharmaceutical TP (Levamisole)
Levetiracetam Carboxylic Acid	102849-49-0	C8H13NO3	Pharmaceutical TP (Levetiracetam)
3-Hydroxy Lidocaine	34604-55-2	C14H22N2O2	Pharmaceutical TP (Lidocaine)
3-Hydroxy-N-desethyl Lidocaine	34604-56-3	C12H18N2O2	Pharmaceutical TP (Lidocaine)
4-Hydroxy Lidocaine	39942-41-1	C14H22N2O2	Pharmaceutical TP (Lidocaine)
Glycinexylidide	18865-38-8	C10H14N2O	Pharmaceutical TP (Lidocaine)
Lidocaine N-Oxide	2903-45-9	C14H22N2O2	Pharmaceutical TP (Lidocaine)
Norlidocaine (Monoethylglycinexylidide)	7728-40-7	C12H18N2O	Pharmaceutical TP (Lidocaine)
Linezolid N-Oxide	189038-36-6	C16H20FN3O5	Pharmaceutical TP (Linezolid)
N,O-Desethylene Linezolid	1219708-30-1	C14H18FN3O4	Pharmaceutical TP (Linezolid)
Loperamide N-Oxide	106900-12-3	C29H33ClN2O3	Pharmaceutical TP (Loperamide)
N-Desmethyl Loperamide	66164-07-6	C28H31ClN2O2	Pharmaceutical TP (Loperamide)
N-Didesmethyl Loperamide	66164-06-5	C27H29ClN2O2	Pharmaceutical TP (Loperamide)
2-Hydroxymethyl Loratadine	609806-39-5	C23H25ClN2O3	Pharmaceutical TP (Loratadine)
3-Hydroxy Desloratadine	119410-08-1	C19H19ClN2O	Pharmaceutical TP (Loratadine)
4-Hydroxymethyl Loratadine	609806-40-8	C23H25ClN2O3	Pharmaceutical TP (Loratadine)
Loratadine N-Oxide	165739-62-8	C22H23ClN2O3	Pharmaceutical TP (Loratadine)
6-Chloro-4-(2-chlorophenyl)-2-quinazolinecarboxylic Acid	54643-79-7	C15H8Cl2N2O2	Pharmaceutical TP (Lorazepam)
N-Hydroxy Lorcaserin	1421747-19-4	C11H14ClNO	Pharmaceutical TP (Lorcaserin)
5'-Hydroxy Lornoxicam	123252-96-0	C13H10ClN3O5S2	Pharmaceutical TP (Lornoxicam)
Losartan Carboxylic Acid	124750-92-1	C22H21ClN6O2	Pharmaceutical TP (Losartan)
omega-1-Hydroxy Losartan	141675-57-2	C22H23ClN6O2	Pharmaceutical TP (Losartan)
Lovastatin Acid	75225-51-3	C24H38O6	Pharmaceutical TP (Lovastatin)
8-Hydroxy Loxapine	61443-77-4	C18H18ClN3O2	Pharmaceutical TP (Loxapine)
8-Methoxy Loxapine	70020-54-1	C19H20ClN3O2	Pharmaceutical TP (Loxapine)
Loxapine N-Oxide	25967-34-4	C18H18ClN3O2	Pharmaceutical TP (Loxapine)
15-Hydroxy Lubiprostone	475992-30-4	C20H34F2O5	Pharmaceutical TP (Lubiprostone)
Lurasidone Sulfoxide	1809325-45-8	C28H36N4O3S	Pharmaceutical TP (Lurasidone)
2-Oxo-3-hydroxy-lysergide (O-H-LSD)	111295-09-1	C20H25N3O3	Pharmaceutical TP (Lysergide)
N-Despropyl Macitentan	1103522-45-7	C16H14Br2N6O4S	Pharmaceutical TP (Macitentan)
4-Hydroxyphenyl Maraviroc	856708-54-8	C29H41F2N5O2	Pharmaceutical TP (Maraviroc)
Des[1-(4,4-difluorocyclohexanecarboxamido)-1-phenylpropyl]-3-hydroxymethyl Maraviroc	856703-83-8	C13H22N4O	Pharmaceutical TP (Maraviroc)

Table S20. Compound database for suspect screening (continued)

Compound Name	CAS	Molecular Formula	Category
Dihydro Mebendazole	60254-95-7	C16H15N3O3	Pharmaceutical TP (Mebendazole)
Mebendazole-amine	52329-60-9	C14H11N3O	Pharmaceutical TP (Mebendazole)
3-Desmethyl 4-Methyl Meclizine	873395-53-0	C25H27ClN2	Pharmaceutical TP (Meclizine)
Meclizine N,N'-Dioxide	114624-70-3	C25H27ClN2O2	Pharmaceutical TP (Meclizine)
Meclizine N'-Oxide	114624-69-0	C25H27ClN2O	Pharmaceutical TP (Meclizine)
3-Hydroxymethyl Meclofenamic Acid	67318-61-0	C14H11Cl2NO3	Pharmaceutical TP (Meclofenamic Acid)
3-Hydroxy Medetomidine	128366-50-7	C13H16N2O	Pharmaceutical TP (Medetomidine)
3-Carboxy Mefenamic Acid	190379-82-9	C15H13NO4	Pharmaceutical TP (Mefenamic Acid)
3-Hydroxymethyl Mefenamic Acid	5129-20-4	C15H15NO3	Pharmaceutical TP (Mefenamic Acid)
Carboxy Mefloquine	35853-50-0	C12H5F6NO2	Pharmaceutical TP (Mefloquine)
2-Hydroxy Melatonin	229018-17-1	C13H16N2O3	Pharmaceutical TP (Melatonin)
4-Hydroxy Melatonin	39998-64-6	C13H16N2O3	Pharmaceutical TP (Melatonin)
5-Methoxy Tryptamine	608-07-1	C11H14N2O	Pharmaceutical TP (Melatonin)
6-Hydroxy Melatonin	2208-41-5	C13H16N2O3	Pharmaceutical TP (Melatonin)
N-Acetyl-5-hydroxy Tryptamine	1210-83-9	C12H14N2O2	Pharmaceutical TP (Melatonin)
5'-Carboxy Meloxicam	130262-93-0	C14H11N3O6S2	Pharmaceutical TP (Meloxicam)
5'-Hydroxy Meloxicam	130262-92-9	C14H13N3O5S2	Pharmaceutical TP (Meloxicam)
Hydroxy Melphalan	61733-01-5	C13H19ClN2O3	Pharmaceutical TP (Melphalan)
Normeperidine	77-17-8	C14H19NO2	Pharmaceutical TP (Meperidine)
2,6-Dimethyl-3-phenylmethoxyaniline	70261-50-6	C15H17NO	Pharmaceutical TP (Mepivacaine)
4'-Hydroxy Mepivacaine	616-66-0	C15H22N2O2	Pharmaceutical TP (Mepivacaine)
N-Desmethyl 3-Hydroxy Mepivacaine	247061-17-2	C14H20N2O2	Pharmaceutical TP (Mepivacaine)
Hydroxy Meprobamate	3567-43-9	C9H18N2O5	Pharmaceutical TP (Meprobamate)
O-Desmethyl Pyrilamine	57830-29-2	C16H21N3O	Pharmaceutical TP (Mepyramine)
N-Acetyl Mesalazine	51-59-2	C9H9NO4	Pharmaceutical TP (Mesalazine)
N-Formyl Mesalazine	104786-99-4	C8H7NO4	Pharmaceutical TP (Mesalazine)
N-Propionyl Mesalazine	93968-80-0	C10H11NO4	Pharmaceutical TP (Mesalazine)
3-Demethyl Mescaline	16046-07-4	C10H15NO3	Pharmaceutical TP (Mescaline)
N-Acetyl Mescaline	4593-89-9	C13H19NO4	Pharmaceutical TP (Mescaline)
4-Hydroxy Mestranol	65694-22-6	C21H26O3	Pharmaceutical TP (Mestranol)
Guanylurea	141-83-3	C2H6N4O	Pharmaceutical TP (Metformin)
2-Ethylidene-1,5-dimethyl-3,3-diphenylpyrrolidine (EDDP)	30223-73-5	C20H23N	Pharmaceutical TP (Methadone)
2-Ethyl-5-methyl-3,3-diphenylpyrrolidine (EMDP)	57100-29-5	C19H23N	Pharmaceutical TP (Methadone)
6-Hydroxy Methaqualone	5060-51-5	C16H14N2O2	Pharmaceutical TP (Methaqualone)
5-(Acetylimino)-4,5-dihydro-4-methyl-1,3,4-thiadiazole-2-sulfonic Acid	1312679-00-7	C5H7N3O4S2	Pharmaceutical TP (Methazolamide)
7-Hydroxy Methotrexate	5939-37-7	C20H22N8O6	Pharmaceutical TP (Methotrexate)
Deoxyaminopteroic Acid (DAMPA)	19741-14-1	C15H15N7O2	Pharmaceutical TP (Methotrexate)
4',5'-Dihydro-8-methoxypsoralen	3779-03-1	C12H10O4	Pharmaceutical TP (Methoxsalen)
N-Desmethyl Methsuximide	1497-17-2	C11H11NO2	Pharmaceutical TP (Methsuximide)
Ritalinic Acid	19395-41-6	C13H17NO2	Pharmaceutical TP (Methylphenidate, Ethylphenidate)
Metoclopramide N4-Sulfonate	27260-42-0	C14H22ClN3O5S	Pharmaceutical TP (Metoclopramide)
N-Des(2-diethylamino) Metoclopramide Acetic Acid	65567-29-5	C10H11ClN2O4	Pharmaceutical TP (Metoclopramide)
N-Desethyl Metoclopramide	27260-19-1	C12H18ClN3O2	Pharmaceutical TP (Metoclopramide)
1-[4-[2-Hydroxy-3-(propan-2-ylamino)propoxy]phenyl]-2-methoxyethanone	73723-85-0	C15H23NO4	Pharmaceutical TP (Metoprolol)
alpha-Hydroxy Metoprolol	56392-16-6	C15H25NO4	Pharmaceutical TP (Metoprolol)
N-Desisopropyl Metoprolol	74027-60-4	C12H19NO3	Pharmaceutical TP (Metoprolol)
O-Demethyl Metoprolol	62572-94-5	C14H23NO3	Pharmaceutical TP (Metoprolol)
Prenalterol	57526-81-5	C12H19NO3	Pharmaceutical TP (Metoprolol)
Metoprolol Acid (Atenolol Acid)	56392-14-4	C14H21NO4	Pharmaceutical TP (Metoprolol, Atenolol)
Hydroxy Metronidazole	4812-40-2	C6H9N3O4	Pharmaceutical TP (Metronidazole)
4-Hydroxy Mexiletine	53566-99-7	C11H17NO2	Pharmaceutical TP (Mexiletine)
N-Hydroxy Mexiletine	55304-17-1	C11H17NO2	Pharmaceutical TP (Mexiletine)
1-Hydroxy Midazolam	59468-90-5	C18H13ClFN3O	Pharmaceutical TP (Midazolam)
4-Hydroxy Midazolam	59468-85-8	C18H13ClFN3O	Pharmaceutical TP (Midazolam)
Desglymidodrine	3600-87-1	C10H15NO3	Pharmaceutical TP (Midodrine)
Didemethyl Mifepristone	104004-92-4	C27H31NO2	Pharmaceutical TP (Mifepristone)
Hydroxy Mifepristone	105012-15-5	C29H35NO3	Pharmaceutical TP (Mifepristone)
N-Demethyl Mifepristone	104004-96-8	C28H33NO2	Pharmaceutical TP (Mifepristone)
N-Desethyl Milnacipran	105310-07-4	C13H18N2O	Pharmaceutical TP (Milnacipran)
9-Didemethyl Minocycline	5874-95-3	C21H23N3O7	Pharmaceutical TP (Minocycline)
8-Hydroxy Mirtazapine	102335-57-9	C17H19N3O	Pharmaceutical TP (Mirtazapine)
Mirtazapine N-Oxide	155172-12-6	C17H19N3O	Pharmaceutical TP (Mirtazapine)
N-Desmethyl Mirtazapine	61337-68-6	C16H17N3	Pharmaceutical TP (Mirtazapine)
Misoprostol Acid	112137-89-0	C21H36O5	Pharmaceutical TP (Misoprostol)
7-Hydroxy Mitomycin	7041-61-4	C15H17N3O6	Pharmaceutical TP (Mitomycin)
Moexiprilat	103775-14-0	C25H30N2O7	Pharmaceutical TP (Moexipril)
6beta-Hydroxy Mometasone Furoate	132160-74-8	C27H30Cl2O7	Pharmaceutical TP (Mometasone)

Table S20. Compound database for suspect screening (continued)

Compound Name	CAS	Molecular Formula	Category
Morphine N-Oxide	639-46-3	C17H19NO4	Pharmaceutical TP (Morphine)
8-Ethoxy Moxifloxacin	1029364-75-7	C22H26FN3O4	Pharmaceutical TP (Moxifloxacin)
O-Desmethyl Mycophenolic Acid	31858-65-8	C16H18O6	Pharmaceutical TP (Mycophenolic Acid)
6-Methoxy-2-Naphthylacetic Acid	23981-47-7	C13H12O3	Pharmaceutical TP (Nabumetone)
Naloxone N-Oxide	112242-14-5	C19H21NO5	Pharmaceutical TP (Naloxone)
2-Hydroxy-3-O-methyl Naltrexone	67829-18-9	C21H25NO5	Pharmaceutical TP (Naltrexone)
O-Desmethyl Naproxen	52079-10-4	C13H12O3	Pharmaceutical TP (Naproxen)
Naratriptan N-Oxide	1159977-52-2	C17H25N3O3S	Pharmaceutical TP (Naratriptan)
4-Hydroxy Nebivolol	178383-76-1	C22H25F2NO5	Pharmaceutical TP (Nebivolol)
Hydroxy Nefazodone	98159-83-2	C25H32ClN5O3	Pharmaceutical TP (Nefazodone)
Nelfinavir Hydroxy-tert-butylamide	213135-56-9	C32H45N3O5S	Pharmaceutical TP (Nelfinavir)
12-Hydroxy Nevirapine	133627-24-4	C15H14N4O2	Pharmaceutical TP (Nevirapine)
2-Hydroxy Nevirapine	254889-31-1	C15H14N4O2	Pharmaceutical TP (Nevirapine)
3-Hydroxy Nevirapine	174532-82-2	C15H14N4O2	Pharmaceutical TP (Nevirapine)
Dehydro Nicardipine	59875-58-0	C26H27N3O6	Pharmaceutical TP (Nicardipine)
2-Hydroxy Nicotine	6969-92-2	C10H14N2O	Pharmaceutical TP (Nicotine)
3-Hydroxy Cotinine	34834-67-8	C10H12N2O2	Pharmaceutical TP (Nicotine)
5-(3-Pyridyl)tetrahydro-2-furanone	20971-79-3	C9H9NO2	Pharmaceutical TP (Nicotine)
5-Methyl Myosmine	102780-52-9	C10H12N2	Pharmaceutical TP (Nicotine)
5-Methyl Nornicotine	126741-11-5	C10H14N2	Pharmaceutical TP (Nicotine)
Cotinine	486-56-6	C10H12N2O	Pharmaceutical TP (Nicotine)
Cotinine N-Oxide	36508-80-2	C10H12N2O2	Pharmaceutical TP (Nicotine)
Nornicotine	5746-86-1	C9H12N2	Pharmaceutical TP (Nicotine)
Dehydro Nifedipine	67035-22-7	C17H16N2O6	Pharmaceutical TP (Nifedipine)
Dehydronitroso Nifedipine	50428-14-3	C17H16N2O5	Pharmaceutical TP (Nifedipine)
Hydroxydehydro Nifedipine Carboxylate	34783-31-8	C16H14N2O7	Pharmaceutical TP (Nifedipine)
Nilotinib N-Oxide	1246817-85-5	C28H22F3N7O2	Pharmaceutical TP (Nilotinib)
7-Amino Nimetazepam	4959-16-4	C16H15N3O	Pharmaceutical TP (Nimetazepam)
4-Hydroxy Nisoldipine	106685-70-5	C20H24N2O7	Pharmaceutical TP (Nisoldipine)
Dehydro Nisoldipine	103026-83-1	C20H22N2O6	Pharmaceutical TP (Nisoldipine)
7-Amino Nitrazepam	4928-02-3	C15H13N3O	Pharmaceutical TP (Nitrazepam)
4-Hydroxy Nitrofurantoin	76644-41-2	C8H6N4O6	Pharmaceutical TP (Nitrofurantoin)
Desmethyl Nizatidine	82586-78-5	C11H19N5O2S2	Pharmaceutical TP (Nizatidine)
10beta-Hydroxy Norethindrone	1236-00-6	C20H26O3	Pharmaceutical TP (Norethindrone)
6beta-Hydroxy Norethindrone	51724-44-8	C20H26O3	Pharmaceutical TP (Norethindrone)
4-Oxo Norfloxacin	74011-42-0	C16H16FN3O4	Pharmaceutical TP (Norfloxacin)
7-Desethyl Norfloxacin	75001-77-3	C14H16FN3O3	Pharmaceutical TP (Norfloxacin)
N-Acetyl Norfloxacin	74011-56-6	C18H20FN3O4	Pharmaceutical TP (Norfloxacin)
N-Formyl Norfloxacin	70459-04-0	C17H18FN3O4	Pharmaceutical TP (Norfloxacin)
N-Hydroxy Norfloxacin	109142-49-6	C16H18FN3O4	Pharmaceutical TP (Norfloxacin)
17-Desacetyl Norgestimate	53016-31-2	C21H29NO2	Pharmaceutical TP (Norgestimate)
16beta-Hydroxy Norgestrel	40915-03-5	C21H28O3	Pharmaceutical TP (Norgestrel)
3alpha,5beta-Tetrahydro Norgestrel	19351-16-7	C21H32O2	Pharmaceutical TP (Norgestrel)
10-Hydroxy Nortriptyline	1156-99-6	C19H21NO	Pharmaceutical TP (Nortriptyline)
cis-10,11-Dihydroxy Nortriptyline	1562-52-3	C19H21NO2	Pharmaceutical TP (Nortriptyline)
N-Desmethyl Ofloxacin	82419-52-1	C17H18FN3O4	Pharmaceutical TP (Ofloxacin)
Ofloxacin N-Oxide	104721-52-0	C18H20FN3O5	Pharmaceutical TP (Ofloxacin)
2-Hydroxymethyl Olanzapine	174756-45-7	C17H20N4OS	Pharmaceutical TP (Olanzapine)
N-Demethyl-N-formyl Olanzapine	639460-79-0	C17H18N4OS	Pharmaceutical TP (Olanzapine)
N-Desmethyl Olanzapine	161696-76-0	C16H18N4S	Pharmaceutical TP (Olanzapine)
Olanzapine 2-Carboxaldehyde	1330277-34-3	C17H18N4OS	Pharmaceutical TP (Olanzapine)
Olanzapine N-Oxide	174794-02-6	C17H20N4OS	Pharmaceutical TP (Olanzapine)
N-Desmethyl Olopatadine	113835-92-0	C20H21NO3	Pharmaceutical TP (Olopatadine)
Olopatadine N-Oxide	203188-31-2	C21H23NO4	Pharmaceutical TP (Olopatadine)
4-Acetyloxy Omeprazole	1246814-65-2	C18H19N3O4S	Pharmaceutical TP (Omeprazole)
4-Desmethoxy Omeprazole	110374-16-8	C16H17N3O2S	Pharmaceutical TP (Omeprazole)
4-Hydroxy Omeprazole Sulfide	103876-98-8	C16H17N3O2S	Pharmaceutical TP (Omeprazole)
5-Benzyloxy Omeprazole	1215799-39-5	C23H23N3O3S	Pharmaceutical TP (Omeprazole)
5-Hydroxy Omeprazole	92340-57-3	C17H19N3O4S	Pharmaceutical TP (Omeprazole)
5-O-Desmethyl Omeprazole	151602-49-2	C16H17N3O3S	Pharmaceutical TP (Omeprazole)
5-O-Desmethyl Omeprazole Sulfide	103877-02-7	C16H17N3O2S	Pharmaceutical TP (Omeprazole)
Omeprazole N-Oxide	176219-04-8	C17H19N3O4S	Pharmaceutical TP (Omeprazole)
Omeprazole Sulfide	73590-85-9	C17H19N3O2S	Pharmaceutical TP (Omeprazole)
Omeprazole Sulfone	88546-55-8	C17H19N3O4S	Pharmaceutical TP (Omeprazole)
Omeprazole Sulfone N-Oxide	158812-85-2	C17H19N3O5S	Pharmaceutical TP (Omeprazole)
8-Hydroxy Ondansetron	126671-71-4	C18H19N3O2	Pharmaceutical TP (Ondansetron)
N-Demethyl Ondansetron	99614-14-9	C17H17N3O	Pharmaceutical TP (Ondansetron)

Table S20. Compound database for suspect screening (continued)

Compound Name	CAS	Molecular Formula	Category
N,N-Didemethyl Orphenadrine	17349-96-1	C16H19NO	Pharmaceutical TP (Orphenadrine)
N-Desmethyl Orphenadrine	15301-93-6	C17H21NO	Pharmaceutical TP (Orphenadrine)
Orphenadrine N-Oxide	29215-00-7	C18H23NO2	Pharmaceutical TP (Orphenadrine)
Oseltamivir Carboxylic Acid	187227-45-8	C14H24N2O4	Pharmaceutical TP (Oseltamivir)
4-Hydroxy Ospemifene	128585-01-3	C24H23ClO3	Pharmaceutical TP (Ospemifene)
N-(Demethyl Formate) Oxibendazole	1239340-33-0	C10H13N3O	Pharmaceutical TP (Oxibendazole)
N-Desethyl Oxybutynin	80976-67-6	C20H27NO3	Pharmaceutical TP (Oxybutynin)
Oxybutynin N-Oxide	80976-68-7	C22H31NO4	Pharmaceutical TP (Oxybutynin)
Noroxycodone	57664-96-7	C17H19NO4	Pharmaceutical TP (Oxycodone)
Dehydro Oxymetazoline	227953-47-1	C16H22N2O	Pharmaceutical TP (Oxymetazoline)
Noroxymorphone	33522-95-1	C16H17NO4	Pharmaceutical TP (Oxymorphone)
alpha-Apo-Oxytetracycline	18695-01-7	C22H22N2O8	Pharmaceutical TP (Oxytetracycline)
beta-Apo-Oxytetracycline	18751-99-0	C22H22N2O8	Pharmaceutical TP (Oxytetracycline)
10-Deacetyl-7-xylosyl Paclitaxel	90332-63-1	C50H57NO17	Pharmaceutical TP (Paclitaxel)
10-Desacetyl Paclitaxel	78432-77-6	C45H49NO13	Pharmaceutical TP (Paclitaxel)
2-m-Hydroxy(benzoyl) Paclitaxel	132160-31-7	C47H51NO15	Pharmaceutical TP (Paclitaxel)
3'-p-Hydroxy Paclitaxel	132160-32-8	C47H51NO15	Pharmaceutical TP (Paclitaxel)
6alpha-Hydroxy Paclitaxel	153212-75-0	C47H51NO15	Pharmaceutical TP (Paclitaxel)
3'-Demethyl Papaverine	18694-10-5	C19H19NO4	Pharmaceutical TP (Paliperidone)
4',6-Didemethyl Papaverine	57170-09-9	C18H17NO4	Pharmaceutical TP (Paliperidone)
4'-Demethyl Papaverine	18813-60-0	C19H19NO4	Pharmaceutical TP (Paliperidone)
6-Demethyl Papaverine	18813-63-3	C19H19NO4	Pharmaceutical TP (Paliperidone)
Paliperidone N-Oxide	761460-08-6	C23H27FN4O4	Pharmaceutical TP (Paliperidone)
(6S)-Hydroxy (S,S)-Palonosetron	848074-08-8	C19H24N2O2	Pharmaceutical TP (Palonosetron)
Palonosetron N-Oxide	813425-83-1	C19H24N2O2	Pharmaceutical TP (Palonosetron)
Pantoprazole N-Oxide	953787-60-5	C16H15F2N3O5S	Pharmaceutical TP (Pantoprazole)
Pantoprazole Sulfide	102625-64-9	C16H15F2N3O3S	Pharmaceutical TP (Pantoprazole)
Pantoprazole Sulfone	127780-16-9	C16H15F2N3O5S	Pharmaceutical TP (Pantoprazole)
Pantoprazole Sulfone N-Oxide	953787-55-8	C16H15F2N3O6S	Pharmaceutical TP (Pantoprazole)
4-(4-Fluorophenyl)-3-(4-hydroxy-3-methoxyphenoxymethyl)piperidine	112058-90-9	C19H22FNO3	Pharmaceutical TP (Paroxetine)
4-(4-Fluorophenyl)-3-(4-methoxy-3-hydroxyphenoxymethyl)piperidine	112058-89-6	C19H22FNO3	Pharmaceutical TP (Paroxetine)
Desmethylene Paroxetine	159126-30-4	C18H20FNO3	Pharmaceutical TP (Paroxetine)
N-Desmethyl Paroxol	125224-43-3	C12H16FNO	Pharmaceutical TP (Paroxetine)
S-Methyl-D-penicillamine	29913-84-6	C6H13NO2S	Pharmaceutical TP (Penicillamine)
Pentamidine Amidoxime	130349-07-4	C19H24N4O3	Pharmaceutical TP (Pentamidine)
Pentamidine Diamidoxime	101689-95-6	C19H24N4O4	Pharmaceutical TP (Pentamidine)
1-(3-Carboxypropyl)-3,7-dimethylxanthine	6493-07-8	C11H14N4O4	Pharmaceutical TP (Pentoxifylline)
Hydroxy Pentoxifylline	100324-81-0	C13H20N4O3	Pharmaceutical TP (Pentoxifylline)
N-Desmethyl Perazine	3240-48-0	C19H23N3S	Pharmaceutical TP (Perazine)
Perazine Sulfoxide	20627-44-5	C20H25N3OS	Pharmaceutical TP (Perazine)
N-Despropyl Pergolide	72821-91-1	C16H20N2S	Pharmaceutical TP (Pergolide)
Pergolide Sulfoxide	72822-01-6	C19H26N2OS	Pharmaceutical TP (Pergolide)
Perindoprilat	95153-31-4	C17H28N2O5	Pharmaceutical TP (Perindopril)
7-Hydroxy Perphenazine	52174-38-6	C21H26ClN3O2S	Pharmaceutical TP (Perphenazine)
Perphenazine Sulfoxide	10078-25-8	C21H26ClN3O2S	Pharmaceutical TP (Perphenazine)
2,3,6-Pyridinetriamine	4318-79-0	C5H8N4	Pharmaceutical TP (Phenazopyridine)
2,6-Diamino-5-hydroxy-3-(phenylazo)pyridine	86271-56-9	C11H11N5O	Pharmaceutical TP (Phenazopyridine)
Pheniramine N-Oxide	12656-98-3	C16H20N2O	Pharmaceutical TP (Pheniramine)
N-Hydroxy Phentermine	38473-30-2	C10H15NO	Pharmaceutical TP (Phentermine)
4-Hydroxy Phenylbutazone	16860-43-8	C19H20N2O3	Pharmaceutical TP (Phenylbutazone)
3-Hydroxy Phenytoin	30074-03-4	C15H12N2O3	Pharmaceutical TP (Phenytoin)
N-Desmethyl Mephenytoin	631-07-2	C11H12N2O2	Pharmaceutical TP (Phenytoin)
2-Debenzoyl rac Bopindolol	23869-98-9	C16H24N2O2	Pharmaceutical TP (Pindolol)
1-Hydroxy Pioglitazone	146062-44-4	C19H20N2O4S	Pharmaceutical TP (Pioglitazone)
2-Hydroxy Pioglitazone	101931-00-4	C19H20N2O4S	Pharmaceutical TP (Pioglitazone)
5-[4-[2-[5-(2-Methyl-1,3-dioxolan-2-YL)-2-pyridyl]ethoxy]benzylidene]-2,4-thiazolidinedione	184766-62-9	C21H20N2O5S	Pharmaceutical TP (Pioglitazone)
5-Desethyl 5-Carboxy Pioglitazone	186751-40-6	C18H16N2O5S	Pharmaceutical TP (Pioglitazone)
Carboxy Pioglitazone (M-V)	146062-48-8	C19H18N2O5S	Pharmaceutical TP (Pioglitazone)
Hydroxy Pioglitazone (M-VII)	625853-72-7	C19H20N2O4S	Pharmaceutical TP (Pioglitazone)
Keto Pioglitazone	146062-45-5	C19H18N2O4S	Pharmaceutical TP (Pioglitazone)
Pioglitazone N-Oxide	145350-09-0	C19H20N2O4S	Pharmaceutical TP (Pioglitazone)
N-Desmethyl Pirenzepine	63257-31-8	C18H19N5O2	Pharmaceutical TP (Pirenzepine)
5-Carboxy-N-phenyl-2-1H-pyridone	77837-08-2	C12H9NO3	Pharmaceutical TP (Pirfenidone)
Methyl 5-Carboxy-N-phenyl-2-1H-pyridone	77837-09-3	C13H11NO3	Pharmaceutical TP (Pirfenidone)
N-(4-Fluorophenyl)pyridin-2(1H)-one	60532-42-5	C11H8FNO	Pharmaceutical TP (Pirfenidone)
N-(4-Hydroxyphenyl)-5-methyl-2-1H-pyridone	851518-71-3	C12H11NO2	Pharmaceutical TP (Pirfenidone)
Piribedil N-Oxide	53954-71-5	C16H18N4O3	Pharmaceutical TP (Piribedil)

Table S20. Compound database for suspect screening (continued)

Compound Name	CAS	Molecular Formula	Category
5'-Hydroxy Piroxicam	77459-78-0	C15H13N3O5S	Pharmaceutical TP (Piroxicam)
Despyridyl Piroxicam	24683-25-8	C10H10N2O4S	Pharmaceutical TP (Piroxicam)
8-Hydroxy Pitavastatin	224320-09-6	C25H24FNO5	Pharmaceutical TP (Pitavastatin)
7-Hydroxy Pramipexole	1246818-51-8	C10H17N3OS	Pharmaceutical TP (Pramipexole)
3alpha-Hydroxy Pravastatin	81131-74-0	C23H36O7	Pharmaceutical TP (Pravastatin)
3-Hydroxy Prazepam	18818-61-6	C19H17ClN2O2	Pharmaceutical TP (Prazepam)
4-Hydroxy Praziquantel	134924-71-3	C19H24N2O3	Pharmaceutical TP (Praziquantel)
6beta-Hydroxy Prednisolone	16355-29-6	C21H28O6	Pharmaceutical TP (Prednisolone)
20beta-Hydroxy Prednisone	600-92-0	C21H28O5	Pharmaceutical TP (Prednisone)
4,5-Dihydro Prednisone	103881-93-2	C21H28O5	Pharmaceutical TP (Prednisone)
6alpha-Methyl Prednisone	91523-05-6	C22H28O5	Pharmaceutical TP (Prednisone)
6beta-Hydroxy Prednisone	95283-34-4	C21H26O6	Pharmaceutical TP (Prednisone)
2-Ethyl-2-phenylmalonamide	7206-76-0	C11H14N2O2	Pharmaceutical TP (Primidone)
2-Hydroxy Probenacid	28242-02-6	C13H19NO5S	Pharmaceutical TP (Probenecid)
7-Hydroxy Prochlorperazine	52172-19-7	C20H24ClN3OS	Pharmaceutical TP (Prochlorperazine)
N-Desmethyl Prochlorperazine	40323-85-1	C19H22ClN3S	Pharmaceutical TP (Prochlorperazine)
Prochlorperazine Sulfoxide	10078-27-0	C20H24ClN3OS	Pharmaceutical TP (Prochlorperazine)
11alpha-Hydroxy Progesterone	80-75-1	C21H30O3	Pharmaceutical TP (Progesterone)
11-Keto Progesterone	516-15-4	C21H28O3	Pharmaceutical TP (Progesterone)
15beta-Hydroxy Progesterone	600-72-6	C21H30O3	Pharmaceutical TP (Progesterone)
5beta-Dihydro Progesterone	128-23-4	C21H32O2	Pharmaceutical TP (Progesterone)
6beta-Hydroxy Progesterone	604-19-3	C21H30O3	Pharmaceutical TP (Progesterone)
Dihydro Progesterone	145-14-2	C21H32O2	Pharmaceutical TP (Progesterone)
Norlevomepromazine	108367-20-0	C18H22N2OS	Pharmaceutical TP (Promazine)
N-Demethyl Promethazine	37707-23-6	C16H18N2S	Pharmaceutical TP (Promethazine)
N-Propargyl Phenothiazine	4282-78-4	C15H11NS	Pharmaceutical TP (Promethazine)
Promethazine N-Oxide	81480-39-9	C17H20N2OS	Pharmaceutical TP (Promethazine)
Promethazine Sulfone	13754-56-8	C17H20N2O2S	Pharmaceutical TP (Promethazine)
Promethazine Sulfoxide	7640-51-9	C17H20N2OS	Pharmaceutical TP (Promethazine)
4-Hydroxy Propafenone	86384-09-0	C21H27NO4	Pharmaceutical TP (Propafenone)
5-Hydroxy Propafenone	86384-10-3	C21H27NO4	Pharmaceutical TP (Propafenone)
N-Despropyl Propafenone	86383-21-3	C18H21NO3	Pharmaceutical TP (Propafenone)
Propiverine N-Oxide	111071-96-6	C23H29NO4	Pharmaceutical TP (Propiverine)
4-Hydroxy Propofol	1988-10-9	C12H18O2	Pharmaceutical TP (Propofol)
(S)-4-Methoxy Propranolol	437999-45-6	C17H23NO3	Pharmaceutical TP (Propranolol)
4-Hydroxy Propranolol	10476-53-6	C16H21NO3	Pharmaceutical TP (Propranolol)
5-Hydroxy Propranolol	111691-89-5	C16H21NO3	Pharmaceutical TP (Propranolol)
5-Methoxy Propranolol	345211-68-9	C17H23NO3	Pharmaceutical TP (Propranolol)
7-Hydroxy Propranolol	81907-81-5	C16H21NO3	Pharmaceutical TP (Propranolol)
N-Desisopropyl Propranolol (Norpropranolol)	20862-11-7	C13H15NO2	Pharmaceutical TP (Propranolol)
4-Deoxy Pyridoxine	61-67-6	C8H11NO2	Pharmaceutical TP (Pyridoxine)
7-Hydroxy Quetiapine	139079-39-3	C21H25N3O3S	Pharmaceutical TP (Quetiapine)
7-Hydroxy-N-des{[2-(2-hydroxy)ethoxy]ethyl} Quetiapine	232597-73-8	C17H17N3OS	Pharmaceutical TP (Quetiapine)
Norquetiapine	5747-48-8	C17H17N3S	Pharmaceutical TP (Quetiapine)
Quetiapine N-Oxide	1076199-40-0	C21H25N3O3S	Pharmaceutical TP (Quetiapine)
Quetiapine Sulfoxide	329216-63-9	C21H25N3O3S	Pharmaceutical TP (Quetiapine)
Quinaprilat	82768-85-2	C23H26N2O5	Pharmaceutical TP (Quinapril)
3-Hydroxy Quinidine	53467-23-5	C20H24N2O3	Pharmaceutical TP (Quinidine)
O-Desmethyl Quinidine	70877-75-7	C19H22N2O2	Pharmaceutical TP (Quinidine)
Quinidine N-Oxide	70116-00-6	C20H24N2O3	Pharmaceutical TP (Quinidine)
3-Hydroxy Quinine	78549-61-8	C20H24N2O3	Pharmaceutical TP (Quinine)
O-Desmethyl Quinine	524-63-0	C19H22N2O2	Pharmaceutical TP (Quinine)
4-Desmethoxypropoxyl-4-methoxy Rabeprazole	102804-77-3	C15H15N3O2S	Pharmaceutical TP (Rabeprazole)
Rabeprazole Sulfide	117977-21-6	C18H21N3O2S	Pharmaceutical TP (Rabeprazole)
Rabeprazole Sulfide N-Oxide	924663-40-1	C18H21N3O3S	Pharmaceutical TP (Rabeprazole)
Rabeprazole Sulfone	117976-47-3	C18H21N3O4S	Pharmaceutical TP (Rabeprazole)
4-Hydroxy Ramelteon	1209458-03-6	C8H11BrN2	Pharmaceutical TP (Ramelteon)
Ramiprilat	87269-97-4	C21H28N2O5	Pharmaceutical TP (Ramipril)
Desmethyl Ranitidine	66357-25-3	C12H20N4O3S	Pharmaceutical TP (Ranitidine)
Ranitidine N-Oxide	73857-20-2	C13H22N4O4S	Pharmaceutical TP (Ranitidine)
Ranitidine S-Oxide	73851-70-4	C13H22N4O4S	Pharmaceutical TP (Ranitidine)
Desmethyl Ranolazine	172430-45-4	C23H31N3O4	Pharmaceutical TP (Ranolazine)
N-(2,6-Dimethylphenyl)-2-(piperazin-1-yl)acetamide	5294-61-1	C14H21N3O	Pharmaceutical TP (Ranolazine)
O-Desaryl Ranolazine	172430-46-5	C17H27N3O3	Pharmaceutical TP (Ranolazine)
Ranolazine Bis(N-Oxide)	1246816-00-1	C24H33N3O6	Pharmaceutical TP (Ranolazine)
N-Desmethyl N-Hydroxymethyl Regorafenib	1343498-71-4	C21H15ClF4N4O4	Pharmaceutical TP (Regorafenib)
N-Desmethyl Regorafenib	1343498-72-5	C20H13ClF4N4O3	Pharmaceutical TP (Regorafenib)

Table S20. Compound database for suspect screening (continued)

Compound Name	CAS	Molecular Formula	Category
N-Desmethyl Regorafenib (Pyridine)-N-oxide	835621-12-0	C20H13ClF4N4O4	Pharmaceutical TP (Regorafenib)
Regorafenib (Pyridine)-N-oxide	835621-11-9	C21H15ClF4N4O4	Pharmaceutical TP (Regorafenib)
N-Acetyl Retigabine	229970-68-7	C15H16FN3O	Pharmaceutical TP (Retigabine)
25-O-Deacetyl Rifabutin	100324-63-8	C44H60N4O10	Pharmaceutical TP (Rifabutin)
25-O-Deacetyl-23-O-acetyl Rifabutin	1242076-43-2	C46H62N4O11	Pharmaceutical TP (Rifabutin)
3-Formyl Rifamycin	13292-22-3	C38H47NO13	Pharmaceutical TP (Rifampicin)
Desacetyl Rifampicin	16783-99-6	C41H56N4O11	Pharmaceutical TP (Rifampicin)
N-Desmethyl Rifampicin	13292-45-0	C42H56N4O12	Pharmaceutical TP (Rifampicin)
Rifampicin N-4'-Oxide	125833-03-6	C43H58N4O13	Pharmaceutical TP (Rifampicin)
25-Desacetyl Rifapentin	79039-56-8	C45H62N4O11	Pharmaceutical TP (Rifapentine)
N-Hydroxy Riluzole	179070-90-7	C8H5F3N2O2S	Pharmaceutical TP (Riluzole)
2-(alpha,beta)-Hydroxy Rimantadine	127619-49-2	C12H21NO	Pharmaceutical TP (Rimantadine)
4-(alpha,beta)-Hydroxy Rimantadine	117821-36-0	C12H21NO	Pharmaceutical TP (Rimantadine)
N-Desmethyl Riociguat	625115-52-8	C19H17FN8O2	Pharmaceutical TP (Riociguat)
7-Hydroxy Risperidone	147663-04-5	C23H27FN4O3	Pharmaceutical TP (Risperidone)
Risperidone Isoxazole-N-oxide	1391053-34-1	C23H27FN4O3	Pharmaceutical TP (Risperidone)
Des(isopropylthiazolyl) Ritonavir	176655-57-5	C30H39N5O5S	Pharmaceutical TP (Ritonavir)
Des(isopropylthiazolyl)-N-methyl Ritonavir	959351-57-6	C31H41N5O5S	Pharmaceutical TP (Ritonavir)
Desthiazolylmethyloxycarbonyl Ritonavir	176655-55-3	C32H45N5O3S	Pharmaceutical TP (Ritonavir)
3-[(1S)-1-(Dimethylamino)ethyl]phenol (NAP 226-90)	139306-10-8	C10H15NO	Pharmaceutical TP (Rivastigmine)
N-Desethyl N-Methyl rac-Rivastigmine	25081-93-0	C13H20N2O2	Pharmaceutical TP (Rivastigmine)
Rivastigmine N-Oxide	1369779-37-2	C14H22N2O3	Pharmaceutical TP (Rivastigmine)
N-Desmethyl Rizatriptan	144034-84-4	C14H17N5	Pharmaceutical TP (Rizatriptan)
Rizatriptan N-oxide	260435-42-5	C15H19N5O	Pharmaceutical TP (Rizatriptan)
2-[2-(Dipropylamino)ethyl]-6-nitrophenyl Acetic Acid	720656-64-4	C16H24N2O4	Pharmaceutical TP (Ropinirole)
N-Despropyl Ropinirole	106916-16-9	C13H18N2O	Pharmaceutical TP (Ropinirole)
Ropinirole N-Oxide	1076199-41-1	C16H24N2O2	Pharmaceutical TP (Ropinirole)
3-Nitro Ropivacaine	247061-07-0	C17H25N3O3	Pharmaceutical TP (Ropivacaine)
4-Hydroxy-N-despropyl Ropivacaine	243989-47-1	C14H20N2O2	Pharmaceutical TP (Ropivacaine)
N-Despropyl Ropivacaine	27262-40-4	C14H20N2O	Pharmaceutical TP (Ropivacaine)
Ropivacaine N-Oxide	1391053-59-0	C17H26N2O2	Pharmaceutical TP (Ropivacaine)
5-Hydroxy Rosiglitazone	257883-22-0	C18H19N3O4S	Pharmaceutical TP (Rosiglitazone)
N-Desmethyl Rosiglitazone	257892-31-2	C17H17N3O3S	Pharmaceutical TP (Rosiglitazone)
N-Desmethyl Rosuvastatin	371775-74-5	C21H26FN3O6S	Pharmaceutical TP (Rosuvastatin)
5-Hydroxy Saxagliptin	841302-24-7	C18H25N3O3	Pharmaceutical TP (Saxagliptin)
Aposcopolamine	535-26-2	C17H19NO3	Pharmaceutical TP (Scopolamine)
Scopine	498-45-3	C8H13NO2	Pharmaceutical TP (Scopolamine)
N-Desmethyl Selegiline	18913-84-3	C12H15N	Pharmaceutical TP (Selegiline)
Selegiline N-Oxide	366462-61-5	C13H17NO	Pharmaceutical TP (Selegiline)
4-(S)-1-Des(methylamine)-1-oxo-2-(R,S)-hydroxy Sertraline	124345-10-4	C16H12Cl2O2	Pharmaceutical TP (Sertraline)
N-Desmethyl Sertraline	87857-41-8	C16H15Cl2N	Pharmaceutical TP (Sertraline)
N-Hydroxy Sertraline	124345-07-9	C17H17Cl2NO	Pharmaceutical TP (Sertraline)
N-Desmethyl Sibutramine	168835-59-4	C16H24ClN	Pharmaceutical TP (Sibutramine)
N-Didesmethyl Sibutramine	84467-54-9	C15H22ClN	Pharmaceutical TP (Sibutramine)
Desethyl Sildenafil	139755-91-2	C20H26N6O4S	Pharmaceutical TP (Sildenafil)
N-Desmethyl Sildenafil	139755-82-1	C21H28N6O4S	Pharmaceutical TP (Sildenafil)
3'-Hydroxy Simvastatin	126313-98-2	C25H38O6	Pharmaceutical TP (Simvastatin)
6'-Carboxy Simvastatin	114883-30-6	C25H36O7	Pharmaceutical TP (Simvastatin)
6'-Hydroxymethyl Simvastatin	114883-29-3	C25H38O6	Pharmaceutical TP (Simvastatin)
Simvastatin Acid	121009-77-6	C25H40O6	Pharmaceutical TP (Simvastatin)
Solifenacin N-Oxide	180272-28-0	C23H26N2O3	Pharmaceutical TP (Solifenacin)
Sorafenib N-Oxide	583840-03-3	C21H16ClF3N4O4	Pharmaceutical TP (Sorafenib)
6beta-Hydroxy-7alpha-thiomethyl Spironolactone	42219-60-3	C23H32O4S	Pharmaceutical TP (Spironolactone)
7alpha-Thiomethyl Spironolactone	38753-77-4	C23H32O3S	Pharmaceutical TP (Spironolactone)
Deacetyl Spironolactone	38753-76-3	C22H30O3S	Pharmaceutical TP (Spironolactone)
16beta-Hydroxy Stanozolol	125590-76-3	C21H32N2O2	Pharmaceutical TP (Stanozolol)
3'-Hydroxy Stanozolol	125709-39-9	C21H32N2O2	Pharmaceutical TP (Stanozolol)
N4-Acetyl Sulfadiazine	127-74-2	C12H12N4O3S	Pharmaceutical TP (Sulfadiazine)
N4-Acetyl Sulfadimethoxine	24341-30-8	C14H16N4O5S	Pharmaceutical TP (Sulfadimethoxine)
N4-Acetyl Sulfamerazine	127-73-1	C13H14N4O3S	Pharmaceutical TP (Sulfamerazine)
N4-Acetyl Sulfamethazine	100-90-3	C14H16N4O3S	Pharmaceutical TP (Sulfamethazine)
4-Nitro Sulfamethoxazole	29699-89-6	C10H9N3O5S	Pharmaceutical TP (Sulfamethoxazole)
4-Nitroso Sulfamethoxazole	131549-85-4	C10H9N3O4S	Pharmaceutical TP (Sulfamethoxazole)
N4-Acetyl Sulfamethoxazole	21312-10-7	C12H13N3O4S	Pharmaceutical TP (Sulfamethoxazole)
Sulfamethoxazole Hydroxylamine	114438-33-4	C10H11N3O4S	Pharmaceutical TP (Sulfamethoxazole)
N4-Acetyl Sulfapyridine	19077-98-6	C13H13N3O3S	Pharmaceutical TP (Sulfapyridine, Sulfasalazine)
N4-Acetyl Sulfathiazole	127-76-4	C11H11N3O3S2	Pharmaceutical TP (Sulfathiazole)

Table S20. Compound database for suspect screening (continued)

Compound Name	CAS	Molecular Formula	Category
2-Methoxy-5-sulfamoylbenzamide	52395-25-2	C8H10N2O4S	Pharmaceutical TP (Sulpiride)
N,N-Dimethyl Sunitinib	326914-17-4	C20H23FN4O2	Pharmaceutical TP (Sunitinib)
N-Desethyl Sunitinib	356068-97-8	C20H23FN4O2	Pharmaceutical TP (Sunitinib)
Sunitinib N-Oxide	356068-99-0	C22H27FN4O3	Pharmaceutical TP (Sunitinib)
N-Desmethyl-N-cyclopentyl Tadalafil	171596-32-0	C26H25N3O4	Pharmaceutical TP (Tadalafil)
4'-Hydroxy Tamoxifen	82413-23-8	C26H29NO2	Pharmaceutical TP (Tamoxifen)
4-Hydroxy-N-desmethyl Tamoxifen	112093-28-4	C25H27NO2	Pharmaceutical TP (Tamoxifen)
Afimoxifene	174592-47-3	C26H29NO2	Pharmaceutical TP (Tamoxifen)
alpha-Hydroxy Tamoxifen	97151-02-5	C26H29NO2	Pharmaceutical TP (Tamoxifen)
N-Desmethyl Tamoxifen	31750-48-8	C25H27NO	Pharmaceutical TP (Tamoxifen)
N-Desmethyl-4'-hydroxy Tamoxifen	170171-12-7	C25H27NO2	Pharmaceutical TP (Tamoxifen)
Tamoxifen N-Oxide	75504-34-6	C26H29NO2	Pharmaceutical TP (Tamoxifen)
N-Desmethyl Tapentadol	1300037-83-5	C13H21NO	Pharmaceutical TP (Tapentadol)
Tapentadol N-Oxide	1346601-17-9	C14H23NO2	Pharmaceutical TP (Tapentadol)
Tazarotenic Acid	118292-41-4	C19H17NO2S	Pharmaceutical TP (Tazarotene)
5-(3-Methyl-1-triazeno)imidazole-4-carboxamide (MTIC)	3413-72-7	C5H8N6O	Pharmaceutical TP (Temozolomide)
5-Aminoimidazole-4-carboxamide	360-97-4	C4H6N4O	Pharmaceutical TP (Temozolomide)
Carboxy Terbinafine	99473-14-0	C21H23NO2	Pharmaceutical TP (Terbinafine)
Hydroxy Terbinafine	162227-13-6	C21H25NO	Pharmaceutical TP (Terbinafine)
N-Desmethyl Terbinafine	99473-11-7	C20H23N	Pharmaceutical TP (Terbinafine)
N-Desmethylcarboxy Terbinafine	99473-15-1	C20H21NO2	Pharmaceutical TP (Terbinafine)
19-Hydroxy Testosterone	2126-37-6	C19H28O3	Pharmaceutical TP (Testosterone)
Androsterone	53-41-8	C19H30O2	Pharmaceutical TP (Testosterone)
Hydroxy Tetrabenazine	3466-75-9	C19H29NO3	Pharmaceutical TP (Tetrabenazine)
Anhydrotetracycline	1665-56-1	C22H22N2O7	Pharmaceutical TP (Tetracycline)
11-Nor-delta(9)-tetrahydrocannabinol-9-carboxylic Acid (11-nor-9-Carboxy-THC)	64280-14-4	C21H28O4	Pharmaceutical TP (delta(9)-Tetrahydrocannabinol)
11-Hydroxy-delta(9)-tetrahydrocannabinol (11-OH-THC)	36557-05-8	C21H30O3	Pharmaceutical TP (delta(9)-Tetrahydrocannabinol)
5'-Hydroxy Thalidomide	222991-42-6	C13H10N2O5	Pharmaceutical TP (Thalidomide)
6-Amino-5-1,3-dimethyl-5-(formamido)uracil	7597-60-6	C7H10N4O3	Pharmaceutical TP (Theophylline)
Proxyphylline	603-00-9	C10H14N4O3	Pharmaceutical TP (Theophylline)
Thioridazine 2-Sulfone	14759-06-9	C21H26N2O2S2	Pharmaceutical TP (Thioridazine)
Thioridazine 5-Sulfoxide	7776-05-8	C21H26N2OS2	Pharmaceutical TP (Thioridazine)
2-(2-Chlorobenzamido)acetic Acid	16555-60-5	C9H8ClNO3	Pharmaceutical TP (Ticlopidine)
Nortilidine	38677-94-0	C16H21NO2	Pharmaceutical TP (Tilidine)
S-Methyl Tiopronin	87254-91-9	C6H11NO3S	Pharmaceutical TP (Tiopronin)
Dehydro Tizanidine	125292-34-4	C9H6ClN5S	Pharmaceutical TP (Tizanidine)
Hydroxy Tizanidine	125292-31-1	C9H8ClN5OS	Pharmaceutical TP (Tizanidine)
4-Carboxy Tolbutamide	2224-10-4	C12H16N2O5S	Pharmaceutical TP (Tolbutamide)
4-Hydroxy Tolbutamide	5719-85-7	C12H18N2O4S	Pharmaceutical TP (Tolbutamide)
3-O-Methyl Tolcapone	134612-80-9	C15H13NO5	Pharmaceutical TP (Tolcapone)
3-Hydroxy Tolperisone	59303-39-8	C16H23NO2	Pharmaceutical TP (Tolperisone)
Carboxy Tolperisone	59303-40-1	C16H21NO3	Pharmaceutical TP (Tolperisone)
Hydroxymethyl Tolperisone	59303-37-6	C17H25NO2	Pharmaceutical TP (Tolperisone)
5-Carboxy Desisopropyl Tolterodine	214601-13-5	C19H23NO3	Pharmaceutical TP (Tolterodine)
5-Carboxy Tolterodine	1076199-77-3	C22H29NO3	Pharmaceutical TP (Tolterodine)
5-Hydroxymethyl Tolterodine	200801-70-3	C22H31NO2	Pharmaceutical TP (Tolterodine)
Didesisopropyl Tolterodine	1189501-90-3	C16H19NO	Pharmaceutical TP (Tolterodine)
Toltrazuril Sulfoxide	69004-15-5	C18H14F3N3O5S	Pharmaceutical TP (Toltrazuril)
10-Hydroxy Topiramate	198215-60-0	C12H21NO9S	Pharmaceutical TP (Topiramate)
2,3-Desisopropylidene Topiramate	851957-35-2	C9H17NO8S	Pharmaceutical TP (Topiramate)
9-Hydroxy Topiramate	198215-62-2	C12H21NO9S	Pharmaceutical TP (Topiramate)
N-Desmethyl Topotecan	190710-79-3	C22H21N3O5	Pharmaceutical TP (Topotecan)
4'-Hydroxy Toremifene	352233-94-4	C26H28ClNO2	Pharmaceutical TP (Toremifene)
N-Desmethyl 4-Hydroxy Toremifene	125618-41-9	C25H26ClNO2	Pharmaceutical TP (Toremifene)
N-Desmethyl Toremifene	110503-61-2	C25H26ClNO	Pharmaceutical TP (Toremifene)
Toremifene N-Oxide	163130-29-8	C26H28ClNO2	Pharmaceutical TP (Toremifene)
4'-Hydroxy Torsemide	99300-68-2	C16H20N4O4S	Pharmaceutical TP (Torsemide)
Torsemide Carboxylic Acid	113844-99-8	C16H18N4O5S	Pharmaceutical TP (Torsemide)
N,O-Didesmethyl Tramadol	138853-73-3	C14H21NO2	Pharmaceutical TP (Tramadol)
N-Bisdesmethyl Tramadol	931115-27-4	C14H21NO2	Pharmaceutical TP (Tramadol)
N-Desmethyl Tramadol	73806-55-0	C15H23NO2	Pharmaceutical TP (Tramadol)
O-Desmethyl Tramadol	73986-53-5	C15H23NO2	Pharmaceutical TP (Tramadol)
Tramadol N-Oxide	147441-56-3	C16H25NO3	Pharmaceutical TP (Tramadol)
Trandolaprilat	87679-71-8	C22H30N2O5	Pharmaceutical TP (Trandolapril)
4'-Hydroxy Trazodone	53818-10-3	C19H22ClN5O2	Pharmaceutical TP (Trazodone)
Trazodone N-Oxide	55290-68-1	C19H22ClN5O2	Pharmaceutical TP (Trazodone)
6beta-Hydroxy Triamcinolone Acetonide	3869-32-7	C24H31FO7	Pharmaceutical TP (Triamcinolone Acetonide)

Table S20. Compound database for suspect screening (continued)

Compound Name	CAS	Molecular Formula	Category
4-Hydroxy Triamterene	1226-52-4	C12H11N7O	Pharmaceutical TP (Triamterene)
Keto Triclabendazole	1201920-88-8	C13H7Cl3N2O2	Pharmaceutical TP (Triclabendazole)
Triclabendazole Sulfone	106791-37-1	C14H9Cl3N2O3S	Pharmaceutical TP (Triclabendazole)
Triclabendazole Sulfoxide	100648-13-3	C14H9Cl3N2O2S	Pharmaceutical TP (Triclabendazole)
N-Desmethyl Trifluoperazine	2804-16-2	C20H22F3N3S	Pharmaceutical TP (Trifluoperazine, Fluphenazine)
alpha-Hydroxy Trimethoprim	29606-06-2	C14H18N4O4	Pharmaceutical TP (Trimethoprim)
3-O-Demethyl Trimethoprim	27653-69-6	C13H16N4O3	Pharmaceutical TP (Trimethoprim)
Keto Trimethoprim	30806-86-1	C14H16N4O4	Pharmaceutical TP (Trimethoprim)
2-Hydroxy Trimipramine	2064-15-5	C20H26N2O	Pharmaceutical TP (Trimipramine)
2-Hydroxy-N-desmethyl Trimipramine	2064-14-4	C19H24N2O	Pharmaceutical TP (Trimipramine)
Trimipramine N-Oxide	14171-70-1	C20H26N2O	Pharmaceutical TP (Trimipramine)
N,N-Didesmethyl Ulipristal	244206-52-8	C26H31NO3	Pharmaceutical TP (Ulipristal)
N-Desmethyl Ulipristal	159681-67-1	C27H33NO3	Pharmaceutical TP (Ulipristal)
N-Formyl Valacyclovir	847670-62-6	C14H20N6O5	Pharmaceutical TP (Valacyclovir)
2-Propylglutaric Acid	32806-62-5	C8H14O4	Pharmaceutical TP (Valproic Acid)
3-Hydroxy Valproic Acid	58888-84-9	C8H16O3	Pharmaceutical TP (Valproic Acid)
3-Keto Valproic Acid	60113-81-7	C8H14O3	Pharmaceutical TP (Valproic Acid)
4-Hydroxy Valproic acid	60113-82-8	C8H16O3	Pharmaceutical TP (Valproic Acid)
5-Hydroxy Valproic Acid	53660-23-4	C8H16O3	Pharmaceutical TP (Valproic Acid)
4-Hydroxy Valsartan	188259-69-0	C24H29N5O4	Pharmaceutical TP (Valsartan)
Valsartan Acid	164265-78-5	C14H10N4O2	Pharmaceutical TP (Valsartan, Losartan, Candesartan, Irbesartan)
N-Demethyl Vandetanib	338992-12-4	C21H22BrFN4O2	Pharmaceutical TP (Vandetanib)
O-Demethyl Vandetanib	910298-60-1	C21H22BrFN4O2	Pharmaceutical TP (Vandetanib)
Hydroxy Vardenafil	224785-98-2	C23H32N6O5S	Pharmaceutical TP (Vardenafil)
N-Desethyl Vardenafil	448184-46-1	C21H28N6O4S	Pharmaceutical TP (Vardenafil)
Hydroxy Varenicline	357424-21-6	C13H13N3O	Pharmaceutical TP (Varenicline)
N-Formyl Varenicline	796865-82-2	C14H13N3O	Pharmaceutical TP (Varenicline)
Varenicline Lactam	873302-30-8	C13H11N3O	Pharmaceutical TP (Varenicline)
N,N,O-Tridesmethyl Venlafaxine	149289-29-2	C14H21NO2	Pharmaceutical TP (Venlafaxine)
N,N-Didesmethyl Venlafaxine	93413-77-5	C15H23NO2	Pharmaceutical TP (Venlafaxine)
N,O-Didesmethyl Venlafaxine	135308-74-6	C15H23NO2	Pharmaceutical TP (Venlafaxine)
N-Desmethyl Venlafaxine	149289-30-5	C16H25NO2	Pharmaceutical TP (Venlafaxine)
Venlafaxine N-Oxide	1094598-37-4	C17H27NO3	Pharmaceutical TP (Venlafaxine)
3-(3,4-Dimethoxyphenyl)-2-methyl-6-methylaminohexane-3-carbonitrile (D617)	34245-14-2	C17H26N2O2	Pharmaceutical TP (Verapamil)
Norverapamil	67018-85-3	C26H36N2O4	Pharmaceutical TP (Verapamil)
p-O-Desmethyl Verapamil	77326-93-3	C26H36N2O4	Pharmaceutical TP (Verapamil)
Vilazodone Carboxylic Acid	163521-19-5	C26H26N4O3	Pharmaceutical TP (Vilazodone)
Vildagliptin Carboxylic Acid	565453-40-9	C17H26N2O4	Pharmaceutical TP (Vildagliptin)
4-Desacetyl 3-Deoxy Vincristine	99435-53-7	C44H54N4O8	Pharmaceutical TP (Vincristine)
4-Desacetyl Vincristine	3704-01-6	C44H54N4O9	Pharmaceutical TP (Vincristine)
N-Desformyl-4-desacetyl Vincristine	55324-83-9	C43H54N4O8	Pharmaceutical TP (Vincristine)
Deacetyl Vinorelbine	126347-74-8	C43H52N4O7	Pharmaceutical TP (Vinorelbine)
4-Hydroxy Voriconazole	943331-64-4	C16H14F3N5O2	Pharmaceutical TP (Voriconazole)
Voriconazole N-Oxide	618109-05-0	C16H14F3N5O2	Pharmaceutical TP (Voriconazole)
Succinanilic Acid	102-14-7	C10H11NO3	Pharmaceutical TP (Vorinostat)
10-Hydroxy Warfarin	83219-99-2	C19H16O5	Pharmaceutical TP (Warfarin)
3'-Hydroxy Warfarin	30992-81-5	C19H16O5	Pharmaceutical TP (Warfarin)
4'-Hydroxy Warfarin	24579-14-4	C19H16O5	Pharmaceutical TP (Warfarin)
6-Benzyloxy Warfarin	30992-68-8	C26H22O5	Pharmaceutical TP (Warfarin)
6-Hydroxy Warfarin	17834-02-5	C19H16O5	Pharmaceutical TP (Warfarin)
7-Hydroxy Warfarin	63740-81-8	C19H16O5	Pharmaceutical TP (Warfarin)
8-Benzyloxy Warfarin	32492-96-9	C26H22O5	Pharmaceutical TP (Warfarin)
8-Hydroxy Warfarin	17834-04-7	C19H16O5	Pharmaceutical TP (Warfarin)
Dehydro Warfarin	67588-18-5	C19H14O4	Pharmaceutical TP (Warfarin)
3-Hydroxy Xylazine	145356-33-8	C12H16N2OS	Pharmaceutical TP (Xylazine)
4-Hydroxy Xylazine	145356-32-7	C12H16N2OS	Pharmaceutical TP (Xylazine)
5-Oxo Zaleplon	159225-99-7	C17H15N5O2	Pharmaceutical TP (Zaleplon)
N-[3-(3-Cyanopyrazolo[1,5-a]pyrimidin-7-yl)phenyl]acetamide	115931-01-6	C15H11N5O	Pharmaceutical TP (Zaleplon)
Zileuton Sulfoxide	1147524-83-1	C11H12N2O3S	Pharmaceutical TP (Zileuton)
S-Methyldihydro Ziprasidone	194280-91-6	C22H25ClN4OS	Pharmaceutical TP (Ziprasidone)
Ziprasidone Sulfoxide	188797-80-0	C21H21ClN4O2S	Pharmaceutical TP (Ziprasidone)
8-Demethyl Zolazepam	31271-94-0	C14H13FN4O	Pharmaceutical TP (Zolazepam)
Didesmethyl Zolmitriptan	139264-15-6	C14H17N3O2	Pharmaceutical TP (Zolmitriptan)
N-Desmethyl Zolmitriptan	139264-35-0	C15H19N3O2	Pharmaceutical TP (Zolmitriptan)
Zolmitriptan N-Oxide	251451-30-6	C16H21N3O3	Pharmaceutical TP (Zolmitriptan)
Zolpidem 6-Carboxylic Acid	109461-15-6	C19H19N3O3	Pharmaceutical TP (Zolpidem)
Zolpidem Phenyl-4-carboxylic Acid	109461-65-6	C19H19N3O3	Pharmaceutical TP (Zolpidem)

Table S20. Compound database for suspect screening (continued)

Compound Name	CAS	Molecular Formula	Category
N-Acetyl Zonisamide	68936-43-6	C10H10N2O4S	Pharmaceutical TP (Zonisamide)
N-Methyl Zonisamide	68292-02-4	C9H10N2O3S	Pharmaceutical TP (Zonisamide)
Deschloro-zopiclone	1348046-61-6	C17H18N6O3	Pharmaceutical TP (Zopiclone)
N-Desmethyl Zopiclone	59878-63-6	C16H15ClN6O3	Pharmaceutical TP (Zopiclone)
Zopiclone N-Oxide	43200-96-0	C17H17ClN6O4	Pharmaceutical TP (Zopiclone)
Cybutryne	28159-98-0	C11H19N5S	Pesticide (Algaecide)
Terbuthylazine	5915-41-3	C9H16ClN5	Pesticide (Algaecide, Herbicide, Microbiocide)
Thidiazuron	51707-55-2	C9H8N4OS	Pesticide (Defoliant, Plant Growth Regulator)
Tribufos	78-48-8	C12H27OPS3	Pesticide (Defoliant, Plant Growth Regulator)
Dazomet	533-74-4	C5H10N2S2	Pesticide (Fumigant, Fungicide, Nematicide)
2-Methyl-1-naphthaleneacetamide	85-07-4	C13H13NO	Pesticide (Fungicide)
Acibenzolar-S-methyl	135158-54-2	C8H6N2OS2	Pesticide (Fungicide)
Ametoctradin	865318-97-4	C15H25N5	Pesticide (Fungicide)
Amisulbrom	348635-87-0	C13H13BrFN5O4S2	Pesticide (Fungicide)
Azoxystrobin	131860-33-8	C22H17N3O5	Pesticide (Fungicide)
Benalaxyl	71626-11-4	C20H23NO3	Pesticide (Fungicide)
Benomyl	17804-35-2	C14H18N4O3	Pesticide (Fungicide)
Benthiavalicarb-isopropyl	177406-68-7	C18H24FN3O3S	Pesticide (Fungicide)
Benzovindiflupyr	1072957-71-1	C18H15Cl2F2N3O	Pesticide (Fungicide)
Bitertanol	55179-31-2	C20H23N3O2	Pesticide (Fungicide)
Bixafen	581809-46-3	C18H12Cl2F3N3O	Pesticide (Fungicide)
Boscalid	188425-85-6	C18H12Cl2N2O	Pesticide (Fungicide)
Bromuconazole	116255-48-2	C13H12BrCl2N3O	Pesticide (Fungicide)
Bupirimate	41483-43-6	C13H24N4O3S	Pesticide (Fungicide)
Captan	133-06-2	C9H8Cl3NO2S	Pesticide (Fungicide)
Carboxin	5234-68-4	C12H13NO2S	Pesticide (Fungicide)
Carpropamid	104030-54-8	C15H18Cl3NO	Pesticide (Fungicide)
Chloroneb	2675-77-6	C8H8Cl2O2	Pesticide (Fungicide)
Chlorothalonil	1897-45-6	C8Cl4N2	Pesticide (Fungicide)
Climbazole	38083-17-9	C15H17ClN2O2	Pesticide (Fungicide)
Cyazofamid	120116-88-3	C13H13ClN4O2S	Pesticide (Fungicide)
Cyflufenamid	180409-60-3	C20H17F5N2O2	Pesticide (Fungicide)
Cymoxanil	57966-95-7	C7H10N4O3	Pesticide (Fungicide)
Cyproconazole	94361-06-5	C15H18ClN3O	Pesticide (Fungicide)
Cyprodinil	121552-61-2	C14H15N3	Pesticide (Fungicide)
Debacarb (2-EEEBC)	62732-91-6	C14H19N3O4	Pesticide (Fungicide)
Dicloran	99-30-9	C6H4Cl2N2O2	Pesticide (Fungicide)
Difenoconazole	119446-68-3	C19H17Cl2N3O3	Pesticide (Fungicide)
Dimethomorph	110488-70-5	C21H22ClNO4	Pesticide (Fungicide)
Dimoxystrobin	149961-52-4	C19H22N2O3	Pesticide (Fungicide)
Diniconazole	83657-24-3	C15H17Cl2N3O	Pesticide (Fungicide)
Dithianon	3347-22-6	C14H4N2O2S2	Pesticide (Fungicide)
Dodemorph	1593-77-7	C18H35NO	Pesticide (Fungicide)
Edifenphos	17109-49-8	C14H15O2PS2	Pesticide (Fungicide)
Enilconazole (Imazalil)	35554-44-0	C14H14Cl2N2O	Pesticide (Fungicide)
Epoxiconazole	133855-98-8	C17H13ClFN3O	Pesticide (Fungicide)
Etridiazole	2593-15-9	C5H5Cl3N2OS	Pesticide (Fungicide)
Famoxadone	131807-57-3	C22H18N2O4	Pesticide (Fungicide)
Fenamidone	161326-34-7	C17H17N3OS	Pesticide (Fungicide)
Fenarimol	60168-88-9	C17H12Cl2N2O	Pesticide (Fungicide)
Fenbuconazole	114369-43-6	C19H17ClN4	Pesticide (Fungicide)
Fenhexamid	126833-17-8	C14H17Cl2NO2	Pesticide (Fungicide)
Fenoxanil	115852-48-7	C15H18Cl2N2O2	Pesticide (Fungicide)
Fenpiclonil	74738-17-3	C11H6Cl2N2	Pesticide (Fungicide)
Fenpropidin	67306-00-7	C19H31N	Pesticide (Fungicide)
Fenpropimorph	67564-91-4	C20H33NO	Pesticide (Fungicide)
Fluazinam	79622-59-6	C13H4Cl2F6N4O4	Pesticide (Fungicide)
Fludioxonil	131341-86-1	C12H6F2N2O2	Pesticide (Fungicide)
Fluopicolide	239110-15-7	C14H8Cl3F3N2O	Pesticide (Fungicide)
Fluopyram	658066-35-4	C16H11ClF6N2O	Pesticide (Fungicide)
Fluoxastrobin	361377-29-9	C21H16ClFN4O5	Pesticide (Fungicide)
Fluquinconazole	136426-54-5	C16H8Cl2FN5O	Pesticide (Fungicide)
Flutolanil	66332-96-5	C17H16F3NO2	Pesticide (Fungicide)
Flutriafol	76674-21-0	C16H13F2N3O	Pesticide (Fungicide)
Fluxapyroxad	907204-31-3	C18H12F5N3O	Pesticide (Fungicide)
Folpet	133-07-3	C9H4Cl3NO2S	Pesticide (Fungicide)
Fuberidazole	3878-19-1	C11H8N2O	Pesticide (Fungicide)

Table S20. Compound database for suspect screening (continued)

Compound Name	CAS	Molecular Formula	Category
Furalaxyl	57646-30-7	C17H19NO4	Pesticide (Fungicide)
Furilazole	121776-33-8	C11H13Cl2NO3	Pesticide (Fungicide)
Hexaconazole	79983-71-4	C14H17Cl2N3O	Pesticide (Fungicide)
Imibenconazole	86598-92-7	C17H13Cl3N4S	Pesticide (Fungicide)
Iminoctadine	13516-27-3	C18H41N7	Pesticide (Fungicide)
Ipconazole	125225-28-7	C18H24ClN3O	Pesticide (Fungicide)
Iprobenfos	26087-47-8	C13H21O3PS	Pesticide (Fungicide)
Iprodione	36734-19-7	C13H13Cl2N3O3	Pesticide (Fungicide)
Iprovalicarb	140923-17-7	C18H28N2O3	Pesticide (Fungicide)
Isopyrazam	881685-58-1	C20H23F2N3O	Pesticide (Fungicide)
Kresoxim-methyl	143390-89-0	C18H19NO4	Pesticide (Fungicide)
Mandipropamid	374726-62-2	C23H22ClNO4	Pesticide (Fungicide)
Mepanipyrim	110235-47-7	C14H13N3	Pesticide (Fungicide)
Metalaxyl	57837-19-1	C15H21NO4	Pesticide (Fungicide)
Metconazole	125116-23-6	C17H22ClN3O	Pesticide (Fungicide)
Metominostrobin	133408-50-1	C16H16N2O3	Pesticide (Fungicide)
Metrafenone	220899-03-6	C19H21BrO5	Pesticide (Fungicide)
Myclobutanil	88671-89-0	C15H17ClN4	Pesticide (Fungicide)
Nitrothal-isopropyl	10552-74-6	C14H17NO6	Pesticide (Fungicide)
Ofurace	58810-48-3	C14H16ClNO3	Pesticide (Fungicide)
Oxadixyl	77732-09-3	C14H18N2O4	Pesticide (Fungicide)
Oxathiapiprolin	1003318-67-9	C24H22F5N5O2S	Pesticide (Fungicide)
Penconazole	66246-88-6	C13H15Cl2N3	Pesticide (Fungicide)
Pencycuron	66063-05-6	C19H21ClN2O	Pesticide (Fungicide)
Penthiopyrad	183675-82-3	C16H20F3N3OS	Pesticide (Fungicide)
Picoxystrobin	117428-22-5	C18H16F3NO4	Pesticide (Fungicide)
Piperalin	3478-94-2	C16H21Cl2NO2	Pesticide (Fungicide)
Probenazole	27605-76-1	C10H9NO3S	Pesticide (Fungicide)
Prochloraz	67747-09-5	C15H16Cl3N3O2	Pesticide (Fungicide)
Procymidone	32809-16-8	C13H11Cl2NO2	Pesticide (Fungicide)
Propamocarb	24579-73-5	C9H20N2O2	Pesticide (Fungicide)
Propiconazole	60207-90-1	C15H17Cl2N3O2	Pesticide (Fungicide)
Proquinazid	189278-12-4	C14H17IN2O2	Pesticide (Fungicide)
Prothioconazole	178928-70-6	C14H15Cl2N3OS	Pesticide (Fungicide)
Pyracarbolid	24691-76-7	C13H15NO2	Pesticide (Fungicide)
Pyraclostrobin	175013-18-0	C19H18ClN3O4	Pesticide (Fungicide)
Pyrazophos	13457-18-6	C14H20N3O5PS	Pesticide (Fungicide)
Pyrifenox	88283-41-4	C14H12Cl2N2O	Pesticide (Fungicide)
Pyrimethanil	53112-28-0	C12H13N3	Pesticide (Fungicide)
Pyroquilone	57369-32-1	C11H11NO	Pesticide (Fungicide)
Quinoxyfen	878790-59-1	C15H8Cl2FNO	Pesticide (Fungicide)
Sedaxane	874967-67-6	C18H19F2N3O	Pesticide (Fungicide)
Spiroxamine	118134-30-8	C18H35NO2	Pesticide (Fungicide)
Tebuconazole	107534-96-3	C16H22ClN3O	Pesticide (Fungicide)
Tetraconazole	112281-77-3	C13H11Cl2F4N3O	Pesticide (Fungicide)
Thiabendazole	148-79-8	C10H7N3S	Pesticide (Fungicide)
Thifluzamide	130000-40-7	C13H6Br2F6N2O2S	Pesticide (Fungicide)
Thiophanate	23564-06-9	C14H18N4O4S2	Pesticide (Fungicide)
Thiophanate-methyl	23564-05-8	C12H14N4O4S2	Pesticide (Fungicide)
Tiadinil	223580-51-6	C11H10ClN3OS	Pesticide (Fungicide)
Tolclofos-methyl	57018-04-9	C9H11Cl2O3PS	Pesticide (Fungicide)
Triadimenol	55219-65-3	C14H18ClN3O2	Pesticide (Fungicide)
Triazoxide	72459-58-6	C10H6ClN5O	Pesticide (Fungicide)
Tricyclazole	41814-78-2	C9H7N3S	Pesticide (Fungicide)
Tridemorph	24602-86-6	C19H39NO	Pesticide (Fungicide)
Trifloxystrobin	141517-21-7	C20H19F3N2O4	Pesticide (Fungicide)
Triflumizole	68694-11-1	C15H15ClF3N3O	Pesticide (Fungicide)
Triticonazole	131983-72-7	C17H20ClN3O	Pesticide (Fungicide)
Uniconazole	83657-22-1	C15H18ClN3O	Pesticide (Fungicide)
Vinclozolin	50471-44-8	C12H9Cl2NO3	Pesticide (Fungicide)
Zoxamide	156052-68-5	C14H16Cl3NO2	Pesticide (Fungicide)
Oxycarboxin	5259-88-1	C12H13NO4S	Pesticide (Fungicide) / Pesticide TP (Carboxin)
Carbendazim	10605-21-7	C9H9N3O2	Pesticide (Fungicide) / Pesticide TP (Thiophanate-methyl)
Triadimefon	43121-43-3	C14H16ClN3O2	Pesticide (Fungicide) / Pesticide TP (Triadimenol A)
Dichlofluanid	1085-98-9	C9H11Cl2FN2O2S2	Pesticide (Fungicide, Insecticide)
Dinocap	39300-45-3	C18H24N2O6	Pesticide (Fungicide, Insecticide)
Tolylfluanid	731-27-1	C10H13Cl2FN2O2S2	Pesticide (Fungicide, Insecticide)

Table S20. Compound database for suspect screening (continued)

Compound Name	CAS	Molecular Formula	Category
Triforine	26644-46-2	C10H14Cl6N4O2	Pesticide (Fungicide, Insecticide)
Salicylanilide	87-17-2	C13H11NO2	Pesticide (Fungicide, Microbiocide)
Pentachloronitrobenzene (PCNB)	82-68-8	C6Cl5NO2	Pesticide (Fungicide, Nematicide, Algaecide)
Ethoxyquin	91-53-2	C14H19NO	Pesticide (Fungicide, Plant Growth Regulator)
Tecnazene	117-18-0	C6HCl4NO2	Pesticide (Fungicide, Plant Growth Regulator)
Iodopropynyl Butyl Carbamate (IPBC)	55406-53-6	C8H12INO2	Pesticide (Fungicide, Wood Preservative)
4-(Dichloroacetyl)-1-oxa-4-azaspiro(4.5)decane (AD-67)	71526-07-3	C10H15Cl2NO2	Pesticide (Herbicide Safener)
Benoxacor	98730-04-2	C11H11Cl2NO2	Pesticide (Herbicide Safener)
Cloquintocet-mexyl	99607-70-2	C18H22ClNO3	Pesticide (Herbicide Safener)
Cyometrinil	63278-33-1	C10H7N3O	Pesticide (Herbicide Safener)
Cyprosulfamide	221667-31-8	C18H18N2O5S	Pesticide (Herbicide Safener)
Dichlormid	37764-25-3	C8H11Cl2NO	Pesticide (Herbicide Safener)
Fenchlorazole-ethyl	103112-35-2	C12H8Cl5N3O2	Pesticide (Herbicide Safener)
Fenclorim	3740-92-9	C10H6Cl2N2	Pesticide (Herbicide Safener)
Flurazole	72850-64-7	C12H7ClF3NO2S	Pesticide (Herbicide Safener)
Fluxofenim	88485-37-4	C12H11ClF3NO3	Pesticide (Herbicide Safener)
Isoxadifen-ethyl	163520-33-0	C18H17NO3	Pesticide (Herbicide Safener)
Mefenpyr-diethyl	135590-91-9	C16H18Cl2N2O4	Pesticide (Herbicide Safener)
Oxabetrinil	74782-23-3	C12H12N2O3	Pesticide (Herbicide Safener)
(2,4,5-Trichlorophenoxy)acetic Acid 2-Butoxyethyl Ester (2,4,5-T-Butotyl)	2545-59-7	C14H17Cl3O4	Pesticide (Herbicide)
(2,4,5-Trichlorophenoxy)acetic Acid 3-Butoxypropyl Ester (2,4,5-T-3-Butoxypropyl)	1928-48-9	C15H19Cl3O4	Pesticide (Herbicide)
(4-Chloro-2-methylphenoxy)acetic Acid (MCPA)	94-74-6	C9H9ClO3	Pesticide (Herbicide)
(4-Chloro-2-methylphenoxy)acetic Acid 2-Butoxyethyl Ester (MCPA-butotyl)	19480-43-4	C15H21ClO4	Pesticide (Herbicide)
(4-Chloro-2-methylphenoxy)acetic Acid Isobutyl Ester (MCPA-isobutyl)	1713-11-7	C13H17ClO3	Pesticide (Herbicide)
(4-Chloro-2-methylphenoxy)acetic Acid Isooctyl Ester (MCPA-isooctyl)	26544-20-7	C17H25ClO3	Pesticide (Herbicide)
(4-Chloro-2-methylphenoxy)acetic Acid Isopropyl Ester (MCPA-isopropyl)	2698-40-0	C12H15ClO3	Pesticide (Herbicide)
(4-Chloro-2-methylphenoxy)acetic Acid n-Butyl Ester (MCPA-butyl)	1713-12-8	C13H17ClO3	Pesticide (Herbicide)
2,3,6-Trichlorobenzoic Acid (2,3,6-TBA)	50-31-7	C7H3Cl3O2	Pesticide (Herbicide)
2,4-Dichlorophenoxyacetic Acid Isooctyl(2-ethyl-4-methylpentyl) Ester	53404-37-8	C16H22Cl2O3	Pesticide (Herbicide)
2-Ethylhexyl (4-chloro-2-methylphenoxy)acetate (MCPA-2-ethylhexyl)	29450-45-1	C17H25ClO3	Pesticide (Herbicide)
4-(2,4-Dichlorophenoxy)butanoic Acid (2,4-DB)	94-82-6	C10H10Cl2O3	Pesticide (Herbicide)
4-(4-Chloro-2-methylphenoxy)butanoic Acid (MCPB)	94-81-5	C11H13ClO3	Pesticide (Herbicide)
Acetochlor	34256-82-1	C14H20ClNO2	Pesticide (Herbicide)
Acifluorfen	50594-66-6	C14H7ClF3NO5	Pesticide (Herbicide)
Acifluorfen-methyl	50594-67-7	C15H9ClF3NO5	Pesticide (Herbicide)
Aclonifen	74070-46-5	C12H9ClN2O3	Pesticide (Herbicide)
Alachlor	15972-60-8	C14H20ClNO2	Pesticide (Herbicide)
Ametryn	834-12-8	C9H17N5S	Pesticide (Herbicide)
Amicarbazone	129909-90-6	C10H19N5O2	Pesticide (Herbicide)
Amidosulfuron	120923-37-7	C9H15N5O7S2	Pesticide (Herbicide)
Aminopyralid	150114-71-9	C6H4Cl2N2O2	Pesticide (Herbicide)
Asulam	3337-71-1	C8H10N2O4S	Pesticide (Herbicide)
Atrazine	1912-24-9	C8H14ClN5	Pesticide (Herbicide)
Azafenidin	68049-83-2	C15H13Cl2N3O2	Pesticide (Herbicide)
Azimsulfuron	120162-55-2	C13H16N10O5S	Pesticide (Herbicide)
Beflubutamid	113614-08-7	C18H17F4NO2	Pesticide (Herbicide)
Benazolin	3813-05-6	C9H6ClNO3S	Pesticide (Herbicide)
Benfluralin	1861-40-1	C13H16F3N3O4	Pesticide (Herbicide)
Benfuresate	68505-69-1	C12H16O4S	Pesticide (Herbicide)
Bensulfuron-methyl	83055-99-6	C16H18N4O7S	Pesticide (Herbicide)
Bensulide	741-58-2	C14H24NO4PS3	Pesticide (Herbicide)
Bentazon	25057-89-0	C10H12N2O3S	Pesticide (Herbicide)
Benzobicyclon	156963-66-5	C22H19ClO4S2	Pesticide (Herbicide)
Bicyclopyrone	352010-68-5	C19H20F3NO5	Pesticide (Herbicide)
Bifenox	42576-02-3	C14H9Cl2NO5	Pesticide (Herbicide)
Bispyribac	125401-75-4	C19H18N4O8	Pesticide (Herbicide)
Bromacil	314-40-9	C9H13BrN2O2	Pesticide (Herbicide)
Bromobutide	74712-19-9	C15H22BrNO	Pesticide (Herbicide)
Bromoxynil	1689-84-5	C7H3Br2NO	Pesticide (Herbicide)
Butachlor	23184-66-9	C17H26ClNO2	Pesticide (Herbicide)
Butafenacil	134605-64-4	C20H18ClF3N2O6	Pesticide (Herbicide)
Butralin	33629-47-9	C14H21N3O4	Pesticide (Herbicide)
Butroxydim	138164-12-2	C24H33NO4	Pesticide (Herbicide)
Butylate	2008-41-5	C11H23NOS	Pesticide (Herbicide)
Cafenstrole	125306-83-4	C16H22N4O3S	Pesticide (Herbicide)
Carbetamide	16118-49-3	C12H16N2O3	Pesticide (Herbicide)
Carfentrazone-ethyl	128639-02-1	C15H14Cl2F3N3O3	Pesticide (Herbicide)

Table S20. Compound database for suspect screening (continued)

Compound Name	CAS	Molecular Formula	Category
Chloridazon	1698-60-8	C10H8ClN3O	Pesticide (Herbicide)
Chlorimuron-ethyl	90982-32-4	C15H15ClN4O6S	Pesticide (Herbicide)
Chlorosulfuron	64902-72-3	C12H12ClN5O4S	Pesticide (Herbicide)
Chlorthal-dimethyl (DCPA)	1861-32-1	C10H6Cl4O4	Pesticide (Herbicide)
Chlortoluron	15545-48-9	C10H13ClN2O	Pesticide (Herbicide)
Cinidon-ethyl	142891-20-1	C19H17Cl2NO4	Pesticide (Herbicide)
Cinmethylin	87818-31-3	C18H26O2	Pesticide (Herbicide)
Clethodim	99129-21-2	C17H26ClNO3S	Pesticide (Herbicide)
Clomazone	81777-89-1	C12H14ClNO2	Pesticide (Herbicide)
Clopyralid	1702-17-6	C6H3Cl2NO2	Pesticide (Herbicide)
Cloransulam-methyl	147150-35-4	C15H13ClFN5O5S	Pesticide (Herbicide)
Cumyluron	99485-76-4	C17H19ClN2O	Pesticide (Herbicide)
Cyanazine	21725-46-2	C9H13ClN6	Pesticide (Herbicide)
Cycloate	1134-23-2	C11H21NOS	Pesticide (Herbicide)
Cycloxydim	101205-02-1	C17H27NO3S	Pesticide (Herbicide)
Cyhalofop-butyl	122008-85-9	C20H20FNO4	Pesticide (Herbicide)
Daimuron	42609-52-9	C17H20N2O	Pesticide (Herbicide)
Dalapon	75-99-0	C3H4Cl2O2	Pesticide (Herbicide)
Desmedipham	13684-56-5	C16H16N2O4	Pesticide (Herbicide)
Dicamba	1918-00-9	C8H6Cl2O3	Pesticide (Herbicide)
Dichlobenil	1194-65-6	C7H3Cl2N	Pesticide (Herbicide)
Dichlorprop-butotyl	53404-31-2	C15H20Cl2O4	Pesticide (Herbicide)
Dichlorprop	120-36-5	C9H8Cl2O3	Pesticide (Herbicide)
Diclofop-methyl	51338-27-3	C16H14Cl2O4	Pesticide (Herbicide)
Diclosulam	145701-21-9	C13H10Cl2FN5O3S	Pesticide (Herbicide)
Diflufenican	83164-33-4	C19H11F5N2O2	Pesticide (Herbicide)
Diflufenzopyr	109293-97-2	C15H12F2N4O3	Pesticide (Herbicide)
Dikegulac	18467-77-1	C12H18O7	Pesticide (Herbicide)
Dimefuron	34205-21-5	C15H19ClN4O3	Pesticide (Herbicide)
Dimepiperate	61432-55-1	C15H21NOS	Pesticide (Herbicide)
Dimethachlor	50563-36-5	C13H18ClNO2	Pesticide (Herbicide)
Dimethametryn	22936-75-0	C11H21N5S	Pesticide (Herbicide)
Dimethenamid	87674-68-8	C12H18ClNO2S	Pesticide (Herbicide)
Dinitramine	29091-05-2	C11H13F3N4O4	Pesticide (Herbicide)
Dinoterb	1420-07-1	C10H12N2O5	Pesticide (Herbicide)
Diphenamid	957-51-7	C16H17NO	Pesticide (Herbicide)
Dipropetryn	4147-51-7	C11H21N5S	Pesticide (Herbicide)
Dithiopyr	97886-45-8	C15H16F5NO2S2	Pesticide (Herbicide)
Diuron	330-54-1	C9H10Cl2N2O	Pesticide (Herbicide)
Esprocarb	85785-20-2	C15H23NOS	Pesticide (Herbicide)
Ethalfluralin	55283-68-6	C13H14F3N3O4	Pesticide (Herbicide)
Ethametsulfuron-methyl	97780-06-8	C15H18N6O6S	Pesticide (Herbicide)
Ethiolate	2941-55-1	C7H15NOS	Pesticide (Herbicide)
Ethofumesate	26225-79-6	C13H18O5S	Pesticide (Herbicide)
Ethoxysulfuron	126801-58-9	C15H18N4O7S	Pesticide (Herbicide)
Etobenzanid	79540-50-4	C16H15Cl2NO3	Pesticide (Herbicide)
Fenoxaprop-ethyl	66441-23-4	C18H16ClNO5	Pesticide (Herbicide)
Fentrazamide	158237-07-1	C16H20ClN5O2	Pesticide (Herbicide)
Flamprop-isopropyl	52756-22-6	C19H19ClFNO3	Pesticide (Herbicide)
Flamprop-methyl	52756-25-9	C17H15ClFNO3	Pesticide (Herbicide)
Flazasulfuron	104040-78-0	C13H12F3N5O5S	Pesticide (Herbicide)
Florasulam	145701-23-1	C12H8F3N5O3S	Pesticide (Herbicide)
Florpyrauxifen	943832-81-3	C13H8Cl2F2N2O3	Pesticide (Herbicide)
Florpyrauxifen-benzyl	1390661-72-9	C20H14Cl2F2N2O3	Pesticide (Herbicide)
Fluazifop-butyl	69806-50-4	C19H20F3NO4	Pesticide (Herbicide)
Flucarbazone	145026-88-6	C12H11F3N4O6S	Pesticide (Herbicide)
Flucetosulfuron	412928-75-7	C18H22FN5O8S	Pesticide (Herbicide)
Flufenacet	142459-58-3	C14H13F4N3O2S	Pesticide (Herbicide)
Flufenpyr-ethyl	188489-07-8	C16H13ClF4N2O4	Pesticide (Herbicide)
Flumetsulam	98967-40-9	C12H9F2N5O2S	Pesticide (Herbicide)
Flumiclorac-pentyl	87546-18-7	C21H23ClFNO5	Pesticide (Herbicide)
Flumioxazin	103361-09-7	C19H15FN2O4	Pesticide (Herbicide)
Fluometuron	2164-17-2	C10H11F3N2O	Pesticide (Herbicide)
Fluoroglycofen-ethyl	77501-90-7	C18H13ClF3NO7	Pesticide (Herbicide)
Fluridone	59756-60-4	C19H14F3NO	Pesticide (Herbicide)
Flurochloridone	61213-25-0	C12H10Cl2F3NO	Pesticide (Herbicide)
Fluroxypyr	69377-81-7	C7H5Cl2FN2O3	Pesticide (Herbicide)

Table S20. Compound database for suspect screening (continued)

Compound Name	CAS	Molecular Formula	Category
Fluroxypyr-meptyl	81406-37-3	C15H21Cl2FN2O3	Pesticide (Herbicide)
Flurtamone	96525-23-4	C18H14F3NO2	Pesticide (Herbicide)
Fluthiacet-methyl	117337-19-6	C15H15ClFN3O3S2	Pesticide (Herbicide)
Fomesafen	72178-02-0	C15H10ClF3N2O6S	Pesticide (Herbicide)
Foramsulfuron	173159-57-4	C17H20N6O7S	Pesticide (Herbicide)
Halosulfuron-methyl	100784-20-1	C13H15ClN6O7S	Pesticide (Herbicide)
Haloxyfop-etotyl	87237-48-7	C19H19ClF3NO5	Pesticide (Herbicide)
Haloxyfop	69806-34-4	C15H11ClF3NO4	Pesticide (Herbicide)
Haloxyfop-methyl	69806-40-2	C16H13ClF3NO4	Pesticide (Herbicide)
Hexazinone	51235-04-2	C12H20N4O2	Pesticide (Herbicide)
Imazamethabenz	89318-82-1	C15H18N2O3	Pesticide (Herbicide)
Imazamethabenz-methyl	69969-22-8	C16H20N2O3	Pesticide (Herbicide)
Imazamox	114311-32-9	C15H19N3O4	Pesticide (Herbicide)
Imazapic	104098-48-8	C14H17N3O3	Pesticide (Herbicide)
Imazapyr	81334-34-1	C13H15N3O3	Pesticide (Herbicide)
Imazosulfuron	122548-33-8	C14H13ClN6O5S	Pesticide (Herbicide)
Indaziflam	950782-86-2	C16H20FN5	Pesticide (Herbicide)
Iodosulfuron-methyl	144550-36-7	C14H14IN5O6S	Pesticide (Herbicide)
Ioxynil	1689-83-4	C7H3I2NO	Pesticide (Herbicide)
Isoproturon	34123-59-6	C12H18N2O	Pesticide (Herbicide)
Isoxaben	82558-50-7	C18H24N2O4	Pesticide (Herbicide)
Isoxaflutole	141112-29-0	C15H12F3NO4S	Pesticide (Herbicide)
Lactofen	77501-63-4	C19H15ClF3NO7	Pesticide (Herbicide)
Lenacil	2164-08-1	C13H18N2O2	Pesticide (Herbicide)
Linuron	330-55-2	C9H10Cl2N2O2	Pesticide (Herbicide)
Mecoprop (MCPP)	93-65-2	C10H11ClO3	Pesticide (Herbicide)
Mecoprop-isoctyl	28473-03-2	C18H27ClO3	Pesticide (Herbicide)
Mefenacet	73250-68-7	C16H14N2O2S	Pesticide (Herbicide)
Mesosulfuron-methyl	208465-21-8	C17H21N5O9S2	Pesticide (Herbicide)
Mesotrione	104206-82-8	C14H13NO7S	Pesticide (Herbicide)
Metamifop	256412-89-2	C23H18ClFN2O4	Pesticide (Herbicide)
Metamitron	41394-05-2	C10H10N4O	Pesticide (Herbicide)
Metazachlor	67129-08-2	C14H16ClN3O	Pesticide (Herbicide)
Metazosulfuron	868680-84-6	C15H18ClN7O7S	Pesticide (Herbicide)
Methabenzthiazuron	18691-97-9	C10H11N3OS	Pesticide (Herbicide)
Metobromuron	3060-89-7	C9H11BrN2O2	Pesticide (Herbicide)
Metolachlor	51218-45-2	C15H22ClNO2	Pesticide (Herbicide)
Metosulam	139528-85-1	C14H13Cl2N5O4S	Pesticide (Herbicide)
Metoxuron	19937-59-8	C10H13ClN2O2	Pesticide (Herbicide)
Metribuzin	21087-64-9	C8H14N4OS	Pesticide (Herbicide)
Molinate	2212-67-1	C9H17NOS	Pesticide (Herbicide)
Monolinuron	1746-81-2	C9H11ClN2O2	Pesticide (Herbicide)
Monuron	150-68-5	C9H11ClN2O	Pesticide (Herbicide)
Naproanilide	52570-16-8	C19H17NO2	Pesticide (Herbicide)
Napropamide	15299-99-7	C17H21NO2	Pesticide (Herbicide)
Naptalam	132-66-1	C18H13NO3	Pesticide (Herbicide)
Nicosulfuron	111991-09-4	C15H18N6O6S	Pesticide (Herbicide)
Norflurazon	27314-13-2	C12H9ClF3N3O	Pesticide (Herbicide)
Novaluron	116714-46-6	C17H9ClF8N2O4	Pesticide (Herbicide)
Orbencarb	34622-58-7	C12H16ClNOS	Pesticide (Herbicide)
Orthosulfamuron	213464-77-8	C16H20N6O6S	Pesticide (Herbicide)
Oryzalin	19044-88-3	C12H18N4O6S	Pesticide (Herbicide)
Oxadiargyl	39807-15-3	C15H14Cl2N2O3	Pesticide (Herbicide)
Oxadiazon	19666-30-9	C15H18Cl2N2O3	Pesticide (Herbicide)
Oxasulfuron	144651-06-9	C17H18N4O6S	Pesticide (Herbicide)
Oxaziclomefone	153197-14-9	C20H19Cl2NO2	Pesticide (Herbicide)
Oxyfluorfen	42874-03-3	C15H11ClF3NO4	Pesticide (Herbicide)
Pebulate	1114-71-2	C10H21NOS	Pesticide (Herbicide)
Pendimethalin	40487-42-1	C13H19N3O4	Pesticide (Herbicide)
Penoxsulam	219714-96-2	C16H14F5N5O5S	Pesticide (Herbicide)
Pentanochlor	2307-68-8	C13H18ClNO	Pesticide (Herbicide)
Pentoxazone	110956-75-7	C17H17ClFNO4	Pesticide (Herbicide)
Pethoxamid	106700-29-2	C16H22ClNO2	Pesticide (Herbicide)
Phenmedipham	13684-63-4	C16H16N2O4	Pesticide (Herbicide)
Picloram	1918-02-1	C6H3Cl3N2O2	Pesticide (Herbicide)
Picolinafen	137641-05-5	C19H12F4N2O2	Pesticide (Herbicide)
Pinoxaden	243973-20-8	C23H32N2O4	Pesticide (Herbicide)

Table S20. Compound database for suspect screening (continued)

Compound Name	CAS	Molecular Formula	Category
Piperophos	24151-93-7	C14H28NO3PS2	Pesticide (Herbicide)
Pretilachlor	51218-49-6	C17H26ClNO2	Pesticide (Herbicide)
Primisulfuron-methyl	86209-51-0	C15H12F4N4O7S	Pesticide (Herbicide)
Procyanazine	32889-48-8	C10H13ClN6	Pesticide (Herbicide)
Prodiamine	29091-21-2	C13H17F3N4O4	Pesticide (Herbicide)
Profoxydim	139001-49-3	C24H32ClNO4S	Pesticide (Herbicide)
Prometon	1610-18-0	C10H19N5O	Pesticide (Herbicide)
Prometryn	7287-19-6	C10H19N5S	Pesticide (Herbicide)
Propachlor	1918-16-7	C11H14ClNO	Pesticide (Herbicide)
Propanil	709-98-8	C9H9Cl2NO	Pesticide (Herbicide)
Propaquizafop	111479-05-1	C22H22ClN3O5	Pesticide (Herbicide)
Propazine	139-40-2	C9H16ClN5	Pesticide (Herbicide)
Propoxycarbazone	181274-15-7	C15H18N4O7S	Pesticide (Herbicide)
Propyrisulfuron	570415-88-2	C16H18ClN7O5S	Pesticide (Herbicide)
Propyzamide	23950-58-5	C12H11Cl2NO	Pesticide (Herbicide)
Prosulfocarb	52888-80-9	C14H21NOS	Pesticide (Herbicide)
Prosulfuron	94125-34-5	C15H16F3N5O4S	Pesticide (Herbicide)
Pyraclonil	158353-15-2	C15H15ClN6	Pesticide (Herbicide)
Pyraflufen-ethyl	129630-19-9	C15H13Cl2F3N2O4	Pesticide (Herbicide)
Pyrasulfotole	365400-11-9	C14H13F3N2O4S	Pesticide (Herbicide)
Pyrazosulfuron-ethyl	93697-74-6	C14H18N6O7S	Pesticide (Herbicide)
Pyribenzoxim	168088-61-7	C32H27N5O8	Pesticide (Herbicide)
Pyributicarb	88678-67-5	C18H22N2O2S	Pesticide (Herbicide)
Pyridate	55512-33-9	C19H23ClN2O2S	Pesticide (Herbicide)
Pyriftalid	135186-78-6	C15H14N2O4S	Pesticide (Herbicide)
Pyriminobac-methyl	136191-64-5	C17H19N3O6	Pesticide (Herbicide)
Pyrimisulfan	221205-90-9	C16H19F2N3O6S	Pesticide (Herbicide)
Pyrithiobac	123343-16-8	C13H11ClN2O4S	Pesticide (Herbicide)
Pyroxsulam	422556-08-9	C14H13F3N6O5S	Pesticide (Herbicide)
Quinchlorac	84087-01-4	C10H5Cl2NO2	Pesticide (Herbicide)
Quinmerac	90717-03-6	C11H8ClNO2	Pesticide (Herbicide)
Quinoclamine	2797-51-5	C10H6ClNO2	Pesticide (Herbicide)
Quizalofop-ethyl	76578-14-8	C19H17ClN2O4	Pesticide (Herbicide)
Rimsulfuron	122931-48-0	C14H17N5O7S2	Pesticide (Herbicide)
Saflufenacil	372137-35-4	C17H17ClF4N4O5S	Pesticide (Herbicide)
Sethoxydim	74051-80-2	C17H29NO3S	Pesticide (Herbicide)
S-Ethyl Dipropylthiocarbamate (EPTC)	759-94-4	C9H19NOS	Pesticide (Herbicide)
Siduron	1982-49-6	C14H20N2O	Pesticide (Herbicide)
Simazine	122-34-9	C7H12ClN5	Pesticide (Herbicide)
Simeton	673-04-1	C8H15N5O	Pesticide (Herbicide)
Sulcotrione	99105-77-8	C14H13ClO5S	Pesticide (Herbicide)
Sulfentrazone	122836-35-5	C11H10Cl2F2N4O3S	Pesticide (Herbicide)
Sulfometuron-methyl	74222-97-2	C15H16N4O5S	Pesticide (Herbicide)
Sulfosulfuron	141776-32-1	C16H18N6O7S2	Pesticide (Herbicide)
Tebutam	35256-85-0	C15H23NO	Pesticide (Herbicide)
Tebuthiuron	34014-18-1	C9H16N4OS	Pesticide (Herbicide)
Tembotrione	335104-84-2	C17H16ClF3O6S	Pesticide (Herbicide)
Tepraloxydim	149979-41-9	C17H24ClNO4	Pesticide (Herbicide)
Terbacil	5902-51-2	C9H13ClN2O2	Pesticide (Herbicide)
Terbucarb	1918-11-2	C17H27NO2	Pesticide (Herbicide)
Terbumeton	33693-04-8	C10H19N5O	Pesticide (Herbicide)
Terbutryn	886-50-0	C10H19N5S	Pesticide (Herbicide)
Thenylchlor	96491-05-3	C16H18ClNO2S	Pesticide (Herbicide)
Thiazopyr	117718-60-2	C16H17F5N2O2S	Pesticide (Herbicide)
Thifensulfuron-methyl	79277-27-3	C12H13N5O6S2	Pesticide (Herbicide)
Thiobencarb	28249-77-6	C12H16ClNOS	Pesticide (Herbicide)
Tiocarbazil	36756-79-3	C16H25NOS	Pesticide (Herbicide)
Topramezone	210631-68-8	C16H17N3O5S	Pesticide (Herbicide)
Tralkoxydim	87820-88-0	C20H27NO3	Pesticide (Herbicide)
Triallate	2303-17-5	C10H16Cl3NOS	Pesticide (Herbicide)
Triasulfuron	82097-50-5	C14H16ClN5O5S	Pesticide (Herbicide)
Triaziflam	131475-57-5	C17H24FN5O	Pesticide (Herbicide)
Tribenuron-methyl	101200-48-0	C15H17N5O6S	Pesticide (Herbicide)
Triclopyr	55335-06-3	C7H4Cl3NO3	Pesticide (Herbicide)
Triclopyr-butotyl	64700-56-7	C13H16Cl3NO4	Pesticide (Herbicide)
Trietazine	1912-26-1	C9H16ClN5	Pesticide (Herbicide)
Trifloxysulfuron	145099-21-4	C14H14F3N5O6S	Pesticide (Herbicide)

Table S20. Compound database for suspect screening (continued)

Compound Name	CAS	Molecular Formula	Category
Trifluralin	1582-09-8	C13H16F3N3O4	Pesticide (Herbicide)
Triflusulfuron-methyl	126535-15-7	C17H19F3N6O6S	Pesticide (Herbicide)
Tritosulfuron	142469-14-5	C13H9F6N5O4S	Pesticide (Herbicide)
Metsulfuron-methyl	74223-64-6	C14H15N5O6S	Pesticide (Herbicide) / Pesticide TP (Iodosulfuron)
Clofibric Acid	882-09-7	C10H11ClO3	Pesticide (Herbicide) / Pharmaceutical TP (Clofibrate)
Endothal	145-73-3	C8H10O5	Pesticide (Herbicide, Defoliant)
Dichlorophen	97-23-4	C13H10Cl2O2	Pesticide (Herbicide, Fungicide, Microbiocide)
2,4-Dichlorophenoxyacetic Acid (2,4-D)	94-75-7	C8H6Cl2O3	Pesticide (Herbicide, Plant Growth Regulator)
Chlorpropham	101-21-3	C10H12ClNO2	Pesticide (Herbicide, Plant Growth Regulator)
Dichlorprop-isoctyl	28631-35-8	C17H24Cl2O3	Pesticide (Herbicide, Plant Growth Regulator)
Imazaquin	81335-37-7	C17H17N3O3	Pesticide (Herbicide, Plant Growth Regulator)
Imazethapyr	81335-77-5	C15H19N3O3	Pesticide (Herbicide, Plant Growth Regulator)
Propham	122-42-9	C10H13NO2	Pesticide (Herbicide, Plant Growth Regulator)
Trinexapac-ethyl	95266-40-3	C13H16O5	Pesticide (Herbicide, Plant Growth Regulator)
Buprofezin	69327-76-0	C16H23N3OS	Pesticide (Insect Growth Regulator)
Diflubenzuron	35367-38-5	C14H9ClF2N2O2	Pesticide (Insect Growth Regulator)
Etoxazole	153233-91-1	C21H23F2NO2	Pesticide (Insect Growth Regulator)
Hexythiazox	78587-05-0	C17H21ClN2O2S	Pesticide (Insect Growth Regulator)
Methoxyfenozide	161050-58-4	C22H28N2O3	Pesticide (Insect Growth Regulator)
Noviflumuron	121451-02-3	C17H7Cl2F9N2O3	Pesticide (Insect Growth Regulator)
Pyrazoxyfen	71561-11-0	C20H16Cl2N2O3	Pesticide (Insect Growth Regulator)
Pyriproxyfen	95737-68-1	C20H19NO3	Pesticide (Insect Growth Regulator)
Tebufenozide	112410-23-8	C22H28N2O2	Pesticide (Insect Growth Regulator)
Chlorfluazuron	71422-67-8	C20H9Cl3F5N3O3	Pesticide (Insect Growth Regulator, Insecticide)
Clofentezine	74115-24-5	C14H8Cl2N4	Pesticide (Insect Growth Regulator, Insecticide)
Ethyl Butylacetylaminopropionate	52304-36-6	C11H21NO3	Pesticide (Insect Repellent)
N-Methylneodecanamide (NMDA)	105726-67-8	C11H23NO	Pesticide (Insect Repellent)
3,5-Xylyl Methylcarbamate (XMC)	2655-14-3	C10H13NO2	Pesticide (Insecticide)
Acephate	30560-19-1	C4H10NO3PS	Pesticide (Insecticide)
Acetamiprid	135410-20-7	C10H11ClN4	Pesticide (Insecticide)
Afoxolaner	1093861-60-9	C26H17ClF9N3O3	Pesticide (Insecticide)
Amitraz	33089-61-1	C19H23N3	Pesticide (Insecticide)
Anilofos	64249-01-0	C13H19ClNO3PS2	Pesticide (Insecticide)
Avermectin B1A (Abamectin)	65195-55-3	C48H72O14	Pesticide (Insecticide)
Avermectin B1b (Abamectin)	65195-56-4	C47H70O14	Pesticide (Insecticide)
Azamethiphos	35575-96-3	C9H10ClN2O5PS	Pesticide (Insecticide)
Azinphos-methyl	86-50-0	C10H12N3O3PS2	Pesticide (Insecticide)
Bendiocarb	22781-23-3	C11H13NO4	Pesticide (Insecticide)
Benfuracarb	82560-54-1	C20H30N2O5S	Pesticide (Insecticide)
Bifenazate	149877-41-8	C17H20N2O3	Pesticide (Insecticide)
Bromchlophos (Naled)	300-76-5	C4H7Br2Cl2O4P	Pesticide (Insecticide)
Bromfenvinphos	33399-00-7	C12H14BrCl2O4P	Pesticide (Insecticide)
Bromopropylate	18181-80-1	C17H16Br2O3	Pesticide (Insecticide)
Butocarboxim	34681-10-2	C7H14N2O2S	Pesticide (Insecticide)
Butoxycarboxim	34681-23-7	C7H14N2O4S	Pesticide (Insecticide)
Cadusafos	95465-99-9	C10H23O2PS2	Pesticide (Insecticide)
Carbosulfan	55285-14-8	C20H32N2O3S	Pesticide (Insecticide)
Chlorantraniliprole	500008-45-7	C18H14BrCl2N5O2	Pesticide (Insecticide)
Chlorethoxyfos	54593-83-8	C6H11Cl4O3PS	Pesticide (Insecticide)
Chlorfenapyr	122453-73-0	C15H11BrClF3N2O	Pesticide (Insecticide)
Chlorfenvinphos	470-90-6	C12H14Cl3O4P	Pesticide (Insecticide)
Chlorpyrifos-methyl	5598-13-0	C7H7Cl3NO3PS	Pesticide (Insecticide)
Coumaphos	56-72-4	C14H16ClO5PS	Pesticide (Insecticide)
Crufomate	299-86-5	C12H19ClNO3P	Pesticide (Insecticide)
Cyanofenphos	13067-93-1	C15H14NO2PS	Pesticide (Insecticide)
Cyanophos	2636-26-2	C9H10NO3PS	Pesticide (Insecticide)
Cyclaniliprole	1031756-98-5	C21H17Br2Cl2N5O2	Pesticide (Insecticide)
Diafenthiuron	80060-09-9	C23H32N2OS	Pesticide (Insecticide)
Diazinon	333-41-5	C12H21N2O3PS	Pesticide (Insecticide)
Dichlorvos (DDVP)	62-73-7	C4H7Cl2O4P	Pesticide (Insecticide) / Pesticide TP (Bromchlophos)
Dicofol	115-32-2	C14H9Cl5O	Pesticide (Insecticide)
Dicrotophos	141-66-2	C8H16NO5P	Pesticide (Insecticide)
Diethofencarb	87130-20-9	C14H21NO4	Pesticide (Insecticide)
Dimethoate	60-51-5	C5H12NO3PS2	Pesticide (Insecticide)
Dinotefuran	165252-70-0	C7H14N4O3	Pesticide (Insecticide)
Emamectin	119791-41-2	C49H75NO13	Pesticide (Insecticide)
Ethiofencarb	29973-13-5	C11H15NO2S	Pesticide (Insecticide)

Table S20. Compound database for suspect screening (continued)

Compound Name	CAS	Molecular Formula	Category
Ethion	563-12-2	C9H22O4P2S4	Pesticide (Insecticide)
Ethiprole	181587-01-9	C13H9Cl2F3N4OS	Pesticide (Insecticide)
Famphur	52-85-7	C10H16NO5PS2	Pesticide (Insecticide)
Fenazaquin	120928-09-8	C20H22N2O	Pesticide (Insecticide)
Fenitrothion	122-14-5	C9H12NO5PS	Pesticide (Insecticide)
Fenobucarb	3766-81-2	C12H17NO2	Pesticide (Insecticide)
Fenpyroximate	134098-61-6	C24H27N3O4	Pesticide (Insecticide)
Fenson	80-38-6	C12H9ClO3S	Pesticide (Insecticide)
Fipronil	120068-37-3	C12H4Cl2F6N4OS	Pesticide (Insecticide)
Flonicamid	158062-67-0	C9H6F3N3O	Pesticide (Insecticide)
Fluacrypyrim	229977-93-9	C20H21F3N2O5	Pesticide (Insecticide)
Fluazuron	86811-58-7	C20H10Cl2F5N3O3	Pesticide (Insecticide)
Flubendiamide	272451-65-7	C23H22F7IN2O4S	Pesticide (Insecticide)
Flufenoxuron	101463-69-8	C21H11ClF6N2O3	Pesticide (Insecticide)
Flupyradifurone	951659-40-8	C12H11ClF2N2O2	Pesticide (Insecticide)
Formetanate	22259-30-9	C11H15N3O2	Pesticide (Insecticide)
Furathiocarb	65907-30-4	C18H26N2O5S	Pesticide (Insecticide)
Hexaflumuron	86479-06-3	C16H8Cl2F6N2O3	Pesticide (Insecticide)
Hydramethylnon	67485-29-4	C25H24F6N4	Pesticide (Insecticide)
Imidacloprid	138261-41-3	C9H10ClN5O2	Pesticide (Insecticide)
Indoxacarb	173584-44-6	C22H17ClF3N3O7	Pesticide (Insecticide)
Isazophos-methyl	42509-83-1	C7H13ClN3O3PS	Pesticide (Insecticide)
Isocarbophos	24353-61-5	C11H16NO4PS	Pesticide (Insecticide)
Isoprocarb	2631-40-5	C11H15NO2	Pesticide (Insecticide)
Isoxathion	18854-01-8	C13H16NO4PS	Pesticide (Insecticide)
Kadethrin	58769-20-3	C23H24O4S	Pesticide (Insecticide)
Lufenuron	103055-07-8	C17H8Cl2F8N2O3	Pesticide (Insecticide)
Malathion	121-75-5	C10H19O6PS2	Pesticide (Insecticide)
Mecarbam	2595-54-2	C10H20NO5PS2	Pesticide (Insecticide)
Methamidophos	10265-92-6	C2H8NO2PS	Pesticide (Insecticide) / Pesticide TP (Acephate)
Methidathion	950-37-8	C6H11N2O4PS3	Pesticide (Insecticide)
Methomyl	16752-77-5	C5H10N2O2S	Pesticide (Insecticide)
Methoxychlor	72-43-5	C16H15Cl3O2	Pesticide (Insecticide)
Milbemycin A3	51596-10-2	C31H44O7	Pesticide (Insecticide)
Milbemycin A4	51596-11-3	C32H46O7	Pesticide (Insecticide)
Nitenpyram	150824-47-8	C11H15ClN4O2	Pesticide (Insecticide)
O-Ethyl O-(4-nitrophenyl) Phenylphosphonothioate (EPN)	2104-64-5	C14H14NO4PS	Pesticide (Insecticide)
Oxydemeton-methyl	301-12-2	C6H15O4PS2	Pesticide (Insecticide)
Parathion	56-38-2	C10H14NO5PS	Pesticide (Insecticide)
Phenthoate	2597-03-7	C12H17O4PS2	Pesticide (Insecticide)
Phosalone	2310-17-0	C12H15ClNO4PS2	Pesticide (Insecticide)
Phosmet	732-11-6	C11H12NO4PS2	Pesticide (Insecticide)
Phosphamidon	13171-21-6	C10H19ClNO5P	Pesticide (Insecticide)
Phoxim	14816-18-3	C12H15N2O3PS	Pesticide (Insecticide)
Pirimicarb	23103-98-2	C11H18N4O2	Pesticide (Insecticide)
Pirimiphos-methyl	29232-93-7	C11H20N3O3PS	Pesticide (Insecticide)
Profenofos	41198-08-7	C11H15BrClO3PS	Pesticide (Insecticide)
Propargite	2312-35-8	C19H26O4S	Pesticide (Insecticide)
Propetamphos	31218-83-4	C10H20NO4PS	Pesticide (Insecticide)
Propoxur	114-26-1	C11H15NO3	Pesticide (Insecticide)
Prothiofos	34643-46-4	C11H15Cl2O2PS2	Pesticide (Insecticide)
Pymetrozine	123312-89-0	C10H11N5O	Pesticide (Insecticide)
Pyrifluquinazon	337458-27-2	C19H15F7N4O2	Pesticide (Insecticide)
Pyridalyl	179101-81-6	C18H14Cl4F3NO3	Pesticide (Insecticide)
Pyridaphenthion	119-12-0	C14H17N2O4PS	Pesticide (Insecticide)
Pyrimidifen	105779-78-0	C20H28ClN3O2	Pesticide (Insecticide)
Quinalphos	13593-03-8	C12H15N2O3PS	Pesticide (Insecticide)
Rotenone	83-79-4	C23H22O6	Pesticide (Insecticide)
Spirodiclofen	148477-71-8	C21H24Cl2O4	Pesticide (Insecticide)
Spiromesifen	283594-90-1	C23H30O4	Pesticide (Insecticide)
Spirotetramat	203313-25-1	C21H27NO5	Pesticide (Insecticide)
Sulfotepp	3689-24-5	C8H20O5P2S2	Pesticide (Insecticide)
Sulfoxaflor	946578-00-3	C10H10F3N3OS	Pesticide (Insecticide)
Tebufenpyrad	119168-77-3	C18H24ClN3O	Pesticide (Insecticide)
Tebupirimfos	96182-53-5	C13H23N2O3PS	Pesticide (Insecticide)
Teflubenzuron	83121-18-0	C14H6Cl2F4N2O2	Pesticide (Insecticide)
Temephos	3383-96-8	C16H20O6P2S3	Pesticide (Insecticide)

Table S20. Compound database for suspect screening (continued)

Compound Name	CAS	Molecular Formula	Category
Tetrachlorvinphos	22248-79-9	C10H9Cl4O4P	Pesticide (Insecticide)
Tetradifon	116-29-0	C12H6Cl4O2S	Pesticide (Insecticide)
Thiacloprid	111988-49-9	C10H9ClN4S	Pesticide (Insecticide)
Thiamethoxam	153719-23-4	C8H10ClN5O3S	Pesticide (Insecticide)
Thiofanox	39196-18-4	C9H18N2O2S	Pesticide (Insecticide)
Thiometon	640-15-3	C6H15O2PS3	Pesticide (Insecticide)
Tolfenpyrad	129558-76-5	C21H22ClN3O2	Pesticide (Insecticide)
Trichlorfon	52-68-6	C4H8Cl3O4P	Pesticide (Insecticide)
Triflumuron	64628-44-0	C15H10ClF3N2O3	Pesticide (Insecticide)
Vamidothion	2275-23-2	C8H18NO4PS2	Pesticide (Insecticide)
Omethoate	1113-02-6	C5H12NO4PS	Pesticide (Insecticide) / Pesticide TP (Dimethoate)
Clothianidin	210880-92-5	C6H8ClN5O2S	Pesticide (Insecticide) / Pesticide TP (Thiamethoxam)
Fenthion	55-38-9	C10H15O3PS2	Pesticide (Insecticide, Avicide)
Azaconazole	60207-31-0	C12H11Cl2N3O2	Pesticide (Insecticide, Fungicide)
Benzyl Benzoate	120-51-4	C14H12O2	Pesticide (Insecticide, Fungicide)
Dinobuton	973-21-7	C14H18N2O7	Pesticide (Insecticide, Fungicide)
Chinomethionate	2439-01-2	C10H6N2OS2	Pesticide (Insecticide, Fungicide, Fumigant)
Camphor	76-22-2	C10H16O	Pesticide (Insecticide, Fungicide, Microbiocide)
Cyromazine	66215-27-8	C6H10N6	Pesticide (Insecticide, Insect Growth Regulator)
Fenoxycarb	72490-01-8	C17H19NO4	Pesticide (Insecticide, Insect Growth Regulator)
Methiocarb	2032-65-7	C11H15NO2S	Pesticide (Insecticide, Molluscicide)
Aldicarb	116-06-3	C7H14N2O2S	Pesticide (Insecticide, Nematicide)
Chlorpyrifos	2921-88-2	C9H11Cl3NO3PS	Pesticide (Insecticide, Nematicide)
Disulfoton	298-04-4	C8H19O2PS3	Pesticide (Insecticide, Nematicide)
Ethoprophos	13194-48-4	C8H19O2PS2	Pesticide (Insecticide, Nematicide)
Fenamiphos	22224-92-6	C13H22NO3PS	Pesticide (Insecticide, Nematicide)
Oxamyl	23135-22-0	C7H13N3O3S	Pesticide (Insecticide, Nematicide)
Parathion-methyl	298-00-0	C8H10NO5PS	Pesticide (Insecticide, Nematicide)
Phorate	298-02-2	C7H17O2PS3	Pesticide (Insecticide, Nematicide)
Terbufos	13071-79-9	C9H21O2PS3	Pesticide (Insecticide, Nematicide)
Triazophos	24017-47-8	C12H16N3O3PS	Pesticide (Insecticide, Nematicide)
Carbofuran	1563-66-2	C12H15NO3	Pesticide (Insecticide, Nematicide) / Pesticide TP (Benfuracarb)
Carbaryl	63-25-2	C12H11NO2	Pesticide (Insecticide, Plant Growth Regulator, Nematicide)
Nitrapyrin	1929-82-4	C6H3Cl4N	Pesticide (Microbiocide)
2-(Thiocyanomethylthio)benzothiazole (TCMTB)	21564-17-0	C9H6N2S3	Pesticide (Microbiocide, Fungicide)
8-Hydroxyquinoline	148-24-3	C9H7NO	Pesticide (Microbiocide, Fungicide)
Diiodomethyl P-chlorophenyl Sulfone	20018-12-6	C7H5ClI2O2S	Pesticide (Microbiocide, Fungicide)
Diiodomethyl P-tolyl Sulfone	20018-09-1	C8H8I2O2S	Pesticide (Microbiocide, Fungicide)
Octhilinone	26530-20-1	C11H19NOS	Pesticide (Microbiocide, Fungicide)
Niclosamide	50-65-7	C13H8Cl2N2O4	Pesticide (Molluscicide)
Thiodicarb	59669-26-0	C10H18N4O4S3	Pesticide (Molluscicide, Insecticide)
Fosthiazate	98886-44-3	C9H18NO3PS2	Pesticide (Nematicide)
Imicyafos	140163-89-9	C11H21N4O2PS	Pesticide (Nematicide)
3-Trifluoromethyl-4-nitrophenol (TFM)	88-30-2	C7H4F3NO3	Pesticide (Piscicide)
1-Naphthaleneacetamide (NAD)	86-86-2	C12H11NO	Pesticide (Plant Growth Regulator)
1-Naphthaleneacetic Acid	86-87-3	C12H10O2	Pesticide (Plant Growth Regulator)
2-Methyl-1-naphthaleneacetic Acid	85-08-5	C13H12O2	Pesticide (Plant Growth Regulator)
2-Naphthoxyacetic Acid (NOA)	120-23-0	C12H10O3	Pesticide (Plant Growth Regulator)
3'-Methylphthalanilic Acid	85-72-3	C15H13NO3	Pesticide (Plant Growth Regulator)
4-Chlorophenoxyacetic Acid (4-CPA)	122-88-3	C8H7ClO3	Pesticide (Plant Growth Regulator)
Abscisic Acid	21293-29-8	C15H20O4	Pesticide (Plant Growth Regulator)
Ancymidol	12771-68-5	C15H16N2O2	Pesticide (Plant Growth Regulator)
Clodinafop-propargyl	105512-06-9	C17H13ClFNO4	Pesticide (Plant Growth Regulator)
Cyclanilide	113136-77-9	C11H9Cl2NO3	Pesticide (Plant Growth Regulator)
Daminozide	1596-84-5	C6H12N2O3	Pesticide (Plant Growth Regulator)
Ethephon	16672-87-0	C2H6ClO3P	Pesticide (Plant Growth Regulator)
Ethyl 1-Naphthaleneacetate	2122-70-5	C14H14O2	Pesticide (Plant Growth Regulator)
Flumetralin	62924-70-3	C16H12ClF4N3O4	Pesticide (Plant Growth Regulator)
Flurprimidol	56425-91-3	C15H15F3N2O2	Pesticide (Plant Growth Regulator)
Forchlorfenuron	68157-60-8	C12H10ClN3O	Pesticide (Plant Growth Regulator)
Gibberellic Acid	77-06-5	C19H22O6	Pesticide (Plant Growth Regulator)
Indole-3-butyric Acid	133-32-4	C12H13NO2	Pesticide (Plant Growth Regulator)
Kinetin	525-79-1	C10H9N5O	Pesticide (Plant Growth Regulator)
Methyl 1-Naphthaleneacetate	2876-78-0	C13H12O2	Pesticide (Plant Growth Regulator)
N6-Benzyladenine	1214-39-7	C12H11N5	Pesticide (Plant Growth Regulator)
Paclobutrazol	76738-62-0	C15H20ClN3O	Pesticide (Plant Growth Regulator)
Dimethipin	55290-64-7	C6H10O4S2	Pesticide (Plant Growth Regulator, Defoliant)

Table S20. Compound database for suspect screening (continued)

Compound Name	CAS	Molecular Formula	Category
Dichlorflurenol-methyl	21634-96-8	C15H10Cl2O3	Pesticide (Plant Growth Regulator, Herbicide, Pruning Aid)
Flurecol-methyl	1216-44-0	C15H12O3	Pesticide (Plant Growth Regulator, Herbicide, Pruning Aid)
Bromethalin	63333-35-7	C14H7Br3F3N3O4	Pesticide (Rodenticide)
Chlorophacinone	3691-35-8	C23H15ClO3	Pesticide (Rodenticide)
Coumatetralyl	5836-29-3	C19H16O3	Pesticide (Rodenticide)
Diphacinone	82-66-6	C23H16O3	Pesticide (Rodenticide)
Pindone	83-26-1	C14H14O3	Pesticide (Rodenticide)
Strychnine	57-24-9	C21H22N2O2	Pesticide (Rodenticide, Avicide)
N-(2-Ethylhexyl)-5-norbornene-2,3-dicarboximide (MGK 264)	113-48-4	C17H25NO2	Pesticide (Synergist)
Piperonyl Butoxide	51-03-6	C19H30O5	Pesticide (Synergist, Insecticide)
Pentachlorophenol (PCP)	87-86-5	C6HCl5O	Pesticide (Wood Preservative, Microbiocide, Algaecide, Fungicide, Molluscicide)
Acetamiprid-N-desmethyl	190604-92-3	C9H9ClN4	Pesticide TP (Acetamiprid)
N-[(6-Chloropyridin-3-yl)methyl]-N-methylamine	120739-62-0	C7H9ClN2	Pesticide TP (Acetamiprid)
Acetochlor Ethanesulfonic Acid (Acetochlor ESA)	187022-11-3	C14H21NO5S	Pesticide TP (Acetochlor)
Acetochlor Oxanilic Acid (Acetochlor OA)	194992-44-4	C14H19NO4	Pesticide TP (Acetochlor)
Acetochlor-2-hydroxy	60090-47-3	C14H21NO3	Pesticide TP (Acetochlor)
Alachlor Ethanesulfonic Acid (Alachlor ESA)	142363-53-9	C14H21NO5S	Pesticide TP (Alachlor)
Alachlor Oxanilic Acid (Alachlor OA)	171262-17-2	C14H19NO4	Pesticide TP (Alachlor)
Alachlor-2-hydroxy	56681-55-1	C14H21NO3	Pesticide TP (Alachlor)
Aldicarb Sulfone (Aldoxycarb)	1646-88-4	C7H14N2O4S	Pesticide TP (Aldicarb)
Aldicarb Sulfoxide	1646-87-3	C7H14N2O3S	Pesticide TP (Aldicarb)
N-(2,4-Dimethylphenyl)formamide	60397-77-5	C9H11NO	Pesticide TP (Amitraz)
N'-(2,4-Dimethylphenyl)-N-Methylformamidine	33089-74-6	C10H14N2	Pesticide TP (Amitraz)
Atrazine-desisopropyl-2-hydroxy	7313-54-4	C5H9N5O	Pesticide TP (Atrazine)
Atrazine-2-hydroxy	2163-68-0	C8H15N5O	Pesticide TP (Atrazine, Ametryn)
2-Chloro-4,6-diamino-S-triazine (Atrazine-desethyl-desisopropyl)	3397-62-4	C3H4ClN5	Pesticide TP (Atrazine, Cyanazine)
Ammeline	645-92-1	C3H5N5O	Pesticide TP (Atrazine, Cyromazine)
Atrazine-desethyl-2-hydroxy	19988-24-0	C6H11N5O	Pesticide TP (Atrazine, Prometon)
Atrazine-desethyl	6190-65-4	C6H10ClN5	Pesticide TP (Atrazine, Propazine)
Atrazine-desisopropyl	1007-28-9	C5H8ClN5	Pesticide TP (Atrazine, Simazine, Cyanazine, Terbuthylazine)
Azinphos-methyl-oxon	961-22-8	C10H12N3O4PS	Pesticide TP (Azinphos-methyl)
Azoxystrobin Acid	1185255-09-7	C21H15N3O5	Pesticide TP (Azoxystrobin)
2-Amino-4,6-dimethoxypyrimidine	36315-01-2	C6H9N3O2	Pesticide TP (Bensulfuron-methyl, Foramsulfuron)
Methyl 2-(Sulfamoylmethyl)benzoate	112941-26-1	C9H11NO4S	Pesticide TP (Bensulfuron-methyl)
Bentazon-methyl	61592-45-8	C11H14N2O3S	Pesticide TP (Bentazon)
Bifenox Acid	53774-07-5	C13H7Cl2NO5	Pesticide TP (Bifenox)
Bis(4-bromophenyl)glycolic Acid	30738-49-9	C14H10Br2O3	Pesticide TP (Bromopropylate)
3,5-Dibromo-4-hydroxybenzoic Acid	3337-62-0	C7H4Br2O3	Pesticide TP (Bromoxynil)
Ethirimol	23947-60-6	C11H19N3O	Pesticide TP (Bupirimate)
Butocarboxim Sulfoxide	34681-24-8	C7H14N2O3S	Pesticide TP (Butocarboxim)
1,2,3,6-Tetrahydrophthalimide	85-40-5	C8H9NO2	Pesticide TP (Captan)
Tetrahydrofurfurylamine	4795-29-3	C5H11NO	Pesticide TP (Captan)
2-Aminobenzimidazole	934-32-7	C7H7N3	Pesticide TP (Carbendazim)
Carbofuran-7-phenol	1563-38-8	C10H12O2	Pesticide TP (Carbofuran)
Carbofuran-3-hydroxy	16655-82-6	C12H15NO4	Pesticide TP (Carbofuran, Benfuracarb)
Carbofuran-3-keto	16709-30-1	C12H13NO4	Pesticide TP (Carbofuran, Benfuracarb)
Carfentrazone	128621-72-7	C13H10Cl2F3N3O3	Pesticide TP (Carfentrazone-ethyl)
Chloridazon-desphenyl	6339-19-1	C4H4ClN3O	Pesticide TP (Chloridazon)
Chloridazon-methyl-desphenyl	17254-80-7	C5H6ClN3O	Pesticide TP (Chloridazon)
4-Hydroxy-2,5,6-trichloroisophthalonitrile	28343-61-5	C8HCl3N2O	Pesticide TP (Chlorothalonil)
Chlorpyrifos-oxon	5598-15-2	C9H11Cl3NO4P	Pesticide TP (Chlorpyrifos)
Chlorpyrifos-methyl-oxon	5598-52-7	C7H7Cl3NO4P	Pesticide TP (Chlorpyrifos)
3,5,6-Trichloro-2-pyridinol	6515-38-4	C5H2Cl3NO	Pesticide TP (Chlorpyrifos, Chlorpyrifos-methyl)
2-Chlorobenzenesulfonamide	6961-82-6	C6H6ClNO2S	Pesticide TP (Chlorsulfuron)
Chlortoluron-desmethyl	22175-22-0	C9H11ClN2O	Pesticide TP (Chlortoluron)
Dimethyl 3-Hydroxy-3-[2-(ethylsulfonyl)propyl]glutarate	116007-17-1	C12H22O7S	Pesticide TP (Clethodim)
4-Chlorophenol	106-48-9	C6H5ClO	Pesticide TP (Climbazole, Difenoconazole, Profoxydim)
Clodinafop	114420-56-3	C14H11ClFNO4	Pesticide TP (Clodinafop-propargyl)
Coumaphos-oxon	321-54-0	C14H16ClO6P	Pesticide TP (Coumaphos)
Cyanazine Acid	36576-43-9	C9H14ClN5O2	Pesticide TP (Cyanazine)
Cyanazine Amide	36576-42-8	C9H15ClN6O	Pesticide TP (Cyanazine)
N-Deethylcyanazine Amide	36556-77-1	C7H11ClN6O	Pesticide TP (Cyanazine)
1,2,4-Triazole-1-acetic Acid	28711-29-7	C4H5N3O2	Pesticide TP (Cyproconazole, Myclobutanil)
Ammelide	645-93-2	C3H4N4O2	Pesticide TP (Cyromazine)
Deltamethric Acid	53179-78-5	C8H10Br2O2	Pesticide TP (Deltamethrin)
Ethyl (3-hydroxyphenyl)carbamate	7159-96-8	C9H11NO3	Pesticide TP (Desmedipham)
Diazoxon	962-58-3	C12H21N2O4P	Pesticide TP (Diazinon)

Table S20. Compound database for suspect screening (continued)

Compound Name	CAS	Molecular Formula	Category
2-Isopropyl-6-methyl-4-pyrimidinol	2814-20-2	C8H12N2O	Pesticide TP (Diazinon, Diazoxon)
Dicamba-desmethyl	3401-80-7	C7H4Cl2O3	Pesticide TP (Dicamba)
2,6-Dichlorobenzamide (BAM)	2008-58-4	C7H5Cl2NO	Pesticide TP (Dichlobenil, Fluopicolide)
N,N-Dimethyl-N'-phenylsulfamide (DMSA)	4710-17-2	C8H12N2O2S	Pesticide TP (Dichlofluanid)
N,N-Dimethylsulfamide	3984-14-3	C2H8N2O2S	Pesticide TP (Dichlofluanid, Tolylfluanid)
4-(2,4-Dichlorophenoxy)phenol	40843-73-0	C12H8Cl2O2	Pesticide TP (Diclofop-methyl)
Diclofop	40843-25-2	C15H12Cl2O4	Pesticide TP (Diclofop-methyl)
Dimethachlor Ethanesulfonic Acid (Dimethachlor ESA)	1231819-32-1	C13H19NO5S	Pesticide TP (Dimethachlor)
Dimethachlor Oxanilic Acid (Dimethachlor OA)	1086384-49-7	C13H17NO4	Pesticide TP (Dimethachlor)
Dimethenamid Ethanesulfonic Acid (Dimethenamid ESA)	205939-58-8	C11H17NO5S2	Pesticide TP (Dimethenamid)
Dimethenamid Oxanilic Acid (Dimethenamid OA)	380412-59-9	C12H17NO4S	Pesticide TP (Dimethenamid)
Disulfoton Sulfone	2497-06-5	C8H19O4PS3	Pesticide TP (Disulfoton)
Disulfoton Sulfoxide	2497-07-6	C8H19O3PS3	Pesticide TP (Disulfoton)
1-(3,4-Dichlorophenyl)3-methylurea (N-Demethoxylinuron)	3567-62-2	C8H8Cl2N2O	Pesticide TP (Diuron, Linuron, Neburon)
1-(3,4-Dichlorophenyl)urea (Diuron-desdimethyl)	2327-02-8	C7H6Cl2N2O	Pesticide TP (Diuron, Linuron, Neburon)
3,4-Dichloroaniline	95-76-1	C6H5Cl2N	Pesticide TP (Diuron, Linuron, Neburon, Propanil, Chlordimeform)
1-(2,4)-Dichloro-phenyl-2-imidazol-1-yl-ethanol	24155-42-8	C11H10Cl2N2O	Pesticide TP (Enilconazole (Imazalil))
O-Ethyl O-4-Nitrophenyl Phenylphosphonate (EPN-oxon)	2012-00-2	C14H14NO5P	Pesticide TP (O-Ethyl O-(4-nitrophenyl) Phenylphosphonothioate (EPN))
Ethiofencarb Sulfone	53380-23-7	C11H15NO4S	Pesticide TP (Ethiofencarb)
Ethiofencarb Sulfoxide	53380-22-6	C11H15NO3S	Pesticide TP (Ethiofencarb)
Ethofumesate-2-keto	26244-33-7	C11H12O5S	Pesticide TP (Ethofumesate)
Fenamiphos Sulfone	31972-44-8	C13H22NO5PS	Pesticide TP (Fenamiphos)
Fenamiphos Sulfoxide	31972-43-7	C13H22NO4PS	Pesticide TP (Fenamiphos)
3-Methyl-4-nitrophenol	2581-34-2	C7H7NO3	Pesticide TP (Fenitrothion)
Fenitrothion-oxon	2255-17-6	C9H12NO6P	Pesticide TP (Fenitrothion)
Fenoxaprop	95617-09-7	C16H12ClNO5	Pesticide TP (Fenoxaprop-ethyl)
Fenthion Sulfone	3761-42-0	C10H15O5PS2	Pesticide TP (Fenthion)
Fenthion Sulfoxide	3761-41-9	C10H15O4PS2	Pesticide TP (Fenthion)
1-(2-Chlorophenyl)-5(4H)-tetrazolinone	98377-35-6	C7H5ClN4O	Pesticide TP (Fentrazamide)
Fipronil Amide	205650-69-7	C12H6Cl2F6N4O2S	Pesticide TP (Fipronil)
Fipronil Sulfide	120067-83-6	C12H4Cl2F6N4S	Pesticide TP (Fipronil)
Fipronil Sulfone	120068-36-2	C12H4Cl2F6N4O2S	Pesticide TP (Fipronil)
Fipronil-desulfinyl	205650-65-3	C12H4Cl2F6N4	Pesticide TP (Fipronil)
4-(Trifluoromethyl)pyridine-3-carboxylic Acid	158063-66-2	C7H4F3NO2	Pesticide TP (Flonicamid)
Fluazifop	69335-91-7	C15H12F3NO4	Pesticide TP (Fluazifop-butyl)
2,2-Difluoro-1,3-benzodioxole-4-carboxylic Acid	126120-85-2	C8H4F2O4	Pesticide TP (Fludioxonil)
Flufenacet Ethanesulfonic Acid (Flufenacet ESA)	201668-32-8	C11H14FNO4S	Pesticide TP (Flufenacet)
Flufenacet Oxanilic Acid (Flufenacet OA)	201668-31-7	C11H12FNO3	Pesticide TP (Flufenacet)
1-(3-(Trifluoromethyl)phenyl)urea	13114-87-9	C8H7F3N2O	Pesticide TP (Fluometuron)
1-Methyl-3-(3-(trifluoromethyl)phenyl)urea	3032-40-4	C9H9F3N2O	Pesticide TP (Fluometuron)
3-(Trifluoromethyl)aniline	98-16-8	C7H6F3N	Pesticide TP (Fluometuron)
3-Chloro-5-(Trifluoromethyl)picolinic Acid	80194-18-9	C7H3ClF3NO2	Pesticide TP (Fluopicolide)
(E)-Deschlorophenyl Fluoxastrobin	852429-78-8	C15H13FN4O5	Pesticide TP (Fluoxastrobin)
Flupyrsulfuron-methyl	144740-53-4	C15H14F3N5O7S	Pesticide TP (Flupyrsulfuron)
3-(Methylphosphinico)propionic Acid	15090-23-0	C4H9O4P	Pesticide TP (Glufosinate)
3-Chloro-5-(trifluoromethyl)pyridin-2-ol	79623-37-3	C6H3ClF3NO	Pesticide TP (Haloxyfop)
Desnitro-imidacloprid	115970-17-7	C9H11ClN4	Pesticide TP (Imidacloprid)
Imidacloprid-olefin	115086-54-9	C9H8ClN5O2	Pesticide TP (Imidacloprid)
Imidacloprid-urea	120868-66-8	C9H10ClN3O	Pesticide TP (Imidacloprid)
3,5-Diiodo-4-hydroxybenzamide	2315-78-8	C7H5I2NO2	Pesticide TP (Ioxynil)
3,5-Diiodo-4-hydroxybenzoic Acid	618-76-8	C7H4I2O3	Pesticide TP (Ioxynil)
Isoiprodione	63637-89-8	C13H13Cl2N3O3	Pesticide TP (Iprodione)
4-Isopropylaniline	99-88-7	C9H13N	Pesticide TP (Isoproturon)
4-Methylphenethylamine	3261-62-9	C9H13N	Pesticide TP (Isoproturon)
Isoproturon-desmethyl	56046-17-4	C10H14N2O	Pesticide TP (Isoproturon)
Isoproturon-monodemethyl	34123-57-4	C11H16N2O	Pesticide TP (Isoproturon)
Kresoxim-methyl Acid	1007364-30-8	C17H17NO4	Pesticide TP (Kresoxim-methyl)
Malaoxon	1634-78-2	C10H19O7PS	Pesticide TP (Malathion)
Malathion Dicarboxylic Acid	1190-28-9	C6H11O6PS2	Pesticide TP (Malathion)
2-Amino-4-(methylsulfonyl)benzoic Acid	393085-45-5	C8H9NO4S	Pesticide TP (Mesotrione)
2-Nitro-4-methylsulfonylbenzoic Acid	110964-79-9	C8H7NO6S	Pesticide TP (Mesotrione)
Metamitron-desamino	36993-94-9	C10H9N3O	Pesticide TP (Metamitron)
Metazachlor Ethanesulfonic Acid (Metazachlor ESA)	172960-62-2	C14H17N3O4S	Pesticide TP (Metazachlor)
Metazachlor Oxanilic Acid (Metazachlor OA)	1231244-60-2	C14H15N3O3	Pesticide TP (Metazachlor)
Methidathion-oxon	39856-16-1	C6H11N2O5PS2	Pesticide TP (Methidathion)
Methiocarb Sulfone	2179-25-1	C11H15NO4S	Pesticide TP (Methiocarb)
Methiocarb Sulfoxide	2635-10-1	C11H15NO3S	Pesticide TP (Methiocarb)

Table S20. Compound database for suspect screening (continued)

Compound Name	CAS	Molecular Formula	Category
Methomyl-oxime	13749-94-5	C3H7NOS	Pesticide TP (Methomyl)
N-(2-Ethyl-6-methylphenyl)-L-alanine (CGA 50267)	82508-03-0	C12H17NO2	Pesticide TP (Metolachlor)
2-[((S)-1-Carboxyethyl)(2-ethyl-6-methylphenyl)amino]-2-oxo-ethanesulfonic Acid (NOA 413173)	1418095-19-8	C14H19NO6S	Pesticide TP (Metolachlor)
2-Chloro-N-(2-ethyl-6-methylphenyl)acetamide	32428-71-0	C11H14ClNO	Pesticide TP (Metolachlor, Acetochlor)
Metolachlor Ethanesulfonic Acid (Metolachlor ESA)	171118-09-5	C15H23NO5S	Pesticide TP (Metolachlor)
Metolachlor Oxanilic Acid (Metolachlor OA)	152019-73-3	C15H21NO4	Pesticide TP (Metolachlor)
Metolachlor-2-hydroxy	131068-72-9	C15H23NO3	Pesticide TP (Metolachlor)
Metolachlor-morpholinone	120375-14-6	C14H19NO2	Pesticide TP (Metolachlor)
N-(2-Ethyl-6-methylphenyl)-2-hydroxyacetamid (CGA 37735)	97055-05-5	C11H15NO2	Pesticide TP (Metolachlor)
N-(2-Ethyl-6-methylphenyl)-N-oxalo-L-alanine (CGA 357704)	1217465-10-5	C14H17NO5	Pesticide TP (Metolachlor)
Metribuzin-desamino	35045-02-4	C8H13N3OS	Pesticide TP (Metribuzin)
Metribuzin-diketo	52236-30-3	C7H11N3O2	Pesticide TP (Metribuzin)
6-Methyl-1,3,5-triazine-2,4(1H,3H)-dione	933-19-7	C4H5N3O2	Pesticide TP (Metsulfuron-methyl)
Methyl 2-(Aminosulfonyl)benzoate	57683-71-3	C8H9NO4S	Pesticide TP (Metsulfuron-methyl)
2-Amino-4-methoxy-6-methyl-1,3,5-triazine	1668-54-8	C5H8N4O	Pesticide TP (Metsulfuron-methyl, Thifensulfuron-methyl, Chlorsulfuron)
Molinate Sulfoxide	52236-29-0	C9H17NO2S	Pesticide TP (Molinate)
4-Chloroaniline	106-47-8	C6H6ClN	Pesticide TP (Monolinuron)
N-Desmethylnorflurazon	23576-24-1	C11H7ClF3N3O	Pesticide TP (Norflurazon)
Oxamyl-oxime	30558-43-1	C5H10N2O2S	Pesticide TP (Oxamyl)
Demeton-S-methyl Sulfone	17040-19-6	C6H15O5PS2	Pesticide TP (Oxydemeton-methyl)
Paraoxon	311-45-5	C10H14NO6P	Pesticide TP (Parathion)
Paraoxon-methyl	950-35-6	C8H10NO6P	Pesticide TP (Parathion-methyl)
Pentachloroaniline	527-20-8	C6H2Cl5N	Pesticide TP (Pentachloronitrobenzene (PCNB))
2,3-Dichloroanisole	1984-59-4	C7H6Cl2O	Pesticide TP (Pentachlorophenol (PCP))
3-Phenoxybenzyl Alcohol	13826-35-2	C13H12O2	Pesticide TP (Permethrin)
3-Phenoxybenzoic Acid	3739-38-6	C13H10O3	Pesticide TP (Permethrin, Deltamethrin)
Methyl N-(3-hydroxyphenyl)carbamate (MHPC)	13683-89-1	C8H9NO3	Pesticide TP (Phenmedipham)
Phorate Sulfone	2588-04-7	C7H17O4PS3	Pesticide TP (Phorate)
Phorate Sulfoxide	2588-03-6	C7H17O3PS3	Pesticide TP (Phorate)
Phoratoxon	2600-69-3	C7H17O3PS2	Pesticide TP (Phorate)
Phoratoxon Sulfone	2588-06-9	C7H17O5PS2	Pesticide TP (Phorate)
Phoratoxon Sulfoxide	2588-05-8	C7H17O4PS2	Pesticide TP (Phorate)
6-(3-(Trifluoromethyl)phenoxy)picolinic Acid	137640-84-7	C13H8F3NO3	Pesticide TP (Picolinafen)
Pirimicarb-desmethyl	30614-22-3	C10H16N4O2	Pesticide TP (Pirimicarb)
Pirimicarb-desmethyl-formamido	27218-04-8	C11H16N4O3	Pesticide TP (Pirimicarb)
2-(Diethylamino)-6-methyl-1H-pyrimidin-4-one	42487-72-9	C9H15N3O	Pesticide TP (Pirimiphos-methyl)
Propachlor Ethanesulfonic Acid (Propachlor ESA)	947601-88-9	C11H15NO4S	Pesticide TP (Propachlor)
Propachlor Oxanilic Acid (Propachlor OA)	70628-36-3	C11H13NO3	Pesticide TP (Propachlor)
Propazine-2-hydroxy	7374-53-0	C9H17N5O	Pesticide TP (Propazine, Prometryn)
Prothioconazole-desthio	120983-64-4	C14H15Cl2N3O	Pesticide TP (Prothioconazole)
Pyraflufen	129630-17-7	C13H9Cl2F3N2O4	Pesticide TP (Pyraflufen-ethyl)
Pyridafol	40020-01-7	C10H7ClN2O	Pesticide TP (Pyridate)
Pyrimethanil-4'-hydroxy	81261-84-9	C12H13N3O	Pesticide TP (Pyrimethanil)
Quizalofop	76578-12-6	C17H13ClN2O4	Pesticide TP (Quizalofop-ethyl, Propaquizafop)
Simazine-2-hydroxy	2599-11-3	C7H13N5O	Pesticide TP (Simazine)
2-Chloro-4-(methylsulfonyl)benzoic Acid	53250-83-2	C8H7ClO4S	Pesticide TP (Sulcotrione)
Tebuconazole-tert-butylhydroxy	212267-64-6	C16H22ClN3O2	Pesticide TP (Tebuconazole)
Terbufos Sulfone	56070-16-7	C9H21O4PS3	Pesticide TP (Terbufos)
Terbufos Sulfoxide	10548-10-4	C9H21O3PS3	Pesticide TP (Terbufos)
Terbumeton-desethyl	30125-64-5	C8H15N5O	Pesticide TP (Terbumeton)
Terbutryn-desethyl	30125-65-6	C8H15N5S	Pesticide TP (Terbutryn, Cybutryne)
Terbuthylazine-desethyl	30125-63-4	C7H12ClN5	Pesticide TP (Terbuthylazine)
Terbuthylazine-desethyl-2-hydroxy	66753-06-8	C7H13N5O	Pesticide TP (Terbuthylazine)
Terbuthylazine-2-hydroxy	66753-07-9	C9H17N5O	Pesticide TP (Terbuthylazine, Terbumeton, Terbutryn)
Thiacloprid-amide	676228-91-4	C10H11ClN4OS	Pesticide TP (Thiacloprid)
Clothianidin-urea	634192-72-6	C6H8ClN3OS	Pesticide TP (Thiamethoxam)
N-Desmethylthiamethoxam	171103-04-1	C7H8ClN5O3S	Pesticide TP (Thiamethoxam)
Thifensulfuron	79277-67-1	C11H11N5O6S2	Pesticide TP (Thifensulfuron-methyl)
Thiofanox Sulfone	39184-59-3	C9H18N2O4S	Pesticide TP (Thiofanox)
Thiofanox Sulfoxide	39184-27-5	C9H18N2O3S	Pesticide TP (Thiofanox)
2,4-Dichloro-3-methylphenol	17788-00-0	C7H6Cl2O	Pesticide TP (Tolclofos-methyl)
N,N-Dimethyl-N'-p-tolylsulphamide (DMST)	66840-71-9	C9H14N2O2S	Pesticide TP (Tolylfluanid)
Trifloxystrobin Acid	252913-85-2	C19H17F3N2O4	Pesticide TP (Trifloxystrobin)
Trinexapac	143294-89-7	C11H12O5	Pesticide TP (Trinexapac-ethyl)
2-(Trifluoromethyl)benzenesulfonamide	1869-24-5	C7H6F3NO2S	Pesticide TP (Tritosulfuron)
Benzoxiquine	86-75-9	C16H11NO2	Personal Care, Household & Industrial Chemical (Antimicrobial)
Triclocarban	101-20-2	C13H9Cl3N2O	Personal Care, Household & Industrial Chemical (Antimicrobial)

Table S20. Compound database for suspect screening (continued)

Compound Name	CAS	Molecular Formula	Category
Triclosan	3380-34-5	C12H7Cl3O2	Personal Care, Household & Industrial Chemical (Antimicrobial)
Benzylparaben	94-18-8	C14H12O3	Personal Care, Household & Industrial Chemical (Antimicrobial, Antifungal)
Benzylparaben	94-18-8	C14H12O3	Personal Care, Household & Industrial Chemical (Antimicrobial, Antifungal)
Butylparaben	94-26-8	C11H14O3	Personal Care, Household & Industrial Chemical (Antimicrobial, Antifungal)
Ethylparaben	120-47-8	C9H10O3	Personal Care, Household & Industrial Chemical (Antimicrobial, Antifungal)
Isobutylparaben	4247-02-3	C11H14O3	Personal Care, Household & Industrial Chemical (Antimicrobial, Antifungal)
Isopropylparaben	4191-73-5	C10H12O3	Personal Care, Household & Industrial Chemical (Antimicrobial, Antifungal)
Methylparaben	99-76-3	C8H8O3	Personal Care, Household & Industrial Chemical (Antimicrobial, Antifungal)
Propylparaben	94-13-3	C10H12O3	Personal Care, Household & Industrial Chemical (Antimicrobial, Antifungal)
Acesulfame	33665-90-6	C4H5NO4S	Personal Care, Household & Industrial Chemical (Artificial Sweetener)
Advantame	245650-17-3	C24H30N2O7	Personal Care, Household & Industrial Chemical (Artificial Sweetener)
Aspartame	22839-47-0	C14H18N2O5	Personal Care, Household & Industrial Chemical (Artificial Sweetener)
Neotame	165450-17-9	C20H30N2O5	Personal Care, Household & Industrial Chemical (Artificial Sweetener)
Saccharin	81-07-2	C7H5NO3S	Personal Care, Household & Industrial Chemical (Artificial Sweetener)
Sucralose	56038-13-2	C12H19Cl3O8	Personal Care, Household & Industrial Chemical (Artificial Sweetener)
1-(Methoxymethyl)-1H-benzotriazole	71878-80-3	C8H9N3O	Personal Care, Household & Industrial Chemical (Corrosion Inhibitor)
4-Methyl-1H-benzotriazole	29878-31-7	C7H7N3	Personal Care, Household & Industrial Chemical (Corrosion Inhibitor)
5,6-Dimethyl-1H-benzotriazole	4184-79-6	C8H9N3	Personal Care, Household & Industrial Chemical (Corrosion Inhibitor)
5-Chloro-1H-benzotriazole	94-97-3	C6H4ClN3	Personal Care, Household & Industrial Chemical (Corrosion Inhibitor)
5-Methyl-1H-benzotriazole	136-85-6	C7H7N3	Personal Care, Household & Industrial Chemical (Corrosion Inhibitor)
1H-Benzotriazole	95-14-7	C6H5N3	Personal Care, Household & Industrial Chemical (Corrosion Inhibitor)
Diphenylphosphinic Acid	1707-03-5	C12H11O2P	Personal Care, Household & Industrial Chemical (Flame Retardant)
Tris(1,3-dichloro-2-propyl) Phosphate (TDCPP)	13674-87-8	C9H15Cl6O4P	Personal Care, Household & Industrial Chemical (Flame Retardant))
Tris(1-chloro-2-propyl) Phosphate (TCPP)	13674-84-5	C9H18Cl3O4P	Personal Care, Household & Industrial Chemical (Flame Retardant)
Tris(2-chloroethyl) Phosphate (TCEP)	115-96-8	C6H12Cl3O4P	Personal Care, Household & Industrial Chemical (Flame Retardant)
Triphenylphosphine Oxide	791-28-6	C18H15OP	Personal Care, Household & Industrial Chemical (Flame Retardant)
Celestolide	13171-00-1	C17H24O	Personal Care, Household & Industrial Chemical (Fragrance)
Galaxolide (HHCB)	1222-05-5	C18H26O	Personal Care, Household & Industrial Chemical (Fragrance)
Tonalide (AHTN)	21145-77-7	C18H26O	Personal Care, Household & Industrial Chemical (Fragrance)
Versalide	88-29-9	C18H26O	Personal Care, Household & Industrial Chemical (Fragrance)
N,N-Diethyl-3-methylbenzamide (DEET)	134-62-3	C12H17NO	Personal Care, Household & Industrial Chemical (Insect Repellent) / Pesticide (Insect Repellent)
Ethyl Butylacetylaminopropionate	52304-36-6	C11H21NO3	Personal Care, Household & Industrial Chemical (Insect Repellent) / Pesticide (Insect Repellent)
Icaridin	119515-38-7	C12H23NO3	Personal Care, Household & Industrial Chemical (Insect Repellent) / Pesticide (Insect Repellent)
6:2 Fluorotelomersulfonic Acid	27619-97-2	C8H5F13O3S	Personal Care, Household & Industrial Chemical (PFAS)
N-Methyl Perfluorooctane Sulfonamide	31506-32-8	C9H4F17NO2S	Personal Care, Household & Industrial Chemical (PFAS)
Perfluorobutanesulfonic Acid	375-73-5	C4HF9O3S	Personal Care, Household & Industrial Chemical (PFAS)
Perfluorobutylsulfonamide	30334-69-1	C4H2F9NO2S	Personal Care, Household & Industrial Chemical (PFAS)
Perfluorobutyric Acid	375-22-4	C4HF7O2	Personal Care, Household & Industrial Chemical (PFAS)
Perfluorodecanesulfonic Acid	335-77-3	C10HF21O3S	Personal Care, Household & Industrial Chemical (PFAS)
Perfluorodecanoic Acid	335-76-2	C10HF19O2	Personal Care, Household & Industrial Chemical (PFAS)
Perfluoroheptanoic Acid	375-85-9	C7HF13O2	Personal Care, Household & Industrial Chemical (PFAS)
Perfluorohexanesulfonic Acid	355-46-4	C6HF13O3S	Personal Care, Household & Industrial Chemical (PFAS)
Perfluorohexanoic Acid	307-24-4	C6HF11O2	Personal Care, Household & Industrial Chemical (PFAS)
Perfluorononanoic Acid	375-95-1	C9HF17O2	Personal Care, Household & Industrial Chemical (PFAS)
Perfluorooctanesulfonamide	754-91-6	C8H2F17NO2S	Personal Care, Household & Industrial Chemical (PFAS)
Perfluorooctanesulfonic Acid	1763-23-1	C8HF17O3S	Personal Care, Household & Industrial Chemical (PFAS)
Perfluorooctanoic Acid	335-67-1	C8HF15O2	Personal Care, Household & Industrial Chemical (PFAS)
Perfluoropentanesulfonic Acid	2706-91-4	C5HF11O3S	Personal Care, Household & Industrial Chemical (PFAS)
Perfluoropentanoic Acid	2706-90-3	C5HF9O2	Personal Care, Household & Industrial Chemical (PFAS)
Perfluoropropanesulfonic Acid	423-41-6	C3HF7O3S	Personal Care, Household & Industrial Chemical (PFAS)
Benzyl Butyl Phthalate	85-68-7	C19H20O4	Personal Care, Household & Industrial Chemical (Plasticizer)
Bisphenol A	80-05-7	C15H16O2	Personal Care, Household & Industrial Chemical (Plasticizer)
Bisphenol AF	1478-61-1	C15H10F6O2	Personal Care, Household & Industrial Chemical (Plasticizer)
Bisphenol AP	1571-75-1	C20H18O2	Personal Care, Household & Industrial Chemical (Plasticizer)
Bisphenol B	77-40-7	C16H18O2	Personal Care, Household & Industrial Chemical (Plasticizer)
Bisphenol C	79-97-0	C17H20O2	Personal Care, Household & Industrial Chemical (Plasticizer)
Bisphenol E	2081-08-5	C14H14O2	Personal Care, Household & Industrial Chemical (Plasticizer)
Bisphenol F	620-92-8	C13H12O2	Personal Care, Household & Industrial Chemical (Plasticizer)
Bisphenol S	80-09-1	C12H10O4S	Personal Care, Household & Industrial Chemical (Plasticizer)
Bisphenol Z	843-55-0	C18H20O2	Personal Care, Household & Industrial Chemical (Plasticizer)
Butyl 2-Ethylhexyl Phthalate	85-69-8	C20H30O4	Personal Care, Household & Industrial Chemical (Plasticizer)
Diallyl Phthalate	131-17-9	C14H14O4	Personal Care, Household & Industrial Chemical (Plasticizer)
Dibutyl Phthalate	84-74-2	C16H22O4	Personal Care, Household & Industrial Chemical (Plasticizer)
Diethyl Phthalate	84-66-2	C12H14O4	Personal Care, Household & Industrial Chemical (Plasticizer)
Diisobutyl Phthalate	84-69-5	C16H22O4	Personal Care, Household & Industrial Chemical (Plasticizer)
Dimethyl Phthalate	131-11-3	C10H10O4	Personal Care, Household & Industrial Chemical (Plasticizer)
Dipentyl Phthalate	131-18-0	C18H26O4	Personal Care, Household & Industrial Chemical (Plasticizer)

Table S20. Compound database for suspect screening (continued)

Compound Name	CAS	Molecular Formula	Category
Dipropyl Phthalate	131-16-8	C14H18O4	Personal Care, Household & Industrial Chemical (Plasticizer)
Tributyl Phosphate	126-73-8	C12H27O4P	Personal Care, Household & Industrial Chemical (Plasticizer)
4-Methylbenzenesulfonamide	70-55-3	C7H9NO2S	Personal Care, Household & Industrial Chemical (Plasticizer)
N-Butylbenzenesulfonamide	3622-84-2	C10H15NO2S	Personal Care, Household & Industrial Chemical (Plasticizer)
N-Phenylbenzenesulfonamide	1678-25-7	C12H11NO2S	Personal Care, Household & Industrial Chemical (Plasticizer)
N-Ethyl-O-toluenesulfonamide	1077-56-1	C9H13NO2S	Personal Care, Household & Industrial Chemical (Plasticizer)
N-Ethyl-P-toluenesulfonamide	80-39-7	C9H13NO2S	Personal Care, Household & Industrial Chemical (Plasticizer)
Triisopropanolamine	122-20-3	C9H21NO3	Personal Care, Household & Industrial Chemical (Emulsifier, Stabilizer, Chemical Intermediate, Neutralizer)
Melamine	108-78-1	C3H6N6	Personal Care, Household & Industrial Chemical (Crosslinker) / Pesticide TP (Cyromazine) / Personal Care & Household Chemical TP (HMMM)
Hexa(methoxymethyl)melamine (HMMM)	3089-11-0	C15H30N6O6	Personal Care, Household & Industrial Chemical (Crosslinker; Tire rubber-derived)
1,1-Dicyclohexyl-3-phenylurea	5765-54-8	C19H28N2O	Personal Care, Household & Industrial Chemical (Antioxidant; Tire rubber-derived)
2,2,4-Trimethyl-1,2-dihydroquinoline	147-47-7	C12H15N	Personal Care, Household & Industrial Chemical (Antioxidant; Tire rubber-derived)
2-Mercapto-4-methylbenzimidazole	27231-33-0	C8H8N2S	Personal Care, Household & Industrial Chemical (Antioxidant; Tire rubber-derived)
2-Mercapto-5-methylbenzimidazole	27231-36-3	C8H8N2S	Personal Care, Household & Industrial Chemical (Antioxidant; Tire rubber-derived)
N-(1,3-Dimethylbutyl)-N'-phenyl-p-phenylenediamine (6PPD)	793-24-8	C18H24N2	Personal Care, Household & Industrial Chemical (Antioxidant; Tire rubber-derived)
N,N-Diphenylethylenediamine	1140-29-0	C14H16N2	Personal Care, Household & Industrial Chemical (Antioxidant; Tire rubber-derived)
N,N'-Diphenyl-p-phenylenediamine (DPPD)	74-31-7	C18H16N2	Personal Care, Household & Industrial Chemical (Antioxidant; Tire rubber-derived)
N-Cyclohexyl-N'-phenyl-p-phenylenediamine (CPPD)	101-87-1	C18H22N2	Personal Care, Household & Industrial Chemical (Antioxidant; Tire rubber-derived)
N-Isopropyl-N'-phenyl-p-phenylenediamine (IPPD)	101-72-4	C15H18N2	Personal Care, Household & Industrial Chemical (Antioxidant; Tire rubber-derived)
1,3-Di-o-tolylguanidine (DTG)	97-39-2	C15H17N3	Personal Care, Household & Industrial Chemical (Vulcanization Accelerator; Tire rubber-derived)
1,3-Diphenylguanidine (N,N′-diphenylguanidine; DPG)	102-06-7	C13H13N3	Personal Care, Household & Industrial Chemical (Vulcanization Accelerator; Tire rubber-derived)
2,4-Diamino-6-mercapto-1,3,5-triazine	767-17-9	C3H5N5S	Personal Care, Household & Industrial Chemical (Vulcanization Accelerator; Tire rubber-derived)
2-Mercaptobenzimidazole	583-39-1	C7H6N2S	Personal Care, Household & Industrial Chemical (Vulcanization Accelerator; Tire rubber-derived)
2-Phenyl-2-imidazoline	936-49-2	C9H10N2	Personal Care, Household & Industrial Chemical (Vulcanization Accelerator; Tire rubber-derived)
N-Cyclohexyl-1,3-benzothiazol-2-amine (NCBA)	28291-75-0	C13H16N2S	Personal Care, Household & Industrial Chemical (Vulcanization Accelerator; Tire rubber-derived)
N-Cyclohexyl-2-benzothiazolesulfenamide (CBS)	95-33-0	C13H16N2S2	Personal Care, Household & Industrial Chemical (Vulcanization Accelerator; Tire rubber-derived)
N-Tert-Butyl-2-benzothiazolesulfenamide	95-31-8	C11H14N2S2	Personal Care, Household & Industrial Chemical (Vulcanization Accelerator; Tire rubber-derived)
2-(Morpholinodithio)benzothiazole	95-32-9	C11H12N2OS3	Personal Care, Household & Industrial Chemical (Vulcanization Accelerator; Tire rubber-derived)
N,N-Diisopropylbenzothiazole-2-sulfenamide	95-29-4	C13H18N2S2	Personal Care, Household & Industrial Chemical (Vulcanization Accelerator; Tire rubber-derived)
4-(Benzothiazol-2-ylthio)-2,6-dimethylmorpholine	102-78-3	C13H16N2OS2	Personal Care, Household & Industrial Chemical (Vulcanization Accelerator; Tire rubber-derived)
3-Aminomethyl-3,5,5-trimethylcyclohexylamine	2855-13-2	C10H22N2	Personal Care, Household & Industrial Chemical (Tire rubber-derived)
1,3-Dicyclohexylurea (N,N′-dicyclohexylurea; DCU)	2387-23-7	C13H24N2O	Personal Care, Household & Industrial Chemical (Tire rubber-derived)
3-Cyclohexyl-1,1-dimethylurea (C-DMU)	31468-12-9	C9H18N2O	Personal Care, Household & Industrial Chemical (Tire rubber-derived)
6-Phenyl-1,3,5-triazine-2,4-diamine (Benzoguanamine)	91-76-9	C9H9N5	Personal Care, Household & Industrial Chemical (Tire rubber-derived)
Caprolactam	105-60-2	C6H11NO	Personal Care, Household & Industrial Chemical (Tire rubber-derived)
N-Cyclohexyl-N-methylcyclohexanamine (N,N-dicyclohexylmethylamine; DCA)	7560-83-0	C13H25N	Personal Care, Household & Industrial Chemical (Tire rubber-derived)
N-Cyclohexyl-N'-phenylurea (1-cyclohexyl-3-phenylurea; CPU)	886-59-9	C13H18N2O	Personal Care, Household & Industrial Chemical (Tire rubber-derived)
N-Ethylphthalimide	5022-29-7	C10H9NO2	Personal Care, Household & Industrial Chemical (Tire rubber-derived)
N,N'-Diethylcarbanilide (1,3-Diethyl-1,3-diphenylurea; D-DPU)	85-98-3	C17H20N2O	Personal Care, Household & Industrial Chemical (Tire rubber-derived)
2-((4-Methylpentan-2-yl)amino)-5-(phenylamino)cyclohexa-2,5-diene-1,4-dione (6PPD-quinone)	NA	C18H22N2O2	Personal Care, Household & Industrial Chemical TP (6PPD)
N-Phenyl-p-phenylenediamine (4-Aminodiphenylamine)	101-54-2	C12H12N2	Personal Care, Household & Industrial Chemical TP (6PPD)
2-Ethylhexyl 4-(dimethylamino)benzoate (ED-PABA)	21245-02-3	C17H27NO2	Personal Care, Household & Industrial Chemical (Sunscreen Agent)
2-Ethylhexyl 4-methoxycinnamate ((EHMC)	83834-59-7	C18H26O3	Personal Care, Household & Industrial Chemical (Sunscreen Agent)
2-Ethylhexyl Salicylate (EHS)	118-60-5	C15H22O3	Personal Care, Household & Industrial Chemical (Sunscreen Agent)
3-(4'-Methylbenzylidene)camphor (4-MBC)	36861-47-9	C18H22O	Personal Care, Household & Industrial Chemical (Sunscreen Agent)
2-Phenylbenzimidazole-5-sulfonic Acid (Ensulizole)	27503-81-7	C13H10N2O3S	Personal Care, Household & Industrial Chemical (Sunscreen Agent)
Benzophenone	119-61-9	C13H10O	Personal Care, Household & Industrial Chemical (Sunscreen Agent)
Benzophenone-1	131-56-6	C13H10O3	Personal Care, Household & Industrial Chemical (Sunscreen Agent)
Benzophenone-2	131-55-5	C13H10O5	Personal Care, Household & Industrial Chemical (Sunscreen Agent)
Benzophenone-3 (Oxybenzone)	131-57-7	C14H12O3	Personal Care, Household & Industrial Chemical (Sunscreen Agent)
Benzophenone-4	4065-45-6	C14H12O6S	Personal Care, Household & Industrial Chemical (Sunscreen Agent)
Benzophenone-6	131-54-4	C15H14O5	Personal Care, Household & Industrial Chemical (Sunscreen Agent)
Benzophenone-8	131-53-3	C14H12O4	Personal Care, Household & Industrial Chemical (Sunscreen Agent)
Benzophenone-9	143982-77-8	C15H14O11S2	Personal Care, Household & Industrial Chemical (Sunscreen Agent)
Butyl methoxydibenzoylmethane (BM-DBM)	70356-09-1	C20H22O3	Personal Care, Household & Industrial Chemical (Sunscreen Agent)
Homosalate	118-56-9	C16H22O3	Personal Care, Household & Industrial Chemical (Sunscreen Agent)
Octocrylene (OC)	6197-30-4	C24H27NO2	Personal Care, Household & Industrial Chemical (Sunscreen Agent)
4-Nonylphenol	104-40-5	C15H24O	Personal Care, Household & Industrial Chemical (Surfactant)
4-Nonylphenol Diethoxylate	20427-84-3	C19H32O3	Personal Care, Household & Industrial Chemical (Surfactant)
4-Nonylphenol Monoethoxylate	104-35-8	C17H28O2	Personal Care, Household & Industrial Chemical (Surfactant)
Octylphenol	949-13-3	C14H22O	Personal Care, Household & Industrial Chemical (Surfactant)
2-(Methylthio)benzothiazole	615-22-5	C8H7NS2	Personal Care, Household & Industrial Chemical (Vulcanization Accelerator)
2-Aminobenzothiazole	136-95-8	C7H6N2S	Personal Care, Household & Industrial Chemical (Vulcanization Accelerator)
2-Hydroxybenzothiazole	934-34-9	C7H5NOS	Personal Care, Household & Industrial Chemical (Vulcanization Accelerator)
2-Mercaptobenzothiazole	149-30-4	C7H5NS2	Personal Care, Household & Industrial Chemical (Vulcanization Accelerator)
Benzothiazole	95-16-9	C7H5NS	Personal Care, Household & Industrial Chemical (Vulcanization Accelerator)
2-(2-Hydroxy-5-methylphenyl)benzotriazole	2440-22-4	C13H11N3O	Personal Care, Household & Industrial Chemical (UV Stabilizer)

Table S20. Compound database for suspect screening (continued)

Compound Name	CAS	Molecular Formula	Category
1-Hydroxy-1H-benzotriazole	2592-95-2	C6H5N3O	Personal Care, Household & Industrial Chemical TP (1H-Benzotriazole)
1-Methyl-1H-benzotriazole	13351-73-0	C7H7N3	Personal Care, Household & Industrial Chemical TP (1H-Benzotriazole)
4-Hydroxy-1H-benzotriazole	26725-51-9	C6H5N3O	Personal Care, Household & Industrial Chemical TP (1H-Benzotriazole)
5-Methoxy-1H-benzotriazole	27799-91-3	C7H7N3O	Personal Care, Household & Industrial Chemical TP (1H-Benzotriazole)
1H-Benzotriazole-5-carboxylic Acid	23814-12-2	C7H5N3O2	Personal Care, Household & Industrial Chemical TP (5-Methyl-1H-benzotriazole)
2-(4-Morpholinyl)benzothiazole	4225-26-7	C11H12N2OS	Personal Care, Household & Industrial Chemical TP (Benzothiazole)
2-Methylbenzothiazole	120-75-2	C8H7NS	Personal Care, Household & Industrial Chemical TP (Benzothiazole)
Benzothiazole-2-sulfonic Acid	941-57-1	C7H5NO3S2	Personal Care, Household & Industrial Chemical TP (Benzothiazole)
3-(Diethylcarbamoyl)benzoic Acid (DEET O-Carboxylic Acid)	72236-23-8	C12H15NO3	Personal Care, Household & Industrial Chemical TP (DEET)
N,N-Diethyl-3-hydroxymethylbenzamide (O-Hydroxy-DEET)	72236-22-7	C12H17NO2	Personal Care, Household & Industrial Chemical TP (DEET)
N-Ethyl-m-toluamide	26819-07-8	C10H13NO	Personal Care, Household & Industrial Chemical TP (DEET)
Galaxolidone	507442-49-1	C18H24O2	Personal Care, Household & Industrial Chemical TP (Galaxolide)
Carbanilide (1,3-Diphenylurea)	102-07-8	C13H12N2O	Personal Care, Household & Industrial Chemical TP (Triclocarban)
Methyl Triclosan	4640-01-1	C13H9Cl3O2	Personal Care, Household & Industrial Chemical TP (Triclosan)

References

(1) Booij, K.; Chen, S.; Trask, J. R. POCIS calibration for organic compound sampling in small headwater streams. *Environmental Toxicology and Chemistry* **2020**, *39* (7), 1334-1342.
(2) Yang, L.; Jin, S.; Danielson, P.; Homer, C.; Gass, L.; Bender, S. M.; Case, A.; Costello, C.; Dewitz, J.; Fry, J.; Funk, M.; Granneman, B.; Liknes, G. C.; Rigge, M.; Xian, G. A new generation of the United States National Land Cover Database: Requirements, research priorities, design, and implementation strategies. *ISPRS Journal of Photogrammetry and Remote Sensing* **2018**, *146*, 108-123.
(3) New York State Water Resources Institute. *Septic Systems, New York State, 2011*. New York State Water Resources Institute, Ithaca, NY, https://cugir.library.cornell.edu/catalog/cugir-008164 (accessed April 15, 2022).
(4) New York State Department of Environmental Conservation. *Combined Sewer Overflows (CSOs): Beginning 2013*. New York State Department of Environmental Conservation, Albany, NY, https://data.ny.gov/Energy-Environment/Combined-Sewer-Overflows-CSOs-Beginning-2013/ephi-ffu6 (accessed April 15, 2022).
(5) New York State Department of Environmental Conservation. *NYS DEC Concentrated Animal Feeding Operations (nysdec_CAFO1)*. New York State Department of Environmental Conservation, Albany, NY, http://opdgig.dos.ny.gov/arcgis/rest/services/NYOPDIG/Erie/MapServer/5 (accessed April 15, 2022).
(6) New York State Department of Environmental Conservation. *Municipal Wastewater Treatment Plants*. New York State Department of Environmental Conservation, Albany, NY, https://data.ny.gov/Energy-Environment/Municipal-Wastewater-Treatment-Plants/rsuw-xxks (accessed April 15, 2022).
(7) New York State Department of Environmental Conservation. *Industrial Wastewater Treatment Plants*. New York State Department of Environmental Conservation, Albany, NY, https://data.ny.gov/Energy-Environment/Industrial-Wastewater-Treatment-Plants/ir2w-y295 (accessed April 15, 2022).
(8) U.S. Census Bureau. *TIGER/Line Geodatabases*. U.S. Census Bureau, Washington, D.C., https://www.census.gov/geographies/mapping-files/time-series/geo/tiger-geodatabase-file.html (accessed April 15, 2022).
(9) Blanchard, P. E.; Lerch, R. N. Watershed vulnerability to losses of agricultural chemicals: Interactions of chemistry, hydrology, and land-use. *Environmental Science & Technology* **2000**, *34* (16), 3315-3322.
(10) U.S. Census Bureau. *American Community Survey 2014-2018 5-Year Estimates*. U.S. Census Bureau, Washington, D.C., https://www.census.gov/programs-surveys/acs (accessed April 15, 2022).
(11) Wang, S.; Matt, M.; Murphy, B. L.; Perkins, M.; Matthews, D. A.; Moran, S. D.; Zeng, T. Organic micropollutants in New York lakes: A statewide citizen science occurrence study. *Environmental Science & Technology* **2020**, *54* (21), 13759-13770.
(12) Green, S. A.; Blough, N. V. Optical absorption and fluorescence properties of chromophoric dissolved organic matter in natural waters. *Limnology and Oceanography* **1994**, *39* (8), 1903-1916.
(13) Hudson, N.; Baker, A.; Reynolds, D. Fluorescence analysis of dissolved organic matter in natural, waste and polluted waters—a review. *River Research and Applications* **2007**, *23* (6), 631-649.
(14) Fellman, J. B.; Hood, E.; Spencer, R. G. M. Fluorescence spectroscopy opens new windows into dissolved organic matter dynamics in freshwater ecosystems: A review. *Limnology and Oceanography* **2010**, *55* (6), 2452-2462.
(15) Hansen, A. M.; Kraus, T. E. C.; Pellerin, B. A.; Fleck, J. A.; Downing, B. D.; Bergamaschi, B. A. Optical properties of dissolved organic matter (DOM): Effects of biological and photolytic degradation. *Limnology and Oceanography* **2016**, *61* (3), 1015-1032.
(16) Hu, C.; Muller-Karger, F. E.; Zepp, R. G. Absorbance, absorption coefficient, and apparent quantum yield: A comment on common ambiguity in the use of these optical concepts. *Limnology and Oceanography* **2002**, *47* (4), 1261-1267.
(17) Chin, Y.-P.; Aiken, G.; O'Loughlin, E. Molecular weight, polydispersity, and spectroscopic properties of aquatic humic substances. *Environmental Science & Technology* **1994**, *28* (11), 1853-1858.

(18) De Haan, H.; De Boer, T. Applicability of light absorbance and fluorescence as measures of concentration and molecular size of dissolved organic carbon in humic Lake Tjeukemeer. *Water Research* **1987**, *21* (6), 731-734.
(19) McKnight, D. M.; Boyer, E. W.; Westerhoff, P. K.; Doran, P. T.; Kulbe, T.; Andersen, D. T. Spectrofluorometric characterization of dissolved organic matter for indication of precursor organic material and aromaticity. *Limnology and Oceanography* **2001**, *46* (1), 38-48.
(20) Cory, R. M.; McKnight, D. M. Fluorescence spectroscopy reveals ubiquitous presence of oxidized and reduced quinones in dissolved organic matter. *Environmental Science & Technology* **2005**, *39* (21), 8142-8149.
(21) Zsolnay, A.; Baigar, E.; Jimenez, M.; Steinweg, B.; Saccomandi, F. Differentiating with fluorescence spectroscopy the sources of dissolved organic matter in soils subjected to drying. *Chemosphere* **1999**, *38* (1), 45-50.
(22) Ohno, T. Fluorescence inner-filtering correction for determining the humification index of dissolved organic matter. *Environmental Science & Technology* **2002**, *36* (4), 742-746.
(23) Halbedel, S.; Herzsprung, P. Short communication on "Differentiating with fluorescence spectroscopy the sources of dissolved organic matter in soils subjected to drying" [Zsolnay, A.; Baigar, E.; Jimenez, M.; Steinweg, B.; Saccomandi, F.; Chemosphere 38, 45–50, 1999]. *Chemosphere* **2020**, *239*, Article Number: 124818.
(24) Parlanti, E.; Wörz, K.; Geoffroy, L.; Lamotte, M. Dissolved organic matter fluorescence spectroscopy as a tool to estimate biological activity in a coastal zone submitted to anthropogenic inputs. *Organic Geochemistry* **2000**, *31* (12), 1765-1781.
(25) Wilson, H. F.; Xenopoulos, M. A. Effects of agricultural land use on the composition of fluvial dissolved organic matter. *Nature Geoscience* **2008**, *2*, 37-41.
(26) Huguet, A.; Vacher, L.; Relexans, S.; Saubusse, S.; Froidefond, J. M.; Parlanti, E. Properties of fluorescent dissolved organic matter in the Gironde Estuary. *Organic Geochemistry* **2009**, *40* (6), 706-719.
(27) Murphy, K. R.; Stedmon, C. A.; Graeber, D.; Bro, R. Fluorescence spectroscopy and multi-way techniques. PARAFAC. *Analytical Methods* **2013**, *5* (23), 6557-6566.
(28) Murphy, K. R.; Stedmon, C. A.; Wenig, P.; Bro, R. OpenFluor- an online spectral library of auto-fluorescence by organic compounds in the environment. *Analytical Methods* **2014**, *6* (3), 658-661.
(29) Ishii, S. K. L.; Boyer, T. H. Behavior of reoccurring PARAFAC components in fluorescent dissolved organic matter in natural and engineered systems: A critical review. *Environmental Science & Technology* **2012**, *46* (4), 2006-2017.
(30) Wünsch, U. J.; Bro, R.; Stedmon, C. A.; Wenig, P.; Murphy, K. R. Emerging patterns in the global distribution of dissolved organic matter fluorescence. *Analytical Methods* **2019**, *11* (7), 888-893.
(31) Wünsch, U. J.; Geuer, J. K.; Lechtenfeld, O. J.; Koch, B. P.; Murphy, K. R.; Stedmon, C. A. Quantifying the impact of solid-phase extraction on chromophoric dissolved organic matter composition. *Marine Chemistry* **2018**, *207*, 33-41.
(32) Murphy, K. R.; Timko, S. A.; Gonsior, M.; Powers, L. C.; Wünsch, U. J.; Stedmon, C. A. Photochemistry illuminates ubiquitous organic matter fluorescence spectra. *Environmental Science & Technology* **2018**, *52* (19), 11243-11250.
(33) Wünsch, U. J.; Murphy, K. R.; Stedmon, C. A. The one-sample PARAFAC approach reveals molecular size distributions of fluorescent components in dissolved organic matter. *Environmental Science & Technology* **2017**, *51* (20), 11900-11908.
(34) Wünsch, U. J.; Stedmon, C. A.; Tranvik, L. J.; Guillemette, F. Unraveling the size-dependent optical properties of dissolved organic matter. *Limnology and Oceanography* **2018**, *63* (2), 588-601.
(35) Lambert, T.; Bouillon, S.; Darchambeau, F.; Massicotte, P.; Borges, A. V. Shift in the chemical composition of dissolved organic matter in the Congo River network. *Biogeosciences* **2016**, *13* (18), 5405-5420.
(36) Wang, S.; Perkins, M.; Matthews, D. A.; Zeng, T. Coupling suspect and nontarget screening with mass balance modeling to characterize organic micropollutants in the Onondaga Lake–Three Rivers system. *Environmental Science & Technology* **2021**, *55* (22), 15215-15226.

(37) Schymanski, E. L.; Jeon, J.; Gulde, R.; Fenner, K.; Ruff, M.; Singer, H. P.; Hollender, J. Identifying small molecules via high resolution mass spectrometry: Communicating confidence. *Environmental Science & Technology* **2014**, *48* (4), 2097-2098.
(38) ChemAxon. *JChem for Excel (Version 20.20.0.732)*. ChemAxon, Budapest, Hungary, https://www.chemaxon.com (accessed March 1, 2021).
(39) Vrana, B.; Mills, G. A.; Dominiak, E.; Greenwood, R. Calibration of the Chemcatcher passive sampler for the monitoring of priority organic pollutants in water. *Environmental Pollution* **2006**, *142* (2), 333-343.
(40) Moschet, C.; Vermeirssen, E. L. M.; Singer, H.; Stamm, C.; Hollender, J. Evaluation of in-situ calibration of Chemcatcher passive samplers for 322 micropollutants in agricultural and urban affected rivers. *Water Research* **2015**, *71*, 306-317.
(41) Petrie, B.; Gravell, A.; Mills, G. A.; Youdan, J.; Barden, R.; Kasprzyk-Hordern, B. In situ calibration of a new chemcatcher configuration for the determination of polar organic micropollutants in wastewater effluent. *Environmental Science and Technology* **2016**, *50* (17), 9469-9478.
(42) Gallé, T.; Pittois, D.; Bayerle, M.; Braun, C. An immission perspective of emerging micropollutant pressure in Luxembourgish surface waters: A simple evaluation scheme for wastewater impact assessment. *Environmental Pollution* **2019**, *253*, 992-999.
(43) Dalton, R. L.; Pick, F. R.; Boutin, C.; Saleem, A. Atrazine contamination at the watershed scale and environmental factors affecting sampling rates of the polar organic chemical integrative sampler (POCIS). *Environmental Pollution* **2014**, *189*, 134-142.
(44) Criquet, J.; Dumoulin, D.; Howsam, M.; Mondamert, L.; Goossens, J.-F.; Prygiel, J.; Billon, G. Comparison of POCIS passive samplers vs. composite water sampling: A case study. *Science of the Total Environment* **2017**, *609*, 982-991.
(45) Plummer, R. E.; Hapeman, C. J.; Rice, C. P.; McCarty, G. W.; Schmidt, W. F.; Downey, P. M.; Moorman, T. B.; Douglas, E. A.; Strickland, T. C.; Pisani, O.; Bosch, D. D.; Elkin, K. R.; Buda, A. R. Method to evaluate the age of groundwater inputs to surface waters by determining the chirality change of metolachlor ethanesulfonic acid (MESA) captured on a polar organic chemical integrative sampler (POCIS). *Journal of Agricultural and Food Chemistry* **2020**, *68* (8), 2297-2305.
(46) Ahrens, L.; Daneshvar, A.; Lau, A. E.; Kreuger, J. Concentrations, fluxes and field calibration of passive water samplers for pesticides and hazard-based risk assessment. *Science of the Total Environment* **2018**, *637-638*, 835-843.
(47) Mazzella, N.; Lissalde, S.; Moreira, S.; Delmas, F.; Mazellier, P.; Huckins, J. N. Evaluation of the use of performance reference compounds in an Oasis-HLB adsorbent based passive sampler for improving water concentration estimates of polar herbicides in freshwater. *Environmental Science & Technology* **2010**, *44* (5), 1713-1719.
(48) U.S. Environmental Protection Agency. *ToxCast & Tox21 Summary Files for invitroDBv3.3*. National Center for Computational Toxicology, Research Triangle Park, NC, 2021. https://epa.figshare.com/articles/ToxCast_Database_invitroDB_/6062623 (accessed November 1, 2021).
(49) Blackwell, B. R.; Ankley, G. T.; Corsi, S. R.; DeCicco, L. A.; Houck, K. A.; Judson, R. S.; Li, S.; Martin, M. T.; Murphy, E.; Schroeder, A. L.; Smith, E. R.; Swintek, J.; Villeneuve, D. L. An "EAR" on environmental surveillance and monitoring: A case study on the use of exposure-activity ratios (EARs) to prioritize sites, chemicals, and bioactivities of concern in Great Lakes waters. *Environmental Science & Technology* **2017**, *51* (15), 8713-8724.
(50) Rose, L. D.; Akob, D. M.; Tuberty, S. R.; Corsi, S. R.; DeCicco, L. A.; Colby, J. D.; Martin, D. J. Use of high-throughput screening results to prioritize chemicals for potential adverse biological effects within a West Virginia watershed. *Science of the Total Environment* **2019**, *677*, 362-372.
(51) Corsi, S. R.; De Cicco, L. A.; Villeneuve, D. L.; Blackwell, B. R.; Fay, K. A.; Ankley, G. T.; Baldwin, A. K. Prioritizing chemicals of ecological concern in Great Lakes tributaries using high-throughput screening data and adverse outcome pathways. *Science of the Total Environment* **2019**, *686*, 995-1009.

(52) Rico, A.; de Oliveira, R.; de Souza Nunes, G. S.; Rizzi, C.; Villa, S.; López-Heras, I.; Vighi, M.; Waichman, A. V. Pharmaceuticals and other urban contaminants threaten Amazonian freshwater ecosystems. *Environment International* **2021**, *155*, 106702.
(53) Munz, N. A.; Burdon, F. J.; de Zwart, D.; Junghans, M.; Melo, L.; Reyes, M.; Schönenberger, U.; Singer, H. P.; Spycher, B.; Hollender, J.; Stamm, C. Pesticides drive risk of micropollutants in wastewater-impacted streams during low flow conditions. *Water Research* **2017**, *110*, 366-377.
